# Metabolites Link Intake of a Healthy Diet to Better Insulin and Glucose Homeostasis in the Microbiome and Insulin Longitudinal Evaluation Study (MILES)

**DOI:** 10.1016/j.cdnut.2024.104462

**Published:** 2024-09-26

**Authors:** Alexis C Wood, Danielle J Lee, Patricia A Sheridan, Elizabeth T Jensen, Gautam Ramesh, Alain G Bertoni, Stephen S Rich, Yii-Der I Chen, David M Herrington, Jerome I Rotter, Mark O Goodarzi

**Affiliations:** 1 USDA/Agricultural Research Service, Children’s Nutrition Research Center, Baylor College of Medicine, Houston, TX, United States; 2 Metabolon, Inc, Morrisville, NC, United States; 3 Department of Epidemiology and Prevention, Wake Forest School of Medicine, Winston-Salem, NC, United States; 4 School of Medicine, University of California, San Diego, La Jolla, CA, United States; 5 Center for Public Health Genomics, University of Virginia, Charlottesville, VA, United States; 6 Institute for Translational Genomics and Population Sciences, The Lundquist Institute for Biomedical Innovation and Department of Pediatrics, Harbor-UCLA Medical Center, Torrance, CA, United States; 7 Department of Internal Medicine, Section on Cardiovascular Medicine, Wake Forest School of Medicine, Winston-Salem, NC, United States; 8 Division of Endocrinology, Diabetes, and Metabolism, Department of Medicine, Cedars-Sinai Medical Center, Los Angeles, United States

**Keywords:** glucose homeostasis, insulin, glycemia, dysglycemia, oral glucose tolerance test, metabolites, diet patterns

## Abstract

**Background:**

Dietary quality has been linked to better glycemic control, but the precise molecular mechanisms giving rise to these associations are not fully understood.

**Objectives:**

To examine the association of metabolites associated with the intake of a healthy diet with measures of insulin/glucose homeostasis.

**Methods:**

Using cross-sectional data from 295 United States adults, the associations between 3 diet pattern scores and metabolome-wide metabolites were estimated via linear regression models, which controlled for demographic factors and health behaviors. Subsequently, the associations between the diet-related metabolites with 6 measures of glucose/insulin homeostasis were examined in similar models. A Bonferroni correction was applied to control the family-wise error rate at 5%.

**Results:**

Fifty-five metabolites were significantly associated with ≥1 diet score (all *P* < 1.7∗10^–5^). When these were summed into each of the 3 diet-specific metabolite summary scores, all 3 aggregate measures showed strong associations with 5 out of 6 measures of glucose/insulin homeostasis (*P* = 9.7∗10^–5^–4.1∗10^–13^).

**Conclusions:**

Adherence to a priori-defined “healthy diet” is associated with the plasma metabolites that, in turn, are associated with better glycemia. If the associations between replicated in future studies and examined using large-scale longitudinal data, the identified molecules could yield insights into mechanisms by which diet may support glucose and insulin homeostasis.

## Introduction

Diet is a critical lifestyle component of type 2 diabetes (T2D) risk. The American Diabetes Association promotes an overall healthy dietary pattern, with a focus on individual foods, as the most effective strategy for the primary and secondary prevention of T2D [1]. The Healthy Eating Index (HEI), which captures adherence to the USDA’s Dietary Guidelines for Americans, the Dietary Approaches to Stop Hypertension (DASH) diet, and a Mediterranean-style diet (AMED) all offer similar degrees of protection against T2D [2,3].

However, the precise molecular mechanisms by which these diet patterns convey protection are not well delineated. As the plasma metabolome measures an abundance of small molecules that reflect, in part, both dietary intake and metabolic health, metabolomic data offer promise for illuminating whether there are biological pathways between diet and indicators of metabolic health, such as dysglycemia [4]. To date, several studies have identified metabolites associated with either self-reported dietary intake [[5], [6], [7], [8], [9]] or dysglycemia [10]; however, whether metabolites associated with dietary intake are also associated with T2D has not been examined in a single unified analysis.

To better understand the pathways linking the intake of a healthy diet to better glucose and insulin homeostasis, the current analyses conducted metabolome-wide association studies (MWAS) with each of 3 healthy diet patterns (HEI, DASH, and AMED scores) and subsequently examined the associations of any metabolites associated with ≥1 healthy diet pattern with 6 measures of glucose/insulin homeostasis.

## Methods

### Participants

Participants were drawn from the Microbiome and Insulin Longitudinal Evaluation Study (MILES) [11], a cohort of 353 United States adults, ages 40–80 y. Exclusion criteria at enrollment included *1*) severe illness (e.g., actively treated cancer), *2*) recent (≤1 mo) use of microbiome-altering medications (e.g., antibiotics or metformin), *3*) current use of oral steroids, *4*) inflammatory bowel disease, *5*) previous surgery for weight loss, *6*) prescription therapy for chronic digestive issues such as constipation or diarrhea, *7*) current pregnancy, *8*) end-stage renal disease, *9*) heavy alcohol use (self-reported), and/or *10*) T2D determined either by self-reported history or point of care fasting glucose ≥126 mg/dL.

Eligible individuals were invited to arrive fasted (overnight fast) for participation in a clinic visit, which included an oral glucose tolerance test (OGTT). During the OGTT, participants completed questionnaires on health behaviors, and had anthropometric and clinical measures taken by trained study staff.

All subjects gave written informed consent prior to participation, and the study was approved by Institutional Review Boards at participating centers.

### Measures

#### Dietary intake

Habitual dietary intake over the past year was assessed using the most recent version of the Diet History Questionnaire (DHQ) at the time: the DHQ II [12]. Participants reported on their consumption frequency and average portion size for 132 foods over the past year. Habitual intake of 176 micro- and macro-nutrients and 124 foods and beverages, based on the USDA’s MyPyramid Equivalents Database and Food Patterns Equivalents Database. This information was used to calculate 3 dietary pattern scores: the HEI (the sum of 13 dietary components included in the 2015 Dietary Guidelines for Americans, each calculated as a ratio per 1000 kcals [13]); the DASH (sum of 8 quintile components [14]); and AMED score (the sum of 9 components cut at the sex-specific median, with a score of 1 added for males who report consuming 1–2 alcoholic drinks per day on average, and females who report ∼1 alcohol drink per day on average [15]).

Details on the components comprising individual scores are available in the Supplemental Methods. Twenty participants had diet data recoded to “missing” for reporting an implausible energy intake (600 ≤ kcals/d ≥ 6000 for males and 400 ≤ kcals/d ≥ 4000 for females; Supplemental Figure 1).

#### Glucose homeostasis traits

Venous blood was drawn before, 30 min after, and 120 min after ingestion of a 75-g glucose load. We focused on 6 traits in this study: fasting glucose, fasting insulin, fasting C-peptide, insulin sensitivity, insulin secretion, and disposition index (DI). Insulin sensitivity was calculated using the Matsuda Insulin Sensitivity Index, which correlates strongly with quantification by euglycemic clamp [16,17]. Insulin secretion was calculated as the ratio of the area under the curve (AUC) for insulin from baseline to 30 min over the corresponding AUC for glucose [17]. DI was calculated as the product of insulin sensitivity and insulin secretion.

#### Covariates

Height and weight were recorded by trained study staff using a stadiometer and a calibrated scale. Self-reported age, sex, race, education, and income levels were recorded via electronic questionnaires, with study staff available to answer questions. Physical activity was assessed via survey, and analyzed as the sum of light, moderate, and vigorous physical activity in metabolic equivalents minutes per week. Fifty-eight participants were missing covariate data and were excluded from analyses (Supplemental Figure 1).

#### Metabolome-wide metabolites

Plasma samples were assayed with untargeted ultra-HPLC coupled to tandem mass spectrometry (ThermoFisher Scientific Q-Exactive) and GC-MS (Orbitrap). Peak alignment and quality control were conducted on raw data via an in-house chemical reference library, with >3500 authentic standards identified by retention time/index), mass to charge spectral profile, in-source fragment, multimers formation, and fragmentation data. The Kyoto Encyclopedia of Genes and Genomes classifications were used to group known compounds into 9 classes (amino acids, carbohydrates, cofactors and vitamins, energy metabolites, lipids, nucleotide metabolites, peptides, and xenobiotics). Unknown chemical identities were tagged with a unique identifier according to an in-house spectral library of >7000 unknowns (denoted by “X” followed by the in-house number). Values were quantified for 1525 known and unknown compounds, of which 1500 with variance >0 were included in analyses.

The metabolites were analyzed in duplicate due to the availability of an updated metabolite panel during the study period. The coefficient of variation (CV) was calculated from the duplicate data and, due to the skewness of metabolomic data, estimated using the CV-analysis of variance (CV-ANOVA) model recommended by Røraas et al. [18]. The range of CVs for all metabolites was 0–135%, with a mean of 8.5 ± 17.7%, and the majority of metabolites (86.6%) having CVs <20%. (Supplemental Table 1).

### Analyses

All analyses were conducted using the latest version of R software (version 4.0.5.) [19].

#### Data preparation

All variables (including metabolites) were included after an inverse normal transformation with blom constant [20] to ensure an approximately normal distribution (Supplemental Table 1).

#### Relationships between diet pattern, metabolites, and glucose and insulin homeostasis traits

All relationships of interest were examined using linear regression models, which controlled for age, sex, race, smoking status, highest education level, income level, daily energy intake, and physical activity level as covariates. First, we conducted individual MWAS with each diet pattern score. Next, for each metabolite associated with ≥1 diet pattern in the MWAS, we examined the associations with each measure of glucose and insulin homeostasis. Subsequently, 3 diet-specific metabolite summary scores (MSSs) were created, in which standardized values of all metabolites showing a significant association with a given diet pattern were summed, with metabolite values reverse scored where the MWAS yielded a negative beta (β), and their associations with each measure of glucose and insulin homeostasis were estimated. Finally, J-tests were used to test whether the strength of associations with glucose and insulin homeostasis measures differed between diet pattern scores compared with diet-specific MSSs conducted using J-tests [21].

#### Sensitivity analysis

The MWAS was re-conducted, controlling for BMI (in kg/m^2^).

##### Significance

Within each set of analyses, significance was set using a Bonferroni correction for multiple testing, maintaining the family-wise error rate at 5%. For the MWAS, this was based on a spectral decomposition of eigenvalues to determine the effective number of independent tests; for other analyses, this was based on the number of associations conducted.

##### Model fidelity

Multicollinearity was assessed for all models via the variance inflation factor. No predictors in any model exceed our threshold of variance inflation factor >5.00 for multicollinearity [22].

## Results

Descriptive information is available in Table 1.

**TABLE 1:** Mean ± SD, or frequency (+ overall percentage; %) for demographic, health, and behavioral characteristics of the Microbiome and Insulin Longitudinal Evaluation Study participants.

Demographics	
Age, y	59.60 (9.07)
Sex, female, *n* (%)	218 (62%)
Race	
African American, *n* (%)	129 (37%)
Non-Hispanic White, *n* (%)	224 (63%)
Education	
High school or less, *n* (%)	33 (9%)
Some college, *n* (%)	102 (29%)
College, *n* (%)	122 (35%)
Postgraduate education, *n* (%)	96 (27%)
Health factors	
BMI, kg/m^2^	28.30 (7.43)
Fasting glucose, (mg/dL)	98.29 (10.92)
Fasting insulin, (μU/mL)	11.63 (9.22)
Fasting C-peptide, (ng/mL)	2.35 (1.14)
Insulin sensitivity index	5.05 (3.69)
Insulin secretion	0.44 (0.30)
Disposition index	1.67 (0.90)
Health behaviors	
AMED score	4.18 (1.86)
DASH score	24.01 (4.50)
HEI score	68.36 (10.58)
Average energy intake, kcals/d	1825.72 (915.10)
Physical activity, MET-min	2279.26 (2930.46)
Smoking status, current smoker, *n* (%)	64 (18%)

Abbreviations: AMED, a Mediterranean-style diet; DASH, Dietary Approaches to Stop Hypertension; HEI, Healthy Eating Index; MET-min, metabolic equivalent minutes; SD, standard deviation.

### MWAS

**FIGURE 1:**
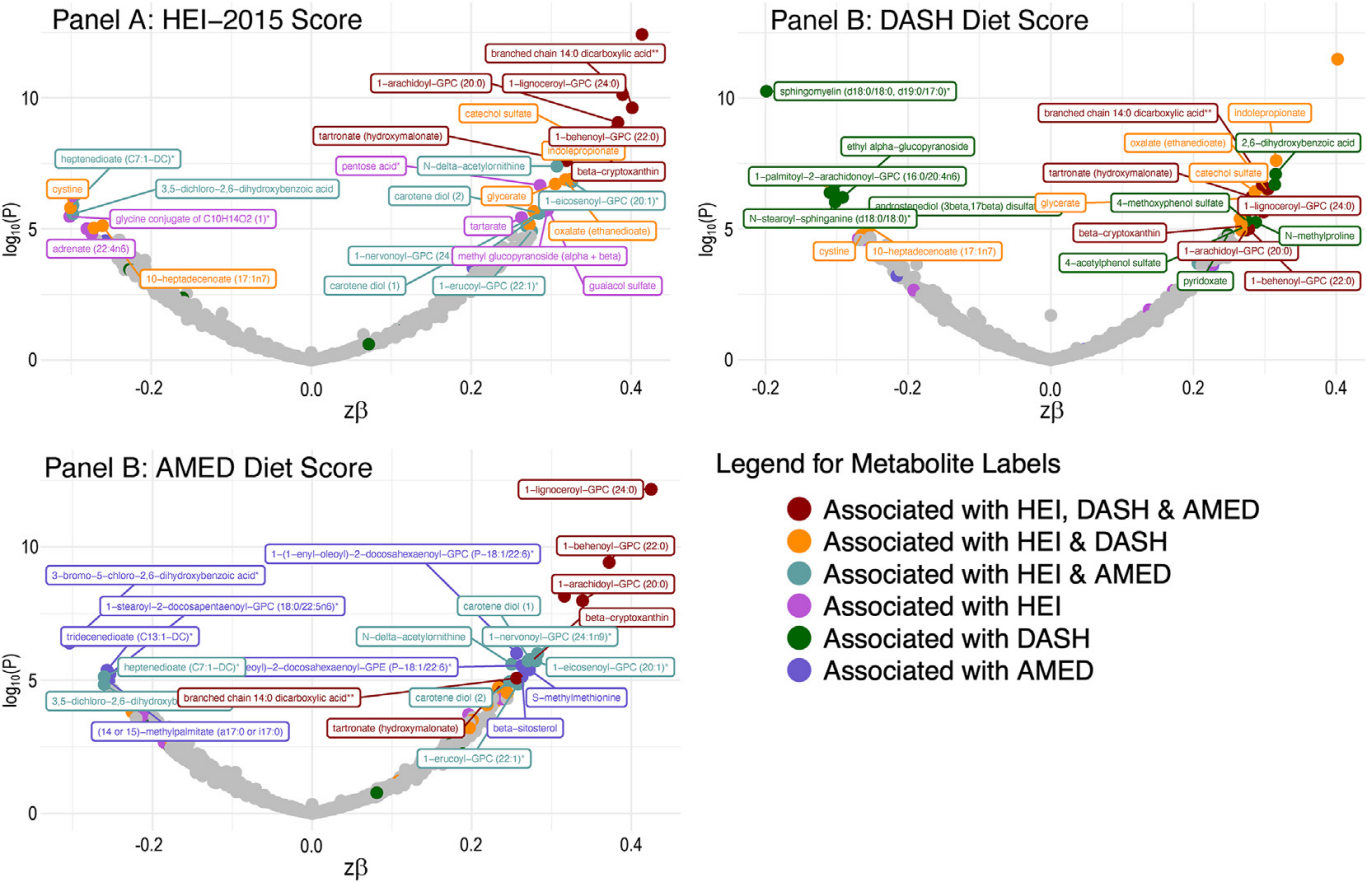
Standardized parameter estimates from metabolome-wide association studies with each of the Healthy Eating Index (HEI; A), the Dietary Approaches to Stop Hypertension (DASH; B), and a Mediterranean-style diet (AMED; C). Within each panel, only significant associations with a given diet pattern are annotated with the metabolite name. Across panels, label colors and metabolite colors denote significance at a Bonferroni corrected *P* < 1.7∗10^–5^. All models controlled for age, sex, race, physical activity, education level, energy intake (kcal/d) and smoking status.

**FIGURE 2:**
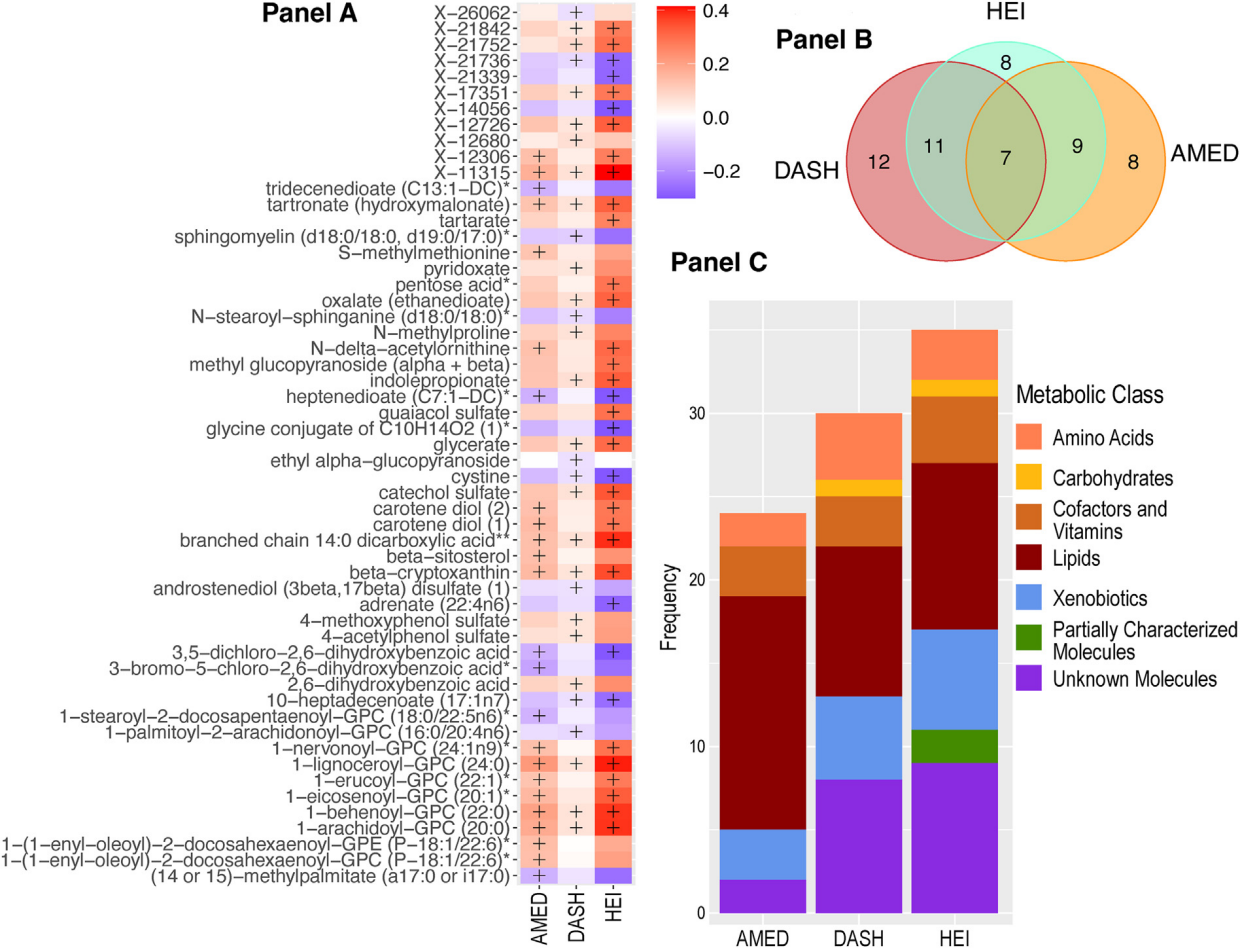
Exploration of significant metabolite-diet associations within and across each of the Healthy Eating Index 2015 (HEI-2015), the Dietary Approaches to Stop Hypertension (DASH), and a Mediterranean-style diet (AMED). (A) Associations with each of the 3 diet patterns for all metabolites showing ≥1 significant association with ≥1 diet pattern in a metabolome-wide association study (*P* < 1.7∗10^–5^ after a Bonferroni correction based on the effective number of independent tests). Note: + denotes significant associations (*P* < 1.7∗10^–5^). (B) Venn diagram of the number of significant associations (*P* < 1.7∗10^–5^ after a Bonferroni correction based on the effective number of independent tests) within and across metabolome-wide association studies for each of the 3 diet patterns. (C) A breakdown of the metabolite class membership accounting for each significant diet-metabolite association (*P* < 1.7∗10^–5^ after a Bonferroni correction based on the effective number of independent tests), arising from metabolome-wide association studies with each of the 3 diet patterns.

Full MWAS results are available in Supplemental Table 2. The MWAS revealed a total of 89 significant metabolite-diet associations (*N* = 35 for HEI, *N* = 30 for DASH, *N* = 24 for AMED; all *P* < 1.5∗10^–5^; Figure 1, Table 2). These encompassed 55 unique metabolites from 5 classes: lipids (*N* = 17), xenobiotics (*N* = 10), amino acids (*N* = 6), cofactors/vitamins (*N* = 5), and carbohydrates (*N* = 1), with 2 partially characterized molecules and 11 unknown molecules (Table 2). The mean correlation between the metabolites was r = 0.07, with a range of r = –0.72 to 0.95 (Supplemental Table 3, Supplemental Figure 2). When a dissimilarity matrix was computed using Euclidean distance and the Ward algorithm [23], the Duda-Hart stopping indicated that the metabolites clustered into 4 groups (Supplemental Figure 2). Seven (12.7%) metabolites were associated with all 3 dietary patterns (FIGURE 1, FIGURE 2, Table 2). Conversely, 28 metabolites (50% of known molecules) were significantly associated with 1 diet pattern only (FIGURE 1, FIGURE 2, Table 2). Controlling for BMI did not materially change results (Supplemental Table 4).

**TABLE 2:**
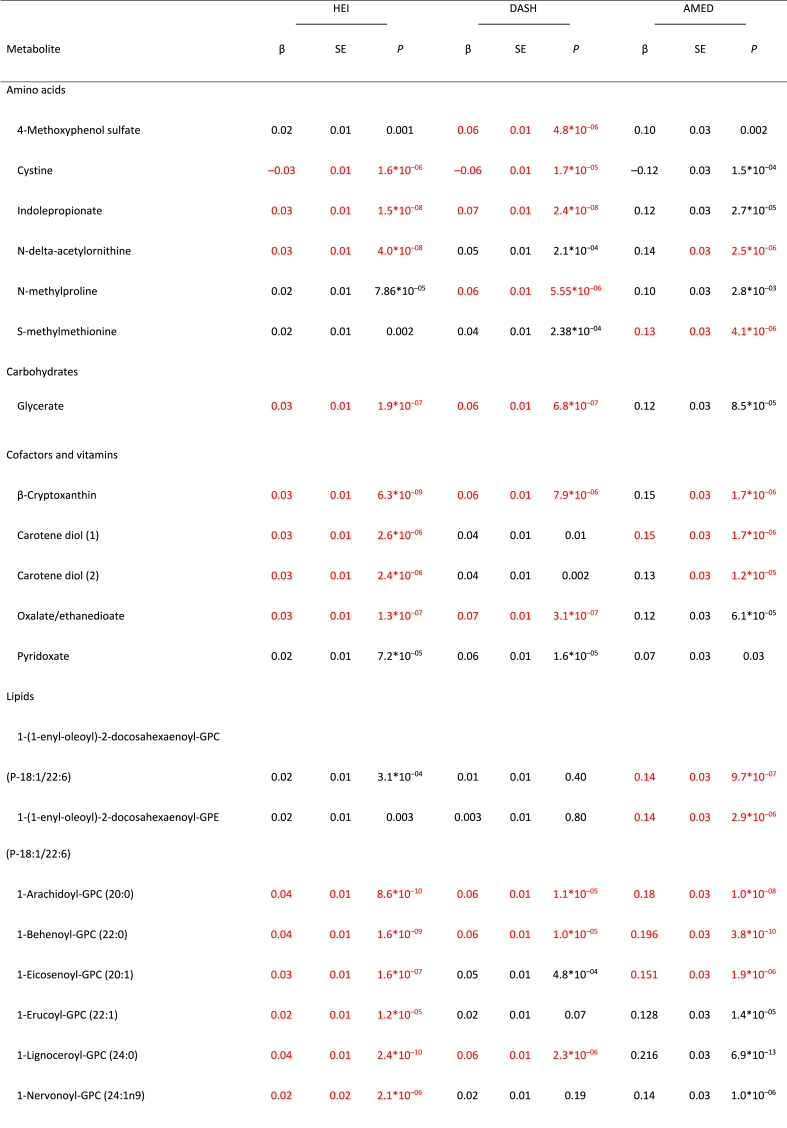

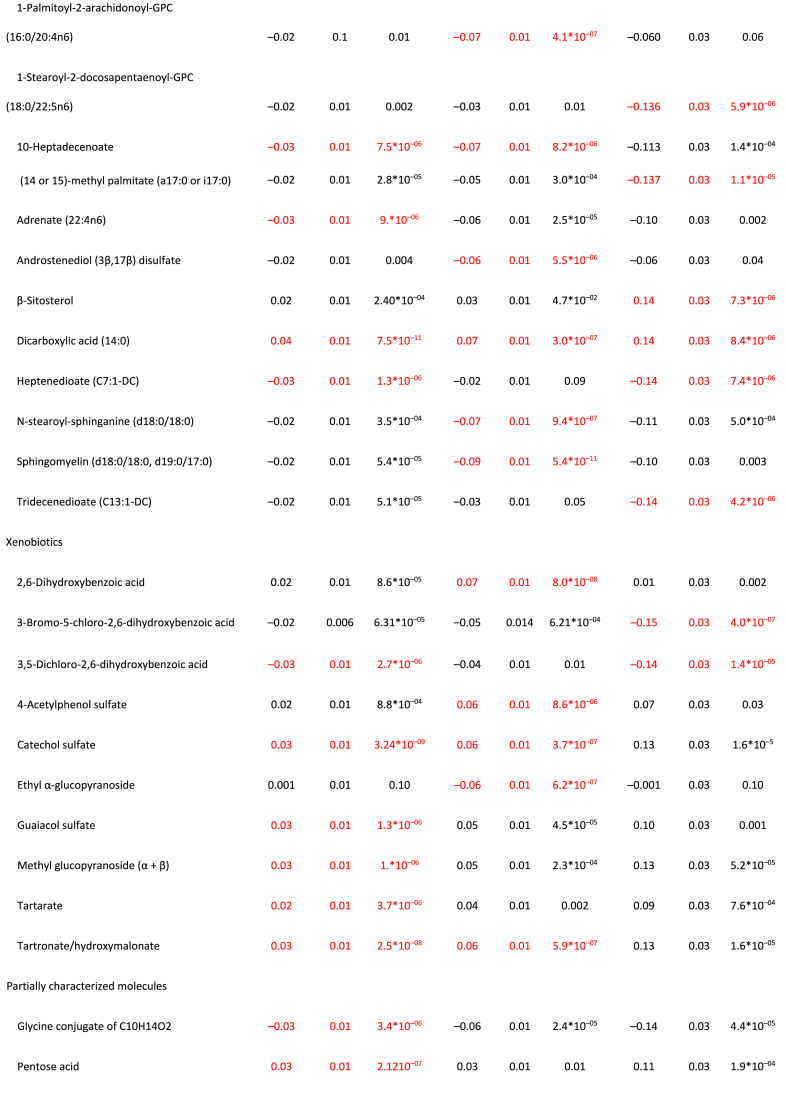

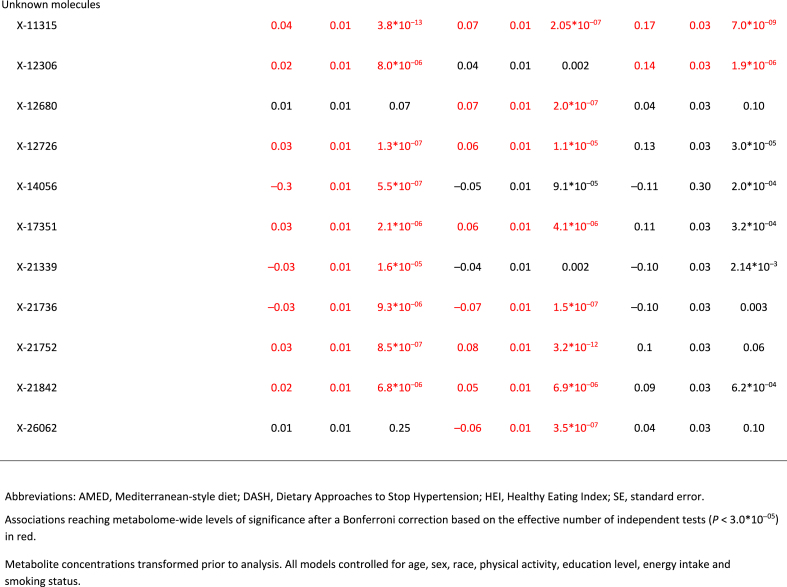
For significant metabolite associations in metabolome-wide associations studies with each the Healthy Eating Index-2015, the Dietary Approaches to Stop Hypertension, and a Mediterranean-style diet, standardized parameter estimates for the metabolite associations with all 3 healthy diet patterns.

### Associations between diet-specific MSSs and measures of glucose and insulin homeostasis

Of the 55 diet-related metabolites, 9 were also significantly associated with fasting glucose, 14 with fasting insulin, 14 with C-peptide, 18 with insulin sensitivity, 2 with insulin secretion, and 11 with DI (all *P* < 3.0∗10^–4^; Supplemental Table 5). Two metabolites were associated with all 6 measures of glucose and insulin homeostasis and, noting that insulin secretion showed very few associations with diet-related metabolites, a further 3 metabolites were associated with all measures of glucose and insulin homeostasis except insulin secretion (all *P* < 3.0∗10^–4^; Supplemental Table 5). Thirty-two molecules did not show a significant association with any measure of glucose and insulin homeostasis, although 5 showed associations with ≥1 measure of glucose and insulin homeostasis that approached significance (defined as: 1.0∗10^–4^> *P* > 3.0∗10^–4^; Supplemental Table 5).

When combined into diet-specific aggregate scores, each MSS was strongly correlated with the associated diet pattern score (r = 0.48–0.61, all *P* < 2.0∗10^–16^), and each diet score explained 31–43% of the variance in the respective MSS (43% in HEI MSS, 43% in DASH MSS, and 31% in AMED MSS). Cross-pattern scores were lower for diet scores (r = 0.51–0.62, all *P* < 2.0∗10^–16^; Supplemental Table 6) for MSSs (r = 0.80–0.93, all *P* < 2.0∗10^–16^; Supplemental Table 6).

**FIGURE 3:**
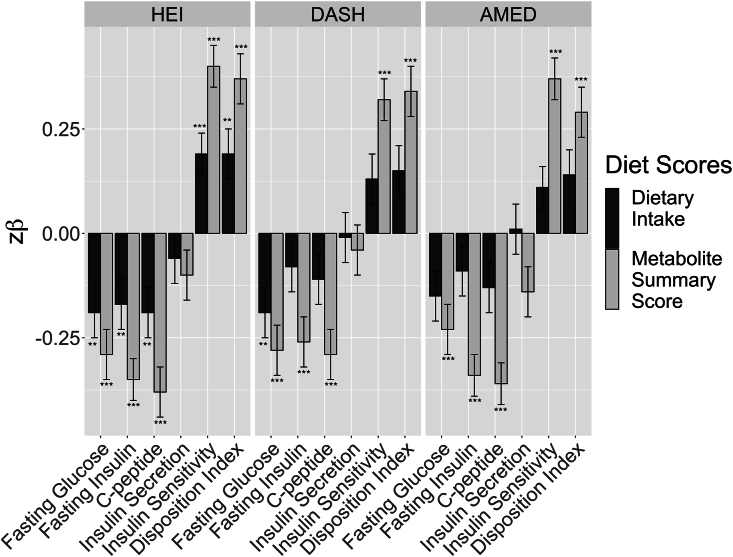
Standardized parameter estimates for associations of 6 glucose and insulin homeostasis traits with 3 healthy diet scores and with metabolite summary scores for each of the 3 healthy diet scores.

All 3 diet-specific MSS were significantly associated with 5 out of 6 measures of glucose/insulin homeostasis (all *P* = 9.7∗10^–5^ – 4.1∗10^–13^; Table 3). The proportion of variance in each glucose/insulin homeostasis measure explained by the respective MSS was 5–8% (Table 3), whereas the individual diet scores only explained ≤3% in glucose/insulin homeostasis (Table 3). J-tests revealed this difference was significant, with each diet-specific MSS explaining significantly more of the variance in glucose/inulin homeostasis than the respective diet scores (*P* = 0.003–9.8∗10^–12^; Table 3, Figure 3).

**TABLE 3:**
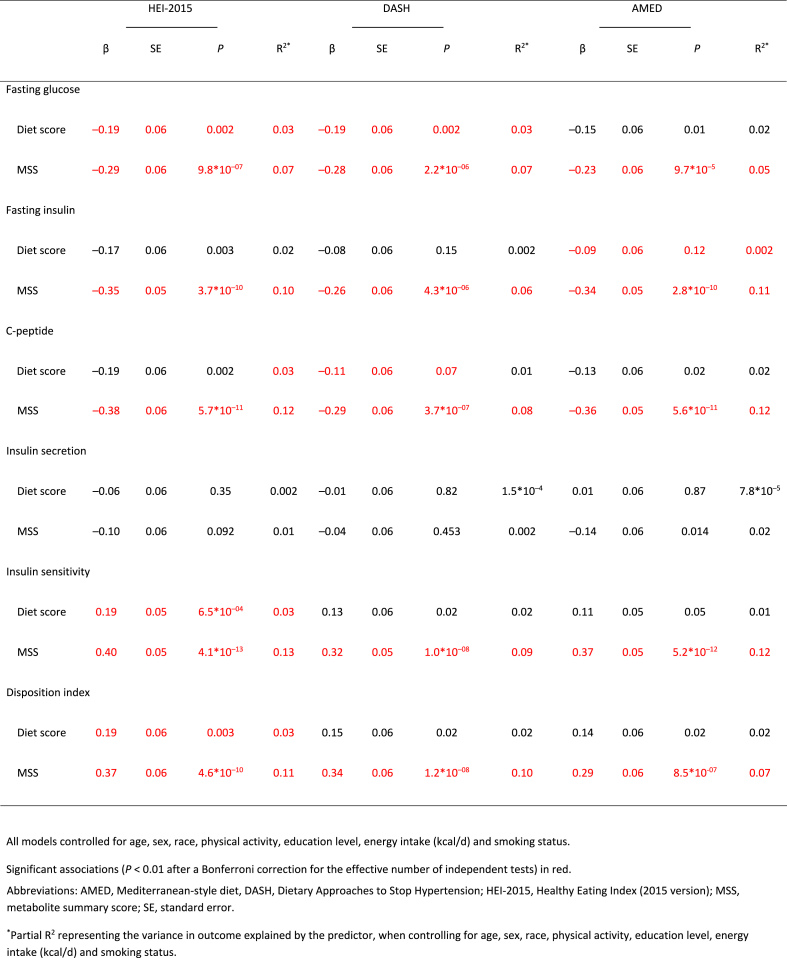
Standardized parameter estimates for associations between 6 glucose and insulin homeostasis traits with scores representing adherence to each of the 3 healthy diet patterns: the Healthy Eating Index-2015, the Dietary Approaches to Stop Hypertension, and a Mediterranean-style diet, and with metabolite summary scores for the same dietary patterns.

When additionally controlling for BMI, the associations with insulin and glucose homeostasis were attenuated, although the same pattern of relative association effect sizes for diet scores compared with MSSs was seen (Supplemental Table 7).

## Discussion

The current analyses sought to use untargeted metabolomic data to investigate whether molecules associated with 3 healthy diet patterns were also associated with glucose and insulin homeostasis. These metabolome-wide investigations indicated both shared and diet-specific diet-metabolite associations across multiple metabolite classes. When the metabolites associated with each diet pattern were aggregated together, the diet-specific MSSs showed strong and consistent relationships with better glucose/insulin homeostasis. These latter relationships were in the expected direction and only surprising for their magnitude, which was significantly greater than the observed diet-glycemia relationships.

Our analyses yielded 55 metabolites from multiple metabolite classes, including lipids, xenobiotics, amino acids, cofactors/vitamins, and carbohydrates, that were associated with ≥1 healthy dietary pattern in the MILES cohort. Although lipids and xenobiotics showed the largest number of associations (*N* = 20 and *N* = 10, respectively), our platform also quantified a greater number of metabolites from these classes relative to others. Overall, 3% of quantified amino acids, 9% of quantified carbohydrates, 12% of quantified vitamins/cofactors, 3% of quantified lipids, and 4% of quantified xenobiotics were significantly associated with ≥1 healthy diet pattern, making it difficult to ascertain whether 1 class of compounds was overrepresented in the associations. This number and pattern of diet-metabolite associations, including the relative representation of the various metabolite classes among the associations, is similar to previous MWAS, increasing our confidence that the 3 healthy diet patterns are associated with both shared and diet-specific metabolites across a breadth of metabolite classes [[5], [6], [7], [8], [9]].

Despite confidence in the pattern of diet-metabolite findings, we urge caution in interpreting specific metabolites as biomarkers of a given diet until our results are confirmed in independent replication analyses. Seventeen of the 68 diet-metabolite associations identified in the current analyses that included an identified (named) compound have been associated with the same diet pattern, in the same direction, in ≥1 prior study (6/25 known compounds associated with HEI, 9/22 associated with DASH, and 2/23 with AMED [[5], [6], [7], [8], [9]]). Three such metabolites are notable for showing associations with all 3 diet patterns: β-cryptoxanthin, a carotenoid, and a precursor of vitamin A [24], which is found in tropical and citrus fruits [25]; glycerate, a carbohydrate derived from fructose metabolism [26], and indolepropionate, an amino acid metabolite of tryptophan arising from the catabolism of phytochemicals and fiber [27,28]. Although these compounds may be interpreted as the strongest candidates for biomarkers of a healthy diet, they nonetheless should be seen as preliminary until subject to extensive additional replication efforts.

The extent to which dietary intake is reflected in the plasma metabolome is moderated by a complex set of intrinsic and extrinsic factors (e.g., [29]). Therefore, in addition to more universal biomarkers of dietary intake, we expected to identify molecules that more selectively correlated dietary intake in the MILES cohort as a function of cohort characteristics such as mean age, racial and ethnic diversity, geographical location, and the year of data collection. Thus, all molecules were investigated for associations with glucose and insulin homeostasis traits. Insulin secretion during the 2-h OGTT was notable for showing the least number of diet-related metabolite associations, with the 5 other measures showing similar numbers of associations (*N* = 9–18). Alongside the associations with self-reported diet patterns, this suggests that healthful diets generally have beneficial associations with tendencies toward insulin and glucose homeostasis rather than with any specific pathway. Across all 44 known compounds identified with ≥1 healthful diet, there was little evidence of prior associations with T2D or its clinical features. Among our diet-related lipids, 1-lignoceroyl-GPC (24:0) and sphingomyelin (d18:0/18:0, d19:0/17:0) have previously been associated with diet-related traits [30,31] and were associated with fasting insulin, C-peptide, and insulin sensitivity in our analysis. Thus, the identification associations with ≥1 measure of insulin and glucose homeostasis for 17 other known compounds [1-eicosenoyl-GPC (20:1), 1-erucoyl-GPC (22:1), 1-arachidoyl-GPC (20:0), N-stearoyl-sphinganine (d18:0/18:0), methyl glucopyranoside (α + β), cystine, 1-lignoceroyl-GPC (24:0), tartronate, 1-behenoyl-GPC (22:0), β-sitosterol, 1-nervonoyl-GPC, 1-nervonoyl-GPC (24:1n9), catechol sulfate, guaiacol sulfate, branched chain 14:0 dicarboxylic acid, glycerate, glycine conjugate of C10H14O2, 10-heptadecenoate (17:1n7), adrenate (22:4n6), (14 or 15)-methyl palmitate (a17:0 or i17:0)] represent novel associations that warrant further investigation and should be considered preliminary until replicated in independent cohorts. That 30 diet-related compounds were not associated with any of our measures of T2D risk was to be expected. For example, even though circulating carotenoids have been associated with better glucose homeostasis [[32], [33], [34]], β-cryptoxanthin, the carotenoid associated with all 3 diet patterns in the current analyses but not with any measure of insulin and glucose homeostasis, has not been previously associated with T2D, or its clinical factors.

Compared to individual compounds, the MSSs provided the most persuasive evidence that the bioactive compounds arising from dietary intake link diet to T2D. All 3 were strongly associated with glucose/insulin homeostasis, except for insulin secretion, in the expected direction. This pattern of results, that is, the relative association effect sizes for diet scores compared with diet-metabolite scores, remained when additionally controlling for BMI, although as expected from our previous analyses [34,35], the associations for both diet scores and metabolite scores were attenuated.

This pattern of associations, observed when comparing the size of associations of glucose/insulin homeostasis measures with the diet compared with diet-related metabolites (both individual metabolites and MSS), replicated a pattern of results seen in 3 of our prior investigations, whereby associations between health indicators and metabolomic correlates of food intake are orders of magnitude stronger than the associations of those same health measures and dietary intake [[35], [36], [37]]. Across a growing body of work, this observation has been replicated across different cohorts, different metabolomic assays, and different diet components and so could be considered robust [[35], [36], [37]]. However, why the magnitude of relationships with health differ is beyond the scope of the current investigation. One possibility is the reduction in measurement error for metabolomic quantification compared with diet self-assessment [38]. An additional possibility is that metabolite levels reflect multiple other environmental exposures or genetic vulnerabilities. Finally, the ability of metabolites to reflect the physiological effects of dietary intake at the individual level (i.e., after digestion, processing, absorption, and accounting for differences in food metabolism) could also drive stronger associations. To fully understand these differences, future research could explore how using more objective dietary measures affects the magnitude of these associations. Furthermore, examining specific characteristics of study populations, such as age or glycemic status, could shed light on how these factors influence associations between diet, metabolites, and health outcomes. Such investigations could offer insights into why metabolomic data appear to provide more robust indicators of diet-health relationships than traditional dietary assessments. Although our current study does not pinpoint the exact reasons for these differences, it underscores the significance of metabolomic information in enhancing our understanding of diet-related health impacts.

Our study benefited from a detailed characterization of glucose/insulin homeostasis ascertained via an OGTT, combined with an untargeted (untargeted with regard to specific compound classes) metabolomic approach. Our analyses yielded 55 diet-metabolite associations from multiple metabolite classes, including lipids, xenobiotics, amino acids, cofactors/vitamins, and carbohydrates, the majority of which represent the first associations of their kind in the literature and, therefore, require replication in further analyses. However, these advantages necessitated stringent corrections for the number of tests conducted, and our ability to identify diet-related metabolites was limited to those metabolites meeting (or exceeding) these thresholds. Although we attempted to mitigate any unwanted effects this could have on true associations through, for example, correcting based on the estimated effective number of independent as opposed to the number of metabolites, given that our study does not include replication attempts, we retained a Bonferroni correction to balance type I compared with type II errors. Systematic biases likely influenced the self-reported dietary intake data [38], and although our food frequency questionnaire (FFQ) captured a wide variety of food groups, it only included a limited number of individual foods. Despite adjustments for potential confounders, our observational study cannot rule out possible residual confounding, thus precluding causal inferences. In light of these differences, the novel diet-metabolite associations identified here should be treated with caution. The nature and extent of dietary intake reflected in the plasma metabolome are moderated by intrinsic and extrinsic factors (e.g., [29]), and thus, we expected that metabolites would be selectively correlated with dietary intake across different cohorts but not others as a function of cohort characteristics. The MILES cohort consists of older United States adults (mean age ∼60 y) who self-reported their race as “Black” or “White,” living within traveling distance of Wake Forest University (where the study visits were conducted), and all of whom were free from T2D. Given that factors such as age [29] and glycemia status [37] moderate how food is digested, it should not be assumed that the results of the current study would generalize to populations with different socio-demographic compositions. Although this makes replication challenging, it also highlights the importance of replication efforts, especially for the diet-metabolite associations, but also for all observations presented here.

Our results indicated that healthy diet patterns are associated with multiple shared and diet-specific plasma metabolites. When these metabolites are aggregated in diet-specific MSSs, the summary scores show strong associations with multiple traits relating to glucose and insulin homeostasis, such as lower fasting glucose, lower fasting insulin, lower C-peptide concentrations, higher insulin sensitivity scores, and higher DI. The associations between the MSSs and insulin and glucose homeostasis are markedly stronger than those of self-reported dietary intake with the same traits. This could suggest that individual differences in how healthy diets are metabolized may moderate the effects of a healthy diet pattern on health, and the extent to which differences in diet and diet-metabolite relationships modulate the effects of diet on T2D should be the subject of future investigations. However, in highlighting the potential importance of individual differences to the results of diet-metabolite, health investigations, the results also speak to needing caution with generalizations beyond the current study. Still, regardless of these future endeavors, this study provides persuasive evidence that the inclusion of metabolite data is an important addition to diet-health investigations.

## Author contributions

The authors’ responsibilities were as follows – ACW: designed the research; DJL, PS, AGB, SSR, Y-DIC, JIR, MOG: conducted the research; JIR, Y-DIC, PS: provided essential reagents or provided essential materials; ACW: analyzed the data and performed statistical analysis; ACW: wrote the article; DJL, MOG: provided critical input to revising the manuscript. ACW: had primary responsibility for final content; and all authors: read and approved the final manuscript.

## Funding

This study was supported in part by NIH grants from the National Institute of Diabetes and Digestive and Kidney Disease (R01-DK109588, P30-DK063491) and from the National Center for Advancing Translational Sciences (UL1TR001420, UL1TR001881). MOG was supported by the Eris M. Field Chair in Diabetes Research. ACW was supported, in part, by USDA/Agricultural Research Service cooperative agreement #58-3092-5-001 and has received funding from the Hass Avocado Board and the Beef Checkoff for analyses unrelated to the current study. The contents of this publication do not necessarily reflect the views or policies of the USDA, nor does mention of trade names, commercial products, or organizations imply endorsement by the United States Government.

## Data availability

Data described in the manuscript, code book, and analytic code will be made available upon request from the senior author (MOG) pending review by the study group and satisfactory completion of assurances.

## Conflict of interest

The authors report no conflicts of interest.
