# Supplementary material for: Metabolites Link Intake of a Healthy Diet to Better Insulin and Glucose Homeostasis in the Microbiome and Insulin Longitudinal Evaluation Study (MILES)
Source: Curr Dev Nutr. 2024 Sep 26;8(11):104462. doi: 10.1016/j.cdnut.2024.104462 (PMC11609643; doi:10.1016/j.cdnut.2024.104462)

Stronger Associations with Glucose Homeostasis for Metabolomic Biomarkers of Dietary Intake Compared to Self-Reported Intake in The Microbiome and Insulin Longitudinal Evaluation Study (MILES)

Supplementary Methods

Diet Score Components

Healthy Eating Index – 2015 version (HEI-15) score: Total HEI-15 score was created from 13 dietary components: (1) total fruit [5 points], (2) whole fruit [5 points], (3) total vegetables [5 points], (4) greens and beans [5 points], (5) whole grains [10 points], (6) dairy [10 points], (7) total protein foods [5 points], (7) seafood and plant proteins [5 points], (8) fatty acids [10 points], (9) refined grains [10 points], (10) sodium [10 points], (11) added sugars [10 points], and (12) saturated fats [10 points]. Each component is calculated as a ratio of each 1,000 kcals consumed, and the components summed (26). Total HEI-15 scores range from 0 to 100.

Dietary Approaches to Stop Hypertension (DASH) score: The following 8 components were quintiled, and summed: (1) fruit and fruit juice, (2) vegetables, (3) whole grains, (4) nuts and legumes, (5) low-fat dairy, (6) sodium, (7) red and processed meat, and (8) sugar-sweetened beverages (SSBs) [sodium, red and processed meat, and SSB components were reverse scored].

Alternative Mediterranean Diet (A-MED) score: The following 9 components were cut at the sex-specific median: (1) vegetables (2) legumes, (3) fruit (4) nuts, (5) whole grains, (6) fish, (7) red meat, (8) dairy and (9) saturated to unsaturated fat ratio. Scores for these components were summed, with red meat and dairy scores reversed before aggregation. A score of 1 for men who reported 1-2 alcoholic drinks per day (3.5-17.5 grams of ethanol) on average, and women who reported ~1 alcohol drink per day (3.5-10.5 grams of ethanol) on average was added to the summed component (28; 29).

Supplementary Table 1: Distribution of raw and transformed metabolites, the correlation between raw and transformed metabolite values, and coefficient of variation of raw metabolites

Supplementary Table 1: Distribution of raw and transformed metabolites, the correlation between raw and transformed metabolite values, and coefficient of variation of raw metabolites

Metabolite	Raw metabolite					Transformed metabolite		Correlation between Raw and Transformed Metabolite Values	Coefficient of Variation %
	Mean	Standard deviation	Skew	Kurtosis	Coefficient of Variation	Skew	Kurtosis		
carnitine	1.00	0.18	-0.27	0.43	17.63	0.00	-0.12	0.99	7.80E-01
3-phenylpropionate (hydrocinnamate)	1.33	1.31	-0.52	-0.48	98.96	0.12	-0.36	0.89	0.00E+00
phenylacetate	1.25	0.93	-0.36	-0.38	74.36	0.18	-0.43	0.90	0.00E+00
hippurate	1.38	1.39	-0.26	-0.03	100.68	0.00	-0.12	0.83	0.00E+00
xanthurenate	1.08	0.52	-0.63	0.90	48.11	0.05	-0.24	0.96	1.37E+01
suberate (C8-DC)	1.26	0.81	0.21	0.31	64.81	0.19	-0.44	0.83	1.04E+01
3-methyl-2-oxovalerate	1.05	0.34	-1.89	10.14	32.09	0.00	-0.12	0.98	0.00E+00
methionine sulfoxide	1.26	1.02	1.67	3.92	80.82	0.00	-0.12	0.73	1.20E+01
3-methylhistidine	1.50	1.43	-0.67	0.29	95.29	0.00	-0.12	0.91	3.04E+00
4-acetamidophenylglucuronide	59.04	177.95	2.00	3.09	301.86	1.42	0.83	0.61	0.00E+00
5-hydroxylysine	1.07	0.43	0.06	0.45	40.69	0.01	-0.15	0.94	0.00E+00
4-guanidinobutanoate	1.51	1.69	-0.07	-0.63	112.57	0.30	-0.52	0.77	9.14E+00
pimelate (C7-DC)	1.22	0.74	0.84	-0.42	61.31	0.89	-0.23	0.87	6.37E+00
glucuronate	1.19	0.94	-0.11	3.45	79.35	0.00	-0.12	0.75	0.00E+00
glycerol 3-phosphate	1.00	0.43	-1.02	0.59	43.21	0.08	-0.30	0.99	7.41E+00
imidazole lactate	1.03	0.36	-0.16	-0.02	35.39	0.00	-0.12	0.98	0.00E+00
kynurenine	1.03	0.29	-0.19	0.27	28.16	0.00	-0.12	0.99	0.00E+00
glycerophosphorylcholine (GPC)	1.00	0.27	-0.15	0.52	26.90	0.00	-0.12	0.98	2.66E+00
N-acetylglutamate	1.07	0.38	-2.36	7.04	35.39	0.07	-0.28	0.94	0.00E+00
tartarate	1.47	2.23	-0.33	-0.53	152.27	0.31	-0.52	0.62	1.32E+01
xanthosine	1.08	0.45	0.37	3.78	41.40	0.02	-0.18	0.80	
ribitol	1.05	0.32	0.31	1.25	30.17	0.02	-0.16	0.92	0.00E+00
2-isopropylmalate	2.19	6.16	0.78	2.28	281.16	0.12	-0.36	0.46	0.00E+00
glycodeoxycholate	1.73	2.18	-0.13	0.11	126.32	0.08	-0.30	0.77	1.46E+01
theophylline	1.14	0.99	-1.26	1.12	87.16	0.08	-0.30	0.92	0.00E+00
quinate	1.55	2.10	-0.48	-0.85	135.62	0.07	-0.28	0.84	1.83E+00
theobromine	1.59	1.87	-0.98	-0.10	117.80	0.23	-0.48	0.85	5.88E+00
gentisate	1.31	1.26	-0.35	-0.39	95.74	0.26	-0.50	0.82	0.00E+00
paraxanthine	1.22	1.05	-0.86	-0.18	85.71	0.17	-0.42	0.93	0.00E+00
indolelactate	1.07	0.43	0.15	0.81	40.66	0.00	-0.12	0.93	0.00E+00
3-indoxyl sulfate	1.11	0.57	-0.60	0.69	51.56	0.00	-0.12	0.97	0.00E+00
gamma-glutamylphenylalanine	1.19	0.60	1.69	3.30	50.64	0.00	-0.12	0.79	0.00E+00
4-methyl-2-oxopentanoate	1.02	0.33	-2.73	15.33	32.01	0.00	-0.12	0.99	0.00E+00
1,5-anhydroglucitol (1,5-AG)	1.01	0.31	-0.75	0.65	30.98	0.00	-0.12	1.00	8.34E-07
2-arachidonoylglycerol (20:4)	1.40	1.45	0.15	-1.17	103.72	0.58	-0.51	0.75	NA
1-palmityl-GPC (O-16:0)	1.10	0.56	0.68	1.42	50.98	0.00	-0.12	0.86	0.00E+00
1-stearoyl-2-arachidonoyl-GPI (18:0/20:4)	1.02	0.23	-0.35	-0.11	23.15	0.00	-0.12	1.00	9.36E-01
sphingosine 1-phosphate	1.07	0.20	0.54	0.21	18.80	0.00	-0.12	0.97	9.88E-01
1-stearoyl-2-oleoyl-GPS (18:0/18:1)	1.41	1.19	-0.04	-0.07	84.33	0.00	-0.12	0.88	4.60E+00
1-stearoyl-GPI (18:0)	1.14	0.51	0.35	1.06	44.84	0.00	-0.12	0.91	0.00E+00
1,2-dipalmitoyl-GPC (16:0/16:0)	1.01	0.17	-0.01	0.86	17.02	0.00	-0.12	0.98	0.00E+00
docosahexaenoate (DHA; 22:6n3)	1.35	1.02	0.22	0.50	75.47	0.00	-0.12	0.85	0.00E+00
1-myristoyl-2-palmitoyl-GPC (14:0/16:0)	1.16	0.71	-0.18	0.07	61.37	0.00	-0.12	0.94	0.00E+00
alpha-hydroxyisocaproate	1.06	0.41	-0.32	0.72	38.18	0.01	-0.13	0.97	0.00E+00
maleate	1.25	1.03	1.92	5.23	82.51	0.00	-0.12	0.69	7.66E+00
isovalerate (i5:0)	2.10	3.93	0.86	1.64	187.27	0.33	-0.53	0.62	3.75E+01
4-acetylphenol sulfate	2.38	12.68	0.78	2.96	533.89	0.12	-0.36	0.30	0.00E+00
2-hydroxyoctanoate	1.19	0.81	0.78	1.80	68.19	0.00	-0.12	0.80	4.94E+00
3-hydroxyoctanoate	1.13	0.51	-0.03	0.02	45.38	0.00	-0.12	0.96	9.71E-01
phenyllactate (PLA)	1.08	0.42	0.17	0.26	39.42	0.00	-0.12	0.95	0.00E+00
palmitoylcarnitine (C16)	1.02	0.27	-0.20	0.07	26.79	0.00	-0.12	0.99	0.00E+00
hexanoylcarnitine (C6)	1.22	1.04	0.73	2.52	85.53	0.01	-0.13	0.73	0.00E+00
theanine	0.81	0.48	13.03	168.76	59.05	13.03	168.78	0.94	4.80E+01
N-acetylaspartate (NAA)	0.96	0.23	-1.16	2.43	24.16	0.00	-0.12	1.00	5.51E+00
dehydroepiandrosterone sulfate (DHEA-S)	1.23	0.86	-0.33	0.33	70.16	0.00	-0.12	0.92	3.20E-07
acetylcarnitine (C2)	1.07	0.36	-0.20	-0.23	33.68	0.00	-0.12	0.98	7.72E-01
cysteine s-sulfate	1.08	0.56	-0.41	-0.30	52.04	0.01	-0.15	0.97	1.04E+01
1-palmitoylglycerol (16:0)	1.09	0.49	-0.24	0.86	45.21	0.04	-0.21	0.92	0.00E+00
tartronate (hydroxymalonate)	1.00	0.51	-1.69	3.49	51.05	0.00	-0.12	0.99	0.00E+00
oxalate (ethanedioate)	0.95	0.40	-1.10	0.99	41.71	0.00	-0.12	0.99	0.00E+00
erythritol	4.09	22.19	3.63	15.29	543.24	0.00	-0.12	0.35	0.00E+00
saccharin	3.41	11.98	0.41	-0.36	352.05	0.37	-0.54	0.46	0.00E+00
3-hydroxymyristate	1.30	0.81	-0.40	-0.06	62.72	0.01	-0.15	0.95	8.75E-08
iminodiacetate (IDA)	0.96	0.37	0.04	-0.95	38.51	0.00	-0.12	0.97	1.46E+00
1-oleoylglycerol (18:1)	1.04	0.62	0.13	0.23	59.57	0.00	-0.12	0.91	0.00E+00
3-methyl-2-oxobutyrate	1.00	0.29	-3.33	15.43	29.38	0.01	-0.13	0.98	0.00E+00
1,6-anhydroglucose	2.04	3.90	0.38	-0.56	191.34	0.44	-0.55	0.63	1.10E+01
2-oleoylglycerol (18:1)	1.07	0.59	0.41	-1.55	55.01	0.87	-0.25	0.94	3.38E-02
homoarginine	1.14	0.51	0.06	-0.21	44.37	0.00	-0.12	0.95	0.00E+00
homocitrulline	1.19	0.71	0.30	-0.08	60.33	0.00	-0.12	0.91	1.74E+01
pyroglutamylglutamine	1.02	0.33	-0.27	0.96	32.57	0.01	-0.15	0.97	2.33E+01
bradykinin	1.96	2.09	2.14	3.17	106.64	1.90	2.38	0.91	0.00E+00
glycylproline	8.78	22.67	1.60	1.44	258.70	1.27	0.48	0.63	NA
2-linoleoylglycerol (18:2)	1.09	0.52	-1.16	0.60	48.19	0.20	-0.45	0.96	2.11E+01
3-hydroxydecanoate	1.20	0.60	-0.07	-0.35	50.44	0.00	-0.12	0.95	1.10E-06
citramalate	1.49	1.48	-0.04	0.48	99.79	0.11	-0.35	0.77	0.00E+00
EDTA	0.99	0.15	0.01	0.89	14.82	0.00	-0.12	0.99	1.95E+00
N-acetylglycine	1.27	0.95	0.63	0.43	74.31	0.00	-0.12	0.84	2.16E+00
ribonate	1.04	0.44	0.45	4.38	42.57	0.00	-0.12	0.78	0.00E+00
threonate	1.08	0.43	-0.86	1.01	39.78	0.00	-0.12	0.99	4.44E+00
galactonate	1.29	1.12	-0.62	-0.41	86.46	0.22	-0.47	0.89	0.00E+00
beta-sitosterol	1.11	0.64	-0.73	-0.04	57.36	0.18	-0.43	0.94	NA
indoleacetate	1.20	0.84	0.52	0.81	69.59	0.00	-0.12	0.84	0.00E+00
1-linoleoylglycerol (18:2)	1.03	0.48	0.09	0.11	46.30	0.00	-0.12	0.93	3.52E+00
1-methylhistidine	1.08	0.41	0.01	0.20	37.59	0.01	-0.13	0.96	1.38E+01
butyrylcarnitine (C4)	1.24	1.45	-0.50	5.43	116.63	0.01	-0.15	0.59	0.00E+00
isobutyrylcarnitine (C4)	1.15	0.73	0.02	0.25	63.40	0.00	-0.12	0.90	1.47E+00
glycolithocholate	1.73	2.11	0.25	-0.81	122.00	0.38	-0.54	0.80	0.00E+00
androsterone sulfate	1.25	1.03	-0.81	2.01	82.31	0.00	-0.12	0.92	3.98E+00
indolepropionate	1.40	1.34	-1.21	6.32	96.33	0.01	-0.15	0.86	0.00E+00
N-(2-furoyl)glycine	2.88	7.81	0.70	1.57	271.47	0.23	-0.47	0.55	0.00E+00
trigonelline (N'-methylnicotinate)	1.59	1.99	-0.42	0.26	125.31	0.00	-0.12	0.83	0.00E+00
dodecanedioate (C12-DC)	1.39	1.46	0.67	1.04	104.73	0.00	-0.12	0.75	0.00E+00
N-acetyltyrosine	1.09	0.54	0.65	1.40	49.31	0.00	-0.12	0.86	1.00E+00
1,3-dimethylurate	1.16	0.92	-0.41	-1.07	79.11	0.34	-0.53	0.91	6.30E+00
3-methylxanthine	1.41	1.54	-0.68	0.24	109.33	0.11	-0.34	0.81	3.80E+00
3-hydroxylaurate	1.27	0.77	-0.58	0.04	61.21	0.00	-0.12	0.96	0.00E+00
pyridoxate	2.14	4.27	1.17	1.39	200.01	0.00	-0.12	0.59	0.00E+00
gamma-glutamylvaline	1.56	1.71	1.56	3.19	110.08	0.00	-0.12	0.70	0.00E+00
pyroglutamylglycine	1.87	2.06	0.37	-0.47	110.35	0.01	-0.13	0.83	0.00E+00
pyroglutamylvaline	1.17	0.51	-0.48	0.75	43.51	0.05	-0.24	0.94	7.13E+00
3-hydroxysebacate	1.33	1.28	-0.17	0.53	96.62	0.10	-0.33	0.78	5.85E+00
5-hydroxyhexanoate	1.18	0.59	0.16	-1.47	50.32	0.65	-0.46	0.95	
propionylglycine	1.20	0.74	-0.85	0.32	61.67	0.19	-0.44	0.91	2.89E+01

Supplementary Table 1: Distribution of raw and transformed metabolites, the correlation between raw and transformed metabolite values, and coefficient of variation of raw metabolites

butyrylglycine	1.21	0.65	-0.01	-0.72	54.25	0.36	-0.54	0.89	
2-methylbutyrylglycine	1.16	0.70	0.91	-0.14	60.65	0.88	-0.24	0.85	
propionylcarnitine (C3)	1.09	0.40	0.06	0.03	36.74	0.00	-0.12	0.96	0.00E+00
pro-hydroxy-pro	1.08	0.44	-0.53	0.86	40.60	0.05	-0.25	0.96	0.00E+00
3-hydroxy-2-ethylpropionate	1.07	0.40	-0.03	0.41	37.58	0.00	-0.12	0.96	0.00E+00
3-carboxy-4-methyl-5-propyl-2-furanpropanoate (CMPF)	2.10	3.22	-0.22	0.24	153.80	0.00	-0.12	0.77	0.00E+00
docosapentaenoate (n3 DPA; 22:5n3)	1.43	1.11	0.11	-0.06	77.65	0.00	-0.12	0.86	0.00E+00
docosadienoate (22:2n6)	1.19	0.63	-0.10	0.22	53.25	0.00	-0.12	0.92	0.00E+00
adrenate (22:4n6)	1.16	0.54	-0.17	0.35	46.74	0.00	-0.12	0.95	0.00E+00
10-undecenoate (11:1n1)	1.09	0.51	-0.45	1.10	47.14	0.00	-0.12	0.95	0.00E+00
4-imidazoleacetate	1.05	0.43	-0.94	-0.40	40.81	0.28	-0.51	0.99	3.17E+01
1-methyl-4-imidazoleacetate	1.04	0.35	-1.69	4.74	33.91	0.05	-0.24	0.97	0.00E+00
sebacate (C10-DC)	1.31	1.07	0.84	2.25	81.61	0.02	-0.16	0.75	2.74E+01
guanidinosuccinate	1.54	1.48	-0.18	-0.93	96.50	0.03	-0.20	0.91	6.13E+01
delta-tocopherol	1.36	1.04	-0.60	-0.10	76.59	0.12	-0.36	0.93	NA
I-urobilinogen	2.06	4.54	2.48	6.61	221.36	1.63	1.44	0.54	0.00E+00
stearidonate (18:4n3)	1.43	1.07	-0.13	-0.11	74.92	0.00	-0.12	0.91	0.00E+00
5-dodecenoate (12:1n7)	1.42	1.18	-0.14	1.33	83.46	0.00	-0.12	0.86	2.30E+00
octanoylcarnitine (C8)	1.30	1.23	0.71	1.68	95.16	0.00	-0.12	0.74	0.00E+00
tauro-beta-muricholate	2.37	7.54	0.83	0.26	318.11	0.59	-0.50	0.44	0.00E+00
decanoylcarnitine (C10)	1.28	1.11	0.56	1.28	86.74	0.00	-0.12	0.78	0.00E+00
N-acetylglutamine	1.11	0.50	0.46	0.89	45.33	0.00	-0.12	0.89	0.00E+00
N-acetyltryptophan	1.08	0.47	0.79	2.11	43.87	0.00	-0.12	0.85	0.00E+00
N-acetylphenylalanine	1.17	0.58	0.69	1.13	49.39	0.00	-0.12	0.87	0.00E+00
1-palmitoyl-GPC (16:0)	0.97	0.17	-0.27	0.02	17.55	0.00	-0.12	1.00	9.75E-02
1-margaroyl-GPC (17:0)	1.01	0.37	-0.32	0.47	36.85	0.01	-0.13	0.98	0.00E+00
N-acetylarginine	1.11	0.50	0.26	0.39	45.62	0.00	-0.12	0.92	0.00E+00
piperine	1.60	1.70	-0.62	0.23	106.70	0.00	-0.12	0.88	0.00E+00
campesterol	1.15	0.66	-0.35	0.35	57.44	0.04	-0.21	0.93	NA
myristoylcarnitine (C14)	1.13	0.79	-1.54	9.17	70.35	0.02	-0.16	0.80	0.00E+00
1-stearoyl-GPC (18:0)	0.97	0.22	-0.63	0.80	23.09	0.00	-0.12	1.00	1.92E+00
1-oleoyl-GPC (18:1)	1.00	0.29	-0.35	0.77	28.83	0.00	-0.12	0.99	0.00E+00
N-acetylthreonine	1.02	0.25	0.35	1.18	24.86	0.00	-0.12	0.95	0.00E+00
N-acetylisoleucine	1.05	0.34	0.17	0.90	32.18	0.00	-0.12	0.94	4.74E+00
10-nonadecenoate (19:1n9)	1.28	0.80	-0.30	0.00	62.93	0.00	-0.12	0.94	0.00E+00
10-heptadecenoate (17:1n7)	1.33	0.93	-0.32	-0.08	70.15	0.00	-0.12	0.93	2.55E-07
hyocholate	1.41	1.23	-0.13	-0.08	87.62	0.19	-0.44	0.84	4.00E+01
HWESASLLR	3.64	7.25	1.35	0.60	199.42	1.15	0.21	0.68	3.75E+01
epiandrosterone sulfate	1.28	1.06	-0.55	0.45	83.05	0.00	-0.12	0.89	0.00E+00
bradykinin, des-arg(9)	1.27	1.50	2.26	3.46	118.12	2.20	3.59	0.82	2.45E+01
N-acetylhistidine	1.07	0.73	0.68	6.87	68.47	0.00	-0.12	0.61	0.00E+00
gamma-glutamylglycine	1.32	0.78	0.62	0.50	58.78	0.00	-0.12	0.88	1.03E+01
gamma-glutamyltryptophan	1.05	0.28	0.16	0.44	27.00	0.00	-0.12	0.96	0.00E+00
stachydrine	1.93	2.43	-0.15	-0.72	126.04	0.00	-0.12	0.84	1.39E+01
alpha-hydroxyisovalerate	1.39	1.58	1.31	3.07	113.62	0.00	-0.12	0.68	0.00E+00
hydroxybupropion	1.00	0.91	5.53	29.17	91.11	5.23	26.16	0.96	1.25E+02
gamma-glutamylmethionine	1.13	0.40	-0.04	0.61	35.60	0.00	-0.12	0.96	5.40E+00
gamma-glutamylthreonine	1.32	1.04	-0.85	2.55	78.84	0.11	-0.34	0.77	0.00E+00
p-cresol sulfate	1.12	0.67	-1.64	4.78	59.71	0.00	-0.12	0.97	0.00E+00
erythronate*	1.07	0.28	0.57	1.53	25.88	0.00	-0.12	0.93	2.45E+00
Fibrinopeptide A*	9.24	36.70	2.01	2.67	397.85	1.71	1.69	0.48	4.13E+01
Fibrinopeptide A, des-ala(1)*	5.87	13.31	1.80	1.83	227.24	1.58	1.30	0.67	4.15E+01
Fibrinopeptide A, phosphono-ser(3)*	4.19	7.45	2.14	3.16	178.31	1.99	2.70	0.76	4.54E+01
XHWESASXXR*	2.32	2.77	2.19	3.46	119.66	1.79	1.97	0.88	NA
HWESASXX*	3.59	6.05	0.54	-0.36	168.58	0.24	-0.48	0.74	4.98E+01
HXGXA*	1.65	1.84	2.57	5.07	111.72	2.59	5.41	0.85	0.00E+00
salicyluric glucuronide*	13.31	39.97	1.06	1.03	300.76	0.24	-0.48	0.60	0.00E+00
eicosenoate (20:1)	1.34	0.87	-0.22	-0.16	65.01	0.00	-0.12	0.93	0.00E+00
linolenate [alpha or gamma; (18:3n3 or 6)]	1.35	1.00	-0.23	0.03	74.71	0.00	-0.12	0.90	0.00E+00
aconitate [cis or trans]	1.03	0.23	-0.40	2.71	22.34	0.00	-0.12	0.98	0.00E+00
1-myristoyl-GPC (14:0)	1.04	0.46	-0.12	0.00	44.04	0.00	-0.12	0.96	0.00E+00
1-arachidoyl-GPC (20:0)	1.03	0.44	-0.40	0.07	42.99	0.08	-0.29	0.97	1.23E+00
metoprolol acid metabolite*	59.77	150.28	5.44	31.37	251.80	4.82	22.02	0.66	7.95E+01
heme	4.00	14.34	0.38	2.09	358.99	0.04	-0.23	0.47	9.90E-06
stearoylcarnitine (C18)	1.02	0.32	-0.16	-0.43	31.37	0.00	-0.12	0.99	0.00E+00
laurylcarnitine (C12)	1.19	0.93	0.53	1.74	78.50	0.00	-0.12	0.77	0.00E+00
isovalerylcarnitine (C5)	1.17	0.54	-0.32	0.22	46.35	0.01	-0.15	0.97	2.37E+00
1-linoleoyl-GPC (18:2)	1.01	0.30	-0.39	1.12	29.57	0.00	-0.12	0.98	8.42E-01
7-methylxanthine	1.34	1.27	-0.26	-0.79	95.05	0.26	-0.50	0.85	1.19E+01
1,3,7-trimethylurate	1.60	2.12	-0.11	-0.71	132.51	0.24	-0.48	0.77	8.98E-06
3,7-dimethylurate	1.50	1.34	-0.19	-0.87	89.56	0.32	-0.53	0.87	7.89E+00
1,7-dimethylurate	1.16	0.99	-1.29	1.10	85.33	0.09	-0.32	0.93	0.00E+00
1-methylurate	1.14	0.98	-0.66	-0.23	86.25	0.03	-0.20	0.92	7.43E+00
5-acetylamino-6-formylamino-3-methyluracil	1.53	1.65	-0.41	-0.57	108.15	0.23	-0.47	0.85	2.12E+01
5-acetylamino-6-amino-3-methyluracil	1.24	1.08	-1.15	0.78	87.50	0.09	-0.32	0.93	0.00E+00
indolebutyrate	1.24	1.03	0.32	-0.38	83.16	0.34	-0.53	0.81	1.65E+01
1-methylxanthine	1.25	1.09	-0.80	-0.40	87.53	0.26	-0.50	0.89	0.00E+00
N1-methylinosine	1.11	0.44	-0.07	-0.20	39.39	0.03	-0.19	0.97	4.10E+00
N2,N2-dimethylguanosine	1.04	0.25	0.06	0.55	23.95	0.01	-0.15	0.97	0.00E+00
N4-acetylcytidine	1.05	0.41	-0.38	0.76	38.79	0.01	-0.13	0.97	0.00E+00
N6-carbamoylthreonyladenosine	1.04	0.24	0.44	0.38	23.15	0.00	-0.12	0.96	1.91E-08
orotidine	1.31	3.14	2.16	16.27	239.54	0.03	-0.19	0.32	0.00E+00
phenylacetylglutamine	1.13	0.62	-0.91	1.53	54.68	0.00	-0.12	0.97	2.79E+00
4-hydroxyhippurate	1.65	1.83	0.67	0.25	111.10	0.00	-0.12	0.79	0.00E+00
5,6-dihydrouridine	1.01	0.22	0.14	1.32	21.34	0.00	-0.12	0.96	0.00E+00
3-(3-amino-3-carboxypropyl)uridine*	1.02	0.24	-0.16	1.26	23.84	0.01	-0.13	0.97	0.00E+00
1-arachidonylglycerol (20:4)	1.24	0.86	-0.75	1.45	69.49	0.08	-0.30	0.89	9.12E+00
1-linolenoylglycerol (18:3)	1.14	0.61	0.07	-1.29	54.06	0.55	-0.52	0.91	0.00E+00
cysteine-glutathione disulfide	1.47	2.17	0.50	-0.77	147.34	0.75	-0.39	0.63	1.13E+01
5-methyluridine (ribothymidine)	0.99	0.18	-0.76	1.46	18.03	0.00	-0.12	0.99	0.00E+00
isovalerylglycine	1.22	0.84	-0.64	1.17	68.70	0.10	-0.32	0.87	2.63E+01
3-hydroxydodecanedioate*	1.48	1.77	0.16	0.25	119.94	0.14	-0.39	0.71	2.66E-06
7-methylguanine	1.05	0.27	0.40	1.56	25.46	0.00	-0.12	0.94	0.00E+00
1-stearoyl-GPE (18:0)	1.00	0.28	-0.04	-0.37	28.23	0.00	-0.12	0.98	0.00E+00
1-stearoyl-GPG (18:0)	1.02	0.43	-0.58	-0.18	42.37	0.19	-0.44	0.96	1.78E+00
N1-Methyl-2-pyridone-5-carboxamide	1.22	1.31	0.81	2.84	107.71	0.00	-0.12	0.65	0.00E+00
mead acid (20:3n9)	1.29	0.88	-0.77	1.23	68.65	0.08	-0.30	0.89	NA
gamma-glutamylisoleucine*	1.59	1.70	1.79	3.54	107.35	0.00	-0.12	0.70	0.00E+00
oleoylcarnitine (C18:1)	1.04	0.31	-0.19	-0.06	29.63	0.00	-0.12	0.99	1.41E+00
2-methylbutyrylcarnitine (C5)	1.10	0.53	-0.34	0.54	48.27	0.05	-0.24	0.94	1.59E+01
phenol sulfate	1.43	1.26	0.60	-0.01	88.16	0.00	-0.12	0.85	0.00E+00
1-palmitoleoyl-GPC (16:1)*	1.02	0.41	0.17	0.38	40.22	0.00	-0.12	0.94	0.00E+00
hexanoylglycine	1.29	1.02	-0.23	-1.02	79.26	0.42	-0.55	0.82	1.72E+01
glutamine_degradant*	1.28	0.93	0.18	-0.36	72.79	0.00	-0.12	0.90	0.00E+00
2-hydroxy-3-methylvalerate	1.32	1.25	0.82	1.75	94.90	0.00	-0.12	0.75	0.00E+00
homostachydrine*	1.30	1.27	-0.18	1.03	97.25	0.03	-0.20	0.77	0.00E+00
1-arachidonoyl-GPC (20:4n6)*	1.02	0.34	0.10	0.03	33.63	0.00	-0.12	0.97	0.00E+00

Supplementary Table 1: Distribution of raw and transformed metabolites, the correlation between raw and transformed metabolite values, and coefficient of variation of raw metabolites

1-dihomo-linolenoyl-GPC (20:3n3 or 6)*	1.04	0.37	-0.10	-0.15	35.10	0.00	-0.12	0.98	0.00E+00
1-dihomo-linoleoyl-GPC (20:2)*	1.01	0.34	-0.11	0.84	33.98	0.00	-0.12	0.96	0.00E+00
2-arachidonoyl-GPC (20:4)*	1.09	0.46	-1.26	4.79	42.59	0.03	-0.20	0.93	1.38E+00
2-oleoyl-GPC (18:1)*	1.04	0.39	0.06	0.68	37.17	0.00	-0.12	0.95	0.00E+00
2-linoleoyl-GPC (18:2)*	1.07	0.44	0.06	0.55	40.85	0.00	-0.12	0.94	0.00E+00
2-palmitoleoyl-GPC (16:1)*	1.10	0.62	-0.97	0.35	56.74	0.19	-0.44	0.93	0.00E+00
2-palmitoyl-GPC (16:0)*	1.00	0.29	0.05	0.51	28.78	0.00	-0.12	0.97	0.00E+00
2-myristoyl-GPC (14:0)*	1.02	0.46	-0.17	0.09	44.78	0.00	-0.12	0.96	0.00E+00
1-docosahexaenoyl-GPC (22:6)*	1.08	0.45	0.26	0.17	41.80	0.00	-0.12	0.94	0.00E+00
1-palmitoyl-GPE (16:0)	1.00	0.31	-0.03	-0.56	31.36	0.00	-0.12	0.98	0.00E+00
1-oleoyl-GPE (18:1)	1.07	0.43	0.03	0.62	39.76	0.00	-0.12	0.95	0.00E+00
1-linoleoyl-GPE (18:2)*	1.03	0.38	-0.28	0.52	36.77	0.00	-0.12	0.97	0.00E+00
1-arachidonoyl-GPE (20:4n6)*	1.04	0.26	0.07	-0.05	24.96	0.00	-0.12	0.98	0.00E+00
N-acetylcitrulline	1.51	1.67	0.05	0.15	110.22	0.03	-0.19	0.79	1.91E+01
2-hydroxypalmitate	1.07	0.32	0.03	0.39	30.04	0.00	-0.12	0.97	9.69E-01
docosapentaenoate (n6 DPA; 22:5n6)	1.19	0.64	-0.46	0.24	53.46	0.00	-0.12	0.96	1.36E+01
gulonate*	1.12	0.47	0.34	0.44	41.61	0.09	-0.31	0.91	4.27E+00
isobutyrylglycine	1.09	0.59	-0.12	-0.07	54.42	0.18	-0.43	0.88	NA
glutarylcarnitine (C5-DC)	1.12	0.52	-0.69	0.91	46.78	0.11	-0.34	0.93	3.86E+00
2-methylmalonylcarnitine (C4-DC)	1.06	0.32	0.24	-1.28	30.23	0.61	-0.49	0.97	NA
tiglylcarnitine (C5:1-DC)	1.09	0.49	-0.73	0.89	44.58	0.07	-0.28	0.97	2.69E+00
hydroquinone sulfate	1.38	1.35	-0.30	0.12	98.57	0.10	-0.32	0.83	5.45E+00
catechol sulfate	1.18	0.96	-0.49	1.64	81.47	0.00	-0.12	0.83	0.00E+00
cholesterol sulfate	1.04	0.31	-0.33	0.32	29.90	0.00	-0.12	0.99	0.00E+00
7-alpha-hydroxy-3-oxo-4-cholestenoate (7-Hoca)	1.07	0.35	0.35	0.06	32.32	0.00	-0.12	0.96	0.00E+00
tetradecanedioate (C14-DC)	1.30	1.18	0.49	1.36	90.64	0.01	-0.13	0.75	0.00E+00
hexadecanedioate (C16-DC)	1.20	0.94	0.54	1.13	78.01	0.00	-0.12	0.79	0.00E+00
octadecanedioate (C18-DC)	1.08	0.52	-1.67	4.96	48.46	0.04	-0.23	0.96	2.38E+00
undecanedioate (C11-DC)	1.05	0.28	0.33	1.46	26.92	0.02	-0.18	0.93	3.00E+01
glycerophosphoethanolamine	1.07	0.41	0.59	1.61	38.59	0.00	-0.12	0.90	4.20E+00
3-(3-hydroxyphenyl)propionate	2.48	5.89	0.28	-0.41	237.76	0.27	-0.50	0.58	2.63E+01
ectoine	1.48	1.62	-0.57	0.60	109.72	0.10	-0.32	0.80	0.00E+00
2-oleoyl-GPE (18:1)*	1.08	0.58	-0.20	0.88	54.04	0.01	-0.13	0.88	1.56E+01
2-palmitoyl-GPE (16:0)*	1.03	0.46	-0.36	0.79	44.54	0.02	-0.16	0.95	3.46E+00
1-arachidonoyl-GPI (20:4)*	1.08	0.38	0.28	0.23	35.26	0.00	-0.12	0.95	3.66E+00
1-palmitoyl-GPI (16:0)	1.17	0.63	0.00	0.53	54.07	0.01	-0.13	0.92	1.39E-07
glycolithocholate sulfate*	1.21	0.94	-0.53	0.65	77.82	0.00	-0.12	0.92	0.00E+00
taurolithocholate 3-sulfate	1.39	1.33	-0.96	2.04	95.67	0.02	-0.16	0.88	0.00E+00
deoxycarnitine	1.04	0.27	-3.12	26.51	26.18	0.00	-0.12	0.98	5.72E-01
N6-succinyladenosine	1.05	0.37	-1.08	3.28	35.35	0.04	-0.21	0.95	2.46E+01
1-ribosyl-imidazoleacetate*	1.04	0.40	-0.21	0.29	38.11	0.02	-0.18	0.96	0.00E+00
2-arachidonoyl-GPE (20:4)*	1.10	0.52	-0.14	1.03	47.49	0.02	-0.18	0.90	9.81E+00
leucylleucine	1.86	2.24	0.40	-0.26	120.43	0.27	-0.51	0.79	1.36E+01
N2-acetyllysine	1.00	0.38	1.87	1.94	37.67	1.95	2.53	0.97	4.92E+01
alpha-hydroxycaproate	1.18	0.69	1.07	0.13	58.74	1.04	0.01	0.84	NA
3,4-dihydroxybutyrate	1.07	0.33	0.53	0.64	31.15	0.00	-0.12	0.94	1.04E-07
indoleacetylglutamine	1.36	1.49	-0.07	0.66	109.78	0.06	-0.26	0.76	4.73E+00
hexanoylglutamine	1.26	0.99	-0.08	-0.01	78.55	0.00	-0.12	0.88	0.00E+00
N6-acetyllysine	1.01	0.18	0.31	0.44	17.46	0.00	-0.12	0.98	0.00E+00
dihomo-linolenate (20:3n3 or n6)	1.15	0.51	-0.33	-0.11	44.42	0.00	-0.12	0.98	0.00E+00
mannitol/sorbitol	1.89	2.85	1.35	1.68	151.17	0.00	-0.12	0.67	0.00E+00
tryptophan betaine	1.54	1.93	-0.67	0.65	125.71	0.01	-0.13	0.82	0.00E+00
4-vinylphenol sulfate	1.54	1.84	-0.09	-0.19	119.55	0.00	-0.12	0.82	0.00E+00
4-ethylphenylsulfate	2.54	5.98	0.78	1.09	235.86	0.00	-0.12	0.59	0.00E+00
thymol sulfate	3.74	6.86	0.14	-0.55	183.97	0.00	-0.12	0.75	1.15E+01
3-methyladipate	1.20	0.88	-0.15	-0.23	73.30	0.23	-0.48	0.85	3.95E+00
pyrraline	1.07	0.65	-0.65	0.69	60.77	0.04	-0.23	0.94	5.40E+00
2-linoleoyl-GPE (18:2)*	1.08	0.56	-0.40	1.02	51.78	0.01	-0.15	0.92	0.00E+00
1-oleoyl-GPI (18:1)	1.11	0.57	0.01	0.35	51.13	0.01	-0.13	0.92	0.00E+00
1-linoleoyl-GPI (18:2)*	1.10	0.48	0.10	0.31	43.64	0.00	-0.12	0.93	2.52E+00
1-palmitoleoyl-GPE (16:1)*	1.32	1.08	-0.13	-0.45	82.07	0.20	-0.45	0.88	1.34E+01
1-palmitoleoyl-GPI (16:1)*	1.55	2.25	0.72	-0.16	145.59	0.68	-0.44	0.63	2.51E+01
desmethylnaproxen	2.19	5.13	4.67	23.53	234.49	3.77	12.95	0.60	1.28E-04
desmethylnaproxen sulfate	7.77	24.16	3.15	8.83	311.42	2.79	6.52	0.55	5.91E+01
2-hydroxyacetaminophen sulfate*	8.51	16.08	2.04	2.96	189.22	1.54	1.18	0.77	1.95E-05
2-methoxyacetaminophen sulfate*	5.45	12.41	2.62	5.74	227.89	2.18	3.48	0.64	4.05E+01
2-methoxyacetaminophen glucuronide*	3.05	6.16	2.96	7.53	202.32	2.67	5.82	0.68	0.00E+00
3-(cystein-S-yl)acetaminophen*	1.44	0.88	4.42	19.08	61.64	4.09	15.46	0.94	2.66E+01
o-cresol sulfate	2.47	3.56	0.28	-0.53	144.59	0.07	-0.28	0.80	1.35E+01
dimethylarginine (SDMA + ADMA)	1.02	0.16	-0.31	0.39	16.04	0.00	-0.12	1.00	0.00E+00
gamma-glutamylalanine	1.63	1.47	0.23	-0.72	90.09	0.33	-0.53	0.86	3.92E+01
N-acetylserine	1.03	0.21	0.44	0.47	20.18	0.00	-0.12	0.97	0.00E+00
1-stearoyl-2-oleoyl-GPE (18:0/18:1)	1.12	0.57	0.34	0.11	50.70	0.00	-0.12	0.91	0.00E+00
chiro-inositol	1.87	3.27	2.03	3.74	175.00	1.45	0.92	0.65	3.54E+01
4-allylphenol sulfate	1.47	1.89	-0.11	0.04	128.28	0.00	-0.12	0.77	0.00E+00
1-stearoyl-2-arachidonoyl-GPC (18:0/20:4)	1.01	0.17	-0.31	-0.08	16.99	0.00	-0.12	1.00	1.03E+00
1-palmitoyl-2-linoleoyl-GPE (16:0/18:2)	1.09	0.53	0.05	-0.39	48.24	0.00	-0.12	0.96	0.00E+00
1-stearoyl-2-arachidonoyl-GPS (18:0/20:4)	1.67	1.54	-0.50	-0.51	92.66	0.23	-0.47	0.89	0.00E+00
sphinganine-1-phosphate	1.14	0.40	0.23	0.44	34.98	0.00	-0.12	0.95	0.00E+00
glycosyl-N-nervonoyl-sphingosine (d18:1/24:1)*	1.01	0.32	-0.39	0.23	32.15	0.00	-0.12	0.99	NA
glycosyl-N-stearoyl-sphingosine (d18:1/18:0)	1.04	0.32	-0.20	0.48	31.17	0.00	-0.12	0.98	1.54E+00
cyclo(leu-pro)	1.27	1.02	0.07	-0.98	80.63	0.36	-0.54	0.89	1.01E+01
succinylcarnitine (C4-DC)	1.07	0.48	-0.44	0.59	45.21	0.14	-0.38	0.87	3.11E+00
bilirubin (E,E)*	1.05	0.56	-2.17	7.49	53.59	0.03	-0.19	0.94	0.00E+00
bilirubin (E,Z or Z,E)*	1.03	0.51	-1.62	2.17	49.37	0.11	-0.35	0.98	8.02E+00
N-methylproline	2.47	3.94	0.60	-0.40	159.59	0.00	-0.12	0.75	0.00E+00
beta-cryptoxanthin	1.35	1.25	-0.86	1.69	93.09	0.05	-0.25	0.87	0.00E+00
5alpha-androstan-3beta,17beta-diol disulfate	2.05	4.89	-0.17	0.24	238.47	0.00	-0.12	0.52	0.00E+00
5alpha-pregnan-3beta,20alpha-diol disulfate	4.49	39.59	0.85	5.15	883.46	0.02	-0.18	0.24	0.00E+00
glycocholenate sulfate*	1.16	0.69	0.05	0.84	59.27	0.00	-0.12	0.89	0.00E+00
taurocholenate sulfate*	1.30	0.93	-0.22	0.15	71.42	0.01	-0.13	0.92	3.12E+00
androstenediol (3beta,17beta) disulfate (1)	1.80	3.68	0.17	0.97	204.95	0.00	-0.12	0.58	0.00E+00
pregnenediol disulfate (C21H34O8S2)*	1.35	1.28	-0.27	0.25	94.47	0.00	-0.12	0.85	0.00E+00
androstenediol (3beta,17beta) disulfate (2)	1.18	0.83	-0.36	0.28	70.31	0.00	-0.12	0.91	0.00E+00
21-hydroxypregnenolone disulfate	1.18	0.74	-0.54	0.57	62.99	0.02	-0.16	0.95	0.00E+00
5alpha-androstan-3alpha,17alpha-diol monosulfate	1.28	0.88	0.07	-1.30	68.63	0.52	-0.53	0.92	8.45E+00
5alpha-pregnan-3beta,20beta-diol monosulfate (1)	3.44	21.89	1.30	5.08	636.92	0.11	-0.34	0.30	0.00E+00
5alpha-pregnan-3beta,20alpha-diol monosulfate (2)	4.52	33.64	1.71	7.20	744.63	0.02	-0.16	0.28	0.00E+00
5alpha-pregnan-diol disulfate	4.62	27.05	1.45	2.75	586.31	0.82	-0.31	0.33	0.00E+00
5alpha-androstan-3alpha,17beta-diol disulfate	1.87	2.66	0.54	-1.10	142.25	0.72	-0.41	0.76	1.45E+01
5alpha-androstan-3alpha,17beta-diol monosulfate (1)	1.35	1.32	-0.49	0.09	97.79	0.05	-0.25	0.87	0.00E+00
5alpha-androstan-3beta,17alpha-diol disulfate	1.50	1.57	-0.04	-1.31	105.20	0.43	-0.55	0.87	0.00E+00
5alpha-androstan-3beta,17beta-diol monosulfate (2)	1.41	1.46	-0.31	-0.33	104.39	0.15	-0.40	0.82	0.00E+00
androstenediol (3alpha, 17alpha) monosulfate (2)	1.20	0.82	-0.70	0.69	68.87	0.06	-0.26	0.92	0.00E+00
androstenediol (3alpha, 17alpha) monosulfate (3)	1.28	1.04	-0.34	0.19	81.56	0.00	-0.12	0.89	0.00E+00
androstenediol (3beta,17beta) monosulfate (1)	1.46	1.51	-0.03	0.32	103.64	0.00	-0.12	0.82	0.00E+00
androstenediol (3beta,17beta) monosulfate (2)	1.58	2.48	-0.24	-0.25	156.85	0.26	-0.50	0.63	0.00E+00

Supplementary Table 1: Distribution of raw and transformed metabolites, the correlation between raw and transformed metabolite values, and coefficient of variation of raw metabolites

5alpha-pregnan-3beta-ol,20-one sulfate	4.05	17.14	2.06	4.28	423.81	1.69	1.63	0.42	0.00E+00
4-hydroxycoumarin	1.61	1.91	-0.19	-0.77	118.37	0.27	-0.50	0.81	0.00E+00
1-docosahexaenoyl-GPE (22:6)*	1.03	0.34	0.08	0.07	32.66	0.00	-0.12	0.97	0.00E+00
2-docosahexaenoyl-GPE (22:6)*	1.12	0.59	-0.29	-1.11	52.94	0.38	-0.54	0.95	
1-docosapentaenoyl-GPC (22:5n3)*	1.04	0.39	-0.21	0.82	37.77	0.00	-0.12	0.96	0.00E+00
1-docosapentaenoyl-GPE (22:5n6)*	1.09	0.61	-1.26	3.55	56.60	0.02	-0.16	0.95	4.83E+00
pregnenediol sulfate (C21H34O5S)*	1.24	0.94	-0.35	0.97	76.05	0.00	-0.12	0.87	0.00E+00
2-hydroxyglutarate	1.13	0.48	-0.25	2.35	41.96	0.02	-0.18	0.91	2.24E+01
gamma-CEHC	1.17	0.84	-0.78	2.35	72.30	0.01	-0.13	0.89	0.00E+00
N-acetyl-beta-alanine	1.03	0.29	-0.10	0.39	27.89	0.00	-0.12	0.98	1.15E+00
5-methylthioribose**	1.07	0.39	-0.53	-0.52	36.22	0.25	-0.49	0.97	NA
sphingomyelin (d18:1/18:1, d18:2/18:0)	1.03	0.26	-0.11	0.10	25.26	0.00	-0.12	0.99	0.00E+00
palmitoyl sphingomyelin (d18:1/16:0)	1.00	0.13	-0.27	0.68	13.21	0.00	-0.12	1.00	1.76E+00
cysteine sulfinic acid	1.43	1.55	1.36	2.50	108.32	0.57	-0.51	0.68	2.21E+01
3-hydroxyhippurate	2.16	3.53	0.05	0.48	163.39	0.00	-0.12	0.74	0.00E+00
16a-hydroxy DHEA 3-sulfate	1.43	1.44	-0.23	0.18	101.07	0.00	-0.12	0.84	0.00E+00
17alpha-hydroxypregnenolone 3-sulfate	1.39	1.13	0.69	-0.50	81.02	0.62	-0.49	0.83	9.02E+00
pregnenolone sulfate	1.32	1.06	-0.41	0.86	80.60	0.01	-0.15	0.88	0.00E+00
5-HEPE	5.08	14.79	1.24	1.37	291.43	0.62	-0.49	0.55	4.36E+01
5-HETE	12.24	40.25	1.18	1.93	329.25	0.42	-0.55	0.56	1.89E+01
andro steroid monosulfate C19H28O6S (1)*	1.39	1.59	-0.11	-0.18	114.54	0.15	-0.40	0.74	0.00E+00
ergothioneine	1.31	1.14	-0.06	0.45	87.04	0.01	-0.13	0.85	0.00E+00
1-margaroyl-GPE (17:0)*	1.07	0.52	-1.67	2.96	48.34	0.12	-0.36	0.94	1.53E+00
1-pentadecanoyl-GPC (15:0)*	0.98	0.38	-0.38	0.00	39.03	0.00	-0.12	0.99	1.05E+00
S-methylmethionine	1.66	2.46	0.64	-0.80	148.22	0.77	-0.37	0.70	1.24E+01
indole-3-carboxylate	1.09	0.47	-0.67	0.80	43.36	0.16	-0.41	0.90	1.80E+01
13-HODE + 9-HODE	2.03	3.47	1.25	2.64	171.13	0.01	-0.13	0.65	0.00E+00
tridecenedioate (C13:1-DC)*	1.13	0.67	-0.76	1.27	59.07	0.00	-0.12	0.95	0.00E+00
N-acetyl-3-methylhistidine*	1.45	1.26	-0.03	-0.97	86.96	0.33	-0.53	0.88	0.00E+00
4-cholesten-3-one	1.17	0.70	0.97	3.69	59.95	0.01	-0.13	0.76	1.36E+01
cinnamoylglycine	1.44	1.70	-0.81	-0.02	117.77	0.05	-0.24	0.85	0.00E+00
stearoyl ethanolamide	1.07	0.30	-0.59	0.73	28.19	0.00	-0.12	0.99	3.83E+00
cis-4-decenoylcarnitine (C10:1)	1.10	0.56	0.15	0.14	51.35	0.00	-0.12	0.92	0.00E+00
2S,3R-dihydroxybutyrate	1.16	0.59	0.20	-0.32	50.70	0.00	-0.12	0.94	0.00E+00
phenylalanylphenylalanine	1.00	0.20	-0.54	0.39	19.69	0.00	-0.12	1.00	2.33E+00
(12 or 13)-methylmyristate (a15:0 or i15:0)	1.28	0.83	-0.75	1.09	65.14	0.02	-0.18	0.94	2.82E+01
(16 or 17)-methylstearate (a19:0 or i19:0)	1.20	0.63	-0.13	0.09	53.10	0.00	-0.12	0.94	0.00E+00
2R,3R-dihydroxybutyrate	1.07	0.42	0.02	0.45	39.03	0.00	-0.12	0.95	0.00E+00
4-methylbenzenesulfonate	23.84	368.76	7.63	103.97	1549.33	0.36	-0.54	0.18	0.00E+00
alpha-ketoglutaramate*	1.03	0.24	0.22	0.57	23.61	0.00	-0.12	0.96	0.00E+00
ibuprofen acyl glucuronide	8.58	23.89	3.43	11.17	278.81	2.94	7.35	0.58	0.00E+00
3-hydroxyquinine	1.00	0.51	18.41	338.02	51.29	18.41	338.02	1.00	4.73E+01
2,3-dihydroxyisovalerate	4.16	11.01	0.97	0.59	264.73	0.00	-0.12	0.59	0.00E+00
allopurinol riboside	0.89	0.15	18.41	338.02	16.74	18.41	338.02	1.00	1.09E+01
3-methylglutaconate	1.10	0.53	0.31	0.18	47.97	0.00	-0.12	0.91	0.00E+00
cysteinylglycine disulfide*	1.14	0.70	-0.43	0.55	61.57	0.00	-0.12	0.94	7.82E+00
isoursodeoxycholate	2.05	3.61	0.33	0.04	176.31	0.00	-0.12	0.65	0.00E+00
oxypurinol	0.98	0.03	18.41	338.02	3.55	18.41	338.02	1.00	7.61E+01
formiminoglutamate	1.14	0.70	0.12	-1.67	61.39	0.73	-0.40	0.91	3.72E+01
hydantoin-5-propionate	1.07	0.53	-0.77	1.02	49.59	0.05	-0.25	0.96	0.00E+00
sulfate*	1.11	0.46	0.44	0.51	41.24	0.00	-0.12	0.92	6.05E+00
4-hydroxy-2-oxoglutaric acid	1.02	0.49	-2.42	5.60	47.86	0.07	-0.28	0.98	5.77E+00
4-hydroxyglutamate	1.21	0.86	-0.20	-1.16	70.50	0.44	-0.55	0.87	6.71E+00
L-urobilin	2.44	4.44	0.42	-0.89	182.20	0.56	-0.52	0.69	1.66E+01
pantoate	1.60	2.85	-0.43	2.36	178.68	0.12	-0.36	0.57	2.84E+01
hydroxycotinine	1.12	1.02	2.19	3.28	91.65	2.15	3.37	0.87	0.00E+00
cotinine N-oxide	1.14	0.67	2.56	5.47	59.46	2.26	3.83	0.95	
atenolol	1.52	1.41	10.70	127.74	93.03	8.21	66.41	0.86	1.15E-05
diphenhydramine	4.09	6.13	3.54	11.61	150.13	3.10	8.35	0.82	0.00E+00
hydrochlorothiazide	1.27	1.24	2.75	6.22	97.60	2.48	4.86	0.92	2.61E+01
S-methylcysteine	1.18	0.69	0.51	0.02	58.16	0.00	-0.12	0.90	0.00E+00
androsterone glucuronide	1.25	0.99	-0.63	0.83	79.56	0.01	-0.15	0.91	0.00E+00
argininate*	1.21	0.78	-0.04	0.56	64.47	0.02	-0.18	0.89	0.00E+00
solanidine	2.10	3.85	1.44	1.32	183.35	1.17	0.25	0.62	0.00E+00
2-oxoarginine*	1.27	0.96	0.22	0.44	76.27	0.08	-0.30	0.83	2.73E+01
cis-4-decenoate (10:1n6)*	1.34	1.29	0.49	1.86	96.21	0.00	-0.12	0.72	0.00E+00
atorvastatin (lipitor)	1.42	1.72	5.14	30.11	121.45	3.68	12.26	0.73	0.00E+00
sertraline	1.16	0.66	6.34	40.22	57.07	6.08	35.79	0.94	7.03E+01
gabapentin	1.57	2.31	4.33	17.33	147.92	4.34	17.62	0.69	0.00E+00
venlafaxine	0.85	0.21	18.41	338.02	24.97	18.41	338.02	1.00	6.06E+00
quetiapine	0.92	0.55	18.41	338.02	59.25	18.41	338.02	1.00	5.11E+01
ethyl glucuronide	2.98	9.17	0.50	1.35	308.33	0.21	-0.46	0.51	5.87E+01
1-behenoyl-GPC (22:0)	1.05	0.51	-0.77	0.62	49.01	0.12	-0.36	0.95	2.50E+01
1-erucoyl-GPC (22:1)*	1.08	0.54	0.18	-0.84	50.65	0.44	-0.55	0.90	NA
1-adrenoyl-GPC (22:4)*	1.07	0.44	-0.39	1.69	40.71	0.00	-0.12	0.96	0.00E+00
1-lignoceroyl-GPC (24:0)	1.03	0.45	-0.23	-1.01	44.02	0.32	-0.53	0.97	1.00E+01
1-nervonoyl-GPC (24:1n9)*	1.10	0.46	0.20	-1.30	41.61	0.56	-0.52	0.97	NA
1-(1-enyl-palmitoyl)-GPC (P-16:0)*	1.06	0.43	0.49	0.97	40.87	0.00	-0.12	0.91	0.00E+00
1-(1-enyl-oleoyl)-GPC (P-18:1)*	1.11	0.77	0.20	1.78	69.54	0.11	-0.34	0.77	5.68E+00
1-(1-enyl-stearoyl)-GPC (P-18:0)*	1.16	0.79	0.07	-0.78	68.52	0.43	-0.55	0.83	4.87E+00
1-methyl-5-imidazoleacetate	1.34	1.32	-0.94	1.76	98.46	0.02	-0.16	0.84	0.00E+00
glycoursodeoxycholate	1.93	2.91	0.09	-0.11	150.98	0.00	-0.12	0.73	0.00E+00
tauroursodeoxycholate	1.75	2.58	1.82	2.99	147.59	1.25	0.43	0.70	4.08E-05
S-methylcysteine sulfoxide	1.33	1.24	0.53	0.17	93.51	0.00	-0.12	0.82	0.00E+00
benzoylecgonine	1.29	1.16	5.96	34.74	89.90	5.48	28.79	0.94	2.40E+01
(14 or 15)-methylpalmitate (a17:0 or i17:0)	1.22	0.74	-0.47	0.28	60.82	0.00	-0.12	0.95	0.00E+00
eicosanedioate (C20-DC)	1.11	0.53	0.04	0.15	48.42	0.00	-0.12	0.93	0.00E+00
docosadioate (C22-DC)	1.21	0.67	0.26	-0.08	55.32	0.00	-0.12	0.91	5.42E+00
16-hydroxypalmitate	1.12	0.48	0.91	2.32	43.51	0.00	-0.12	0.85	0.00E+00
quinine	1.00	0.68	18.41	338.02	68.48	18.41	338.02	1.00	6.45E+01
oleoyl-linoleoyl-glycerol (18:1/18:2) [1]	1.12	0.57	0.20	-0.22	50.57	0.00	-0.12	0.93	9.27E+00
oleoyl-linoleoyl-glycerol (18:1/18:2) [2]	1.11	0.52	0.17	-0.43	46.96	0.00	-0.12	0.95	4.11E+00
1-(1-enyl-palmitoyl)-GPE (P-16:0)*	1.14	0.57	0.79	1.48	49.88	0.00	-0.12	0.86	7.05E+00
1-(1-enyl-stearoyl)-GPE (P-18:0)*	1.11	0.54	0.64	1.46	48.54	0.00	-0.12	0.87	1.96E+00
2-stearoyl-GPI (18:0)*	1.13	0.54	-0.35	1.27	47.92	0.04	-0.23	0.91	2.78E+00
alpha-CEHC glucuronide*	2.08	3.23	1.53	1.82	156.02	0.84	-0.28	0.72	1.80E+01
2-oxindole-3-acetate	1.39	1.56	0.50	-0.38	112.53	0.51	-0.53	0.70	1.71E+01
N-oleoyltaurine	1.17	0.65	-0.45	-0.19	55.28	0.15	-0.39	0.94	1.21E+01
linoleoylcarnitine (C18:2)*	1.03	0.31	-0.11	0.01	29.76	0.00	-0.12	0.98	0.00E+00
isoleucylglycine	1.12	0.63	0.73	2.99	56.54	0.02	-0.18	0.77	3.32E+00
leucylglycine	1.18	0.50	-0.88	2.40	42.51	0.07	-0.28	0.92	1.43E+01
phenylalanylisoleucine	1.09	0.43	1.27	0.97	39.66	1.02	-0.03	0.89	NA
phenylalanylleucine	1.01	0.25	-0.65	0.79	25.17	0.02	-0.18	1.00	NA
N-palmitoyltaurine	1.19	0.79	0.59	-0.67	66.08	0.67	-0.45	0.86	NA
N-stearoyltaurine	1.09	0.51	-0.47	0.12	47.01	0.09	-0.32	0.96	1.17E+01
2-O-methylascorbic acid	1.04	0.34	-0.64	2.46	32.47	0.00	-0.12	0.98	0.00E+00
beta-citrylglutamate	1.08	0.37	0.03	0.24	34.14	0.00	-0.12	0.96	3.23E+00

Supplementary Table 1: Distribution of raw and transformed metabolites, the correlation between raw and transformed metabolite values, and coefficient of variation of raw metabolites

rosuvastatin	1.09	0.54	7.65	62.18	49.31	6.92	46.75	0.88	3.92E+01
trimethylamine N-oxide	1.24	0.97	0.21	2.15	78.19	0.00	-0.12	0.80	0.00E+00
N6-methyllysine	1.80	1.72	0.39	-0.91	95.97	0.00	-0.12	0.88	0.00E+00
dihydroferulate	1.64	3.20	1.10	0.87	195.95	0.87	-0.24	0.54	8.98E+00
imidazole propionate	1.16	0.62	-0.12	1.06	53.58	0.06	-0.27	0.88	8.61E+00
citalopram/escitalopram	1.25	1.10	4.84	22.11	87.81	4.82	22.02	0.92	9.02E+01
duloxetine	0.96	0.23	18.41	338.02	24.35	18.41	338.02	1.00	NA
pregnanediol-3-glucuronide	2.56	10.36	0.91	3.09	404.92	0.05	-0.25	0.40	0.00E+00
(15:2)-anacardic acid	1.15	0.72	8.55	82.06	62.57	5.76	31.95	0.87	0.00E+00
alliin	1.80	3.06	0.88	-0.24	169.64	0.76	-0.38	0.69	2.31E+01
seryltyrosine	2.06	5.43	3.06	9.57	264.05	2.51	5.03	0.51	3.53E+01
histidylalanine	4.00	13.99	2.46	5.14	350.46	2.18	3.48	0.46	2.78E+01
phenylalanylglycine	1.03	0.27	0.40	-1.16	26.41	0.62	-0.49	0.98	NA
phenylalanylmethionine	1.03	0.29	-1.31	3.00	28.53	0.03	-0.19	0.99	6.12E+00
phenylalanyltryptophan	0.99	0.40	-2.15	4.72	40.04	0.00	-0.12	0.98	1.14E-03
tyrosyllysine	1.67	2.11	2.79	6.94	126.23	2.41	4.53	0.79	NA
tryptophylasparagine	1.13	0.70	2.82	6.50	62.39	2.75	6.28	0.97	2.02E+01
aspartylaspartate	1.05	0.37	-0.21	-0.91	34.83	0.31	-0.52	0.97	3.98E+00
valylalanine	3.42	7.65	1.57	2.66	224.03	0.55	-0.52	0.62	7.67E+01
valylglycine	1.35	1.03	0.09	0.24	76.44	0.10	-0.33	0.84	1.68E+01
valylphenylalanine	3.29	6.86	1.87	3.43	208.63	0.48	-0.54	0.64	NA
methionylalanine	15.74	28.06	1.40	0.78	178.55	0.98	-0.09	0.79	1.03E+02
N-palmitoylglycine	1.08	0.40	-0.55	1.38	37.32	0.01	-0.15	0.97	2.00E+00
mannonate*	1.08	0.36	0.55	0.79	33.49	0.00	-0.12	0.93	0.00E+00
norfluoxetine	1.14	0.91	6.30	40.61	79.96	5.76	31.95	0.87	1.52E+01
2-stearoyl-GPE (18:0)*	1.05	0.43	-0.02	0.53	40.78	0.00	-0.12	0.95	0.00E+00
(R)-3-hydroxybutyrylcarnitine	1.41	1.22	0.41	-0.90	86.47	0.54	-0.53	0.85	1.23E+01
N-octanoylglycine	1.24	0.74	-0.13	-0.56	59.52	0.29	-0.52	0.85	2.18E+01
doxylamine	0.95	0.55	9.15	82.39	57.53	9.20	83.56	0.95	0.00E+00
triamterene	1.00	0.62	18.41	338.02	61.60	18.41	338.02	1.00	2.52E+01
diltiazem	0.92	0.31	9.88	99.96	33.58	9.20	83.58	0.97	1.03E+01
fexofenadine	12.24	28.41	8.90	87.11	232.56	6.08	35.80	0.68	4.22E+01
verapamil	1.20	0.48	11.18	128.16	39.69	10.63	112.06	0.90	5.19E+01
N-acetylcarnosine	1.11	0.53	-0.07	-0.21	48.28	0.00	-0.12	0.96	4.79E-07
margaroylcarnitine (C17)*	1.06	0.38	-0.45	0.36	35.93	0.00	-0.12	0.99	2.56E+00
N-methyltaurine	1.57	1.82	0.53	-0.86	116.23	0.58	-0.51	0.83	8.91E-06
histidine betaine (hercynine)*	1.47	1.67	0.58	0.74	114.20	0.01	-0.13	0.75	1.05E+01
glycohyocholate	1.51	1.48	-0.02	-0.74	97.66	0.20	-0.45	0.86	1.05E+01
2-hydroxydecanoate	1.19	0.74	0.39	1.33	62.47	0.00	-0.12	0.85	0.00E+00
3-methyl catechol sulfate (2)	1.79	2.07	1.04	-0.01	115.92	0.91	-0.19	0.81	1.07E+01
4-methylcatechol sulfate	1.36	1.29	-0.15	0.90	94.79	0.00	-0.12	0.83	0.00E+00
3-methyl catechol sulfate (1)	1.76	2.05	-0.08	-0.61	116.22	0.01	-0.13	0.86	0.00E+00
2-hydroxyibuprofen	9.89	19.48	2.78	6.77	197.29	2.01	2.78	0.74	0.00E+00
carboxyibuprofen	53.02	182.09	2.51	6.10	343.96	1.36	0.68	0.56	4.77E+01
O-desmethylvenlafaxine	1.18	0.77	12.65	172.26	65.89	10.63	112.06	0.93	9.53E+00
zolpidem	0.96	0.07	12.96	166.52	7.61	12.96	166.52	1.00	9.14E+01
warfarin	0.97	0.89	8.70	75.30	92.00	8.22	66.44	0.94	0.00E+00
3b-hydroxy-5-cholenoic acid	1.14	0.65	-0.27	0.12	57.27	0.11	-0.34	0.91	1.16E+01
guaiacol sulfate	1.22	0.93	-0.33	1.50	76.29	0.00	-0.12	0.87	0.00E+00
2-aminooctanoate	1.11	0.64	-0.41	0.69	58.06	0.00	-0.12	0.94	0.00E+00
furosemide	1.19	0.32	9.51	91.74	26.92	9.20	83.56	0.91	0.00E+00
gamma-CEHC glucuronide*	1.24	1.05	-0.19	0.21	84.75	0.07	-0.28	0.83	0.00E+00
dimethyl sulfone	2.46	8.52	0.78	2.67	346.20	0.03	-0.19	0.44	0.00E+00
N-acetyl-1-methylhistidine*	1.25	0.90	0.04	-1.34	72.02	0.53	-0.53	0.90	1.21E+01
indolin-2-one	1.11	0.51	0.42	-1.30	46.23	0.76	-0.38	0.95	2.20E+01
2,8-quinolinediol sulfate	1.59	1.64	2.31	4.70	103.00	1.69	1.63	0.82	1.78E+01
2-aminophenol sulfate	1.82	2.16	-0.15	-0.30	119.15	0.00	-0.12	0.86	0.00E+00
3-acetylphenol sulfate	1.56	1.75	0.43	-0.69	112.19	0.42	-0.55	0.80	1.07E+01
sphingomyelin (d18:1/14:0, d16:1/16:0)*	1.00	0.26	-0.31	-0.07	25.47	0.00	-0.12	0.99	0.00E+00
sphingomyelin (d18:2/16:0, d18:1/16:1)*	1.01	0.19	-0.17	-0.10	18.54	0.00	-0.12	1.00	0.00E+00
3-hydroxyadipate	1.22	0.84	-0.51	0.12	69.12	0.19	-0.44	0.87	0.00E+00
3-hydroxycotinine glucuronide	1.26	1.15	2.33	4.16	91.79	2.20	3.59	0.84	0.00E+00
6-oxopiperidine-2-carboxylate	1.11	0.50	0.42	1.06	45.63	0.00	-0.12	0.89	2.71E+01
S-allylcysteine	1.81	2.75	-0.17	-0.92	152.02	0.28	-0.51	0.74	9.33E-08
allopurinol	1.08	0.11	14.49	218.61	10.07	13.03	168.78	1.00	6.18E+00
N-delta-acetylornithine	1.15	0.86	0.17	0.45	74.72	0.00	-0.12	0.85	0.00E+00
acisoga	1.08	0.47	-0.36	0.06	43.57	0.01	-0.13	0.98	3.91E+00
2-aminoheptanoate	1.09	0.43	-0.70	3.85	39.93	0.01	-0.13	0.95	0.00E+00
1-eicosapentaenoyl-GPE (20:5)*	1.19	0.93	0.06	0.38	78.42	0.00	-0.12	0.86	0.00E+00
N-formylanthranilic acid	1.36	1.88	1.49	6.21	138.64	0.01	-0.13	0.55	0.00E+00
N2,N5-diacetylornithine	1.14	0.79	0.54	0.82	69.19	0.00	-0.12	0.82	0.00E+00
1H-indole-7-acetic acid	1.14	0.76	-0.73	-0.08	67.12	0.19	-0.44	0.89	1.35E+01
3-methoxytyramine sulfate	1.08	0.41	-0.38	-0.13	38.28	0.16	-0.41	0.96	7.48E-01
methionine sulfone	1.15	0.90	0.70	2.09	78.66	0.00	-0.12	0.76	0.00E+00
cyclo(ala-pro)	1.19	0.66	-0.11	-1.22	55.12	0.40	-0.55	0.95	5.11E+00
norbenzoylecgonine*	1.97	2.74	9.79	113.27	139.39	6.46	40.60	0.70	7.70E+01
1-linolenoyl-GPC (18:3)*	1.11	0.57	-0.01	1.38	51.04	0.00	-0.12	0.89	0.00E+00
1-eicosapentaenoyl-GPC (20:5)*	1.21	0.95	0.27	0.50	78.78	0.00	-0.12	0.85	0.00E+00
1-eicosenoyl-GPC (20:1)*	1.00	0.36	-0.14	0.06	36.40	0.00	-0.12	0.98	6.73E-01
1-nonadecanoyl-GPC (19:0)	1.01	0.52	-0.38	-1.27	51.83	0.39	-0.55	0.97	1.51E+01
N-acetylalliin	1.80	2.23	-0.11	-1.03	124.43	0.25	-0.49	0.83	2.11E-07
1-dihomo-linolenoyl-GPE (20:3n3 or 6)*	1.06	0.44	-0.06	-0.38	41.89	0.00	-0.12	0.97	3.01E+00
1-(1-enyl-oleoyl)-GPE (P-18:1)*	1.18	0.70	0.32	0.86	59.67	0.00	-0.12	0.87	7.06E+00
fructosyllysine	1.05	0.29	-0.59	1.03	27.12	0.01	-0.13	0.99	6.58E+00
1-eicosenoyl-GPE (20:1)*	1.09	0.59	-0.69	0.35	53.72	0.11	-0.35	0.93	5.26E+00
N-methylpipecolate	1.54	1.47	0.18	-0.64	95.67	0.01	-0.13	0.89	NA
O-sulfo-L-tyrosine	1.04	0.29	0.14	0.68	27.95	0.00	-0.12	0.96	0.00E+00
ferulic acid 4-sulfate	1.71	1.98	1.16	0.12	115.96	1.07	0.07	0.83	1.31E+01
3-(3-hydroxyphenyl)propionate sulfate	1.98	3.51	0.17	-0.58	177.36	0.29	-0.51	0.65	2.22E+01
11-ketoetiocholanolone glucuronide	1.12	0.67	-0.51	-0.56	59.85	0.22	-0.47	0.94	8.95E+00
etiocholanolone glucuronide	1.19	1.12	-0.42	1.26	94.24	0.01	-0.15	0.76	0.00E+00
17alpha-hydroxypregnanolone glucuronide	1.30	1.10	-0.44	-0.35	85.02	0.15	-0.39	0.89	1.56E+01
N-acetyltaurine	1.09	0.57	0.70	2.50	52.33	0.00	-0.12	0.81	4.53E-08
1-docosapentaenoyl-GPC (22:5n6)*	1.12	0.55	-0.39	0.53	49.13	0.02	-0.18	0.95	1.38E+01
1-linolenoyl-GPE (18:3)*	1.15	0.87	-0.18	0.95	75.88	0.02	-0.18	0.80	7.19E-01
1-oleoyl-GPG (18:1)*	1.11	0.60	0.28	-1.56	54.05	0.80	-0.33	0.90	2.27E+01
1-palmitoyl-GPG (16:0)*	1.07	0.39	-0.55	0.44	36.89	0.11	-0.34	0.96	5.84E+00
N-linoleoyltaurine*	1.27	1.02	-0.08	-0.94	79.84	0.41	-0.55	0.79	1.40E+01
N-acetyl-S-allyl-L-cysteine	1.55	1.64	0.83	-0.59	105.68	0.80	-0.33	0.84	1.99E+01
9-hydroxystearate	1.34	1.04	-0.36	0.17	77.56	0.12	-0.36	0.86	0.00E+00
3-methylglutarylcarnitine (2)	1.24	1.02	-0.79	-0.04	82.13	0.24	-0.49	0.84	5.53E+00
methyl glucopyranoside (alpha + beta)	1.40	1.46	-0.18	0.50	104.62	0.08	-0.29	0.79	0.00E+00
2-keto-3-deoxy-gluconate	1.08	0.44	-1.04	4.62	40.99	0.02	-0.16	0.92	0.00E+00
alpha-CEHC sulfate	2.17	4.15	0.97	0.08	191.33	0.79	-0.34	0.66	1.69E+01
alpha-CMBHC glucuronide	1.37	1.40	0.11	-0.91	102.54	0.41	-0.55	0.79	8.36E+00
sphingomyelin (d18:2/14:0, d18:1/14:1)*	1.07	0.45	-0.05	-0.31	41.90	0.00	-0.12	0.97	0.00E+00
sphingomyelin (d18:1/24:1, d18:2/24:0)*	1.04	0.23	0.17	-0.41	22.50	0.00	-0.12	0.98	2.30E+00

Supplementary Table 1: Distribution of raw and transformed metabolites, the correlation between raw and transformed metabolite values, and coefficient of variation of raw metabolites

octadecenedioylcarnitine (C18:1-DC)*	1.14	0.64	0.03	0.04	56.52	0.00	-0.12	0.93	0.00E+00
octadecanedioylcarnitine (C18-DC)*	1.08	0.52	-0.24	0.22	48.30	0.00	-0.12	0.95	0.00E+00
N,O-didesmethylvenlafaxine glucuronide	0.62	0.45	11.23	127.67	72.65	10.58	110.58	0.99	7.83E+01
5alpha-androstan-3alpha,17beta-diol monosulfate (2)	2.95	4.81	-0.13	-0.38	163.06	0.16	-0.41	0.77	1.96E+01
myristoleoylcarnitine (C14:1)*	1.19	0.84	0.15	0.36	70.44	0.00	-0.12	0.85	0.00E+00
N-formylphenylalanine	1.12	0.78	1.16	3.66	69.58	0.01	-0.15	0.74	8.20E+00
cyclo(pro-val)	1.23	0.82	-0.57	1.36	66.98	0.02	-0.18	0.92	0.00E+00
4-hydroxychlorothalonil	1.08	0.49	-0.72	1.52	45.60	0.00	-0.12	0.98	0.00E+00
isoeugenol sulfate	2.06	3.33	0.96	-0.06	161.65	0.78	-0.35	0.70	2.85E+01
tyramine O-sulfate	1.94	3.33	0.40	0.29	172.04	0.14	-0.39	0.66	0.00E+00
3-hydroxypyridine sulfate	2.21	4.62	0.12	0.39	209.11	0.00	-0.12	0.65	0.00E+00
4-methylguaiacol sulfate	1.77	2.60	0.02	-0.23	146.87	0.13	-0.38	0.73	0.00E+00
maltol sulfate	1.75	2.46	2.58	6.67	141.01	1.74	1.80	0.72	NA
phenylacetylcarnitine	1.35	1.07	-0.31	-0.96	78.94	0.34	-0.53	0.89	8.41E+00
arabonate/xylonate	1.07	0.44	0.34	0.48	41.48	0.00	-0.12	0.92	0.00E+00
methyl-4-hydroxybenzoate sulfate	3.71	7.16	-0.13	-0.87	193.04	0.00	-0.12	0.73	0.00E+00
1-dihomo-linolenylglycerol (20:3)	1.20	0.72	-0.59	0.14	60.37	0.14	-0.39	0.93	3.18E+01
vanillic alcohol sulfate	2.09	2.61	1.66	1.88	125.31	1.07	0.07	0.81	1.77E+01
4-vinylguaiacol sulfate	2.62	4.12	0.86	-0.23	157.79	0.50	-0.54	0.75	4.43E+01
vanillactate	1.19	0.97	0.90	4.74	81.50	0.02	-0.16	0.66	3.70E+00
eugenol sulfate	2.87	9.70	0.12	-0.14	338.98	0.22	-0.47	0.46	7.02E-06
pregnanolone/allopregnanolone sulfate	3.79	19.35	1.60	2.79	511.17	1.20	0.33	0.35	1.39E-05
2-methoxyresorcinol sulfate	3.60	9.67	1.33	0.88	269.07	0.96	-0.12	0.57	0.00E+00
2-acetamidophenol sulfate	1.91	2.38	-0.08	-0.99	124.70	0.30	-0.52	0.83	9.14E+00
p-cresol glucuronide*	1.51	1.59	-0.69	0.47	105.45	0.02	-0.18	0.88	0.00E+00
acesulfame	5.75	14.45	0.93	-0.60	251.70	0.95	-0.14	0.62	2.72E+01
valsartan	1.04	0.83	12.60	163.06	79.76	10.63	112.06	1.00	4.67E+01
6-hydroxyindole sulfate	1.13	0.64	-0.53	0.70	57.03	0.00	-0.12	0.95	0.00E+00
4-methoxyphenol sulfate	1.55	1.87	-0.31	0.40	120.67	0.12	-0.36	0.77	6.67E+00
2,4-dichlorophenol sulfate	3.53	7.59	6.42	47.87	215.17	4.49	18.90	0.66	0.00E+00
propyl 4-hydroxybenzoate sulfate	3.13	6.40	0.44	-1.15	204.77	0.65	-0.47	0.70	0.00E+00
ethylparaben sulfate	10.81	39.93	0.47	-0.44	370.00	0.33	-0.53	0.51	3.80E+01
umbelliferone sulfate	2.84	4.82	0.23	-0.84	170.10	0.30	-0.52	0.73	0.00E+00
sphingomyelin (d18:1/20:0, d16:1/22:0)*	1.01	0.22	-0.48	0.99	21.51	0.00	-0.12	1.00	0.00E+00
sphingomyelin (d18:1/20:1, d18:2/20:0)*	1.01	0.25	-0.61	0.85	25.01	0.00	-0.12	1.00	0.00E+00
sphingomyelin (d18:1/20:2, d18:2/20:1, d16:1/22:2)*	1.06	0.40	-0.29	0.05	37.40	0.00	-0.12	0.98	0.00E+00
behenoyl sphingomyelin (d18:1/22:0)*	1.04	0.31	-0.02	-0.66	30.26	0.00	-0.12	0.98	2.54E+00
sphingomyelin (d18:1/22:1, d18:2/22:0, d16:1/24:1)*	1.01	0.20	-0.35	0.32	19.72	0.00	-0.12	1.00	0.00E+00
sphingomyelin (d18:1/22:2, d18:2/22:1, d16:1/24:2)*	1.04	0.31	-0.08	-0.25	29.44	0.00	-0.12	0.98	0.00E+00
lignoceroyl sphingomyelin (d18:1/24:0)	1.01	0.23	-0.37	0.61	22.76	0.00	-0.12	0.99	0.00E+00
sphingomyelin (d17:1/16:0, d18:1/15:0, d16:1/17:0)*	1.03	0.30	-0.31	0.17	29.12	0.00	-0.12	0.99	0.00E+00
dopamine 3-O-sulfate	1.26	1.14	1.12	2.45	90.56	0.00	-0.12	0.70	7.11E-08
3-hydroxyhexanoate	1.11	0.54	0.11	0.22	49.09	0.00	-0.12	0.93	0.00E+00
N-carbamoylalanine	1.16	0.74	-0.52	-0.41	63.74	0.28	-0.51	0.88	4.98E+00
3beta-hydroxy-5-cholestenoate	1.09	0.40	-0.07	0.30	36.88	0.00	-0.12	0.97	3.89E+00
1,2,3-benzenetriol sulfate (2)	2.09	3.44	1.95	3.08	165.00	1.49	1.03	0.72	1.09E+02
3-methoxycatechol sulfate (1)	3.12	5.07	0.04	-0.53	162.80	0.02	-0.16	0.78	0.00E+00
3-methoxycatechol sulfate (2)	1.26	0.98	-0.39	-0.39	77.94	0.19	-0.44	0.88	1.43E+01
N-acetylkynurenine (2)	1.44	1.51	-0.07	0.04	104.88	0.11	-0.34	0.77	1.67E+01
C-glycosyltryptophan	1.02	0.22	0.14	1.18	21.61	0.00	-0.12	0.97	0.00E+00
tramadol	1.00	1.39	18.41	338.02	139.04	18.41	338.02	1.00	4.16E+01
O-desmethyltramadol	1.00	1.38	18.41	338.02	137.97	18.41	338.02	1.00	7.36E+01
O-desmethyltramadol glucuronide	1.00	1.34	18.41	338.02	133.72	18.41	338.02	1.00	3.61E+01
arabitol/xylitol	1.04	0.30	0.48	1.15	29.24	0.00	-0.12	0.93	0.00E+00
N-acetylglucosamine/N-acetylgalactosamine	1.06	0.29	-0.65	1.35	27.07	0.05	-0.24	0.98	0.00E+00
citraconate/glutaconate	2.24	4.93	2.02	4.54	220.50	0.00	-0.12	0.56	0.00E+00
adipoylcarnitine (C6-DC)	1.18	0.63	-0.03	-0.83	53.23	0.30	-0.52	0.93	8.83E+00
nonanoylcarnitine (C9)	1.30	1.56	0.41	2.37	119.98	0.06	-0.27	0.64	0.00E+00
glycochenodeoxycholate 3-sulfate	1.51	1.99	-0.88	0.28	132.11	0.20	-0.45	0.73	0.00E+00
glycodeoxycholate 3-sulfate	1.36	1.29	-0.66	0.28	95.35	0.13	-0.37	0.84	0.00E+00
taurodeoxycholic acid 3-sulfate	1.46	1.65	-0.29	-0.75	113.59	0.29	-0.51	0.80	0.00E+00
trans-3,4-methyleneheptanoate	1.17	0.76	-0.16	-1.43	65.08	0.49	-0.54	0.94	NA
phenol glucuronide	1.77	2.57	0.64	0.01	145.19	0.36	-0.54	0.70	0.00E+00
linoleoyl ethanolamide	1.35	1.11	-0.33	0.13	82.26	0.13	-0.38	0.79	0.00E+00
1,2-dilinoleoyl-GPC (18:2/18:2)	1.02	0.31	-0.61	0.52	30.23	0.00	-0.12	0.99	2.06E+00
1-stearoyl-2-oleoyl-GPC (18:0/18:1)	1.04	0.25	0.27	0.27	23.64	0.00	-0.12	0.97	1.36E+00
1-palmitoyl-2-arachidonoyl-GPC (16:0/20:4n6)	1.00	0.16	-0.24	0.31	15.81	0.00	-0.12	0.99	0.00E+00
1-palmitoyl-2-docosahexaenoyl-GPC (16:0/22:6)	1.02	0.25	0.01	-0.06	24.30	0.00	-0.12	0.98	0.00E+00
1-stearoyl-2-docosahexaenoyl-GPC (18:0/22:6)	1.04	0.34	-0.27	0.88	32.98	0.00	-0.12	0.98	0.00E+00
1-(1-enyl-stearoyl)-2-oleoyl-GPC (P-18:0/18:1)	1.03	0.38	-0.08	-0.34	37.51	0.00	-0.12	0.98	2.50E+00
1-(1-enyl-stearoyl)-2-arachidonoyl-GPC (P-18:0/20:4)	1.07	0.39	-0.18	0.22	36.67	0.00	-0.12	0.98	0.00E+00
1-(1-enyl-stearoyl)-2-oleoyl-GPE (P-18:0/18:1)	1.06	0.39	0.07	-0.01	36.52	0.00	-0.12	0.96	0.00E+00
sphingomyelin (d18:1/17:0, d17:1/18:0, d19:1/16:0)	1.03	0.29	-0.31	0.36	27.85	0.00	-0.12	0.99	0.00E+00
1-palmitoyl-2-stearoyl-GPC (16:0/18:0)	1.03	0.19	-0.08	-0.26	19.02	0.00	-0.12	0.99	0.00E+00
2-hydroxybutyrate/2-hydroxyisobutyrate	1.12	0.48	-0.01	0.38	43.17	0.00	-0.12	0.93	0.00E+00
oleate/vaccenate (18:1)	1.18	0.60	-0.74	0.57	50.72	0.00	-0.12	0.98	1.88E+00
isoleucylleucine/leucylisoleucine	1.09	0.38	-0.73	0.43	34.84	0.15	-0.40	0.95	4.67E+00
aripiprazole	1.00	0.68	13.60	188.66	68.04	13.03	168.78	1.00	7.18E+01
leucylphenylalanine/isoleucylphenylalanine	15.86	46.02	1.83	3.03	290.66	0.24	-0.48	0.61	
1-palmitoleoylglycerol (16:1)*	1.25	1.18	-0.32	-0.81	94.35	0.35	-0.54	0.80	1.70E+01
palmitoyl dihydrosphingomyelin (d18:0/16:0)*	1.02	0.24	-0.12	0.19	23.48	0.00	-0.12	0.99	0.00E+00
tricosanoyl sphingomyelin (d18:1/23:0)*	0.99	0.24	-0.74	1.37	24.04	0.00	-0.12	1.00	1.31E-08
sphingomyelin (d18:2/23:0, d18:1/23:1, d17:1/24:1)*	1.00	0.27	-0.55	0.72	27.31	0.00	-0.12	1.00	3.19E+00
sphingomyelin (d18:2/24:1, d18:1/24:2)*	1.02	0.20	-0.05	-0.25	19.65	0.00	-0.12	0.99	0.00E+00
diclofenac	1.06	0.27	10.67	113.15	25.34	10.63	112.06	0.92	0.00E+00
1-stearoyl-2-linoleoyl-GPE (18:0/18:2)*	1.10	0.49	0.18	-0.34	45.18	0.00	-0.12	0.95	0.00E+00
1-stearoyl-2-arachidonoyl-GPE (18:0/20:4)	1.06	0.37	-0.21	0.31	35.28	0.00	-0.12	0.98	0.00E+00
1-stearoyl-2-linoleoyl-GPC (18:0/18:2)*	1.00	0.15	-0.24	-0.09	14.98	0.00	-0.12	1.00	3.51E-01
1-palmitoyl-2-palmitoleoyl-GPC (16:0/16:1)*	1.18	0.72	0.39	0.51	61.56	0.00	-0.12	0.87	1.21E+00
1-palmitoyl-2-eicosapentaenoyl-GPC (16:0/20:5)*	1.07	0.51	0.02	0.78	47.26	0.00	-0.12	0.92	0.00E+00
1-palmitoyl-2-arachidonoyl-GPE (16:0/20:4)*	1.06	0.43	-0.16	0.18	40.83	0.00	-0.12	0.97	0.00E+00
1-palmitoyl-2-docosahexaenoyl-GPE (16:0/22:6)*	1.11	0.65	-0.38	1.16	58.25	0.00	-0.12	0.93	3.31E+00
1-stearoyl-2-docosahexaenoyl-GPE (18:0/22:6)*	1.11	0.60	-0.58	1.81	54.42	0.02	-0.16	0.91	0.00E+00
1-palmitoyl-2-arachidonoyl-GPI (16:0/20:4)*	1.10	0.54	-0.16	0.21	48.72	0.00	-0.12	0.95	0.00E+00
1-stearoyl-2-linoleoyl-GPI (18:0/18:2)	1.03	0.35	-0.29	-0.17	34.49	0.00	-0.12	0.99	0.00E+00
1-palmitoyl-2-palmitoleoyl-GPE (16:0/16:1)*	1.42	1.33	1.46	1.16	93.76	1.20	0.33	0.82	0.00E+00
gamma-tocopherol/beta-tocopherol	1.23	0.81	-2.93	12.81	66.36	0.02	-0.18	0.95	2.22E-06
1-(1-enyl-stearoyl)-2-arachidonoyl-GPE (P-18:0/20:4)*	1.08	0.41	-0.06	-0.30	37.98	0.00	-0.12	0.98	1.83E+00
1-(1-enyl-palmitoyl)-2-docosahexaenoyl-GPE (P-16:0/22:6)*	1.04	0.37	-0.61	1.27	35.32	0.00	-0.12	0.98	0.00E+00
1-(1-enyl-palmitoyl)-2-arachidonoyl-GPE (P-16:0/20:4)*	1.06	0.41	0.05	0.04	38.39	0.00	-0.12	0.96	0.00E+00
1-(1-enyl-oleoyl)-2-linoleoyl-GPE (P-18:1/18:2)*	1.21	0.71	-0.26	0.25	58.60	0.04	-0.23	0.93	1.11E+01
1-(1-enyl-stearoyl)-2-docosahexaenoyl-GPE (P-18:0/22:6)*	1.04	0.43	-0.34	0.32	41.23	0.00	-0.12	0.98	0.00E+00
1-(1-enyl-palmitoyl)-2-oleoyl-GPE (P-16:0/18:1)*	1.03	0.29	0.02	0.34	27.59	0.00	-0.12	0.97	0.00E+00
1-(1-enyl-palmitoyl)-2-dihomo-linolenoyl-GPC (P-16:0/20:3)*	1.06	0.36	-0.50	0.02	34.06	0.11	-0.35	0.98	7.12E+00
1-(1-enyl-palmitoyl)-2-oleoyl-GPC (P-16:0/18:1)*	1.04	0.31	0.02	-0.54	30.43	0.00	-0.12	0.98	0.00E+00
1-(1-enyl-palmitoyl)-2-docosahexaenoyl-GPC (P-16:0/22:6)*	1.05	0.39	0.11	-0.40	37.38	0.00	-0.12	0.97	7.73E-08
1-(1-enyl-palmitoyl)-2-linoleoyl-GPC (P-16:0/18:2)*	1.03	0.30	-0.26	-0.06	29.07	0.00	-0.12	0.99	0.00E+00

Supplementary Table 1: Distribution of raw and transformed metabolites, the correlation between raw and transformed metabolite values, and coefficient of variation of raw metabolites

1-(1-enyl-palmitoyl)-2-arachidonoyl-GPC (P-16:0/20:4)*	1.04	0.28	0.00	0.10	27.37	0.00	-0.12	0.98	0.00E+00
1-(1-enyl-stearoyl)-2-docosahexaenoyl-GPC (P-18:0/22:6)*	1.10	0.50	0.01	-0.16	45.02	0.00	-0.12	0.95	0.00E+00
1-stearyl-2-arachidonoyl-GPC (O-18:0/20:4)*	1.03	0.29	-0.67	1.14	28.05	0.00	-0.12	0.99	0.00E+00
1-palmityl-2-oleoyl-GPC (O-16:0/18:1)*	1.02	0.27	-0.08	-0.66	26.26	0.00	-0.12	0.99	0.00E+00
1-palmityl-2-arachidonoyl-GPC (O-16:0/20:4)*	1.06	0.33	0.12	0.89	31.28	0.00	-0.12	0.94	0.00E+00
sphingomyelin (d18:1/21:0, d17:1/22:0, d16:1/23:0)*	1.00	0.31	-0.86	1.90	30.68	0.00	-0.12	1.00	4.06E+00
behenoyl dihydrosphingomyelin (d18:0/22:0)*	1.08	0.44	-0.05	-0.09	40.64	0.00	-0.12	0.97	0.00E+00
sphingomyelin (d18:0/18:0, d19:0/17:0)*	1.12	0.55	0.06	-0.08	49.47	0.00	-0.12	0.94	0.00E+00
N-palmitoyl-sphinganine (d18:0/16:0)	1.06	0.38	0.47	1.00	36.14	0.00	-0.12	0.91	5.60E+00
lactosyl-N-palmitoyl-sphingosine (d18:1/16:0)	1.02	0.23	-0.11	-0.14	22.77	0.00	-0.12	0.99	0.00E+00
cetirizine	1.39	1.57	3.01	7.63	113.09	2.94	7.35	0.83	0.00E+00
1-pentadecanoyl-2-linoleoyl-GPC (15:0/18:2)*	1.04	0.36	-0.45	0.18	34.35	0.00	-0.12	0.99	0.00E+00
1-margaroyl-2-oleoyl-GPC (17:0/18:1)*	0.99	0.27	-0.13	-0.05	26.99	0.00	-0.12	0.99	0.00E+00
1-margaroyl-2-linoleoyl-GPC (17:0/18:2)*	1.00	0.24	-0.57	0.65	23.60	0.00	-0.12	1.00	0.00E+00
myristoyl dihydrosphingomyelin (d18:0/14:0)*	1.03	0.37	-0.05	0.22	35.82	0.00	-0.12	0.96	0.00E+00
5-hydroxyindole sulfate	1.46	3.03	0.14	1.50	208.03	0.14	-0.39	0.44	0.00E+00
7-hydroxyindole sulfate	1.47	1.45	1.07	0.61	98.97	0.72	-0.41	0.77	2.70E+01
phenylacetylglutamate	1.28	1.06	-0.19	-1.09	82.75	0.36	-0.54	0.86	1.46E+01
1-stearoyl-2-dihomo-linolenoyl-GPC (18:0/20:3n3 or 6)*	1.02	0.26	-0.31	-0.03	25.65	0.00	-0.12	0.99	0.00E+00
palmitoyl-linoleoyl-glycerol (16:0/18:2) [1]*	1.15	0.66	-0.52	1.95	57.26	0.01	-0.13	0.93	
palmitoyl-linoleoyl-glycerol (16:0/18:2) [2]*	1.12	0.62	0.23	-0.05	55.43	0.00	-0.12	0.92	
1-palmitoyl-2-oleoyl-GPI (16:0/18:1)*	1.10	0.58	0.05	0.38	52.94	0.00	-0.12	0.92	2.29E+00
1-stearoyl-2-docosahexaenoyl-GPI (18:0/22:6)*	1.15	0.65	0.11	-0.04	56.28	0.00	-0.12	0.93	6.01E+00
1-(1-enyl-palmitoyl)-2-linoleoyl-GPE (P-16:0/18:2)*	1.07	0.41	-0.04	0.83	38.23	0.00	-0.12	0.94	2.32E+00
1-oleoyl-2-linoleoyl-GPE (18:1/18:2)*	1.07	0.51	-0.20	-0.24	47.33	0.03	-0.19	0.96	6.81E+00
1-pentadecanoyl-2-arachidonoyl-GPC (15:0/20:4)*	1.04	0.41	-0.57	0.85	39.24	0.02	-0.18	0.97	0.00E+00
1-pentadecanoyl-2-docosahexaenoyl-GPC (15:0/22:6)*	1.09	0.61	-0.13	0.66	56.51	0.01	-0.15	0.92	0.00E+00
1-margaroyl-2-arachidonoyl-GPC (17:0/20:4)*	1.07	0.43	-0.58	1.11	40.48	0.00	-0.12	0.98	0.00E+00
1-arachidoyl-2-arachidonoyl-GPC (20:0/20:4)*	1.05	0.37	-0.08	0.08	34.70	0.01	-0.13	0.97	1.54E+00
1-oleoyl-2-docosahexaenoyl-GPC (18:1/22:6)*	1.04	0.32	-0.15	1.12	30.46	0.00	-0.12	0.97	4.45E-08
1-linoleoyl-2-arachidonoyl-GPC (18:2/20:4n6)*	0.99	0.25	-0.62	0.68	25.17	0.00	-0.12	1.00	2.60E-08
1-linoleoyl-2-docosahexaenoyl-GPC (18:2/22:6)*	1.04	0.39	-0.01	0.43	37.09	0.00	-0.12	0.96	2.18E-07
1-palmityl-2-linoleoyl-GPC (O-16:0/18:2)*	1.07	0.42	0.10	0.44	39.35	0.00	-0.12	0.94	0.00E+00
1-(1-enyl-stearoyl)-2-linoleoyl-GPC (P-18:0/18:2)*	1.03	0.36	-0.12	-0.15	35.46	0.00	-0.12	0.97	3.54E+00
1-myristoyl-2-linoleoyl-GPC (14:0/18:2)*	1.04	0.37	-0.30	0.04	35.80	0.00	-0.12	0.98	0.00E+00
1-myristoyl-2-arachidonoyl-GPC (14:0/20:4)*	1.08	0.50	-0.49	0.65	46.75	0.00	-0.12	0.97	0.00E+00
1-myristoyl-2-docosahexaenoyl-GPC (14:0/22:6)*	1.14	0.73	-0.14	1.59	63.82	0.01	-0.13	0.86	0.00E+00
1-stearoyl-2-docosapentaenoyl-GPC (18:0/22:5n3)*	1.04	0.37	-0.14	-0.06	35.35	0.00	-0.12	0.97	2.14E-07
1-stearoyl-2-docosapentaenoyl-GPC (18:0/22:5n6)*	1.13	0.56	-0.76	1.94	50.07	0.00	-0.12	0.97	1.40E-06
1-palmitoyl-2-adrenoyl-GPC (16:0/22:4)*	1.02	0.36	-0.51	0.20	35.01	0.00	-0.12	0.99	0.00E+00
1-stearoyl-2-adrenoyl-GPC (18:0/22:4)*	1.05	0.44	-0.15	0.17	42.15	0.00	-0.12	0.96	0.00E+00
1-stearyl-GPC (O-18:0)*	1.14	0.63	0.30	0.25	55.54	0.33	-0.53	0.81	0.00E+00
1-myristoyl-2-palmitoleoyl-GPC (14:0/16:1)*	1.28	1.04	-0.55	-0.05	81.88	0.13	-0.38	0.90	9.06E+00
1-stearoyl-2-meadoyl-GPC (18:0/20:3n9)*	1.10	0.45	-0.28	-1.34	41.12	0.47	-0.54	0.96	1.38E+01
1-(1-enyl-palmitoyl)-2-palmitoleoyl-GPC (P-16:0/16:1)*	1.05	0.31	0.16	-0.18	29.51	0.00	-0.12	0.97	0.00E+00
1-(1-enyl-palmitoyl)-2-myristoyl-GPC (P-16:0/14:0)*	1.09	0.42	-0.38	0.04	39.11	0.08	-0.29	0.97	2.81E+00
1-(1-enyl-palmitoyl)-2-palmitoyl-GPC (P-16:0/16:0)*	1.03	0.25	0.07	-0.22	24.67	0.00	-0.12	0.98	0.00E+00
phosphatidylcholine (16:0/22:5n3, 18:1/20:4)*	0.99	0.25	-0.46	-0.02	25.39	0.00	-0.12	1.00	7.15E-01
phosphatidylcholine (18:0/20:5, 16:0/22:5n6)*	1.13	0.56	-0.18	-1.62	49.37	0.60	-0.50	0.91	8.26E-01
1-palmitoyl-2-docosahexaenoyl-GPI (16:0/22:6)*	1.20	0.80	-0.21	-0.07	66.82	0.11	-0.35	0.89	NA
1-stearoyl-2-oleoyl-GPI (18:0/18:1)*	1.08	0.54	-1.68	7.04	49.64	0.02	-0.18	0.95	0.00E+00
1-stearoyl-2-dihomo-linolenoyl-GPI (18:0/20:3n3 or 6)*	1.04	0.39	-0.16	-0.20	37.94	0.00	-0.12	0.98	0.00E+00
1-stearyl-GPE (O-18:0)*	1.21	0.77	0.41	-0.26	64.08	0.48	-0.54	0.80	2.73E+01
1,2-dipalmitoyl-GPE (16:0/16:0)*	1.11	0.63	0.98	0.21	56.46	0.84	-0.28	0.84	
1-palmitoyl-2-stearoyl-GPE (16:0/18:0)*	1.11	0.59	0.18	-0.94	53.05	0.45	-0.55	0.91	
1-palmitoyl-2-eicosapentaenoyl-GPE (16:0/20:5)*	1.23	0.85	0.12	-1.14	68.93	0.50	-0.54	0.90	6.34E+00
1-stearoyl-2-dihomo-linolenoyl-GPE (18:0/20:3n3 or 6)*	1.11	0.58	-0.19	0.31	52.44	0.00	-0.12	0.95	0.00E+00
1,2-dilinoleoyl-GPE (18:2/18:2)*	1.21	0.85	0.49	-0.66	70.21	0.53	-0.53	0.87	5.57E+01
1-oleoyl-2-arachidonoyl-GPE (18:1/20:4)*	1.05	0.43	-0.40	0.03	40.45	0.00	-0.12	0.98	1.28E+01
1-(1-enyl-stearoyl)-2-linoleoyl-GPE (P-18:0/18:2)*	1.03	0.34	-0.29	0.42	33.05	0.00	-0.12	0.98	0.00E+00
1-linoleoyl-GPG (18:2)*	1.10	0.45	-0.39	0.13	40.93	0.06	-0.27	0.97	6.11E+00
thioproline	1.02	0.39	-1.10	2.20	38.00	0.00	-0.12	0.99	5.34E+00
palmitoylcholine	1.09	0.52	-0.57	0.32	48.26	0.00	-0.12	0.97	0.00E+00
trans-3,4-methyleneheptanoylcarnitine	1.09	0.65	-0.85	-0.22	59.67	0.23	-0.48	0.94	
glycocholate glucuronide (1)	1.79	2.48	0.70	-0.60	139.30	0.67	-0.45	0.75	8.77E+00
glycochenodeoxycholate glucuronide (1)	1.79	3.59	-0.35	0.63	200.60	0.11	-0.34	0.57	0.00E+00
(S)-3-hydroxybutyrylcarnitine	1.27	0.85	-0.59	-0.09	67.17	0.20	-0.45	0.91	5.27E+00
glycosyl-N-palmitoyl-sphingosine (d18:1/16:0)	1.02	0.26	-0.05	-0.31	25.05	0.00	-0.12	0.99	0.00E+00
catechol glucuronide	1.56	2.04	1.71	2.85	130.96	1.02	-0.03	0.71	3.17E+01
ascorbic acid 2-sulfate	1.03	0.43	-0.75	2.03	41.79	0.00	-0.12	0.97	0.00E+00
oleoylcholine	1.11	0.57	-0.58	0.28	51.57	0.00	-0.12	0.97	0.00E+00
arachidonoylcholine	1.11	0.60	-0.30	0.01	54.07	0.00	-0.12	0.96	0.00E+00
docosahexaenoylcholine	1.12	0.68	-0.69	0.97	60.80	0.03	-0.20	0.94	0.00E+00
palmitoloelycholine	1.10	0.66	0.36	-1.25	59.89	0.70	-0.43	0.91	1.71E+01
dihomo-linolenoyl-choline	1.11	0.60	-0.77	1.07	54.39	0.02	-0.16	0.97	4.93E+00
caffeic acid sulfate	3.30	6.09	1.49	1.47	184.94	0.89	-0.22	0.72	8.79E-06
1-linoleoyl-2-linolenoyl-GPC (18:2/18:3)*	1.16	0.73	-0.41	0.68	63.16	0.03	-0.19	0.92	0.00E+00
1-palmitoleoyl-2-linolenoyl-GPC (16:1/18:3)*	1.09	0.54	-0.57	-1.17	49.97	0.42	-0.55	0.93	1.40E+01
phosphatidylcholine (14:0/14:0, 16:0/12:0)	1.49	1.82	0.00	0.13	122.38	0.05	-0.24	0.75	0.00E+00
phosphatidylcholine (15:0/18:1, 17:0/16:1, 16:0/17:1)*	1.03	0.35	-0.08	0.08	33.93	0.00	-0.12	0.97	0.00E+00
1-oleoyl-2-dihomo-linoleoyl-GPC (18:1/20:2)*	1.01	0.30	-1.33	-0.11	30.23	0.32	-0.53	0.99	1.15E+01
1-oleoyl-2-docosapentaenoyl-GPC (18:1/22:5n3)*	1.03	0.52	-1.41	1.43	51.14	0.11	-0.35	0.99	1.53E+00
phosphatidylcholine (18:0/20:2, 20:0/18:2)*	1.00	0.22	-0.33	0.42	22.04	0.00	-0.12	0.99	0.00E+00
1-(1-enyl-oleoyl)-2-docosahexaenoyl-GPE (P-18:1/22:6)*	1.07	0.51	-0.83	0.97	48.26	0.05	-0.24	0.97	0.00E+00
1-(1-enyl-stearoyl)-2-dihomo-linolenoyl-GPE (P-18:0/20:3)*	1.06	0.44	-0.11	-0.03	41.52	0.00	-0.12	0.97	0.00E+00
lisinopril	1.02	0.55	5.22	26.37	53.89	4.82	22.02	0.98	
lamotrigine	1.98	1.29	16.08	270.08	65.13	13.03	168.78	1.00	2.21E+01
hexadecatrienoate (16:3n3)	1.41	1.24	0.40	-0.97	88.49	0.60	-0.50	0.81	0.00E+00
hexadecadienoate (16:2n6)	1.28	0.84	-0.43	0.36	66.14	0.00	-0.12	0.92	1.01E+01
1-myristoyl-2-eicosapentaenoyl-GPC (14:0/20:5)*	1.21	1.07	-0.40	1.66	89.01	0.03	-0.19	0.79	0.00E+00
palmitoleoylcarnitine (C16:1)*	1.12	0.70	0.33	1.28	62.83	0.00	-0.12	0.83	0.00E+00
4-acetamidobenzoate	4.37	30.97	1.78	5.52	709.54	0.99	-0.08	0.28	0.00E+00
4-hydroxyphenylacetylglutamine	1.13	0.67	-0.80	1.30	59.01	0.07	-0.28	0.91	1.30E-06
2'-O-methyluridine	1.13	0.43	0.50	0.84	38.41	0.06	-0.26	0.90	3.68E+00
gamma-glutamyl-alpha-lysine	1.20	0.60	0.91	2.77	50.48	0.00	-0.12	0.83	8.91E-01
palmitoyl-oleoyl-glycerol (16:0/18:1) [1]*	1.22	0.96	0.25	0.74	78.61	0.02	-0.16	0.80	
palmitoyl-oleoyl-glycerol (16:0/18:1) [2]*	1.26	0.97	0.34	0.80	77.39	0.00	-0.12	0.80	
oleoyl-oleoyl-glycerol (18:1/18:1) [1]*	1.13	0.59	0.00	0.08	52.12	0.00	-0.12	0.93	
oleoyl-oleoyl-glycerol (18:1/18:1) [2]*	1.14	0.64	-0.20	0.24	56.29	0.00	-0.12	0.93	
linoleoyl-arachidonoyl-glycerol (18:2/20:4) [1]*	1.18	0.69	-0.29	0.69	58.73	0.00	-0.12	0.93	
linoleoyl-arachidonoyl-glycerol (18:2/20:4) [2]*	1.15	0.90	-1.21	1.19	78.17	0.09	-0.31	0.88	
palmitoyl-arachidonoyl-glycerol (16:0/20:4) [1]*	1.49	1.77	-0.16	-0.29	119.55	0.24	-0.48	0.73	
palmitoyl-arachidonoyl-glycerol (16:0/20:4) [2]*	1.33	1.56	-0.16	0.58	117.39	0.12	-0.36	0.68	
linoleoyl-linolenoyl-glycerol (18:2/18:3) [1]*	1.37	1.24	-0.09	-1.08	90.03	0.40	-0.55	0.87	
linoleoyl-linolenoyl-glycerol (18:2/18:3) [2]*	1.20	0.91	-0.13	0.31	75.82	0.00	-0.12	0.89	
linoleoyl-docosahexaenoyl-glycerol (18:2/22:6) [2]*	1.28	1.17	-0.44	-0.24	91.71	0.18	-0.43	0.83	
palmitoleoyl-linoleoyl-glycerol (16:1/18:2) [1]*	1.36	1.43	-0.32	-0.80	105.41	0.27	-0.51	0.83	1.51E+01

Supplementary Table 1: Distribution of raw and transformed metabolites, the correlation between raw and transformed metabolite values, and coefficient of variation of raw metabolites

diacylglycerol (14:0/18:1, 16:0/16:1) [1]*	1.47	2.06	0.17	-0.85	140.99	0.47	-0.54	0.67	
diacylglycerol (14:0/18:1, 16:0/16:1) [2]*	1.55	2.04	-0.19	-0.25	132.24	0.23	-0.47	0.68	
oleoyl-arachidonoyl-glycerol (18:1/20:4) [1]*	1.14	0.66	0.12	0.71	57.63	0.00	-0.12	0.89	1.33E+01
oleoyl-arachidonoyl-glycerol (18:1/20:4) [2]*	1.13	0.68	0.04	0.70	60.30	0.02	-0.16	0.87	1.01E+01
palmitoyl-linolenoyl-glycerol (16:0/18:3) [2]*	1.33	1.30	0.21	-1.40	98.24	0.64	-0.47	0.81	
diacylglycerol (16:1/18:2 [2], 16:0/18:3 [1])*	1.33	1.03	-0.34	0.01	77.35	0.01	-0.13	0.93	1.11E+01
linoleoyl-linoleoyl-glycerol (18:2/18:2) [1]*	1.35	1.18	0.05	-0.13	87.20	0.00	-0.12	0.87	6.83E+00
linoleoyl-linoleoyl-glycerol (18:2/18:2) [2]*	1.12	0.67	-0.91	1.01	59.74	0.07	-0.28	0.96	7.16E+00
stearoyl-arachidonoyl-glycerol (18:0/20:4) [1]*	1.10	0.45	0.10	0.26	40.73	0.00	-0.12	0.95	
stearoyl-arachidonoyl-glycerol (18:0/20:4) [2]*	1.08	0.37	-1.64	3.63	34.35	0.10	-0.32	0.95	
perfluorooctanesulfonate (PFOS)	1.27	1.08	-1.14	4.11	85.46	0.01	-0.13	0.87	0.00E+00
1-palmityl-GPE (O-16:0)*	1.39	1.17	0.88	0.71	84.22	0.29	-0.51	0.80	1.96E+01
1-palmityl-2-stearoyl-GPC (O-16:0/18:0)*	1.02	0.28	-0.32	0.26	27.31	0.00	-0.12	0.99	4.49E+00
1-palmityl-2-palmitoyl-GPC (O-16:0/16:0)*	1.03	0.26	-0.16	-0.30	25.00	0.00	-0.12	0.99	0.00E+00
1-stearyl-2-docosapentaenoyl-GPC (O-18:0/22:5n3)*	0.99	0.27	-0.25	0.01	26.86	0.00	-0.12	0.99	0.00E+00
1-stearyl-2-linoleoyl-GPC (O-18:0/18:2)*	1.05	0.34	-0.08	-0.08	32.84	0.00	-0.12	0.98	0.00E+00
1-stearoyl-2-docosapentaenoyl-GPE (18:0/22:5n3)*	1.10	0.55	-0.27	0.44	49.61	0.03	-0.20	0.93	2.52E+01
1-stearoyl-2-docosapentaenoyl-GPE (18:0/22:5n6)*	1.27	1.12	-0.30	0.45	88.47	0.03	-0.20	0.81	6.75E+00
1-stearoyl-2-adrenoyl-GPE (18:0/22:4)*	1.20	0.88	-0.23	0.95	73.30	0.01	-0.15	0.83	1.22E+01
1-(1-enyl-stearoyl)-2-docosapentaenoyl-GPE (P-18:0/22:5n3)*	1.06	0.35	-0.27	0.45	32.79	0.00	-0.12	0.98	0.00E+00
N-palmitoyl-sphingadienine (d18:2/16:0)*	1.01	0.29	-0.20	0.03	28.26	0.00	-0.12	0.99	3.27E+00
lactosyl-N-nervonoyl-sphingosine (d18:1/24:1)*	1.02	0.32	-0.12	-0.04	31.73	0.00	-0.12	0.98	0.00E+00
glycosyl-N-behenoyl-sphingosine (d18:1/22:0)*	1.02	0.28	-0.23	0.20	27.75	0.00	-0.12	0.99	
lactosyl-N-behenoyl-sphingosine (d18:1/22:0)*	1.04	0.33	-1.81	6.30	31.41	0.04	-0.21	0.98	8.92E+00
N-behenoyl-sphingadienine (d18:2/22:0)*	1.03	0.34	-0.62	2.19	33.23	0.00	-0.12	0.97	
glycosyl-N-behenoyl-sphingadienine (d18:2/22:0)*	1.03	0.35	-0.66	1.11	34.65	0.00	-0.12	0.99	0.00E+00
N-stearoyl-sphingadienine (d18:2/18:0)*	1.06	0.40	-0.47	0.65	38.40	0.00	-0.12	0.98	9.30E+00
N-palmitoylserine	1.06	0.40	-0.52	-1.40	38.27	0.48	-0.54	0.97	2.82E+01
N-oleoylserine	1.05	0.40	-0.26	-0.13	37.98	0.06	-0.26	0.97	7.04E+00
sphingadienine	1.04	0.31	-0.18	0.81	29.94	0.02	-0.16	0.97	7.26E+00
palmitoleoyl-arachidonoyl-glycerol (16:1/20:4) [2]*	1.23	1.01	-1.19	2.47	82.00	0.07	-0.28	0.88	
myristoyl-linoleoyl-glycerol (14:0/18:2) [2]*	1.35	1.22	-0.26	-0.28	90.79	0.11	-0.35	0.87	3.13E+01
phosphatidylethanolamine (P-18:1/20:4, P-16:0/22:5n3)*	1.07	0.38	0.16	0.03	35.50	0.00	-0.12	0.96	0.00E+00
1-stearoyl-2-(hydroxylinoleoyl)-GPC (18:0/18:2(OH))*	10.10	33.27	2.19	5.14	329.85	0.18	-0.43	0.55	0.00E+00
1-palmitoyl-2-(hydroxylinoleoyl)-GPC (16:0/18:2(OH))*	6.17	19.56	2.85	7.31	317.50	0.00	-0.12	0.55	3.98E+01
hexadecasphingosine (d16:1)*	1.01	0.31	-1.04	1.86	30.51	0.05	-0.25	0.99	
ceramide (d16:1/24:1, d18:1/22:1)*	1.03	0.40	-0.71	1.38	38.94	0.02	-0.16	0.98	2.31E+01
N-palmitoyl-heptadecasphingosine (d17:1/16:0)*	1.03	0.43	-1.00	0.30	41.74	0.18	-0.43	0.98	2.90E+00
ceramide (d18:1/14:0, d16:1/16:0)*	1.06	0.45	-0.26	0.41	42.68	0.01	-0.13	0.97	0.00E+00
ceramide (d18:1/17:0, d17:1/18:0)*	1.06	0.41	-0.58	0.57	38.57	0.04	-0.21	0.98	
ceramide (d18:2/24:1, d18:1/24:2)*	1.04	0.33	-0.19	-0.06	31.58	0.00	-0.12	0.98	3.24E-01
glycosyl ceramide (d18:2/24:1, d18:1/24:2)*	1.03	0.37	-0.17	0.18	35.71	0.00	-0.12	0.97	6.58E-08
glycosyl-N-tricosanoyl-sphingadienine (d18:2/23:0)*	1.04	0.43	-0.60	0.99	40.92	0.01	-0.13	0.98	
glycosyl ceramide (d18:1/23:1, d17:1/24:1)*	1.02	0.43	-0.91	-0.28	42.36	0.24	-0.49	0.98	
glycosyl-N-(2-hydroxynervonoyl)-sphingosine (d18:1/24:1(2OH))*	1.08	0.51	-0.26	0.10	46.93	0.00	-0.12	0.97	1.18E+01
ceramide (d18:1/20:0, d16:1/22:0, d20:1/18:0)*	1.03	0.31	-0.47	1.21	29.72	0.00	-0.12	0.98	
stearoylcholine*	1.11	0.59	-0.76	1.01	53.04	0.00	-0.12	0.97	0.00E+00
linoleoylcholine*	1.08	0.55	-0.59	0.42	51.46	0.00	-0.12	0.97	0.00E+00
1-adrenoyl-GPE (22:4)*	1.20	0.78	-0.74	0.96	64.74	0.09	-0.31	0.91	1.33E-07
1-docosapentaenoyl-GPE (22:5n3)*	1.09	0.48	-0.82	2.14	44.02	0.02	-0.18	0.96	2.92E+00
trazadone	1.33	1.24	6.04	36.23	93.22	5.76	31.94	0.88	3.18E+01
nisinate (24:6n3)	1.42	1.74	0.74	-0.69	122.43	0.86	-0.26	0.68	
sphingomyelin (d18:0/20:0, d16:0/22:0)*	1.08	0.46	-0.07	0.09	42.54	0.00	-0.12	0.96	0.00E+00
sphingomyelin (d18:1/19:0, d19:1/18:0)*	1.04	0.37	-0.56	1.04	35.43	0.00	-0.12	0.99	0.00E+00
sphingomyelin (d18:2/18:1)*	1.02	0.29	-0.32	-0.07	28.45	0.00	-0.12	0.99	8.78E-01
sphingomyelin (d18:2/24:2)*	1.06	0.33	0.07	-0.15	31.17	0.00	-0.12	0.97	0.00E+00
sphingomyelin (d18:2/21:0, d16:2/23:0)*	1.05	0.39	-0.71	2.05	36.96	0.00	-0.12	0.98	0.00E+00
sphingomyelin (d18:2/23:1)*	1.04	0.38	-0.27	0.08	36.42	0.00	-0.12	0.98	0.00E+00
sphingomyelin (d18:1/25:0, d19:0/24:1, d20:1/23:0, d19:1/24:0)*	1.09	0.51	-0.25	0.19	47.28	0.00	-0.12	0.96	0.00E+00
sphingomyelin (d17:2/16:0, d18:2/15:0)*	1.07	0.43	-0.44	0.80	40.39	0.00	-0.12	0.98	1.30E+00
heneicosapentaenoate (21:5n3)	3.15	5.95	1.20	0.40	189.10	1.01	-0.04	0.66	0.00E+00
linolenoylcarnitine (C18:3)*	1.05	0.44	-0.40	0.21	42.29	0.02	-0.16	0.97	1.04E+00
behenoylcarnitine (C22)*	1.18	0.73	0.43	0.27	62.16	0.07	-0.28	0.87	3.54E+00
arachidoylcarnitine (C20)*	1.02	0.37	-0.51	0.60	36.73	0.01	-0.15	0.98	0.00E+00
lignoceroylcarnitine (C24)*	1.08	0.47	0.53	0.44	43.31	0.00	-0.12	0.92	0.00E+00
cerotoylcarnitine (C26)*	1.05	0.36	-0.01	-0.06	34.28	0.00	-0.12	0.97	0.00E+00
ximenoylcarnitine (C26:1)*	1.01	0.31	-0.04	-0.07	30.94	0.00	-0.12	0.98	1.82E+00
arachidonoylcarnitine (C20:4)	1.07	0.40	-0.16	-0.12	37.10	0.01	-0.13	0.98	0.00E+00
eicosenoylcarnitine (C20:1)*	1.06	0.39	-0.18	-0.11	36.40	0.00	-0.12	0.98	3.19E+00
dihomo-linoleoylcarnitine (C20:2)*	1.05	0.34	-0.10	-0.27	32.66	0.00	-0.12	0.98	0.00E+00
dihomo-linolenoylcarnitine (C20:3n3 or 6)*	1.04	0.33	-0.28	0.19	31.79	0.00	-0.12	0.99	0.00E+00
erucoylcarnitine (C22:1)*	1.10	0.55	-0.20	-0.63	50.33	0.24	-0.48	0.93	
nervonoylcarnitine (C24:1)*	1.04	0.33	-0.32	0.16	31.94	0.02	-0.18	0.98	0.00E+00
adrenoylcarnitine (C22:4)*	1.09	0.41	-0.15	-1.30	37.53	0.45	-0.55	0.97	
glycosyl ceramide (d18:1/20:0, d16:1/22:0)*	1.02	0.33	-0.08	-0.01	32.43	0.00	-0.12	0.98	9.28E-01
1-lignoceroyl-2-arachidonoyl-GPC (24:0/20:4)*	1.08	0.48	-0.30	0.47	44.16	0.03	-0.20	0.96	
1-nervonoyl-2-arachidonoyl-GPC (24:1/20:4)*	1.03	0.40	-0.92	2.47	39.08	0.03	-0.20	0.96	1.68E+01
N-oxalyl glycine (NOG)	1.09	0.61	-0.51	-0.53	55.62	0.21	-0.46	0.94	5.71E+00
N,N,N-trimethyl-5-aminovalerate	1.09	0.51	-0.13	0.16	47.08	0.00	-0.12	0.96	0.00E+00
ethyl alpha-glucopyranoside	3.20	5.91	0.48	-1.05	184.92	0.66	-0.46	0.72	0.00E+00
carotene diol (1)	1.11	0.68	-2.23	9.59	61.78	0.01	-0.13	0.94	0.00E+00
carotene diol (2)	1.14	0.72	-1.38	4.29	63.72	0.03	-0.19	0.92	0.00E+00
carotene diol (3)	1.11	0.59	-1.15	1.09	53.16	0.13	-0.37	0.96	1.24E+01
cortolone glucuronide (1)	1.07	0.41	-0.58	1.42	38.07	0.02	-0.18	0.97	3.66E+00
fluconazole	1.00	0.87	13.30	178.34	87.00	13.03	168.78	1.00	1.19E+02
Fibrinopeptide B	1.60	2.30	2.49	4.78	143.97	2.38	4.38	0.76	0.00E+00
1-oleyl-2-linoleoyl-GPC (O-18:1/18:2)*	1.09	0.42	-0.38	-1.28	38.73	0.45	-0.55	0.96	1.44E+01
1-palmityl-2-dihomo-linolenoyl-GPC (O-16:0/20:3)*	1.03	0.33	-0.27	-0.15	31.90	0.01	-0.15	0.99	4.22E-01
phosphatidylcholine (O-18:1/20:4, O-16:0/22:5n3)*	1.00	0.24	0.14	0.41	23.77	0.00	-0.12	0.97	4.76E-08
1-palmitoyl-2-pentadecanoyl-GPC (16:0/15:0)*	1.06	0.40	-0.06	-0.42	38.07	0.00	-0.12	0.97	0.00E+00
(N(1) + N(8))-acetylspermidine	1.06	0.34	0.09	0.11	32.59	0.00	-0.12	0.97	9.34E-01
THC carboxylic acid	1.81	1.91	4.43	19.05	105.90	3.87	13.71	0.88	4.68E+01
THC carboxylic acid glucuronide	1.73	1.80	4.41	18.79	104.39	3.98	14.54	0.89	5.86E+01
5-dodecenoylcarnitine (C12:1)	1.21	0.80	-0.44	0.49	65.95	0.03	-0.20	0.92	4.78E+00
2-butenoylglycine	1.17	0.79	-0.09	-1.08	67.36	0.44	-0.55	0.88	2.62E+01
hydroxy-CMPF*	2.00	2.89	-0.20	0.18	144.83	0.00	-0.12	0.78	0.00E+00
3-hydroxyoleoylcarnitine	1.10	0.53	-0.52	0.12	48.46	0.06	-0.26	0.97	6.44E+00
3-hydroxyphenylacetoylglutamine	1.24	1.05	-0.04	-0.33	84.73	0.11	-0.35	0.85	7.83E+00
trans-2-hexenoylglycine	1.32	1.06	-0.36	-0.47	80.23	0.18	-0.43	0.90	1.24E+01
2-hydroxyarachidate*	1.08	0.33	0.17	0.43	30.63	0.02	-0.16	0.96	1.28E+01
N-stearoylserine*	1.06	0.40	-0.31	0.50	37.73	0.03	-0.19	0.97	3.09E+01
lyxonate	1.04	0.48	-0.88	1.85	46.43	0.04	-0.23	0.94	0.00E+00
dodecenedioate (C12:1-DC)*	1.30	1.26	0.14	1.13	97.01	0.02	-0.16	0.76	6.45E+00
hexadecenedioate (C16:1-DC)*	1.13	0.67	0.39	1.54	59.53	0.00	-0.12	0.81	0.00E+00
octadecenedioate (C18:1-DC)	1.15	0.62	0.01	-0.01	53.94	0.00	-0.12	0.93	0.00E+00
heptenedioate (C7:1-DC)*	1.08	0.57	-0.76	1.20	52.77	0.01	-0.13	0.95	0.00E+00
octadecadienedioate (C18:2-DC)*	1.57	2.38	1.17	2.68	151.45	0.00	-0.12	0.63	0.00E+00

Supplementary Table 1: Distribution of raw and transformed metabolites, the correlation between raw and transformed metabolite values, and coefficient of variation of raw metabolites

glucuronide of C12H22O4 (1)*	1.59	1.88	2.11	4.13	118.35	1.45	0.92	0.74	2.57E+01
glucuronide of C10H18O2 (1)*	2.12	4.04	1.19	0.61	190.62	0.97	-0.11	0.64	1.46E+01
3-carboxy-4-methyl-5-pentyl-2-furanpropionate (3-CMPFP)**	1.07	0.48	-0.03	0.26	44.38	0.00	-0.12	0.94	0.00E+00
methylnaphthyl sulfate (1)*	3.12	4.83	2.88	7.92	154.68	2.18	3.48	0.79	2.81E+01
methylnaphthyl sulfate (2)*	2.32	3.13	1.53	0.86	135.40	1.42	0.83	0.83	5.00E+01
glucuronide of C10H18O2 (7)*	2.87	8.36	0.42	0.32	291.26	0.23	-0.48	0.51	3.17E+01
glucuronide of C10H18O2 (8)*	2.68	5.69	1.46	1.58	212.48	1.07	0.07	0.62	4.26E+01
N-acetyl-2-aminooctanoate*	1.17	0.72	0.09	-0.12	61.79	0.00	-0.12	0.92	0.00E+00
hydroxyasparagine**	1.05	0.29	0.34	0.44	28.08	0.00	-0.12	0.95	7.01E-01
perfluorooctanoate (PFOA)	1.13	0.74	-0.83	2.61	65.90	0.00	-0.12	0.87	0.00E+00
pyroglutamylphenylalanine	1.08	0.38	-0.16	-1.15	34.86	0.45	-0.55	0.94	NA
3-hydroxybutyroylglycine**	1.25	1.01	-0.46	-0.29	81.09	0.14	-0.38	0.90	3.93E+00
carboxyibuprofen glucuronide*	2.34	2.79	4.13	16.29	119.58	3.59	11.63	0.86	0.00E+00
glyco-beta-muricholate**	1.81	4.33	0.02	0.75	239.02	0.11	-0.35	0.48	0.00E+00
N-methylhydroxyproline**	1.73	2.33	0.79	-0.58	134.82	0.85	-0.27	0.77	4.70E+01
N,N,N-trimethyl-alanylproline betaine (TMAP)	1.04	0.29	0.09	1.06	28.39	0.00	-0.12	0.95	2.06E-07
3-formylindole	0.99	0.31	-0.28	0.32	31.30	0.00	-0.12	0.98	3.48E+00
1-(1-enyl-oleoyl)-2-docosahexaenoyl-GPC (P-18:1/22:6)*	1.09	0.49	-0.01	-0.26	45.36	0.00	-0.12	0.96	0.00E+00
pyroglutamylalanine*	2.24	4.11	1.16	2.58	183.96	0.16	-0.41	0.62	NA
pyroglutamylproline*	1.41	1.38	-0.15	0.58	97.92	0.13	-0.38	0.76	3.47E+01
pyroglutamylleucine*	1.08	0.35	-0.48	0.95	32.52	0.03	-0.19	0.97	1.75E+00
pyroglutamylisoleucine*	1.06	0.32	-0.22	-1.04	29.81	0.38	-0.54	0.97	NA
gamma-glutamylcitrulline*	1.13	0.59	-0.84	1.23	52.40	0.07	-0.28	0.96	6.28E+00
resveratrol disulfate (1)*	1.13	1.13	3.75	12.78	100.10	3.59	11.63	0.90	9.67E+01
resveratrol disulfate (2)*	2.63	5.94	3.70	14.62	226.28	2.75	6.28	0.64	5.23E+01
glycine conjugate of C10H12O2*	1.14	0.59	-0.41	-1.03	52.15	0.39	-0.54	0.93	5.90E-01
glycine conjugate of C10H14O2 (1)*	1.17	0.71	-0.29	0.41	61.13	0.00	-0.12	0.94	0.00E+00
glutamine conjugate of C7H12O2*	1.59	2.04	-0.35	0.33	128.19	0.02	-0.16	0.77	0.00E+00
glutamine conjugate of C6H10O2 (1)*	1.29	1.15	0.37	0.50	89.24	0.00	-0.12	0.80	0.00E+00
glutamine conjugate of C6H10O2 (2)*	1.69	2.37	0.06	-0.03	140.64	0.00	-0.12	0.73	0.00E+00
dihydroferulic acid sulfate	2.53	5.21	1.31	1.08	206.40	0.97	-0.11	0.63	3.48E+01
sphingomyelin (d17:1/14:0, d16:1/15:0)*	1.05	0.51	-0.28	0.11	48.82	0.00	-0.12	0.96	0.00E+00
tetradecadienoate (14:2)*	1.41	1.39	0.05	0.75	98.29	0.00	-0.12	0.76	4.27E+00
8-methoxykynurenate	1.16	0.66	-0.72	0.20	56.78	0.11	-0.35	0.94	9.09E+00
3-amino-2-piperidone	1.05	0.40	0.06	0.75	37.69	0.00	-0.12	0.94	0.00E+00
N,N-dimethylalanine	1.48	1.59	0.11	-0.21	107.24	0.13	-0.37	0.79	0.00E+00
3-indoleglyoxylic acid	1.13	0.69	0.97	2.00	61.11	0.00	-0.12	0.82	1.49E+01
ethyl beta-glucopyranoside	2.08	3.31	0.16	-0.30	159.33	0.03	-0.19	0.72	0.00E+00
2-hydroxysebacate	1.24	0.75	0.04	-0.02	60.35	0.09	-0.32	0.90	1.12E+01
enterolactone sulfate	1.65	1.82	1.08	-0.15	110.57	1.04	0.01	0.84	0.00E+00
ascorbic acid 3-sulfate*	1.12	0.68	-0.34	1.22	61.26	0.00	-0.12	0.91	0.00E+00
3-hydroxyhippurate sulfate	2.25	6.09	0.50	-0.49	270.49	0.49	-0.54	0.50	1.67E+01
6-bromotryptophan	1.04	0.46	0.46	6.99	44.09	0.00	-0.12	0.71	0.00E+00
delta-CEHC	1.28	1.01	-0.39	0.87	79.49	0.04	-0.23	0.86	0.00E+00
N6,N6-dimethyllysine	1.29	0.73	-0.32	-0.77	57.03	0.22	-0.47	0.95	0.00E+00
1-carboxyethylphenylalanine	1.11	0.53	0.39	0.21	48.06	0.00	-0.12	0.91	
1-carboxyethyltyrosine	1.16	0.55	-0.13	-0.20	47.33	0.10	-0.32	0.94	
1-carboxyethylvaline	1.08	0.55	-0.32	0.07	51.25	0.01	-0.13	0.96	
1-carboxyethylleucine	1.11	0.54	0.08	0.15	48.18	0.01	-0.13	0.94	
1-carboxyethylisoleucine	1.14	0.64	0.08	0.08	56.24	0.04	-0.21	0.91	
dodecadienoate (12:2)*	1.09	0.54	-0.28	0.82	49.35	0.00	-0.12	0.92	0.00E+00
indoleacetoylcarnitine*	1.41	1.22	-0.14	-0.86	87.10	0.38	-0.54	0.82	1.30E-03
gamma-CEHC sulfate	1.85	2.27	0.99	0.11	123.02	0.82	-0.31	0.78	1.28E+01
delta-CEHC glucuronide	1.34	1.34	0.32	-0.40	99.86	0.35	-0.54	0.76	5.57E+00
N-acetyl-isoputreanine	1.06	0.45	-0.94	3.51	42.65	0.01	-0.15	0.97	0.00E+00
glucuronide of piperine metabolite C17H21NO3 (3)*	1.39	1.22	-0.26	-1.17	87.66	0.37	-0.54	0.91	1.20E+01
glucuronide of piperine metabolite C17H21NO3 (4)*	1.46	1.26	-0.65	-0.58	86.55	0.26	-0.50	0.91	4.82E+00
glucuronide of piperine metabolite C17H21NO3 (5)*	1.43	1.26	-0.54	-0.68	87.74	0.24	-0.48	0.91	0.00E+00
sulfate of piperine metabolite C16H19NO3 (2)*	1.35	1.14	-0.70	0.48	84.10	0.05	-0.25	0.92	0.00E+00
sulfate of piperine metabolite C16H19NO3 (3)*	1.31	1.14	-0.61	0.25	87.10	0.05	-0.24	0.91	0.00E+00
sulfate of piperine metabolite C18H21NO3 (1)*	1.33	1.08	-0.68	0.10	80.93	0.09	-0.32	0.92	4.50E-08
5-hydroxyindole glucuronide	1.37	2.64	0.14	-0.33	193.49	0.44	-0.55	0.42	9.23E+00
N-acetylhomocitrulline	1.32	1.00	1.11	0.02	76.08	1.11	0.13	0.84	
2-naphthol sulfate	2.36	3.86	0.16	-0.28	163.68	0.03	-0.19	0.72	0.00E+00
(2,4 or 2,5)-dimethylphenol sulfate	2.86	3.86	0.57	-0.84	135.25	0.47	-0.54	0.83	4.23E+01
4-ethylcatechol sulfate	1.51	1.68	-0.40	0.56	111.35	0.01	-0.13	0.83	1.96E-06
11beta-hydroxyandrosterone glucuronide	1.11	0.62	-1.01	2.89	56.30	0.02	-0.18	0.94	0.00E+00
11beta-hydroxyetiocholanolone glucuronide*	1.04	0.62	-0.59	-0.44	60.18	0.16	-0.41	0.96	1.81E+00
N2-acetyl,N6-methyllysine	1.51	1.62	-0.19	-1.03	107.00	0.35	-0.54	0.85	0.00E+00
cholic acid glucuronide	1.48	1.39	0.79	-0.67	94.24	0.77	-0.36	0.88	1.37E+01
deoxycholic acid glucuronide	1.35	1.27	-0.56	0.63	94.30	0.01	-0.13	0.86	0.00E+00
4-allylcatechol sulfate	2.30	4.32	0.16	0.42	188.31	0.02	-0.18	0.68	0.00E+00
2-hydroxyfluorene sulfate	2.52	4.32	1.17	0.38	171.92	0.91	-0.20	0.72	3.65E+01
vanillate glucuronide	1.44	1.01	5.06	27.34	70.11	4.09	15.46	0.86	1.29E+01
4-methylhexanoylglutamine	1.31	1.04	-0.15	-0.49	79.73	0.20	-0.45	0.87	1.36E+01
Benazeprilat	1.00	0.61	13.72	192.43	61.01	13.03	168.78	1.00	4.51E+01
1-nonadecenoyl-GPC (19:1)*	1.05	0.52	-0.75	-0.69	49.36	0.31	-0.52	0.97	6.22E+00
desmethylcitalopram*	1.69	1.58	6.14	38.84	93.63	4.82	22.02	0.91	3.82E+01
citalopram propionate*	1.21	0.94	6.44	43.79	78.06	5.01	23.93	0.90	1.14E+01
4-hydroxy duloxetine glucuronide*	0.97	0.45	18.41	338.02	46.40	18.41	338.02	1.00	1.17E+02
5-hydroxy-6-methoxy duloxetine sulfate*	0.83	0.24	18.41	338.02	28.66	18.41	338.02	1.00	1.22E+02
N-desalkylquetiapine*	0.82	0.33	18.41	338.02	40.06	18.41	338.02	1.00	5.31E+01
ranitidine N-oxide*	1.00	0.19	18.41	338.02	19.20	18.41	338.02	1.00	NA
7-hydroxywarfarin	0.94	0.13	10.54	109.47	13.51	10.63	111.92	0.84	3.20E+01
glycoursodeoxycholic acid sulfate (1)	1.94	2.51	-0.21	-1.05	129.28	0.38	-0.54	0.80	0.00E+00
dihydrocaffeate sulfate (2)	2.34	5.85	0.25	0.31	250.33	0.20	-0.45	0.53	0.00E+00
lithocholate sulfate (1)	1.39	1.29	-0.73	1.00	92.77	0.04	-0.23	0.89	0.00E+00
3-hydroxyhexanoylcarnitine (1)	1.13	0.52	-0.66	-0.84	46.19	0.35	-0.54	0.94	1.74E+01
3-(methylthio)acetaminophen sulfate*	3.14	5.91	2.03	2.81	188.64	1.71	1.69	0.72	0.00E+00
2-ketocaprylate	1.14	0.72	-1.75	5.52	63.16	0.04	-0.23	0.90	1.89E-07
2,6-dihydroxybenzoic acid	1.30	1.05	-0.05	-0.37	80.78	0.00	-0.12	0.91	0.00E+00
tetrahydrocortisol sulfate (1)	1.22	0.83	0.59	-0.96	68.22	0.73	-0.40	0.91	6.47E+00
3-ethylcatechol sulfate (1)	1.47	1.47	-0.44	-0.78	100.36	0.25	-0.49	0.89	8.10E+00
3-ethylcatechol sulfate (2)	1.25	1.10	1.38	0.64	87.94	1.19	0.29	0.87	1.17E+01
4-acetylcatechol sulfate (1)	1.35	1.44	-0.20	-0.43	106.76	0.18	-0.43	0.81	0.00E+00
1-(14 or 15-methyl)palmitoyl-GPC (a17:0 or i17:0)*	1.04	0.48	-0.32	0.44	45.93	0.01	-0.15	0.96	0.00E+00
montelukast	1.15	0.29	12.08	159.46	25.03	9.20	83.58	0.94	3.00E+01
2,3-dihydroxy-5-methylthio-4-pentenoate (DMTPA)*	1.02	0.23	0.10	0.72	22.31	0.00	-0.12	0.98	0.00E+00
Fibrinopeptide A (3-16)**	1.13	0.87	2.45	4.33	76.88	2.51	5.03	0.87	
meloxicam	1.05	0.54	11.30	137.90	50.87	9.20	83.58	0.95	1.36E+02
Fibrinopeptide B (1-13)**	2.09	3.13	2.41	4.30	150.40	2.26	3.83	0.79	0.00E+00
losartan	1.75	2.52	4.73	21.85	144.80	4.09	15.46	0.82	0.00E+00
cyclobenzaprine	1.33	1.30	5.85	34.20	97.38	5.48	28.79	0.84	8.19E+01
emtricitabine	1.00	0.16	10.63	112.00	16.40	10.63	112.06	1.00	6.94E+01
darunavir	1.57	0.53	13.07	170.23	33.89	13.03	168.78	1.00	0.00E+00
5-hydroxy-2-methylpyridine sulfate	4.10	7.73	1.32	0.74	188.81	0.93	-0.17	0.72	0.00E+00
3-hydroxy-2-methylpyridine sulfate	4.31	12.35	1.46	2.21	287.20	0.44	-0.55	0.57	0.00E+00

Supplementary Table 1: Distribution of raw and transformed metabolites, the correlation between raw and transformed metabolite values, and coefficient of variation of raw metabolites

hydroxypalmitoyl sphingomyelin (d18:1/16:0(OH))**	1.03	0.30	-0.11	0.44	29.09	0.00	-0.12	0.98	0.00E+00
tamoxifen	0.99	0.02	18.41	338.02	1.67	18.41	338.02	1.00	8.67E+00
taurochenodeoxycholic acid 3-sulfate	1.72	2.54	0.06	-1.07	147.97	0.43	-0.55	0.70	0.00E+00
tetradecadienedioate (C14:2-DC)*	1.31	1.14	-0.47	0.25	87.07	0.10	-0.33	0.83	0.00E+00
pregnenetriol sulfate*	1.14	0.69	-0.75	1.45	60.49	0.00	-0.12	0.95	0.00E+00
pregnenetriol disulfate*	1.38	1.34	-0.61	1.70	97.49	0.00	-0.12	0.83	0.00E+00
eicosenedioate (C20:1-DC)*	1.08	0.56	-0.43	0.17	51.72	0.00	-0.12	0.96	0.00E+00
hydroxy-N6,N6,N6-trimethyllysine*	1.02	0.52	0.38	-1.23	50.92	0.67	-0.45	0.94	2.72E+01
undecenoylcarnitine (C11:1)	1.03	0.41	-0.41	0.32	39.72	0.00	-0.12	0.98	0.00E+00
3-decenoylcarnitine	1.02	0.40	-0.08	-0.40	39.31	0.00	-0.12	0.97	5.60E+01
3-hydroxydecanoylcarnitine	1.11	0.57	-0.35	0.04	51.08	0.01	-0.13	0.97	0.00E+00
palmitoyl-sphingosine-phosphoethanolamine (d18:1/16:0)	1.01	0.21	-0.28	0.41	20.56	0.00	-0.12	0.99	0.00E+00
picolinoylglycine	1.12	0.60	0.12	0.30	53.80	0.00	-0.12	0.91	0.00E+00
4-vinylcatechol sulfate	2.12	3.16	-0.40	-0.21	149.03	0.18	-0.43	0.77	0.00E+00
succinoyltaurine	1.17	0.73	0.18	-0.08	62.06	0.01	-0.15	0.91	0.00E+00
phenylalanylhydroxyproline*	1.31	0.92	-0.49	-0.25	70.55	0.20	-0.45	0.89	1.71E+01
ginkgolic acid C15:1	3.25	7.54	5.86	39.35	232.43	4.49	18.90	0.62	3.97E+01
(15:3)-anacardic acid	1.59	2.24	2.83	7.51	140.97	2.32	4.10	0.71	2.04E+01
ginkgolic acid C17:1	1.44	1.69	7.59	69.11	117.97	5.01	23.93	0.71	1.45E+01
3,5-dichloro-2,6-dihydroxybenzoic acid	1.02	0.39	-1.32	4.51	38.15	0.00	-0.12	0.98	9.86E-01
metabolonic lactone sulfate	1.38	1.20	-0.19	0.47	87.05	0.01	-0.13	0.88	0.00E+00
vanillic acid glycine	1.46	1.38	-0.09	-0.86	94.87	0.27	-0.50	0.87	1.14E+01
2-hydroxy-4-(methylthio)butanoic acid	1.12	0.58	0.04	0.04	51.36	0.00	-0.12	0.94	0.00E+00
branched chain 14:0 dicarboxylic acid**	1.39	1.42	-0.58	0.05	102.20	0.08	-0.30	0.88	0.00E+00
(2-butoxyethoxy)acetic acid	3.41	13.34	2.09	4.91	391.24	1.46	0.96	0.41	0.00E+00
pentose acid*	1.46	1.70	0.29	0.22	116.76	0.01	-0.15	0.76	0.00E+00
N-succinyl-phenylalanine	1.15	0.65	0.21	-1.40	56.64	0.64	-0.47	0.93	0.00E+00
1-methyl-5-imidazolelactate	1.26	1.02	-0.53	0.68	81.45	0.00	-0.12	0.88	1.88E-06
(2 or 3)-decenoate (10:1n7 or n8)	1.23	0.70	-0.43	-0.07	57.29	0.01	-0.15	0.96	9.56E+00
chenodeoxycholic acid sulfate (1)	1.55	1.68	1.28	0.54	108.20	1.13	0.18	0.77	
branched-chain, straight-chain, or cyclopropyl 10:1 fatty acid (1)*	1.09	0.64	-0.56	0.57	59.03	0.01	-0.13	0.96	0.00E+00
branched-chain, straight-chain, or cyclopropyl 10:1 fatty acid (2)*	1.05	0.55	-0.49	-1.14	52.08	0.37	-0.54	0.97	2.09E+01
carnitine of C10H14O2 (5)*	1.13	0.64	-1.07	0.51	56.77	0.15	-0.40	0.97	3.70E+01
3-bromo-5-chloro-2,6-dihydroxybenzoic acid*	1.00	0.41	-2.57	12.91	40.77	0.00	-0.12	0.99	6.23E+00
branched-chain, straight-chain, or cyclopropyl 12:1 fatty acid*	1.29	0.79	-0.58	0.25	60.79	0.00	-0.12	0.96	0.00E+00
decadienedioic acid (C10:2-DC)**	1.26	1.13	-0.68	1.24	89.45	0.02	-0.18	0.86	0.00E+00
deoxycholic acid 12-sulfate*	1.46	1.41	-0.35	-0.49	96.34	0.19	-0.44	0.87	0.00E+00
GlcNAc sulfate conjugate of C21H34O2 steroid**	1.38	1.27	0.03	-0.98	92.06	0.36	-0.54	0.84	7.25E+00
cis-3,4-methyleneheptanoate	1.13	0.64	-0.47	0.48	57.19	0.00	-0.12	0.96	0.00E+00
cis-3,4-methyleneheptanoylcarnitine	1.13	0.57	-0.44	-0.04	50.53	0.00	-0.12	0.97	0.00E+00
N-acetyl-2-aminoadipate	1.05	0.39	-0.06	0.03	37.29	0.02	-0.16	0.97	1.92E-08
De(carboxymethoxy) cetirizine acetic acid	2.20	4.73	3.42	12.26	214.72	2.89	7.06	0.60	8.84E-06
4-chlorobenzoic acid	1.01	0.13	0.22	1.27	12.91	0.00	-0.12	0.98	0.00E+00
2-methoxyhydroquinone sulfate (1)	1.64	2.05	0.43	-0.79	124.93	0.45	-0.55	0.80	0.00E+00
temazepam	1.39	1.49	10.99	122.66	107.29	10.63	111.92	0.95	1.03E+02
2,4-di-tert-butylphenol	1.04	0.33	-0.05	1.79	32.13	0.01	-0.13	0.94	6.67E+00
3-hydroxyoctanoylcarnitine (1)	1.12	0.55	-0.35	0.15	49.12	0.02	-0.18	0.96	0.00E+00
3-hydroxyoctanoylcarnitine (2)	1.11	0.52	-0.83	0.98	47.48	0.06	-0.27	0.97	0.00E+00
cis-3,4-methyleneheptanoylglycine	1.24	0.85	0.07	-0.24	69.28	0.00	-0.12	0.91	0.00E+00
bilirubin degradation product, C16H18N2O5 (1)**	1.17	0.78	-0.58	0.09	66.63	0.11	-0.34	0.92	0.00E+00
bilirubin degradation product, C16H18N2O5 (2)**	1.14	0.70	-0.17	0.06	62.06	0.00	-0.12	0.92	3.32E+00
bilirubin degradation product, C17H18N2O4 (1)**	1.06	0.53	-2.91	11.20	49.68	0.03	-0.20	0.97	0.00E+00
bilirubin degradation product, C17H18N2O4 (2)**	1.06	0.56	-2.68	10.49	52.90	0.01	-0.13	0.97	0.00E+00
bilirubin degradation product, C17H18N2O4 (3)**	1.06	0.55	-1.89	6.17	52.01	0.00	-0.12	0.97	0.00E+00
bilirubin degradation product, C17H20N2O5 (1)**	1.22	0.94	-0.54	-0.11	77.45	0.00	-0.12	0.93	6.97E+00
bilirubin degradation product, C17H20N2O5 (2)**	1.21	0.89	-0.38	-0.33	73.75	0.00	-0.12	0.94	6.03E+00
tetrahydrocortisol glucuronide	1.04	0.47	-1.83	9.11	45.38	0.01	-0.15	0.96	0.00E+00
bilirubin degradation product, C16H18N2O5 (3)**	1.14	0.61	-0.24	0.71	53.58	0.00	-0.12	0.93	3.03E+00
bilirubin degradation product, C16H18N2O5 (4)**	1.13	0.68	-0.27	0.29	60.12	0.00	-0.12	0.92	2.18E-06
N,N-dimethyl-pro-pro	1.06	0.37	-1.92	10.04	34.66	0.02	-0.16	0.93	0.00E+00
oxindolylalanine	0.99	0.34	-1.01	0.71	34.34	0.15	-0.40	0.98	6.37E+00
tetrahydrocortisone glucuronide (5)	1.06	0.53	-0.51	1.18	49.85	0.02	-0.16	0.95	4.57E-07
perfluorohexanesulfonic acid	1.59	2.04	-0.27	1.08	128.20	0.02	-0.16	0.77	0.00E+00
trans-4-hydroxyproline	1.15	0.56	0.43	0.01	48.69	0.00	-0.12	0.92	0.00E+00
allantoin	1.03	0.31	1.49	7.01	29.96	0.00	-0.12	0.80	4.57E+00
xanthine	1.17	1.56	2.38	19.78	132.99	0.00	-0.12	0.38	0.00E+00
5-oxoproline	1.08	0.44	1.68	4.13	40.93	0.00	-0.12	0.81	4.77E+00
sarcosine	0.84	0.36	-0.36	-0.81	43.42	0.00	-0.12	0.98	6.01E+00
pantothenate	1.25	0.92	0.83	1.21	73.42	0.00	-0.12	0.81	0.00E+00
pipecolate	1.50	1.67	0.98	0.92	111.44	0.00	-0.12	0.75	0.00E+00
phosphoethanolamine	1.11	0.44	-0.44	1.32	39.62	0.00	-0.12	0.97	1.65E+00
glycerate	0.97	0.39	-0.95	0.52	40.29	0.00	-0.12	0.99	0.00E+00
3-ureidopropionate	1.09	0.49	-0.18	2.79	45.22	0.03	-0.19	0.86	1.17E+01
5-KETE	1.29	1.23	4.47	20.44	95.76	3.87	13.71	0.82	8.08E+01
N-acetylleucine	1.07	0.37	0.32	1.13	35.05	0.01	-0.13	0.92	7.64E+00
N-acetylmethionine	1.07	0.26	0.15	2.07	24.44	0.00	-0.12	0.95	0.00E+00
N-acetylvaline	1.03	0.22	0.38	1.18	21.03	0.00	-0.12	0.96	0.00E+00
erucate (22:1n9)	1.28	0.93	0.41	0.99	72.35	0.00	-0.12	0.81	0.00E+00
bilirubin (Z,Z)	0.99	0.47	-3.64	14.30	47.44	0.03	-0.19	0.98	6.42E-01
thyroxine	1.01	0.23	-0.29	0.93	22.68	0.00	-0.12	0.99	4.87E-08
gamma-glutamyltyrosine	1.17	0.61	1.19	2.80	51.93	0.00	-0.12	0.81	0.00E+00
alpha-tocopherol	0.96	0.34	-4.08	18.06	35.19	0.00	-0.12	0.97	0.00E+00
3-hydroxyisobutyrate	1.14	0.61	-0.57	3.81	53.31	0.01	-0.15	0.88	1.88E+00
N-acetylalanine	1.02	0.18	0.42	1.03	17.37	0.00	-0.12	0.97	0.00E+00
vanillylmandelate (VMA)	1.01	0.34	-0.49	0.94	34.06	0.00	-0.12	0.98	1.95E+00
4-acetamidobutanoate	1.05	0.33	0.22	0.74	31.57	0.00	-0.12	0.95	3.91E-07
3-aminoisobutyrate	1.10	0.57	-0.62	1.15	52.01	0.00	-0.12	0.96	2.74E+00
3-hydroxy-3-methylglutarate	1.09	0.43	0.96	2.19	39.25	0.00	-0.12	0.86	0.00E+00
citrate	1.02	0.16	0.17	0.34	15.46	0.00	-0.12	0.98	0.00E+00
2-aminobutyrate	1.06	0.35	-0.08	0.26	32.59	0.00	-0.12	0.97	1.86E+01
urate	1.03	0.26	-0.52	0.88	25.61	0.00	-0.12	1.00	0.00E+00
ursodeoxycholate	2.11	2.76	0.19	-0.41	130.92	0.05	-0.25	0.81	4.13E+01
oleoyl ethanolamide	1.24	0.65	-0.07	-0.04	52.64	0.00	-0.12	0.94	2.88E+00
gamma-glutamylglutamine	1.05	0.31	-2.58	18.00	29.47	0.00	-0.12	0.98	4.40E+00
4-hydroxyphenylpyruvate	1.04	0.42	-0.08	-1.45	40.16	0.56	-0.52	0.94	8.55E+00
butyrate/isobutyrate (4:0)	1.38	1.41	-0.03	-0.38	102.39	0.32	-0.53	0.77	3.62E+01
N-acetylneuraminate	1.05	0.33	0.27	2.53	31.09	0.01	-0.15	0.90	0.00E+00
homovanillate (HVA)	1.19	0.94	0.31	2.56	78.97	0.11	-0.35	0.63	1.95E+01
creatine	1.11	0.48	-0.47	0.20	43.74	0.00	-0.12	0.98	0.00E+00
cys-gly, oxidized	1.23	0.99	-0.75	0.82	80.59	0.12	-0.36	0.84	1.13E+01
2,3-diphosphoglycerate	5.19	14.28	2.19	4.80	275.44	1.15	0.21	0.58	
estrone 3-sulfate	1.87	3.14	1.72	3.02	168.15	1.15	0.21	0.61	9.53E+00
dihomo-linoleate (20:2n6)	1.28	0.78	-0.23	-0.06	61.50	0.00	-0.12	0.93	0.00E+00
gamma-glutamylhistidine	1.36	1.38	-0.20	-0.56	101.72	0.26	-0.50	0.78	1.36E+01
2-hydroxystearate	1.05	0.28	-0.19	0.07	26.99	0.00	-0.12	0.99	0.00E+00
N1-methyladenosine	1.00	0.18	-0.86	3.22	18.54	0.00	-0.12	0.99	0.00E+00
glycerol	1.16	0.56	0.13	-0.35	48.54	0.00	-0.12	0.95	4.48E+00

Supplementary Table 1: Distribution of raw and transformed metabolites, the correlation between raw and transformed metabolite values, and coefficient of variation of raw metabolites

choline	1.03	0.22	0.04	0.04	21.01	0.00	-0.12	0.99	2.64E+00
anthranilate	1.20	0.74	0.69	-0.63	61.82	0.86	-0.26	0.82	2.53E+01
gamma-glutamylleucine	1.42	1.21	1.89	3.92	84.98	0.00	-0.12	0.72	0.00E+00
3-phosphoglycerate	1.64	2.56	0.66	0.70	156.56	0.29	-0.52	0.65	3.45E+01
3-methoxytyrosine	1.08	0.33	0.28	0.48	30.71	0.00	-0.12	0.95	1.55E+00
cholate	3.28	6.78	0.57	0.07	206.97	0.05	-0.24	0.68	0.00E+00
fluoxetine	1.68	1.80	6.97	49.79	107.20	5.76	31.94	0.91	1.05E+01
4-acetamidophenol	8.95	25.45	2.55	5.48	284.93	1.92	2.45	0.60	3.31E+01
naproxen	5.39	16.89	3.18	9.10	313.72	2.84	6.78	0.54	3.87E+01
topiramate	1.00	0.55	10.07	103.98	55.25	8.22	66.44	0.97	1.15E+02
beta-hydroxyisovalerate	1.13	0.50	-0.16	1.01	44.47	0.00	-0.12	0.93	0.00E+00
ibuprofen	2.72	3.31	5.43	30.63	121.71	3.98	14.54	0.85	1.05E+02
arachidonoyl ethanolamide	1.34	1.11	0.83	1.85	82.35	0.25	-0.49	0.71	
palmitoyl ethanolamide	1.14	0.42	0.01	0.62	37.02	0.00	-0.12	0.95	1.29E+00
N-linoleoylglycine	1.17	0.60	-0.40	-1.00	51.07	0.36	-0.54	0.94	9.65E+00
N-palmitoyl-sphingosine (d18:1/16:0)	1.02	0.24	-0.03	0.16	23.79	0.00	-0.12	0.98	0.00E+00
1-palmitoyl-2-oleoyl-GPE (16:0/18:1)	1.14	0.63	0.21	0.05	55.20	0.00	-0.12	0.91	0.00E+00
1-palmitoyl-2-linoleoyl-GPI (16:0/18:2)	1.07	0.46	-0.41	0.45	43.46	0.00	-0.12	0.98	3.82E+00
1-palmitoyl-2-linoleoyl-GPC (16:0/18:2)	1.00	0.12	-0.10	-0.28	12.16	0.00	-0.12	1.00	0.00E+00
stearoyl sphingomyelin (d18:1/18:0)	1.03	0.26	-0.15	0.11	25.04	0.00	-0.12	0.99	0.00E+00
1-palmitoyl-2-oleoyl-GPC (16:0/18:1)	1.02	0.20	0.21	0.15	19.91	0.00	-0.12	0.98	0.00E+00
N-stearoyl-sphingosine (d18:1/18:0)*	1.03	0.37	-0.39	0.63	35.79	0.00	-0.12	0.98	0.00E+00
5,6-dihydrothymine	1.06	0.32	-0.31	0.81	30.24	0.01	-0.15	0.98	0.00E+00
glycochenodeoxycholate	1.63	1.93	0.04	-0.01	119.09	0.01	-0.13	0.81	0.00E+00
taurochenodeoxycholate	2.17	3.78	0.21	0.07	174.64	0.06	-0.26	0.70	0.00E+00
taurocholate	3.45	10.00	0.56	-0.02	290.35	0.29	-0.51	0.54	0.00E+00
taurodeoxycholate	2.07	3.44	-0.45	0.41	166.56	0.12	-0.36	0.68	0.00E+00
hypoxanthine	1.32	0.92	0.24	0.41	69.62	0.00	-0.12	0.87	0.00E+00
9,10-DiHOME	1.17	0.70	-0.36	0.72	60.10	0.15	-0.39	0.85	3.11E+01
linoleate (18:2n6)	1.16	0.59	-0.49	0.21	51.16	0.00	-0.12	0.97	0.00E+00
laurate (12:0)	1.40	1.12	0.13	-0.26	79.96	0.00	-0.12	0.89	0.00E+00
quinolinate	1.09	0.54	0.29	0.76	49.29	0.00	-0.12	0.89	0.00E+00
2-hydroxyhippurate (salicylurate)	22.62	74.67	1.19	0.99	330.57	0.01	-0.13	0.57	0.00E+00
N6,N6,N6-trimethyllysine	1.18	0.64	0.92	1.56	54.36	0.00	-0.12	0.85	0.00E+00
N-acetylputrescine	1.07	0.45	-0.56	7.08	41.77	0.00	-0.12	0.85	0.00E+00
N-formylmethionine	1.01	0.21	-0.14	1.27	20.86	0.00	-0.12	0.98	0.00E+00
S-adenosylhomocysteine (SAH)	1.10	0.55	0.07	1.51	49.98	0.03	-0.19	0.88	5.36E+00
metoprolol	2.20	3.28	8.53	81.64	149.29	5.23	26.16	0.75	3.81E+01
azelate (C9-DC)	1.14	0.50	0.73	1.94	44.28	0.01	-0.15	0.86	1.60E+01
3-(N-acetyl-L-cystein-S-yl) acetaminophen	3.48	5.11	2.71	6.03	146.92	2.26	3.83	0.83	0.00E+00
4-acetaminophen sulfate	46.25	123.58	1.68	1.78	267.58	1.12	0.15	0.65	0.00E+00
eicosapentaenoate (EPA; 20:5n3)	1.57	1.93	0.55	1.23	123.10	0.00	-0.12	0.69	0.00E+00
methylsuccinate	1.12	0.94	1.32	6.83	84.02	0.00	-0.12	0.56	2.28E+00
ethylmalonate	1.27	1.03	1.38	3.23	81.20	0.00	-0.12	0.72	0.00E+00
adenosine 3',5'-cyclic monophosphate (cAMP)	1.01	0.23	-0.78	1.75	22.53	0.01	-0.15	1.00	1.64E+01
adenosine 5'-monophosphate (AMP)	1.78	1.76	0.03	-0.09	99.17	0.00	-0.12	0.84	0.00E+00
5-methylthioadenosine (MTA)	1.11	0.77	1.51	12.98	69.56	0.01	-0.13	0.52	2.46E-07
N6-methyladenosine	0.99	0.28	-0.40	-0.15	28.86	0.00	-0.12	0.99	9.06E+00
arachidonate (20:4n6)	1.12	0.42	-0.25	-0.12	37.61	0.00	-0.12	0.98	0.00E+00
arginine	1.03	0.26	0.29	1.03	25.02	0.00	-0.12	0.96	3.84E+00
aspartate	1.29	0.91	1.21	1.98	71.08	0.00	-0.12	0.80	1.57E+00
2-hydroxyphenylacetate	1.16	0.69	-1.56	2.14	59.69	0.15	-0.40	0.89	5.88E+00
3-(4-hydroxyphenyl)lactate	1.06	0.38	0.04	0.30	36.31	0.00	-0.12	0.96	0.00E+00
phenylpyruvate	1.00	0.35	-0.36	0.47	35.36	0.06	-0.26	0.95	1.05E+01
beta-alanine	1.29	4.30	4.69	56.25	334.80	0.01	-0.13	0.22	0.00E+00
biliverdin	1.29	0.98	0.19	0.59	75.53	0.00	-0.12	0.86	1.71E+01
succinate	1.04	0.49	-2.61	6.27	47.19	0.00	-0.12	0.94	0.00E+00
3-hydroxybutyrate (BHBA)	2.09	3.11	0.55	0.22	148.81	0.00	-0.12	0.72	0.00E+00
cholesterol	1.02	0.23	-0.40	-0.18	22.09	0.00	-0.12	1.00	0.00E+00
corticosterone	1.27	0.88	0.11	-1.21	68.78	0.45	-0.55	0.93	0.00E+00
cortisone	0.97	0.27	-4.29	27.64	27.36	0.01	-0.15	1.00	3.63E-07
creatinine	1.02	0.17	0.01	0.10	16.47	0.00	-0.12	0.99	0.00E+00
cysteinylglycine	1.12	0.60	-0.63	1.02	53.46	0.04	-0.23	0.93	4.79E+00
cystine	1.12	0.58	0.10	-0.18	52.24	0.00	-0.12	0.94	0.00E+00
sphingosine	1.06	0.53	0.44	0.69	50.37	0.01	-0.15	0.89	0.00E+00
deoxycholate	1.31	1.49	-0.24	0.72	114.09	0.04	-0.23	0.73	0.00E+00
cystathionine	1.28	1.42	-0.35	-0.42	111.17	0.32	-0.53	0.63	0.00E+00
sphinganine	1.04	0.51	0.54	0.85	48.46	0.00	-0.12	0.89	5.01E+00
flavin adenine dinucleotide (FAD)	1.03	0.25	0.00	-1.35	24.45	0.49	-0.54	0.99	6.02E+00
fumarate	1.07	0.33	0.42	1.59	30.84	0.00	-0.12	0.92	1.93E+00
gamma-glutamylglutamate	2.64	5.47	1.56	3.32	207.21	0.03	-0.19	0.61	5.38E+00
gluconate	1.07	0.37	-0.03	2.54	34.14	0.00	-0.12	0.93	0.00E+00
glutarate (C5-DC)	1.74	2.11	0.14	2.01	121.53	0.02	-0.16	0.73	0.00E+00
glycine	1.05	0.30	0.21	0.20	28.47	0.00	-0.12	0.97	0.00E+00
glycocholate	2.28	4.39	0.49	0.32	192.87	0.00	-0.12	0.63	0.00E+00
guanidinoacetate	1.04	0.28	-0.24	1.40	27.34	0.00	-0.12	0.98	0.00E+00
S-1-pyrroline-5-carboxylate	1.01	0.42	-0.55	0.52	41.26	0.07	-0.28	0.96	3.24E+00
histidine	0.99	0.14	-0.04	-0.32	13.73	0.00	-0.12	1.00	0.00E+00
cortisol	1.02	0.37	-1.12	4.33	36.35	0.00	-0.12	0.99	0.00E+00
hypotaurine	1.06	0.48	-0.33	0.68	45.38	0.01	-0.15	0.94	0.00E+00
inosine	1.21	0.66	0.07	1.02	54.91	0.10	-0.32	0.86	1.17E+01
myo-inositol	1.03	0.29	-0.04	-0.23	28.43	0.00	-0.12	0.98	2.80E+00
isoleucine	1.03	0.21	0.01	-0.15	20.43	0.00	-0.12	0.99	5.06E-08
2-aminoadipate	1.07	0.55	-1.70	3.07	51.42	0.08	-0.30	0.97	0.00E+00
citrulline	1.02	0.26	-0.23	0.24	25.66	0.00	-0.12	0.99	7.62E-01
leucine	1.02	0.18	0.00	-0.30	17.84	0.00	-0.12	0.99	0.00E+00
lithocholate	1.66	2.01	0.78	-0.37	121.11	0.67	-0.45	0.77	6.27E+00
lysine	1.01	0.17	-0.42	0.95	17.18	0.00	-0.12	1.00	0.00E+00
malate	1.06	0.27	0.90	2.14	25.23	0.00	-0.12	0.91	NA
methionine	1.01	0.17	-0.79	2.76	16.39	0.00	-0.12	0.99	0.00E+00
methylmalonate (MMA)	1.07	0.51	-2.22	3.89	47.82	0.13	-0.38	0.93	NA
palmitate (16:0)	1.13	0.52	-0.34	0.05	45.87	0.00	-0.12	0.96	2.69E+00
nicotinamide	1.21	0.70	0.25	1.50	57.85	0.01	-0.13	0.85	1.30E+01
stearate (18:0)	1.19	0.55	-0.17	-0.13	45.92	0.00	-0.12	0.96	4.11E+00
ornithine	1.01	0.23	-0.60	1.17	22.33	0.00	-0.12	1.00	2.73E+00
orotate	1.09	0.70	1.53	8.13	64.26	0.00	-0.12	0.63	0.00E+00
palmitoleate (16:1n7)	1.54	1.45	-0.17	-0.23	94.78	0.00	-0.12	0.86	0.00E+00
phenylalanine	1.02	0.16	0.35	0.78	15.56	0.00	-0.12	0.98	8.77E-01
phosphate	0.99	0.16	-0.04	0.21	15.71	0.00	-0.12	0.99	5.16E-01
phytanate	1.35	1.34	-0.02	0.48	98.95	0.00	-0.12	0.75	0.00E+00
proline	1.05	0.29	0.39	0.04	27.52	0.00	-0.12	0.96	1.34E+00
lactate	1.12	0.41	0.10	0.25	36.83	0.00	-0.12	0.96	0.00E+00
pyridoxal	1.38	1.61	2.03	3.57	116.97	1.71	1.69	0.71	8.26E+00
retinol (Vitamin A)	0.99	0.32	-2.10	7.73	31.90	0.00	-0.12	1.00	1.58E+00
spermidine	1.27	1.21	0.54	3.22	95.57	0.06	-0.27	0.65	6.08E+00
salicylate	21.12	78.16	1.23	1.07	370.61	0.00	-0.12	0.53	5.84E+00
serine	1.03	0.21	-0.31	0.52	20.83	0.00	-0.12	0.99	0.00E+00

Supplementary Table 1: Distribution of raw and transformed metabolites, the correlation between raw and transformed metabolite values, and coefficient of variation of raw metabolites

serotonin	1.51	1.32	0.00	-1.13	87.24	0.37	-0.54	0.89	0.00E+00
taurine	1.04	0.24	0.33	0.02	23.18	0.00	-0.12	0.97	0.00E+00
myristate (14:0)	1.32	0.89	-0.21	-0.10	67.65	0.00	-0.12	0.92	0.00E+00
urea	1.03	0.35	-0.37	1.10	34.38	0.00	-0.12	0.97	0.00E+00
uridine	1.01	0.20	-0.62	1.31	19.39	0.00	-0.12	1.00	0.00E+00
2'-deoxyuridine	1.05	0.30	-0.51	0.34	28.64	0.02	-0.18	0.99	3.94E+00
trans-urocanate	1.39	0.84	-0.16	-0.43	60.45	0.00	-0.12	0.94	8.29E+00
1-methylnicotinamide	1.23	2.12	1.26	7.05	172.27	0.00	-0.12	0.44	0.00E+00
glutamate	1.41	1.22	1.17	1.60	87.08	0.00	-0.12	0.78	0.00E+00
glutamine	0.96	0.20	-3.30	14.69	21.19	0.00	-0.12	0.94	6.56E-01
threonine	1.03	0.22	0.11	-0.25	21.62	0.00	-0.12	0.98	0.00E+00
tryptophan	1.01	0.21	-0.34	1.16	20.62	0.00	-0.12	0.99	2.30E-02
valine	1.01	0.21	0.06	0.17	20.73	0.00	-0.12	0.98	0.00E+00
glucose	1.02	0.12	0.07	0.00	12.11	0.00	-0.12	0.99	5.87E-01
12,13-DiHOME	1.19	0.73	-0.42	0.74	62.08	0.09	-0.31	0.90	1.03E+01
alpha-ketobutyrate	1.10	0.49	-1.27	1.81	44.45	0.10	-0.32	0.96	0.00E+00
betaine	1.04	0.25	0.28	0.30	24.20	0.00	-0.12	0.97	0.00E+00
cysteine	1.03	0.39	-1.22	4.23	37.94	0.00	-0.12	0.95	6.67E+00
mannose	1.04	0.26	-0.07	0.17	25.11	0.00	-0.12	0.98	0.00E+00
dimethylglycine	1.10	0.43	0.69	1.84	39.67	0.00	-0.12	0.88	0.00E+00
alanine	1.04	0.29	0.14	-0.51	27.55	0.00	-0.12	0.98	2.27E+00
tyrosine	1.02	0.20	-0.11	-0.07	19.76	0.00	-0.12	0.99	0.00E+00
pseudouridine	1.01	0.19	0.27	1.26	19.06	0.00	-0.12	0.97	1.03E-07
pyruvate	1.08	0.48	-2.48	7.23	44.74	0.00	-0.12	0.99	0.00E+00
uracil	1.03	0.32	-0.45	1.63	31.32	0.03	-0.20	0.95	7.02E+00
xylose	1.00	0.41	0.13	-1.63	40.67	0.70	-0.43	0.95	5.93E+00
cytidine	1.14	0.59	0.19	1.48	51.42	0.03	-0.20	0.86	1.63E+01
arabinose	1.07	0.50	-0.26	-1.65	46.76	0.55	-0.52	0.96	4.15E-01
cotinine	1.72	2.59	1.86	1.78	150.66	1.76	1.85	0.80	4.10E+01
caffeine	1.88	3.02	-0.52	-0.18	160.73	0.13	-0.37	0.72	0.00E+00
fructose	1.04	0.25	0.32	0.87	24.14	0.00	-0.12	0.96	0.00E+00
adenine	1.06	0.44	-0.42	0.39	41.91	0.13	-0.37	0.91	1.07E+01
cytosine	1.40	1.09	1.32	0.62	78.20	1.16	0.23	0.87	0.00E+00
caprate (10:0)	1.36	1.17	0.46	0.57	86.41	0.00	-0.12	0.80	0.00E+00
margarate (17:0)	1.23	0.68	-0.18	0.10	55.73	0.00	-0.12	0.94	0.00E+00
nonadecanoate (19:0)	1.14	0.46	0.09	-0.05	40.62	0.00	-0.12	0.95	0.00E+00
arachidate (20:0)	1.20	0.56	0.77	0.81	47.13	0.00	-0.12	0.89	9.72E-01
maltose	1.54	1.34	-0.47	0.53	87.46	0.09	-0.31	0.86	0.00E+00
asparagine	0.99	0.16	-0.31	1.01	16.32	0.00	-0.12	0.99	0.00E+00
N-stearoyl-sphinganine (d18:0/18:0)*	1.18	0.75	0.08	0.30	63.42	0.00	-0.12	0.87	NA
dihydroorotate	1.00	0.62	-0.74	-0.49	61.89	0.20	-0.45	0.97	9.20E+00
alpha-ketoglutarate	1.00	0.33	-3.38	14.80	33.09	0.00	-0.12	0.95	0.00E+00
caprylate (8:0)	1.33	1.26	0.45	0.64	94.73	0.39	-0.54	0.67	4.44E+00
sucrose	46.55	755.96	0.56	4.74	1626.51	0.24	-0.49	0.17	2.62E+01
kynurenate	1.06	0.38	0.18	0.47	35.94	0.00	-0.12	0.95	0.00E+00
pentadecanoate (15:0)	1.20	0.62	-0.21	-0.11	51.98	0.00	-0.12	0.96	0.00E+00
X-07765	1.64	1.75	-1.17	1.61	107.24	0.00	-0.12	0.89	0.00E+00
X-10458	1.18	0.90	0.48	2.41	76.22	0.03	-0.19	0.73	6.03E+00
X-11299	1.35	1.35	-0.64	1.18	100.36	0.00	-0.12	0.87	2.13E+00
X-11308	1.08	0.55	-0.55	1.25	50.81	0.00	-0.12	0.95	0.00E+00
X-11315	1.10	0.57	0.09	0.24	51.71	0.00	-0.12	0.93	0.00E+00
X-11372	1.08	0.50	-0.95	3.36	46.08	0.00	-0.12	0.97	0.00E+00
X-11381	1.05	0.36	-1.13	3.02	34.18	0.00	-0.12	0.99	1.34E+00
X-11444	1.09	0.55	-1.86	9.17	50.42	0.01	-0.13	0.95	0.00E+00
X-11470	1.09	0.60	-0.79	1.20	54.91	0.03	-0.19	0.96	0.00E+00
X-11478	1.15	0.70	-0.33	0.12	60.78	0.00	-0.12	0.94	0.00E+00
X-11483	1.53	2.09	-0.14	0.54	136.74	0.01	-0.13	0.73	0.00E+00
X-11632	1.21	0.94	1.21	3.30	77.99	0.02	-0.16	0.73	1.45E+01
X-11787	1.00	0.24	-0.06	2.35	24.19	0.00	-0.12	0.96	3.37E-07
X-11795	1.55	2.56	-1.11	1.17	165.57	0.16	-0.41	0.59	0.00E+00
X-11843	4.29	8.46	-0.12	-0.83	197.55	0.18	-0.43	0.73	3.05E+01
X-11847	3.26	6.53	-0.07	-1.09	200.28	0.29	-0.51	0.71	0.00E+00
X-11849	2.52	4.62	-0.19	-1.08	183.33	0.31	-0.52	0.72	0.00E+00
X-11850	4.47	9.60	-0.02	-0.81	215.16	0.08	-0.30	0.70	2.71E+01
X-11852	4.52	10.49	0.12	-0.52	232.13	0.02	-0.16	0.65	0.00E+00
X-11858	4.21	12.43	0.67	-0.80	295.68	0.77	-0.37	0.56	0.00E+00
X-11880	1.09	0.49	-0.58	0.88	45.46	0.00	-0.12	0.98	1.60E+00
X-11979	1.05	0.35	-0.34	0.20	33.06	0.12	-0.36	0.96	2.56E+01
X-12007	1.54	2.15	-0.11	0.34	140.19	0.04	-0.23	0.68	0.00E+00
X-12013	2.87	5.83	0.38	-0.63	203.05	0.36	-0.54	0.65	0.00E+00
X-12026	1.05	0.36	-0.24	0.93	34.67	0.00	-0.12	0.97	0.00E+00
X-12027	3.11	6.45	2.63	7.26	207.48	1.54	1.18	0.66	
X-12100	1.08	0.37	1.29	3.91	34.33	0.00	-0.12	0.84	3.34E+00
X-12101	1.22	0.87	0.21	0.20	70.99	0.00	-0.12	0.88	0.00E+00
X-12104	1.02	0.31	0.02	0.17	30.63	0.00	-0.12	0.97	0.00E+00
X-12111	1.77	2.33	0.82	-0.52	131.98	0.85	-0.27	0.76	
X-12193	1.13	0.52	-0.32	0.18	46.49	0.11	-0.35	0.94	3.36E+01
X-12216	1.32	1.12	-0.53	0.26	85.03	0.05	-0.24	0.91	0.00E+00
X-12221	1.62	2.04	1.35	1.13	126.45	1.00	-0.06	0.74	0.00E+00
X-12261	2.34	3.57	0.49	-1.09	152.72	0.58	-0.51	0.78	2.44E+01
X-12262	1.76	2.29	1.14	-0.04	130.14	1.13	0.18	0.78	3.41E+01
X-12306	1.38	1.44	-0.07	-1.05	104.85	0.37	-0.54	0.82	0.00E+00
X-12407	1.53	1.59	0.39	-1.06	104.12	0.59	-0.50	0.83	0.00E+00
X-12410	1.28	0.99	-0.51	0.02	77.45	0.20	-0.45	0.87	0.00E+00
X-12411	1.24	1.74	0.81	3.76	139.97	0.00	-0.12	0.49	0.00E+00
X-12456	1.38	1.14	-0.20	-0.75	82.94	0.20	-0.45	0.90	1.24E+01
X-12544	13.92	58.29	1.94	3.90	419.46	0.05	-0.25	0.49	0.00E+00
X-12680	1.20	0.83	0.10	0.54	69.13	0.02	-0.16	0.87	2.80E-06
X-12701	1.54	2.36	2.59	7.54	153.83	1.72	1.74	0.58	0.00E+00
X-12707	1.19	0.83	0.14	1.16	70.03	0.03	-0.19	0.77	1.29E+01
X-12714	1.27	1.25	1.41	1.74	98.18	0.99	-0.08	0.70	1.85E+01
X-12726	1.64	2.29	0.22	-0.04	140.10	0.20	-0.45	0.72	2.17E+01
X-12729	1.81	3.64	0.25	1.04	201.61	0.04	-0.21	0.53	0.00E+00
X-12730	4.45	9.09	1.77	2.61	204.79	1.03	-0.01	0.69	0.00E+00
X-12731	1.15	0.80	0.86	-0.34	69.86	0.77	-0.37	0.88	0.00E+00
X-12738	2.47	3.38	1.92	2.82	137.14	1.42	0.83	0.80	0.00E+00
X-12740	2.71	4.77	-0.16	-0.96	176.42	0.28	-0.51	0.75	0.00E+00
X-12798	1.09	0.62	-1.32	1.30	57.37	0.13	-0.38	0.95	5.23E+00
X-12812	1.59	2.92	0.05	0.34	183.30	0.12	-0.36	0.59	0.00E+00
X-12815	2.52	3.78	2.12	3.77	150.12	1.71	1.69	0.75	0.00E+00
X-12816	1.25	1.20	0.39	-1.59	95.81	0.83	-0.30	0.91	0.00E+00
X-12822	1.12	0.65	-0.08	0.57	57.70	0.00	-0.12	0.91	2.94E+00
X-12830	1.54	1.72	-0.10	-1.30	112.24	0.41	-0.55	0.85	0.00E+00
X-12839	1.48	1.43	-0.10	-0.97	97.09	0.35	-0.54	0.85	1.40E+01
X-12844	1.01	0.41	-3.02	20.18	40.43	0.01	-0.13	0.98	0.00E+00
X-12847	2.40	3.89	-0.31	0.27	162.24	0.04	-0.21	0.74	0.00E+00
X-12849	1.69	2.67	-0.67	1.24	158.38	0.04	-0.23	0.66	

Supplementary Table 1: Distribution of raw and transformed metabolites, the correlation between raw and transformed metabolite values, and coefficient of variation of raw metabolites

X-12851	2.00	3.02	-0.48	-0.31	151.15	0.19	-0.44	0.76	0.00E+00
X-12906	1.01	0.40	-0.03	0.47	39.21	0.00	-0.12	0.95	7.68E+00
X-13431	1.14	0.65	0.10	0.94	57.13	0.01	-0.13	0.88	0.00E+00
X-13507	1.25	1.04	0.21	-1.16	82.90	0.63	-0.48	0.80	1.07E+01
X-13553	1.12	0.56	0.31	0.34	49.96	0.01	-0.15	0.91	0.00E+00
X-13658	6.66	49.59	1.52	4.49	746.16	0.71	-0.42	0.29	0.00E+00
X-13695	1.42	1.47	1.00	0.32	103.83	0.77	-0.37	0.76	1.46E+01
X-13723	1.38	1.69	1.43	1.05	122.95	1.33	0.60	0.71	
X-13726	1.90	2.65	0.82	-0.16	139.87	0.63	-0.48	0.72	2.71E+01
X-13728	1.35	1.33	-1.11	1.18	98.93	0.07	-0.28	0.89	0.00E+00
X-13729	1.21	0.82	-0.83	1.30	67.93	0.06	-0.27	0.91	2.74E+00
X-13844	1.52	1.78	-0.01	-0.69	117.22	0.21	-0.46	0.84	4.87E+00
X-13846	1.78	1.87	3.53	12.32	105.48	2.71	6.04	0.86	1.64E-06
X-13866	1.46	1.33	0.24	-0.27	91.80	0.00	-0.12	0.86	3.48E-07
X-14056	1.28	0.85	0.04	-0.10	66.24	0.00	-0.12	0.92	3.09E+00
X-14939	1.09	0.56	-0.21	0.45	51.42	0.00	-0.12	0.95	0.00E+00
X-15486	1.17	0.73	-0.46	0.93	62.42	0.00	-0.12	0.94	0.00E+00
X-15503	1.07	0.39	-0.02	-0.22	36.59	0.00	-0.12	0.97	0.00E+00
X-15728	2.33	4.51	-0.13	-0.25	193.88	0.10	-0.33	0.69	0.00E+00
X-16087	1.19	0.78	-1.26	1.98	65.70	0.11	-0.35	0.91	7.71E+00
X-16124	26.13	72.50	1.12	0.53	277.88	0.41	-0.55	0.64	1.95E+01
X-16397	1.08	0.51	-0.58	-0.80	47.57	0.28	-0.51	0.97	0.00E+00
X-16576	1.18	0.93	-1.04	0.31	79.07	0.27	-0.50	0.69	1.39E+01
X-16580	1.12	0.60	-0.40	0.17	53.72	0.01	-0.15	0.96	3.06E+00
X-16649	5.85	13.87	1.45	1.10	237.44	1.07	0.07	0.65	4.95E+01
X-16935	1.24	0.95	-0.57	0.44	76.68	0.01	-0.13	0.93	0.00E+00
X-16964	0.97	0.30	-1.68	4.32	30.88	0.02	-0.18	1.00	2.45E+01
X-17010	1.77	4.09	1.72	3.71	231.80	0.00	-0.12	0.57	0.00E+00
X-17146	1.73	3.25	0.26	-1.54	187.92	0.07	-0.28	0.76	7.93E+01
X-17162	2.19	4.14	0.89	-0.10	189.61	0.76	-0.37	0.62	2.85E+01
X-17301	1.23	1.13	2.18	3.26	91.70	2.20	3.59	0.86	NA
X-17306	1.85	2.50	5.13	27.69	135.57	4.34	17.62	0.77	NA
X-17325	1.93	2.34	0.13	-0.96	121.40	0.29	-0.51	0.84	1.63E+01
X-17335	1.05	0.45	-0.43	0.13	43.01	0.01	-0.13	0.98	2.77E+00
X-17346	1.49	1.79	0.64	0.50	120.25	0.20	-0.45	0.72	1.36E+01
X-17348	2.48	4.52	1.58	1.57	182.20	1.29	0.53	0.68	0.00E+00
X-17351	1.34	1.23	-0.06	0.31	92.27	0.02	-0.16	0.85	0.00E+00
X-17353	1.42	1.58	0.73	-0.50	111.16	0.79	-0.34	0.74	2.20E+00
X-17354	2.57	5.27	0.83	-0.14	205.15	0.62	-0.49	0.63	0.00E+00
X-17357	1.06	0.49	-1.78	4.69	46.14	0.07	-0.28	0.95	0.00E+00
X-17365	1.44	1.74	0.39	-0.94	121.25	0.59	-0.50	0.74	0.00E+00
X-17367	1.77	2.06	0.35	-1.04	116.95	0.50	-0.54	0.84	3.02E+01
X-17438	1.51	1.64	-0.12	0.24	108.74	0.06	-0.27	0.82	6.12E+00
X-17612	1.23	0.94	-0.63	-0.21	76.80	0.19	-0.44	0.89	2.97E+01
X-17653	1.05	0.45	-0.47	1.19	42.71	0.00	-0.12	0.97	4.51E+00
X-17654	1.10	0.52	-0.42	-0.98	47.29	0.36	-0.54	0.94	2.11E+01
X-17655	3.14	5.24	2.36	4.52	166.90	2.10	3.16	0.78	2.28E-05
X-17676	1.09	0.47	-0.03	-0.25	43.13	0.00	-0.12	0.97	0.00E+00
X-17682	1.13	0.59	0.97	-0.33	52.59	0.95	-0.14	0.94	
X-17685	3.30	6.53	0.39	-0.65	198.26	0.33	-0.53	0.69	1.92E+01
X-17686	1.88	2.80	2.12	4.26	149.13	1.44	0.89	0.72	1.14E+01
X-17690	2.81	4.88	-0.12	-0.55	174.07	0.11	-0.34	0.74	0.00E+00
X-17692	2.46	4.17	2.14	4.02	169.85	1.53	1.14	0.66	5.45E+01
X-17735	1.19	0.73	-0.06	-1.30	62.05	0.46	-0.54	0.93	1.38E+01
X-17761	1.10	0.67	-0.37	-1.02	60.78	0.35	-0.54	0.93	4.79E+00
X-17765	1.62	1.98	3.69	14.52	122.94	2.67	5.82	0.76	NA
X-18240	3.29	5.36	3.16	9.90	162.97	2.23	3.71	0.75	NA
X-18345	1.49	1.46	0.88	0.65	98.11	0.01	-0.13	0.77	2.18E+01
X-18779	0.93	0.32	-2.36	9.09	34.44	0.02	-0.16	0.99	3.72E+00
X-18838	1.22	0.72	0.45	-0.69	59.42	0.60	-0.50	0.82	
X-18886	1.10	0.53	-0.14	-0.10	48.34	0.01	-0.15	0.96	0.00E+00
X-18887	1.10	0.49	-0.28	0.98	44.97	0.01	-0.13	0.95	0.00E+00
X-18888	1.32	1.19	-0.16	-0.31	90.47	0.24	-0.48	0.81	1.92E+01
X-18899	1.10	0.71	-0.15	2.61	64.71	0.01	-0.13	0.76	1.60E+01
X-18901	2.00	3.58	0.44	0.37	179.41	0.00	-0.12	0.67	0.00E+00
X-18913	1.14	0.66	0.60	1.96	58.36	0.00	-0.12	0.83	0.00E+00
X-18921	1.13	0.67	-0.30	-0.03	58.89	0.00	-0.12	0.95	0.00E+00
X-18922	1.06	0.46	-0.19	0.28	43.74	0.00	-0.12	0.95	0.00E+00
X-18935	1.22	0.85	0.69	-0.84	70.33	0.82	-0.31	0.90	1.44E+01
X-19141	1.06	0.54	-0.38	-0.13	50.98	0.00	-0.12	0.97	0.00E+00
X-19183	1.88	2.40	2.26	4.10	127.44	1.76	1.85	0.81	2.17E+01
X-19299	2.22	3.82	-0.44	-0.49	172.68	0.27	-0.50	0.69	3.16E+01
X-19438	1.21	0.71	-1.01	-0.46	58.84	0.31	-0.52	0.94	0.00E+00
X-21258	1.51	2.77	-0.11	1.53	183.05	0.00	-0.12	0.55	0.00E+00
X-21286	1.15	0.62	-0.54	0.58	54.21	0.01	-0.13	0.96	0.00E+00
X-21310	1.02	0.37	-1.00	2.00	36.92	0.00	-0.12	1.00	0.00E+00
X-21312	1.56	1.72	0.40	-0.65	110.31	0.40	-0.55	0.80	2.30E+01
X-21315	18.42	89.54	1.94	3.63	486.68	1.22	0.37	0.43	0.00E+00
X-21319	1.10	0.59	-0.37	0.71	54.07	0.00	-0.12	0.94	6.04E+00
X-21339	1.10	0.54	-0.57	0.19	49.47	0.00	-0.12	0.98	0.00E+00
X-21351	1.58	1.37	0.10	-0.15	86.95	0.04	-0.23	0.87	1.69E+01
X-21353	1.24	0.66	-0.07	-0.26	53.55	0.00	-0.12	0.95	1.80E+00
X-21364	1.10	0.60	-0.57	1.13	54.35	0.01	-0.15	0.94	0.00E+00
X-21383	1.34	1.47	-0.08	1.66	110.34	0.01	-0.15	0.74	0.00E+00
X-21441	1.27	0.96	-0.52	-0.04	75.68	0.14	-0.38	0.90	0.00E+00
X-21442	2.22	5.82	-0.01	-1.22	262.03	0.45	-0.55	0.56	0.00E+00
X-21467	1.22	0.88	-0.43	0.08	72.13	0.13	-0.37	0.89	0.00E+00
X-21470	1.57	2.11	-0.41	-0.07	134.77	0.17	-0.42	0.72	0.00E+00
X-21471	1.26	1.04	-0.29	0.33	82.59	0.07	-0.28	0.84	0.00E+00
X-21607	1.14	0.58	-0.66	1.89	50.75	0.04	-0.21	0.91	3.03E+00
X-21628	1.43	1.40	3.08	8.29	98.37	2.94	7.35	0.85	NA
X-21661	1.80	2.52	1.58	1.36	139.97	1.44	0.89	0.75	0.00E+00
X-21733	1.16	0.92	-0.74	0.15	79.64	0.09	-0.31	0.91	5.64E+00
X-21736	1.24	0.98	0.00	0.68	79.08	0.00	-0.12	0.82	0.00E+00
X-21740	1.00	0.30	0.17	-1.53	29.62	0.65	-0.47	0.99	
X-21742	5.00	17.51	0.22	-0.96	351.01	0.49	-0.54	0.48	5.45E+01
X-21752	2.14	4.59	-0.02	-0.95	215.25	0.35	-0.54	0.61	2.14E+01
X-21788	1.20	0.73	0.67	-0.58	60.91	0.73	-0.40	0.85	0.00E+00
X-21796	1.34	1.33	1.75	5.32	99.47	0.00	-0.12	0.64	4.59E+00
X-21803	2.77	4.74	0.89	-0.44	171.34	0.81	-0.32	0.74	0.00E+00
X-21807	1.23	1.10	1.70	2.05	89.47	1.39	0.77	0.80	
X-21815	2.21	3.89	0.72	-0.67	176.23	0.69	-0.44	0.70	0.00E+00
X-21816	1.54	1.51	1.16	0.26	98.11	1.05	0.03	0.81	1.65E+01
X-21821	1.38	1.34	0.00	-0.70	97.04	0.24	-0.49	0.85	1.56E+00
X-21830	2.01	3.20	1.27	0.57	159.58	1.02	-0.03	0.74	3.08E+01
X-21831	1.54	1.74	0.74	-0.47	113.49	0.74	-0.39	0.75	1.81E+01
X-21834	1.38	1.67	-0.35	0.16	121.67	0.11	-0.35	0.74	0.00E+00

Supplementary Table 1: Distribution of raw and transformed metabolites, the correlation between raw and transformed metabolite values, and coefficient of variation of raw metabolites

X-21838	3.19	5.00	2.82	7.38	156.82	2.18	3.48	0.79	6.80E+00
X-21839	1.43	1.31	0.57	-1.11	91.89	0.77	-0.37	0.88	5.35E+00
X-21840	1.75	2.27	0.82	-0.10	130.09	0.62	-0.49	0.75	3.33E+01
X-21842	1.88	3.90	0.80	0.18	207.84	0.65	-0.47	0.57	0.00E+00
X-21845	1.49	1.41	0.34	-1.45	94.99	0.75	-0.38	0.88	1.21E+01
X-21851	1.13	0.67	0.54	-0.94	59.27	0.70	-0.43	0.90	
X-22162	1.04	0.45	-0.21	0.71	43.72	0.00	-0.12	0.94	0.00E+00
X-22509	2.13	4.05	0.52	-0.57	190.36	0.52	-0.53	0.67	0.00E+00
X-22771	0.98	0.29	-2.59	11.94	29.94	0.01	-0.15	1.00	1.42E+01
X-22776	2.17	8.13	2.87	11.69	375.86	0.03	-0.20	0.38	0.00E+00
X-22834	1.38	1.60	-0.41	-0.10	116.78	0.15	-0.40	0.73	1.30E+01
X-23276	0.96	0.43	0.38	-1.00	45.32	0.52	-0.53	0.95	4.78E+01
X-23481	1.15	0.94	-0.10	2.21	82.07	0.21	-0.46	0.59	5.98E+01
X-23587	1.92	2.71	0.45	-0.64	141.09	0.47	-0.54	0.74	2.54E+01
X-23593	1.05	0.34	-0.27	0.37	32.59	0.02	-0.18	0.98	5.20E+00
X-23636	0.94	0.30	-0.35	0.52	32.09	0.00	-0.12	0.98	0.00E+00
X-23639	1.09	0.58	0.46	0.51	53.11	0.00	-0.12	0.88	0.00E+00
X-23641	1.23	0.78	-0.19	-1.30	63.24	0.50	-0.54	0.87	1.24E+01
X-23644	2.32	4.05	0.10	-0.38	174.68	0.00	-0.12	0.72	6.23E-07
X-23654	1.05	0.39	-1.22	0.82	37.16	0.19	-0.44	0.98	6.86E+00
X-23659	1.13	0.53	-0.81	-0.11	47.44	0.23	-0.48	0.95	0.00E+00
X-23678	1.10	0.52	-0.79	-0.17	47.12	0.22	-0.47	0.96	9.52E+00
X-23680	1.05	0.47	-0.90	2.15	44.35	0.01	-0.13	0.97	0.00E+00
X-23739	1.11	0.49	-1.00	1.15	43.72	0.15	-0.40	0.92	0.00E+00
X-23767	1.90	2.99	1.38	0.97	157.74	1.19	0.29	0.69	
X-23780	1.38	1.44	0.03	-1.38	104.47	0.59	-0.50	0.75	1.79E+01
X-23782	1.15	0.67	0.28	0.37	58.11	0.00	-0.12	0.89	7.21E+00
X-23787	1.20	0.94	-0.66	0.39	78.65	0.11	-0.35	0.86	9.56E+00
X-23890	2.02	2.79	2.24	4.52	138.06	1.58	1.30	0.75	
X-23974	1.14	0.58	-0.48	-1.01	51.47	0.41	-0.55	0.88	7.85E+00
X-23997	1.28	0.98	-0.61	-0.26	76.85	0.15	-0.39	0.93	8.41E+00
X-24295	1.20	0.75	-1.92	4.32	62.46	0.07	-0.28	0.92	0.00E+00
X-24306	0.92	0.34	-1.92	10.69	36.81	0.01	-0.13	0.99	9.62E+00
X-24307	0.91	0.41	-0.73	0.03	45.15	0.00	-0.12	0.99	4.87E+00
X-24309	1.10	0.50	-0.41	0.29	45.79	0.01	-0.13	0.97	
X-24328	1.20	0.76	-0.19	0.04	63.48	0.01	-0.15	0.93	5.15E+00
X-24334	1.07	0.51	-0.06	0.05	47.96	0.01	-0.13	0.95	1.84E+00
X-24337	1.21	0.77	0.34	0.39	63.93	0.00	-0.12	0.86	0.00E+00
X-24338	1.11	0.66	2.17	3.65	59.71	1.81	2.04	0.93	
X-24344	1.56	1.65	0.90	-0.56	105.53	0.89	-0.22	0.85	1.52E+01
X-24352	2.76	8.77	1.47	1.74	317.81	1.01	-0.04	0.44	2.43E+01
X-24414	1.72	1.80	1.40	0.92	104.91	1.19	0.29	0.81	
X-24418	1.16	0.75	-0.31	-0.42	64.58	0.20	-0.45	0.91	1.71E+01
X-24456	1.19	0.59	0.45	-1.45	50.15	0.89	-0.22	0.94	
X-24475	1.95	2.77	-0.17	-0.40	142.29	0.20	-0.45	0.79	0.00E+00
X-24494	1.16	0.73	-0.46	-0.06	63.17	0.15	-0.40	0.90	1.09E+01
X-24541	2.02	2.53	0.91	-0.33	125.23	0.79	-0.34	0.82	3.79E+01
X-24543	3.13	4.90	2.05	3.35	156.95	1.48	0.99	0.76	3.66E+01
X-24544	1.17	0.68	-0.30	0.35	58.07	0.02	-0.18	0.94	8.01E-01
X-24545	1.23	1.06	0.87	0.07	86.73	0.70	-0.43	0.77	
X-24546	1.46	1.54	-0.47	-0.52	105.75	0.25	-0.49	0.80	0.00E+00
X-24556	1.12	0.57	-0.60	0.95	51.27	0.03	-0.20	0.95	0.00E+00
X-24565	1.68	2.91	0.74	-0.28	173.99	0.82	-0.31	0.59	5.06E+01
X-24571	2.97	8.05	1.14	0.80	271.47	0.73	-0.40	0.55	2.68E+01
X-24576	1.18	0.74	5.02	24.62	63.13	4.65	20.36	0.98	0.00E+00
X-24588	1.10	0.48	-0.04	-0.26	43.92	0.00	-0.12	0.96	0.00E+00
X-24657	2.22	4.18	1.83	3.18	188.11	1.20	0.33	0.62	
X-24736	1.17	0.79	0.05	-1.63	67.69	0.68	-0.44	0.90	1.22E+01
X-24747	1.14	0.64	-0.73	1.26	56.17	0.03	-0.19	0.96	
X-24748	1.13	0.91	1.13	5.96	80.51	0.00	-0.12	0.59	
X-24757	1.57	1.56	0.59	-0.89	99.72	0.70	-0.43	0.86	2.85E+01
X-24761	1.81	2.26	2.65	6.32	125.03	2.10	3.16	0.81	0.00E+00
X-24762	1.24	0.99	1.04	1.96	80.30	0.43	-0.55	0.71	
X-24947	1.60	1.87	-0.30	0.18	117.48	0.06	-0.26	0.82	1.56E+00
X-24949	1.16	0.69	-0.15	0.27	59.11	0.00	-0.12	0.93	0.00E+00
X-24951	1.13	0.67	-0.62	0.89	59.64	0.02	-0.18	0.94	1.12E+01
X-24953	1.28	0.97	-0.33	0.00	75.86	0.06	-0.26	0.90	2.25E+01
X-24970	0.83	0.74	-0.79	-0.56	89.97	0.02	-0.18	0.92	6.01E+00
X-24980	1.79	2.40	0.38	-1.09	134.37	0.64	-0.47	0.78	
X-25009	1.20	0.78	1.30	5.18	64.95	0.01	-0.13	0.69	1.55E+01
X-25172	0.97	0.38	-0.86	0.90	39.59	0.00	-0.12	0.99	3.79E+00
X-25217	2.51	4.25	0.99	-0.17	169.15	0.83	-0.30	0.73	0.00E+00
X-25247	1.49	1.33	1.43	1.00	89.34	1.16	0.23	0.86	0.00E+00
X-25271	4.52	9.63	-0.19	-0.44	213.57	0.10	-0.32	0.69	2.96E+01
X-25279	10.77	67.96	1.48	3.08	631.81	0.68	-0.44	0.35	7.94E+01
X-25343	1.08	0.46	-1.64	4.34	42.88	0.03	-0.19	0.98	1.04E+01
X-25371	1.07	0.38	-0.01	-0.07	36.04	0.00	-0.12	0.97	0.00E+00
X-25417	1.95	2.62	1.65	1.61	134.10	1.50	1.07	0.76	2.90E+01
X-25419	1.63	2.00	0.06	-1.13	122.83	0.47	-0.54	0.79	2.31E+01
X-25420	1.27	0.92	-0.17	-1.21	72.05	0.43	-0.55	0.90	6.17E+00
X-25422	1.17	0.76	0.16	-0.01	64.53	0.00	-0.12	0.90	1.42E+00
X-25433	1.31	1.16	0.37	-0.92	88.87	0.56	-0.52	0.83	0.00E+00
X-25450	7.09	47.61	4.35	28.27	672.70	1.67	1.58	0.31	0.00E+00
X-25454	1.38	1.31	-0.04	0.02	95.23	0.21	-0.46	0.79	
X-25457	1.17	0.61	-0.35	-1.09	51.95	0.35	-0.54	0.96	1.67E+01
X-25468	1.14	0.78	2.93	7.07	68.33	2.84	6.78	0.96	NA
X-25519	1.10	0.46	-0.40	0.11	41.84	0.21	-0.46	0.89	1.96E+01
X-25520	1.45	1.83	-0.07	0.09	125.97	0.13	-0.38	0.70	0.00E+00
X-25524	2.93	5.56	2.30	4.37	189.94	1.85	2.17	0.68	
X-25656	2.74	8.88	1.52	3.44	324.17	0.57	-0.51	0.44	
X-25790	1.21	0.79	-0.63	-0.29	65.58	0.24	-0.48	0.91	0.00E+00
X-25805	1.90	3.09	2.34	5.34	162.96	1.61	1.39	0.67	0.00E+00
X-25810	1.27	1.13	0.86	2.44	89.64	0.00	-0.12	0.69	0.00E+00
X-25937	1.29	1.10	-0.19	-1.00	85.22	0.27	-0.50	0.89	3.07E+00
X-25957	1.14	0.60	1.43	5.34	52.95	0.00	-0.12	0.73	3.19E+01
X-25991	1.05	0.42	0.03	-1.59	40.33	0.61	-0.49	0.99	
X-26054	1.06	0.45	0.07	0.76	42.37	0.01	-0.15	0.93	3.06E+01
X-26062	12.71	73.07	1.06	0.28	575.55	0.75	-0.39	0.35	7.87E+01
X-26106	1.07	0.46	-0.25	-0.42	43.45	0.00	-0.12	0.98	0.00E+00
X-26107	1.04	0.39	-0.24	-0.46	37.90	0.00	-0.12	0.98	0.00E+00
X-26108	1.03	0.38	-0.87	1.75	37.07	0.05	-0.24	0.97	6.18E+00
X-26109	1.36	1.19	0.11	-1.04	87.69	0.34	-0.53	0.89	9.07E+00
X-26111	1.52	1.58	-0.41	0.18	104.35	0.01	-0.15	0.86	1.49E+01
X-26119	1.11	0.64	-0.51	1.88	57.97	0.02	-0.16	0.90	

Supplementary Table 2: Parameter estimates for metabolome-wide association studies for diet-metabolite associations for each of: The Healthy Eating Index-2015 (HEI-15), DASH and AMED diet

Supplementary Table 2: Parameter estimates for metabolome-wide association studies for diet-metabolite associations for each of: The Healthy Eating Index-2015 (HEI-15), The Dietary Appro

Metabolite		HEI-15			DASH			AMED	
	Beta	SE	P	Beta	SE	P	Beta	SE	P
carnitine	-2.49E-03	5.97E-03	6.77E-01	-1.32E-02	1.37E-02	3.38E-01	1.98E-02	3.21E-02	5.38E-01
3-phenylpropionate (hydrocinnamate)	2.10E-02	5.64E-03	2.40E-04	4.36E-02	1.30E-02	9.47E-04	6.70E-02	3.09E-02	3.09E-02
phenylacetate	6.25E-03	5.96E-03	2.95E-01	3.31E-02	1.36E-02	1.56E-02	2.29E-02	3.22E-02	4.78E-01
hippurate	2.18E-02	5.70E-03	1.60E-04	5.45E-02	1.31E-02	4.02E-05	7.14E-02	3.12E-02	2.29E-02
xanthurenate	3.22E-03	5.41E-03	5.52E-01	2.73E-03	1.25E-02	8.26E-01	-1.25E-02	2.92E-02	6.69E-01
suberate (C8-DC)	-1.23E-03	5.79E-03	8.32E-01	1.38E-02	1.33E-02	2.99E-01	8.86E-03	3.12E-02	7.77E-01
3-methyl-2-oxovalerate	-3.95E-03	5.41E-03	4.66E-01	-8.08E-03	1.25E-02	5.17E-01	-9.33E-03	2.92E-02	7.49E-01
methionine sulfoxide	1.84E-02	5.85E-03	1.84E-03	4.09E-02	1.35E-02	2.62E-03	9.94E-02	3.15E-02	1.80E-03
3-methylhistidine	-6.67E-03	6.22E-03	2.84E-01	-8.62E-03	1.43E-02	5.48E-01	-4.84E-02	3.34E-02	1.49E-01
4-acetamidophenylglucuronide	7.13E-03	4.59E-03	1.21E-01	2.51E-02	1.05E-02	1.75E-02	2.31E-02	2.48E-02	3.52E-01
5-hydroxylysine	-1.99E-02	5.73E-03	5.86E-04	-2.32E-02	1.34E-02	8.50E-02	-8.37E-02	3.11E-02	7.62E-03
4-guanidinobutanoate	1.44E-02	5.33E-03	7.22E-03	2.83E-02	1.23E-02	2.20E-02	6.53E-02	2.88E-02	2.43E-02
pimelate (C7-DC)	4.14E-03	5.07E-03	4.16E-01	1.74E-02	1.16E-02	1.36E-01	4.76E-02	2.72E-02	8.15E-02
glucuronate	9.68E-04	5.77E-03	8.67E-01	6.57E-03	1.33E-02	6.21E-01	-2.87E-02	3.10E-02	3.56E-01
glycerol 3-phosphate	-3.13E-04	5.96E-03	9.58E-01	-2.86E-03	1.37E-02	8.35E-01	9.86E-03	3.21E-02	7.59E-01
imidazole lactate	-4.56E-03	5.74E-03	4.28E-01	-1.40E-02	1.32E-02	2.89E-01	2.55E-02	3.09E-02	4.10E-01
kynurenine	-5.46E-03	5.98E-03	3.62E-01	1.12E-02	1.38E-02	4.16E-01	-7.53E-02	3.20E-02	1.91E-02
glycerophosphorylcholine (GPC)	1.48E-02	6.10E-03	1.58E-02	1.03E-02	1.42E-02	4.69E-01	6.76E-02	3.30E-02	4.13E-02
N-acetylglutamate	-1.46E-02	5.38E-03	7.14E-03	-6.47E-03	1.26E-02	6.07E-01	-3.52E-02	2.93E-02	2.31E-01
tartarate	2.34E-02	4.95E-03	3.65E-06	3.59E-02	1.17E-02	2.27E-03	9.26E-02	2.72E-02	7.56E-04
xanthosine	-1.23E-02	5.75E-03	3.33E-02	1.70E-02	1.33E-02	2.02E-01	-8.49E-02	3.08E-02	6.25E-03
ribitol	-4.69E-03	5.87E-03	4.25E-01	2.81E-03	1.35E-02	8.36E-01	-7.08E-03	3.17E-02	8.23E-01
2-isopropylmalate	1.07E-02	5.72E-03	6.29E-02	5.78E-03	1.32E-02	6.63E-01	7.06E-02	3.07E-02	2.22E-02
glycodeoxycholate	3.42E-03	5.92E-03	5.64E-01	-6.72E-03	1.36E-02	6.22E-01	-3.23E-02	3.19E-02	3.12E-01
theophylline	-1.69E-02	5.78E-03	3.79E-03	-3.54E-02	1.33E-02	8.32E-03	-6.17E-02	3.14E-02	5.01E-02
quinate	1.19E-02	5.40E-03	2.86E-02	2.79E-03	1.25E-02	8.24E-01	1.94E-02	2.93E-02	5.09E-01
theobromine	-1.21E-02	5.69E-03	3.41E-02	-1.77E-03	1.32E-02	8.94E-01	-2.39E-02	3.09E-02	4.39E-01
gentisate	1.77E-02	5.41E-03	1.21E-03	3.10E-02	1.26E-02	1.42E-02	4.33E-02	2.96E-02	1.45E-01
paraxanthine	-1.47E-02	5.79E-03	1.16E-02	-2.99E-02	1.34E-02	2.58E-02	-5.16E-02	3.14E-02	1.02E-01
indolelactate	5.30E-03	5.22E-03	3.10E-01	6.23E-03	1.20E-02	6.05E-01	2.72E-02	2.81E-02	3.34E-01
3-indoxyl sulfate	-4.72E-03	6.02E-03	4.34E-01	6.83E-03	1.39E-02	6.22E-01	-4.79E-02	3.24E-02	1.39E-01
gamma-glutamylphenylalanine	-9.50E-03	5.55E-03	8.79E-02	7.48E-03	1.28E-02	5.60E-01	-4.73E-02	2.99E-02	1.15E-01
4-methyl-2-oxopentanoate	-1.48E-03	5.51E-03	7.88E-01	-1.26E-02	1.27E-02	3.21E-01	-1.37E-02	2.97E-02	6.45E-01
1,5-anhydroglucitol (1,5-AG)	-1.60E-02	5.61E-03	4.72E-03	-1.45E-02	1.31E-02	2.68E-01	-6.17E-02	3.04E-02	4.34E-02
2-arachidonoylglycerol (20:4)	5.18E-03	5.35E-03	3.34E-01	-1.94E-04	1.23E-02	9.87E-01	2.24E-02	2.88E-02	4.39E-01
1-palmityl-GPC (O-16:0)	2.40E-03	6.16E-03	6.97E-01	-6.98E-03	1.42E-02	6.23E-01	-1.32E-02	3.32E-02	6.91E-01
1-stearoyl-2-arachidonoyl-GPI (18:0/20:4)	-1.64E-04	6.00E-03	9.78E-01	-2.29E-02	1.38E-02	9.75E-02	2.56E-02	3.23E-02	4.29E-01
sphingosine 1-phosphate	-6.06E-03	6.07E-03	3.19E-01	-1.10E-02	1.40E-02	4.32E-01	-2.24E-02	3.28E-02	4.95E-01
1-stearoyl-2-oleoyl-GPS (18:0/18:1)	-1.28E-03	6.25E-03	8.37E-01	9.80E-03	1.44E-02	4.96E-01	2.38E-02	3.36E-02	4.79E-01
1-stearoyl-GPI (18:0)	6.66E-03	5.83E-03	2.54E-01	-4.20E-03	1.34E-02	7.55E-01	4.47E-02	3.14E-02	1.55E-01
1,2-dipalmitoyl-GPC (16:0/16:0)	-7.29E-03	6.06E-03	2.30E-01	-3.60E-02	1.38E-02	9.57E-03	-1.84E-02	3.27E-02	5.75E-01
docosahexaenoate (DHA; 22:6n3)	6.08E-04	5.83E-03	9.17E-01	-2.14E-02	1.34E-02	1.11E-01	3.50E-02	3.13E-02	2.65E-01
1-myristoyl-2-palmitoyl-GPC (14:0/16:0)	-6.53E-03	5.97E-03	2.74E-01	-2.82E-02	1.37E-02	4.01E-02	-4.82E-02	3.21E-02	1.35E-01
alpha-hydroxyisocaproate	-2.88E-03	5.23E-03	5.82E-01	-1.69E-02	1.20E-02	1.60E-01	-9.20E-03	2.82E-02	7.45E-01
maleate	2.85E-03	5.70E-03	6.17E-01	1.12E-03	1.31E-02	9.32E-01	3.30E-02	3.07E-02	2.83E-01
isovalerate (i5:0)	-5.99E-03	5.34E-03	2.63E-01	-6.00E-03	1.23E-02	6.27E-01	-3.01E-02	2.88E-02	2.96E-01
4-acetylphenol sulfate	1.92E-02	5.70E-03	8.76E-04	5.86E-02	1.29E-02	8.60E-06	6.82E-02	3.11E-02	2.90E-02
2-hydroxyoctanoate	3.66E-03	6.18E-03	5.54E-01	1.60E-02	1.42E-02	2.61E-01	-1.21E-02	3.33E-02	7.16E-01
3-hydroxyoctanoate	-6.59E-03	5.72E-03	2.50E-01	-2.35E-03	1.32E-02	8.59E-01	-2.87E-02	3.09E-02	3.54E-01
phenyllactate (PLA)	8.35E-04	4.93E-03	8.66E-01	2.38E-03	1.14E-02	8.34E-01	2.16E-02	2.66E-02	4.17E-01
palmitoylcarnitine (C16)	-2.16E-02	5.72E-03	1.90E-04	-5.65E-02	1.31E-02	2.10E-05	-1.04E-01	3.10E-02	8.75E-04
hexanoylcarnitine (C6)	-1.51E-02	5.93E-03	1.15E-02	-3.79E-02	1.36E-02	5.80E-03	-2.69E-02	3.23E-02	4.06E-01
theanine	2.86E-03	1.41E-03	4.40E-02	5.85E-03	3.26E-03	7.42E-02	9.39E-03	7.66E-03	2.21E-01
N-acetylaspartate (NAA)	6.42E-04	6.06E-03	9.16E-01	2.66E-02	1.39E-02	5.61E-02	-2.52E-02	3.26E-02	4.41E-01
dehydroepiandrosterone sulfate (DHEA-S)	-1.01E-02	5.38E-03	6.18E-02	-1.77E-02	1.24E-02	1.56E-01	-6.42E-02	2.89E-02	2.71E-02
acetylcarnitine (C2)	-3.93E-04	5.97E-03	9.48E-01	-6.40E-04	1.37E-02	9.63E-01	6.65E-03	3.22E-02	8.36E-01
cysteine s-sulfate	-2.07E-02	5.98E-03	6.35E-04	-3.84E-02	1.39E-02	5.94E-03	-1.15E-01	3.22E-02	4.01E-04
1-palmitoylglycerol (16:0)	-2.33E-03	6.10E-03	7.03E-01	-1.21E-02	1.40E-02	3.91E-01	-2.27E-02	3.29E-02	4.90E-01
tartronate (hydroxymalonate)	3.01E-02	5.24E-03	2.46E-08	6.23E-02	1.22E-02	5.93E-07	1.27E-01	2.89E-02	1.55E-05
oxalate (ethanedioate)	2.99E-02	5.53E-03	1.32E-07	6.69E-02	1.28E-02	3.06E-07	1.24E-01	3.04E-02	6.06E-05
erythritol	3.61E-03	5.91E-03	5.42E-01	1.57E-02	1.36E-02	2.48E-01	1.05E-02	3.19E-02	7.41E-01
saccharin	-4.88E-03	5.56E-03	3.81E-01	-5.31E-03	1.28E-02	6.79E-01	-5.58E-02	2.98E-02	6.22E-02
3-hydroxymyristate	-1.32E-02	5.88E-03	2.56E-02	-3.23E-02	1.35E-02	1.77E-02	-5.61E-02	3.18E-02	7.89E-02
iminodiacetate (IDA)	-5.17E-03	6.27E-03	4.10E-01	-2.01E-03	1.44E-02	8.89E-01	-4.78E-02	3.37E-02	1.57E-01
1-oleoylglycerol (18:1)	6.46E-03	5.99E-03	2.82E-01	3.43E-03	1.38E-02	8.04E-01	-4.44E-03	3.24E-02	8.91E-01
3-methyl-2-oxobutyrate	-8.39E-03	5.79E-03	1.48E-01	-1.72E-02	1.33E-02	1.98E-01	-3.35E-02	3.13E-02	2.85E-01
1,6-anhydroglucose	-1.18E-02	5.26E-03	2.60E-02	-1.57E-02	1.22E-02	1.99E-01	-5.22E-02	2.84E-02	6.75E-02
2-oleoylglycerol (18:1)	7.96E-03	4.87E-03	1.04E-01	6.19E-03	1.13E-02	5.83E-01	5.99E-03	2.64E-02	8.20E-01
homoarginine	8.99E-03	5.44E-03	9.99E-02	4.57E-03	1.26E-02	7.17E-01	1.30E-02	2.95E-02	6.59E-01
homocitrulline	6.29E-03	6.11E-03	3.05E-01	3.34E-02	1.40E-02	1.74E-02	-2.31E-03	3.30E-02	9.44E-01
pyroglutamylglutamine	-1.63E-03	5.86E-03	7.81E-01	1.22E-03	1.35E-02	9.28E-01	-6.46E-02	3.14E-02	4.02E-02
bradykinin	3.21E-03	4.28E-03	4.53E-01	5.75E-03	9.84E-03	5.60E-01	1.43E-02	2.30E-02	5.35E-01
glycylproline	-1.16E-03	4.85E-03	8.11E-01	-1.27E-03	1.12E-02	9.09E-01	8.83E-03	2.62E-02	7.36E-01
2-linoleoylglycerol (18:2)	6.69E-03	6.04E-03	2.69E-01	9.38E-03	1.39E-02	5.01E-01	8.53E-03	3.26E-02	7.94E-01
3-hydroxydecanoate	-7.70E-03	5.78E-03	1.84E-01	-1.09E-02	1.33E-02	4.16E-01	-3.78E-02	3.12E-02	2.27E-01
citramalate	1.46E-02	5.67E-03	1.05E-02	2.68E-02	1.31E-02	4.14E-02	5.27E-02	3.08E-02	8.75E-02
EDTA	-7.20E-03	5.65E-03	2.03E-01	-2.66E-02	1.29E-02	4.08E-02	-5.47E-02	3.03E-02	7.26E-02
N-acetylglycine	2.73E-04	5.95E-03	9.63E-01	-1.19E-02	1.37E-02	3.85E-01	2.40E-02	3.20E-02	4.55E-01
ribonate	3.41E-03	5.88E-03	5.62E-01	9.27E-03	1.35E-02	4.94E-01	-4.42E-02	3.16E-02	1.63E-01
threonate	2.12E-02	5.44E-03	1.24E-04	5.35E-02	1.24E-02	2.34E-05	7.81E-02	2.97E-02	9.11E-03
galactonate	3.82E-03	5.65E-03	4.99E-01	1.06E-02	1.30E-02	4.15E-01	-1.04E-02	3.04E-02	7.34E-01

beta-sitosterol	2.10E-02	5.64E-03	2.40E-04	2.64E-02	1.32E-02	4.66E-02	1.37E-01	3.00E-02	7.25E-06
indoleacetate	-1.51E-03	5.49E-03	7.84E-01	-9.02E-03	1.26E-02	4.76E-01	-4.69E-03	2.96E-02	8.74E-01
1-linoleoylglycerol (18:2)	5.78E-03	6.01E-03	3.37E-01	1.02E-02	1.38E-02	4.60E-01	2.66E-03	3.25E-02	9.35E-01
1-methylhistidine	-4.23E-03	5.87E-03	4.72E-01	-1.28E-02	1.35E-02	3.45E-01	-2.58E-02	3.16E-02	4.16E-01
butyrylcarnitine (C4)	-1.15E-02	5.86E-03	5.03E-02	-2.50E-02	1.35E-02	6.55E-02	-5.70E-02	3.16E-02	7.25E-02
isobutyrylcarnitine (C4)	5.85E-03	5.98E-03	3.28E-01	3.13E-02	1.37E-02	2.28E-02	5.88E-02	3.21E-02	6.80E-02
glycolithocholate	1.01E-03	5.63E-03	8.57E-01	-4.93E-03	1.30E-02	7.04E-01	-4.99E-02	3.02E-02	9.94E-02
androsterone sulfate	-1.40E-02	5.59E-03	1.28E-02	-2.67E-02	1.29E-02	3.98E-02	-4.66E-02	3.04E-02	1.26E-01
indolepropionate	3.08E-02	5.27E-03	1.48E-08	6.98E-02	1.22E-02	2.43E-08	1.24E-01	2.92E-02	2.72E-05
N-(2-furoyl)glycine	6.52E-03	5.76E-03	2.59E-01	7.02E-03	1.33E-02	5.98E-01	8.08E-03	3.11E-02	7.95E-01
trigonelline (N'-methylnicotinate)	1.26E-02	5.63E-03	2.55E-02	2.26E-02	1.30E-02	8.41E-02	3.73E-02	3.05E-02	2.23E-01
dodecanedioate (C12-DC)	-8.12E-03	5.68E-03	1.54E-01	1.54E-03	1.31E-02	9.06E-01	-4.19E-02	3.06E-02	1.72E-01
N-acetyltyrosine	-1.27E-02	5.72E-03	2.75E-02	-2.48E-02	1.32E-02	6.09E-02	-4.91E-02	3.09E-02	1.14E-01
1,3-dimethylurate	-1.72E-02	5.56E-03	2.14E-03	-3.54E-02	1.28E-02	6.26E-03	-7.90E-02	3.01E-02	9.10E-03
3-methylxanthine	-5.41E-03	5.64E-03	3.38E-01	1.36E-02	1.30E-02	2.94E-01	-2.29E-02	3.04E-02	4.52E-01
3-hydroxylaurate	-1.17E-02	5.73E-03	4.19E-02	-1.82E-02	1.33E-02	1.71E-01	-5.07E-02	3.10E-02	1.03E-01
pyridoxate	2.21E-02	5.47E-03	7.18E-05	5.50E-02	1.25E-02	1.63E-05	6.51E-02	3.01E-02	3.14E-02
gamma-glutamylvaline	-1.58E-02	5.82E-03	7.18E-03	-1.85E-02	1.35E-02	1.72E-01	-8.56E-02	3.13E-02	6.71E-03
pyroglutamylglycine	7.79E-03	5.97E-03	1.93E-01	1.20E-02	1.38E-02	3.85E-01	5.62E-02	3.21E-02	8.09E-02
pyroglutamylvaline	-5.23E-03	5.96E-03	3.81E-01	1.30E-02	1.37E-02	3.43E-01	-4.12E-02	3.21E-02	2.00E-01
3-hydroxysebacate	-1.16E-04	5.79E-03	9.84E-01	1.39E-02	1.33E-02	2.96E-01	-2.44E-03	3.12E-02	9.38E-01
5-hydroxyhexanoate	7.10E-03	5.17E-03	1.71E-01	2.66E-02	1.18E-02	2.55E-02	4.16E-02	2.79E-02	1.36E-01
propionylglycine	1.18E-03	5.83E-03	8.40E-01	2.10E-02	1.34E-02	1.18E-01	1.61E-02	3.14E-02	6.08E-01
butyrylglycine	6.43E-03	5.66E-03	2.57E-01	1.09E-02	1.30E-02	4.02E-01	3.05E-02	3.05E-02	3.19E-01
2-methylbutyrylglycine	4.00E-03	5.09E-03	4.33E-01	1.26E-02	1.17E-02	2.81E-01	9.18E-03	2.75E-02	7.38E-01
propionylcarnitine (C3)	-1.85E-03	5.90E-03	7.54E-01	1.24E-02	1.36E-02	3.62E-01	7.00E-03	3.18E-02	8.26E-01
pro-hydroxy-pro	-1.74E-02	5.83E-03	3.13E-03	-1.15E-02	1.36E-02	4.00E-01	-5.58E-02	3.17E-02	7.97E-02
3-hydroxy-2-ethylpropionate	-9.92E-03	5.56E-03	7.55E-02	4.73E-03	1.29E-02	7.13E-01	-6.17E-02	2.99E-02	4.00E-02
3-carboxy-4-methyl-5-propyl-2-furanpropanoate (CMPF)	1.05E-02	5.91E-03	7.61E-02	4.05E-05	1.37E-02	9.98E-01	8.74E-02	3.16E-02	6.04E-03
docosapentaenoate (n3 DPA; 22:5n3)	-1.13E-02	5.89E-03	5.68E-02	-3.24E-02	1.35E-02	1.69E-02	-3.32E-02	3.19E-02	2.98E-01
docosadienoate (22:2n6)	-1.37E-02	5.92E-03	2.11E-02	-4.38E-02	1.35E-02	1.33E-03	-6.82E-02	3.20E-02	3.37E-02
adrenate (22:4n6)	-2.64E-02	5.87E-03	9.89E-06	-5.81E-02	1.36E-02	2.51E-05	-9.97E-02	3.22E-02	2.17E-03
10-undecenoate (11:1n1)	-1.23E-02	5.79E-03	3.49E-02	-3.56E-02	1.33E-02	7.75E-03	-9.11E-02	3.10E-02	3.52E-03
4-imidazoleacetate	1.25E-02	5.56E-03	2.53E-02	3.05E-02	1.28E-02	1.77E-02	6.05E-02	3.00E-02	4.48E-02
1-methyl-4-imidazoleacetate	-8.98E-03	5.52E-03	1.05E-01	-5.45E-03	1.28E-02	6.69E-01	-4.32E-02	2.98E-02	1.48E-01
sebacate (C10-DC)	-9.57E-03	5.65E-03	9.11E-02	-8.77E-03	1.31E-02	5.02E-01	-6.58E-02	3.03E-02	3.10E-02
guanidinosuccinate	1.80E-02	5.74E-03	1.91E-03	4.47E-02	1.32E-02	7.92E-04	6.81E-02	3.12E-02	2.99E-02
delta-tocopherol	-9.41E-03	5.82E-03	1.07E-01	-1.10E-02	1.35E-02	4.14E-01	-5.70E-03	3.15E-02	8.57E-01
I-urobilinogen	-1.05E-02	4.34E-03	1.64E-02	-1.42E-02	1.00E-02	1.58E-01	-1.19E-03	2.36E-02	9.60E-01
stearidonate (18:4n3)	-1.06E-02	5.64E-03	6.13E-02	-2.91E-02	1.29E-02	2.52E-02	-2.08E-02	3.05E-02	4.97E-01
5-dodecenoate (12:1n7)	-1.64E-02	5.52E-03	3.32E-03	-3.92E-02	1.27E-02	2.19E-03	-4.96E-02	3.01E-02	1.00E-01
octanoylcarnitine (C8)	-8.40E-03	5.97E-03	1.61E-01	-3.11E-02	1.37E-02	2.38E-02	-5.21E-03	3.23E-02	8.72E-01
tauro-beta-muricholate	9.81E-03	5.31E-03	6.57E-02	2.20E-02	1.22E-02	7.31E-02	8.34E-03	2.88E-02	7.72E-01
decanoylcarnitine (C10)	-8.22E-03	6.04E-03	1.74E-01	-2.96E-02	1.38E-02	3.32E-02	-1.99E-03	3.26E-02	9.51E-01
N-acetylglutamine	-1.41E-02	5.48E-03	1.08E-02	-2.77E-02	1.27E-02	2.97E-02	-5.14E-02	2.97E-02	8.54E-02
N-acetyltryptophan	-2.95E-03	5.51E-03	5.93E-01	-1.04E-02	1.27E-02	4.13E-01	-1.43E-02	2.97E-02	6.29E-01
N-acetylphenylalanine	-1.59E-02	5.45E-03	3.83E-03	-2.98E-02	1.26E-02	1.87E-02	-5.52E-02	2.96E-02	6.37E-02
1-palmitoyl-GPC (16:0)	7.31E-03	5.92E-03	2.18E-01	-1.19E-02	1.37E-02	3.83E-01	2.29E-02	3.20E-02	4.75E-01
1-margaroyl-GPC (17:0)	1.34E-02	5.94E-03	2.52E-02	3.92E-02	1.36E-02	4.25E-03	2.78E-02	3.23E-02	3.90E-01
N-acetylarginine	-4.64E-03	5.98E-03	4.38E-01	-5.69E-03	1.38E-02	6.80E-01	2.62E-02	3.22E-02	4.16E-01
piperine	6.07E-03	6.06E-03	3.17E-01	-2.78E-02	1.39E-02	4.63E-02	5.97E-02	3.25E-02	6.76E-02
campesterol	5.01E-03	5.93E-03	3.99E-01	-1.44E-02	1.36E-02	2.93E-01	7.87E-02	3.17E-02	1.35E-02
myristoylcarnitine (C14)	-2.27E-02	5.66E-03	7.64E-05	-4.21E-02	1.32E-02	1.56E-03	-9.94E-02	3.08E-02	1.41E-03
1-stearoyl-GPC (18:0)	1.30E-02	5.99E-03	3.08E-02	1.62E-02	1.39E-02	2.43E-01	6.50E-02	3.23E-02	4.53E-02
1-oleoyl-GPC (18:1)	1.88E-02	5.57E-03	8.63E-04	2.22E-02	1.30E-02	8.84E-02	9.46E-02	3.01E-02	1.85E-03
N-acetylthreonine	-3.30E-03	5.79E-03	5.69E-01	2.08E-02	1.33E-02	1.18E-01	-1.88E-02	3.12E-02	5.48E-01
N-acetylisoleucine	-7.01E-03	5.99E-03	2.43E-01	-1.49E-02	1.38E-02	2.82E-01	-4.27E-03	3.23E-02	8.95E-01
10-nonadecenoate (19:1n9)	-2.18E-02	5.62E-03	1.37E-04	-5.27E-02	1.29E-02	5.84E-05	-1.10E-01	3.04E-02	3.35E-04
10-heptadecenoate (17:1n7)	-2.46E-02	5.38E-03	7.51E-06	-5.63E-02	1.24E-02	8.21E-06	-1.13E-01	2.93E-02	1.36E-04
hyocholate	-1.44E-02	5.68E-03	1.19E-02	-1.90E-02	1.32E-02	1.50E-01	3.44E-03	3.10E-02	9.12E-01
HWESASLLR	1.01E-03	4.75E-03	8.32E-01	7.79E-03	1.09E-02	4.77E-01	-7.30E-03	2.56E-02	7.76E-01
epiandrosterone sulfate	-9.20E-03	5.44E-03	9.21E-02	-1.89E-02	1.25E-02	1.33E-01	-3.00E-02	2.94E-02	3.09E-01
bradykinin, des-arg(9)	3.38E-03	4.08E-03	4.08E-01	1.17E-02	9.37E-03	2.13E-01	1.25E-02	2.20E-02	5.69E-01
N-acetylhistidine	-7.49E-03	5.84E-03	2.01E-01	-4.64E-03	1.35E-02	7.31E-01	-2.33E-02	3.15E-02	4.60E-01
gamma-glutamylglycine	-6.86E-03	6.03E-03	2.57E-01	1.84E-03	1.39E-02	8.95E-01	-3.22E-02	3.25E-02	3.24E-01
gamma-glutamyltryptophan	-1.92E-03	5.81E-03	7.41E-01	5.34E-03	1.34E-02	6.90E-01	8.04E-03	3.13E-02	7.97E-01
stachydrine	2.01E-02	6.06E-03	1.06E-03	5.01E-02	1.39E-02	3.73E-04	1.03E-01	3.27E-02	1.87E-03
alpha-hydroxyisovalerate	-4.12E-03	5.50E-03	4.55E-01	-3.53E-02	1.25E-02	5.09E-03	3.87E-02	2.96E-02	1.92E-01
hydroxybupropion	1.64E-03	2.60E-03	5.29E-01	1.04E-02	5.97E-03	8.29E-02	-1.35E-02	1.40E-02	3.37E-01
gamma-glutamylmethionine	-8.66E-03	5.54E-03	1.19E-01	-7.84E-03	1.28E-02	5.40E-01	-4.81E-02	2.98E-02	1.08E-01
gamma-glutamylthreonine	-1.51E-02	5.89E-03	1.09E-02	-2.20E-02	1.36E-02	1.08E-01	-3.85E-02	3.20E-02	2.30E-01
p-cresol sulfate	3.28E-03	6.00E-03	5.86E-01	2.54E-02	1.37E-02	6.57E-02	-2.96E-02	3.23E-02	3.60E-01
erythronate*	6.99E-03	5.82E-03	2.30E-01	3.46E-02	1.33E-02	9.63E-03	4.47E-02	3.13E-02	1.55E-01
Fibrinopeptide A*	4.92E-03	4.40E-03	2.65E-01	8.01E-03	1.01E-02	4.31E-01	1.07E-02	2.38E-02	6.53E-01
Fibrinopeptide A, des-ala(1)*	5.98E-03	4.47E-03	1.82E-01	1.02E-02	1.03E-02	3.24E-01	1.88E-02	2.41E-02	4.37E-01
Fibrinopeptide A, phosphono-ser(3)*	1.69E-03	4.22E-03	6.89E-01	4.95E-03	9.72E-03	6.11E-01	4.85E-03	2.28E-02	8.31E-01
XHWESASXXR*	5.63E-03	4.35E-03	1.96E-01	1.59E-02	9.99E-03	1.14E-01	1.59E-02	2.35E-02	4.99E-01
HWESASXX*	7.31E-03	5.93E-03	2.18E-01	1.89E-02	1.36E-02	1.67E-01	2.49E-02	3.20E-02	4.38E-01
HXGXA*	6.65E-03	3.85E-03	8.53E-02	1.31E-02	8.88E-03	1.42E-01	1.84E-02	2.08E-02	3.77E-01
salicyluric glucuronide*	8.17E-03	5.78E-03	1.58E-01	2.74E-02	1.32E-02	3.95E-02	-4.60E-03	3.12E-02	8.83E-01
eicosenoate (20:1)	-8.18E-03	5.74E-03	1.55E-01	-2.66E-02	1.32E-02	4.43E-02	-3.30E-02	3.10E-02	2.87E-01
linolenate [alpha or gamma; (18:3n3 or 6)]	-1.01E-02	5.52E-03	6.89E-02	-2.10E-02	1.27E-02	9.96E-02	-3.07E-02	2.99E-02	3.06E-01
aconitate [cis or trans]	-1.48E-02	5.49E-03	7.31E-03	-6.45E-03	1.28E-02	6.15E-01	-6.33E-02	2.98E-02	3.42E-02
1-myristoyl-GPC (14:0)	3.75E-04	5.79E-03	9.48E-01	-9.52E-03	1.33E-02	4.75E-01	-2.92E-02	3.11E-02	3.49E-01

Supplementary Table 2: Parameter estimates for metabolome-wide association studies for diet-metabolite associations for each of: The Healthy Eating Index-2015 (HEI-15), DASH and AMED diet

1-arachidoyl-GPC (20:0)	3.57E-02	5.62E-03	8.63E-10	5.98E-02	1.34E-02	1.11E-05	1.80E-01	3.06E-02	1.04E-08
metoprolol acid metabolite*	1.11E-03	2.71E-03	6.82E-01	9.60E-03	6.21E-03	1.23E-01	1.43E-02	1.46E-02	3.27E-01
heme	9.00E-04	5.93E-03	8.80E-01	3.57E-04	1.37E-02	9.79E-01	5.34E-03	3.20E-02	8.68E-01
stearoylcarnitine (C18)	-1.01E-02	5.76E-03	8.05E-02	-1.63E-02	1.33E-02	2.21E-01	-2.57E-02	3.12E-02	4.10E-01
laurylcarnitine (C12)	-1.48E-02	5.88E-03	1.27E-02	-4.02E-02	1.35E-02	3.13E-03	-4.59E-02	3.19E-02	1.52E-01
isovalerylcarnitine (C5)	-1.17E-02	5.99E-03	5.24E-02	-1.27E-02	1.39E-02	3.59E-01	-3.57E-02	3.24E-02	2.72E-01
1-linoleoyl-GPC (18:2)	1.13E-02	5.68E-03	4.75E-02	2.32E-02	1.31E-02	7.76E-02	7.45E-02	3.05E-02	1.52E-02
7-methylxanthine	-5.11E-03	5.67E-03	3.68E-01	1.85E-02	1.30E-02	1.55E-01	-2.16E-02	3.06E-02	4.79E-01
1,3,7-trimethylurate	-1.51E-02	5.69E-03	8.32E-03	-2.93E-02	1.31E-02	2.67E-02	-6.64E-02	3.08E-02	3.18E-02
3,7-dimethylurate	-6.18E-03	5.59E-03	2.70E-01	8.81E-03	1.29E-02	4.94E-01	-1.99E-02	3.01E-02	5.10E-01
1,7-dimethylurate	-1.67E-02	5.81E-03	4.24E-03	-2.72E-02	1.35E-02	4.46E-02	-6.83E-02	3.15E-02	3.10E-02
1-methylurate	-7.67E-03	5.87E-03	1.93E-01	-1.15E-02	1.35E-02	3.96E-01	-3.01E-02	3.17E-02	3.43E-01
5-acetylamino-6-formylamino-3-methyluracil	-8.36E-03	5.75E-03	1.47E-01	-1.94E-02	1.32E-02	1.44E-01	-6.15E-02	3.09E-02	4.74E-02
5-acetylamino-6-amino-3-methyluracil	-1.09E-02	5.80E-03	6.15E-02	-1.27E-02	1.34E-02	3.46E-01	-5.51E-02	3.13E-02	7.91E-02
indolebutyrate	-7.36E-04	5.41E-03	8.92E-01	2.91E-02	1.23E-02	1.90E-02	-3.38E-02	2.91E-02	2.47E-01
1-methylxanthine	-1.20E-02	5.64E-03	3.49E-02	-2.06E-02	1.30E-02	1.15E-01	-4.03E-02	3.05E-02	1.88E-01
N1-methylinosine	-1.02E-02	6.03E-03	9.17E-02	6.28E-03	1.39E-02	6.53E-01	-7.72E-02	3.23E-02	1.75E-02
N2,N2-dimethylguanosine	-1.33E-02	5.85E-03	2.41E-02	-5.91E-03	1.36E-02	6.64E-01	-9.57E-02	3.13E-02	2.47E-03
N4-acetylcytidine	-1.50E-02	5.76E-03	9.65E-03	-1.23E-02	1.34E-02	3.61E-01	-6.93E-02	3.11E-02	2.69E-02
N6-carbamoylthreonyladenosine	-1.13E-02	5.67E-03	4.70E-02	-1.40E-03	1.31E-02	9.15E-01	-8.70E-02	3.03E-02	4.44E-03
orotidine	-1.57E-02	5.57E-03	5.12E-03	-1.26E-02	1.30E-02	3.34E-01	-8.10E-02	3.01E-02	7.47E-03
phenylacetylglutamine	2.85E-03	6.09E-03	6.40E-01	2.95E-02	1.39E-02	3.48E-02	-1.20E-03	3.28E-02	9.71E-01
4-hydroxyhippurate	-3.70E-03	6.07E-03	5.43E-01	1.55E-02	1.40E-02	2.66E-01	1.32E-02	3.27E-02	6.87E-01
5,6-dihydrouridine	-9.70E-03	5.81E-03	9.63E-02	-2.54E-03	1.34E-02	8.50E-01	-5.40E-02	3.13E-02	8.54E-02
3-(3-amino-3-carboxypropyl)uridine*	-9.19E-03	5.87E-03	1.19E-01	-1.12E-02	1.35E-02	4.07E-01	-8.03E-02	3.14E-02	1.10E-02
1-arachidonylglycerol (20:4)	-5.01E-03	5.41E-03	3.55E-01	-2.24E-02	1.24E-02	7.25E-02	-1.76E-02	2.92E-02	5.46E-01
1-linolenoylglycerol (18:3)	1.05E-02	5.42E-03	5.27E-02	1.36E-02	1.25E-02	2.77E-01	3.77E-02	2.93E-02	1.99E-01
cysteine-glutathione disulfide	-3.33E-03	5.25E-03	5.27E-01	-2.43E-03	1.21E-02	8.41E-01	-2.94E-02	2.83E-02	3.00E-01
5-methyluridine (ribothymidine)	-6.14E-03	6.09E-03	3.14E-01	1.43E-04	1.41E-02	9.92E-01	-3.50E-02	3.28E-02	2.88E-01
isovalerylglycine	7.85E-03	5.72E-03	1.71E-01	3.60E-02	1.30E-02	6.12E-03	4.32E-02	3.08E-02	1.62E-01
3-hydroxydodecanedioate*	-3.21E-03	5.57E-03	5.64E-01	-3.67E-03	1.28E-02	7.75E-01	-3.47E-03	3.00E-02	9.08E-01
7-methylguanine	-1.14E-02	5.97E-03	5.68E-02	-1.44E-02	1.38E-02	2.97E-01	-6.27E-02	3.22E-02	5.25E-02
1-stearoyl-GPE (18:0)	9.72E-03	5.94E-03	1.03E-01	5.16E-03	1.37E-02	7.07E-01	5.94E-02	3.20E-02	6.43E-02
1-stearoyl-GPG (18:0)	-7.35E-03	5.82E-03	2.08E-01	-1.41E-02	1.34E-02	2.93E-01	-6.99E-02	3.12E-02	2.58E-02
N1-Methyl-2-pyridone-5-carboxamide	-2.27E-03	6.00E-03	7.05E-01	2.02E-02	1.38E-02	1.44E-01	-3.39E-02	3.23E-02	2.95E-01
mead acid (20:3n9)	-8.22E-03	6.02E-03	1.73E-01	-2.86E-02	1.38E-02	3.92E-02	-5.77E-02	3.24E-02	7.58E-02
gamma-glutamylisoleucine*	-9.67E-03	5.58E-03	8.38E-02	1.71E-03	1.29E-02	8.95E-01	-4.33E-02	3.01E-02	1.51E-01
oleoylcarnitine (C18:1)	-3.21E-03	5.69E-03	5.73E-01	-2.32E-02	1.30E-02	7.62E-02	-8.69E-03	3.07E-02	7.77E-01
2-methylbutyrylcarnitine (C5)	6.67E-04	6.05E-03	9.12E-01	1.38E-02	1.39E-02	3.21E-01	1.11E-03	3.26E-02	9.73E-01
phenol sulfate	-2.27E-03	5.90E-03	7.01E-01	4.58E-03	1.36E-02	7.36E-01	1.55E-02	3.18E-02	6.27E-01
1-palmitoleoyl-GPC (16:1)*	-2.22E-04	5.72E-03	9.69E-01	-2.31E-02	1.31E-02	7.84E-02	-1.97E-02	3.08E-02	5.22E-01
hexanoylglycine	-2.96E-03	5.57E-03	5.96E-01	-2.04E-02	1.28E-02	1.11E-01	-2.06E-02	3.00E-02	4.92E-01
glutamine_degradant*	2.17E-03	4.96E-03	6.62E-01	1.92E-02	1.14E-02	9.33E-02	-7.42E-05	2.68E-02	9.98E-01
2-hydroxy-3-methylvalerate	1.54E-03	5.14E-03	7.65E-01	-2.21E-02	1.18E-02	6.16E-02	6.29E-02	2.75E-02	2.28E-02
homostachydrine*	5.28E-03	5.88E-03	3.70E-01	1.17E-02	1.35E-02	3.88E-01	-1.64E-02	3.17E-02	6.05E-01
1-arachidonoyl-GPC (20:4n6)*	-2.38E-03	6.03E-03	6.94E-01	-1.58E-02	1.39E-02	2.57E-01	1.25E-04	3.25E-02	9.97E-01
1-dihomo-linolenoyl-GPC (20:3n3 or 6)*	9.45E-03	5.71E-03	9.89E-02	1.22E-02	1.32E-02	3.54E-01	5.53E-04	3.09E-02	9.86E-01
1-dihomo-linoleoyl-GPC (20:2)*	2.12E-02	5.67E-03	2.31E-04	2.83E-02	1.33E-02	3.38E-02	9.33E-02	3.08E-02	2.70E-03
2-arachidonoyl-GPC (20:4)*	-2.23E-03	6.12E-03	7.16E-01	-2.01E-02	1.40E-02	1.53E-01	-7.61E-03	3.30E-02	8.18E-01
2-oleoyl-GPC (18:1)*	1.07E-02	5.76E-03	6.40E-02	5.34E-03	1.33E-02	6.90E-01	5.53E-02	3.11E-02	7.62E-02
2-linoleoyl-GPC (18:2)*	4.63E-03	5.85E-03	4.30E-01	1.52E-02	1.35E-02	2.59E-01	3.67E-02	3.15E-02	2.45E-01
2-palmitoleoyl-GPC (16:1)*	-4.24E-03	5.60E-03	4.50E-01	-2.53E-02	1.28E-02	4.99E-02	-3.03E-02	3.02E-02	3.17E-01
2-palmitoyl-GPC (16:0)*	2.73E-03	5.92E-03	6.46E-01	-1.73E-02	1.36E-02	2.04E-01	-8.61E-03	3.19E-02	7.88E-01
2-myristoyl-GPC (14:0)*	3.18E-03	5.82E-03	5.85E-01	-6.21E-03	1.34E-02	6.44E-01	-1.98E-02	3.14E-02	5.28E-01
1-docosahexaenoyl-GPC (22:6)*	1.55E-02	5.84E-03	8.30E-03	7.25E-03	1.36E-02	5.94E-01	1.13E-01	3.11E-02	3.50E-04
1-palmitoyl-GPE (16:0)	6.21E-03	5.98E-03	3.00E-01	-1.15E-02	1.38E-02	4.03E-01	3.21E-02	3.22E-02	3.19E-01
1-oleoyl-GPE (18:1)	1.22E-02	5.67E-03	3.24E-02	2.71E-02	1.31E-02	3.86E-02	7.08E-02	3.05E-02	2.11E-02
1-linoleoyl-GPE (18:2)*	1.12E-03	5.74E-03	8.45E-01	9.63E-03	1.32E-02	4.66E-01	1.25E-02	3.09E-02	6.87E-01
1-arachidonoyl-GPE (20:4n6)*	-1.03E-02	5.91E-03	8.18E-02	-3.15E-02	1.35E-02	2.09E-02	-3.38E-02	3.20E-02	2.91E-01
N-acetylcitrulline	-1.13E-02	5.32E-03	3.52E-02	-4.45E-03	1.23E-02	7.19E-01	-3.58E-02	2.88E-02	2.15E-01
2-hydroxypalmitate	-9.35E-03	5.83E-03	1.10E-01	-2.46E-02	1.34E-02	6.70E-02	-5.30E-02	3.14E-02	9.24E-02
docosapentaenoate (n6 DPA; 22:5n6)	-1.85E-02	6.02E-03	2.35E-03	-5.36E-02	1.37E-02	1.17E-04	-1.06E-01	3.24E-02	1.20E-03
gulonate*	3.77E-03	5.81E-03	5.17E-01	1.70E-02	1.33E-02	2.05E-01	-1.74E-02	3.13E-02	5.79E-01
isobutyrylglycine	8.00E-03	5.75E-03	1.65E-01	2.73E-02	1.32E-02	3.91E-02	3.14E-02	3.10E-02	3.13E-01
glutarylcarnitine (C5-DC)	-9.14E-03	5.87E-03	1.21E-01	-2.36E-02	1.35E-02	8.13E-02	-6.58E-04	3.18E-02	9.83E-01
2-methylmalonylcarnitine (C4-DC)	-1.23E-03	5.35E-03	8.18E-01	7.13E-03	1.23E-02	5.63E-01	-3.14E-02	2.88E-02	2.76E-01
tiglylcarnitine (C5:1-DC)	3.43E-03	5.69E-03	5.47E-01	2.48E-02	1.30E-02	5.79E-02	-1.47E-02	3.06E-02	6.32E-01
hydroquinone sulfate	6.62E-03	6.16E-03	2.83E-01	3.21E-02	1.41E-02	2.33E-02	4.71E-02	3.32E-02	1.56E-01
catechol sulfate	3.18E-02	5.20E-03	3.24E-09	6.33E-02	1.22E-02	3.73E-07	1.26E-01	2.89E-02	1.88E-05
cholesterol sulfate	6.48E-03	5.76E-03	2.62E-01	-7.79E-03	1.33E-02	5.58E-01	5.14E-02	3.10E-02	9.81E-02
7-alpha-hydroxy-3-oxo-4-cholestenoate (7-Hoca)	-1.32E-02	5.92E-03	2.65E-02	-1.92E-02	1.37E-02	1.63E-01	-4.33E-02	3.21E-02	1.78E-01
tetradecanedioate (C14-DC)	-9.75E-03	5.52E-03	7.84E-02	-3.10E-02	1.26E-02	1.49E-02	-5.74E-02	2.97E-02	5.43E-02
hexadecanedioate (C16-DC)	-1.29E-02	5.60E-03	2.20E-02	-3.13E-02	1.29E-02	1.55E-02	-5.38E-02	3.03E-02	7.69E-02
octadecanedioate (C18-DC)	-6.94E-03	5.86E-03	2.37E-01	-5.47E-03	1.35E-02	6.86E-01	-1.93E-02	3.17E-02	5.43E-01
undecanedioate (C11-DC)	2.31E-04	6.13E-03	9.70E-01	7.61E-04	1.41E-02	9.57E-01	3.63E-03	3.30E-02	9.13E-01
glycerophosphoethanolamine	5.52E-03	5.93E-03	3.53E-01	3.84E-03	1.37E-02	7.79E-01	4.74E-02	3.19E-02	1.39E-01
3-(3-hydroxyphenyl)propionate	8.43E-03	5.77E-03	1.45E-01	2.81E-02	1.32E-02	3.48E-02	6.39E-02	3.10E-02	4.00E-02
ectoine	1.74E-03	5.95E-03	7.71E-01	1.32E-02	1.37E-02	3.35E-01	-1.76E-02	3.21E-02	5.83E-01
2-oleoyl-GPE (18:1)*	9.69E-03	5.78E-03	9.46E-02	2.63E-02	1.33E-02	4.80E-02	5.25E-02	3.11E-02	9.28E-02
2-palmitoyl-GPE (16:0)*	2.28E-03	6.00E-03	7.04E-01	-2.19E-02	1.37E-02	1.12E-01	1.41E-02	3.23E-02	6.63E-01
1-arachidonoyl-GPI (20:4)*	-1.23E-02	6.04E-03	4.30E-02	-5.27E-02	1.36E-02	1.39E-04	-5.57E-02	3.26E-02	8.88E-02
1-palmitoyl-GPI (16:0)	1.16E-03	5.95E-03	8.46E-01	-9.76E-03	1.37E-02	4.76E-01	-1.68E-02	3.21E-02	6.01E-01
glycolithocholate sulfate*	8.35E-03	5.86E-03	1.55E-01	1.40E-02	1.35E-02	3.02E-01	-2.72E-02	3.16E-02	3.91E-01
taurolithocholate 3-sulfate	7.82E-03	5.99E-03	1.92E-01	1.36E-02	1.38E-02	3.24E-01	-4.71E-02	3.22E-02	1.45E-01

Supplementary Table 2: Parameter estimates for metabolome-wide association studies for diet-metabolite associations for each of: The Healthy Eating Index-2015 (HEI-15), DASH and AMED diet

deoxycarnitine	-1.04E-02	5.74E-03	7.07E-02	3.62E-03	1.33E-02	7.85E-01	-3.46E-02	3.10E-02	2.65E-01
N6-succinyladenosine	-2.08E-03	5.53E-03	7.07E-01	-4.15E-03	1.27E-02	7.45E-01	-2.34E-02	2.98E-02	4.33E-01
1-ribosyl-imidazoleacetate*	6.01E-03	5.59E-03	2.83E-01	2.36E-02	1.28E-02	6.70E-02	7.11E-03	3.02E-02	8.14E-01
2-arachidonoyl-GPE (20:4)*	-1.45E-02	5.95E-03	1.56E-02	-2.42E-02	1.38E-02	8.04E-02	-4.41E-02	3.23E-02	1.74E-01
leucylleucine	1.38E-03	5.83E-03	8.12E-01	5.91E-03	1.34E-02	6.60E-01	-4.21E-02	3.13E-02	1.80E-01
N2-acetyllysine	-8.10E-03	4.09E-03	4.87E-02	-1.47E-02	9.44E-03	1.20E-01	-9.04E-03	2.22E-02	6.84E-01
alpha-hydroxycaproate	-1.18E-03	4.91E-03	8.10E-01	-1.56E-02	1.13E-02	1.68E-01	-2.46E-02	2.64E-02	3.52E-01
3,4-dihydroxybutyrate	-9.63E-03	5.67E-03	9.08E-02	-1.73E-02	1.31E-02	1.87E-01	-5.91E-02	3.05E-02	5.39E-02
indoleacetylglutamine	-8.85E-03	5.34E-03	9.87E-02	-3.26E-02	1.22E-02	8.02E-03	-2.08E-02	2.89E-02	4.72E-01
hexanoylglutamine	-1.10E-02	5.77E-03	5.75E-02	-2.95E-02	1.33E-02	2.69E-02	-6.54E-02	3.11E-02	3.62E-02
N6-acetyllysine	4.19E-03	5.73E-03	4.65E-01	2.16E-02	1.31E-02	1.01E-01	-3.01E-04	3.09E-02	9.92E-01
dihomo-linolenate (20:3n3 or n6)	-1.24E-02	5.91E-03	3.74E-02	-3.53E-02	1.36E-02	9.62E-03	-6.97E-02	3.18E-02	2.95E-02
mannitol/sorbitol	3.40E-03	5.94E-03	5.67E-01	1.87E-02	1.36E-02	1.72E-01	3.89E-02	3.20E-02	2.25E-01
tryptophan betaine	2.19E-02	5.41E-03	6.66E-05	4.99E-02	1.25E-02	7.87E-05	1.06E-01	2.93E-02	3.52E-04
4-vinylphenol sulfate	1.25E-02	6.00E-03	3.75E-02	4.36E-02	1.37E-02	1.62E-03	3.22E-02	3.25E-02	3.23E-01
4-ethylphenylsulfate	1.15E-02	6.13E-03	6.26E-02	3.82E-02	1.40E-02	6.86E-03	1.24E-02	3.32E-02	7.10E-01
thymol sulfate	8.65E-03	6.00E-03	1.50E-01	-8.22E-03	1.38E-02	5.53E-01	2.72E-02	3.24E-02	4.02E-01
3-methyladipate	1.04E-02	5.18E-03	4.52E-02	2.73E-02	1.19E-02	2.27E-02	6.03E-02	2.79E-02	3.14E-02
pyrraline	1.77E-02	5.84E-03	2.67E-03	5.13E-02	1.33E-02	1.45E-04	8.39E-02	3.16E-02	8.39E-03
2-linoleoyl-GPE (18:2)*	-3.02E-03	5.79E-03	6.03E-01	4.76E-03	1.33E-02	7.21E-01	-3.00E-03	3.12E-02	9.23E-01
1-oleoyl-GPI (18:1)	1.27E-02	6.08E-03	3.78E-02	4.38E-03	1.41E-02	7.56E-01	6.30E-02	3.28E-02	5.60E-02
1-linoleoyl-GPI (18:2)*	-4.88E-03	5.88E-03	4.08E-01	-3.42E-02	1.34E-02	1.13E-02	-7.44E-03	3.17E-02	8.15E-01
1-palmitoleoyl-GPE (16:1)*	-1.26E-02	5.45E-03	2.18E-02	-4.12E-02	1.24E-02	1.04E-03	-7.09E-02	2.93E-02	1.62E-02
1-palmitoleoyl-GPI (16:1)*	-4.60E-03	5.15E-03	3.72E-01	-4.27E-02	1.16E-02	2.75E-04	-5.52E-02	2.76E-02	4.66E-02
desmethylnaproxen	1.42E-03	3.09E-03	6.47E-01	9.43E-03	7.09E-03	1.85E-01	1.94E-03	1.67E-02	9.08E-01
desmethylnaproxen sulfate	2.10E-03	3.57E-03	5.57E-01	1.34E-02	8.18E-03	1.02E-01	1.07E-02	1.92E-02	5.79E-01
2-hydroxyacetaminophen sulfate*	7.48E-03	4.51E-03	9.83E-02	2.48E-02	1.03E-02	1.69E-02	2.97E-02	2.43E-02	2.23E-01
2-methoxyacetaminophen sulfate*	4.89E-03	3.93E-03	2.15E-01	1.85E-02	9.01E-03	4.10E-02	2.70E-02	2.12E-02	2.03E-01
2-methoxyacetaminophen glucuronide*	4.12E-03	3.67E-03	2.63E-01	1.69E-02	8.41E-03	4.55E-02	1.86E-02	1.98E-02	3.49E-01
3-(cystein-S-yl)acetaminophen*	5.25E-03	3.03E-03	8.40E-02	1.43E-02	6.95E-03	4.08E-02	1.24E-02	1.64E-02	4.49E-01
o-cresol sulfate	3.71E-03	5.33E-03	4.87E-01	-1.54E-02	1.22E-02	2.08E-01	2.37E-02	2.87E-02	4.09E-01
dimethylarginine (SDMA + ADMA)	-3.60E-03	5.81E-03	5.36E-01	9.75E-04	1.34E-02	9.42E-01	-4.16E-02	3.12E-02	1.84E-01
gamma-glutamylalanine	-1.26E-02	5.67E-03	2.70E-02	-1.25E-02	1.31E-02	3.41E-01	-3.72E-02	3.07E-02	2.26E-01
N-acetylserine	-1.32E-03	6.09E-03	8.29E-01	2.78E-02	1.39E-02	4.72E-02	-1.10E-02	3.28E-02	7.38E-01
1-stearoyl-2-oleoyl-GPE (18:0/18:1)	1.19E-02	5.99E-03	4.84E-02	1.39E-02	1.38E-02	3.15E-01	8.09E-02	3.21E-02	1.24E-02
chiro-inositol	1.47E-02	4.63E-03	1.67E-03	3.46E-02	1.07E-02	1.32E-03	6.75E-02	2.51E-02	7.60E-03
4-allylphenol sulfate	1.68E-02	6.24E-03	7.42E-03	3.34E-02	1.44E-02	2.14E-02	5.93E-02	3.39E-02	8.13E-02
1-stearoyl-2-arachidonoyl-GPC (18:0/20:4)	-8.65E-03	5.87E-03	1.41E-01	-3.05E-02	1.34E-02	2.40E-02	-2.52E-02	3.17E-02	4.27E-01
1-palmitoyl-2-linoleoyl-GPE (16:0/18:2)	4.73E-03	6.02E-03	4.33E-01	-2.84E-03	1.39E-02	8.38E-01	1.93E-02	3.25E-02	5.52E-01
1-stearoyl-2-arachidonoyl-GPS (18:0/20:4)	-2.17E-03	5.88E-03	7.12E-01	4.32E-03	1.35E-02	7.50E-01	-2.98E-03	3.17E-02	9.25E-01
sphinganine-1-phosphate	-4.89E-03	6.13E-03	4.25E-01	-2.59E-04	1.41E-02	9.85E-01	-1.97E-03	3.31E-02	9.53E-01
glycosyl-N-nervonoyl-sphingosine (d18:1/24:1)*	2.11E-04	5.82E-03	9.71E-01	-1.30E-02	1.34E-02	3.30E-01	3.80E-02	3.13E-02	2.25E-01
glycosyl-N-stearoyl-sphingosine (d18:1/18:0)	-1.28E-02	5.76E-03	2.68E-02	-3.91E-02	1.32E-02	3.25E-03	-4.31E-02	3.12E-02	1.69E-01
cyclo(leu-pro)	-1.06E-02	5.61E-03	6.09E-02	-3.56E-02	1.28E-02	5.87E-03	-2.85E-02	3.04E-02	3.50E-01
succinylcarnitine (C4-DC)	-3.96E-03	5.91E-03	5.03E-01	2.11E-02	1.36E-02	1.21E-01	-2.60E-02	3.18E-02	4.15E-01
bilirubin (E,E)*	-4.50E-03	5.61E-03	4.24E-01	-9.58E-03	1.29E-02	4.59E-01	2.21E-03	3.03E-02	9.42E-01
bilirubin (E,Z or Z,E)*	2.79E-04	5.60E-03	9.60E-01	-4.21E-03	1.29E-02	7.44E-01	1.43E-02	3.02E-02	6.35E-01
N-methylproline	2.41E-02	6.02E-03	7.86E-05	6.36E-02	1.37E-02	5.55E-06	9.89E-02	3.28E-02	2.82E-03
beta-cryptoxanthin	3.30E-02	5.51E-03	6.26E-09	5.92E-02	1.30E-02	7.86E-06	1.48E-01	3.03E-02	1.69E-06
5alpha-androstan-3beta,17beta-diol disulfate	-1.47E-02	4.88E-03	2.92E-03	-4.16E-02	1.11E-02	2.26E-04	-4.41E-02	2.66E-02	9.90E-02
5alpha-pregnan-3beta,20alpha-diol disulfate	-9.45E-03	6.13E-03	1.24E-01	-1.82E-02	1.41E-02	1.98E-01	-7.66E-02	3.29E-02	2.05E-02
glycocholenate sulfate*	-1.25E-02	5.53E-03	2.45E-02	-2.02E-02	1.28E-02	1.15E-01	-6.58E-02	2.98E-02	2.80E-02
taurocholenate sulfate*	-2.61E-03	5.97E-03	6.62E-01	-2.03E-02	1.37E-02	1.40E-01	-4.99E-02	3.21E-02	1.20E-01
androstenediol (3beta,17beta) disulfate (1)	-1.51E-02	5.26E-03	4.31E-03	-5.48E-02	1.18E-02	5.49E-06	-5.89E-02	2.85E-02	3.98E-02
pregnenediol disulfate (C21H34O8S2)*	-1.20E-02	5.39E-03	2.66E-02	-3.42E-02	1.23E-02	5.93E-03	-6.69E-02	2.90E-02	2.20E-02
androstenediol (3beta,17beta) disulfate (2)	-1.96E-02	5.11E-03	1.56E-04	-3.43E-02	1.19E-02	4.21E-03	-9.64E-02	2.76E-02	5.71E-04
21-hydroxypregnenolone disulfate	-1.07E-02	5.72E-03	6.34E-02	-2.27E-02	1.32E-02	8.55E-02	-6.08E-02	3.08E-02	4.94E-02
5alpha-androstan-3alpha,17alpha-diol monosulfate	-8.17E-03	4.88E-03	9.52E-02	7.90E-04	1.13E-02	9.44E-01	-4.44E-02	2.63E-02	9.24E-02
5alpha-pregnan-3beta,20beta-diol monosulfate (1)	-1.03E-02	5.85E-03	7.86E-02	-1.73E-02	1.35E-02	2.01E-01	-8.26E-02	3.13E-02	8.83E-03
5alpha-pregnan-3beta,20alpha-diol monosulfate (2)	-5.47E-03	5.93E-03	3.57E-01	-2.97E-03	1.37E-02	8.28E-01	-6.22E-02	3.18E-02	5.14E-02
5alpha-pregnan-diol disulfate	-1.35E-02	4.88E-03	6.10E-03	-2.41E-02	1.13E-02	3.35E-02	-6.76E-02	2.64E-02	1.09E-02
5alpha-androstan-3alpha,17beta-diol disulfate	-5.67E-03	4.48E-03	2.07E-01	-9.11E-03	1.03E-02	3.78E-01	-4.39E-02	2.41E-02	6.94E-02
5alpha-androstan-3alpha,17beta-diol monosulfate (1)	-1.43E-02	5.64E-03	1.16E-02	-3.31E-02	1.30E-02	1.12E-02	-5.00E-02	3.06E-02	1.03E-01
5alpha-androstan-3beta,17alpha-diol disulfate	-7.08E-03	5.41E-03	1.92E-01	-7.64E-03	1.25E-02	5.41E-01	-2.08E-02	2.92E-02	4.77E-01
5alpha-androstan-3beta,17beta-diol monosulfate (2)	-1.16E-02	5.28E-03	2.94E-02	-3.12E-02	1.21E-02	1.06E-02	-3.48E-02	2.86E-02	2.25E-01
androstenediol (3alpha, 17alpha) monosulfate (2)	-1.36E-02	5.06E-03	7.55E-03	-4.36E-03	1.18E-02	7.12E-01	-7.47E-02	2.73E-02	6.52E-03
androstenediol (3alpha, 17alpha) monosulfate (3)	-1.68E-02	5.00E-03	8.94E-04	-3.26E-02	1.16E-02	5.21E-03	-8.33E-02	2.71E-02	2.27E-03
androstenediol (3beta,17beta) monosulfate (1)	-1.25E-02	5.27E-03	1.86E-02	-3.43E-02	1.21E-02	4.82E-03	-5.85E-02	2.85E-02	4.06E-02
androstenediol (3beta,17beta) monosulfate (2)	-1.22E-02	5.46E-03	2.60E-02	-2.86E-02	1.26E-02	2.35E-02	-2.82E-02	2.96E-02	3.42E-01
5alpha-pregnan-3beta-ol,20-one sulfate	4.06E-04	4.27E-03	9.24E-01	-2.92E-03	9.82E-03	7.66E-01	-8.53E-03	2.30E-02	7.11E-01
4-hydroxycoumarin	1.20E-02	5.64E-03	3.42E-02	1.77E-02	1.30E-02	1.76E-01	2.11E-02	3.06E-02	4.92E-01
1-docosahexaenoyl-GPE (22:6)*	3.96E-03	5.67E-03	4.85E-01	-2.54E-02	1.30E-02	5.10E-02	5.99E-02	3.04E-02	4.93E-02
2-docosahexaenoyl-GPE (22:6)*	1.43E-03	5.73E-03	8.03E-01	-1.45E-02	1.32E-02	2.71E-01	1.30E-02	3.09E-02	6.74E-01
1-docosapentaenoyl-GPC (22:5n3)*	-6.58E-04	6.01E-03	9.13E-01	5.27E-03	1.38E-02	7.04E-01	2.24E-02	3.24E-02	4.90E-01
1-docosapentaenoyl-GPE (22:5n6)*	-2.13E-02	5.69E-03	2.22E-04	-4.79E-02	1.31E-02	3.16E-04	-1.06E-01	3.08E-02	6.95E-04
pregnenediol sulfate (C21H34O5S)*	-6.72E-03	5.58E-03	2.29E-01	-1.77E-02	1.28E-02	1.70E-01	-5.26E-02	3.00E-02	8.02E-02
2-hydroxyglutarate	-7.91E-03	6.14E-03	1.99E-01	-3.33E-02	1.40E-02	1.82E-02	4.59E-04	3.32E-02	9.89E-01
gamma-CEHC	-9.83E-03	5.87E-03	9.51E-02	9.26E-03	1.36E-02	4.96E-01	-4.31E-02	3.17E-02	1.75E-01
N-acetyl-beta-alanine	1.01E-02	5.46E-03	6.60E-02	1.58E-02	1.26E-02	2.10E-01	5.94E-02	2.94E-02	4.42E-02
5-methylthioribose**	9.92E-04	5.65E-03	8.61E-01	2.53E-02	1.29E-02	5.11E-02	5.08E-03	3.04E-02	8.68E-01
sphingomyelin (d18:1/18:1, d18:2/18:0)	-2.26E-02	5.28E-03	2.60E-05	-5.08E-02	1.22E-02	4.03E-05	-7.26E-02	2.91E-02	1.30E-02
palmitoyl sphingomyelin (d18:1/16:0)	-7.35E-04	5.89E-03	9.01E-01	-1.05E-02	1.36E-02	4.39E-01	1.20E-02	3.18E-02	7.05E-01
cysteine sulfinic acid	-1.71E-02	5.00E-03	7.52E-04	-4.69E-02	1.14E-02	5.21E-05	-6.33E-02	2.73E-02	2.09E-02
3-hydroxyhippurate	1.13E-02	5.88E-03	5.51E-02	2.87E-02	1.35E-02	3.50E-02	7.19E-02	3.16E-02	2.38E-02

Supplementary Table 2: Parameter estimates for metabolome-wide association studies for diet-metabolite associations for each of: The Healthy Eating Index-2015 (HEI-15), DASH and AMED diet

16a-hydroxy DHEA 3-sulfate	-1.41E-02	5.77E-03	1.51E-02	-1.43E-02	1.34E-02	2.88E-01	-9.58E-02	3.09E-02	2.13E-03
17alpha-hydroxypregnenolone 3-sulfate	-1.01E-02	4.87E-03	3.91E-02	-2.04E-02	1.12E-02	6.95E-02	-8.41E-02	2.59E-02	1.33E-03
pregnenolone sulfate	-8.02E-03	5.68E-03	1.59E-01	-2.33E-02	1.31E-02	7.47E-02	-7.16E-02	3.04E-02	1.92E-02
5-HEPE	1.78E-03	5.27E-03	7.36E-01	-7.69E-03	1.21E-02	5.26E-01	4.15E-02	2.83E-02	1.43E-01
5-HETE	-7.24E-03	5.57E-03	1.95E-01	-2.83E-02	1.27E-02	2.73E-02	-2.89E-02	3.01E-02	3.37E-01
andro steroid monosulfate C19H28O6S (1)*	-1.27E-02	5.47E-03	2.07E-02	-1.87E-02	1.27E-02	1.41E-01	-8.99E-02	2.93E-02	2.35E-03
ergothioneine	1.72E-02	5.65E-03	2.61E-03	4.43E-03	1.32E-02	7.38E-01	8.64E-02	3.05E-02	4.99E-03
1-margaroyl-GPE (17:0)*	7.03E-03	5.77E-03	2.24E-01	2.33E-02	1.32E-02	7.98E-02	-4.39E-03	3.12E-02	8.88E-01
1-pentadecanoyl-GPC (15:0)*	-7.28E-04	5.42E-03	8.93E-01	2.13E-02	1.24E-02	8.81E-02	-5.75E-02	2.90E-02	4.86E-02
S-methylmethionine	1.56E-02	5.08E-03	2.37E-03	4.32E-02	1.16E-02	2.38E-04	1.26E-01	2.68E-02	4.07E-06
indole-3-carboxylate	1.19E-02	5.89E-03	4.45E-02	2.73E-02	1.36E-02	4.49E-02	-1.08E-03	3.20E-02	9.73E-01
13-HODE + 9-HODE	-4.01E-03	5.78E-03	4.88E-01	-9.58E-03	1.33E-02	4.72E-01	-2.11E-03	3.12E-02	9.46E-01
tridecenedioate (C13:1-DC)*	-2.27E-02	5.52E-03	5.11E-05	-2.58E-02	1.30E-02	4.80E-02	-1.38E-01	2.95E-02	4.22E-06
N-acetyl-3-methylhistidine*	-8.79E-03	5.76E-03	1.28E-01	-1.81E-02	1.33E-02	1.75E-01	-4.73E-02	3.11E-02	1.29E-01
4-cholesten-3-one	5.33E-03	5.97E-03	3.73E-01	-1.60E-02	1.37E-02	2.45E-01	1.59E-02	3.22E-02	6.23E-01
cinnamoylglycine	1.80E-02	5.65E-03	1.60E-03	3.94E-02	1.30E-02	2.71E-03	6.37E-02	3.08E-02	3.93E-02
stearoyl ethanolamide	-7.53E-03	5.82E-03	1.97E-01	-2.02E-02	1.34E-02	1.32E-01	-2.88E-02	3.14E-02	3.60E-01
cis-4-decenoylcarnitine (C10:1)	-1.13E-02	5.90E-03	5.74E-02	-2.61E-02	1.36E-02	5.59E-02	-1.97E-02	3.20E-02	5.39E-01
2S,3R-dihydroxybutyrate	-2.64E-03	5.36E-03	6.22E-01	-1.92E-02	1.23E-02	1.20E-01	5.07E-03	2.89E-02	8.61E-01
phenylalanylphenylalanine	1.16E-02	6.05E-03	5.58E-02	3.62E-02	1.38E-02	9.43E-03	3.00E-02	3.28E-02	3.61E-01
(12 or 13)-methylmyristate (a15:0 or i15:0)	-2.12E-02	5.61E-03	1.92E-04	-3.36E-02	1.31E-02	1.06E-02	-1.14E-01	3.02E-02	1.87E-04
(16 or 17)-methylstearate (a19:0 or i19:0)	-1.87E-02	5.79E-03	1.40E-03	-4.75E-02	1.33E-02	4.04E-04	-1.01E-01	3.12E-02	1.39E-03
2R,3R-dihydroxybutyrate	-1.21E-02	6.05E-03	4.71E-02	-1.52E-02	1.40E-02	2.79E-01	-5.83E-02	3.27E-02	7.51E-02
4-methylbenzenesulfonate	-2.58E-03	5.55E-03	6.42E-01	3.79E-03	1.28E-02	7.67E-01	-1.17E-02	2.99E-02	6.96E-01
alpha-ketoglutaramate*	1.15E-02	5.62E-03	4.24E-02	3.67E-02	1.28E-02	4.63E-03	4.68E-02	3.04E-02	1.25E-01
metformin	4.46E-04	1.06E-03	6.73E-01	1.43E-03	2.43E-03	5.57E-01	-3.07E-03	5.69E-03	5.90E-01
ibuprofen acyl glucuronide	2.22E-03	3.25E-03	4.95E-01	-1.03E-02	7.47E-03	1.70E-01	9.95E-04	1.75E-02	9.55E-01
3-hydroxyquinine	-4.87E-04	1.06E-03	6.46E-01	-1.30E-03	2.44E-03	5.95E-01	-1.62E-03	5.71E-03	7.76E-01
2,3-dihydroxyisovalerate	9.67E-03	5.58E-03	8.44E-02	-2.17E-02	1.29E-02	9.25E-02	1.89E-02	3.02E-02	5.33E-01
allopurinol riboside	-2.31E-03	1.04E-03	2.81E-02	-1.19E-04	2.43E-03	9.61E-01	-1.23E-02	5.63E-03	2.92E-02
3-methylglutaconate	1.12E-03	5.51E-03	8.39E-01	2.70E-02	1.26E-02	3.26E-02	-2.34E-02	2.96E-02	4.30E-01
cysteinylglycine disulfide*	-2.16E-02	5.70E-03	1.90E-04	-3.61E-02	1.33E-02	6.98E-03	-1.11E-01	3.08E-02	3.53E-04
isoursodeoxycholate	-1.51E-02	6.04E-03	1.30E-02	-2.80E-02	1.40E-02	4.61E-02	-3.53E-02	3.29E-02	2.84E-01
oxypurinol	-2.31E-03	1.04E-03	2.81E-02	-1.19E-04	2.43E-03	9.61E-01	-1.23E-02	5.63E-03	2.92E-02
formiminoglutamate	-9.68E-03	4.94E-03	5.11E-02	-3.27E-02	1.13E-02	4.08E-03	-3.80E-02	2.67E-02	1.56E-01
hydantoin-5-propionate	4.62E-03	6.04E-03	4.44E-01	3.99E-02	1.37E-02	3.86E-03	-2.04E-02	3.25E-02	5.32E-01
sulfate*	9.22E-03	6.15E-03	1.35E-01	2.51E-02	1.41E-02	7.70E-02	3.21E-02	3.32E-02	3.35E-01
4-hydroxy-2-oxoglutaric acid	1.93E-03	5.82E-03	7.40E-01	1.68E-02	1.34E-02	2.11E-01	3.37E-03	3.14E-02	9.14E-01
4-hydroxyglutamate	-7.47E-04	5.24E-03	8.87E-01	4.39E-03	1.21E-02	7.16E-01	-2.43E-02	2.82E-02	3.89E-01
L-urobilin	-1.67E-03	5.34E-03	7.55E-01	-6.17E-03	1.23E-02	6.16E-01	-5.95E-02	2.86E-02	3.80E-02
pantoate	1.33E-02	5.94E-03	2.65E-02	3.35E-02	1.36E-02	1.46E-02	2.48E-02	3.23E-02	4.43E-01
hydroxycotinine	-3.41E-03	2.89E-03	2.38E-01	-1.59E-02	6.59E-03	1.68E-02	-9.35E-03	1.56E-02	5.49E-01
cotinine N-oxide	-2.71E-03	2.89E-03	3.48E-01	-1.42E-02	6.60E-03	3.25E-02	-5.77E-03	1.56E-02	7.11E-01
omeprazole	5.06E-04	1.05E-03	6.30E-01	1.23E-03	2.41E-03	6.11E-01	2.76E-04	5.65E-03	9.61E-01
atenolol	2.34E-03	1.59E-03	1.42E-01	7.38E-03	3.65E-03	4.39E-02	-1.21E-02	8.57E-03	1.59E-01
diphenhydramine	-3.52E-03	3.46E-03	3.09E-01	4.20E-03	7.96E-03	5.99E-01	-1.14E-02	1.86E-02	5.40E-01
hydrochlorothiazide	1.41E-03	3.54E-03	6.90E-01	-3.03E-04	8.14E-03	9.70E-01	7.24E-03	1.91E-02	7.04E-01
pseudoephedrine	6.60E-20	1.01E-19	5.12E-01	5.38E-19	2.29E-19	1.98E-02	3.95E-19	5.42E-19	4.67E-01
S-methylcysteine	4.79E-03	6.08E-03	4.31E-01	1.67E-02	1.40E-02	2.34E-01	7.23E-02	3.25E-02	2.70E-02
androsterone glucuronide	-9.46E-03	5.11E-03	6.50E-02	4.35E-03	1.18E-02	7.14E-01	-5.02E-02	2.75E-02	6.94E-02
argininate*	1.87E-02	5.85E-03	1.56E-03	4.03E-02	1.35E-02	3.05E-03	9.86E-02	3.16E-02	1.97E-03
solanidine	-1.32E-03	4.79E-03	7.83E-01	-8.17E-03	1.10E-02	4.59E-01	2.67E-02	2.58E-02	3.02E-01
2-oxoarginine*	1.41E-02	5.79E-03	1.54E-02	3.97E-02	1.33E-02	3.01E-03	4.74E-02	3.14E-02	1.32E-01
cis-4-decenoate (10:1n6)*	-1.25E-02	5.96E-03	3.64E-02	-3.90E-02	1.36E-02	4.49E-03	-3.60E-02	3.23E-02	2.65E-01
atorvastatin (lipitor)	5.19E-04	3.19E-03	8.71E-01	-3.71E-03	7.34E-03	6.14E-01	-4.40E-03	1.72E-02	7.98E-01
sertraline	-1.11E-03	2.52E-03	6.59E-01	-7.18E-03	5.78E-03	2.15E-01	-5.86E-03	1.36E-02	6.66E-01
gabapentin	-5.21E-04	2.89E-03	8.57E-01	-2.68E-03	6.65E-03	6.87E-01	-3.85E-03	1.56E-02	8.05E-01
venlafaxine	1.33E-03	1.05E-03	2.09E-01	-2.06E-04	2.43E-03	9.33E-01	9.77E-03	5.67E-03	8.58E-02
quetiapine	6.98E-04	1.03E-03	4.99E-01	-1.28E-03	2.38E-03	5.90E-01	-6.11E-03	5.55E-03	2.72E-01
ethyl glucuronide	8.03E-03	5.58E-03	1.52E-01	-4.28E-02	1.26E-02	8.13E-04	3.49E-02	3.01E-02	2.47E-01
1-behenoyl-GPC (22:0)	3.52E-02	5.64E-03	1.61E-09	6.02E-02	1.34E-02	1.00E-05	1.96E-01	3.03E-02	3.81E-10
1-erucoyl-GPC (22:1)*	2.39E-02	5.36E-03	1.21E-05	2.35E-02	1.27E-02	6.51E-02	1.28E-01	2.89E-02	1.41E-05
1-adrenoyl-GPC (22:4)*	-1.41E-02	5.61E-03	1.27E-02	-2.38E-02	1.30E-02	6.78E-02	-7.69E-02	3.02E-02	1.15E-02
1-lignoceroyl-GPC (24:0)	3.57E-02	5.43E-03	2.39E-10	6.23E-02	1.29E-02	2.27E-06	2.16E-01	2.86E-02	6.89E-13
1-nervonoyl-GPC (24:1n9)*	2.46E-02	5.08E-03	2.14E-06	1.60E-02	1.21E-02	1.89E-01	1.36E-01	2.73E-02	1.00E-06
1-(1-enyl-palmitoyl)-GPC (P-16:0)*	7.68E-03	6.02E-03	2.03E-01	6.48E-04	1.39E-02	9.63E-01	3.43E-02	3.25E-02	2.91E-01
1-(1-enyl-oleoyl)-GPC (P-18:1)*	1.58E-02	5.74E-03	6.16E-03	1.28E-02	1.34E-02	3.38E-01	1.04E-01	3.07E-02	7.92E-04
1-(1-enyl-stearoyl)-GPC (P-18:0)*	3.31E-03	5.40E-03	5.40E-01	3.97E-03	1.24E-02	7.50E-01	1.51E-02	2.91E-02	6.05E-01
1-methyl-5-imidazoleacetate	-9.87E-04	6.00E-03	8.69E-01	5.59E-03	1.38E-02	6.85E-01	-5.10E-02	3.22E-02	1.14E-01
glycoursodeoxycholate	-1.06E-02	6.15E-03	8.53E-02	-2.78E-02	1.41E-02	4.98E-02	-5.78E-02	3.31E-02	8.19E-02
tauroursodeoxycholate	-7.86E-03	4.80E-03	1.03E-01	-2.08E-02	1.10E-02	6.05E-02	-3.79E-02	2.59E-02	1.45E-01
S-methylcysteine sulfoxide	1.81E-02	5.89E-03	2.28E-03	4.18E-02	1.36E-02	2.23E-03	1.12E-01	3.16E-02	4.79E-04
benzoylecgonine	-1.59E-03	2.00E-03	4.29E-01	-2.57E-03	4.62E-03	5.78E-01	4.84E-03	1.08E-02	6.55E-01
(14 or 15)-methylpalmitate (a17:0 or i17:0)	-2.42E-02	5.69E-03	2.82E-05	-4.84E-02	1.32E-02	3.00E-04	-1.37E-01	3.06E-02	1.07E-05
eicosanedioate (C20-DC)	-1.22E-02	6.10E-03	4.57E-02	-1.81E-02	1.41E-02	2.01E-01	-3.79E-02	3.30E-02	2.52E-01
docosadioate (C22-DC)	-8.66E-03	6.23E-03	1.65E-01	-4.53E-03	1.44E-02	7.53E-01	-4.78E-02	3.36E-02	1.55E-01
16-hydroxypalmitate	-1.47E-02	5.91E-03	1.32E-02	-3.31E-02	1.36E-02	1.57E-02	-6.90E-02	3.19E-02	3.16E-02
quinine	-4.87E-04	1.06E-03	6.46E-01	-1.30E-03	2.44E-03	5.95E-01	-1.62E-03	5.71E-03	7.76E-01
oleoyl-linoleoyl-glycerol (18:1/18:2) [1]	6.52E-03	5.80E-03	2.62E-01	1.46E-02	1.33E-02	2.74E-01	1.24E-02	3.13E-02	6.93E-01
oleoyl-linoleoyl-glycerol (18:1/18:2) [2]	9.15E-03	5.83E-03	1.18E-01	1.64E-02	1.35E-02	2.23E-01	3.06E-02	3.15E-02	3.32E-01
1-(1-enyl-palmitoyl)-GPE (P-16:0)*	-1.84E-03	5.99E-03	7.59E-01	-1.00E-02	1.38E-02	4.68E-01	7.96E-03	3.23E-02	8.05E-01
1-(1-enyl-stearoyl)-GPE (P-18:0)*	-1.24E-03	6.03E-03	8.37E-01	-7.75E-03	1.39E-02	5.77E-01	2.80E-02	3.25E-02	3.89E-01
2-stearoyl-GPI (18:0)*	7.04E-03	5.87E-03	2.31E-01	7.57E-03	1.35E-02	5.76E-01	4.83E-02	3.16E-02	1.27E-01
alpha-CEHC glucuronide*	1.15E-02	5.12E-03	2.58E-02	2.04E-02	1.18E-02	8.52E-02	5.02E-03	2.78E-02	8.57E-01

Supplementary Table 2: Parameter estimates for metabolome-wide association studies for diet-metabolite associations for each of: The Healthy Eating Index-2015 (HEI-15), DASH and AMED diet

2-oxindole-3-acetate	6.67E-03	5.58E-03	2.33E-01	1.46E-02	1.28E-02	2.55E-01	3.44E-02	3.01E-02	2.54E-01
N-oleoyltaurine	9.78E-03	5.90E-03	9.82E-02	1.07E-02	1.36E-02	4.34E-01	3.04E-02	3.19E-02	3.42E-01
linoleoylcarnitine (C18:2)*	-7.25E-03	5.93E-03	2.22E-01	-1.52E-02	1.36E-02	2.66E-01	-3.27E-03	3.20E-02	9.19E-01
isoleucylglycine	6.81E-04	6.02E-03	9.10E-01	1.43E-02	1.38E-02	3.01E-01	2.98E-02	3.24E-02	3.59E-01
leucylglycine	-1.76E-03	6.02E-03	7.70E-01	1.44E-02	1.38E-02	2.99E-01	3.12E-02	3.24E-02	3.36E-01
phenylalanylisoleucine	3.72E-03	4.91E-03	4.50E-01	1.81E-02	1.13E-02	1.10E-01	2.55E-02	2.65E-02	3.36E-01
phenylalanylleucine	5.36E-03	6.32E-03	3.97E-01	1.34E-02	1.45E-02	3.59E-01	9.44E-03	3.41E-02	7.82E-01
N-palmitoyltaurine	4.28E-03	5.26E-03	4.16E-01	4.84E-03	1.21E-02	6.90E-01	-7.81E-03	2.84E-02	7.83E-01
N-stearoyltaurine	-5.98E-03	5.95E-03	3.16E-01	-7.95E-03	1.37E-02	5.63E-01	-2.74E-02	3.21E-02	3.94E-01
2-O-methylascorbic acid	6.34E-03	5.74E-03	2.70E-01	1.76E-02	1.32E-02	1.82E-01	-1.61E-02	3.10E-02	6.04E-01
beta-citrylglutamate	-1.16E-02	6.12E-03	5.94E-02	-2.18E-02	1.41E-02	1.25E-01	-1.75E-02	3.32E-02	5.99E-01
rosuvastatin	-7.03E-05	2.16E-03	9.74E-01	3.39E-05	4.98E-03	9.95E-01	7.63E-03	1.17E-02	5.13E-01
trimethylamine N-oxide	-4.41E-04	5.94E-03	9.41E-01	9.72E-04	1.37E-02	9.43E-01	1.12E-02	3.20E-02	7.26E-01
N6-methyllysine	-1.94E-03	5.39E-03	7.19E-01	-1.37E-02	1.24E-02	2.68E-01	2.41E-02	2.90E-02	4.08E-01
dihydroferulate	9.10E-03	5.02E-03	7.06E-02	2.73E-02	1.15E-02	1.82E-02	4.08E-02	2.71E-02	1.33E-01
imidazole propionate	-5.07E-03	5.95E-03	3.95E-01	-1.30E-02	1.37E-02	3.44E-01	-2.11E-03	3.21E-02	9.48E-01
citalopram/escitalopram	-3.93E-03	2.78E-03	1.58E-01	-2.12E-03	6.41E-03	7.42E-01	-4.26E-03	1.50E-02	7.77E-01
duloxetine	6.60E-20	1.01E-19	5.12E-01	5.38E-19	2.29E-19	1.98E-02	3.95E-19	5.42E-19	4.67E-01
milnacipran	6.50E-04	1.06E-03	5.41E-01	4.24E-03	2.43E-03	8.20E-02	-5.30E-03	5.71E-03	3.54E-01
pregnanediol-3-glucuronide	1.24E-03	5.94E-03	8.34E-01	-7.59E-03	1.37E-02	5.79E-01	-3.21E-02	3.19E-02	3.16E-01
(15:2)-anacardic acid	2.10E-03	2.52E-03	4.07E-01	4.57E-03	5.81E-03	4.32E-01	-3.35E-03	1.36E-02	8.06E-01
alliin	2.78E-03	5.03E-03	5.81E-01	-1.18E-02	1.16E-02	3.07E-01	5.31E-02	2.69E-02	4.95E-02
seryltyrosine	7.11E-03	3.87E-03	6.72E-02	1.74E-02	8.90E-03	5.20E-02	2.72E-02	2.09E-02	1.95E-01
histidylalanine	4.44E-03	4.05E-03	2.73E-01	1.05E-02	9.31E-03	2.61E-01	2.16E-02	2.18E-02	3.24E-01
phenylalanylglycine	7.58E-03	5.48E-03	1.68E-01	1.10E-02	1.26E-02	3.85E-01	3.32E-02	2.96E-02	2.63E-01
phenylalanylmethionine	9.53E-03	6.13E-03	1.22E-01	2.92E-02	1.41E-02	3.92E-02	1.16E-02	3.32E-02	7.26E-01
phenylalanyltryptophan	-7.78E-03	5.94E-03	1.91E-01	-3.27E-02	1.36E-02	1.67E-02	-3.11E-02	3.20E-02	3.33E-01
tyrosyllysine	6.12E-03	3.94E-03	1.21E-01	1.97E-02	9.03E-03	3.02E-02	2.80E-02	2.13E-02	1.88E-01
tryptophylasparagine	6.35E-03	3.73E-03	8.93E-02	1.29E-02	8.59E-03	1.35E-01	1.50E-02	2.02E-02	4.58E-01
aspartylaspartate	1.88E-03	5.80E-03	7.46E-01	1.63E-03	1.34E-02	9.03E-01	4.63E-02	3.11E-02	1.38E-01
valylalanine	3.76E-03	5.38E-03	4.86E-01	1.33E-02	1.24E-02	2.85E-01	4.06E-02	2.89E-02	1.61E-01
valylglycine	-1.40E-03	6.04E-03	8.17E-01	1.46E-02	1.39E-02	2.95E-01	1.56E-02	3.26E-02	6.32E-01
valylphenylalanine	9.73E-03	5.53E-03	7.96E-02	3.04E-02	1.27E-02	1.69E-02	5.67E-02	2.98E-02	5.81E-02
methionylalanine	4.69E-03	5.08E-03	3.57E-01	1.41E-02	1.17E-02	2.29E-01	2.46E-02	2.74E-02	3.70E-01
N-palmitoylglycine	-6.73E-03	6.01E-03	2.63E-01	-2.52E-02	1.38E-02	6.80E-02	-5.33E-02	3.23E-02	1.00E-01
mannonate*	-1.09E-02	5.98E-03	7.04E-02	-5.76E-03	1.38E-02	6.77E-01	-7.53E-02	3.21E-02	1.96E-02
norfluoxetine	1.95E-04	2.51E-03	9.38E-01	-4.95E-03	5.78E-03	3.93E-01	7.88E-03	1.35E-02	5.61E-01
2-stearoyl-GPE (18:0)*	-5.52E-03	6.14E-03	3.70E-01	-2.09E-02	1.41E-02	1.40E-01	-2.63E-02	3.31E-02	4.28E-01
(R)-3-hydroxybutyrylcarnitine	-1.09E-02	5.32E-03	4.03E-02	-2.83E-02	1.22E-02	2.12E-02	-4.09E-02	2.88E-02	1.56E-01
N-octanoylglycine	-7.10E-03	5.78E-03	2.21E-01	-7.36E-03	1.33E-02	5.82E-01	-5.28E-02	3.11E-02	9.04E-02
doxylamine	-8.78E-04	1.38E-03	5.24E-01	-3.68E-03	3.17E-03	2.46E-01	-3.95E-03	7.43E-03	5.95E-01
triamterene	4.03E-04	1.06E-03	7.04E-01	-1.12E-04	2.43E-03	9.63E-01	-5.61E-04	5.70E-03	9.22E-01
diltiazem	-3.25E-04	1.88E-03	8.63E-01	3.46E-03	4.32E-03	4.24E-01	3.98E-03	1.01E-02	6.94E-01
fexofenadine	-8.50E-04	2.36E-03	7.19E-01	-6.41E-03	5.42E-03	2.38E-01	-2.45E-02	1.26E-02	5.37E-02
verapamil	1.53E-03	9.45E-04	1.07E-01	1.99E-03	2.18E-03	3.63E-01	4.87E-03	5.11E-03	3.41E-01
N-acetylcarnosine	-3.80E-03	4.77E-03	4.26E-01	1.43E-02	1.10E-02	1.95E-01	-3.23E-02	2.57E-02	2.10E-01
margaroylcarnitine (C17)*	-2.17E-02	5.65E-03	1.46E-04	-4.01E-02	1.31E-02	2.44E-03	-1.06E-01	3.06E-02	5.76E-04
N-methyltaurine	1.15E-02	5.48E-03	3.71E-02	-2.06E-03	1.27E-02	8.72E-01	9.52E-02	2.92E-02	1.25E-03
histidine betaine (hercynine)*	1.48E-02	5.90E-03	1.25E-02	1.15E-03	1.37E-02	9.33E-01	1.17E-01	3.14E-02	2.28E-04
glycohyocholate	7.72E-03	5.82E-03	1.85E-01	1.33E-02	1.34E-02	3.23E-01	2.08E-02	3.14E-02	5.08E-01
2-hydroxydecanoate	1.71E-02	5.97E-03	4.49E-03	3.22E-02	1.38E-02	2.06E-02	4.26E-02	3.25E-02	1.91E-01
3-methyl catechol sulfate (2)	3.78E-03	4.65E-03	4.17E-01	-6.87E-04	1.07E-02	9.49E-01	2.34E-03	2.51E-02	9.26E-01
4-methylcatechol sulfate	1.89E-02	5.91E-03	1.54E-03	4.29E-02	1.36E-02	1.80E-03	5.24E-02	3.23E-02	1.06E-01
3-methyl catechol sulfate (1)	-4.06E-03	5.80E-03	4.85E-01	-1.94E-02	1.33E-02	1.46E-01	-3.30E-02	3.12E-02	2.91E-01
2-hydroxyibuprofen	-7.24E-04	3.91E-03	8.53E-01	-2.24E-02	8.90E-03	1.25E-02	-1.70E-02	2.11E-02	4.20E-01
carboxyibuprofen	-7.77E-03	4.40E-03	7.84E-02	-2.48E-02	1.01E-02	1.43E-02	-4.14E-02	2.37E-02	8.18E-02
O-desmethylvenlafaxine	8.27E-04	1.68E-03	6.24E-01	-1.84E-03	3.88E-03	6.35E-01	1.75E-02	9.02E-03	5.31E-02
zolpidem	-1.35E-03	1.39E-03	3.33E-01	-6.92E-03	3.18E-03	3.06E-02	-3.23E-03	7.51E-03	6.67E-01
warfarin	9.70E-04	2.02E-03	6.32E-01	5.75E-03	4.65E-03	2.17E-01	-2.24E-02	1.08E-02	3.93E-02
3b-hydroxy-5-cholenoic acid	6.73E-03	5.83E-03	2.49E-01	1.95E-02	1.34E-02	1.48E-01	7.76E-03	3.15E-02	8.06E-01
guaiacol sulfate	2.77E-02	5.59E-03	1.27E-06	5.40E-02	1.30E-02	4.53E-05	1.00E-01	3.09E-02	1.33E-03
2-aminooctanoate	1.07E-02	5.82E-03	6.80E-02	2.43E-02	1.34E-02	7.05E-02	4.59E-02	3.14E-02	1.45E-01
furosemide	1.94E-03	1.65E-03	2.41E-01	8.13E-03	3.78E-03	3.22E-02	9.51E-03	8.89E-03	2.86E-01
gamma-CEHC glucuronide*	-1.14E-02	6.16E-03	6.57E-02	1.25E-02	1.43E-02	3.80E-01	-5.76E-02	3.32E-02	8.43E-02
dimethyl sulfone	5.84E-04	6.15E-03	9.24E-01	1.69E-02	1.41E-02	2.33E-01	-2.65E-02	3.31E-02	4.23E-01
N-acetyl-1-methylhistidine*	-1.70E-02	5.13E-03	1.07E-03	-3.79E-02	1.18E-02	1.51E-03	-5.75E-02	2.80E-02	4.07E-02
indolin-2-one	-1.23E-03	5.41E-03	8.20E-01	1.43E-02	1.24E-02	2.52E-01	-1.30E-02	2.92E-02	6.55E-01
2,8-quinolinediol sulfate	-9.09E-04	4.28E-03	8.32E-01	8.12E-03	9.85E-03	4.10E-01	-6.38E-03	2.31E-02	7.82E-01
2-aminophenol sulfate	1.10E-02	5.86E-03	6.13E-02	5.11E-02	1.32E-02	1.37E-04	4.20E-02	3.17E-02	1.85E-01
3-acetylphenol sulfate	4.89E-03	5.54E-03	3.78E-01	1.42E-02	1.27E-02	2.66E-01	4.61E-02	2.98E-02	1.22E-01
sphingomyelin (d18:1/14:0, d16:1/16:0)*	-6.85E-03	5.49E-03	2.13E-01	-1.24E-02	1.27E-02	3.27E-01	-6.61E-02	2.94E-02	2.53E-02
sphingomyelin (d18:2/16:0, d18:1/16:1)*	-1.22E-02	5.59E-03	2.99E-02	-2.55E-02	1.29E-02	4.93E-02	-1.60E-02	3.04E-02	6.00E-01
3-hydroxyadipate	-6.01E-03	5.68E-03	2.90E-01	7.47E-03	1.31E-02	5.69E-01	-1.76E-02	3.06E-02	5.66E-01
3-hydroxycotinine glucuronide	-2.47E-03	2.90E-03	3.95E-01	-1.08E-02	6.65E-03	1.06E-01	-3.09E-03	1.56E-02	8.44E-01
6-oxopiperidine-2-carboxylate	3.72E-03	6.03E-03	5.37E-01	2.37E-02	1.38E-02	8.66E-02	3.81E-02	3.24E-02	2.40E-01
S-allylcysteine	5.84E-04	5.60E-03	9.17E-01	-1.46E-02	1.29E-02	2.56E-01	4.54E-02	3.01E-02	1.33E-01
allopurinol	-1.89E-03	1.41E-03	1.81E-01	4.28E-04	3.26E-03	8.95E-01	-1.37E-02	7.58E-03	7.15E-02
N-delta-acetylornithine	2.89E-02	5.12E-03	4.04E-08	4.56E-02	1.21E-02	2.09E-04	1.35E-01	2.80E-02	2.51E-06
acisoga	8.94E-03	5.94E-03	1.33E-01	1.45E-02	1.37E-02	2.89E-01	8.51E-02	3.17E-02	7.74E-03
2-aminoheptanoate	4.46E-03	5.97E-03	4.56E-01	1.27E-02	1.37E-02	3.58E-01	2.65E-02	3.22E-02	4.11E-01
1-eicosapentaenoyl-GPE (20:5)*	8.84E-03	5.81E-03	1.29E-01	-1.38E-03	1.34E-02	9.18E-01	7.81E-02	3.11E-02	1.25E-02
N-formylanthranilic acid	-3.11E-03	6.06E-03	6.08E-01	-5.12E-03	1.40E-02	7.14E-01	-1.54E-02	3.27E-02	6.38E-01
N2,N5-diacetylornithine	1.87E-02	5.71E-03	1.19E-03	1.05E-03	1.34E-02	9.38E-01	1.08E-01	3.07E-02	5.31E-04
1H-indole-7-acetic acid	7.74E-04	5.96E-03	8.97E-01	8.81E-03	1.37E-02	5.21E-01	-8.05E-02	3.18E-02	1.18E-02

Supplementary Table 2: Parameter estimates for metabolome-wide association studies for diet-metabolite associations for each of: The Healthy Eating Index-2015 (HEI-15), DASH and AMED diet

3-methoxytyramine sulfate	1.20E-02	5.51E-03	3.06E-02	1.18E-02	1.28E-02	3.56E-01	2.62E-02	2.99E-02	3.82E-01
methionine sulfone	3.40E-03	5.94E-03	5.67E-01	1.27E-02	1.37E-02	3.54E-01	-9.69E-03	3.20E-02	7.62E-01
cyclo(ala-pro)	6.89E-04	5.55E-03	9.01E-01	-1.20E-02	1.28E-02	3.47E-01	6.86E-03	2.99E-02	8.19E-01
norbenzoylecgonine*	-1.38E-03	1.59E-03	3.86E-01	-3.20E-03	3.65E-03	3.82E-01	7.46E-04	8.56E-03	9.31E-01
1-linolenoyl-GPC (18:3)*	6.60E-03	5.92E-03	2.66E-01	7.30E-03	1.37E-02	5.93E-01	4.48E-02	3.19E-02	1.61E-01
1-eicosapentaenoyl-GPC (20:5)*	1.18E-02	5.94E-03	4.84E-02	1.39E-03	1.38E-02	9.20E-01	8.46E-02	3.18E-02	8.28E-03
1-eicosenoyl-GPC (20:1)*	3.07E-02	5.70E-03	1.57E-07	4.77E-02	1.35E-02	4.84E-04	1.51E-01	3.10E-02	1.87E-06
1-nonadecanoyl-GPC (19:0)	1.59E-02	5.35E-03	3.19E-03	3.81E-02	1.23E-02	2.15E-03	7.62E-02	2.89E-02	8.91E-03
N-acetylalliin	1.36E-03	5.77E-03	8.14E-01	-1.38E-02	1.33E-02	2.99E-01	4.54E-02	3.10E-02	1.44E-01
1-dihomo-linolenoyl-GPE (20:3n3 or 6)*	4.78E-03	5.97E-03	4.24E-01	6.54E-03	1.38E-02	6.35E-01	-1.17E-02	3.22E-02	7.16E-01
1-(1-enyl-oleoyl)-GPE (P-18:1)*	6.12E-04	6.18E-03	9.21E-01	-1.22E-02	1.42E-02	3.91E-01	3.16E-02	3.32E-02	3.42E-01
fructosyllysine	-8.42E-03	5.98E-03	1.60E-01	-1.89E-02	1.38E-02	1.70E-01	-7.76E-02	3.20E-02	1.60E-02
1-eicosenoyl-GPE (20:1)*	1.60E-02	5.71E-03	5.57E-03	2.72E-02	1.32E-02	4.07E-02	6.48E-02	3.10E-02	3.71E-02
N-methylpipecolate	5.88E-03	5.21E-03	2.60E-01	4.40E-03	1.20E-02	7.15E-01	2.63E-02	2.81E-02	3.50E-01
O-sulfo-L-tyrosine	1.38E-04	5.92E-03	9.81E-01	1.15E-03	1.36E-02	9.33E-01	6.08E-03	3.19E-02	8.49E-01
ferulic acid 4-sulfate	1.22E-02	4.72E-03	1.00E-02	2.91E-02	1.08E-02	7.69E-03	4.65E-02	2.56E-02	7.00E-02
3-(3-hydroxyphenyl)propionate sulfate	1.61E-02	5.82E-03	5.95E-03	3.74E-02	1.34E-02	5.65E-03	6.52E-02	3.16E-02	3.98E-02
11-ketoetiocholanolone glucuronide	6.58E-03	5.74E-03	2.52E-01	6.22E-03	1.32E-02	6.39E-01	-4.11E-02	3.09E-02	1.84E-01
etiocholanolone glucuronide	2.28E-04	5.56E-03	9.67E-01	-4.71E-03	1.28E-02	7.13E-01	-9.72E-03	2.99E-02	7.46E-01
17alpha-hydroxypregnanolone glucuronide	-5.91E-04	5.11E-03	9.08E-01	-1.15E-02	1.18E-02	3.29E-01	-3.68E-02	2.75E-02	1.81E-01
N-acetyltaurine	-2.84E-03	5.47E-03	6.03E-01	7.73E-03	1.26E-02	5.40E-01	2.76E-03	2.95E-02	9.25E-01
1-docosapentaenoyl-GPC (22:5n6)*	-1.13E-02	5.70E-03	4.93E-02	-2.86E-02	1.31E-02	2.96E-02	-9.49E-02	3.04E-02	2.01E-03
1-linolenoyl-GPE (18:3)*	4.81E-04	5.97E-03	9.36E-01	3.50E-03	1.37E-02	7.99E-01	6.08E-03	3.22E-02	8.50E-01
1-oleoyl-GPG (18:1)*	7.21E-03	5.13E-03	1.61E-01	1.78E-02	1.18E-02	1.33E-01	-2.62E-02	2.77E-02	3.44E-01
1-palmitoyl-GPG (16:0)*	-5.01E-03	5.88E-03	3.95E-01	-8.96E-03	1.35E-02	5.08E-01	-3.11E-02	3.17E-02	3.27E-01
N-linoleoyltaurine*	6.30E-03	5.55E-03	2.57E-01	1.68E-02	1.28E-02	1.90E-01	2.31E-02	3.00E-02	4.42E-01
N-acetyl-S-allyl-L-cysteine	-8.13E-04	5.03E-03	8.72E-01	-1.29E-02	1.16E-02	2.64E-01	2.07E-02	2.71E-02	4.46E-01
9-hydroxystearate	-1.92E-02	5.25E-03	3.03E-04	-3.51E-02	1.22E-02	4.22E-03	-8.42E-02	2.85E-02	3.40E-03
3-methylglutarylcarnitine (2)	-1.11E-03	5.46E-03	8.40E-01	2.03E-02	1.25E-02	1.06E-01	-2.81E-02	2.94E-02	3.39E-01
methyl glucopyranoside (alpha + beta)	2.75E-02	5.65E-03	1.86E-06	4.94E-02	1.32E-02	2.28E-04	1.27E-01	3.08E-02	5.15E-05
2-keto-3-deoxy-gluconate	-5.71E-03	5.62E-03	3.10E-01	1.88E-02	1.29E-02	1.46E-01	-5.43E-02	3.02E-02	7.29E-02
alpha-CEHC sulfate	9.12E-03	5.26E-03	8.42E-02	1.42E-02	1.21E-02	2.43E-01	-1.27E-02	2.85E-02	6.55E-01
alpha-CMBHC glucuronide	3.78E-04	5.55E-03	9.46E-01	5.60E-03	1.28E-02	6.61E-01	-1.05E-02	2.99E-02	7.24E-01
sphingomyelin (d18:2/14:0, d18:1/14:1)*	-2.53E-03	4.94E-03	6.09E-01	-7.28E-03	1.14E-02	5.23E-01	-1.14E-03	2.66E-02	9.66E-01
sphingomyelin (d18:1/24:1, d18:2/24:0)*	-4.88E-03	5.81E-03	4.01E-01	-4.35E-02	1.31E-02	1.04E-03	1.66E-02	3.13E-02	5.96E-01
octadecenedioylcarnitine (C18:1-DC)*	-1.81E-04	5.95E-03	9.76E-01	1.62E-02	1.37E-02	2.37E-01	-3.30E-03	3.21E-02	9.18E-01
octadecanedioylcarnitine (C18-DC)*	-9.66E-03	5.89E-03	1.02E-01	6.07E-03	1.36E-02	6.56E-01	-6.08E-02	3.17E-02	5.62E-02
N,O-didesmethylvenlafaxine glucuronide	9.32E-04	1.66E-03	5.75E-01	1.57E-03	3.82E-03	6.81E-01	1.41E-02	8.91E-03	1.14E-01
5alpha-androstan-3alpha,17beta-diol monosulfate (2)	-1.02E-02	4.24E-03	1.66E-02	-1.57E-02	9.82E-03	1.10E-01	-5.03E-02	2.29E-02	2.90E-02
myristoleoylcarnitine (C14:1)*	-9.27E-03	5.88E-03	1.16E-01	-3.49E-02	1.34E-02	9.87E-03	-2.22E-02	3.18E-02	4.86E-01
N-formylphenylalanine	7.54E-03	6.02E-03	2.11E-01	8.11E-03	1.39E-02	5.60E-01	2.41E-02	3.25E-02	4.59E-01
cyclo(pro-val)	-7.60E-03	5.90E-03	1.99E-01	-6.83E-03	1.36E-02	6.16E-01	1.39E-03	3.19E-02	9.65E-01
4-hydroxychlorothalonil	-1.28E-02	5.58E-03	2.28E-02	-3.62E-02	1.28E-02	4.98E-03	-5.46E-02	3.02E-02	7.15E-02
isoeugenol sulfate	-3.80E-04	4.89E-03	9.38E-01	-2.11E-03	1.13E-02	8.51E-01	2.98E-02	2.63E-02	2.58E-01
tyramine O-sulfate	-5.27E-03	5.71E-03	3.57E-01	-1.14E-02	1.31E-02	3.86E-01	5.53E-03	3.08E-02	8.58E-01
3-hydroxypyridine sulfate	1.07E-02	5.61E-03	5.83E-02	-2.81E-03	1.30E-02	8.29E-01	3.16E-02	3.04E-02	3.00E-01
4-methylguaiacol sulfate	1.89E-02	6.04E-03	1.92E-03	3.86E-02	1.40E-02	6.02E-03	5.29E-02	3.30E-02	1.10E-01
maltol sulfate	5.02E-03	4.41E-03	2.57E-01	8.33E-03	1.02E-02	4.13E-01	2.08E-02	2.38E-02	3.84E-01
phenylacetylcarnitine	6.16E-03	5.62E-03	2.74E-01	3.18E-02	1.28E-02	1.35E-02	4.99E-02	3.02E-02	9.91E-02
arabonate/xylonate	1.13E-02	5.27E-03	3.35E-02	2.35E-02	1.21E-02	5.39E-02	6.00E-03	2.86E-02	8.34E-01
methyl-4-hydroxybenzoate sulfate	-2.44E-03	5.51E-03	6.58E-01	1.46E-03	1.27E-02	9.08E-01	-1.09E-02	2.97E-02	7.14E-01
1-dihomo-linolenylglycerol (20:3)	-1.89E-03	5.68E-03	7.40E-01	8.21E-04	1.31E-02	9.50E-01	-3.71E-02	3.05E-02	2.26E-01
vanillic alcohol sulfate	3.01E-03	4.84E-03	5.34E-01	2.42E-02	1.11E-02	2.96E-02	2.11E-02	2.61E-02	4.19E-01
4-vinylguaiacol sulfate	9.02E-03	5.07E-03	7.65E-02	1.76E-02	1.17E-02	1.34E-01	2.60E-02	2.75E-02	3.44E-01
vanillactate	-5.32E-03	5.54E-03	3.38E-01	9.14E-03	1.28E-02	4.74E-01	-3.46E-02	2.98E-02	2.47E-01
eugenol sulfate	5.86E-03	5.76E-03	3.10E-01	6.78E-03	1.33E-02	6.10E-01	3.76E-02	3.10E-02	2.27E-01
pregnanolone/allopregnanolone sulfate	4.22E-04	4.82E-03	9.30E-01	-7.97E-03	1.11E-02	4.73E-01	-2.87E-02	2.59E-02	2.70E-01
2-methoxyresorcinol sulfate	2.01E-03	5.11E-03	6.94E-01	-2.45E-03	1.18E-02	8.35E-01	4.26E-02	2.74E-02	1.21E-01
2-acetamidophenol sulfate	1.69E-02	5.59E-03	2.83E-03	5.34E-02	1.27E-02	3.51E-05	6.06E-02	3.04E-02	4.73E-02
p-cresol glucuronide*	2.61E-03	6.04E-03	6.66E-01	2.50E-02	1.38E-02	7.21E-02	-1.74E-02	3.26E-02	5.93E-01
acesulfame	-4.01E-03	5.17E-03	4.39E-01	-3.26E-04	1.19E-02	9.78E-01	-1.29E-02	2.79E-02	6.44E-01
valsartan	3.21E-04	1.38E-03	8.16E-01	-7.88E-04	3.17E-03	8.04E-01	-2.29E-03	7.43E-03	7.58E-01
6-hydroxyindole sulfate	-5.99E-03	6.08E-03	3.25E-01	3.81E-03	1.40E-02	7.86E-01	-4.48E-02	3.27E-02	1.72E-01
4-methoxyphenol sulfate	1.85E-02	5.66E-03	1.24E-03	5.96E-02	1.28E-02	4.83E-06	9.73E-02	3.05E-02	1.59E-03
2,4-dichlorophenol sulfate	9.35E-04	2.78E-03	7.37E-01	6.58E-04	6.40E-03	9.18E-01	-4.95E-04	1.50E-02	9.74E-01
propyl 4-hydroxybenzoate sulfate	-8.88E-03	4.77E-03	6.38E-02	-6.47E-03	1.10E-02	5.59E-01	-4.20E-02	2.58E-02	1.04E-01
ethylparaben sulfate	-3.29E-03	5.48E-03	5.49E-01	-3.20E-02	1.25E-02	1.09E-02	-2.67E-02	2.95E-02	3.67E-01
umbelliferone sulfate	3.72E-03	5.53E-03	5.02E-01	1.02E-02	1.27E-02	4.23E-01	3.72E-02	2.97E-02	2.11E-01
sphingomyelin (d18:1/20:0, d16:1/22:0)*	-6.41E-03	5.79E-03	2.70E-01	-1.66E-02	1.33E-02	2.14E-01	-5.03E-02	3.11E-02	1.07E-01
sphingomyelin (d18:1/20:1, d18:2/20:0)*	-9.62E-03	5.42E-03	7.72E-02	-1.30E-02	1.25E-02	2.99E-01	-1.64E-02	2.94E-02	5.76E-01
sphingomyelin (d18:1/20:2, d18:2/20:1, d16:1/22:2)*	-1.84E-02	5.35E-03	6.62E-04	-4.23E-02	1.23E-02	6.82E-04	-1.04E-01	2.88E-02	3.65E-04
behenoyl sphingomyelin (d18:1/22:0)*	-4.00E-03	6.12E-03	5.15E-01	-2.10E-02	1.40E-02	1.36E-01	-1.57E-03	3.30E-02	9.62E-01
sphingomyelin (d18:1/22:1, d18:2/22:0, d16:1/24:1)*	-2.58E-03	5.44E-03	6.36E-01	-7.53E-03	1.25E-02	5.48E-01	-2.34E-03	2.93E-02	9.37E-01
sphingomyelin (d18:1/22:2, d18:2/22:1, d16:1/24:2)*	-1.70E-02	5.58E-03	2.58E-03	-4.56E-02	1.28E-02	4.22E-04	-9.33E-02	3.01E-02	2.11E-03
lignoceroyl sphingomyelin (d18:1/24:0)	1.17E-02	5.97E-03	5.08E-02	-1.18E-03	1.38E-02	9.32E-01	7.19E-02	3.21E-02	2.60E-02
sphingomyelin (d17:1/16:0, d18:1/15:0, d16:1/17:0)*	-1.73E-02	5.48E-03	1.78E-03	-2.49E-02	1.27E-02	5.16E-02	-1.07E-01	2.93E-02	3.20E-04
dopamine 3-O-sulfate	2.23E-02	5.97E-03	2.24E-04	3.90E-02	1.39E-02	5.36E-03	6.43E-02	3.28E-02	5.08E-02
3-hydroxyhexanoate	-4.00E-03	5.58E-03	4.75E-01	-2.61E-03	1.29E-02	8.40E-01	-1.71E-02	3.01E-02	5.70E-01
N-carbamoylalanine	1.75E-02	5.48E-03	1.54E-03	4.79E-02	1.25E-02	1.61E-04	6.62E-02	2.98E-02	2.72E-02
3beta-hydroxy-5-cholestenoate	-7.36E-04	5.83E-03	9.00E-01	-1.31E-02	1.34E-02	3.31E-01	-1.01E-02	3.14E-02	7.48E-01
1,2,3-benzenetriol sulfate (2)	-2.40E-04	4.62E-03	9.59E-01	-6.66E-03	1.06E-02	5.31E-01	5.57E-03	2.49E-02	8.23E-01
3-methoxycatechol sulfate (1)	3.96E-03	6.08E-03	5.15E-01	1.05E-02	1.40E-02	4.55E-01	7.57E-02	3.25E-02	2.04E-02
3-methoxycatechol sulfate (2)	8.63E-03	5.66E-03	1.28E-01	2.95E-02	1.30E-02	2.37E-02	2.97E-02	3.06E-02	3.33E-01
N-acetylkynurenine (2)	-4.27E-03	5.48E-03	4.37E-01	-9.67E-03	1.26E-02	4.44E-01	-2.62E-02	2.95E-02	3.75E-01

Supplementary Table 2: Parameter estimates for metabolome-wide association studies for diet-metabolite associations for each of: The Healthy Eating Index-2015 (HEI-15), DASH and AMED diet

C-glycosyltryptophan	-1.12E-02	5.78E-03	5.44E-02	-3.70E-03	1.34E-02	7.83E-01	-9.30E-02	3.09E-02	2.82E-03
tramadol	-4.00E-04	1.05E-03	7.04E-01	-2.91E-03	2.41E-03	2.28E-01	-2.38E-03	5.66E-03	6.74E-01
O-desmethyltramadol	-4.00E-04	1.05E-03	7.04E-01	-2.91E-03	2.41E-03	2.28E-01	-2.38E-03	5.66E-03	6.74E-01
O-desmethyltramadol glucuronide	-4.00E-04	1.05E-03	7.04E-01	-2.91E-03	2.41E-03	2.28E-01	-2.38E-03	5.66E-03	6.74E-01
arabitol/xylitol	4.38E-03	5.53E-03	4.30E-01	9.49E-03	1.27E-02	4.57E-01	5.30E-03	2.99E-02	8.59E-01
N-acetylglucosamine/N-acetylgalactosamine	-1.02E-02	5.89E-03	8.40E-02	-1.34E-02	1.36E-02	3.25E-01	-6.96E-02	3.16E-02	2.87E-02
citraconate/glutaconate	6.12E-03	5.69E-03	2.83E-01	-1.02E-02	1.31E-02	4.36E-01	6.30E-03	3.07E-02	8.38E-01
adipoylcarnitine (C6-DC)	-2.08E-02	5.76E-03	3.61E-04	-9.47E-03	1.35E-02	4.85E-01	-8.54E-02	3.13E-02	6.84E-03
nonanoylcarnitine (C9)	-1.77E-02	5.94E-03	3.17E-03	-3.94E-02	1.37E-02	4.28E-03	-5.54E-02	3.23E-02	8.77E-02
glycochenodeoxycholate 3-sulfate	-3.35E-03	5.47E-03	5.40E-01	-1.01E-02	1.26E-02	4.23E-01	-2.93E-02	2.94E-02	3.20E-01
glycodeoxycholate 3-sulfate	5.52E-03	5.77E-03	3.39E-01	3.45E-03	1.33E-02	7.96E-01	-2.48E-02	3.11E-02	4.26E-01
taurodeoxycholic acid 3-sulfate	8.34E-04	5.81E-03	8.86E-01	-1.10E-02	1.34E-02	4.11E-01	-4.99E-02	3.12E-02	1.11E-01
trans-3,4-methyleneheptanoate	-8.61E-03	5.58E-03	1.24E-01	-1.08E-02	1.29E-02	4.02E-01	-2.95E-02	3.01E-02	3.29E-01
phenol glucuronide	-7.01E-03	5.68E-03	2.18E-01	-6.03E-03	1.31E-02	6.46E-01	-8.39E-03	3.07E-02	7.85E-01
linoleoyl ethanolamide	-5.86E-03	5.71E-03	3.05E-01	-7.41E-03	1.32E-02	5.74E-01	-1.34E-02	3.08E-02	6.65E-01
1,2-dilinoleoyl-GPC (18:2/18:2)	1.16E-02	5.82E-03	4.74E-02	2.96E-02	1.34E-02	2.79E-02	6.79E-02	3.13E-02	3.09E-02
1-stearoyl-2-oleoyl-GPC (18:0/18:1)	3.88E-03	6.12E-03	5.27E-01	-6.98E-03	1.41E-02	6.21E-01	3.88E-02	3.29E-02	2.40E-01
1-palmitoyl-2-arachidonoyl-GPC (16:0/20:4n6)	-1.54E-02	5.94E-03	9.86E-03	-6.85E-02	1.32E-02	4.07E-07	-6.01E-02	3.22E-02	6.30E-02
1-palmitoyl-2-docosahexaenoyl-GPC (16:0/22:6)	7.95E-03	5.90E-03	1.79E-01	-2.34E-02	1.35E-02	8.50E-02	6.41E-02	3.16E-02	4.37E-02
1-stearoyl-2-docosahexaenoyl-GPC (18:0/22:6)	7.36E-03	5.74E-03	2.01E-01	-4.29E-03	1.32E-02	7.46E-01	6.87E-02	3.07E-02	2.63E-02
1-(1-enyl-stearoyl)-2-oleoyl-GPC (P-18:0/18:1)	8.09E-03	5.82E-03	1.66E-01	-3.73E-03	1.34E-02	7.81E-01	5.64E-02	3.13E-02	7.26E-02
1-(1-enyl-stearoyl)-2-arachidonoyl-GPC (P-18:0/20:4)	-1.14E-02	5.70E-03	4.63E-02	-3.03E-02	1.31E-02	2.14E-02	-4.38E-02	3.08E-02	1.56E-01
1-(1-enyl-stearoyl)-2-oleoyl-GPE (P-18:0/18:1)	5.34E-03	6.04E-03	3.77E-01	-2.32E-03	1.39E-02	8.68E-01	4.83E-02	3.25E-02	1.38E-01
sphingomyelin (d18:1/17:0, d17:1/18:0, d19:1/16:0)	-1.94E-02	5.60E-03	6.19E-04	-3.97E-02	1.30E-02	2.43E-03	-9.54E-02	3.03E-02	1.84E-03
1-palmitoyl-2-stearoyl-GPC (16:0/18:0)	2.29E-03	6.10E-03	7.07E-01	-1.59E-02	1.40E-02	2.56E-01	5.23E-02	3.27E-02	1.11E-01
2-hydroxybutyrate/2-hydroxyisobutyrate	-2.51E-03	5.81E-03	6.66E-01	-3.09E-02	1.33E-02	2.04E-02	1.32E-02	3.13E-02	6.73E-01
oleate/vaccenate (18:1)	-1.03E-02	5.55E-03	6.46E-02	-2.73E-02	1.28E-02	3.33E-02	-4.95E-02	3.00E-02	1.00E-01
isoleucylleucine/leucylisoleucine	8.71E-03	5.94E-03	1.44E-01	3.18E-02	1.36E-02	2.01E-02	6.95E-02	3.19E-02	3.01E-02
aripiprazole	-1.51E-03	1.39E-03	2.80E-01	-1.41E-03	3.20E-03	6.60E-01	-2.64E-03	7.50E-03	7.25E-01
rivaroxaban	8.65E-04	1.06E-03	4.16E-01	-2.89E-05	2.45E-03	9.91E-01	3.04E-03	5.73E-03	5.96E-01
leucylphenylalanine/isoleucylphenylalanine	1.14E-02	6.01E-03	5.90E-02	2.15E-02	1.39E-02	1.21E-01	4.38E-02	3.25E-02	1.78E-01
1-palmitoleoylglycerol (16:1)*	1.70E-03	5.55E-03	7.60E-01	-1.39E-02	1.28E-02	2.78E-01	-3.96E-02	2.98E-02	1.85E-01
palmitoyl dihydrosphingomyelin (d18:0/16:0)*	9.85E-03	5.92E-03	9.72E-02	-1.98E-02	1.36E-02	1.48E-01	7.51E-02	3.17E-02	1.87E-02
tricosanoyl sphingomyelin (d18:1/23:0)*	2.86E-03	5.64E-03	6.12E-01	-1.71E-02	1.29E-02	1.88E-01	1.15E-02	3.04E-02	7.07E-01
sphingomyelin (d18:2/23:0, d18:1/23:1, d17:1/24:1)*	-2.83E-03	5.10E-03	5.80E-01	-1.79E-02	1.17E-02	1.26E-01	3.93E-03	2.75E-02	8.86E-01
sphingomyelin (d18:2/24:1, d18:1/24:2)*	-3.91E-03	5.36E-03	4.66E-01	-3.63E-02	1.22E-02	3.04E-03	9.10E-03	2.89E-02	7.53E-01
diclofenac	8.96E-04	1.67E-03	5.91E-01	-3.77E-04	3.84E-03	9.22E-01	-8.10E-03	8.97E-03	3.68E-01
1-stearoyl-2-linoleoyl-GPE (18:0/18:2)*	9.16E-03	6.06E-03	1.32E-01	1.32E-02	1.40E-02	3.47E-01	5.77E-02	3.26E-02	7.80E-02
1-stearoyl-2-arachidonoyl-GPE (18:0/20:4)	2.06E-03	6.14E-03	7.38E-01	-8.41E-03	1.41E-02	5.52E-01	2.90E-02	3.31E-02	3.81E-01
1-stearoyl-2-linoleoyl-GPC (18:0/18:2)*	3.98E-03	5.88E-03	4.99E-01	7.25E-03	1.35E-02	5.92E-01	5.61E-02	3.15E-02	7.62E-02
1-palmitoyl-2-palmitoleoyl-GPC (16:0/16:1)*	-1.08E-02	5.86E-03	6.65E-02	-4.87E-02	1.33E-02	2.86E-04	-6.55E-02	3.16E-02	3.88E-02
1-palmitoyl-2-eicosapentaenoyl-GPC (16:0/20:5)*	5.82E-03	6.03E-03	3.35E-01	-2.13E-02	1.39E-02	1.25E-01	5.75E-02	3.24E-02	7.68E-02
1-palmitoyl-2-arachidonoyl-GPE (16:0/20:4)*	2.78E-03	6.11E-03	6.49E-01	-2.02E-02	1.40E-02	1.51E-01	1.76E-02	3.29E-02	5.93E-01
1-palmitoyl-2-docosahexaenoyl-GPE (16:0/22:6)*	8.70E-03	6.03E-03	1.50E-01	-1.48E-02	1.39E-02	2.90E-01	5.03E-02	3.25E-02	1.22E-01
1-stearoyl-2-docosahexaenoyl-GPE (18:0/22:6)*	4.20E-03	5.98E-03	4.83E-01	-1.11E-02	1.38E-02	4.23E-01	2.06E-02	3.22E-02	5.24E-01
1-palmitoyl-2-arachidonoyl-GPI (16:0/20:4)*	-5.61E-03	6.17E-03	3.64E-01	-2.52E-02	1.41E-02	7.63E-02	-3.34E-02	3.33E-02	3.16E-01
1-stearoyl-2-linoleoyl-GPI (18:0/18:2)	1.39E-02	5.80E-03	1.69E-02	7.21E-03	1.35E-02	5.93E-01	9.44E-02	3.11E-02	2.61E-03
1-palmitoyl-2-palmitoleoyl-GPE (16:0/16:1)*	-2.93E-03	4.68E-03	5.31E-01	-1.77E-02	1.07E-02	9.92E-02	-3.36E-02	2.51E-02	1.82E-01
gamma-tocopherol/beta-tocopherol	-1.55E-02	6.14E-03	1.18E-02	-3.32E-02	1.41E-02	1.98E-02	-3.13E-02	3.34E-02	3.49E-01
1-(1-enyl-stearoyl)-2-arachidonoyl-GPE (P-18:0/20:4)*	-1.41E-02	5.54E-03	1.15E-02	-4.32E-02	1.26E-02	7.25E-04	-3.00E-02	3.01E-02	3.21E-01
1-(1-enyl-palmitoyl)-2-docosahexaenoyl-GPE (P-16:0/22:6)*	6.17E-03	5.61E-03	2.72E-01	-2.08E-02	1.29E-02	1.07E-01	7.32E-02	3.00E-02	1.52E-02
1-(1-enyl-palmitoyl)-2-arachidonoyl-GPE (P-16:0/20:4)*	-3.97E-03	5.80E-03	4.94E-01	-2.58E-02	1.33E-02	5.27E-02	-8.92E-03	3.13E-02	7.75E-01
1-(1-enyl-oleoyl)-2-linoleoyl-GPE (P-18:1/18:2)*	7.21E-03	6.01E-03	2.31E-01	1.08E-02	1.39E-02	4.38E-01	7.11E-02	3.22E-02	2.81E-02
1-(1-enyl-stearoyl)-2-docosahexaenoyl-GPE (P-18:0/22:6)*	6.84E-03	5.62E-03	2.25E-01	-1.03E-02	1.30E-02	4.27E-01	8.91E-02	2.99E-02	3.17E-03
1-(1-enyl-palmitoyl)-2-oleoyl-GPE (P-16:0/18:1)*	8.13E-03	6.01E-03	1.77E-01	-1.43E-03	1.39E-02	9.18E-01	6.10E-02	3.23E-02	5.97E-02
1-(1-enyl-palmitoyl)-2-dihomo-linolenoyl-GPC (P-16:0/20:3)*	1.12E-03	6.03E-03	8.53E-01	-1.40E-02	1.39E-02	3.14E-01	-3.05E-02	3.25E-02	3.49E-01
1-(1-enyl-palmitoyl)-2-oleoyl-GPC (P-16:0/18:1)*	7.70E-03	5.74E-03	1.81E-01	-7.16E-03	1.33E-02	5.89E-01	5.97E-02	3.08E-02	5.38E-02
1-(1-enyl-palmitoyl)-2-docosahexaenoyl-GPC (P-16:0/22:6)*	9.94E-03	5.64E-03	7.91E-02	-1.49E-02	1.30E-02	2.55E-01	8.99E-02	3.01E-02	3.08E-03
1-(1-enyl-palmitoyl)-2-linoleoyl-GPC (P-16:0/18:2)*	2.24E-03	5.83E-03	7.01E-01	3.21E-03	1.34E-02	8.11E-01	4.51E-02	3.13E-02	1.51E-01
1-(1-enyl-palmitoyl)-2-arachidonoyl-GPC (P-16:0/20:4)*	-1.18E-02	5.56E-03	3.55E-02	-4.61E-02	1.26E-02	3.07E-04	-4.27E-02	3.01E-02	1.57E-01
1-(1-enyl-stearoyl)-2-docosahexaenoyl-GPC (P-18:0/22:6)*	9.02E-03	5.51E-03	1.02E-01	-3.75E-03	1.27E-02	7.69E-01	9.00E-02	2.93E-02	2.37E-03
1-stearyl-2-arachidonoyl-GPC (O-18:0/20:4)*	-2.25E-02	5.85E-03	1.49E-04	-2.47E-02	1.37E-02	7.33E-02	-1.33E-01	3.14E-02	3.01E-05
1-palmityl-2-oleoyl-GPC (O-16:0/18:1)*	-2.56E-04	5.99E-03	9.66E-01	-9.54E-03	1.38E-02	4.89E-01	3.30E-03	3.23E-02	9.19E-01
1-palmityl-2-arachidonoyl-GPC (O-16:0/20:4)*	-1.84E-02	5.70E-03	1.36E-03	-5.02E-02	1.30E-02	1.45E-04	-7.69E-02	3.09E-02	1.35E-02
sphingomyelin (d18:1/21:0, d17:1/22:0, d16:1/23:0)*	-3.15E-03	5.44E-03	5.64E-01	-6.09E-03	1.25E-02	6.27E-01	-4.19E-02	2.92E-02	1.53E-01
behenoyl dihydrosphingomyelin (d18:0/22:0)*	-3.55E-03	6.14E-03	5.64E-01	-4.05E-02	1.39E-02	3.92E-03	3.13E-04	3.31E-02	9.92E-01
sphingomyelin (d18:0/18:0, d19:0/17:0)*	-2.42E-02	5.89E-03	5.42E-05	-8.82E-02	1.29E-02	5.42E-11	-9.62E-02	3.22E-02	3.03E-03
N-palmitoyl-sphinganine (d18:0/16:0)	-1.47E-03	5.98E-03	8.06E-01	-3.17E-02	1.36E-02	2.10E-02	1.40E-02	3.22E-02	6.64E-01
lactosyl-N-palmitoyl-sphingosine (d18:1/16:0)	-6.94E-03	5.81E-03	2.33E-01	-3.56E-03	1.34E-02	7.90E-01	-2.36E-02	3.13E-02	4.52E-01
cetirizine	3.42E-03	3.42E-03	3.18E-01	6.41E-03	7.88E-03	4.17E-01	7.14E-04	1.85E-02	9.69E-01
1-pentadecanoyl-2-linoleoyl-GPC (15:0/18:2)*	-7.54E-03	5.24E-03	1.51E-01	9.77E-03	1.21E-02	4.20E-01	-6.71E-02	2.80E-02	1.73E-02
1-margaroyl-2-oleoyl-GPC (17:0/18:1)*	7.85E-03	5.67E-03	1.67E-01	1.71E-02	1.31E-02	1.92E-01	3.60E-02	3.06E-02	2.40E-01
1-margaroyl-2-linoleoyl-GPC (17:0/18:2)*	1.13E-02	5.57E-03	4.40E-02	4.68E-02	1.26E-02	2.47E-04	6.46E-02	3.00E-02	3.19E-02
myristoyl dihydrosphingomyelin (d18:0/14:0)*	-4.11E-03	5.56E-03	4.61E-01	-2.25E-02	1.28E-02	7.85E-02	-3.57E-02	2.99E-02	2.34E-01
5-hydroxyindole sulfate	-1.44E-03	5.90E-03	8.07E-01	6.12E-03	1.36E-02	6.52E-01	-2.38E-02	3.18E-02	4.54E-01
7-hydroxyindole sulfate	6.94E-03	5.06E-03	1.72E-01	1.44E-02	1.17E-02	2.18E-01	4.37E-02	2.73E-02	1.10E-01
phenylacetylglutamate	-3.47E-03	5.55E-03	5.33E-01	1.11E-02	1.28E-02	3.86E-01	-1.24E-02	2.99E-02	6.78E-01
1-stearoyl-2-dihomo-linolenoyl-GPC (18:0/20:3n3 or 6)*	1.19E-03	6.18E-03	8.47E-01	-1.45E-03	1.42E-02	9.19E-01	-3.86E-02	3.32E-02	2.46E-01
palmitoyl-linoleoyl-glycerol (16:0/18:2) [1]*	1.87E-04	5.88E-03	9.75E-01	-4.41E-04	1.35E-02	9.74E-01	-2.74E-02	3.17E-02	3.88E-01
palmitoyl-linoleoyl-glycerol (16:0/18:2) [2]*	-4.44E-03	5.90E-03	4.52E-01	-1.46E-02	1.36E-02	2.83E-01	-5.06E-02	3.17E-02	1.12E-01
1-palmitoyl-2-oleoyl-GPI (16:0/18:1)*	3.53E-03	6.11E-03	5.64E-01	-1.46E-02	1.41E-02	2.99E-01	2.24E-02	3.29E-02	4.97E-01
1-stearoyl-2-docosahexaenoyl-GPI (18:0/22:6)*	7.30E-03	6.18E-03	2.39E-01	-1.42E-02	1.42E-02	3.20E-01	6.37E-02	3.32E-02	5.59E-02
1-(1-enyl-palmitoyl)-2-linoleoyl-GPE (P-16:0/18:2)*	-2.28E-03	6.04E-03	7.06E-01	3.29E-03	1.39E-02	8.13E-01	1.81E-03	3.26E-02	9.56E-01
1-oleoyl-2-linoleoyl-GPE (18:1/18:2)*	2.15E-02	5.75E-03	2.17E-04	4.70E-02	1.33E-02	4.60E-04	1.00E-01	3.12E-02	1.48E-03

Supplementary Table 2: Parameter estimates for metabolome-wide association studies for diet-metabolite associations for each of: The Healthy Eating Index-2015 (HEI-15), DASH and AMED diet

1-pentadecanoyl-2-arachidonoyl-GPC (15:0/20:4)*	-1.61E-02	5.84E-03	6.29E-03	-1.45E-02	1.36E-02	2.88E-01	-1.12E-01	3.12E-02	3.94E-04
1-pentadecanoyl-2-docosahexaenoyl-GPC (15:0/22:6)*	6.69E-03	5.56E-03	2.30E-01	1.46E-02	1.28E-02	2.55E-01	3.50E-02	3.00E-02	2.44E-01
1-margaroyl-2-arachidonoyl-GPC (17:0/20:4)*	-1.19E-02	5.97E-03	4.78E-02	-8.64E-03	1.38E-02	5.32E-01	-7.00E-02	3.21E-02	3.02E-02
1-arachidoyl-2-arachidonoyl-GPC (20:0/20:4)*	1.22E-02	6.02E-03	4.31E-02	1.30E-02	1.39E-02	3.51E-01	6.42E-02	3.24E-02	4.89E-02
1-oleoyl-2-docosahexaenoyl-GPC (18:1/22:6)*	2.25E-02	5.46E-03	4.94E-05	3.45E-02	1.28E-02	7.33E-03	1.20E-01	2.94E-02	6.06E-05
1-linoleoyl-2-arachidonoyl-GPC (18:2/20:4n6)*	8.31E-03	5.78E-03	1.52E-01	-1.43E-03	1.34E-02	9.15E-01	5.70E-02	3.11E-02	6.79E-02
1-linoleoyl-2-docosahexaenoyl-GPC (18:2/22:6)*	1.94E-02	5.51E-03	5.00E-04	6.11E-03	1.30E-02	6.38E-01	1.22E-01	2.95E-02	4.67E-05
1-palmityl-2-linoleoyl-GPC (O-16:0/18:2)*	-3.93E-03	5.96E-03	5.10E-01	-1.72E-02	1.37E-02	2.10E-01	-6.65E-03	3.21E-02	8.36E-01
1-(1-enyl-stearoyl)-2-linoleoyl-GPC (P-18:0/18:2)*	3.32E-03	5.90E-03	5.75E-01	-2.86E-03	1.36E-02	8.33E-01	5.04E-02	3.17E-02	1.13E-01
1-myristoyl-2-linoleoyl-GPC (14:0/18:2)*	-4.65E-03	5.90E-03	4.32E-01	-1.52E-02	1.36E-02	2.64E-01	-3.60E-02	3.18E-02	2.58E-01
1-myristoyl-2-arachidonoyl-GPC (14:0/20:4)*	-9.45E-04	6.00E-03	8.75E-01	-2.76E-02	1.37E-02	4.49E-02	-1.54E-02	3.23E-02	6.33E-01
1-myristoyl-2-docosahexaenoyl-GPC (14:0/22:6)*	1.38E-02	5.75E-03	1.69E-02	6.79E-04	1.34E-02	9.60E-01	8.80E-02	3.09E-02	4.72E-03
1-stearoyl-2-docosapentaenoyl-GPC (18:0/22:5n3)*	1.53E-03	6.10E-03	8.02E-01	1.51E-02	1.40E-02	2.81E-01	6.24E-03	3.29E-02	8.49E-01
1-stearoyl-2-docosapentaenoyl-GPC (18:0/22:5n6)*	-1.71E-02	5.59E-03	2.48E-03	-3.34E-02	1.29E-02	1.03E-02	-1.36E-01	2.95E-02	5.87E-06
1-palmitoyl-2-adrenoyl-GPC (16:0/22:4)*	-1.16E-02	5.90E-03	5.10E-02	-3.19E-02	1.35E-02	1.92E-02	-8.24E-02	3.16E-02	9.67E-03
1-stearoyl-2-adrenoyl-GPC (18:0/22:4)*	-1.64E-02	5.57E-03	3.49E-03	-3.57E-02	1.28E-02	5.78E-03	-9.03E-02	3.00E-02	2.86E-03
1-stearyl-GPC (O-18:0)*	3.06E-03	5.50E-03	5.79E-01	8.09E-03	1.27E-02	5.23E-01	4.70E-03	2.97E-02	8.74E-01
1-myristoyl-2-palmitoleoyl-GPC (14:0/16:1)*	-5.94E-03	5.82E-03	3.08E-01	-1.91E-02	1.34E-02	1.53E-01	-5.24E-02	3.13E-02	9.51E-02
1-stearoyl-2-meadoyl-GPC (18:0/20:3n9)*	-5.02E-03	5.53E-03	3.65E-01	-2.04E-02	1.27E-02	1.09E-01	-3.17E-02	2.98E-02	2.87E-01
1-(1-enyl-palmitoyl)-2-palmitoleoyl-GPC (P-16:0/16:1)*	-2.67E-03	5.87E-03	6.50E-01	-3.92E-02	1.33E-02	3.53E-03	6.76E-03	3.17E-02	8.31E-01
1-(1-enyl-palmitoyl)-2-myristoyl-GPC (P-16:0/14:0)*	-1.66E-02	5.88E-03	5.16E-03	-3.09E-02	1.36E-02	2.39E-02	-5.93E-02	3.19E-02	6.42E-02
1-(1-enyl-palmitoyl)-2-palmitoyl-GPC (P-16:0/16:0)*	-8.84E-03	5.95E-03	1.39E-01	-3.97E-02	1.36E-02	3.64E-03	-1.01E-02	3.22E-02	7.55E-01
phosphatidylcholine (16:0/22:5n3, 18:1/20:4)*	4.66E-03	5.89E-03	4.30E-01	1.21E-02	1.36E-02	3.73E-01	5.59E-02	3.16E-02	7.81E-02
phosphatidylcholine (18:0/20:5, 16:0/22:5n6)*	4.52E-03	5.55E-03	4.16E-01	-1.15E-02	1.28E-02	3.67E-01	2.64E-02	2.99E-02	3.77E-01
1-palmitoyl-2-docosahexaenoyl-GPI (16:0/22:6)*	3.95E-03	6.10E-03	5.17E-01	-6.84E-03	1.40E-02	6.27E-01	4.12E-02	3.28E-02	2.10E-01
1-stearoyl-2-oleoyl-GPI (18:0/18:1)*	2.21E-02	6.01E-03	2.83E-04	3.17E-02	1.40E-02	2.47E-02	1.10E-01	3.25E-02	8.14E-04
1-stearoyl-2-dihomo-linolenoyl-GPI (18:0/20:3n3 or 6)*	1.30E-02	5.78E-03	2.52E-02	1.02E-02	1.34E-02	4.47E-01	3.89E-02	3.13E-02	2.16E-01
1-stearyl-GPE (O-18:0)*	7.32E-03	5.51E-03	1.85E-01	1.29E-02	1.27E-02	3.09E-01	5.58E-02	2.96E-02	6.03E-02
1,2-dipalmitoyl-GPE (16:0/16:0)*	4.59E-03	5.03E-03	3.62E-01	-6.40E-03	1.16E-02	5.81E-01	-1.48E-02	2.71E-02	5.85E-01
1-palmitoyl-2-stearoyl-GPE (16:0/18:0)*	2.52E-03	5.44E-03	6.43E-01	7.24E-03	1.25E-02	5.64E-01	2.69E-02	2.93E-02	3.59E-01
1-palmitoyl-2-eicosapentaenoyl-GPE (16:0/20:5)*	7.48E-03	5.32E-03	1.61E-01	-6.49E-05	1.23E-02	9.96E-01	4.50E-02	2.86E-02	1.17E-01
1-stearoyl-2-dihomo-linolenoyl-GPE (18:0/20:3n3 or 6)*	1.26E-02	6.10E-03	3.95E-02	2.16E-02	1.41E-02	1.26E-01	2.99E-02	3.31E-02	3.67E-01
1,2-dilinoleoyl-GPE (18:2/18:2)*	4.93E-03	5.34E-03	3.57E-01	1.53E-02	1.23E-02	2.15E-01	6.49E-03	2.88E-02	8.22E-01
1-oleoyl-2-arachidonoyl-GPE (18:1/20:4)*	1.30E-02	6.19E-03	3.61E-02	2.41E-02	1.43E-02	9.27E-02	8.24E-02	3.32E-02	1.38E-02
1-(1-enyl-stearoyl)-2-linoleoyl-GPE (P-18:0/18:2)*	1.57E-05	5.97E-03	9.98E-01	8.59E-04	1.37E-02	9.50E-01	3.40E-02	3.21E-02	2.90E-01
1-linoleoyl-GPG (18:2)*	-4.99E-03	6.04E-03	4.09E-01	-4.64E-03	1.39E-02	7.39E-01	-4.75E-02	3.24E-02	1.44E-01
thioproline	-1.60E-02	5.69E-03	5.19E-03	-3.65E-02	1.31E-02	5.69E-03	-5.02E-02	3.09E-02	1.06E-01
palmitoylcholine	1.47E-02	5.90E-03	1.35E-02	3.10E-02	1.36E-02	2.35E-02	8.34E-02	3.17E-02	9.07E-03
trans-3,4-methyleneheptanoylcarnitine	-1.26E-02	5.73E-03	2.82E-02	-9.55E-03	1.33E-02	4.73E-01	-3.39E-02	3.11E-02	2.77E-01
glycocholate glucuronide (1)	9.80E-04	5.12E-03	8.48E-01	2.12E-03	1.18E-02	8.57E-01	5.68E-03	2.76E-02	8.37E-01
glycochenodeoxycholate glucuronide (1)	-8.36E-03	5.50E-03	1.30E-01	-2.31E-02	1.26E-02	6.93E-02	-5.43E-02	2.96E-02	6.77E-02
(S)-3-hydroxybutyrylcarnitine	-1.07E-02	5.52E-03	5.41E-02	-7.04E-03	1.28E-02	5.83E-01	-3.59E-02	2.99E-02	2.31E-01
glycosyl-N-palmitoyl-sphingosine (d18:1/16:0)	-1.48E-02	6.02E-03	1.47E-02	-3.75E-02	1.38E-02	7.11E-03	-4.32E-02	3.27E-02	1.87E-01
catechol glucuronide	1.93E-02	4.71E-03	5.42E-05	3.60E-02	1.10E-02	1.14E-03	6.91E-02	2.58E-02	7.90E-03
ascorbic acid 2-sulfate	2.08E-02	5.57E-03	2.29E-04	5.51E-02	1.27E-02	2.09E-05	5.37E-02	3.06E-02	8.06E-02
oleoylcholine	1.90E-02	5.84E-03	1.24E-03	4.31E-02	1.34E-02	1.52E-03	1.00E-01	3.15E-02	1.67E-03
arachidonoylcholine	9.44E-03	5.88E-03	1.10E-01	2.15E-02	1.35E-02	1.14E-01	6.57E-02	3.16E-02	3.85E-02
docosahexaenoylcholine	1.90E-02	5.64E-03	8.82E-04	2.90E-02	1.31E-02	2.80E-02	1.20E-01	3.01E-02	8.76E-05
palmitoloelycholine	7.35E-03	5.12E-03	1.52E-01	7.17E-03	1.18E-02	5.45E-01	2.76E-02	2.77E-02	3.19E-01
dihomo-linolenoyl-choline	1.67E-02	5.91E-03	5.21E-03	3.99E-02	1.36E-02	3.63E-03	5.75E-02	3.21E-02	7.48E-02
caffeic acid sulfate	1.17E-02	4.97E-03	1.94E-02	1.89E-02	1.15E-02	1.01E-01	4.99E-02	2.69E-02	6.46E-02
1-linoleoyl-2-linolenoyl-GPC (18:2/18:3)*	1.15E-02	5.73E-03	4.62E-02	2.21E-02	1.32E-02	9.51E-02	9.02E-02	3.06E-02	3.53E-03
1-palmitoleoyl-2-linolenoyl-GPC (16:1/18:3)*	-3.60E-04	5.36E-03	9.46E-01	-2.51E-03	1.23E-02	8.39E-01	1.59E-02	2.89E-02	5.82E-01
phosphatidylcholine (14:0/14:0, 16:0/12:0)	-4.90E-03	6.03E-03	4.17E-01	-1.31E-02	1.39E-02	3.45E-01	-4.62E-02	3.24E-02	1.55E-01
phosphatidylcholine (15:0/18:1, 17:0/16:1, 16:0/17:1)*	-9.72E-03	5.52E-03	7.93E-02	-1.70E-02	1.27E-02	1.82E-01	-7.36E-02	2.96E-02	1.34E-02
1-oleoyl-2-dihomo-linoleoyl-GPC (18:1/20:2)*	1.86E-02	5.69E-03	1.24E-03	3.40E-02	1.32E-02	1.05E-02	6.30E-02	3.10E-02	4.31E-02
1-oleoyl-2-docosapentaenoyl-GPC (18:1/22:5n3)*	1.26E-02	5.76E-03	2.93E-02	3.24E-02	1.32E-02	1.51E-02	5.19E-02	3.12E-02	9.73E-02
phosphatidylcholine (18:0/20:2, 20:0/18:2)*	2.55E-02	5.98E-03	2.78E-05	3.12E-02	1.41E-02	2.75E-02	1.18E-01	3.25E-02	3.54E-04
1-(1-enyl-oleoyl)-2-docosahexaenoyl-GPE (P-18:1/22:6)*	1.69E-02	5.58E-03	2.68E-03	2.57E-03	1.31E-02	8.44E-01	1.41E-01	2.94E-02	2.78E-06
1-(1-enyl-stearoyl)-2-dihomo-linolenoyl-GPE (P-18:0/20:3)*	-4.03E-03	6.04E-03	5.05E-01	-1.35E-02	1.39E-02	3.31E-01	-4.40E-02	3.25E-02	1.77E-01
lisinopril	-1.88E-03	2.38E-03	4.29E-01	-2.07E-04	5.48E-03	9.70E-01	1.54E-02	1.28E-02	2.29E-01
lamotrigine	-4.61E-05	1.42E-03	9.74E-01	-4.36E-03	3.25E-03	1.81E-01	-6.62E-04	7.64E-03	9.31E-01
hexadecatrienoate (16:3n3)	1.60E-02	5.18E-03	2.18E-03	2.93E-02	1.20E-02	1.51E-02	7.03E-02	2.81E-02	1.28E-02
hexadecadienoate (16:2n6)	-1.72E-02	5.58E-03	2.30E-03	-3.26E-02	1.29E-02	1.22E-02	-6.36E-02	3.03E-02	3.68E-02
1-myristoyl-2-eicosapentaenoyl-GPC (14:0/20:5)*	1.02E-02	5.81E-03	7.96E-02	-2.27E-03	1.34E-02	8.66E-01	6.72E-02	3.12E-02	3.23E-02
palmitoleoylcarnitine (C16:1)*	-1.44E-02	5.60E-03	1.05E-02	-4.75E-02	1.27E-02	2.35E-04	-5.99E-02	3.03E-02	4.93E-02
4-acetamidobenzoate	1.29E-02	4.81E-03	7.59E-03	3.43E-02	1.10E-02	2.09E-03	7.40E-02	2.59E-02	4.62E-03
4-hydroxyphenylacetylglutamine	-2.39E-04	5.89E-03	9.68E-01	1.11E-02	1.35E-02	4.13E-01	2.21E-02	3.17E-02	4.86E-01
4-aminophenol sulfate (2)	2.43E-03	1.06E-03	2.26E-02	5.22E-03	2.44E-03	3.32E-02	6.68E-03	5.74E-03	2.46E-01
2'-O-methyluridine	-9.90E-03	5.80E-03	8.86E-02	-1.90E-03	1.34E-02	8.87E-01	-4.33E-02	3.13E-02	1.68E-01
gamma-glutamyl-alpha-lysine	-5.35E-03	5.95E-03	3.69E-01	-3.45E-03	1.37E-02	8.01E-01	-4.82E-02	3.20E-02	1.33E-01
palmitoyl-oleoyl-glycerol (16:0/18:1) [1]*	-1.92E-03	5.87E-03	7.45E-01	-1.59E-02	1.35E-02	2.41E-01	-4.52E-02	3.15E-02	1.53E-01
palmitoyl-oleoyl-glycerol (16:0/18:1) [2]*	-3.24E-03	5.91E-03	5.84E-01	-1.65E-02	1.36E-02	2.24E-01	-4.43E-02	3.17E-02	1.64E-01
oleoyl-oleoyl-glycerol (18:1/18:1) [1]*	1.11E-02	5.93E-03	6.34E-02	2.10E-02	1.37E-02	1.25E-01	3.56E-02	3.21E-02	2.68E-01
oleoyl-oleoyl-glycerol (18:1/18:1) [2]*	1.32E-02	5.93E-03	2.68E-02	1.92E-02	1.37E-02	1.63E-01	3.95E-02	3.22E-02	2.21E-01
linoleoyl-arachidonoyl-glycerol (18:2/20:4) [1]*	-3.76E-03	5.83E-03	5.19E-01	-9.04E-03	1.34E-02	5.01E-01	-1.11E-02	3.14E-02	7.25E-01
linoleoyl-arachidonoyl-glycerol (18:2/20:4) [2]*	-7.41E-03	5.92E-03	2.12E-01	-2.33E-02	1.36E-02	8.83E-02	-2.20E-02	3.20E-02	4.92E-01
palmitoyl-arachidonoyl-glycerol (16:0/20:4) [1]*	-9.39E-03	5.52E-03	9.00E-02	-3.00E-02	1.26E-02	1.81E-02	-8.74E-02	2.94E-02	3.23E-03
palmitoyl-arachidonoyl-glycerol (16:0/20:4) [2]*	-1.07E-02	5.60E-03	5.76E-02	-3.63E-02	1.28E-02	4.86E-03	-5.33E-02	3.02E-02	7.88E-02
linoleoyl-linolenoyl-glycerol (18:2/18:3) [1]*	5.88E-03	5.59E-03	2.93E-01	1.92E-02	1.28E-02	1.35E-01	1.65E-02	3.02E-02	5.85E-01
linoleoyl-linolenoyl-glycerol (18:2/18:3) [2]*	6.63E-03	5.86E-03	2.59E-01	1.61E-02	1.35E-02	2.35E-01	4.86E-02	3.15E-02	1.25E-01
linoleoyl-docosahexaenoyl-glycerol (18:2/22:6) [2]*	1.06E-02	5.92E-03	7.40E-02	-2.82E-03	1.37E-02	8.37E-01	3.79E-02	3.20E-02	2.37E-01
palmitoleoyl-linoleoyl-glycerol (16:1/18:2) [1]*	-7.30E-03	5.66E-03	1.98E-01	-2.22E-02	1.30E-02	8.83E-02	-3.96E-02	3.05E-02	1.95E-01
diacylglycerol (14:0/18:1, 16:0/16:1) [1]*	-3.91E-03	5.44E-03	4.73E-01	-1.42E-02	1.25E-02	2.55E-01	-4.43E-02	2.92E-02	1.30E-01

Supplementary Table 2: Parameter estimates for metabolome-wide association studies for diet-metabolite associations for each of: The Healthy Eating Index-2015 (HEI-15), DASH and AMED diet

diacylglycerol (14:0/18:1, 16:0/16:1) [2]*	1.58E-03	5.68E-03	7.81E-01	-6.32E-03	1.31E-02	6.29E-01	-2.14E-02	3.06E-02	4.85E-01
oleoyl-arachidonoyl-glycerol (18:1/20:4) [1]*	1.43E-03	6.01E-03	8.13E-01	-8.74E-03	1.38E-02	5.28E-01	-5.63E-03	3.24E-02	8.62E-01
oleoyl-arachidonoyl-glycerol (18:1/20:4) [2]*	-1.36E-03	5.99E-03	8.21E-01	-1.01E-02	1.38E-02	4.62E-01	-2.04E-02	3.23E-02	5.29E-01
palmitoyl-linolenoyl-glycerol (16:0/18:3) [2]*	2.32E-03	5.35E-03	6.65E-01	2.28E-03	1.23E-02	8.53E-01	-2.04E-02	2.88E-02	4.79E-01
diacylglycerol (16:1/18:2 [2], 16:0/18:3 [1])*	2.62E-03	6.11E-03	6.68E-01	1.91E-03	1.41E-02	8.92E-01	5.00E-04	3.29E-02	9.88E-01
linoleoyl-linoleoyl-glycerol (18:2/18:2) [1]*	9.26E-04	5.95E-03	8.76E-01	1.39E-02	1.37E-02	3.12E-01	-4.42E-03	3.21E-02	8.91E-01
linoleoyl-linoleoyl-glycerol (18:2/18:2) [2]*	1.08E-02	6.02E-03	7.46E-02	2.09E-02	1.39E-02	1.33E-01	3.81E-02	3.25E-02	2.42E-01
stearoyl-arachidonoyl-glycerol (18:0/20:4) [1]*	8.86E-03	6.09E-03	1.47E-01	3.62E-05	1.41E-02	9.98E-01	5.43E-02	3.28E-02	9.93E-02
stearoyl-arachidonoyl-glycerol (18:0/20:4) [2]*	-4.14E-03	5.64E-03	4.64E-01	-2.57E-02	1.29E-02	4.76E-02	1.92E-02	3.04E-02	5.28E-01
perfluorooctanesulfonate (PFOS)	4.35E-03	5.38E-03	4.20E-01	1.66E-03	1.24E-02	8.94E-01	7.99E-03	2.90E-02	7.83E-01
1-palmityl-GPE (O-16:0)*	6.43E-03	5.58E-03	2.50E-01	-5.71E-03	1.29E-02	6.58E-01	3.33E-02	3.01E-02	2.69E-01
1-palmityl-2-stearoyl-GPC (O-16:0/18:0)*	-8.39E-03	6.09E-03	1.69E-01	-5.65E-03	1.41E-02	6.88E-01	-4.89E-02	3.28E-02	1.37E-01
1-palmityl-2-palmitoyl-GPC (O-16:0/16:0)*	-2.28E-02	5.89E-03	1.38E-04	-4.64E-02	1.36E-02	7.72E-04	-1.12E-01	3.19E-02	5.24E-04
1-stearyl-2-docosapentaenoyl-GPC (O-18:0/22:5n3)*	-1.01E-02	5.64E-03	7.53E-02	-8.47E-03	1.31E-02	5.17E-01	-4.25E-02	3.05E-02	1.64E-01
1-stearyl-2-linoleoyl-GPC (O-18:0/18:2)*	-4.94E-03	5.97E-03	4.08E-01	-3.99E-03	1.37E-02	7.72E-01	1.56E-03	3.22E-02	9.61E-01
1-stearoyl-2-docosapentaenoyl-GPE (18:0/22:5n3)*	3.40E-03	6.03E-03	5.74E-01	1.11E-02	1.39E-02	4.23E-01	3.67E-02	3.25E-02	2.60E-01
1-stearoyl-2-docosapentaenoyl-GPE (18:0/22:5n6)*	-1.26E-02	5.96E-03	3.59E-02	-3.42E-02	1.37E-02	1.29E-02	-7.70E-02	3.20E-02	1.70E-02
1-stearoyl-2-adrenoyl-GPE (18:0/22:4)*	-9.36E-03	5.83E-03	1.09E-01	-2.50E-02	1.34E-02	6.25E-02	-5.28E-02	3.14E-02	9.33E-02
1-(1-enyl-stearoyl)-2-docosapentaenoyl-GPE (P-18:0/22:5n3)*	-1.27E-02	5.60E-03	2.40E-02	-3.56E-02	1.28E-02	5.91E-03	-2.80E-02	3.04E-02	3.57E-01
N-palmitoyl-sphingadienine (d18:2/16:0)*	3.34E-03	5.90E-03	5.72E-01	2.66E-03	1.36E-02	8.45E-01	3.95E-02	3.17E-02	2.15E-01
lactosyl-N-nervonoyl-sphingosine (d18:1/24:1)*	8.96E-03	5.75E-03	1.20E-01	1.14E-02	1.33E-02	3.90E-01	6.40E-02	3.09E-02	3.91E-02
glycosyl-N-behenoyl-sphingosine (d18:1/22:0)*	3.05E-03	6.02E-03	6.13E-01	1.10E-02	1.38E-02	4.28E-01	3.44E-02	3.24E-02	2.90E-01
lactosyl-N-behenoyl-sphingosine (d18:1/22:0)*	2.31E-03	5.72E-03	6.87E-01	2.65E-02	1.31E-02	4.38E-02	-3.10E-03	3.08E-02	9.20E-01
N-behenoyl-sphingadienine (d18:2/22:0)*	3.21E-03	5.88E-03	5.85E-01	2.74E-02	1.34E-02	4.25E-02	1.27E-02	3.17E-02	6.89E-01
glycosyl-N-behenoyl-sphingadienine (d18:2/22:0)*	2.22E-03	5.87E-03	7.05E-01	-8.52E-04	1.35E-02	9.50E-01	5.44E-02	3.15E-02	8.53E-02
N-stearoyl-sphingadienine (d18:2/18:0)*	-1.52E-02	5.57E-03	6.86E-03	-3.24E-02	1.29E-02	1.23E-02	-6.49E-02	3.02E-02	3.24E-02
N-palmitoylserine	6.01E-03	5.46E-03	2.72E-01	-9.80E-04	1.26E-02	9.38E-01	-3.64E-03	2.95E-02	9.02E-01
N-oleoylserine	4.81E-03	6.18E-03	4.37E-01	1.04E-02	1.42E-02	4.67E-01	5.10E-02	3.32E-02	1.26E-01
sphingadienine	-1.08E-02	6.03E-03	7.44E-02	-1.78E-02	1.39E-02	2.03E-01	-4.60E-02	3.26E-02	1.59E-01
palmitoleoyl-arachidonoyl-glycerol (16:1/20:4) [2]*	2.29E-03	5.91E-03	6.99E-01	2.89E-03	1.36E-02	8.32E-01	7.86E-03	3.19E-02	8.05E-01
myristoyl-linoleoyl-glycerol (14:0/18:2) [2]*	5.48E-03	5.80E-03	3.45E-01	1.64E-02	1.33E-02	2.20E-01	-3.40E-03	3.13E-02	9.14E-01
phosphatidylethanolamine (P-18:1/20:4, P-16:0/22:5n3)*	-2.06E-03	5.89E-03	7.27E-01	-2.69E-02	1.35E-02	4.68E-02	2.81E-02	3.17E-02	3.75E-01
1-stearoyl-2-(hydroxylinoleoyl)-GPC (18:0/18:2(OH))*	-1.03E-02	5.77E-03	7.40E-02	-2.82E-02	1.32E-02	3.42E-02	-2.20E-02	3.12E-02	4.81E-01
1-palmitoyl-2-(hydroxylinoleoyl)-GPC (16:0/18:2(OH))*	-1.34E-02	5.79E-03	2.14E-02	-3.66E-02	1.33E-02	6.14E-03	-1.89E-02	3.15E-02	5.48E-01
hexadecasphingosine (d16:1)*	-5.99E-03	5.92E-03	3.13E-01	-1.39E-02	1.36E-02	3.08E-01	-5.89E-02	3.18E-02	6.48E-02
ceramide (d16:1/24:1, d18:1/22:1)*	-2.17E-04	5.76E-03	9.70E-01	-1.16E-02	1.32E-02	3.82E-01	-5.59E-02	3.09E-02	7.15E-02
N-palmitoyl-heptadecasphingosine (d17:1/16:0)*	-9.78E-03	5.65E-03	8.45E-02	-2.15E-02	1.30E-02	1.00E-01	-9.00E-02	3.01E-02	3.09E-03
ceramide (d18:1/14:0, d16:1/16:0)*	6.20E-03	5.96E-03	2.99E-01	1.23E-02	1.37E-02	3.72E-01	-7.66E-03	3.22E-02	8.12E-01
ceramide (d18:1/17:0, d17:1/18:0)*	-1.62E-02	5.76E-03	5.30E-03	-3.22E-02	1.33E-02	1.59E-02	-1.02E-01	3.09E-02	1.05E-03
ceramide (d18:2/24:1, d18:1/24:2)*	-2.42E-03	5.88E-03	6.81E-01	-1.27E-02	1.35E-02	3.48E-01	-1.85E-02	3.17E-02	5.60E-01
glycosyl ceramide (d18:2/24:1, d18:1/24:2)*	-3.42E-03	5.73E-03	5.51E-01	-2.12E-02	1.31E-02	1.08E-01	2.88E-02	3.09E-02	3.51E-01
glycosyl-N-tricosanoyl-sphingadienine (d18:2/23:0)*	-1.37E-03	5.72E-03	8.12E-01	-1.27E-02	1.32E-02	3.35E-01	3.76E-02	3.08E-02	2.22E-01
glycosyl ceramide (d18:1/23:1, d17:1/24:1)*	-1.33E-02	5.54E-03	1.66E-02	-3.01E-02	1.27E-02	1.89E-02	-9.34E-02	2.96E-02	1.78E-03
glycosyl-N-(2-hydroxynervonoyl)-sphingosine (d18:1/24:1(2OH))*	1.71E-03	5.52E-03	7.57E-01	-2.35E-02	1.26E-02	6.44E-02	-1.29E-02	2.97E-02	6.65E-01
ceramide (d18:1/20:0, d16:1/22:0, d20:1/18:0)*	-1.82E-04	5.92E-03	9.75E-01	1.09E-02	1.36E-02	4.23E-01	-5.11E-02	3.17E-02	1.09E-01
stearoylcholine*	1.40E-02	5.92E-03	1.87E-02	4.13E-02	1.35E-02	2.48E-03	8.70E-02	3.18E-02	6.62E-03
linoleoylcholine*	1.72E-02	5.81E-03	3.41E-03	4.46E-02	1.33E-02	9.33E-04	1.04E-01	3.12E-02	9.74E-04
1-adrenoyl-GPE (22:4)*	-1.59E-02	5.75E-03	6.06E-03	-2.37E-02	1.34E-02	7.69E-02	-9.17E-02	3.10E-02	3.30E-03
1-docosapentaenoyl-GPE (22:5n3)*	-1.37E-02	5.90E-03	2.06E-02	-2.01E-02	1.36E-02	1.43E-01	-3.90E-02	3.20E-02	2.24E-01
trazadone	-4.18E-04	2.27E-03	8.54E-01	-4.87E-03	5.22E-03	3.52E-01	-1.77E-02	1.22E-02	1.49E-01
nisinate (24:6n3)	9.84E-03	5.21E-03	6.00E-02	1.10E-03	1.21E-02	9.28E-01	6.44E-02	2.80E-02	2.23E-02
sphingomyelin (d18:0/20:0, d16:0/22:0)*	-9.14E-03	6.04E-03	1.32E-01	-4.88E-02	1.37E-02	4.13E-04	-3.63E-02	3.26E-02	2.67E-01
sphingomyelin (d18:1/19:0, d19:1/18:0)*	-1.50E-02	5.46E-03	6.34E-03	-3.59E-02	1.25E-02	4.48E-03	-8.38E-02	2.94E-02	4.66E-03
sphingomyelin (d18:2/18:1)*	-2.11E-02	5.37E-03	1.09E-04	-3.59E-02	1.25E-02	4.44E-03	-7.68E-02	2.94E-02	9.40E-03
sphingomyelin (d18:2/24:2)*	-1.49E-02	5.44E-03	6.54E-03	-3.63E-02	1.25E-02	4.00E-03	-4.92E-02	2.95E-02	9.73E-02
sphingomyelin (d18:2/21:0, d16:2/23:0)*	-1.07E-02	4.95E-03	3.17E-02	-2.02E-02	1.14E-02	7.83E-02	-4.87E-02	2.68E-02	7.00E-02
sphingomyelin (d18:2/23:1)*	-1.73E-02	5.22E-03	1.05E-03	-4.86E-02	1.19E-02	5.90E-05	-9.34E-02	2.81E-02	1.02E-03
sphingomyelin (d18:1/25:0, d19:0/24:1, d20:1/23:0, d19:1/24:0)*	-1.04E-02	5.83E-03	7.55E-02	-2.61E-02	1.34E-02	5.29E-02	-6.28E-02	3.14E-02	4.64E-02
sphingomyelin (d17:2/16:0, d18:2/15:0)*	-8.94E-03	5.15E-03	8.41E-02	-1.82E-02	1.19E-02	1.26E-01	-3.95E-02	2.78E-02	1.57E-01
heneicosapentaenoate (21:5n3)	9.73E-03	4.82E-03	4.45E-02	-7.50E-03	1.12E-02	5.03E-01	7.33E-02	2.58E-02	4.82E-03
linolenoylcarnitine (C18:3)*	-8.21E-03	5.94E-03	1.68E-01	-1.09E-02	1.37E-02	4.28E-01	-5.70E-03	3.21E-02	8.59E-01
behenoylcarnitine (C22)*	1.10E-02	6.18E-03	7.62E-02	1.74E-02	1.43E-02	2.23E-01	5.68E-02	3.33E-02	8.94E-02
arachidoylcarnitine (C20)*	9.42E-03	5.99E-03	1.17E-01	2.61E-02	1.38E-02	5.84E-02	4.73E-02	3.23E-02	1.44E-01
lignoceroylcarnitine (C24)*	1.13E-02	6.18E-03	6.82E-02	2.32E-02	1.42E-02	1.04E-01	6.35E-02	3.33E-02	5.77E-02
cerotoylcarnitine (C26)*	1.65E-02	5.93E-03	5.63E-03	3.41E-02	1.37E-02	1.34E-02	7.42E-02	3.21E-02	2.15E-02
ximenoylcarnitine (C26:1)*	1.45E-02	5.88E-03	1.42E-02	6.91E-03	1.37E-02	6.14E-01	7.63E-02	3.17E-02	1.68E-02
arachidonoylcarnitine (C20:4)	2.85E-03	5.64E-03	6.14E-01	6.52E-03	1.30E-02	6.16E-01	2.62E-02	3.04E-02	3.89E-01
eicosenoylcarnitine (C20:1)*	5.32E-03	5.63E-03	3.45E-01	6.48E-03	1.30E-02	6.18E-01	2.61E-02	3.03E-02	3.90E-01
dihomo-linoleoylcarnitine (C20:2)*	-8.88E-04	5.73E-03	8.77E-01	2.56E-03	1.32E-02	8.46E-01	2.02E-02	3.09E-02	5.13E-01
dihomo-linolenoylcarnitine (C20:3n3 or 6)*	4.08E-03	5.73E-03	4.77E-01	9.68E-03	1.32E-02	4.64E-01	7.80E-03	3.09E-02	8.01E-01
erucoylcarnitine (C22:1)*	4.65E-03	5.39E-03	3.89E-01	5.82E-03	1.24E-02	6.39E-01	1.50E-02	2.91E-02	6.07E-01
nervonoylcarnitine (C24:1)*	3.16E-03	5.89E-03	5.92E-01	-1.35E-02	1.35E-02	3.19E-01	2.92E-02	3.17E-02	3.57E-01
adrenoylcarnitine (C22:4)*	-1.81E-03	5.13E-03	7.24E-01	9.61E-03	1.18E-02	4.16E-01	-1.07E-03	2.77E-02	9.69E-01
glycosyl ceramide (d18:1/20:0, d16:1/22:0)*	-1.08E-03	5.66E-03	8.49E-01	-9.47E-03	1.30E-02	4.68E-01	1.54E-02	3.05E-02	6.14E-01
1-lignoceroyl-2-arachidonoyl-GPC (24:0/20:4)*	2.46E-02	5.88E-03	3.80E-05	4.00E-02	1.37E-02	3.92E-03	1.31E-01	3.17E-02	4.83E-05
1-nervonoyl-2-arachidonoyl-GPC (24:1/20:4)*	1.00E-02	5.97E-03	9.46E-02	-1.57E-02	1.38E-02	2.57E-01	7.17E-02	3.21E-02	2.62E-02
N-oxalyl glycine (NOG)	1.01E-02	5.59E-03	7.13E-02	2.65E-02	1.29E-02	4.04E-02	5.95E-02	3.01E-02	4.90E-02
N,N,N-trimethyl-5-aminovalerate	-1.95E-03	5.85E-03	7.39E-01	9.11E-03	1.35E-02	4.99E-01	-3.27E-02	3.15E-02	3.00E-01
ethyl alpha-glucopyranoside	-2.10E-04	5.05E-03	9.67E-01	-5.68E-02	1.11E-02	6.19E-07	-1.22E-03	2.72E-02	9.64E-01
carotene diol (1)	2.68E-02	5.58E-03	2.58E-06	3.46E-02	1.32E-02	9.31E-03	1.47E-01	3.00E-02	1.68E-06
carotene diol (2)	2.64E-02	5.48E-03	2.38E-06	3.94E-02	1.29E-02	2.48E-03	1.33E-01	2.97E-02	1.15E-05
carotene diol (3)	1.45E-02	5.91E-03	1.49E-02	2.10E-02	1.37E-02	1.27E-01	8.13E-02	3.18E-02	1.13E-02
cortolone glucuronide (1)	-1.86E-02	5.91E-03	1.84E-03	-2.76E-02	1.37E-02	4.56E-02	-7.23E-02	3.21E-02	2.51E-02
fluconazole	4.03E-04	1.06E-03	7.04E-01	-1.12E-04	2.44E-03	9.63E-01	-5.61E-04	5.71E-03	9.22E-01

Fibrinopeptide B	4.68E-03	3.94E-03	2.36E-01	8.59E-03	9.07E-03	3.45E-01	1.93E-02	2.13E-02	3.66E-01
1-oleyl-2-linoleoyl-GPC (O-18:1/18:2)*	3.57E-03	5.46E-03	5.14E-01	1.02E-02	1.26E-02	4.17E-01	3.98E-02	2.94E-02	1.77E-01
1-palmityl-2-dihomo-linolenoyl-GPC (O-16:0/20:3)*	-5.08E-03	6.14E-03	4.09E-01	-2.95E-02	1.41E-02	3.65E-02	-4.12E-02	3.31E-02	2.14E-01
phosphatidylcholine (O-18:1/20:4, O-16:0/22:5n3)*	-1.98E-03	5.83E-03	7.34E-01	-1.79E-02	1.34E-02	1.81E-01	1.83E-02	3.14E-02	5.61E-01
1-palmitoyl-2-pentadecanoyl-GPC (16:0/15:0)*	-9.28E-03	5.60E-03	9.87E-02	-3.93E-03	1.30E-02	7.62E-01	-7.56E-02	3.00E-02	1.22E-02
(N(1) + N(8))-acetylspermidine	2.16E-03	5.71E-03	7.05E-01	9.04E-03	1.31E-02	4.92E-01	2.72E-02	3.07E-02	3.76E-01
THC carboxylic acid	2.09E-03	2.78E-03	4.52E-01	-5.75E-03	6.39E-03	3.69E-01	1.28E-02	1.50E-02	3.95E-01
THC carboxylic acid glucuronide	2.21E-03	2.71E-03	4.15E-01	-4.65E-03	6.23E-03	4.56E-01	1.19E-02	1.46E-02	4.15E-01
5-dodecenoylcarnitine (C12:1)	-2.13E-02	5.69E-03	2.26E-04	-5.57E-02	1.30E-02	2.48E-05	-7.56E-02	3.11E-02	1.56E-02
2-butenoylglycine	-1.05E-03	5.14E-03	8.38E-01	8.83E-03	1.18E-02	4.56E-01	3.14E-02	2.76E-02	2.57E-01
hydroxy-CMPF*	1.64E-02	5.77E-03	4.87E-03	1.97E-02	1.34E-02	1.43E-01	1.21E-01	3.07E-02	9.96E-05
3-hydroxyoleoylcarnitine	-2.01E-03	5.58E-03	7.19E-01	-9.01E-03	1.28E-02	4.83E-01	-1.59E-02	3.01E-02	5.97E-01
3-hydroxyphenylacetoylglutamine	1.58E-02	5.58E-03	5.01E-03	3.56E-02	1.29E-02	5.97E-03	7.42E-02	3.02E-02	1.46E-02
trans-2-hexenoylglycine	-1.06E-02	5.21E-03	4.25E-02	-1.03E-02	1.21E-02	3.93E-01	-2.28E-02	2.83E-02	4.19E-01
2-hydroxyarachidate*	-8.87E-03	5.83E-03	1.29E-01	-2.94E-02	1.34E-02	2.89E-02	-5.94E-02	3.14E-02	5.95E-02
N-stearoylserine*	-5.29E-03	6.03E-03	3.81E-01	-1.66E-02	1.39E-02	2.32E-01	1.42E-02	3.26E-02	6.63E-01
lyxonate	1.15E-02	5.60E-03	4.07E-02	2.94E-03	1.30E-02	8.21E-01	2.52E-02	3.04E-02	4.07E-01
dodecenedioate (C12:1-DC)*	6.91E-03	5.78E-03	2.33E-01	1.15E-02	1.33E-02	3.90E-01	1.96E-02	3.12E-02	5.30E-01
hexadecenedioate (C16:1-DC)*	-1.05E-02	5.86E-03	7.46E-02	-2.33E-02	1.35E-02	8.49E-02	-5.80E-02	3.16E-02	6.75E-02
octadecenedioate (C18:1-DC)	3.98E-03	6.07E-03	5.12E-01	2.20E-02	1.39E-02	1.14E-01	2.26E-02	3.27E-02	4.90E-01
heptenedioate (C7:1-DC)*	-2.80E-02	5.66E-03	1.25E-06	-2.27E-02	1.35E-02	9.38E-02	-1.40E-01	3.07E-02	7.40E-06
octadecadienedioate (C18:2-DC)*	6.64E-03	6.32E-03	2.94E-01	9.49E-03	1.46E-02	5.16E-01	3.71E-02	3.41E-02	2.78E-01
glucuronide of C12H22O4 (1)*	8.93E-03	4.58E-03	5.19E-02	3.52E-02	1.04E-02	8.15E-04	1.38E-02	2.48E-02	5.79E-01
glucuronide of C10H18O2 (1)*	2.34E-05	4.92E-03	9.96E-01	1.39E-03	1.13E-02	9.02E-01	1.76E-02	2.65E-02	5.07E-01
3-carboxy-4-methyl-5-pentyl-2-furanpropionate (3-CMPFP)**	-6.94E-03	5.57E-03	2.14E-01	-4.11E-03	1.29E-02	7.49E-01	-5.35E-03	3.01E-02	8.59E-01
methylnaphthyl sulfate (1)*	1.65E-03	3.00E-03	5.81E-01	-3.91E-03	6.89E-03	5.71E-01	-2.50E-02	1.61E-02	1.22E-01
methylnaphthyl sulfate (2)*	-2.75E-03	3.61E-03	4.46E-01	-1.47E-02	8.27E-03	7.68E-02	2.12E-03	1.95E-02	9.13E-01
glucuronide of C10H18O2 (7)*	-5.07E-04	5.77E-03	9.30E-01	-3.18E-03	1.33E-02	8.11E-01	4.65E-03	3.11E-02	8.81E-01
glucuronide of C10H18O2 (8)*	2.67E-03	4.93E-03	5.89E-01	-4.61E-03	1.13E-02	6.85E-01	1.28E-02	2.66E-02	6.30E-01
N-acetyl-2-aminooctanoate*	3.09E-03	5.86E-03	5.98E-01	1.53E-02	1.35E-02	2.58E-01	2.49E-02	3.16E-02	4.31E-01
hydroxyasparagine**	-1.09E-02	5.74E-03	5.90E-02	-1.71E-03	1.33E-02	8.98E-01	-7.95E-02	3.08E-02	1.04E-02
perfluorooctanoate (PFOA)	-2.31E-03	5.31E-03	6.64E-01	-1.61E-02	1.22E-02	1.88E-01	-4.82E-03	2.86E-02	8.66E-01
pyroglutamylphenylalanine	1.59E-03	5.49E-03	7.72E-01	-4.73E-03	1.26E-02	7.08E-01	9.48E-03	2.96E-02	7.49E-01
3-hydroxybutyroylglycine**	-8.93E-03	5.45E-03	1.02E-01	-8.91E-03	1.26E-02	4.79E-01	2.66E-03	2.95E-02	9.28E-01
carboxyibuprofen glucuronide*	2.82E-03	2.99E-03	3.46E-01	-5.00E-03	6.88E-03	4.68E-01	-2.47E-03	1.61E-02	8.78E-01
glyco-beta-muricholate**	1.19E-02	5.99E-03	4.82E-02	2.88E-02	1.38E-02	3.71E-02	1.69E-02	3.25E-02	6.04E-01
N-methylhydroxyproline**	1.30E-02	5.17E-03	1.25E-02	2.60E-02	1.19E-02	2.97E-02	4.29E-02	2.80E-02	1.27E-01
N,N,N-trimethyl-alanylproline betaine (TMAP)	-2.88E-03	5.56E-03	6.05E-01	2.68E-02	1.27E-02	3.60E-02	-4.35E-02	2.99E-02	1.46E-01
3-formylindole	1.45E-02	5.40E-03	7.68E-03	3.58E-02	1.24E-02	4.19E-03	6.44E-02	2.92E-02	2.84E-02
1-(1-enyl-oleoyl)-2-docosahexaenoyl-GPC (P-18:1/22:6)*	1.90E-02	5.21E-03	3.07E-04	1.05E-02	1.23E-02	3.91E-01	1.38E-01	2.75E-02	9.68E-07
pyroglutamylalanine*	-3.69E-03	5.91E-03	5.33E-01	7.36E-03	1.36E-02	5.89E-01	-4.94E-02	3.17E-02	1.20E-01
pyroglutamylproline*	-1.39E-02	5.98E-03	2.06E-02	-2.58E-02	1.38E-02	6.31E-02	-5.39E-02	3.24E-02	9.71E-02
pyroglutamylleucine*	-3.18E-03	6.01E-03	5.97E-01	7.40E-03	1.38E-02	5.93E-01	-6.48E-02	3.22E-02	4.48E-02
pyroglutamylisoleucine*	-1.52E-04	5.73E-03	9.79E-01	1.72E-03	1.32E-02	8.96E-01	-3.26E-02	3.08E-02	2.91E-01
gamma-glutamylcitrulline*	-9.47E-03	5.86E-03	1.07E-01	-1.54E-02	1.35E-02	2.57E-01	-3.43E-02	3.17E-02	2.80E-01
resveratrol disulfate (1)*	1.46E-03	3.15E-03	6.43E-01	-1.05E-02	7.22E-03	1.45E-01	7.43E-03	1.70E-02	6.62E-01
resveratrol disulfate (2)*	1.10E-03	3.62E-03	7.62E-01	-2.08E-02	8.24E-03	1.23E-02	4.61E-03	1.95E-02	8.14E-01
glycine conjugate of C10H12O2*	-1.28E-02	5.72E-03	2.58E-02	-3.28E-02	1.31E-02	1.30E-02	-8.06E-02	3.07E-02	9.21E-03
glycine conjugate of C10H14O2 (1)*	-2.84E-02	5.99E-03	3.35E-06	-5.97E-02	1.39E-02	2.36E-05	-1.35E-01	3.26E-02	4.41E-05
glutamine conjugate of C7H12O2*	-1.90E-02	5.68E-03	9.19E-04	-2.09E-02	1.33E-02	1.16E-01	-8.09E-02	3.08E-02	9.19E-03
glutamine conjugate of C6H10O2 (1)*	-1.13E-02	5.80E-03	5.19E-02	-8.67E-03	1.34E-02	5.19E-01	-3.86E-02	3.14E-02	2.19E-01
glutamine conjugate of C6H10O2 (2)*	-1.25E-02	5.59E-03	2.65E-02	-1.84E-02	1.29E-02	1.56E-01	-4.37E-02	3.03E-02	1.51E-01
dihydroferulic acid sulfate	7.82E-03	5.07E-03	1.24E-01	2.75E-02	1.16E-02	1.86E-02	1.73E-02	2.74E-02	5.29E-01
sphingomyelin (d17:1/14:0, d16:1/15:0)*	-2.97E-03	5.20E-03	5.68E-01	-2.92E-03	1.20E-02	8.08E-01	-2.75E-02	2.80E-02	3.27E-01
tetradecadienoate (14:2)*	-1.36E-02	5.86E-03	2.10E-02	-3.65E-02	1.34E-02	7.03E-03	-4.68E-02	3.18E-02	1.41E-01
8-methoxykynurenate	-8.98E-03	6.03E-03	1.38E-01	-2.10E-02	1.39E-02	1.32E-01	-5.82E-02	3.25E-02	7.38E-02
3-amino-2-piperidone	2.18E-03	5.54E-03	6.94E-01	2.45E-02	1.27E-02	5.38E-02	2.12E-02	2.98E-02	4.78E-01
N,N-dimethylalanine	-8.66E-04	5.66E-03	8.79E-01	-3.18E-03	1.30E-02	8.08E-01	1.59E-02	3.05E-02	6.03E-01
3-indoleglyoxylic acid	1.52E-02	5.71E-03	8.12E-03	2.99E-02	1.32E-02	2.40E-02	4.76E-02	3.10E-02	1.26E-01
ethyl beta-glucopyranoside	1.33E-02	5.75E-03	2.12E-02	-3.22E-03	1.34E-02	8.10E-01	6.03E-02	3.11E-02	5.31E-02
2-hydroxysebacate	1.39E-02	5.83E-03	1.77E-02	3.83E-02	1.34E-02	4.46E-03	5.17E-02	3.16E-02	1.03E-01
enterolactone sulfate	1.12E-02	4.72E-03	1.80E-02	1.12E-02	1.10E-02	3.10E-01	4.57E-02	2.56E-02	7.51E-02
ascorbic acid 3-sulfate*	1.83E-02	5.91E-03	2.17E-03	5.23E-02	1.35E-02	1.33E-04	5.09E-02	3.23E-02	1.16E-01
3-hydroxyhippurate sulfate	8.22E-03	5.46E-03	1.34E-01	2.44E-02	1.25E-02	5.25E-02	5.46E-02	2.94E-02	6.43E-02
6-bromotryptophan	1.31E-03	5.96E-03	8.26E-01	9.25E-04	1.37E-02	9.46E-01	-3.05E-02	3.21E-02	3.43E-01
delta-CEHC	-2.44E-02	5.96E-03	5.61E-05	-2.20E-02	1.41E-02	1.20E-01	-9.24E-02	3.26E-02	4.97E-03
N6,N6-dimethyllysine	-5.85E-03	5.25E-03	2.66E-01	-8.07E-03	1.21E-02	5.05E-01	-5.31E-03	2.83E-02	8.51E-01
1-carboxyethylphenylalanine	-6.61E-03	5.44E-03	2.25E-01	1.18E-02	1.25E-02	3.46E-01	-3.90E-02	2.93E-02	1.85E-01
1-carboxyethyltyrosine	-6.89E-03	5.57E-03	2.17E-01	6.72E-03	1.28E-02	6.01E-01	-3.38E-02	3.00E-02	2.62E-01
1-carboxyethylvaline	-3.64E-03	5.56E-03	5.13E-01	1.92E-02	1.28E-02	1.34E-01	-1.49E-02	3.00E-02	6.19E-01
1-carboxyethylleucine	-5.59E-03	5.40E-03	3.01E-01	-5.01E-03	1.24E-02	6.88E-01	-3.32E-02	2.91E-02	2.55E-01
1-carboxyethylisoleucine	-5.58E-03	5.41E-03	3.03E-01	1.19E-02	1.24E-02	3.38E-01	-2.58E-02	2.92E-02	3.78E-01
dodecadienoate (12:2)*	-3.90E-03	5.81E-03	5.03E-01	-1.48E-02	1.34E-02	2.70E-01	-1.40E-02	3.13E-02	6.55E-01
indoleacetoylcarnitine*	-6.28E-03	5.29E-03	2.36E-01	-3.75E-02	1.20E-02	1.97E-03	2.18E-02	2.85E-02	4.46E-01
gamma-CEHC sulfate	-4.06E-03	4.89E-03	4.07E-01	1.06E-02	1.12E-02	3.48E-01	-3.77E-02	2.63E-02	1.52E-01
delta-CEHC glucuronide	-1.73E-02	5.68E-03	2.52E-03	-5.78E-03	1.33E-02	6.64E-01	-6.70E-02	3.08E-02	3.05E-02
N-acetyl-isoputreanine	9.26E-03	5.95E-03	1.21E-01	9.61E-03	1.37E-02	4.85E-01	5.86E-02	3.20E-02	6.84E-02
glucuronide of piperine metabolite C17H21NO3 (3)*	5.86E-03	5.69E-03	3.04E-01	-1.88E-02	1.31E-02	1.52E-01	4.05E-02	3.06E-02	1.87E-01
glucuronide of piperine metabolite C17H21NO3 (4)*	3.99E-03	5.68E-03	4.82E-01	-2.19E-02	1.30E-02	9.32E-02	4.27E-02	3.05E-02	1.63E-01
glucuronide of piperine metabolite C17H21NO3 (5)*	4.01E-03	5.79E-03	4.89E-01	-2.63E-02	1.32E-02	4.77E-02	3.80E-02	3.11E-02	2.23E-01
sulfate of piperine metabolite C16H19NO3 (2)*	4.53E-03	5.89E-03	4.43E-01	-2.90E-02	1.35E-02	3.22E-02	4.17E-02	3.17E-02	1.89E-01
sulfate of piperine metabolite C16H19NO3 (3)*	4.25E-03	5.83E-03	4.67E-01	-2.64E-02	1.33E-02	4.86E-02	3.94E-02	3.14E-02	2.10E-01
sulfate of piperine metabolite C18H21NO3 (1)*	5.14E-04	5.66E-03	9.28E-01	-2.66E-02	1.29E-02	4.03E-02	2.15E-02	3.05E-02	4.82E-01
5-hydroxyindole glucuronide	9.57E-04	5.75E-03	8.68E-01	5.73E-03	1.32E-02	6.65E-01	6.61E-03	3.10E-02	8.31E-01

Supplementary Table 2: Parameter estimates for metabolome-wide association studies for diet-metabolite associations for each of: The Healthy Eating Index-2015 (HEI-15), DASH and AMED diet

N-acetylhomocitrulline	1.15E-03	4.61E-03	8.03E-01	-4.01E-03	1.06E-02	7.06E-01	-1.52E-02	2.49E-02	5.40E-01
2-naphthol sulfate	-4.71E-03	5.33E-03	3.78E-01	-1.83E-02	1.22E-02	1.36E-01	-3.28E-02	2.87E-02	2.54E-01
(2,4 or 2,5)-dimethylphenol sulfate	-6.03E-03	4.72E-03	2.03E-01	-3.26E-02	1.07E-02	2.61E-03	-2.33E-03	2.55E-02	9.27E-01
4-ethylcatechol sulfate	1.19E-02	5.81E-03	4.14E-02	2.99E-02	1.33E-02	2.59E-02	3.67E-02	3.15E-02	2.44E-01
11beta-hydroxyandrosterone glucuronide	-5.66E-03	6.03E-03	3.49E-01	6.21E-03	1.39E-02	6.56E-01	-5.42E-02	3.24E-02	9.52E-02
11beta-hydroxyetiocholanolone glucuronide*	5.85E-03	5.73E-03	3.09E-01	6.50E-03	1.32E-02	6.23E-01	-2.09E-02	3.09E-02	5.01E-01
N2-acetyl,N6-methyllysine	-6.99E-03	5.73E-03	2.24E-01	-2.21E-02	1.32E-02	9.49E-02	4.12E-04	3.10E-02	9.89E-01
cholic acid glucuronide	6.21E-03	5.25E-03	2.38E-01	1.27E-02	1.21E-02	2.96E-01	4.44E-02	2.83E-02	1.17E-01
deoxycholic acid glucuronide	2.03E-04	6.07E-03	9.73E-01	-1.18E-02	1.40E-02	4.00E-01	4.11E-02	3.26E-02	2.09E-01
4-allylcatechol sulfate	1.34E-02	6.18E-03	3.08E-02	2.86E-02	1.43E-02	4.57E-02	7.01E-02	3.33E-02	3.64E-02
2-hydroxyfluorene sulfate	-1.79E-03	4.07E-03	6.60E-01	-1.61E-02	9.32E-03	8.59E-02	9.41E-04	2.19E-02	9.66E-01
vanillate glucuronide	2.87E-03	2.99E-03	3.39E-01	1.18E-02	6.86E-03	8.52E-02	6.29E-04	1.61E-02	9.69E-01
4-methylhexanoylglutamine	-2.17E-02	5.62E-03	1.40E-04	-4.45E-02	1.30E-02	7.21E-04	-1.30E-01	3.01E-02	2.26E-05
Benazeprilat	2.10E-03	1.40E-03	1.35E-01	2.24E-03	3.23E-03	4.89E-01	-9.35E-03	7.56E-03	2.17E-01
1-nonadecenoyl-GPC (19:1)*	1.18E-02	5.37E-03	2.86E-02	1.89E-02	1.24E-02	1.28E-01	3.43E-02	2.91E-02	2.41E-01
desmethylcitalopram*	-5.63E-03	2.84E-03	4.83E-02	-2.28E-03	6.58E-03	7.29E-01	-4.31E-03	1.54E-02	7.80E-01
citalopram propionate*	-3.06E-03	2.69E-03	2.57E-01	-1.60E-03	6.21E-03	7.97E-01	-4.21E-05	1.45E-02	9.98E-01
4-hydroxy duloxetine glucuronide*	6.60E-20	1.01E-19	5.12E-01	5.38E-19	2.29E-19	1.98E-02	3.95E-19	5.42E-19	4.67E-01
5-hydroxy-6-methoxy duloxetine sulfate*	-4.34E-04	1.07E-03	6.84E-01	-5.44E-04	2.45E-03	8.25E-01	-1.97E-03	5.74E-03	7.32E-01
N-desalkylquetiapine*	6.98E-04	1.03E-03	4.99E-01	-1.28E-03	2.38E-03	5.90E-01	-6.11E-03	5.55E-03	2.72E-01
ranitidine N-oxide*	-1.11E-03	1.04E-03	2.87E-01	-6.06E-04	2.41E-03	8.02E-01	-3.83E-04	5.64E-03	9.46E-01
7-hydroxywarfarin	7.63E-04	1.65E-03	6.45E-01	3.72E-03	3.80E-03	3.29E-01	-4.44E-03	8.91E-03	6.19E-01
glycoursodeoxycholic acid sulfate (1)	-1.71E-02	5.29E-03	1.36E-03	-3.76E-02	1.22E-02	2.26E-03	-8.36E-02	2.86E-02	3.74E-03
dihydrocaffeate sulfate (2)	2.03E-02	5.77E-03	4.95E-04	4.17E-02	1.33E-02	1.95E-03	9.78E-02	3.12E-02	1.93E-03
lithocholate sulfate (1)	5.96E-03	5.97E-03	3.19E-01	2.28E-02	1.37E-02	9.69E-02	-6.06E-02	3.20E-02	5.95E-02
3-hydroxyhexanoylcarnitine (1)	-4.15E-03	5.65E-03	4.64E-01	7.07E-03	1.30E-02	5.87E-01	-7.70E-03	3.05E-02	8.01E-01
3-(methylthio)acetaminophen sulfate*	4.46E-03	4.30E-03	3.00E-01	1.90E-02	9.85E-03	5.48E-02	2.45E-02	2.32E-02	2.91E-01
2-ketocaprylate	1.51E-02	5.74E-03	8.93E-03	3.36E-02	1.32E-02	1.15E-02	4.41E-02	3.12E-02	1.59E-01
2,6-dihydroxybenzoic acid	2.24E-02	5.63E-03	8.61E-05	6.97E-02	1.26E-02	8.04E-08	9.45E-02	3.07E-02	2.25E-03
tetrahydrocortisol sulfate (1)	-1.09E-02	4.94E-03	2.86E-02	-9.84E-03	1.14E-02	3.90E-01	-7.43E-02	2.65E-02	5.31E-03
3-ethylcatechol sulfate (1)	-6.25E-03	5.47E-03	2.54E-01	-3.55E-02	1.24E-02	4.60E-03	-1.50E-02	2.95E-02	6.12E-01
3-ethylcatechol sulfate (2)	-1.46E-03	4.14E-03	7.24E-01	-2.01E-03	9.54E-03	8.33E-01	-3.72E-03	2.23E-02	8.68E-01
4-acetylcatechol sulfate (1)	1.54E-02	5.50E-03	5.64E-03	4.22E-02	1.26E-02	9.12E-04	6.51E-02	2.98E-02	2.97E-02
1-(14 or 15-methyl)palmitoyl-GPC (a17:0 or i17:0)*	-5.65E-03	5.72E-03	3.25E-01	-3.53E-03	1.32E-02	7.89E-01	-6.27E-02	3.07E-02	4.19E-02
montelukast	-1.46E-03	1.85E-03	4.30E-01	2.55E-03	4.27E-03	5.51E-01	-1.42E-02	9.96E-03	1.55E-01
2,3-dihydroxy-5-methylthio-4-pentenoate (DMTPA)*	-1.09E-02	5.51E-03	4.87E-02	8.05E-03	1.28E-02	5.28E-01	-8.21E-02	2.95E-02	5.71E-03
Fibrinopeptide A (3-16)**	5.19E-03	3.85E-03	1.79E-01	1.18E-02	8.87E-03	1.83E-01	1.44E-02	2.08E-02	4.88E-01
meloxicam	-2.61E-03	1.86E-03	1.61E-01	1.10E-03	4.29E-03	7.98E-01	-6.50E-03	1.00E-02	5.18E-01
Fibrinopeptide B (1-13)**	5.17E-03	4.04E-03	2.01E-01	1.39E-02	9.28E-03	1.34E-01	7.28E-03	2.18E-02	7.39E-01
losartan	-5.11E-03	2.86E-03	7.53E-02	-5.44E-03	6.62E-03	4.12E-01	-1.60E-02	1.55E-02	3.01E-01
cyclobenzaprine	-1.86E-03	2.15E-03	3.88E-01	-6.16E-03	4.94E-03	2.13E-01	-3.92E-03	1.16E-02	7.35E-01
emtricitabine	-7.81E-05	1.66E-03	9.63E-01	-4.13E-03	3.82E-03	2.81E-01	4.68E-03	8.96E-03	6.01E-01
darunavir	-3.29E-04	1.42E-03	8.16E-01	-3.61E-03	3.25E-03	2.68E-01	1.01E-03	7.63E-03	8.95E-01
5-hydroxy-2-methylpyridine sulfate	5.25E-04	4.83E-03	9.14E-01	-2.95E-03	1.11E-02	7.91E-01	-3.64E-02	2.60E-02	1.61E-01
3-hydroxy-2-methylpyridine sulfate	8.98E-03	5.26E-03	8.89E-02	8.48E-03	1.22E-02	4.86E-01	5.45E-02	2.83E-02	5.52E-02
hydroxypalmitoyl sphingomyelin (d18:1/16:0(OH))**	2.90E-03	5.92E-03	6.24E-01	9.65E-03	1.36E-02	4.79E-01	1.44E-02	3.19E-02	6.53E-01
tamoxifen	6.60E-20	1.01E-19	5.12E-01	5.38E-19	2.29E-19	1.98E-02	3.95E-19	5.42E-19	4.67E-01
taurochenodeoxycholic acid 3-sulfate	-6.67E-03	5.41E-03	2.19E-01	-1.57E-02	1.25E-02	2.09E-01	-3.93E-02	2.92E-02	1.79E-01
tetradecadienedioate (C14:2-DC)*	-4.72E-03	5.69E-03	4.08E-01	-1.62E-02	1.31E-02	2.18E-01	-4.26E-02	3.06E-02	1.65E-01
pregnenetriol sulfate*	-1.16E-02	5.42E-03	3.30E-02	-2.38E-02	1.25E-02	5.81E-02	-8.73E-02	2.90E-02	2.82E-03
pregnenetriol disulfate*	-1.73E-02	5.46E-03	1.70E-03	-3.78E-02	1.26E-02	2.91E-03	-9.69E-02	2.94E-02	1.10E-03
eicosenedioate (C20:1-DC)*	-2.08E-02	5.90E-03	5.01E-04	-3.65E-02	1.37E-02	8.22E-03	-9.14E-02	3.20E-02	4.66E-03
hydroxy-N6,N6,N6-trimethyllysine*	-1.48E-02	5.34E-03	5.75E-03	-9.73E-03	1.24E-02	4.35E-01	-6.33E-02	2.89E-02	2.93E-02
undecenoylcarnitine (C11:1)	-1.24E-02	5.42E-03	2.32E-02	-1.51E-02	1.25E-02	2.31E-01	-7.30E-02	2.91E-02	1.28E-02
3-decenoylcarnitine	-1.42E-03	5.85E-03	8.08E-01	6.26E-03	1.35E-02	6.42E-01	-2.07E-02	3.15E-02	5.13E-01
3-hydroxydecanoylcarnitine	-1.29E-03	5.68E-03	8.21E-01	1.01E-02	1.31E-02	4.40E-01	-4.13E-03	3.06E-02	8.93E-01
palmitoyl-sphingosine-phosphoethanolamine (d18:1/16:0)	-6.54E-03	5.88E-03	2.68E-01	-1.52E-02	1.35E-02	2.61E-01	5.62E-03	3.18E-02	8.60E-01
picolinoylglycine	3.10E-03	5.64E-03	5.84E-01	2.65E-02	1.29E-02	4.08E-02	-3.63E-03	3.04E-02	9.05E-01
4-vinylcatechol sulfate	1.21E-02	5.30E-03	2.35E-02	1.37E-02	1.23E-02	2.64E-01	4.55E-02	2.87E-02	1.14E-01
succinoyltaurine	1.47E-03	6.06E-03	8.08E-01	2.33E-02	1.39E-02	9.47E-02	-1.28E-02	3.26E-02	6.95E-01
phenylalanylhydroxyproline*	-1.10E-02	5.83E-03	6.12E-02	-1.06E-02	1.35E-02	4.32E-01	4.32E-03	3.16E-02	8.91E-01
ginkgolic acid C15:1	-6.04E-05	2.93E-03	9.84E-01	1.93E-03	6.75E-03	7.75E-01	-1.16E-02	1.58E-02	4.64E-01
(15:3)-anacardic acid	3.40E-03	3.88E-03	3.81E-01	6.23E-03	8.93E-03	4.86E-01	3.95E-03	2.09E-02	8.50E-01
ginkgolic acid C17:1	2.15E-03	2.46E-03	3.83E-01	7.72E-03	5.65E-03	1.73E-01	6.48E-04	1.33E-02	9.61E-01
3,5-dichloro-2,6-dihydroxybenzoic acid	-2.81E-02	5.86E-03	2.70E-06	-3.77E-02	1.38E-02	6.88E-03	-1.40E-01	3.18E-02	1.44E-05
metabolonic lactone sulfate	-1.42E-02	5.60E-03	1.18E-02	-3.14E-02	1.29E-02	1.58E-02	-8.41E-02	3.01E-02	5.62E-03
vanillic acid glycine	1.00E-02	5.43E-03	6.51E-02	3.96E-02	1.23E-02	1.47E-03	5.84E-02	2.92E-02	4.65E-02
2-hydroxy-4-(methylthio)butanoic acid	-1.36E-02	5.17E-03	9.12E-03	-2.29E-02	1.20E-02	5.71E-02	-1.13E-02	2.82E-02	6.88E-01
branched chain 14:0 dicarboxylic acid**	3.62E-02	5.35E-03	7.45E-11	6.66E-02	1.27E-02	2.99E-07	1.36E-01	3.00E-02	8.37E-06
(2-butoxyethoxy)acetic acid	8.60E-04	4.65E-03	8.53E-01	1.01E-02	1.07E-02	3.45E-01	-2.78E-02	2.50E-02	2.67E-01
pentose acid*	2.69E-02	5.06E-03	2.17E-07	3.04E-02	1.21E-02	1.24E-02	1.05E-01	2.79E-02	1.94E-04
N-succinyl-phenylalanine	3.08E-03	5.40E-03	5.69E-01	-6.19E-04	1.24E-02	9.60E-01	-9.33E-03	2.91E-02	7.49E-01
1-methyl-5-imidazolelactate	-5.23E-03	6.02E-03	3.86E-01	-1.83E-02	1.38E-02	1.88E-01	-2.60E-02	3.25E-02	4.24E-01
(2 or 3)-decenoate (10:1n7 or n8)	-6.85E-03	5.85E-03	2.43E-01	-1.96E-02	1.35E-02	1.46E-01	-2.69E-02	3.16E-02	3.95E-01
chenodeoxycholic acid sulfate (1)	-5.12E-03	4.70E-03	2.77E-01	-8.91E-03	1.08E-02	4.12E-01	-1.68E-02	2.54E-02	5.08E-01
branched-chain, straight-chain, or cyclopropyl 10:1 fatty acid (1)*	-1.64E-02	5.77E-03	4.73E-03	-4.75E-02	1.32E-02	3.69E-04	-7.45E-02	3.12E-02	1.78E-02
branched-chain, straight-chain, or cyclopropyl 10:1 fatty acid (2)*	-1.63E-02	5.66E-03	4.17E-03	-3.95E-02	1.30E-02	2.63E-03	-7.54E-02	3.06E-02	1.44E-02
carnitine of C10H14O2 (5)*	-1.17E-02	5.96E-03	5.13E-02	-2.73E-03	1.38E-02	8.43E-01	-4.34E-02	3.22E-02	1.79E-01
3-bromo-5-chloro-2,6-dihydroxybenzoic acid*	-2.42E-02	5.95E-03	6.31E-05	-4.78E-02	1.38E-02	6.21E-04	-1.64E-01	3.15E-02	4.01E-07
branched-chain, straight-chain, or cyclopropyl 12:1 fatty acid*	-1.39E-02	5.76E-03	1.61E-02	-3.31E-02	1.33E-02	1.30E-02	-6.17E-02	3.12E-02	4.88E-02
decadienedioic acid (C10:2-DC)**	-1.07E-02	5.84E-03	6.86E-02	-2.76E-02	1.34E-02	4.06E-02	-7.18E-02	3.14E-02	2.28E-02
deoxycholic acid 12-sulfate*	-5.61E-03	5.75E-03	3.30E-01	-9.84E-03	1.33E-02	4.58E-01	-4.34E-02	3.09E-02	1.62E-01
GlcNAc sulfate conjugate of C21H34O2 steroid**	-1.02E-03	5.61E-03	8.56E-01	-1.19E-02	1.29E-02	3.57E-01	-2.98E-02	3.02E-02	3.23E-01
cis-3,4-methyleneheptanoate	-1.81E-02	5.95E-03	2.64E-03	-1.35E-02	1.39E-02	3.33E-01	-6.09E-02	3.24E-02	6.11E-02

Supplementary Table 2: Parameter estimates for metabolome-wide association studies for diet-metabolite associations for each of: The Healthy Eating Index-2015 (HEI-15), DASH and AMED diet

cis-3,4-methyleneheptanoylcarnitine	-2.23E-02	5.93E-03	2.12E-04	-1.98E-02	1.39E-02	1.57E-01	-7.24E-02	3.25E-02	2.65E-02
N-acetyl-2-aminoadipate	-1.68E-02	5.78E-03	3.89E-03	-2.56E-02	1.34E-02	5.72E-02	-6.15E-02	3.14E-02	5.13E-02
De(carboxymethoxy) cetirizine acetic acid	3.71E-03	3.44E-03	2.81E-01	6.35E-03	7.92E-03	4.24E-01	6.81E-04	1.86E-02	9.71E-01
4-chlorobenzoic acid	-5.10E-03	5.88E-03	3.86E-01	-9.82E-03	1.35E-02	4.69E-01	1.07E-04	3.17E-02	9.97E-01
torasemide	-7.73E-04	1.06E-03	4.65E-01	-3.12E-03	2.43E-03	2.00E-01	-1.05E-02	5.67E-03	6.53E-02
2-methoxyhydroquinone sulfate (1)	-3.95E-03	5.53E-03	4.76E-01	4.72E-03	1.27E-02	7.11E-01	8.11E-03	2.98E-02	7.86E-01
diazepam	4.77E-04	1.06E-03	6.52E-01	2.68E-03	2.43E-03	2.71E-01	6.96E-03	5.68E-03	2.21E-01
temazepam	2.89E-03	1.65E-03	8.17E-02	3.97E-03	3.82E-03	3.00E-01	2.19E-02	8.87E-03	1.43E-02
2,4-di-tert-butylphenol	-7.09E-03	6.18E-03	2.52E-01	-2.88E-02	1.41E-02	4.29E-02	-2.34E-02	3.33E-02	4.84E-01
3-hydroxyoctanoylcarnitine (1)	1.86E-03	5.68E-03	7.44E-01	9.57E-03	1.31E-02	4.65E-01	-4.25E-03	3.06E-02	8.90E-01
3-hydroxyoctanoylcarnitine (2)	2.99E-03	5.64E-03	5.96E-01	5.07E-03	1.30E-02	6.96E-01	1.17E-02	3.04E-02	7.00E-01
cis-3,4-methyleneheptanoylglycine	-2.10E-02	5.89E-03	4.20E-04	-3.00E-02	1.37E-02	3.01E-02	-8.91E-02	3.20E-02	5.74E-03
bilirubin degradation product, C16H18N2O5 (1)**	-5.21E-03	5.75E-03	3.65E-01	-2.25E-02	1.32E-02	8.89E-02	-7.20E-03	3.10E-02	8.17E-01
bilirubin degradation product, C16H18N2O5 (2)**	-3.64E-03	5.76E-03	5.27E-01	-1.82E-02	1.32E-02	1.70E-01	9.71E-03	3.10E-02	7.55E-01
bilirubin degradation product, C17H18N2O4 (1)**	-6.26E-03	5.84E-03	2.85E-01	-2.13E-02	1.34E-02	1.13E-01	-6.68E-04	3.16E-02	9.83E-01
bilirubin degradation product, C17H18N2O4 (2)**	-5.49E-03	5.77E-03	3.42E-01	-2.10E-02	1.33E-02	1.14E-01	6.25E-04	3.12E-02	9.84E-01
bilirubin degradation product, C17H18N2O4 (3)**	-6.60E-03	5.82E-03	2.57E-01	-2.32E-02	1.33E-02	8.38E-02	-7.10E-04	3.14E-02	9.82E-01
bilirubin degradation product, C17H20N2O5 (1)**	-7.53E-03	5.94E-03	2.05E-01	-2.41E-02	1.36E-02	7.84E-02	-9.55E-03	3.21E-02	7.66E-01
bilirubin degradation product, C17H20N2O5 (2)**	-7.76E-03	5.94E-03	1.93E-01	-2.47E-02	1.36E-02	7.07E-02	-2.80E-03	3.21E-02	9.30E-01
tetrahydrocortisol glucuronide	-4.92E-03	5.66E-03	3.86E-01	-2.75E-02	1.29E-02	3.48E-02	-4.17E-02	3.05E-02	1.72E-01
bilirubin degradation product, C16H18N2O5 (3)**	-5.18E-03	5.71E-03	3.66E-01	-1.55E-02	1.31E-02	2.39E-01	1.21E-02	3.08E-02	6.95E-01
bilirubin degradation product, C16H18N2O5 (4)**	-6.39E-03	5.68E-03	2.62E-01	-1.87E-02	1.31E-02	1.54E-01	7.17E-03	3.07E-02	8.16E-01
N,N-dimethyl-pro-pro	-1.29E-02	5.76E-03	2.54E-02	-2.32E-03	1.34E-02	8.63E-01	-7.44E-02	3.10E-02	1.70E-02
oxindolylalanine	1.85E-02	5.85E-03	1.69E-03	4.40E-02	1.35E-02	1.20E-03	7.23E-02	3.18E-02	2.37E-02
tetrahydrocortisone glucuronide (5)	-4.38E-03	5.46E-03	4.22E-01	-1.13E-02	1.26E-02	3.68E-01	-5.59E-02	2.92E-02	5.68E-02
perfluorohexanesulfonic acid	5.00E-03	5.15E-03	3.33E-01	1.38E-03	1.19E-02	9.07E-01	1.13E-03	2.78E-02	9.68E-01
trans-4-hydroxyproline	-1.52E-02	5.66E-03	7.54E-03	-3.05E-02	1.31E-02	2.06E-02	-6.63E-02	3.07E-02	3.14E-02
allantoin	1.07E-02	5.82E-03	6.81E-02	3.93E-02	1.33E-02	3.31E-03	4.93E-02	3.14E-02	1.18E-01
xanthine	-2.02E-02	5.84E-03	6.17E-04	-1.05E-02	1.37E-02	4.44E-01	-9.29E-02	3.16E-02	3.61E-03
5-oxoproline	2.40E-03	5.80E-03	6.79E-01	1.18E-02	1.33E-02	3.78E-01	5.04E-03	3.12E-02	8.72E-01
sarcosine	-6.30E-03	6.13E-03	3.05E-01	-1.61E-02	1.41E-02	2.56E-01	-4.97E-02	3.30E-02	1.33E-01
pantothenate	1.20E-02	5.50E-03	3.00E-02	3.37E-02	1.26E-02	7.87E-03	2.73E-02	2.98E-02	3.61E-01
pipecolate	1.36E-02	6.01E-03	2.43E-02	1.55E-02	1.39E-02	2.67E-01	8.33E-02	3.23E-02	1.04E-02
phosphoethanolamine	3.75E-03	6.26E-03	5.49E-01	1.88E-02	1.44E-02	1.93E-01	3.53E-02	3.37E-02	2.96E-01
glycerate	2.87E-02	5.38E-03	1.94E-07	6.32E-02	1.24E-02	6.81E-07	1.18E-01	2.96E-02	8.50E-05
3-ureidopropionate	1.66E-02	5.39E-03	2.27E-03	2.60E-02	1.25E-02	3.86E-02	7.26E-02	2.92E-02	1.36E-02
5-KETE	-3.87E-04	2.72E-03	8.87E-01	-2.20E-03	6.26E-03	7.25E-01	2.75E-03	1.47E-02	8.51E-01
N-acetylleucine	-9.71E-03	5.43E-03	7.49E-02	-1.34E-02	1.25E-02	2.87E-01	-1.90E-02	2.94E-02	5.19E-01
N-acetylmethionine	-1.68E-02	5.79E-03	4.08E-03	5.77E-03	1.35E-02	6.70E-01	-8.64E-02	3.13E-02	6.10E-03
N-acetylvaline	8.00E-05	5.86E-03	9.89E-01	1.31E-02	1.35E-02	3.33E-01	-8.12E-03	3.16E-02	7.97E-01
erucate (22:1n9)	9.25E-04	5.94E-03	8.76E-01	-1.54E-02	1.36E-02	2.60E-01	2.45E-03	3.20E-02	9.39E-01
bilirubin (Z,Z)	1.97E-03	5.72E-03	7.31E-01	-7.91E-03	1.32E-02	5.48E-01	3.43E-02	3.08E-02	2.67E-01
thyroxine	-2.44E-03	5.90E-03	6.80E-01	1.38E-02	1.36E-02	3.08E-01	-3.01E-02	3.18E-02	3.44E-01
gamma-glutamyltyrosine	-4.23E-03	5.74E-03	4.62E-01	2.81E-03	1.32E-02	8.32E-01	-2.83E-02	3.09E-02	3.60E-01
alpha-tocopherol	1.59E-02	5.67E-03	5.26E-03	3.08E-03	1.32E-02	8.16E-01	5.15E-02	3.08E-02	9.59E-02
3-hydroxyisobutyrate	-2.36E-03	5.77E-03	6.83E-01	-1.12E-05	1.33E-02	9.99E-01	-3.33E-02	3.10E-02	2.84E-01
N-acetylalanine	-8.86E-03	6.05E-03	1.44E-01	2.35E-02	1.39E-02	9.29E-02	-5.47E-02	3.26E-02	9.41E-02
vanillylmandelate (VMA)	5.70E-03	5.47E-03	2.99E-01	2.53E-02	1.25E-02	4.48E-02	1.54E-03	2.95E-02	9.58E-01
4-acetamidobutanoate	-1.13E-03	5.62E-03	8.40E-01	2.13E-02	1.29E-02	9.86E-02	-1.70E-02	3.03E-02	5.76E-01
3-aminoisobutyrate	-5.07E-03	5.80E-03	3.83E-01	-1.67E-02	1.33E-02	2.12E-01	-2.16E-02	3.13E-02	4.90E-01
3-hydroxy-3-methylglutarate	2.59E-03	5.46E-03	6.36E-01	2.39E-02	1.25E-02	5.67E-02	1.84E-02	2.94E-02	5.31E-01
citrate	-6.30E-03	5.62E-03	2.63E-01	7.37E-03	1.30E-02	5.70E-01	-2.97E-02	3.03E-02	3.28E-01
2-aminobutyrate	-4.00E-03	5.97E-03	5.03E-01	-4.38E-02	1.35E-02	1.30E-03	1.02E-02	3.22E-02	7.52E-01
urate	-6.70E-03	5.17E-03	1.96E-01	-4.17E-04	1.19E-02	9.72E-01	-2.33E-02	2.79E-02	4.04E-01
ursodeoxycholate	-1.66E-02	5.89E-03	5.20E-03	-3.60E-02	1.36E-02	8.39E-03	-2.16E-02	3.21E-02	5.02E-01
oleoyl ethanolamide	-5.46E-03	5.76E-03	3.44E-01	-1.99E-02	1.32E-02	1.33E-01	-6.83E-03	3.11E-02	8.26E-01
gamma-glutamylglutamine	-5.41E-03	5.69E-03	3.43E-01	-6.59E-04	1.31E-02	9.60E-01	-2.36E-02	3.07E-02	4.43E-01
4-hydroxyphenylpyruvate	-3.51E-03	5.44E-03	5.19E-01	2.80E-03	1.25E-02	8.23E-01	-2.97E-02	2.93E-02	3.12E-01
butyrate/isobutyrate (4:0)	2.43E-03	5.56E-03	6.63E-01	3.73E-03	1.28E-02	7.71E-01	3.49E-02	2.99E-02	2.44E-01
N-acetylneuraminate	-3.08E-03	6.00E-03	6.08E-01	1.39E-02	1.38E-02	3.15E-01	-1.46E-02	3.24E-02	6.53E-01
homovanillate (HVA)	5.40E-03	5.68E-03	3.43E-01	8.15E-03	1.31E-02	5.34E-01	1.15E-02	3.07E-02	7.09E-01
creatine	-1.18E-02	5.50E-03	3.34E-02	-4.57E-02	1.25E-02	2.98E-04	-2.28E-02	2.99E-02	4.46E-01
cys-gly, oxidized	-1.90E-02	5.70E-03	9.52E-04	-2.15E-02	1.33E-02	1.07E-01	-9.94E-02	3.07E-02	1.37E-03
2,3-diphosphoglycerate	-4.98E-03	4.78E-03	2.98E-01	-1.16E-03	1.10E-02	9.16E-01	-1.38E-02	2.58E-02	5.94E-01
estrone 3-sulfate	-2.97E-03	4.77E-03	5.34E-01	-2.81E-02	1.09E-02	1.01E-02	-4.09E-02	2.56E-02	1.11E-01
dihomo-linoleate (20:2n6)	-1.48E-02	5.77E-03	1.06E-02	-3.86E-02	1.32E-02	3.81E-03	-6.06E-02	3.13E-02	5.36E-02
gamma-glutamylhistidine	7.94E-03	5.75E-03	1.68E-01	1.42E-02	1.32E-02	2.85E-01	4.38E-02	3.10E-02	1.58E-01
2-hydroxystearate	-1.01E-02	6.05E-03	9.67E-02	-2.85E-02	1.39E-02	4.09E-02	-6.11E-02	3.26E-02	6.17E-02
N1-methyladenosine	-6.77E-03	5.80E-03	2.44E-01	7.26E-03	1.34E-02	5.88E-01	-6.01E-02	3.12E-02	5.48E-02
glycerol	-1.74E-02	5.15E-03	8.26E-04	-3.67E-02	1.19E-02	2.24E-03	-7.75E-02	2.79E-02	5.87E-03
choline	-4.54E-03	6.01E-03	4.50E-01	8.81E-03	1.38E-02	5.25E-01	-1.07E-02	3.24E-02	7.41E-01
anthranilate	3.10E-03	5.08E-03	5.42E-01	-1.42E-02	1.17E-02	2.25E-01	-4.41E-03	2.74E-02	8.72E-01
gamma-glutamylleucine	-1.04E-02	5.53E-03	6.11E-02	-5.24E-03	1.28E-02	6.83E-01	-4.90E-02	2.99E-02	1.02E-01
3-phosphoglycerate	-2.27E-03	5.73E-03	6.93E-01	9.16E-05	1.32E-02	9.94E-01	-2.19E-02	3.09E-02	4.78E-01
3-methoxytyrosine	-4.88E-03	5.69E-03	3.92E-01	1.10E-02	1.31E-02	4.01E-01	-4.00E-02	3.06E-02	1.92E-01
cholate	-9.05E-04	6.02E-03	8.81E-01	5.00E-04	1.38E-02	9.71E-01	4.66E-02	3.23E-02	1.50E-01
fluoxetine	2.73E-04	2.52E-03	9.14E-01	-4.81E-03	5.80E-03	4.08E-01	9.52E-03	1.36E-02	4.84E-01
4-acetamidophenol	3.21E-03	4.24E-03	4.50E-01	1.82E-02	9.71E-03	6.23E-02	2.27E-02	2.28E-02	3.21E-01
naproxen	2.07E-03	3.55E-03	5.60E-01	1.38E-02	8.13E-03	8.98E-02	1.23E-02	1.91E-02	5.19E-01
topiramate	-5.13E-04	1.53E-03	7.38E-01	-1.13E-03	3.53E-03	7.50E-01	7.68E-03	8.25E-03	3.53E-01
beta-hydroxyisovalerate	-7.24E-03	5.39E-03	1.81E-01	-1.75E-02	1.24E-02	1.59E-01	-4.00E-03	2.91E-02	8.91E-01
ibuprofen	1.95E-03	2.92E-03	5.04E-01	-8.54E-03	6.72E-03	2.05E-01	-1.72E-03	1.58E-02	9.13E-01
arachidonoyl ethanolamide	-1.11E-02	5.54E-03	4.59E-02	-2.29E-02	1.28E-02	7.39E-02	-2.32E-02	3.00E-02	4.41E-01
palmitoyl ethanolamide	-1.42E-02	5.86E-03	1.62E-02	-3.31E-02	1.35E-02	1.46E-02	-7.07E-02	3.16E-02	2.61E-02

Supplementary Table 2: Parameter estimates for metabolome-wide association studies for diet-metabolite associations for each of: The Healthy Eating Index-2015 (HEI-15), DASH and AMED diet

N-linoleoylglycine	-5.10E-04	5.69E-03	9.29E-01	1.31E-02	1.31E-02	3.16E-01	2.16E-02	3.07E-02	4.82E-01
N-palmitoyl-sphingosine (d18:1/16:0)	-2.72E-03	5.90E-03	6.45E-01	-1.90E-02	1.35E-02	1.61E-01	-2.70E-02	3.17E-02	3.96E-01
1-palmitoyl-2-oleoyl-GPE (16:0/18:1)	5.36E-03	6.05E-03	3.76E-01	-7.22E-03	1.39E-02	6.05E-01	1.22E-02	3.26E-02	7.10E-01
1-palmitoyl-2-linoleoyl-GPI (16:0/18:2)	-4.32E-03	6.00E-03	4.72E-01	-1.90E-02	1.38E-02	1.70E-01	-5.75E-03	3.24E-02	8.59E-01
1-palmitoyl-2-linoleoyl-GPC (16:0/18:2)	-7.33E-03	5.95E-03	2.19E-01	-3.69E-02	1.36E-02	6.95E-03	-8.91E-03	3.21E-02	7.82E-01
stearoyl sphingomyelin (d18:1/18:0)	-2.09E-02	5.63E-03	2.46E-04	-4.67E-02	1.30E-02	3.79E-04	-1.06E-01	3.04E-02	5.62E-04
1-palmitoyl-2-oleoyl-GPC (16:0/18:1)	-3.65E-03	5.98E-03	5.42E-01	-4.35E-02	1.35E-02	1.43E-03	-1.20E-02	3.22E-02	7.10E-01
N-stearoyl-sphingosine (d18:1/18:0)*	-1.60E-02	5.88E-03	6.84E-03	-3.86E-02	1.35E-02	4.56E-03	-1.01E-01	3.15E-02	1.53E-03
5,6-dihydrothymine	-1.01E-02	5.87E-03	8.66E-02	-1.78E-02	1.35E-02	1.88E-01	-8.82E-02	3.13E-02	5.23E-03
glycochenodeoxycholate	-6.14E-04	6.04E-03	9.19E-01	-4.09E-03	1.39E-02	7.69E-01	-4.04E-02	3.25E-02	2.15E-01
taurochenodeoxycholate	-4.18E-03	6.07E-03	4.91E-01	-1.05E-02	1.40E-02	4.51E-01	-4.92E-02	3.26E-02	1.32E-01
taurocholate	-2.25E-03	5.80E-03	6.99E-01	3.09E-04	1.34E-02	9.82E-01	-2.60E-02	3.12E-02	4.06E-01
taurodeoxycholate	-9.10E-04	6.01E-03	8.80E-01	6.08E-04	1.38E-02	9.65E-01	-5.82E-02	3.22E-02	7.21E-02
hypoxanthine	-8.80E-03	6.24E-03	1.59E-01	-1.17E-03	1.44E-02	9.35E-01	-1.90E-02	3.37E-02	5.73E-01
9,10-DiHOME	4.69E-03	5.82E-03	4.21E-01	3.76E-02	1.32E-02	4.73E-03	3.00E-02	3.13E-02	3.40E-01
linoleate (18:2n6)	-1.16E-02	5.63E-03	4.05E-02	-2.61E-02	1.30E-02	4.49E-02	-4.58E-02	3.04E-02	1.33E-01
laurate (12:0)	-1.15E-02	5.63E-03	4.14E-02	-6.63E-03	1.31E-02	6.12E-01	-4.30E-02	3.05E-02	1.59E-01
quinolinate	-1.30E-02	6.16E-03	3.51E-02	3.35E-04	1.43E-02	9.81E-01	-1.18E-01	3.27E-02	3.86E-04
2-hydroxyhippurate (salicylurate)	1.01E-02	6.07E-03	9.66E-02	4.21E-02	1.38E-02	2.54E-03	2.04E-02	3.28E-02	5.35E-01
N6,N6,N6-trimethyllysine	-1.03E-02	5.73E-03	7.29E-02	-3.64E-03	1.33E-02	7.84E-01	-4.14E-02	3.10E-02	1.82E-01
N-acetylputrescine	-1.70E-03	5.82E-03	7.71E-01	-1.09E-02	1.34E-02	4.14E-01	-2.22E-02	3.13E-02	4.79E-01
N-formylmethionine	-9.78E-03	5.77E-03	9.10E-02	1.11E-02	1.33E-02	4.06E-01	-7.10E-02	3.10E-02	2.25E-02
S-adenosylhomocysteine (SAH)	-1.57E-03	5.93E-03	7.91E-01	2.01E-02	1.36E-02	1.40E-01	-4.77E-02	3.18E-02	1.35E-01
metoprolol	1.62E-03	2.65E-03	5.40E-01	1.15E-02	6.05E-03	5.75E-02	1.56E-02	1.42E-02	2.74E-01
azelate (C9-DC)	-1.86E-04	6.01E-03	9.75E-01	1.87E-02	1.38E-02	1.75E-01	-4.10E-02	3.23E-02	2.05E-01
3-(N-acetyl-L-cystein-S-yl) acetaminophen	4.42E-03	3.86E-03	2.54E-01	1.86E-02	8.83E-03	3.57E-02	1.17E-02	2.08E-02	5.75E-01
4-acetaminophen sulfate	7.28E-03	4.88E-03	1.37E-01	2.80E-02	1.11E-02	1.24E-02	3.13E-02	2.63E-02	2.35E-01
eicosapentaenoate (EPA; 20:5n3)	-4.01E-04	5.83E-03	9.45E-01	-2.02E-02	1.34E-02	1.31E-01	3.43E-02	3.13E-02	2.75E-01
methylsuccinate	1.91E-02	5.65E-03	8.07E-04	3.83E-02	1.31E-02	3.67E-03	8.76E-02	3.06E-02	4.57E-03
ethylmalonate	-3.88E-03	5.63E-03	4.92E-01	-6.14E-03	1.30E-02	6.36E-01	-2.36E-02	3.03E-02	4.38E-01
adenosine 3',5'-cyclic monophosphate (cAMP)	5.62E-04	5.92E-03	9.24E-01	1.72E-02	1.36E-02	2.07E-01	-2.02E-02	3.19E-02	5.26E-01
adenosine 5'-monophosphate (AMP)	-2.46E-03	5.95E-03	6.80E-01	1.29E-02	1.37E-02	3.48E-01	-5.94E-04	3.21E-02	9.85E-01
5-methylthioadenosine (MTA)	-8.20E-03	5.95E-03	1.69E-01	-5.79E-04	1.37E-02	9.66E-01	-3.98E-02	3.21E-02	2.16E-01
N6-methyladenosine	-9.36E-03	5.83E-03	1.10E-01	-3.08E-03	1.35E-02	8.20E-01	-6.22E-02	3.14E-02	4.83E-02
arachidonate (20:4n6)	-1.30E-02	6.06E-03	3.30E-02	-4.20E-02	1.38E-02	2.62E-03	-5.78E-02	3.27E-02	7.85E-02
arginine	7.91E-03	6.30E-03	2.11E-01	1.82E-02	1.45E-02	2.10E-01	4.32E-02	3.40E-02	2.04E-01
aspartate	2.91E-03	5.72E-03	6.11E-01	4.31E-03	1.32E-02	7.44E-01	7.08E-03	3.09E-02	8.19E-01
2-hydroxyphenylacetate	5.42E-04	5.87E-03	9.26E-01	1.87E-02	1.35E-02	1.67E-01	1.19E-02	3.16E-02	7.07E-01
3-(4-hydroxyphenyl)lactate	-6.20E-03	5.12E-03	2.27E-01	-5.30E-03	1.18E-02	6.54E-01	-4.25E-03	2.76E-02	8.78E-01
phenylpyruvate	-1.50E-03	5.78E-03	7.95E-01	1.83E-02	1.33E-02	1.68E-01	-8.69E-03	3.12E-02	7.80E-01
beta-alanine	-1.58E-03	5.95E-03	7.91E-01	1.57E-02	1.37E-02	2.53E-01	-2.51E-02	3.20E-02	4.35E-01
biliverdin	-8.91E-03	5.84E-03	1.28E-01	-1.94E-02	1.35E-02	1.51E-01	-2.74E-02	3.16E-02	3.86E-01
succinate	3.94E-03	5.85E-03	5.01E-01	7.58E-03	1.35E-02	5.74E-01	3.33E-02	3.15E-02	2.91E-01
3-hydroxybutyrate (BHBA)	-1.83E-03	5.65E-03	7.46E-01	-1.46E-02	1.30E-02	2.61E-01	1.41E-02	3.04E-02	6.45E-01
cholesterol	3.41E-03	5.89E-03	5.63E-01	-1.17E-02	1.36E-02	3.88E-01	2.52E-02	3.17E-02	4.28E-01
corticosterone	3.65E-03	5.28E-03	4.90E-01	-1.30E-02	1.21E-02	2.86E-01	-6.51E-03	2.85E-02	8.19E-01
cortisone	1.85E-03	6.06E-03	7.60E-01	-2.63E-02	1.39E-02	5.87E-02	2.61E-04	3.27E-02	9.94E-01
creatinine	-5.34E-03	4.96E-03	2.83E-01	7.61E-03	1.14E-02	5.06E-01	-3.81E-02	2.67E-02	1.55E-01
cysteinylglycine	-1.49E-02	5.57E-03	8.03E-03	-2.30E-02	1.29E-02	7.54E-02	-5.90E-02	3.02E-02	5.15E-02
cystine	-2.84E-02	5.78E-03	1.57E-06	-5.87E-02	1.34E-02	1.70E-05	-1.22E-01	3.16E-02	1.51E-04
sphingosine	-1.08E-02	5.96E-03	7.16E-02	-1.87E-02	1.38E-02	1.74E-01	-4.25E-02	3.22E-02	1.89E-01
deoxycholate	-7.65E-03	5.81E-03	1.89E-01	-2.14E-02	1.33E-02	1.10E-01	-1.57E-02	3.14E-02	6.18E-01
cystathionine	5.14E-03	5.89E-03	3.83E-01	2.81E-02	1.35E-02	3.77E-02	2.67E-02	3.17E-02	4.00E-01
sphinganine	-7.35E-03	6.10E-03	2.29E-01	-1.28E-02	1.41E-02	3.64E-01	-4.84E-02	3.28E-02	1.42E-01
flavin adenine dinucleotide (FAD)	5.24E-03	5.64E-03	3.53E-01	4.21E-03	1.30E-02	7.46E-01	2.61E-02	3.04E-02	3.90E-01
fumarate	-4.31E-03	5.62E-03	4.43E-01	-3.54E-03	1.29E-02	7.85E-01	9.15E-04	3.03E-02	9.76E-01
gamma-glutamylglutamate	-9.47E-03	5.67E-03	9.60E-02	-8.91E-03	1.31E-02	4.97E-01	-4.64E-02	3.06E-02	1.30E-01
gluconate	-4.01E-03	5.72E-03	4.84E-01	1.48E-02	1.32E-02	2.63E-01	-6.93E-03	3.09E-02	8.23E-01
glutarate (C5-DC)	-1.67E-02	5.57E-03	3.01E-03	-1.69E-02	1.30E-02	1.94E-01	2.50E-02	3.05E-02	4.12E-01
glycine	-7.62E-03	5.83E-03	1.92E-01	5.68E-03	1.34E-02	6.73E-01	-3.87E-02	3.14E-02	2.19E-01
glycocholate	-4.26E-04	6.05E-03	9.44E-01	6.10E-03	1.39E-02	6.61E-01	-2.19E-02	3.26E-02	5.02E-01
guanidinoacetate	3.43E-03	5.78E-03	5.53E-01	9.82E-03	1.33E-02	4.61E-01	2.27E-02	3.11E-02	4.66E-01
S-1-pyrroline-5-carboxylate	1.63E-03	5.66E-03	7.73E-01	1.12E-02	1.30E-02	3.90E-01	1.16E-02	3.05E-02	7.03E-01
histidine	1.11E-02	5.84E-03	5.79E-02	3.89E-03	1.35E-02	7.74E-01	4.61E-02	3.15E-02	1.45E-01
cortisol	3.49E-03	6.11E-03	5.68E-01	-1.76E-02	1.40E-02	2.10E-01	-1.17E-02	3.29E-02	7.23E-01
hypotaurine	2.15E-03	5.35E-03	6.88E-01	1.62E-02	1.23E-02	1.87E-01	2.88E-02	2.88E-02	3.18E-01
inosine	-4.44E-03	5.94E-03	4.56E-01	3.35E-03	1.37E-02	8.07E-01	-3.01E-03	3.21E-02	9.25E-01
myo-inositol	2.22E-02	5.45E-03	6.03E-05	3.25E-02	1.28E-02	1.15E-02	1.02E-01	2.96E-02	6.40E-04
isoleucine	-9.91E-03	5.10E-03	5.31E-02	-6.24E-03	1.18E-02	5.98E-01	-3.77E-02	2.76E-02	1.73E-01
2-aminoadipate	-3.50E-03	5.72E-03	5.42E-01	-7.04E-03	1.32E-02	5.93E-01	-6.37E-03	3.09E-02	8.37E-01
citrulline	-2.96E-03	6.02E-03	6.23E-01	4.94E-03	1.39E-02	7.22E-01	3.33E-02	3.24E-02	3.05E-01
leucine	-4.46E-03	5.12E-03	3.85E-01	-5.85E-03	1.18E-02	6.21E-01	-3.30E-02	2.76E-02	2.33E-01
lithocholate	-9.04E-04	5.24E-03	8.63E-01	9.19E-03	1.21E-02	4.47E-01	-1.22E-02	2.82E-02	6.65E-01
lysine	-6.15E-03	5.92E-03	3.00E-01	-8.81E-03	1.36E-02	5.19E-01	-6.06E-02	3.17E-02	5.72E-02
malate	-6.01E-03	5.81E-03	3.02E-01	2.07E-03	1.34E-02	8.77E-01	-1.12E-02	3.14E-02	7.21E-01
methionine	-1.13E-02	5.52E-03	4.24E-02	-6.63E-03	1.28E-02	6.05E-01	-5.56E-02	2.98E-02	6.33E-02
methylmalonate (MMA)	-7.35E-03	5.66E-03	1.95E-01	6.51E-03	1.31E-02	6.19E-01	-6.77E-03	3.06E-02	8.25E-01
palmitate (16:0)	-1.87E-02	5.69E-03	1.17E-03	-4.41E-02	1.31E-02	8.64E-04	-9.19E-02	3.08E-02	3.07E-03
nicotinamide	1.70E-03	6.02E-03	7.77E-01	2.96E-02	1.37E-02	3.22E-02	1.10E-02	3.24E-02	7.35E-01
stearate (18:0)	-1.64E-02	5.87E-03	5.44E-03	-2.98E-02	1.36E-02	2.87E-02	-7.27E-02	3.18E-02	2.28E-02
ornithine	-3.59E-03	5.99E-03	5.50E-01	5.87E-03	1.38E-02	6.71E-01	1.33E-02	3.23E-02	6.80E-01
orotate	-2.91E-03	5.98E-03	6.26E-01	-5.06E-03	1.38E-02	7.13E-01	-2.58E-02	3.22E-02	4.23E-01
palmitoleate (16:1n7)	-1.69E-02	5.18E-03	1.23E-03	-4.63E-02	1.18E-02	1.14E-04	-7.33E-02	2.81E-02	9.66E-03
phenylalanine	-5.24E-03	5.91E-03	3.76E-01	-3.36E-03	1.36E-02	8.05E-01	-4.37E-02	3.18E-02	1.70E-01

Supplementary Table 2: Parameter estimates for metabolome-wide association studies for diet-metabolite associations for each of: The Healthy Eating Index-2015 (HEI-15), DASH and AMED diet

phosphate	-1.58E-04	6.00E-03	9.79E-01	6.76E-03	1.38E-02	6.25E-01	8.13E-03	3.23E-02	8.02E-01
phytanate	-4.32E-03	5.48E-03	4.32E-01	-1.85E-02	1.26E-02	1.42E-01	-2.08E-02	2.96E-02	4.81E-01
proline	-1.95E-02	5.79E-03	8.64E-04	-1.65E-02	1.36E-02	2.25E-01	-7.87E-02	3.15E-02	1.30E-02
lactate	-6.44E-03	5.53E-03	2.46E-01	-3.69E-03	1.28E-02	7.73E-01	-1.66E-02	2.99E-02	5.79E-01
pyridoxal	1.21E-02	4.29E-03	5.04E-03	2.08E-02	9.95E-03	3.79E-02	4.31E-02	2.33E-02	6.58E-02
retinol (Vitamin A)	6.93E-03	5.73E-03	2.28E-01	7.46E-03	1.32E-02	5.73E-01	4.21E-03	3.10E-02	8.92E-01
spermidine	-2.65E-03	5.94E-03	6.56E-01	1.79E-02	1.36E-02	1.90E-01	-2.09E-02	3.20E-02	5.14E-01
salicylate	1.08E-02	6.18E-03	8.26E-02	4.35E-02	1.41E-02	2.16E-03	2.53E-02	3.34E-02	4.50E-01
serine	-9.94E-03	5.80E-03	8.73E-02	-2.76E-02	1.33E-02	3.92E-02	-1.96E-02	3.14E-02	5.33E-01
serotonin	7.85E-03	5.84E-03	1.80E-01	1.60E-02	1.34E-02	2.35E-01	3.64E-02	3.15E-02	2.49E-01
taurine	5.54E-03	6.22E-03	3.74E-01	7.59E-03	1.43E-02	5.97E-01	4.27E-02	3.35E-02	2.03E-01
myristate (14:0)	-2.07E-02	5.33E-03	1.33E-04	-3.80E-02	1.24E-02	2.38E-03	-9.71E-02	2.89E-02	8.98E-04
urea	3.41E-03	5.92E-03	5.66E-01	2.37E-02	1.36E-02	8.24E-02	2.48E-02	3.19E-02	4.37E-01
uridine	1.16E-02	5.94E-03	5.22E-02	2.74E-02	1.37E-02	4.62E-02	6.04E-02	3.20E-02	6.06E-02
2'-deoxyuridine	1.74E-03	6.10E-03	7.75E-01	4.16E-03	1.40E-02	7.67E-01	8.41E-03	3.29E-02	7.98E-01
trans-urocanate	-8.88E-03	5.87E-03	1.32E-01	-6.93E-03	1.36E-02	6.10E-01	-2.70E-02	3.17E-02	3.95E-01
1-methylnicotinamide	-3.49E-03	6.08E-03	5.67E-01	-6.67E-03	1.40E-02	6.34E-01	-4.78E-02	3.27E-02	1.44E-01
glutamate	-6.47E-03	5.39E-03	2.31E-01	-6.79E-03	1.24E-02	5.85E-01	-3.30E-02	2.91E-02	2.57E-01
glutamine	-2.27E-03	5.65E-03	6.88E-01	-3.84E-03	1.30E-02	7.68E-01	-1.88E-02	3.04E-02	5.37E-01
threonine	-1.80E-02	5.90E-03	2.49E-03	-2.27E-02	1.37E-02	1.00E-01	-5.64E-02	3.22E-02	8.09E-02
tryptophan	6.83E-03	5.51E-03	2.17E-01	1.44E-02	1.27E-02	2.58E-01	3.62E-03	2.98E-02	9.03E-01
valine	-5.15E-03	5.51E-03	3.50E-01	2.50E-03	1.27E-02	8.44E-01	-1.13E-02	2.97E-02	7.04E-01
glucose	-1.47E-02	6.00E-03	1.48E-02	-3.61E-02	1.38E-02	9.47E-03	-7.19E-02	3.24E-02	2.73E-02
12,13-DiHOME	7.42E-03	5.55E-03	1.83E-01	3.29E-02	1.27E-02	9.93E-03	4.01E-02	2.99E-02	1.81E-01
alpha-ketobutyrate	-7.00E-03	5.76E-03	2.25E-01	-3.01E-02	1.32E-02	2.33E-02	-9.23E-03	3.11E-02	7.67E-01
betaine	1.32E-02	5.78E-03	2.35E-02	3.05E-02	1.33E-02	2.26E-02	8.47E-02	3.10E-02	6.75E-03
cysteine	-1.09E-02	5.74E-03	5.87E-02	-1.15E-02	1.33E-02	3.89E-01	-7.70E-02	3.08E-02	1.31E-02
mannose	-1.76E-02	5.69E-03	2.14E-03	-3.99E-02	1.31E-02	2.51E-03	-9.71E-02	3.06E-02	1.68E-03
dimethylglycine	4.76E-03	5.86E-03	4.17E-01	2.41E-02	1.34E-02	7.40E-02	2.50E-02	3.16E-02	4.30E-01
alanine	-1.64E-02	5.99E-03	6.57E-03	-2.94E-02	1.39E-02	3.47E-02	-8.13E-02	3.23E-02	1.25E-02
tyrosine	-4.69E-04	5.90E-03	9.37E-01	-6.20E-03	1.36E-02	6.48E-01	-1.70E-02	3.18E-02	5.94E-01
pseudouridine	-4.80E-03	5.67E-03	3.98E-01	1.10E-02	1.31E-02	3.99E-01	-5.43E-02	3.04E-02	7.58E-02
pyruvate	-1.25E-02	5.82E-03	3.32E-02	-1.69E-02	1.35E-02	2.10E-01	-5.88E-02	3.14E-02	6.21E-02
uracil	7.78E-03	5.64E-03	1.69E-01	2.78E-02	1.29E-02	3.24E-02	8.95E-02	3.00E-02	3.14E-03
xylose	1.34E-02	5.37E-03	1.28E-02	2.33E-02	1.24E-02	6.20E-02	6.02E-02	2.90E-02	3.90E-02
cytidine	-7.47E-03	6.11E-03	2.23E-01	6.57E-03	1.41E-02	6.41E-01	-6.26E-03	3.30E-02	8.50E-01
arabinose	6.09E-03	5.19E-03	2.42E-01	1.06E-02	1.20E-02	3.79E-01	4.83E-02	2.79E-02	8.49E-02
cotinine	-5.08E-03	3.06E-03	9.78E-02	-1.66E-02	7.01E-03	1.85E-02	-7.93E-03	1.66E-02	6.32E-01
caffeine	-1.35E-02	5.87E-03	2.21E-02	-3.02E-02	1.35E-02	2.65E-02	-5.37E-02	3.18E-02	9.19E-02
fructose	-2.27E-03	6.02E-03	7.06E-01	-1.16E-02	1.38E-02	4.03E-01	-1.02E-03	3.25E-02	9.75E-01
adenine	-8.71E-03	5.88E-03	1.39E-01	-1.61E-02	1.35E-02	2.35E-01	-6.83E-02	3.15E-02	3.11E-02
cytosine	5.92E-03	4.75E-03	2.14E-01	5.90E-03	1.10E-02	5.91E-01	2.82E-02	2.56E-02	2.72E-01
caprate (10:0)	-1.23E-02	5.73E-03	3.35E-02	-2.56E-02	1.32E-02	5.42E-02	-3.10E-02	3.11E-02	3.20E-01
margarate (17:0)	-2.32E-02	5.80E-03	8.14E-05	-4.34E-02	1.35E-02	1.44E-03	-1.12E-01	3.15E-02	4.45E-04
nonadecanoate (19:0)	-1.09E-02	5.81E-03	6.21E-02	-1.91E-02	1.34E-02	1.55E-01	-5.10E-02	3.14E-02	1.05E-01
arachidate (20:0)	1.41E-03	5.89E-03	8.10E-01	7.77E-03	1.35E-02	5.67E-01	6.28E-03	3.17E-02	8.43E-01
maltose	-2.03E-05	6.13E-03	9.97E-01	1.94E-02	1.41E-02	1.70E-01	1.33E-02	3.30E-02	6.87E-01
asparagine	6.01E-03	5.87E-03	3.07E-01	1.75E-02	1.35E-02	1.96E-01	4.06E-02	3.16E-02	2.00E-01
N-stearoyl-sphinganine (d18:0/18:0)*	-2.14E-02	5.91E-03	3.49E-04	-6.69E-02	1.33E-02	9.37E-07	-1.12E-01	3.19E-02	5.03E-04
dihydroorotate	4.34E-04	5.80E-03	9.40E-01	-8.68E-03	1.33E-02	5.16E-01	-3.65E-02	3.12E-02	2.42E-01
alpha-ketoglutarate	-1.40E-02	5.83E-03	1.73E-02	-1.35E-02	1.35E-02	3.19E-01	-5.37E-02	3.16E-02	9.03E-02
caprylate (8:0)	-5.49E-03	5.58E-03	3.26E-01	-2.23E-02	1.28E-02	8.23E-02	4.17E-03	3.01E-02	8.90E-01
sucrose	-2.04E-03	5.59E-03	7.15E-01	2.85E-02	1.28E-02	2.60E-02	-2.98E-02	3.01E-02	3.23E-01
kynurenate	-2.43E-03	5.72E-03	6.72E-01	2.28E-03	1.32E-02	8.62E-01	-5.24E-02	3.07E-02	8.90E-02
pentadecanoate (15:0)	-2.19E-02	5.39E-03	6.52E-05	-3.64E-02	1.26E-02	4.15E-03	-1.13E-01	2.91E-02	1.37E-04
X-07765	-1.57E-02	6.04E-03	1.00E-02	-2.09E-02	1.40E-02	1.36E-01	-8.51E-02	3.26E-02	9.42E-03
X-10458	1.09E-02	5.84E-03	6.37E-02	2.31E-02	1.34E-02	8.70E-02	3.56E-02	3.16E-02	2.60E-01
X-11299	-5.79E-03	6.27E-03	3.56E-01	-3.13E-02	1.43E-02	2.97E-02	-2.93E-02	3.38E-02	3.87E-01
X-11308	-2.02E-02	5.74E-03	5.21E-04	-4.32E-02	1.33E-02	1.25E-03	-7.72E-02	3.13E-02	1.42E-02
X-11315	3.89E-02	5.11E-03	3.76E-13	6.56E-02	1.23E-02	2.05E-07	1.70E-01	2.85E-02	7.02E-09
X-11372	-1.49E-02	5.90E-03	1.21E-02	-1.60E-02	1.37E-02	2.45E-01	-4.60E-02	3.20E-02	1.52E-01
X-11381	-1.99E-02	5.69E-03	5.44E-04	-5.60E-02	1.30E-02	2.17E-05	-1.17E-01	3.06E-02	1.70E-04
X-11444	-1.03E-03	5.71E-03	8.57E-01	9.38E-03	1.31E-02	4.76E-01	-3.23E-02	3.07E-02	2.95E-01
X-11470	-1.32E-03	5.52E-03	8.12E-01	-7.06E-03	1.27E-02	5.79E-01	-3.35E-02	2.97E-02	2.60E-01
X-11478	-1.97E-02	6.01E-03	1.22E-03	-2.51E-02	1.40E-02	7.45E-02	-9.50E-02	3.25E-02	3.79E-03
X-11483	-8.61E-03	6.23E-03	1.68E-01	-3.64E-02	1.42E-02	1.11E-02	-3.52E-02	3.36E-02	2.96E-01
X-11632	-4.01E-03	5.88E-03	4.95E-01	-1.03E-02	1.35E-02	4.48E-01	-1.30E-02	3.17E-02	6.81E-01
X-11787	2.08E-02	5.55E-03	2.21E-04	4.02E-02	1.29E-02	1.97E-03	6.95E-02	3.04E-02	2.28E-02
X-11795	-3.36E-03	5.79E-03	5.62E-01	-3.44E-02	1.32E-02	9.59E-03	1.02E-02	3.12E-02	7.45E-01
X-11843	1.40E-03	5.80E-03	8.10E-01	-3.68E-04	1.34E-02	9.78E-01	-5.11E-02	3.11E-02	1.02E-01
X-11847	2.15E-02	5.27E-03	5.66E-05	1.41E-02	1.25E-02	2.60E-01	1.05E-01	2.85E-02	2.74E-04
X-11849	1.83E-02	5.15E-03	4.62E-04	1.13E-02	1.21E-02	3.50E-01	1.03E-01	2.77E-02	2.47E-04
X-11850	7.64E-03	5.91E-03	1.97E-01	1.23E-02	1.36E-02	3.68E-01	-2.49E-02	3.19E-02	4.36E-01
X-11852	6.57E-03	6.15E-03	2.86E-01	1.04E-02	1.42E-02	4.64E-01	1.84E-02	3.32E-02	5.79E-01
X-11858	1.43E-02	5.04E-03	4.97E-03	9.59E-03	1.17E-02	4.15E-01	8.31E-02	2.71E-02	2.37E-03
X-11880	-2.12E-02	5.90E-03	3.98E-04	-2.64E-02	1.38E-02	5.64E-02	-8.33E-02	3.21E-02	1.01E-02
X-11979	4.40E-03	5.75E-03	4.45E-01	2.79E-02	1.31E-02	3.45E-02	-1.24E-02	3.10E-02	6.90E-01
X-12007	7.60E-03	5.81E-03	1.92E-01	2.77E-02	1.33E-02	3.86E-02	1.72E-02	3.14E-02	5.85E-01
X-12013	2.27E-03	5.67E-03	6.90E-01	7.60E-03	1.30E-02	5.61E-01	-4.10E-02	3.05E-02	1.80E-01
X-12026	-8.55E-03	6.05E-03	1.59E-01	2.17E-02	1.39E-02	1.21E-01	-8.01E-02	3.24E-02	1.40E-02
X-12027	3.89E-03	4.51E-03	3.89E-01	2.15E-02	1.03E-02	3.77E-02	-1.64E-02	2.43E-02	5.01E-01
X-12100	-2.47E-03	5.94E-03	6.78E-01	1.43E-02	1.37E-02	2.94E-01	-3.51E-02	3.20E-02	2.74E-01
X-12101	-6.17E-03	5.68E-03	2.79E-01	-1.29E-02	1.31E-02	3.26E-01	1.46E-02	3.07E-02	6.34E-01
X-12104	-1.03E-02	6.18E-03	9.70E-02	2.17E-02	1.42E-02	1.29E-01	-6.38E-02	3.33E-02	5.59E-02

Supplementary Table 2: Parameter estimates for metabolome-wide association studies for diet-metabolite associations for each of: The Healthy Eating Index-2015 (HEI-15), DASH and AMED diet

X-12111	1.59E-02	5.20E-03	2.48E-03	4.61E-02	1.18E-02	1.25E-04	4.68E-02	2.83E-02	9.97E-02
X-12193	3.46E-03	5.97E-03	5.62E-01	2.33E-02	1.37E-02	9.03E-02	3.15E-02	3.21E-02	3.28E-01
X-12216	9.83E-04	5.94E-03	8.69E-01	1.33E-02	1.36E-02	3.32E-01	-2.10E-02	3.20E-02	5.13E-01
X-12221	8.85E-03	4.95E-03	7.46E-02	9.99E-03	1.14E-02	3.83E-01	1.06E-02	2.68E-02	6.93E-01
X-12261	2.83E-03	5.32E-03	5.95E-01	8.05E-03	1.22E-02	5.11E-01	-2.50E-02	2.86E-02	3.83E-01
X-12262	3.45E-03	4.75E-03	4.69E-01	8.20E-03	1.09E-02	4.54E-01	-1.06E-02	2.56E-02	6.80E-01
X-12306	2.37E-02	5.20E-03	7.98E-06	3.78E-02	1.22E-02	2.17E-03	1.36E-01	2.79E-02	1.87E-06
X-12407	2.97E-04	5.37E-03	9.56E-01	1.12E-02	1.23E-02	3.66E-01	-6.52E-04	2.89E-02	9.82E-01
X-12410	2.00E-02	5.58E-03	4.04E-04	3.86E-02	1.29E-02	3.10E-03	4.61E-02	3.06E-02	1.33E-01
X-12411	2.70E-03	5.62E-03	6.31E-01	1.40E-02	1.29E-02	2.80E-01	-1.85E-02	3.03E-02	5.42E-01
X-12456	-1.48E-02	5.64E-03	9.35E-03	-3.07E-02	1.30E-02	1.91E-02	-1.03E-01	3.02E-02	7.43E-04
X-12544	8.28E-03	6.17E-03	1.81E-01	2.98E-02	1.41E-02	3.55E-02	-1.23E-02	3.33E-02	7.12E-01
X-12680	1.08E-02	5.89E-03	6.79E-02	6.93E-02	1.30E-02	2.02E-07	4.13E-02	3.19E-02	1.96E-01
X-12701	5.89E-03	4.25E-03	1.67E-01	1.77E-02	9.77E-03	7.16E-02	-7.95E-03	2.30E-02	7.30E-01
X-12707	-2.58E-03	5.93E-03	6.64E-01	1.95E-02	1.36E-02	1.54E-01	-1.54E-02	3.20E-02	6.30E-01
X-12714	6.65E-03	4.92E-03	1.77E-01	1.50E-02	1.13E-02	1.87E-01	1.23E-02	2.66E-02	6.44E-01
X-12726	2.96E-02	5.46E-03	1.25E-07	5.71E-02	1.28E-02	1.11E-05	1.27E-01	3.00E-02	2.96E-05
X-12729	4.42E-03	5.19E-03	3.95E-01	1.79E-02	1.19E-02	1.35E-01	4.38E-02	2.79E-02	1.18E-01
X-12730	4.71E-03	4.80E-03	3.28E-01	-2.57E-03	1.11E-02	8.16E-01	-1.13E-02	2.59E-02	6.64E-01
X-12731	1.00E-02	4.95E-03	4.33E-02	2.86E-02	1.14E-02	1.24E-02	1.01E-02	2.69E-02	7.06E-01
X-12738	2.87E-03	4.53E-03	5.27E-01	6.98E-03	1.04E-02	5.04E-01	1.39E-02	2.44E-02	5.71E-01
X-12740	7.10E-03	5.75E-03	2.18E-01	1.37E-02	1.33E-02	3.03E-01	5.55E-02	3.09E-02	7.37E-02
X-12798	-1.04E-02	5.49E-03	5.96E-02	-2.72E-02	1.26E-02	3.22E-02	-5.34E-02	2.96E-02	7.22E-02
X-12812	1.25E-02	5.74E-03	3.02E-02	2.89E-02	1.32E-02	2.94E-02	5.41E-02	3.10E-02	8.21E-02
X-12815	6.45E-04	4.22E-03	8.79E-01	3.14E-04	9.70E-03	9.74E-01	1.27E-02	2.27E-02	5.78E-01
X-12816	1.99E-04	5.01E-03	9.68E-01	-1.64E-02	1.15E-02	1.55E-01	-2.25E-03	2.70E-02	9.34E-01
X-12822	-9.23E-03	5.87E-03	1.17E-01	-1.59E-02	1.35E-02	2.40E-01	-1.34E-02	3.17E-02	6.73E-01
X-12830	1.85E-03	5.66E-03	7.44E-01	-1.84E-02	1.30E-02	1.58E-01	1.43E-02	3.05E-02	6.40E-01
X-12839	-8.89E-03	5.27E-03	9.27E-02	-2.09E-02	1.21E-02	8.62E-02	-4.27E-02	2.84E-02	1.34E-01
X-12844	9.13E-03	5.67E-03	1.09E-01	6.03E-03	1.31E-02	6.46E-01	7.93E-03	3.07E-02	7.96E-01
X-12847	-1.48E-03	6.10E-03	8.09E-01	-7.55E-03	1.40E-02	5.91E-01	9.97E-03	3.29E-02	7.62E-01
X-12849	2.20E-03	5.69E-03	7.00E-01	-5.44E-03	1.31E-02	6.78E-01	-8.63E-03	3.07E-02	7.78E-01
X-12851	1.67E-03	5.76E-03	7.72E-01	4.40E-04	1.33E-02	9.74E-01	7.37E-03	3.11E-02	8.13E-01
X-12906	1.11E-02	5.81E-03	5.65E-02	3.58E-02	1.33E-02	7.50E-03	4.53E-02	3.14E-02	1.50E-01
X-13431	-8.81E-03	5.67E-03	1.21E-01	-9.36E-03	1.31E-02	4.75E-01	-3.67E-02	3.06E-02	2.31E-01
X-13507	1.34E-02	5.14E-03	9.47E-03	2.32E-02	1.19E-02	5.24E-02	7.38E-02	2.77E-02	8.07E-03
X-13553	-3.91E-03	5.51E-03	4.78E-01	1.59E-02	1.27E-02	2.11E-01	-3.12E-02	2.96E-02	2.94E-01
X-13658	-1.47E-04	5.22E-03	9.78E-01	1.29E-02	1.20E-02	2.83E-01	9.68E-03	2.82E-02	7.31E-01
X-13695	7.98E-03	5.31E-03	1.34E-01	7.01E-03	1.23E-02	5.68E-01	1.54E-02	2.87E-02	5.93E-01
X-13723	8.99E-03	4.54E-03	4.85E-02	2.05E-02	1.04E-02	5.08E-02	4.55E-02	2.45E-02	6.41E-02
X-13726	1.55E-02	5.43E-03	4.51E-03	3.25E-02	1.25E-02	1.01E-02	3.95E-02	2.96E-02	1.83E-01
X-13728	-7.34E-03	5.78E-03	2.05E-01	9.89E-03	1.33E-02	4.59E-01	-2.39E-02	3.12E-02	4.44E-01
X-13729	3.61E-04	5.97E-03	9.52E-01	2.40E-02	1.37E-02	8.06E-02	-2.13E-02	3.21E-02	5.07E-01
X-13844	1.20E-02	5.30E-03	2.46E-02	3.75E-02	1.21E-02	2.16E-03	2.89E-02	2.88E-02	3.17E-01
X-13846	5.25E-03	3.68E-03	1.55E-01	5.29E-03	8.50E-03	5.34E-01	1.16E-02	1.99E-02	5.60E-01
X-13866	4.40E-03	5.98E-03	4.62E-01	-6.56E-03	1.38E-02	6.34E-01	5.54E-02	3.21E-02	8.50E-02
X-14056	-2.81E-02	5.48E-03	5.54E-07	-5.10E-02	1.28E-02	9.08E-05	-1.14E-01	3.01E-02	1.97E-04
X-14939	-9.59E-03	5.82E-03	1.00E-01	-1.97E-02	1.34E-02	1.42E-01	-2.32E-02	3.15E-02	4.62E-01
X-15486	-2.53E-02	5.93E-03	2.66E-05	-3.65E-02	1.39E-02	9.11E-03	-1.19E-01	3.22E-02	2.71E-04
X-15503	-4.50E-03	5.83E-03	4.40E-01	2.02E-02	1.34E-02	1.32E-01	-5.58E-02	3.13E-02	7.53E-02
X-15728	1.24E-02	5.88E-03	3.55E-02	1.06E-02	1.36E-02	4.38E-01	2.63E-02	3.19E-02	4.10E-01
X-16087	-1.61E-02	5.80E-03	5.84E-03	-3.16E-02	1.34E-02	1.89E-02	-5.61E-02	3.15E-02	7.58E-02
X-16124	5.60E-03	5.58E-03	3.16E-01	-5.59E-03	1.29E-02	6.64E-01	2.50E-02	3.01E-02	4.07E-01
X-16397	-1.14E-02	5.65E-03	4.46E-02	-1.98E-02	1.30E-02	1.30E-01	-2.48E-02	3.06E-02	4.18E-01
X-16576	4.55E-03	5.64E-03	4.21E-01	4.94E-03	1.30E-02	7.04E-01	1.20E-02	3.04E-02	6.93E-01
X-16580	-1.21E-02	5.76E-03	3.61E-02	-1.77E-02	1.33E-02	1.85E-01	-3.84E-02	3.12E-02	2.19E-01
X-16649	7.22E-03	4.96E-03	1.47E-01	2.24E-02	1.14E-02	5.00E-02	2.02E-02	2.68E-02	4.52E-01
X-16935	-2.28E-02	5.38E-03	2.97E-05	-3.41E-02	1.26E-02	7.30E-03	-6.85E-02	2.96E-02	2.14E-02
X-16964	-1.12E-02	5.78E-03	5.29E-02	-4.93E-02	1.31E-02	1.98E-04	-5.79E-02	3.12E-02	6.41E-02
X-17010	-1.35E-03	5.70E-03	8.13E-01	-1.82E-02	1.31E-02	1.64E-01	-1.45E-03	3.07E-02	9.62E-01
X-17146	-5.71E-04	6.01E-03	9.24E-01	-1.82E-02	1.38E-02	1.88E-01	-5.42E-03	3.24E-02	8.67E-01
X-17162	-3.28E-03	5.03E-03	5.16E-01	-9.80E-03	1.16E-02	3.98E-01	-2.90E-02	2.71E-02	2.85E-01
X-17301	-1.10E-03	2.87E-03	7.00E-01	-9.12E-03	6.58E-03	1.67E-01	-5.69E-03	1.55E-02	7.13E-01
X-17306	-4.98E-05	2.56E-03	9.84E-01	-1.01E-02	5.86E-03	8.67E-02	3.00E-03	1.38E-02	8.28E-01
X-17325	9.41E-03	5.62E-03	9.54E-02	3.40E-02	1.28E-02	8.58E-03	3.39E-02	3.04E-02	2.66E-01
X-17335	-4.37E-03	5.67E-03	4.41E-01	-1.93E-02	1.30E-02	1.39E-01	-2.47E-02	3.05E-02	4.20E-01
X-17346	-1.52E-02	5.84E-03	9.78E-03	-7.84E-03	1.36E-02	5.65E-01	-8.82E-02	3.14E-02	5.35E-03
X-17348	5.70E-03	4.61E-03	2.17E-01	-1.28E-03	1.06E-02	9.04E-01	3.60E-02	2.48E-02	1.48E-01
X-17351	2.61E-02	5.40E-03	2.13E-06	5.86E-02	1.25E-02	4.06E-06	1.08E-01	2.96E-02	3.15E-04
X-17353	-3.17E-04	4.90E-03	9.48E-01	6.75E-03	1.13E-02	5.49E-01	3.89E-03	2.64E-02	8.83E-01
X-17354	2.20E-02	5.11E-03	2.37E-05	3.64E-02	1.20E-02	2.55E-03	8.37E-02	2.80E-02	3.05E-03
X-17357	1.33E-04	5.65E-03	9.81E-01	-2.84E-03	1.30E-02	8.27E-01	-2.09E-02	3.04E-02	4.92E-01
X-17365	2.34E-03	5.33E-03	6.61E-01	-5.13E-03	1.23E-02	6.76E-01	6.30E-02	2.85E-02	2.78E-02
X-17367	5.68E-03	5.44E-03	2.97E-01	2.94E-02	1.24E-02	1.85E-02	1.74E-02	2.93E-02	5.53E-01
X-17438	2.24E-02	5.80E-03	1.43E-04	3.62E-02	1.35E-02	7.79E-03	7.41E-02	3.18E-02	2.03E-02
X-17612	5.89E-03	5.85E-03	3.16E-01	8.85E-03	1.35E-02	5.12E-01	-1.19E-02	3.16E-02	7.06E-01
X-17653	-1.17E-02	5.96E-03	5.08E-02	-1.92E-02	1.38E-02	1.65E-01	-6.35E-02	3.21E-02	4.90E-02
X-17654	2.27E-03	5.71E-03	6.92E-01	2.02E-02	1.31E-02	1.23E-01	1.29E-02	3.08E-02	6.74E-01
X-17655	2.94E-03	4.03E-03	4.66E-01	-4.23E-03	9.28E-03	6.49E-01	1.32E-02	2.17E-02	5.43E-01
X-17676	2.74E-03	5.89E-03	6.42E-01	3.44E-02	1.34E-02	1.08E-02	-1.12E-02	3.18E-02	7.25E-01
X-17682	-1.09E-02	4.77E-03	2.33E-02	-7.79E-03	1.11E-02	4.82E-01	-5.44E-02	2.57E-02	3.56E-02
X-17685	5.46E-03	5.62E-03	3.33E-01	8.83E-03	1.30E-02	4.96E-01	6.83E-02	3.01E-02	2.39E-02
X-17686	8.81E-03	4.63E-03	5.79E-02	1.37E-02	1.07E-02	2.01E-01	2.69E-02	2.50E-02	2.83E-01
X-17690	6.80E-03	6.02E-03	2.59E-01	-5.08E-03	1.39E-02	7.14E-01	3.90E-02	3.24E-02	2.30E-01

X-17692	-4.50E-03	4.52E-03	3.20E-01	-1.32E-02	1.04E-02	2.05E-01	7.35E-03	2.44E-02	7.63E-01
X-17735	1.07E-03	5.43E-03	8.44E-01	1.87E-02	1.25E-02	1.34E-01	-1.73E-03	2.93E-02	9.53E-01
X-17761	-8.51E-03	5.47E-03	1.21E-01	-4.67E-02	1.23E-02	1.86E-04	-3.66E-02	2.95E-02	2.17E-01
X-17765	2.95E-03	3.43E-03	3.91E-01	3.39E-03	7.90E-03	6.68E-01	-9.09E-03	1.85E-02	6.24E-01
X-18240	1.15E-03	3.83E-03	7.65E-01	-4.99E-03	8.81E-03	5.71E-01	-1.57E-02	2.06E-02	4.48E-01
X-18345	-3.63E-03	6.11E-03	5.53E-01	2.02E-02	1.40E-02	1.51E-01	1.35E-02	3.29E-02	6.82E-01
X-18779	-1.59E-03	5.96E-03	7.89E-01	-1.20E-02	1.37E-02	3.82E-01	-6.32E-02	3.19E-02	4.88E-02
X-18838	-4.87E-03	4.56E-03	2.86E-01	-2.85E-03	1.05E-02	7.87E-01	1.87E-04	2.46E-02	9.94E-01
X-18886	-1.85E-02	5.74E-03	1.40E-03	-4.97E-02	1.31E-02	1.88E-04	-8.90E-02	3.11E-02	4.51E-03
X-18887	-1.25E-02	5.80E-03	3.24E-02	-5.51E-03	1.35E-02	6.82E-01	-2.45E-02	3.15E-02	4.37E-01
X-18888	3.60E-03	5.60E-03	5.21E-01	1.25E-02	1.29E-02	3.33E-01	1.34E-02	3.02E-02	6.57E-01
X-18899	7.62E-04	5.96E-03	8.98E-01	-2.51E-03	1.37E-02	8.55E-01	1.96E-02	3.21E-02	5.41E-01
X-18901	1.53E-02	5.25E-03	3.94E-03	2.06E-02	1.22E-02	9.28E-02	7.39E-02	2.84E-02	9.73E-03
X-18913	-2.97E-03	6.00E-03	6.21E-01	1.13E-02	1.38E-02	4.15E-01	-7.90E-03	3.23E-02	8.07E-01
X-18921	-1.72E-02	5.97E-03	4.40E-03	-3.73E-02	1.38E-02	7.15E-03	-8.11E-02	3.23E-02	1.26E-02
X-18922	-5.85E-03	6.06E-03	3.35E-01	-3.24E-02	1.38E-02	1.99E-02	1.93E-02	3.27E-02	5.56E-01
X-18935	1.04E-02	5.09E-03	4.24E-02	1.52E-02	1.18E-02	1.98E-01	4.16E-02	2.75E-02	1.31E-01
X-19141	6.60E-03	5.77E-03	2.53E-01	2.43E-02	1.32E-02	6.71E-02	2.69E-02	3.11E-02	3.87E-01
X-19183	5.53E-03	4.55E-03	2.25E-01	-1.29E-03	1.05E-02	9.02E-01	5.25E-02	2.44E-02	3.22E-02
X-19299	6.28E-03	5.74E-03	2.74E-01	1.19E-02	1.32E-02	3.70E-01	4.24E-03	3.10E-02	8.91E-01
X-19438	-2.55E-03	5.78E-03	6.60E-01	-8.31E-03	1.33E-02	5.33E-01	-2.32E-02	3.11E-02	4.56E-01
X-21258	1.02E-02	6.18E-03	9.91E-02	1.65E-02	1.43E-02	2.50E-01	5.45E-02	3.33E-02	1.03E-01
X-21286	-5.36E-03	5.97E-03	3.70E-01	2.02E-03	1.38E-02	8.83E-01	-2.57E-02	3.22E-02	4.25E-01
X-21310	-1.16E-02	5.94E-03	5.08E-02	-2.18E-02	1.37E-02	1.13E-01	-7.54E-02	3.19E-02	1.88E-02
X-21312	-6.33E-04	5.66E-03	9.11E-01	-3.59E-03	1.30E-02	7.83E-01	1.06E-02	3.05E-02	7.27E-01
X-21315	-2.20E-03	3.92E-03	5.75E-01	-2.45E-03	9.02E-03	7.86E-01	-1.46E-02	2.11E-02	4.90E-01
X-21319	-1.52E-02	5.99E-03	1.15E-02	-2.78E-02	1.39E-02	4.61E-02	-7.30E-02	3.24E-02	2.49E-02
X-21339	-2.58E-02	5.88E-03	1.57E-05	-4.25E-02	1.38E-02	2.18E-03	-9.98E-02	3.22E-02	2.14E-03
X-21351	4.13E-04	6.06E-03	9.46E-01	-1.89E-03	1.39E-02	8.92E-01	1.54E-03	3.26E-02	9.63E-01
X-21353	-7.72E-03	5.92E-03	1.94E-01	-8.45E-03	1.37E-02	5.37E-01	-2.99E-02	3.20E-02	3.50E-01
X-21364	-1.76E-02	5.46E-03	1.40E-03	-1.79E-02	1.28E-02	1.62E-01	-9.47E-02	2.94E-02	1.44E-03
X-21383	-1.17E-02	5.77E-03	4.36E-02	-3.80E-02	1.32E-02	4.23E-03	-4.92E-02	3.12E-02	1.16E-01
X-21441	-1.39E-02	5.51E-03	1.24E-02	-2.39E-02	1.27E-02	6.21E-02	-7.53E-02	2.97E-02	1.17E-02
X-21442	4.73E-03	5.29E-03	3.72E-01	-1.31E-02	1.22E-02	2.82E-01	1.50E-03	2.85E-02	9.58E-01
X-21467	-1.23E-02	5.91E-03	3.79E-02	1.86E-03	1.37E-02	8.92E-01	-4.09E-02	3.20E-02	2.02E-01
X-21470	-9.92E-03	5.54E-03	7.47E-02	-3.48E-02	1.27E-02	6.32E-03	-6.59E-02	2.98E-02	2.77E-02
X-21471	-9.41E-03	5.60E-03	9.41E-02	4.29E-03	1.30E-02	7.41E-01	-4.72E-02	3.02E-02	1.20E-01
X-21607	-2.65E-04	5.99E-03	9.65E-01	-4.83E-03	1.38E-02	7.26E-01	-2.30E-03	3.23E-02	9.43E-01
X-21628	8.39E-03	3.66E-03	2.28E-02	1.65E-02	8.45E-03	5.14E-02	2.66E-02	1.99E-02	1.82E-01
X-21661	1.14E-02	4.48E-03	1.12E-02	4.31E-03	1.04E-02	6.80E-01	6.76E-02	2.41E-02	5.29E-03
X-21733	1.98E-02	5.54E-03	4.19E-04	5.21E-02	1.27E-02	5.05E-05	4.01E-02	3.04E-02	1.88E-01
X-21736	-2.56E-02	5.67E-03	9.30E-06	-6.94E-02	1.29E-02	1.49E-07	-9.49E-02	3.11E-02	2.54E-03
X-21740	-1.54E-02	5.21E-03	3.29E-03	-3.53E-02	1.20E-02	3.50E-03	-4.84E-02	2.84E-02	8.86E-02
X-21742	3.81E-03	5.02E-03	4.49E-01	5.03E-03	1.16E-02	6.64E-01	-1.41E-02	2.71E-02	6.04E-01
X-21752	2.62E-02	5.19E-03	8.52E-07	8.34E-02	1.14E-02	3.22E-12	5.46E-02	2.91E-02	6.11E-02
X-21788	-1.00E-02	4.98E-03	4.50E-02	-9.45E-03	1.15E-02	4.13E-01	-4.31E-02	2.69E-02	1.10E-01
X-21796	-8.71E-03	5.59E-03	1.20E-01	-1.36E-02	1.29E-02	2.93E-01	-2.86E-02	3.02E-02	3.45E-01
X-21803	-6.77E-04	5.15E-03	8.95E-01	-1.11E-03	1.18E-02	9.25E-01	-2.93E-03	2.77E-02	9.16E-01
X-21807	1.03E-02	4.55E-03	2.51E-02	2.57E-02	1.05E-02	1.47E-02	3.29E-02	2.47E-02	1.84E-01
X-21815	-4.24E-03	4.97E-03	3.94E-01	-2.24E-02	1.14E-02	5.00E-02	-4.02E-02	2.67E-02	1.34E-01
X-21816	-6.82E-03	4.76E-03	1.53E-01	-5.23E-03	1.10E-02	6.35E-01	-3.66E-02	2.57E-02	1.55E-01
X-21821	2.26E-02	5.19E-03	1.79E-05	5.01E-02	1.20E-02	3.76E-05	1.02E-01	2.82E-02	3.80E-04
X-21830	6.56E-03	4.81E-03	1.73E-01	1.26E-02	1.11E-02	2.55E-01	-3.17E-03	2.60E-02	9.03E-01
X-21831	9.60E-03	4.97E-03	5.45E-02	2.35E-02	1.14E-02	4.07E-02	2.05E-02	2.70E-02	4.48E-01
X-21834	1.29E-02	5.93E-03	3.09E-02	1.53E-02	1.37E-02	2.66E-01	6.59E-02	3.20E-02	4.02E-02
X-21838	-4.99E-03	3.87E-03	1.99E-01	2.94E-03	8.93E-03	7.42E-01	-1.64E-02	2.09E-02	4.32E-01
X-21839	3.74E-03	5.33E-03	4.83E-01	-1.08E-02	1.23E-02	3.77E-01	1.25E-02	2.87E-02	6.64E-01
X-21840	8.51E-03	5.27E-03	1.07E-01	1.38E-02	1.22E-02	2.57E-01	4.19E-02	2.84E-02	1.41E-01
X-21842	2.26E-02	4.93E-03	6.81E-06	5.21E-02	1.14E-02	6.88E-06	9.35E-02	2.70E-02	6.20E-04
X-21845	-2.68E-03	5.28E-03	6.12E-01	-3.25E-02	1.20E-02	7.27E-03	7.07E-04	2.85E-02	9.80E-01
X-21851	-6.93E-03	5.22E-03	1.85E-01	-9.32E-03	1.20E-02	4.40E-01	-4.83E-02	2.81E-02	8.62E-02
X-22162	1.32E-02	5.61E-03	1.90E-02	2.47E-02	1.30E-02	5.76E-02	8.04E-02	3.01E-02	8.09E-03
X-22509	1.89E-02	5.23E-03	3.74E-04	2.10E-02	1.23E-02	8.84E-02	7.84E-02	2.85E-02	6.28E-03
X-22771	-9.83E-03	5.87E-03	9.51E-02	-5.03E-02	1.32E-02	1.80E-04	-5.22E-02	3.16E-02	9.98E-02
X-22776	-8.64E-04	5.97E-03	8.85E-01	-1.56E-02	1.37E-02	2.55E-01	-4.42E-03	3.22E-02	8.91E-01
X-22834	-4.22E-03	5.82E-03	4.69E-01	-2.67E-03	1.34E-02	8.42E-01	-6.27E-02	3.12E-02	4.53E-02
X-23276	1.33E-03	5.52E-03	8.09E-01	-6.46E-03	1.27E-02	6.11E-01	-5.94E-03	2.97E-02	8.42E-01
X-23481	-2.02E-03	5.79E-03	7.27E-01	2.58E-03	1.33E-02	8.46E-01	-3.61E-02	3.11E-02	2.46E-01
X-23587	-1.02E-03	5.29E-03	8.48E-01	-3.34E-02	1.20E-02	5.81E-03	3.28E-02	2.84E-02	2.50E-01
X-23593	-7.53E-04	6.00E-03	9.00E-01	1.57E-02	1.38E-02	2.55E-01	5.08E-03	3.23E-02	8.75E-01
X-23636	-4.49E-03	6.01E-03	4.56E-01	-2.29E-02	1.38E-02	9.80E-02	-6.18E-02	3.22E-02	5.61E-02
X-23639	4.52E-03	5.63E-03	4.23E-01	-4.88E-04	1.30E-02	9.70E-01	4.89E-02	3.02E-02	1.07E-01
X-23641	2.24E-03	5.33E-03	6.75E-01	-3.88E-04	1.23E-02	9.75E-01	-2.54E-02	2.87E-02	3.76E-01
X-23644	1.93E-02	5.89E-03	1.21E-03	5.60E-02	1.34E-02	3.99E-05	7.95E-02	3.20E-02	1.36E-02
X-23654	4.49E-03	5.84E-03	4.42E-01	1.79E-02	1.34E-02	1.83E-01	8.40E-03	3.15E-02	7.90E-01
X-23659	1.87E-02	5.36E-03	5.64E-04	5.06E-02	1.22E-02	4.72E-05	7.87E-02	2.92E-02	7.35E-03
X-23678	-7.60E-03	5.59E-03	1.75E-01	-1.40E-02	1.29E-02	2.77E-01	-2.81E-02	3.02E-02	3.52E-01
X-23680	-1.60E-02	6.03E-03	8.39E-03	-1.97E-02	1.40E-02	1.61E-01	-5.96E-02	3.27E-02	6.98E-02
X-23739	-5.48E-04	5.70E-03	9.24E-01	-6.65E-03	1.31E-02	6.13E-01	-4.88E-06	3.07E-02	1.00E+00
X-23767	-1.53E-02	4.55E-03	8.59E-04	-1.84E-02	1.06E-02	8.49E-02	-2.37E-02	2.50E-02	3.44E-01
X-23780	8.68E-03	5.39E-03	1.08E-01	1.05E-02	1.24E-02	3.99E-01	5.32E-02	2.90E-02	6.78E-02
X-23782	4.64E-03	5.44E-03	3.95E-01	4.61E-04	1.25E-02	9.71E-01	7.97E-02	2.90E-02	6.36E-03
X-23787	-1.92E-02	5.57E-03	6.77E-04	-3.84E-02	1.29E-02	3.16E-03	-8.29E-02	3.03E-02	6.58E-03
X-23890	7.09E-03	4.32E-03	1.02E-01	2.19E-02	9.90E-03	2.77E-02	9.90E-03	2.34E-02	6.72E-01

X-23974	1.94E-03	5.57E-03	7.28E-01	9.12E-03	1.28E-02	4.77E-01	3.96E-02	2.99E-02	1.87E-01
X-23997	5.71E-03	5.86E-03	3.31E-01	1.34E-02	1.35E-02	3.23E-01	7.38E-03	3.17E-02	8.16E-01
X-24295	-7.99E-03	5.76E-03	1.66E-01	-1.01E-02	1.33E-02	4.47E-01	-3.24E-02	3.11E-02	2.99E-01
X-24306	1.47E-03	5.73E-03	7.97E-01	-1.21E-02	1.32E-02	3.60E-01	-2.40E-02	3.08E-02	4.37E-01
X-24307	-1.31E-03	5.86E-03	8.24E-01	-6.90E-03	1.35E-02	6.09E-01	-4.21E-02	3.15E-02	1.81E-01
X-24309	-1.26E-02	5.62E-03	2.55E-02	-1.59E-02	1.30E-02	2.22E-01	-7.81E-02	3.02E-02	1.02E-02
X-24328	-6.24E-03	4.85E-03	1.99E-01	-1.24E-02	1.12E-02	2.69E-01	-5.77E-02	2.60E-02	2.70E-02
X-24334	-6.78E-03	5.27E-03	2.00E-01	3.75E-04	1.22E-02	9.75E-01	-6.95E-02	2.82E-02	1.44E-02
X-24337	-1.38E-02	5.49E-03	1.23E-02	-1.01E-02	1.28E-02	4.28E-01	-9.66E-02	2.94E-02	1.12E-03
X-24338	2.29E-03	3.84E-03	5.52E-01	7.31E-03	8.84E-03	4.09E-01	-4.14E-03	2.07E-02	8.42E-01
X-24344	-3.57E-03	4.54E-03	4.33E-01	-1.46E-02	1.04E-02	1.62E-01	-1.50E-02	2.45E-02	5.41E-01
X-24352	1.35E-02	5.03E-03	7.63E-03	8.60E-03	1.17E-02	4.64E-01	3.15E-02	2.74E-02	2.51E-01
X-24414	-6.99E-04	4.10E-03	8.65E-01	-3.80E-03	9.44E-03	6.87E-01	-1.84E-02	2.21E-02	4.06E-01
X-24418	1.01E-02	5.76E-03	8.20E-02	-2.59E-03	1.33E-02	8.46E-01	2.99E-02	3.12E-02	3.38E-01
X-24456	-5.87E-04	5.00E-03	9.07E-01	1.41E-03	1.15E-02	9.03E-01	3.15E-02	2.69E-02	2.42E-01
X-24475	2.20E-02	5.50E-03	8.32E-05	4.39E-02	1.27E-02	6.62E-04	9.03E-02	3.00E-02	2.85E-03
X-24494	9.06E-03	5.87E-03	1.24E-01	9.47E-04	1.36E-02	9.44E-01	3.91E-02	3.17E-02	2.19E-01
X-24541	1.27E-02	5.05E-03	1.27E-02	1.51E-02	1.17E-02	1.98E-01	7.61E-02	2.72E-02	5.44E-03
X-24543	-8.46E-03	4.28E-03	4.94E-02	-1.60E-02	9.88E-03	1.08E-01	-4.28E-02	2.31E-02	6.53E-02
X-24544	-1.61E-02	5.36E-03	2.85E-03	-2.48E-02	1.24E-02	4.73E-02	-1.06E-01	2.86E-02	2.53E-04
X-24545	9.52E-03	5.32E-03	7.46E-02	6.84E-03	1.23E-02	5.79E-01	1.94E-02	2.88E-02	5.00E-01
X-24546	-1.12E-02	5.36E-03	3.67E-02	-2.19E-02	1.24E-02	7.73E-02	-9.01E-02	2.86E-02	1.80E-03
X-24556	-3.94E-04	5.74E-03	9.45E-01	1.72E-02	1.32E-02	1.91E-01	4.32E-02	3.08E-02	1.62E-01
X-24565	-7.56E-03	5.30E-03	1.55E-01	9.95E-03	1.22E-02	4.16E-01	-4.65E-02	2.85E-02	1.04E-01
X-24571	-6.99E-03	5.45E-03	2.01E-01	-1.76E-02	1.25E-02	1.62E-01	-2.60E-02	2.94E-02	3.78E-01
X-24576	2.70E-03	2.83E-03	3.42E-01	6.73E-03	6.51E-03	3.02E-01	3.48E-03	1.53E-02	8.20E-01
X-24588	-1.75E-02	5.42E-03	1.39E-03	-3.61E-02	1.25E-02	4.21E-03	-6.80E-02	2.95E-02	2.18E-02
X-24657	4.70E-03	4.91E-03	3.38E-01	1.64E-02	1.13E-02	1.46E-01	3.82E-03	2.65E-02	8.85E-01
X-24736	2.26E-02	5.18E-03	1.86E-05	3.52E-02	1.21E-02	3.97E-03	1.05E-01	2.82E-02	2.43E-04
X-24747	4.38E-04	5.70E-03	9.39E-01	1.91E-02	1.31E-02	1.45E-01	1.28E-02	3.07E-02	6.77E-01
X-24748	8.21E-03	6.16E-03	1.84E-01	1.42E-02	1.42E-02	3.18E-01	5.03E-02	3.32E-02	1.30E-01
X-24757	1.41E-02	5.25E-03	7.86E-03	4.06E-02	1.20E-02	8.17E-04	3.50E-02	2.86E-02	2.22E-01
X-24761	1.83E-03	3.89E-03	6.38E-01	6.32E-03	8.95E-03	4.81E-01	1.98E-02	2.09E-02	3.44E-01
X-24762	-8.49E-03	5.23E-03	1.06E-01	-1.37E-02	1.21E-02	2.57E-01	-4.90E-02	2.82E-02	8.35E-02
X-24947	-1.44E-02	5.40E-03	7.92E-03	-3.14E-02	1.24E-02	1.21E-02	-6.16E-02	2.92E-02	3.62E-02
X-24949	-1.37E-02	5.97E-03	2.28E-02	-3.16E-02	1.37E-02	2.21E-02	-2.80E-02	3.24E-02	3.89E-01
X-24951	-1.27E-02	5.79E-03	2.96E-02	-3.77E-02	1.32E-02	4.73E-03	-6.90E-02	3.12E-02	2.76E-02
X-24953	-9.23E-03	5.63E-03	1.02E-01	-2.31E-02	1.30E-02	7.50E-02	-4.72E-02	3.04E-02	1.21E-01
X-24970	-1.42E-03	5.79E-03	8.06E-01	-9.03E-03	1.33E-02	4.98E-01	-2.59E-02	3.12E-02	4.06E-01
X-24980	3.86E-03	5.25E-03	4.63E-01	2.10E-02	1.20E-02	8.19E-02	-2.12E-02	2.83E-02	4.56E-01
X-25009	-7.12E-03	5.97E-03	2.34E-01	7.09E-03	1.38E-02	6.07E-01	9.84E-04	3.23E-02	9.76E-01
X-25172	4.50E-04	6.04E-03	9.41E-01	2.93E-04	1.39E-02	9.83E-01	-1.56E-02	3.26E-02	6.32E-01
X-25217	4.19E-03	5.21E-03	4.22E-01	4.20E-03	1.20E-02	7.27E-01	6.70E-03	2.81E-02	8.12E-01
X-25247	1.32E-03	4.80E-03	7.84E-01	2.04E-02	1.10E-02	6.40E-02	1.51E-02	2.59E-02	5.60E-01
X-25271	1.60E-02	5.96E-03	7.48E-03	3.16E-02	1.38E-02	2.22E-02	3.65E-02	3.24E-02	2.61E-01
X-25279	6.68E-03	4.63E-03	1.51E-01	-6.69E-03	1.07E-02	5.32E-01	1.43E-02	2.50E-02	5.70E-01
X-25343	-5.62E-03	6.00E-03	3.50E-01	-1.63E-02	1.38E-02	2.38E-01	-3.61E-02	3.23E-02	2.66E-01
X-25371	-7.52E-03	5.52E-03	1.74E-01	1.26E-02	1.27E-02	3.24E-01	-8.64E-02	2.94E-02	3.56E-03
X-25417	2.38E-03	4.62E-03	6.08E-01	-2.32E-02	1.06E-02	2.85E-02	3.41E-02	2.48E-02	1.71E-01
X-25419	5.24E-03	5.27E-03	3.21E-01	-3.12E-02	1.20E-02	9.91E-03	8.13E-02	2.81E-02	4.05E-03
X-25420	1.93E-04	5.33E-03	9.71E-01	-2.03E-03	1.23E-02	8.69E-01	-1.54E-02	2.87E-02	5.92E-01
X-25422	-1.60E-02	5.53E-03	4.09E-03	-3.88E-02	1.27E-02	2.49E-03	-5.79E-02	3.00E-02	5.49E-02
X-25433	-8.69E-03	5.47E-03	1.13E-01	-1.65E-02	1.26E-02	1.93E-01	-8.55E-02	2.92E-02	3.62E-03
X-25450	-2.33E-03	4.04E-03	5.66E-01	8.76E-03	9.30E-03	3.47E-01	-1.04E-02	2.18E-02	6.32E-01
X-25454	4.10E-03	5.73E-03	4.75E-01	1.58E-02	1.32E-02	2.32E-01	-3.63E-02	3.08E-02	2.40E-01
X-25457	-4.43E-03	4.88E-03	3.65E-01	5.83E-03	1.13E-02	6.05E-01	1.10E-02	2.64E-02	6.78E-01
X-25468	-4.62E-04	3.49E-03	8.95E-01	1.37E-02	7.99E-03	8.69E-02	-1.01E-02	1.88E-02	5.91E-01
X-25519	-1.24E-02	5.70E-03	3.06E-02	-2.11E-03	1.32E-02	8.73E-01	-2.59E-02	3.10E-02	4.03E-01
X-25520	6.37E-03	5.73E-03	2.67E-01	1.20E-02	1.32E-02	3.65E-01	3.28E-02	3.09E-02	2.89E-01
X-25524	1.14E-02	4.09E-03	5.67E-03	2.09E-02	9.47E-03	2.83E-02	4.31E-02	2.22E-02	5.35E-02
X-25656	7.08E-03	5.39E-03	1.90E-01	2.50E-03	1.24E-02	8.41E-01	2.45E-02	2.91E-02	4.01E-01
X-25790	3.09E-03	5.39E-03	5.67E-01	1.24E-02	1.24E-02	3.19E-01	1.57E-02	2.91E-02	5.90E-01
X-25805	-2.79E-03	4.32E-03	5.19E-01	-6.08E-03	9.94E-03	5.42E-01	-2.24E-03	2.33E-02	9.23E-01
X-25810	-6.60E-04	5.98E-03	9.12E-01	-9.32E-03	1.38E-02	4.99E-01	5.49E-02	3.21E-02	8.79E-02
X-25937	-1.40E-02	5.24E-03	7.91E-03	-1.84E-02	1.22E-02	1.30E-01	-5.73E-02	2.84E-02	4.43E-02
X-25957	1.24E-02	5.78E-03	3.26E-02	2.90E-02	1.33E-02	3.02E-02	7.81E-02	3.10E-02	1.25E-02
X-25991	9.88E-03	5.12E-03	5.43E-02	2.87E-02	1.17E-02	1.51E-02	4.59E-02	2.76E-02	9.80E-02
X-26054	-6.54E-05	5.87E-03	9.91E-01	1.17E-02	1.35E-02	3.85E-01	-2.84E-03	3.16E-02	9.29E-01
X-26062	5.84E-03	5.06E-03	2.49E-01	-5.82E-02	1.11E-02	3.45E-07	3.76E-02	2.72E-02	1.68E-01
X-26106	-5.19E-03	5.61E-03	3.56E-01	-1.68E-04	1.29E-02	9.90E-01	-3.13E-02	3.02E-02	3.02E-01
X-26107	-4.85E-03	5.60E-03	3.87E-01	-5.35E-03	1.29E-02	6.79E-01	-3.22E-02	3.02E-02	2.86E-01
X-26108	-8.00E-03	5.62E-03	1.56E-01	-1.53E-02	1.29E-02	2.39E-01	-4.50E-02	3.03E-02	1.38E-01
X-26109	-1.20E-02	5.15E-03	2.09E-02	-1.62E-02	1.19E-02	1.75E-01	-8.27E-02	2.76E-02	2.98E-03
X-26111	-1.83E-02	6.11E-03	2.95E-03	-5.91E-02	1.38E-02	2.61E-05	-7.72E-02	3.31E-02	2.05E-02
X-26119	-3.12E-03	5.96E-03	6.01E-01	-1.24E-03	1.37E-02	9.28E-01	-3.57E-02	3.21E-02	2.66E-01

Abbreviations : AMED: Mediterranean Style diet, DASH: Dietary Approaches to Stop Hypertension; HEI-15: Healthy eating index (2015)

Note : Metabolite concentrations transformed prior to analysis..

Note : All models controlled for age, sex, race, physical activity, education level, energy intake and smoking status

Supplementary Table 3: Pearson Correlations Between All Metabolites Associated with at Least One Diet Pattern

Supplementary Table 3: Pearson Correlations Between All Metabolites Associated with at Least One Diet Pattern

	tartarate	4-acetylphenol sulfate	tartronate (hydroxymalonate)	oxalate (ethanedioate)	beta-sitosterol	indolepropionate	pyridoxate	adrenate (22:4n6)	10-heptadecenoate (17:1n7)	1-arachidoyl-GPC (20:0)	catechol sulfate
tartarate	1.00	0.16	0.57	0.63	0.10	0.40	0.35	-0.08	0.03	0.19	0.28
4-acetylphenol sulfate	0.16	1.00	0.11	0.13	0.15	0.21	0.19	-0.25	-0.23	0.31	0.48
tartronate (hydroxymalonate)	0.57	0.11	1.00	0.88	0.23	0.41	0.48	-0.14	0.05	0.38	0.28
oxalate (ethanedioate)	0.63	0.13	0.88	1.00	0.21	0.35	0.48	-0.18	-0.01	0.32	0.26
beta-sitosterol	0.10	0.15	0.23	0.21	1.00	0.20	0.05	-0.24	-0.21	0.44	0.29
indolepropionate	0.40	0.21	0.41	0.35	0.20	1.00	0.31	-0.18	-0.05	0.27	0.43
pyridoxate	0.35	0.19	0.48	0.48	0.05	0.31	1.00	-0.15	-0.01	0.21	0.28
adrenate (22:4n6)	-0.08	-0.25	-0.14	-0.18	-0.24	-0.18	-0.15	1.00	0.78	-0.31	-0.25
10-heptadecenoate (17:1n7)	0.03	-0.23	0.05	-0.01	-0.21	-0.05	-0.01	0.78	1.00	-0.21	-0.15
1-arachidoyl-GPC (20:0)	0.19	0.31	0.38	0.32	0.44	0.27	0.21	-0.31	-0.21	1.00	0.42
catechol sulfate	0.28	0.48	0.28	0.26	0.29	0.43	0.28	-0.25	-0.15	0.42	1.00
N-methylproline	0.13	0.12	0.17	0.19	0.19	0.21	0.14	-0.14	-0.10	0.10	0.18
beta-cryptoxanthin	0.36	0.15	0.39	0.42	0.29	0.37	0.14	-0.20	-0.10	0.25	0.26
androstenediol (3beta,17beta) disulfate (1)	-0.23	-0.08	-0.36	-0.34	-0.18	-0.22	-0.22	0.18	0.00	-0.20	-0.17
S-methylmethionine	0.16	0.18	0.34	0.33	0.24	0.29	0.15	-0.22	-0.15	0.32	0.26
tridecenedioate (C13:1-DC)*	0.00	-0.15	0.01	0.00	-0.19	0.00	0.07	0.37	0.45	-0.18	-0.06
1-behenoyl-GPC (22:0)	0.21	0.31	0.35	0.33	0.39	0.22	0.22	-0.22	-0.13	0.86	0.39
1-erucoyl-GPC (22:1)*	0.20	0.23	0.42	0.35	0.24	0.25	0.32	-0.14	-0.03	0.58	0.31
1-lignoceroyl-GPC (24:0)	0.22	0.26	0.38	0.31	0.29	0.22	0.24	-0.13	-0.07	0.70	0.34
1-nervonoyl-GPC (24:1n9)*	0.20	0.14	0.37	0.28	0.14	0.22	0.23	-0.02	0.10	0.50	0.27
(14 or 15)-methylpalmitate (a17:0 or i17:0)	0.01	-0.16	0.05	0.01	-0.22	-0.03	0.02	0.68	0.88	-0.21	-0.09
guaiacol sulfate	0.22	0.45	0.14	0.15	0.17	0.25	0.30	-0.12	-0.06	0.27	0.78
N-delta-acetylornithine	0.35	0.29	0.36	0.35	0.25	0.35	0.40	-0.10	0.08	0.35	0.42
1-eicosenoyl-GPC (20:1)*	0.22	0.25	0.38	0.32	0.35	0.26	0.22	-0.22	-0.11	0.85	0.37
methyl glucopyranoside (alpha + beta)	0.46	0.23	0.50	0.46	0.24	0.42	0.28	-0.14	-0.04	0.27	0.37
4-methoxyphenol sulfate	0.26	0.34	0.20	0.22	0.14	0.28	0.23	-0.14	-0.06	0.24	0.34
1-palmitoyl-2-arachidonoyl-GPC (16:0/20:4n6)	-0.17	-0.19	-0.21	-0.18	-0.15	-0.29	-0.18	0.32	0.15	-0.20	-0.30
sphingomyelin (d18:0/18:0, d19:0/17:0)*	-0.14	-0.18	-0.05	-0.05	-0.18	-0.21	0.03	0.21	0.20	-0.33	-0.21
1-stearoyl-2-docosapentaenoyl-GPC (18:0/22:5n6)*	-0.22	-0.14	-0.36	-0.31	-0.25	-0.36	-0.39	0.25	0.09	-0.39	-0.32
1-(1-enyl-oleoyl)-2-docosahexaenoyl-GPE (P-18:1/22:6)*	0.23	-0.03	0.30	0.37	0.29	0.12	0.28	-0.17	-0.13	0.18	0.06
ethyl alpha-glucopyranoside	-0.07	0.03	-0.14	-0.18	-0.01	-0.14	-0.16	0.13	0.09	-0.01	-0.05
carotene diol (1)	0.35	0.12	0.40	0.42	0.29	0.29	0.24	-0.16	-0.11	0.27	0.27
carotene diol (2)	0.33	0.14	0.37	0.40	0.30	0.28	0.16	-0.20	-0.15	0.23	0.24
heptenedioate (C7:1-DC)*	-0.04	-0.10	-0.01	-0.01	-0.21	-0.09	0.15	0.31	0.38	-0.21	-0.06
1-(1-enyl-oleoyl)-2-docosahexaenoyl-GPC (P-18:1/22:6)*	0.21	-0.01	0.36	0.36	0.31	0.15	0.28	-0.11	-0.02	0.28	0.08
glycine conjugate of C10H14O2 (1)*	-0.08	-0.21	-0.20	-0.13	-0.09	-0.25	-0.08	0.12	0.09	-0.24	-0.19
2,6-dihydroxybenzoic acid	0.31	0.26	0.27	0.24	0.19	0.38	0.34	0.01	0.04	0.27	0.38
3,5-dichloro-2,6-dihydroxybenzoic acid	-0.20	-0.15	-0.29	-0.24	-0.14	-0.16	-0.08	0.12	0.06	-0.28	-0.16
branched chain 14:0 dicarboxylic acid**	0.42	0.26	0.47	0.42	0.25	0.51	0.28	-0.20	-0.07	0.27	0.44
pentose acid*	0.53	0.35	0.38	0.36	0.19	0.41	0.31	-0.19	-0.09	0.31	0.48
3-bromo-5-chloro-2,6-dihydroxybenzoic acid*	-0.14	-0.16	-0.18	-0.17	-0.16	-0.13	0.00	0.22	0.23	-0.28	-0.11
glycerate	0.53	0.11	0.94	0.87	0.20	0.37	0.49	-0.17	0.01	0.39	0.25
cystine	-0.34	-0.15	-0.70	-0.60	-0.22	-0.30	-0.27	0.23	0.13	-0.39	-0.21
N-stearoyl-sphinganine (d18:0/18:0)*	-0.14	-0.18	-0.10	-0.08	-0.28	-0.26	0.05	0.30	0.28	-0.46	-0.21
X-11315	0.44	0.19	0.54	0.53	0.29	0.41	0.36	-0.17	-0.06	0.30	0.44
X-12306	0.19	0.34	0.24	0.21	0.23	0.50	0.15	-0.29	-0.21	0.33	0.53
X-12680	0.21	0.32	0.09	0.15	0.13	0.10	0.17	-0.14	-0.16	0.17	0.24
X-12726	0.31	0.46	0.25	0.26	0.25	0.39	0.23	-0.30	-0.17	0.34	0.60
X-14056	-0.41	-0.09	-0.75	-0.65	-0.25	-0.36	-0.35	0.18	0.05	-0.42	-0.18
X-17351	0.41	0.20	0.31	0.27	0.18	0.85	0.29	-0.02	0.07	0.19	0.39
X-21339	-0.21	-0.20	-0.32	-0.26	-0.03	-0.18	-0.23	0.04	-0.03	-0.30	-0.28
X-21736	-0.05	-0.06	-0.10	-0.09	-0.13	-0.16	0.05	0.28	0.32	-0.18	-0.10
X-21752	0.26	0.09	0.25	0.24	0.11	0.36	0.24	-0.18	-0.07	0.20	0.27
X-21842	0.25	0.26	0.25	0.25	0.21	0.40	0.23	-0.24	-0.18	0.26	0.40
X-26062	0.08	0.07	0.01	-0.07	-0.02	-0.01	-0.06	0.07	0.08	0.05	0.02

	N-methylproline	beta-cryptoxanthin	androstenediol (3beta,17beta) disulfate (1)	S-methylmethionine	tridecenedioate (C13:1-DC)*	1-behenoyl-GPC (22:0)	1-erucoyl-GPC (22:1)*	1-lignoceroyl-GPC (24:0)	1-nervonoyl-GPC (24:1n9)*	(14 or 15)-methylpalmitate (a17:0 or i17:0)	guaiacol sulfate
tartarate	0.13	0.36	-0.23	0.16	0.00	0.21	0.20	0.22	0.20	0.01	0.22
4-acetylphenol sulfate	0.12	0.15	-0.08	0.18	-0.15	0.31	0.23	0.26	0.14	-0.16	0.45
tartronate (hydroxymalonate)	0.17	0.39	-0.36	0.34	0.01	0.35	0.42	0.38	0.37	0.05	0.14
oxalate (ethanedioate)	0.19	0.42	-0.34	0.33	0.00	0.33	0.35	0.31	0.28	0.01	0.15
beta-sitosterol	0.19	0.29	-0.18	0.24	-0.19	0.39	0.24	0.29	0.14	-0.22	0.17
indolepropionate	0.21	0.37	-0.22	0.29	0.00	0.22	0.25	0.22	0.22	-0.03	0.25
pyridoxate	0.14	0.14	-0.22	0.15	0.07	0.22	0.32	0.24	0.23	0.02	0.30
adrenate (22:4n6)	-0.14	-0.20	0.18	-0.22	0.37	-0.22	-0.14	-0.13	-0.02	0.68	-0.12
10-heptadecenoate (17:1n7)	-0.10	-0.10	0.00	-0.15	0.45	-0.13	-0.03	-0.07	0.10	0.88	-0.06
1-arachidoyl-GPC (20:0)	0.10	0.25	-0.20	0.32	-0.18	0.86	0.58	0.70	0.50	-0.21	0.27
catechol sulfate	0.18	0.26	-0.17	0.26	-0.06	0.39	0.31	0.34	0.27	-0.09	0.78
N-methylproline	1.00	0.39	-0.08	0.16	-0.12	0.07	0.06	0.05	0.00	-0.10	0.16
beta-cryptoxanthin	0.39	1.00	-0.31	0.31	-0.06	0.28	0.15	0.20	0.20	-0.07	0.11
androstenediol (3beta,17beta) disulfate (1)	-0.08	-0.31	1.00	-0.20	0.08	-0.24	-0.20	-0.27	-0.20	0.04	-0.06
S-methylmethionine	0.16	0.31	-0.20	1.00	-0.13	0.29	0.25	0.25	0.19	-0.10	0.15
tridecenedioate (C13:1-DC)*	-0.12	-0.06	0.08	-0.13	1.00	-0.12	-0.02	-0.07	-0.05	0.57	-0.02
1-behenoyl-GPC (22:0)	0.07	0.28	-0.24	0.29	-0.12	1.00	0.49	0.76	0.47	-0.12	0.27
1-erucoyl-GPC (22:1)*	0.06	0.15	-0.20	0.25	-0.02	0.49	1.00	0.50	0.63	-0.05	0.26
1-lignoceroyl-GPC (24:0)	0.05	0.20	-0.27	0.25	-0.07	0.76	0.50	1.00	0.55	-0.06	0.26
1-nervonoyl-GPC (24:1n9)*	0.00	0.20	-0.20	0.19	-0.05	0.47	0.63	0.55	1.00	0.07	0.19
(14 or 15)-methylpalmitate (a17:0 or i17:0)	-0.10	-0.07	0.04	-0.10	0.57	-0.12	-0.05	-0.06	0.07	1.00	-0.04
guaiacol sulfate	0.16	0.11	-0.06	0.15	-0.02	0.27	0.26	0.26	0.19	-0.04	1.00
N-delta-acetylornithine	0.17	0.26	-0.11	0.27	0.07	0.38	0.24	0.31	0.29	0.08	0.34
1-eicosenoyl-GPC (20:1)*	0.09	0.23	-0.18	0.27	-0.12	0.68	0.68	0.61	0.62	-0.12	0.24
methyl glucopyranoside (alpha + beta)	0.44	0.47	-0.14	0.31	-0.09	0.23	0.30	0.31	0.31	-0.05	0.28
4-methoxyphenol sulfate	0.09	0.14	-0.15	0.19	0.01	0.27	0.17	0.23	0.21	0.01	0.24
1-palmitoyl-2-arachidonoyl-GPC (16:0/20:4n6)	-0.15	-0.11	0.19	-0.23	0.07	-0.16	-0.08	-0.10	-0.05	0.08	-0.20
sphingomyelin (d18:0/18:0, d19:0/17:0)*	-0.07	-0.13	0.10	-0.14	0.11	-0.31	-0.04	-0.27	-0.06	0.22	-0.15
1-stearoyl-2-docosapentaenoyl-GPC (18:0/22:5n6)*	-0.13	-0.17	0.20	-0.33	0.06	-0.39	-0.34	-0.32	-0.26	0.07	-0.27
1-(1-enyl-oleoyl)-2-docosahexaenoyl-GPE (P-18:1/22:6)*	0.13	0.42	-0.30	0.25	-0.23	0.26	0.21	0.17	0.19	-0.19	0.05
ethyl alpha-glucopyranoside	-0.20	-0.19	0.28	-0.08	0.02	0.00	0.14	0.09	0.16	0.09	0.00
carotene diol (1)	0.14	0.60	-0.26	0.33	-0.05	0.40	0.25	0.28	0.22	-0.10	0.15
carotene diol (2)	0.14	0.58	-0.27	0.32	-0.10	0.32	0.24	0.21	0.18	-0.13	0.14
heptenedioate (C7:1-DC)*	-0.12	-0.13	0.01	-0.12	0.80	-0.16	-0.06	-0.11	-0.09	0.51	-0.01
1-(1-enyl-oleoyl)-2-docosahexaenoyl-GPC (P-18:1/22:6)*	0.10	0.37	-0.44	0.15	-0.16	0.33	0.28	0.27	0.30	-0.09	0.03
glycine conjugate of C10H14O2 (1)*	-0.12	-0.16	0.15	-0.03	0.12	-0.24	-0.27	-0.28	-0.25	0.07	-0.08
2,6-dihydroxybenzoic acid	0.09	0.16	-0.03	0.14	0.06	0.22	0.17	0.25	0.20	0.05	0.34
3,5-dichloro-2,6-dihydroxybenzoic acid	-0.06	-0.24	0.15	-0.16	0.20	-0.28	-0.29	-0.37	-0.36	0.09	-0.12
branched chain 14:0 dicarboxylic acid**	0.42	0.47	-0.24	0.35	-0.08	0.29	0.31	0.27	0.25	-0.04	0.33
pentose acid*	0.28	0.27	-0.14	0.39	-0.10	0.27	0.33	0.32	0.33	-0.05	0.44
3-bromo-5-chloro-2,6-dihydroxybenzoic acid*	-0.09	-0.18	0.10	-0.14	0.32	-0.26	-0.20	-0.32	-0.22	0.31	-0.04
glycerate	0.21	0.35	-0.36	0.32	-0.02	0.35	0.43	0.35	0.33	0.01	0.12
cystine	-0.12	-0.26	0.27	-0.23	0.15	-0.33	-0.36	-0.31	-0.32	0.10	-0.07
N-stearoyl-sphinganine (d18:0/18:0)*	-0.05	-0.23	0.14	-0.20	0.26	-0.43	-0.14	-0.33	-0.16	0.34	-0.11
X-11315	0.19	0.34	-0.21	0.33	-0.04	0.33	0.28	0.32	0.27	-0.01	0.34
X-12306	0.16	0.31	-0.17	0.32	-0.17	0.32	0.29	0.27	0.25	-0.17	0.31
X-12680	0.14	0.10	-0.09	0.15	-0.09	0.15	0.07	0.12	0.01	-0.13	0.26
X-12726	0.22	0.27	-0.11	0.37	-0.10	0.35	0.26	0.29	0.23	-0.07	0.52
X-14056	-0.12	-0.36	0.35	-0.25	0.03	-0.36	-0.36	-0.37	-0.33	0.08	-0.02
X-17351	0.28	0.29	-0.15	0.23	0.03	0.16	0.18	0.18	0.14	0.06	0.32
X-21339	-0.09	-0.21	0.23	-0.15	0.09	-0.33	-0.38	-0.50	-0.44	-0.04	-0.26
X-21736	-0.12	-0.18	0.06	-0.13	0.30	-0.12	0.00	-0.14	-0.05	0.30	0.04
X-21752	0.17	0.28	-0.29	0.09	0.05	0.19	0.19	0.24	0.13	-0.06	0.21
X-21842	0.26	0.38	-0.22	0.28	-0.09	0.27	0.20	0.23	0.21	-0.10	0.30
X-26062	0.02	-0.06	0.26	-0.05	-0.02	0.02	0.18	0.14	0.25	0.06	0.05

	N-delta-acetylornithine	1-eicosenoyl-GPC (20:1)*	methyl glucopyranoside (alpha + beta)	4-methoxyphenol sulfate	1-palmitoyl-2-arachidonoyl-GPC (16:0/20:4n6)	sphingomyelin (d18:0/18:0, d19:0/17:0)*	1-stearoyl-2-docosapentaenoyl-GPC (18:0/22:5n6)*	1-(1-enyl-oleoyl)-2-docosahexaenoyl-GPE (P-18:1/22:6)*	ethyl alpha-glucopyranoside	carotene diol (1)	carotene diol (2)
tartarate	0.35	0.22	0.46	0.26	-0.17	-0.14	-0.22	0.23	-0.07	0.35	0.33
4-acetylphenol sulfate	0.29	0.25	0.23	0.34	-0.19	-0.18	-0.14	-0.03	0.03	0.12	0.14
tartronate (hydroxymalonate)	0.36	0.38	0.50	0.20	-0.21	-0.05	-0.36	0.30	-0.14	0.40	0.37
oxalate (ethanedioate)	0.35	0.32	0.46	0.22	-0.18	-0.05	-0.31	0.37	-0.18	0.42	0.40
beta-sitosterol	0.25	0.35	0.24	0.14	-0.15	-0.18	-0.25	0.29	-0.01	0.29	0.30
indolepropionate	0.35	0.26	0.42	0.28	-0.29	-0.21	-0.36	0.12	-0.14	0.29	0.28
pyridoxate	0.40	0.22	0.28	0.23	-0.18	0.03	-0.39	0.28	-0.16	0.24	0.16
adrenate (22:4n6)	-0.10	-0.22	-0.14	-0.14	0.32	0.21	0.25	-0.17	0.13	-0.16	-0.20
10-heptadecenoate (17:1n7)	0.08	-0.11	-0.04	-0.06	0.15	0.20	0.09	-0.13	0.09	-0.11	-0.15
1-arachidoyl-GPC (20:0)	0.35	0.85	0.27	0.24	-0.20	-0.33	-0.39	0.18	-0.01	0.27	0.23
catechol sulfate	0.42	0.37	0.37	0.34	-0.30	-0.21	-0.32	0.06	-0.05	0.27	0.24
N-methylproline	0.17	0.09	0.44	0.09	-0.15	-0.07	-0.13	0.13	-0.20	0.14	0.14
beta-cryptoxanthin	0.26	0.23	0.47	0.14	-0.11	-0.13	-0.17	0.42	-0.19	0.60	0.58
androstenediol (3beta,17beta) disulfate (1)	-0.11	-0.18	-0.14	-0.15	0.19	0.10	0.20	-0.30	0.28	-0.26	-0.27
S-methylmethionine	0.27	0.27	0.31	0.19	-0.23	-0.14	-0.33	0.25	-0.08	0.33	0.32
tridecenedioate (C13:1-DC)*	0.07	-0.12	-0.09	0.01	0.07	0.11	0.06	-0.23	0.02	-0.05	-0.10
1-behenoyl-GPC (22:0)	0.38	0.68	0.23	0.27	-0.16	-0.31	-0.39	0.26	0.00	0.40	0.32
1-erucoyl-GPC (22:1)*	0.24	0.68	0.30	0.17	-0.08	-0.04	-0.34	0.21	0.14	0.25	0.24
1-lignoceroyl-GPC (24:0)	0.31	0.61	0.31	0.23	-0.10	-0.27	-0.32	0.17	0.09	0.28	0.21
1-nervonoyl-GPC (24:1n9)*	0.29	0.62	0.31	0.21	-0.05	-0.06	-0.26	0.19	0.16	0.22	0.18
(14 or 15)-methylpalmitate (a17:0 or i17:0)	0.08	-0.12	-0.05	0.01	0.08	0.22	0.07	-0.19	0.09	-0.10	-0.13
guaiacol sulfate	0.34	0.24	0.28	0.24	-0.20	-0.15	-0.27	0.05	0.00	0.15	0.14
N-delta-acetylornithine	1.00	0.29	0.41	0.29	-0.19	-0.14	-0.41	0.19	-0.03	0.25	0.17
1-eicosenoyl-GPC (20:1)*	0.29	1.00	0.30	0.23	-0.13	-0.25	-0.32	0.14	0.04	0.24	0.21
methyl glucopyranoside (alpha + beta)	0.41	0.30	1.00	0.26	-0.16	-0.16	-0.25	0.13	-0.08	0.36	0.35
4-methoxyphenol sulfate	0.29	0.23	0.26	1.00	-0.22	-0.17	-0.25	0.03	0.02	0.13	0.12
1-palmitoyl-2-arachidonoyl-GPC (16:0/20:4n6)	-0.19	-0.13	-0.16	-0.22	1.00	0.34	0.47	-0.03	0.15	0.07	-0.01
sphingomyelin (d18:0/18:0, d19:0/17:0)*	-0.14	-0.25	-0.16	-0.17	0.34	1.00	0.16	0.07	0.01	-0.01	-0.02
1-stearoyl-2-docosapentaenoyl-GPC (18:0/22:5n6)*	-0.41	-0.32	-0.25	-0.25	0.47	0.16	1.00	-0.37	0.06	-0.25	-0.21
1-(1-enyl-oleoyl)-2-docosahexaenoyl-GPE (P-18:1/22:6)*	0.19	0.14	0.13	0.03	-0.03	0.07	-0.37	1.00	-0.17	0.54	0.56
ethyl alpha-glucopyranoside	-0.03	0.04	-0.08	0.02	0.15	0.01	0.06	-0.17	1.00	-0.09	-0.12
carotene diol (1)	0.25	0.24	0.36	0.13	0.07	-0.01	-0.25	0.54	-0.09	1.00	0.89
carotene diol (2)	0.17	0.21	0.35	0.12	-0.01	-0.02	-0.21	0.56	-0.12	0.89	1.00
heptenedioate (C7:1-DC)*	0.04	-0.14	-0.13	0.00	0.04	0.22	0.03	-0.18	-0.05	-0.06	-0.09
1-(1-enyl-oleoyl)-2-docosahexaenoyl-GPC (P-18:1/22:6)*	0.15	0.25	0.11	0.07	0.00	0.10	-0.35	0.80	-0.21	0.46	0.49
glycine conjugate of C10H14O2 (1)*	-0.05	-0.31	-0.20	-0.12	0.09	0.09	0.04	0.05	0.10	-0.10	-0.14
2,6-dihydroxybenzoic acid	0.30	0.24	0.34	0.54	-0.11	-0.18	-0.19	-0.02	-0.04	0.15	0.15
3,5-dichloro-2,6-dihydroxybenzoic acid	-0.17	-0.28	-0.29	-0.14	0.11	0.13	0.21	-0.06	-0.14	-0.18	-0.17
branched chain 14:0 dicarboxylic acid**	0.34	0.29	0.59	0.29	-0.24	-0.20	-0.33	0.22	-0.11	0.37	0.38
pentose acid*	0.41	0.33	0.55	0.33	-0.22	-0.14	-0.32	0.11	0.02	0.27	0.26
3-bromo-5-chloro-2,6-dihydroxybenzoic acid*	-0.08	-0.24	-0.24	-0.09	0.04	0.12	0.13	-0.01	-0.06	-0.09	-0.09
glycerate	0.32	0.39	0.46	0.16	-0.22	-0.01	-0.37	0.29	-0.13	0.37	0.32
cystine	-0.18	-0.37	-0.39	-0.13	0.32	0.08	0.29	-0.12	0.06	-0.23	-0.22
N-stearoyl-sphinganine (d18:0/18:0)*	-0.15	-0.37	-0.17	-0.19	0.29	0.82	0.25	-0.09	0.01	-0.12	-0.12
X-11315	0.47	0.31	0.43	0.29	-0.23	-0.11	-0.44	0.30	-0.03	0.44	0.42
X-12306	0.23	0.30	0.36	0.34	-0.25	-0.22	-0.31	0.14	-0.03	0.25	0.24
X-12680	0.18	0.16	0.11	0.32	-0.14	-0.10	-0.05	0.05	-0.09	0.05	0.08
X-12726	0.34	0.31	0.38	0.37	-0.40	-0.23	-0.32	0.09	0.02	0.23	0.25
X-14056	-0.28	-0.39	-0.37	-0.08	0.18	0.14	0.30	-0.24	0.08	-0.30	-0.31
X-17351	0.37	0.18	0.43	0.25	-0.22	-0.20	-0.29	0.05	-0.10	0.20	0.19
X-21339	-0.24	-0.30	-0.31	-0.22	0.05	0.01	0.13	-0.04	-0.03	-0.24	-0.21
X-21736	-0.01	-0.11	-0.16	-0.16	0.13	0.33	-0.04	0.04	0.06	-0.12	-0.09
X-21752	0.20	0.22	0.28	0.31	-0.13	-0.29	-0.16	0.04	-0.12	0.15	0.18
X-21842	0.26	0.22	0.33	0.34	-0.24	-0.29	-0.25	0.24	-0.07	0.25	0.28
X-26062	0.13	0.11	0.22	-0.04	0.08	-0.02	-0.04	-0.10	0.62	-0.02	-0.06

	heptenedioate (C7:1-DC)*	1-(1-enyl-oleoyl)-2-docosahexaenoyl-GPC (P-18:1/22:6)*	glycine conjugate of C10H14O2 (1)*	2,6-dihydroxybenzoic acid	3,5-dichloro-2,6-dihydroxybenzoic acid	branched chain 14:0 dicarboxylic acid**	pentose acid*	3-bromo-5-chloro-2,6-dihydroxybenzoic acid*	glycerate	cystine	N-stearoyl-sphinganine (d18:0/18:0)*
tartarate	-0.04	0.21	-0.08	0.31	-0.20	0.42	0.53	-0.14	0.53	-0.34	-0.14
4-acetylphenol sulfate	-0.10	-0.01	-0.21	0.26	-0.15	0.26	0.35	-0.16	0.11	-0.15	-0.18
tartronate (hydroxymalonate)	-0.01	0.36	-0.20	0.27	-0.29	0.47	0.38	-0.18	0.94	-0.70	-0.10
oxalate (ethanedioate)	-0.01	0.36	-0.13	0.24	-0.24	0.42	0.36	-0.17	0.87	-0.60	-0.08
beta-sitosterol	-0.21	0.31	-0.09	0.19	-0.14	0.25	0.19	-0.16	0.20	-0.22	-0.28
indolepropionate	-0.09	0.15	-0.25	0.38	-0.16	0.51	0.41	-0.13	0.37	-0.30	-0.26
pyridoxate	0.15	0.28	-0.08	0.34	-0.08	0.28	0.31	0.00	0.49	-0.27	0.05
adrenate (22:4n6)	0.31	-0.11	0.12	0.01	0.12	-0.20	-0.19	0.22	-0.17	0.23	0.30
10-heptadecenoate (17:1n7)	0.38	-0.02	0.09	0.04	0.06	-0.07	-0.09	0.23	0.01	0.13	0.28
1-arachidoyl-GPC (20:0)	-0.21	0.28	-0.24	0.27	-0.28	0.27	0.31	-0.28	0.39	-0.39	-0.46
catechol sulfate	-0.06	0.08	-0.19	0.38	-0.16	0.44	0.48	-0.11	0.25	-0.21	-0.21
N-methylproline	-0.12	0.10	-0.12	0.09	-0.06	0.42	0.28	-0.09	0.21	-0.12	-0.05
beta-cryptoxanthin	-0.13	0.37	-0.16	0.16	-0.24	0.47	0.27	-0.18	0.35	-0.26	-0.23
androstenediol (3beta,17beta) disulfate (1)	0.01	-0.44	0.15	-0.03	0.15	-0.24	-0.14	0.10	-0.36	0.27	0.14
S-methylmethionine	-0.12	0.15	-0.03	0.14	-0.16	0.35	0.39	-0.14	0.32	-0.23	-0.20
tridecenedioate (C13:1-DC)*	0.80	-0.16	0.12	0.06	0.20	-0.08	-0.10	0.32	-0.02	0.15	0.26
1-behenoyl-GPC (22:0)	-0.16	0.33	-0.24	0.22	-0.28	0.29	0.27	-0.26	0.35	-0.33	-0.43
1-erucoyl-GPC (22:1)*	-0.06	0.28	-0.27	0.17	-0.29	0.31	0.33	-0.20	0.43	-0.36	-0.14
1-lignoceroyl-GPC (24:0)	-0.11	0.27	-0.28	0.25	-0.37	0.27	0.32	-0.32	0.35	-0.31	-0.33
1-nervonoyl-GPC (24:1n9)*	-0.09	0.30	-0.25	0.20	-0.36	0.25	0.33	-0.22	0.33	-0.32	-0.16
(14 or 15)-methylpalmitate (a17:0 or i17:0)	0.51	-0.09	0.07	0.05	0.09	-0.04	-0.05	0.31	0.01	0.10	0.34
guaiacol sulfate	-0.01	0.03	-0.08	0.34	-0.12	0.33	0.44	-0.04	0.12	-0.07	-0.11
N-delta-acetylornithine	0.04	0.15	-0.05	0.30	-0.17	0.34	0.41	-0.08	0.32	-0.18	-0.15
1-eicosenoyl-GPC (20:1)*	-0.14	0.25	-0.31	0.24	-0.28	0.29	0.33	-0.24	0.39	-0.37	-0.37
methyl glucopyranoside (alpha + beta)	-0.13	0.11	-0.20	0.34	-0.29	0.59	0.55	-0.24	0.46	-0.39	-0.17
4-methoxyphenol sulfate	0.00	0.07	-0.12	0.54	-0.14	0.29	0.33	-0.09	0.16	-0.13	-0.19
1-palmitoyl-2-arachidonoyl-GPC (16:0/20:4n6)	0.04	0.00	0.09	-0.11	0.11	-0.24	-0.22	0.04	-0.22	0.32	0.29
sphingomyelin (d18:0/18:0, d19:0/17:0)*	0.22	0.10	0.09	-0.18	0.13	-0.20	-0.14	0.12	-0.01	0.08	0.82
1-stearoyl-2-docosapentaenoyl-GPC (18:0/22:5n6)*	0.03	-0.35	0.04	-0.19	0.21	-0.33	-0.32	0.13	-0.37	0.29	0.25
1-(1-enyl-oleoyl)-2-docosahexaenoyl-GPE (P-18:1/22:6)*	-0.18	0.80	0.05	-0.02	-0.06	0.22	0.11	-0.01	0.29	-0.12	-0.09
ethyl alpha-glucopyranoside	-0.05	-0.21	0.10	-0.04	-0.14	-0.11	0.02	-0.06	-0.13	0.06	0.01
carotene diol (1)	-0.06	0.46	-0.10	0.15	-0.18	0.37	0.27	-0.09	0.37	-0.23	-0.12
carotene diol (2)	-0.09	0.49	-0.14	0.15	-0.17	0.38	0.26	-0.09	0.32	-0.22	-0.12
heptenedioate (C7:1-DC)*	1.00	-0.06	0.11	0.06	0.25	-0.15	-0.08	0.34	-0.01	0.16	0.34
1-(1-enyl-oleoyl)-2-docosahexaenoyl-GPC (P-18:1/22:6)*	-0.06	1.00	-0.10	0.06	-0.08	0.23	0.09	-0.05	0.33	-0.16	-0.09
glycine conjugate of C10H14O2 (1)*	0.11	-0.10	1.00	-0.12	0.32	-0.24	-0.05	0.22	-0.18	0.31	0.11
2,6-dihydroxybenzoic acid	0.06	0.06	-0.12	1.00	-0.08	0.26	0.32	-0.07	0.22	-0.11	-0.16
3,5-dichloro-2,6-dihydroxybenzoic acid	0.25	-0.08	0.32	-0.08	1.00	-0.23	-0.23	0.74	-0.26	0.36	0.18
branched chain 14:0 dicarboxylic acid**	-0.15	0.23	-0.24	0.26	-0.23	1.00	0.47	-0.16	0.43	-0.31	-0.22
pentose acid*	-0.08	0.09	-0.05	0.32	-0.23	0.47	1.00	-0.14	0.39	-0.23	-0.12
3-bromo-5-chloro-2,6-dihydroxybenzoic acid*	0.34	-0.05	0.22	-0.07	0.74	-0.16	-0.14	1.00	-0.17	0.29	0.19
glycerate	-0.01	0.33	-0.18	0.22	-0.26	0.43	0.39	-0.17	1.00	-0.69	-0.06
cystine	0.16	-0.16	0.31	-0.11	0.36	-0.31	-0.23	0.29	-0.69	1.00	0.15
N-stearoyl-sphinganine (d18:0/18:0)*	0.34	-0.09	0.11	-0.16	0.18	-0.22	-0.12	0.19	-0.06	0.15	1.00
X-11315	-0.03	0.30	-0.22	0.29	-0.23	0.47	0.45	-0.06	0.49	-0.35	-0.14
X-12306	-0.16	0.12	-0.25	0.25	-0.18	0.45	0.39	-0.16	0.23	-0.24	-0.26
X-12680	-0.06	0.02	0.05	0.35	0.04	0.09	0.30	-0.01	0.09	0.05	-0.06
X-12726	-0.10	0.04	-0.14	0.24	-0.15	0.42	0.47	-0.07	0.22	-0.21	-0.25
X-14056	0.07	-0.36	0.31	-0.15	0.32	-0.36	-0.25	0.23	-0.72	0.61	0.23
X-17351	-0.04	0.04	-0.12	0.39	-0.09	0.47	0.50	-0.10	0.27	-0.17	-0.18
X-21339	0.05	-0.11	0.29	-0.15	0.51	-0.22	-0.25	0.33	-0.27	0.27	0.03
X-21736	0.40	0.06	0.28	-0.08	0.19	-0.19	-0.02	0.20	-0.09	0.17	0.32
X-21752	0.02	0.12	-0.23	0.43	-0.09	0.41	0.20	-0.09	0.23	-0.11	-0.25
X-21842	-0.14	0.16	-0.08	0.31	-0.10	0.45	0.31	-0.09	0.22	-0.15	-0.30
X-26062	-0.11	-0.14	0.05	-0.03	-0.20	0.07	0.29	-0.10	-0.01	-0.09	-0.02

	X-11315	X-12306	X-12680	X-12726	X-14056	X-17351	X-21339	X-21736	X-21752	X-21842	X-26062
tartarate	0.44	0.19	0.21	0.31	-0.41	0.41	-0.21	-0.05	0.26	0.25	0.08
4-acetylphenol sulfate	0.19	0.34	0.32	0.46	-0.09	0.20	-0.20	-0.06	0.09	0.26	0.07
tartronate (hydroxymalonate)	0.54	0.24	0.09	0.25	-0.75	0.31	-0.32	-0.10	0.25	0.25	0.01
oxalate (ethanedioate)	0.53	0.21	0.15	0.26	-0.65	0.27	-0.26	-0.09	0.24	0.25	-0.07
beta-sitosterol	0.29	0.23	0.13	0.25	-0.25	0.18	-0.03	-0.13	0.11	0.21	-0.02
indolepropionate	0.41	0.50	0.10	0.39	-0.36	0.85	-0.18	-0.16	0.36	0.40	-0.01
pyridoxate	0.36	0.15	0.17	0.23	-0.35	0.29	-0.23	0.05	0.24	0.23	-0.06
adrenate (22:4n6)	-0.17	-0.29	-0.14	-0.30	0.18	-0.02	0.04	0.28	-0.18	-0.24	0.07
10-heptadecenoate (17:1n7)	-0.06	-0.21	-0.16	-0.17	0.05	0.07	-0.03	0.32	-0.07	-0.18	0.08
1-arachidoyl-GPC (20:0)	0.30	0.33	0.17	0.34	-0.42	0.19	-0.30	-0.18	0.20	0.26	0.05
catechol sulfate	0.44	0.53	0.24	0.60	-0.18	0.39	-0.28	-0.10	0.27	0.40	0.02
N-methylproline	0.19	0.16	0.14	0.22	-0.12	0.28	-0.09	-0.12	0.17	0.26	0.02
beta-cryptoxanthin	0.34	0.31	0.10	0.27	-0.36	0.29	-0.21	-0.18	0.28	0.38	-0.06
androstenediol (3beta,17beta) disulfate (1)	-0.21	-0.17	-0.09	-0.11	0.35	-0.15	0.23	0.06	-0.29	-0.22	0.26
S-methylmethionine	0.33	0.32	0.15	0.37	-0.25	0.23	-0.15	-0.13	0.09	0.28	-0.05
tridecenedioate (C13:1-DC)*	-0.04	-0.17	-0.09	-0.10	0.03	0.03	0.09	0.30	0.05	-0.09	-0.02
1-behenoyl-GPC (22:0)	0.33	0.32	0.15	0.35	-0.36	0.16	-0.33	-0.12	0.19	0.27	0.02
1-erucoyl-GPC (22:1)*	0.28	0.29	0.07	0.26	-0.36	0.18	-0.38	0.00	0.19	0.20	0.18
1-lignoceroyl-GPC (24:0)	0.32	0.27	0.12	0.29	-0.37	0.18	-0.50	-0.14	0.24	0.23	0.14
1-nervonoyl-GPC (24:1n9)*	0.27	0.25	0.01	0.23	-0.33	0.14	-0.44	-0.05	0.13	0.21	0.25
(14 or 15)-methylpalmitate (a17:0 or i17:0)	-0.01	-0.17	-0.13	-0.07	0.08	0.06	-0.04	0.30	-0.06	-0.10	0.06
guaiacol sulfate	0.34	0.31	0.26	0.52	-0.02	0.32	-0.26	0.04	0.21	0.30	0.05
N-delta-acetylornithine	0.47	0.23	0.18	0.34	-0.28	0.37	-0.24	-0.01	0.20	0.26	0.13
1-eicosenoyl-GPC (20:1)*	0.31	0.30	0.16	0.31	-0.39	0.18	-0.30	-0.11	0.22	0.22	0.11
methyl glucopyranoside (alpha + beta)	0.43	0.36	0.11	0.38	-0.37	0.43	-0.31	-0.16	0.28	0.33	0.22
4-methoxyphenol sulfate	0.29	0.34	0.32	0.37	-0.08	0.25	-0.22	-0.16	0.31	0.34	-0.04
1-palmitoyl-2-arachidonoyl-GPC (16:0/20:4n6)	-0.23	-0.25	-0.14	-0.40	0.18	-0.22	0.05	0.13	-0.13	-0.24	0.08
sphingomyelin (d18:0/18:0, d19:0/17:0)*	-0.11	-0.22	-0.10	-0.23	0.14	-0.20	0.01	0.33	-0.29	-0.29	-0.02
1-stearoyl-2-docosapentaenoyl-GPC (18:0/22:5n6)*	-0.44	-0.31	-0.05	-0.32	0.30	-0.29	0.13	-0.04	-0.16	-0.25	-0.04
1-(1-enyl-oleoyl)-2-docosahexaenoyl-GPE (P-18:1/22:6)*	0.30	0.14	0.05	0.09	-0.24	0.05	-0.04	0.04	0.04	0.24	-0.10
ethyl alpha-glucopyranoside	-0.03	-0.03	-0.09	0.02	0.08	-0.10	-0.03	0.06	-0.12	-0.07	0.62
carotene diol (1)	0.44	0.25	0.05	0.23	-0.30	0.20	-0.24	-0.12	0.15	0.25	-0.02
carotene diol (2)	0.42	0.24	0.08	0.25	-0.31	0.19	-0.21	-0.09	0.18	0.28	-0.06
heptenedioate (C7:1-DC)*	-0.03	-0.16	-0.06	-0.10	0.07	-0.04	0.05	0.40	0.02	-0.14	-0.11
1-(1-enyl-oleoyl)-2-docosahexaenoyl-GPC (P-18:1/22:6)*	0.30	0.12	0.02	0.04	-0.36	0.04	-0.11	0.06	0.12	0.16	-0.14
glycine conjugate of C10H14O2 (1)*	-0.22	-0.25	0.05	-0.14	0.31	-0.12	0.29	0.28	-0.23	-0.08	0.05
2,6-dihydroxybenzoic acid	0.29	0.25	0.35	0.24	-0.15	0.39	-0.15	-0.08	0.43	0.31	-0.03
3,5-dichloro-2,6-dihydroxybenzoic acid	-0.23	-0.18	0.04	-0.15	0.32	-0.09	0.51	0.19	-0.09	-0.10	-0.20
branched chain 14:0 dicarboxylic acid**	0.47	0.45	0.09	0.42	-0.36	0.47	-0.22	-0.19	0.41	0.45	0.07
pentose acid*	0.45	0.39	0.30	0.47	-0.25	0.50	-0.25	-0.02	0.20	0.31	0.29
3-bromo-5-chloro-2,6-dihydroxybenzoic acid*	-0.06	-0.16	-0.01	-0.07	0.23	-0.10	0.33	0.20	-0.09	-0.09	-0.10
glycerate	0.49	0.23	0.09	0.22	-0.72	0.27	-0.27	-0.09	0.23	0.22	-0.01
cystine	-0.35	-0.24	0.05	-0.21	0.61	-0.17	0.27	0.17	-0.11	-0.15	-0.09
N-stearoyl-sphinganine (d18:0/18:0)*	-0.14	-0.26	-0.06	-0.25	0.23	-0.18	0.03	0.32	-0.25	-0.30	-0.02
X-11315	1.00	0.30	0.11	0.39	-0.39	0.38	-0.28	-0.07	0.24	0.30	0.10
X-12306	0.30	1.00	0.11	0.52	-0.19	0.42	-0.14	-0.11	0.29	0.52	0.04
X-12680	0.11	0.11	1.00	0.20	0.03	0.19	0.00	-0.05	0.17	0.10	-0.16
X-12726	0.39	0.52	0.20	1.00	-0.17	0.38	-0.21	-0.06	0.25	0.39	0.12
X-14056	-0.39	-0.19	0.03	-0.17	1.00	-0.21	0.30	0.14	-0.23	-0.13	-0.01
X-17351	0.38	0.42	0.19	0.38	-0.21	1.00	-0.14	-0.07	0.32	0.35	0.05
X-21339	-0.28	-0.14	0.00	-0.21	0.30	-0.14	1.00	0.07	-0.12	-0.15	-0.13
X-21736	-0.07	-0.11	-0.05	-0.06	0.14	-0.07	0.07	1.00	-0.23	-0.21	0.10
X-21752	0.24	0.29	0.17	0.25	-0.23	0.32	-0.12	-0.23	1.00	0.36	-0.12
X-21842	0.30	0.52	0.10	0.39	-0.13	0.35	-0.15	-0.21	0.36	1.00	0.00
X-26062	0.10	0.04	-0.16	0.12	-0.01	0.05	-0.13	0.10	-0.12	0.00	1.00

Supplementary Table 4: Parameter estimates for metabolome-wide association studies for diet-metabolite associations for each of: The Healthy Eating Index-2015 (HEI-15), The Dietary Approaches to Stop Hypertension (DASH) and a Meditteranean-style (AMED) diet, controlling for BMI

	HEI-15			DASH			AMED		
Metabolite	Beta	SE	P	Beta	SE	P	Beta	SE	P
carnitine	-1.75E-02	6.45E-02	7.86E-01	-5.49E-02	6.23E-02	3.79E-01	4.61E-02	6.06E-02	4.47E-01
3-phenylpropionate (hydrocinnamate)	1.83E-01	6.04E-02	2.73E-03	1.75E-01	5.85E-02	3.00E-03	8.63E-02	5.75E-02	1.35E-01
phenylacetate	4.75E-02	6.61E-02	4.73E-01	1.43E-01	6.34E-02	2.46E-02	2.54E-02	6.22E-02	6.84E-01
hippurate	2.22E-01	6.16E-02	3.81E-04	2.39E-01	5.93E-02	7.04E-05	1.21E-01	5.88E-02	4.03E-02
xanthurenate	6.26E-02	5.81E-02	2.83E-01	2.68E-02	5.64E-02	6.34E-01	-1.16E-03	5.48E-02	9.83E-01
suberate (C8-DC)	1.64E-02	6.39E-02	7.98E-01	8.12E-02	6.16E-02	1.89E-01	4.31E-02	6.00E-02	4.73E-01
3-methyl-2-oxovalerate	-3.68E-02	5.85E-02	5.30E-01	-3.34E-02	5.66E-02	5.56E-01	-1.23E-02	5.50E-02	8.24E-01
methionine sulfoxide	1.59E-01	6.21E-02	1.10E-02	1.64E-01	6.00E-02	6.66E-03	1.54E-01	5.84E-02	9.03E-03
3-methylhistidine	-4.31E-02	6.66E-02	5.18E-01	-2.34E-02	6.45E-02	7.18E-01	-6.68E-02	6.25E-02	2.86E-01
4-acetamidophenylglucuronide	1.15E-01	6.56E-02	7.99E-02	1.58E-01	6.31E-02	1.30E-02	6.83E-02	6.19E-02	2.71E-01
5-hydroxylysine	-1.55E-01	5.91E-02	9.33E-03	-7.19E-02	5.78E-02	2.14E-01	-1.06E-01	5.59E-02	5.98E-02
4-guanidinobutanoate	1.66E-01	6.08E-02	6.60E-03	1.36E-01	5.90E-02	2.19E-02	1.31E-01	5.73E-02	2.32E-02
pimelate (C7-DC)	7.11E-02	6.52E-02	2.76E-01	1.04E-01	6.29E-02	1.01E-01	1.23E-01	6.09E-02	4.50E-02
glucuronate	5.06E-02	6.10E-02	4.08E-01	5.11E-02	5.90E-02	3.88E-01	-2.11E-02	5.74E-02	7.14E-01
glycerol 3-phosphate	-1.26E-03	6.52E-02	9.85E-01	-1.20E-02	6.31E-02	8.49E-01	2.09E-02	6.13E-02	7.33E-01
imidazole lactate	-5.13E-02	6.21E-02	4.09E-01	-6.46E-02	6.00E-02	2.83E-01	4.76E-02	5.84E-02	4.15E-01
kynurenine	1.01E-02	6.06E-02	8.68E-01	8.81E-02	5.85E-02	1.33E-01	-8.46E-02	5.68E-02	1.38E-01
glycerophosphorylcholine (GPC)	1.11E-01	6.41E-02	8.52E-02	1.96E-02	6.24E-02	7.54E-01	8.53E-02	6.04E-02	1.59E-01
N-acetylglutamate	-1.03E-01	5.61E-02	6.83E-02	1.45E-03	5.46E-02	9.79E-01	-1.78E-02	5.31E-02	7.37E-01
tartarate	2.52E-01	5.66E-02	1.18E-05	1.63E-01	5.58E-02	3.78E-03	1.71E-01	5.41E-02	1.79E-03
xanthosine	-7.14E-02	5.92E-02	2.28E-01	1.12E-01	5.70E-02	5.12E-02	-1.08E-01	5.54E-02	5.12E-02
ribitol	-3.22E-03	6.18E-02	9.58E-01	3.84E-02	5.97E-02	5.21E-01	2.74E-02	5.80E-02	6.37E-01
2-isopropylmalate	1.25E-01	6.30E-02	4.79E-02	2.98E-02	6.13E-02	6.27E-01	1.42E-01	5.90E-02	1.65E-02
glycodeoxycholate	4.35E-02	6.48E-02	5.02E-01	-2.81E-02	6.27E-02	6.54E-01	-5.77E-02	6.08E-02	3.44E-01
theophylline	-1.66E-01	6.30E-02	8.90E-03	-1.52E-01	6.11E-02	1.32E-02	-1.01E-01	5.97E-02	9.09E-02
quinate	1.17E-01	5.89E-02	4.75E-02	5.20E-03	5.74E-02	9.28E-01	2.51E-02	5.58E-02	6.53E-01
theobromine	-1.12E-01	6.36E-02	8.09E-02	5.87E-03	6.19E-02	9.25E-01	-2.48E-02	6.02E-02	6.81E-01
gentisate	1.89E-01	6.12E-02	2.20E-03	1.40E-01	5.96E-02	1.92E-02	7.45E-02	5.83E-02	2.03E-01
paraxanthine	-1.35E-01	6.38E-02	3.58E-02	-1.24E-01	6.18E-02	4.59E-02	-7.46E-02	6.03E-02	2.17E-01
indolelactate	6.51E-02	5.64E-02	2.49E-01	3.21E-02	5.47E-02	5.58E-01	5.78E-02	5.30E-02	2.77E-01
3-indoxyl sulfate	-2.80E-02	6.47E-02	6.65E-01	4.37E-02	6.26E-02	4.85E-01	-7.14E-02	6.07E-02	2.40E-01
gamma-glutamylphenylalanine	-4.12E-02	5.67E-02	4.68E-01	6.75E-02	5.48E-02	2.19E-01	-3.69E-02	5.33E-02	4.89E-01
4-methyl-2-oxopentanoate	-2.11E-02	5.95E-02	7.23E-01	-6.00E-02	5.75E-02	2.98E-01	-3.02E-02	5.60E-02	5.90E-01
1,5-anhydroglucitol (1,5-AG)	-1.36E-01	5.96E-02	2.32E-02	-4.54E-02	5.82E-02	4.36E-01	-8.47E-02	5.63E-02	1.34E-01
2-arachidonoylglycerol (20:4)	7.64E-02	6.46E-02	2.38E-01	5.69E-03	6.27E-02	9.28E-01	5.85E-02	6.08E-02	3.37E-01
1-palmityl-GPC (O-16:0)	7.16E-03	6.63E-02	9.14E-01	-4.20E-02	6.41E-02	5.14E-01	-4.14E-02	6.23E-02	5.06E-01
1-stearoyl-2-arachidonoyl-GPI (18:0/20:4)	-1.11E-02	6.49E-02	8.64E-01	-1.09E-01	6.24E-02	8.13E-02	4.10E-02	6.09E-02	5.01E-01
sphingosine 1-phosphate	-6.13E-02	6.57E-02	3.52E-01	-4.75E-02	6.36E-02	4.56E-01	-3.83E-02	6.18E-02	5.36E-01
1-stearoyl-2-oleoyl-GPS (18:0/18:1)	-1.14E-02	6.76E-02	8.67E-01	4.61E-02	6.53E-02	4.81E-01	4.78E-02	6.35E-02	4.52E-01
1-stearoyl-GPI (18:0)	4.72E-02	6.25E-02	4.51E-01	-3.29E-02	6.06E-02	5.88E-01	6.34E-02	5.87E-02	2.82E-01
1,2-dipalmitoyl-GPC (16:0/16:0)	-9.67E-02	6.52E-02	1.39E-01	-1.73E-01	6.25E-02	5.96E-03	-4.92E-02	6.15E-02	4.24E-01
docosahexaenoate (DHA; 22:6n3)	-3.61E-03	6.30E-02	9.54E-01	-1.03E-01	6.06E-02	9.08E-02	5.82E-02	5.91E-02	3.25E-01
1-myristoyl-2-palmitoyl-GPC (14:0/16:0)	-5.65E-02	6.44E-02	3.81E-01	-1.20E-01	6.20E-02	5.29E-02	-7.91E-02	6.04E-02	1.92E-01
alpha-hydroxyisocaproate	-2.10E-02	5.65E-02	7.10E-01	-7.15E-02	5.46E-02	1.91E-01	-8.56E-03	5.32E-02	8.72E-01
maleate	7.56E-02	5.99E-02	2.08E-01	2.85E-02	5.81E-02	6.24E-01	1.00E-01	5.61E-02	7.45E-02
isovalerate (i5:0)	-8.15E-02	6.11E-02	1.83E-01	-3.53E-02	5.93E-02	5.53E-01	-7.10E-02	5.75E-02	2.18E-01
4-acetylphenol sulfate	1.93E-01	6.27E-02	2.31E-03	2.61E-01	5.96E-02	1.74E-05	1.14E-01	5.95E-02	5.59E-02
2-hydroxyoctanoate	3.53E-02	6.68E-02	5.97E-01	7.04E-02	6.45E-02	2.77E-01	-2.73E-02	6.28E-02	6.64E-01
3-hydroxyoctanoate	-7.53E-02	6.18E-02	2.24E-01	-1.23E-02	6.00E-02	8.38E-01	-5.72E-02	5.82E-02	3.26E-01
phenyllactate (PLA)	2.12E-02	5.32E-02	6.90E-01	1.72E-02	5.15E-02	7.38E-01	5.14E-02	4.99E-02	3.05E-01
palmitoylcarnitine (C16)	-2.14E-01	6.16E-02	6.02E-04	-2.45E-01	5.91E-02	4.36E-05	-1.79E-01	5.82E-02	2.31E-03
hexanoylcarnitine (C6)	-1.56E-01	6.42E-02	1.54E-02	-1.68E-01	6.20E-02	7.14E-03	-4.33E-02	6.09E-02	4.77E-01
theanine	1.36E-01	7.23E-02	6.20E-02	1.19E-01	7.01E-02	9.00E-02	7.37E-02	6.83E-02	2.82E-01
N-acetylaspartate (NAA)	-2.22E-02	6.49E-02	7.32E-01	1.06E-01	6.25E-02	9.13E-02	-7.29E-02	6.08E-02	2.32E-01
dehydroepiandrosterone sulfate (DHEA-S)	-1.32E-01	5.76E-02	2.30E-02	-9.16E-02	5.60E-02	1.03E-01	-1.40E-01	5.40E-02	9.84E-03
acetylcarnitine (C2)	-1.31E-03	6.45E-02	9.84E-01	-1.33E-03	6.25E-02	9.83E-01	1.52E-02	6.07E-02	8.02E-01
cysteine s-sulfate	-1.79E-01	6.34E-02	4.95E-03	-1.50E-01	6.15E-02	1.51E-02	-1.80E-01	5.94E-02	2.68E-03
1-palmitoylglycerol (16:0)	-8.24E-03	6.61E-02	9.01E-01	-4.61E-02	6.40E-02	4.72E-01	-2.88E-02	6.22E-02	6.43E-01
tartronate (hydroxymalonate)	2.85E-01	5.55E-02	5.38E-07	2.60E-01	5.40E-02	2.33E-06	2.04E-01	5.32E-02	1.55E-04
oxalate (ethanedioate)	2.95E-01	5.93E-02	1.15E-06	2.88E-01	5.73E-02	9.45E-07	2.08E-01	5.68E-02	3.00E-04
erythritol	4.98E-02	6.38E-02	4.35E-01	7.71E-02	6.16E-02	2.12E-01	2.87E-02	6.00E-02	6.33E-01
saccharin	-5.90E-02	6.43E-02	3.59E-01	-2.68E-02	6.23E-02	6.67E-01	-1.15E-01	6.01E-02	5.60E-02
3-hydroxymyristate	-1.42E-01	6.37E-02	2.66E-02	-1.46E-01	6.16E-02	1.86E-02	-1.05E-01	6.01E-02	8.28E-02
iminodiacetate (IDA)	-8.12E-02	6.72E-02	2.28E-01	-2.21E-02	6.52E-02	7.35E-01	-1.12E-01	6.30E-02	7.68E-02
1-oleoylglycerol (18:1)	7.77E-02	6.48E-02	2.31E-01	1.92E-02	6.28E-02	7.60E-01	-2.71E-03	6.10E-02	9.65E-01
3-methyl-2-oxobutyrate	-6.70E-02	6.22E-02	2.83E-01	-6.53E-02	6.02E-02	2.79E-01	-4.26E-02	5.86E-02	4.67E-01
1,6-anhydroglucose	-8.90E-02	5.98E-02	1.38E-01	-5.05E-02	5.81E-02	3.85E-01	-6.47E-02	5.64E-02	2.52E-01
2-oleoylglycerol (18:1)	8.54E-02	6.24E-02	1.73E-01	2.38E-02	6.06E-02	6.95E-01	-1.99E-03	5.89E-02	9.73E-01
homoarginine	1.10E-01	5.87E-02	6.22E-02	2.68E-02	5.71E-02	6.39E-01	3.44E-02	5.55E-02	5.36E-01
homocitrulline	6.50E-02	6.61E-02	3.26E-01	1.50E-01	6.35E-02	1.86E-02	-7.83E-03	6.23E-02	9.00E-01
pyroglutamylglutamine	3.54E-02	6.10E-02	5.63E-01	3.37E-02	5.91E-02	5.69E-01	-7.85E-02	5.72E-02	1.71E-01
bradykinin	5.27E-02	6.71E-02	4.33E-01	3.89E-02	6.50E-02	5.50E-01	4.10E-02	6.32E-02	5.17E-01
glycylproline	-7.26E-03	6.76E-02	9.15E-01	-2.66E-03	6.54E-02	9.68E-01	2.95E-02	6.35E-02	6.43E-01
2-linoleoylglycerol (18:2)	7.50E-02	6.75E-02	2.68E-01	4.37E-02	6.54E-02	5.04E-01	1.60E-02	6.36E-02	8.01E-01
3-hydroxydecanoate	-8.68E-02	6.25E-02	1.66E-01	-5.08E-02	6.07E-02	4.03E-01	-7.41E-02	5.88E-02	2.09E-01
citramalate	1.69E-01	6.23E-02	6.93E-03	1.28E-01	6.06E-02	3.55E-02	1.08E-01	5.90E-02	6.87E-02
EDTA	-7.69E-02	6.11E-02	2.09E-01	-1.20E-01	5.88E-02	4.21E-02	-1.03E-01	5.72E-02	7.43E-02

N-acetylglycine	-5.68E-02	6.13E-02	3.55E-01	-8.59E-02	5.92E-02	1.48E-01	-4.80E-03	5.77E-02	9.34E-01
ribonate	8.70E-02	6.14E-02	1.57E-01	6.85E-02	5.95E-02	2.51E-01	-4.25E-02	5.78E-02	4.63E-01
threonate	2.06E-01	5.85E-02	4.98E-04	2.30E-01	5.62E-02	5.42E-05	1.27E-01	5.57E-02	2.36E-02
galactonate	6.30E-02	6.30E-02	3.18E-01	6.05E-02	6.10E-02	3.22E-01	-4.09E-03	5.94E-02	9.45E-01
beta-sitosterol	1.88E-01	6.13E-02	2.44E-03	9.78E-02	6.01E-02	1.04E-01	2.28E-01	5.70E-02	8.06E-05
indoleacetate	-1.58E-02	5.94E-02	7.90E-01	-4.08E-02	5.74E-02	4.78E-01	-8.34E-03	5.58E-02	8.81E-01
1-linoleoylglycerol (18:2)	7.69E-02	6.48E-02	2.37E-01	5.38E-02	6.28E-02	3.92E-01	1.64E-02	6.11E-02	7.89E-01
1-methylhistidine	-2.26E-02	6.31E-02	7.20E-01	-4.58E-02	6.11E-02	4.54E-01	-2.92E-02	5.93E-02	6.24E-01
butyrylcarnitine (C4)	-8.60E-02	6.24E-02	1.69E-01	-9.26E-02	6.03E-02	1.26E-01	-7.46E-02	5.87E-02	2.05E-01
isobutyrylcarnitine (C4)	5.65E-02	6.46E-02	3.83E-01	1.39E-01	6.21E-02	2.64E-02	1.06E-01	6.05E-02	8.20E-02
glycolithocholate	1.23E-02	6.53E-02	8.51E-01	-2.39E-02	6.31E-02	7.05E-01	-1.02E-01	6.11E-02	9.65E-02
androsterone sulfate	-1.73E-01	5.99E-02	4.11E-03	-1.32E-01	5.84E-02	2.43E-02	-1.06E-01	5.68E-02	6.43E-02
indolepropionate	2.90E-01	5.58E-02	3.85E-07	2.93E-01	5.37E-02	1.05E-07	1.97E-01	5.36E-02	2.86E-04
N-(2-furoyl)glycine	8.66E-02	6.46E-02	1.81E-01	3.97E-02	6.27E-02	5.27E-01	2.63E-02	6.09E-02	6.66E-01
trigonelline (N'-methylnicotinate)	1.24E-01	6.08E-02	4.25E-02	9.51E-02	5.90E-02	1.09E-01	5.86E-02	5.75E-02	3.09E-01
dodecanedioate (C12-DC)	-1.03E-01	6.11E-02	9.18E-02	-5.13E-04	5.95E-02	9.93E-01	-9.21E-02	5.75E-02	1.11E-01
N-acetyltyrosine	-7.74E-02	5.89E-02	1.90E-01	-8.07E-02	5.70E-02	1.58E-01	-4.16E-02	5.55E-02	4.54E-01
1,3-dimethylurate	-1.78E-01	6.37E-02	5.57E-03	-1.59E-01	6.18E-02	1.04E-02	-1.41E-01	6.02E-02	1.98E-02
3-methylxanthine	-4.87E-02	6.20E-02	4.33E-01	6.95E-02	5.99E-02	2.47E-01	-3.45E-02	5.83E-02	5.54E-01
3-hydroxylaurate	-1.36E-01	6.19E-02	2.93E-02	-8.67E-02	6.02E-02	1.51E-01	-1.03E-01	5.84E-02	7.96E-02
pyridoxate	2.31E-01	5.92E-02	1.20E-04	2.45E-01	5.69E-02	2.37E-05	1.15E-01	5.67E-02	4.41E-02
gamma-glutamylvaline	-1.12E-01	6.03E-02	6.30E-02	-5.25E-02	5.86E-02	3.71E-01	-1.12E-01	5.66E-02	4.82E-02
pyroglutamylglycine	8.78E-02	6.46E-02	1.75E-01	5.59E-02	6.26E-02	3.73E-01	1.09E-01	6.06E-02	7.22E-02
pyroglutamylvaline	-3.98E-02	6.47E-02	5.39E-01	6.93E-02	6.25E-02	2.69E-01	-6.40E-02	6.08E-02	2.93E-01
3-hydroxysebacate	-5.10E-03	6.36E-02	9.36E-01	6.26E-02	6.14E-02	3.09E-01	-7.95E-03	5.98E-02	8.94E-01
5-hydroxyhexanoate	9.00E-02	6.36E-02	1.58E-01	1.39E-01	6.12E-02	2.42E-02	9.15E-02	5.97E-02	1.27E-01
propionylglycine	-5.83E-03	6.48E-02	9.28E-01	8.86E-02	6.25E-02	1.57E-01	1.56E-02	6.09E-02	7.98E-01
butyrylglycine	7.19E-02	6.53E-02	2.72E-01	5.15E-02	6.33E-02	4.17E-01	5.91E-02	6.15E-02	3.37E-01
2-methylbutyrylglycine	1.07E-01	6.29E-02	8.87E-02	9.78E-02	6.09E-02	1.09E-01	6.74E-02	5.93E-02	2.56E-01
propionylcarnitine (C3)	7.42E-03	6.31E-02	9.07E-01	7.11E-02	6.10E-02	2.45E-01	3.68E-02	5.93E-02	5.36E-01
pro-hydroxy-pro	-1.84E-01	6.35E-02	4.04E-03	-4.85E-02	6.23E-02	4.37E-01	-1.01E-01	6.03E-02	9.64E-02
3-hydroxy-2-ethylpropionate	-1.06E-01	6.01E-02	7.85E-02	2.30E-02	5.85E-02	6.94E-01	-1.16E-01	5.64E-02	4.16E-02
3-carboxy-4-methyl-5-propyl-2-furanpropanoate (CMPF)	1.13E-01	6.39E-02	7.76E-02	-1.03E-03	6.22E-02	9.87E-01	1.65E-01	5.96E-02	6.03E-03
docosapentaenoate (n3 DPA; 22:5n3)	-1.15E-01	6.36E-02	7.20E-02	-1.43E-01	6.14E-02	2.01E-02	-5.61E-02	6.01E-02	3.51E-01
docosadienoate (22:2n6)	-1.43E-01	6.40E-02	2.63E-02	-1.96E-01	6.14E-02	1.60E-03	-1.24E-01	6.03E-02	4.11E-02
adrenate (22:4n6)	-2.64E-01	6.32E-02	4.01E-05	-2.51E-01	6.13E-02	5.44E-05	-1.68E-01	6.05E-02	5.77E-03
10-undecenoate (11:1n1)	-1.46E-01	6.24E-02	2.00E-02	-1.68E-01	6.01E-02	5.51E-03	-1.83E-01	5.82E-02	1.79E-03
4-imidazoleacetate	1.26E-01	6.31E-02	4.73E-02	1.36E-01	6.09E-02	2.60E-02	1.06E-01	5.94E-02	7.64E-02
1-methyl-4-imidazoleacetate	-7.49E-02	5.97E-02	2.10E-01	-1.22E-02	5.79E-02	8.33E-01	-6.25E-02	5.61E-02	2.67E-01
sebacate (C10-DC)	-9.09E-02	6.11E-02	1.38E-01	-3.25E-02	5.93E-02	5.84E-01	-1.14E-01	5.73E-02	4.81E-02
guanidinosuccinate	1.78E-01	6.22E-02	4.53E-03	1.94E-01	6.00E-02	1.35E-03	1.13E-01	5.89E-02	5.55E-02
delta-tocopherol	-6.44E-02	6.30E-02	3.07E-01	-2.96E-02	6.10E-02	6.28E-01	2.35E-02	5.93E-02	6.93E-01
I-urobilinogen	-1.45E-01	6.46E-02	2.54E-02	-8.24E-02	6.29E-02	1.92E-01	8.20E-03	6.13E-02	8.94E-01
stearidonate (18:4n3)	-9.23E-02	6.06E-02	1.29E-01	-1.20E-01	5.85E-02	4.07E-02	-1.94E-02	5.72E-02	7.35E-01
5-dodecenoate (12:1n7)	-1.69E-01	5.97E-02	5.09E-03	-1.73E-01	5.77E-02	2.91E-03	-8.55E-02	5.67E-02	1.33E-01
octanoylcarnitine (C8)	-1.02E-01	6.44E-02	1.15E-01	-1.47E-01	6.20E-02	1.86E-02	-1.81E-02	6.08E-02	7.66E-01
tauro-beta-muricholate	1.03E-01	6.43E-02	1.10E-01	1.03E-01	6.22E-02	9.83E-02	2.74E-03	6.07E-02	9.64E-01
decanoylcarnitine (C10)	-1.09E-01	6.49E-02	9.33E-02	-1.45E-01	6.25E-02	2.11E-02	-1.98E-02	6.13E-02	7.46E-01
N-acetylglutamine	-1.50E-01	5.93E-02	1.21E-02	-1.24E-01	5.76E-02	3.25E-02	-9.40E-02	5.61E-02	9.51E-02
N-acetyltryptophan	8.59E-03	5.81E-02	8.83E-01	-2.57E-02	5.62E-02	6.48E-01	7.26E-03	5.46E-02	8.94E-01
N-acetylphenylalanine	-1.31E-01	5.76E-02	2.40E-02	-1.13E-01	5.58E-02	4.40E-02	-6.83E-02	5.45E-02	2.11E-01
1-palmitoyl-GPC (16:0)	3.52E-02	6.25E-02	5.74E-01	-7.82E-02	6.04E-02	1.97E-01	5.67E-03	5.88E-02	9.23E-01
1-margaroyl-GPC (17:0)	1.13E-01	6.36E-02	7.74E-02	1.61E-01	6.11E-02	8.92E-03	2.42E-02	6.01E-02	6.87E-01
N-acetylarginine	-5.57E-02	6.46E-02	3.89E-01	-2.85E-02	6.26E-02	6.50E-01	4.61E-02	6.08E-02	4.49E-01
piperine	7.43E-02	6.55E-02	2.57E-01	-1.23E-01	6.31E-02	5.28E-02	1.20E-01	6.13E-02	5.04E-02
campesterol	3.49E-03	6.24E-02	9.55E-01	-9.33E-02	6.01E-02	1.21E-01	1.07E-01	5.83E-02	6.68E-02
myristoylcarnitine (C14)	-2.37E-01	6.13E-02	1.41E-04	-1.85E-01	5.99E-02	2.18E-03	-1.79E-01	5.82E-02	2.34E-03
1-stearoyl-GPC (18:0)	1.04E-01	6.38E-02	1.04E-01	5.35E-02	6.19E-02	3.88E-01	9.12E-02	6.00E-02	1.29E-01
1-oleoyl-GPC (18:1)	1.42E-01	5.72E-02	1.35E-02	6.78E-02	5.58E-02	2.26E-01	1.27E-01	5.38E-02	1.94E-02
N-acetylthreonine	4.42E-04	6.15E-02	9.94E-01	1.14E-01	5.91E-02	5.43E-02	-4.80E-03	5.78E-02	9.34E-01
N-acetylisoleucine	-3.84E-02	6.37E-02	5.47E-01	-4.74E-02	6.16E-02	4.42E-01	2.45E-02	5.99E-02	6.83E-01
10-nonadecenoate (19:1n9)	-2.23E-01	6.08E-02	3.00E-04	-2.32E-01	5.86E-02	9.67E-05	-1.97E-01	5.73E-02	6.73E-04
10-heptadecenoate (17:1n7)	-2.45E-01	5.80E-02	3.28E-05	-2.44E-01	5.60E-02	1.89E-05	-1.95E-01	5.50E-02	4.49E-04
hyocholate	-1.46E-01	6.33E-02	2.22E-02	-8.04E-02	6.17E-02	1.94E-01	2.15E-02	6.01E-02	7.20E-01
HWESASLLR	2.76E-02	6.45E-02	6.70E-01	5.19E-02	6.24E-02	4.06E-01	-6.04E-03	6.07E-02	9.21E-01
epiandrosterone sulfate	-1.30E-01	5.79E-02	2.51E-02	-1.02E-01	5.62E-02	7.16E-02	-8.21E-02	5.47E-02	1.34E-01
bradykinin, des-arg(9)	5.27E-02	6.76E-02	4.37E-01	7.96E-02	6.54E-02	2.24E-01	3.31E-02	6.36E-02	6.03E-01
N-acetylhistidine	-9.05E-02	6.31E-02	1.52E-01	-2.55E-02	6.13E-02	6.77E-01	-5.16E-02	5.94E-02	3.86E-01
gamma-glutamylglycine	-6.95E-02	6.53E-02	2.88E-01	1.15E-02	6.33E-02	8.56E-01	-5.65E-02	6.14E-02	3.58E-01
gamma-glutamyltryptophan	2.56E-02	6.09E-02	6.74E-01	4.90E-02	5.89E-02	4.06E-01	5.49E-02	5.72E-02	3.38E-01
stachydrine	1.99E-01	6.54E-02	2.54E-03	2.17E-01	6.30E-02	6.46E-04	1.78E-01	6.15E-02	4.07E-03
alpha-hydroxyisovalerate	-4.13E-02	5.95E-02	4.88E-01	-1.59E-01	5.69E-02	5.52E-03	7.72E-02	5.58E-02	1.67E-01
hydroxybupropion	5.28E-02	6.77E-02	4.36E-01	1.19E-01	6.52E-02	6.90E-02	-5.40E-02	6.37E-02	3.97E-01
gamma-glutamylmethionine	-6.94E-02	5.94E-02	2.44E-01	-2.22E-02	5.76E-02	7.00E-01	-7.02E-02	5.58E-02	2.10E-01
gamma-glutamylthreonine	-1.62E-01	6.48E-02	1.28E-02	-9.87E-02	6.31E-02	1.19E-01	-6.95E-02	6.14E-02	2.59E-01
p-cresol sulfate	2.68E-02	6.49E-02	6.80E-01	1.11E-01	6.25E-02	7.66E-02	-6.43E-02	6.09E-02	2.92E-01
erythronate*	8.96E-02	6.27E-02	1.54E-01	1.65E-01	6.01E-02	6.59E-03	9.61E-02	5.89E-02	1.04E-01
Fibrinopeptide A*	7.53E-02	6.67E-02	2.60E-01	5.10E-02	6.46E-02	4.31E-01	2.83E-02	6.28E-02	6.52E-01
Fibrinopeptide A, des-ala(1)*	9.07E-02	6.61E-02	1.72E-01	6.41E-02	6.41E-02	3.19E-01	4.98E-02	6.23E-02	4.25E-01
Fibrinopeptide A, phosphono-ser(3)*	3.87E-02	6.72E-02	5.66E-01	3.93E-02	6.50E-02	5.47E-01	2.32E-02	6.32E-02	7.14E-01
XHWESASXXR*	8.74E-02	6.69E-02	1.92E-01	1.03E-01	6.46E-02	1.14E-01	4.26E-02	6.30E-02	4.99E-01
HWESASXX*	1.12E-01	6.60E-02	9.05E-02	1.05E-01	6.39E-02	1.02E-01	7.35E-02	6.22E-02	2.38E-01

HXGXA*	1.20E-01	6.82E-02	8.03E-02	9.77E-02	6.61E-02	1.41E-01	5.78E-02	6.44E-02	3.70E-01
salicyluric glucuronide*	1.19E-01	6.44E-02	6.66E-02	1.43E-01	6.21E-02	2.16E-02	1.21E-02	6.09E-02	8.43E-01
eicosenoate (20:1)	-9.60E-02	6.20E-02	1.23E-01	-1.25E-01	5.98E-02	3.82E-02	-6.83E-02	5.84E-02	2.43E-01
linolenate [alpha or gamma; (18:3n3 or 6)]	-9.94E-02	5.97E-02	9.69E-02	-9.00E-02	5.78E-02	1.21E-01	-4.89E-02	5.63E-02	3.86E-01
aconitate [cis or trans]	-1.13E-01	5.76E-02	5.08E-02	-2.95E-03	5.61E-02	9.58E-01	-7.86E-02	5.43E-02	1.49E-01
1-myristoyl-GPC (14:0)	1.80E-02	6.24E-02	7.74E-01	-3.61E-02	6.04E-02	5.50E-01	-4.41E-02	5.86E-02	4.52E-01
1-arachidoyl-GPC (20:0)	3.15E-01	5.72E-02	8.35E-08	2.33E-01	5.66E-02	5.03E-05	2.79E-01	5.41E-02	4.64E-07
metoprolol acid metabolite*	5.33E-02	6.64E-02	4.23E-01	1.14E-01	6.40E-02	7.69E-02	8.40E-02	6.23E-02	1.79E-01
heme	3.12E-02	6.42E-02	6.27E-01	1.29E-02	6.21E-02	8.36E-01	2.82E-02	6.03E-02	6.40E-01
stearoylcarnitine (C18)	-1.35E-01	6.16E-02	2.94E-02	-8.71E-02	5.99E-02	1.48E-01	-6.94E-02	5.83E-02	2.35E-01
laurylcarnitine (C12)	-1.75E-01	6.34E-02	6.26E-03	-1.90E-01	6.11E-02	2.07E-03	-9.83E-02	6.01E-02	1.03E-01
isovalerylcarnitine (C5)	-1.07E-01	6.47E-02	9.89E-02	-4.69E-02	6.28E-02	4.56E-01	-5.03E-02	6.10E-02	4.11E-01
1-linoleoyl-GPC (18:2)	6.72E-02	5.89E-02	2.55E-01	7.57E-02	5.69E-02	1.85E-01	9.41E-02	5.52E-02	8.93E-02
7-methylxanthine	-3.86E-02	6.38E-02	5.46E-01	9.91E-02	6.15E-02	1.08E-01	-2.63E-02	6.00E-02	6.62E-01
1,3,7-trimethylurate	-1.36E-01	6.33E-02	3.27E-02	-1.20E-01	6.13E-02	5.22E-02	-1.01E-01	5.97E-02	9.25E-02
3,7-dimethylurate	-4.42E-02	6.35E-02	4.87E-01	5.73E-02	6.14E-02	3.52E-01	-1.68E-02	5.98E-02	7.79E-01
1,7-dimethylurate	-1.51E-01	6.30E-02	1.71E-02	-1.07E-01	6.12E-02	8.15E-02	-1.02E-01	5.95E-02	8.64E-02
1-methylurate	-6.31E-02	6.35E-02	3.21E-01	-4.14E-02	6.15E-02	5.02E-01	-3.96E-02	5.97E-02	5.08E-01
5-acetylamino-6-formylamino-3-methyluracil	-7.83E-02	6.45E-02	2.26E-01	-8.30E-02	6.24E-02	1.85E-01	-1.08E-01	6.05E-02	7.62E-02
5-acetylamino-6-amino-3-methyluracil	-1.05E-01	6.34E-02	1.00E-01	-4.98E-02	6.16E-02	4.20E-01	-9.26E-02	5.97E-02	1.22E-01
indolebutyrate	2.46E-02	6.13E-02	6.88E-01	1.59E-01	5.86E-02	7.12E-03	-4.05E-02	5.76E-02	4.83E-01
1-methylxanthine	-1.24E-01	6.37E-02	5.36E-02	-9.11E-02	6.18E-02	1.42E-01	-6.88E-02	6.02E-02	2.54E-01
N1-methylinosine	-5.69E-02	6.31E-02	3.68E-01	5.81E-02	6.11E-02	3.43E-01	-1.01E-01	5.91E-02	8.86E-02
N2,N2-dimethylguanosine	-7.32E-02	5.93E-02	2.18E-01	1.14E-02	5.75E-02	8.43E-01	-1.21E-01	5.54E-02	2.94E-02
N4-acetylcytidine	-1.08E-01	5.99E-02	7.36E-02	-2.58E-02	5.83E-02	6.59E-01	-8.39E-02	5.65E-02	1.38E-01
N6-carbamoylthreonyladenosine	-5.81E-02	5.78E-02	3.16E-01	2.85E-02	5.60E-02	6.11E-01	-1.10E-01	5.40E-02	4.24E-02
orotidine	-1.04E-01	5.68E-02	6.68E-02	-2.13E-02	5.53E-02	7.01E-01	-9.71E-02	5.34E-02	7.00E-02
phenylacetylglutamine	3.22E-02	6.59E-02	6.26E-01	1.35E-01	6.33E-02	3.36E-02	-1.55E-03	6.20E-02	9.80E-01
4-hydroxyhippurate	-3.33E-02	6.57E-02	6.12E-01	7.47E-02	6.34E-02	2.40E-01	3.13E-02	6.17E-02	6.12E-01
5,6-dihydrouridine	-4.96E-02	6.03E-02	4.12E-01	1.85E-02	5.85E-02	7.52E-01	-5.51E-02	5.67E-02	3.32E-01
3-(3-amino-3-carboxypropyl)uridine*	-4.13E-02	6.07E-02	4.97E-01	-1.98E-02	5.88E-02	7.36E-01	-1.03E-01	5.68E-02	7.07E-02
1-arachidonylglycerol (20:4)	-3.28E-02	5.89E-02	5.78E-01	-9.12E-02	5.67E-02	1.09E-01	-1.47E-02	5.54E-02	7.91E-01
1-linolenoylglycerol (18:3)	1.59E-01	6.42E-02	1.36E-02	8.53E-02	6.26E-02	1.74E-01	1.06E-01	6.07E-02	8.18E-02
cysteine-glutathione disulfide	-1.71E-02	6.54E-02	7.94E-01	4.71E-04	6.33E-02	9.94E-01	-4.37E-02	6.14E-02	4.78E-01
5-methyluridine (ribothymidine)	-1.05E-01	6.46E-02	1.05E-01	-1.93E-02	6.28E-02	7.59E-01	-9.87E-02	6.07E-02	1.05E-01
isovalerylglycine	7.66E-02	6.27E-02	2.23E-01	1.61E-01	6.01E-02	7.84E-03	7.45E-02	5.90E-02	2.07E-01
3-hydroxydodecanedioate*	-4.53E-02	6.15E-02	4.62E-01	-2.20E-02	5.96E-02	7.12E-01	-1.46E-02	5.79E-02	8.01E-01
7-methylguanine	-8.57E-02	6.35E-02	1.78E-01	-4.48E-02	6.16E-02	4.67E-01	-8.60E-02	5.97E-02	1.51E-01
1-stearoyl-GPE (18:0)	5.72E-02	6.24E-02	3.60E-01	-2.61E-03	6.05E-02	9.66E-01	7.15E-02	5.86E-02	2.24E-01
1-stearoyl-GPG (18:0)	-4.95E-02	6.42E-02	4.41E-01	-4.87E-02	6.21E-02	4.33E-01	-1.09E-01	6.01E-02	7.00E-02
N1-Methyl-2-pyridone-5-carboxamide	1.33E-02	6.38E-02	8.35E-01	1.12E-01	6.13E-02	6.86E-02	-3.23E-02	5.99E-02	5.90E-01
mead acid (20:3n9)	-8.53E-02	6.59E-02	1.97E-01	-1.29E-01	6.35E-02	4.34E-02	-1.06E-01	6.18E-02	8.64E-02
gamma-glutamylisoleucine*	-5.05E-02	5.78E-02	3.83E-01	3.72E-02	5.60E-02	5.07E-01	-3.57E-02	5.44E-02	5.12E-01
oleoylcarnitine (C18:1)	-3.57E-02	6.15E-02	5.62E-01	-1.06E-01	5.93E-02	7.45E-02	-1.70E-02	5.79E-02	7.69E-01
2-methylbutyrylcarnitine (C5)	3.40E-02	6.52E-02	6.02E-01	7.76E-02	6.30E-02	2.19E-01	2.47E-02	6.14E-02	6.88E-01
phenol sulfate	-1.54E-02	6.37E-02	8.09E-01	2.59E-02	6.17E-02	6.75E-01	3.75E-02	5.99E-02	5.32E-01
1-palmitoleoyl-GPC (16:1)*	2.68E-03	6.18E-02	9.65E-01	-1.03E-01	5.95E-02	8.52E-02	-3.34E-02	5.81E-02	5.66E-01
hexanoylglycine	-4.41E-02	6.51E-02	4.98E-01	-1.05E-01	6.27E-02	9.39E-02	-5.02E-02	6.11E-02	4.12E-01
glutamine_degradant*	3.58E-02	5.35E-02	5.04E-01	9.37E-02	5.15E-02	7.01E-02	9.98E-03	5.04E-02	8.43E-01
2-hydroxy-3-methylvalerate	1.23E-02	5.56E-02	8.25E-01	-1.03E-01	5.35E-02	5.46E-02	1.16E-01	5.18E-02	2.58E-02
homostachydrine*	5.48E-02	6.39E-02	3.92E-01	5.18E-02	6.19E-02	4.03E-01	-3.44E-02	6.01E-02	5.67E-01
1-arachidonoyl-GPC (20:4n6)*	-3.96E-02	6.51E-02	5.43E-01	-7.90E-02	6.29E-02	2.10E-01	-1.12E-02	6.12E-02	8.55E-01
1-dihomo-linolenoyl-GPC (20:3n3 or 6)*	1.18E-01	6.14E-02	5.61E-02	6.33E-02	5.97E-02	2.90E-01	1.30E-02	5.81E-02	8.23E-01
1-dihomo-linoleoyl-GPC (20:2)*	1.86E-01	6.00E-02	2.16E-03	1.04E-01	5.87E-02	7.61E-02	1.39E-01	5.67E-02	1.52E-02
2-arachidonoyl-GPC (20:4)*	-4.02E-02	6.63E-02	5.45E-01	-1.00E-01	6.39E-02	1.17E-01	-2.77E-02	6.23E-02	6.57E-01
2-oleoyl-GPC (18:1)*	6.94E-02	6.06E-02	2.53E-01	-1.12E-03	5.88E-02	9.85E-01	6.48E-02	5.70E-02	2.56E-01
2-linoleoyl-GPC (18:2)*	6.87E-03	6.18E-02	9.11E-01	4.62E-02	5.97E-02	4.40E-01	3.29E-02	5.80E-02	5.71E-01
2-palmitoleoyl-GPC (16:1)*	-3.70E-02	6.25E-02	5.54E-01	-1.13E-01	6.02E-02	6.10E-02	-5.04E-02	5.88E-02	3.92E-01
2-palmitoyl-GPC (16:0)*	-6.08E-04	6.33E-02	9.92E-01	-9.51E-02	6.10E-02	1.20E-01	-4.23E-02	5.95E-02	4.78E-01
2-myristoyl-GPC (14:0)*	4.71E-02	6.28E-02	4.54E-01	-2.19E-02	6.09E-02	7.19E-01	-2.76E-02	5.91E-02	6.41E-01
1-docosahexaenoyl-GPC (22:6)*	1.27E-01	6.19E-02	4.16E-02	9.99E-03	6.03E-02	8.69E-01	1.78E-01	5.76E-02	2.19E-03
1-palmitoyl-GPE (16:0)	2.76E-02	6.34E-02	6.64E-01	-7.41E-02	6.12E-02	2.27E-01	2.70E-02	5.96E-02	6.51E-01
1-oleoyl-GPE (18:1)	1.12E-01	6.10E-02	6.64E-02	1.13E-01	5.91E-02	5.77E-02	1.17E-01	5.73E-02	4.20E-02
1-linoleoyl-GPE (18:2)*	5.28E-03	6.20E-02	9.32E-01	4.02E-02	6.00E-02	5.03E-01	1.79E-02	5.83E-02	7.60E-01
1-arachidonoyl-GPE (20:4n6)*	-1.11E-01	6.39E-02	8.25E-02	-1.43E-01	6.16E-02	2.14E-02	-6.30E-02	6.03E-02	2.97E-01
N-acetylcitrulline	-7.67E-02	5.60E-02	1.72E-01	4.67E-03	5.43E-02	9.32E-01	-2.86E-02	5.28E-02	5.88E-01
2-hydroxypalmitate	-1.11E-01	6.29E-02	7.74E-02	-1.17E-01	6.08E-02	5.54E-02	-1.09E-01	5.91E-02	6.72E-02
docosapentaenoate (n6 DPA; 22:5n6)	-1.97E-01	6.51E-02	2.74E-03	-2.41E-01	6.24E-02	1.36E-04	-1.97E-01	6.11E-02	1.39E-03
gulonate*	7.35E-02	6.27E-02	2.42E-01	9.51E-02	6.06E-02	1.18E-01	-6.96E-03	5.91E-02	9.06E-01
isobutyrylglycine	7.61E-02	6.39E-02	2.35E-01	1.21E-01	6.16E-02	5.09E-02	4.95E-02	6.01E-02	4.11E-01
glutarylcarnitine (C5-DC)	-7.12E-02	6.40E-02	2.67E-01	-9.33E-02	6.18E-02	1.32E-01	2.48E-02	6.02E-02	6.81E-01
2-methylmalonylcarnitine (C4-DC)	-5.92E-03	6.52E-02	9.28E-01	4.16E-02	6.30E-02	5.10E-01	-5.98E-02	6.12E-02	3.29E-01
tiglylcarnitine (C5:1-DC)	3.39E-02	6.21E-02	5.86E-01	1.12E-01	5.98E-02	6.20E-02	-3.19E-02	5.84E-02	5.85E-01
hydroquinone sulfate	9.34E-02	6.72E-02	1.66E-01	1.59E-01	6.46E-02	1.44E-02	1.08E-01	6.31E-02	8.83E-02
catechol sulfate	3.18E-01	5.59E-02	3.04E-08	2.73E-01	5.48E-02	1.10E-06	2.14E-01	5.40E-02	9.31E-05
cholesterol sulfate	3.32E-02	6.12E-02	5.88E-01	-5.56E-02	5.91E-02	3.48E-01	6.60E-02	5.74E-02	2.51E-01
7-alpha-hydroxy-3-oxo-4-cholestenoate (7-Hoca)	-1.10E-01	6.32E-02	8.40E-02	-6.89E-02	6.14E-02	2.63E-01	-5.29E-02	5.97E-02	3.76E-01
tetradecanedioate (C14-DC)	-1.19E-01	5.95E-02	4.56E-02	-1.48E-01	5.73E-02	1.03E-02	-1.20E-01	5.59E-02	3.24E-02
hexadecanedioate (C16-DC)	-1.56E-01	6.02E-02	1.02E-02	-1.51E-01	5.83E-02	1.03E-02	-1.15E-01	5.69E-02	4.46E-02
octadecanedioate (C18-DC)	-9.58E-02	6.34E-02	1.32E-01	-3.52E-02	6.16E-02	5.68E-01	-5.32E-02	5.97E-02	3.74E-01
undecanedioate (C11-DC)	1.92E-02	6.62E-02	7.72E-01	1.23E-02	6.41E-02	8.48E-01	2.10E-02	6.22E-02	7.36E-01
glycerophosphoethanolamine	3.35E-02	6.36E-02	5.98E-01	3.23E-03	6.16E-02	9.58E-01	6.76E-02	5.97E-02	2.59E-01

Supplementary Table 4: Parameter estimates for metabolome-wide association studies for diet-metabolite associations for each of: HEI-15, DASH and AMED diet, controlling for BMI

3-(3-hydroxyphenyl)propionate	1.07E-01	6.53E-02	1.04E-01	1.39E-01	6.29E-02	2.78E-02	1.36E-01	6.11E-02	2.69E-02
ectoine	4.53E-02	6.48E-02	4.85E-01	7.49E-02	6.26E-02	2.32E-01	-1.22E-02	6.09E-02	8.41E-01
2-oleoyl-GPE (18:1)*	8.23E-02	6.21E-02	1.86E-01	1.08E-01	6.00E-02	7.41E-02	7.99E-02	5.84E-02	1.73E-01
2-palmitoyl-GPE (16:0)*	-3.68E-03	6.43E-02	9.54E-01	-1.15E-01	6.19E-02	6.36E-02	2.63E-03	6.05E-02	9.65E-01
1-arachidonoyl-GPI (20:4)*	-1.17E-01	6.52E-02	7.26E-02	-2.31E-01	6.19E-02	2.27E-04	-9.17E-02	6.14E-02	1.37E-01
1-palmitoyl-GPI (16:0)	2.23E-02	6.43E-02	7.29E-01	-3.95E-02	6.22E-02	5.26E-01	-2.40E-02	6.05E-02	6.92E-01
glycolithocholate sulfate*	6.65E-02	6.29E-02	2.91E-01	5.04E-02	6.09E-02	4.09E-01	-7.31E-02	5.91E-02	2.17E-01
taurolithocholate 3-sulfate	6.40E-02	6.46E-02	3.23E-01	5.06E-02	6.25E-02	4.19E-01	-1.09E-01	6.05E-02	7.31E-02
deoxycarnitine	-9.70E-02	6.19E-02	1.18E-01	2.56E-02	6.01E-02	6.70E-01	-5.15E-02	5.83E-02	3.78E-01
N6-succinyladenosine	-2.77E-02	6.01E-02	6.45E-01	-2.15E-02	5.82E-02	7.11E-01	-4.89E-02	5.64E-02	3.87E-01
1-ribosyl-imidazoleacetate*	8.23E-02	6.03E-02	1.73E-01	1.16E-01	5.81E-02	4.64E-02	2.72E-02	5.68E-02	6.32E-01
2-arachidonoyl-GPE (20:4)*	-1.41E-01	6.44E-02	2.93E-02	-1.01E-01	6.26E-02	1.08E-01	-6.90E-02	6.10E-02	2.59E-01
leucylleucine	5.49E-02	6.49E-02	3.98E-01	4.89E-02	6.28E-02	4.37E-01	-5.15E-02	6.10E-02	3.99E-01
N2-acetyllysine	-1.23E-01	6.47E-02	5.92E-02	-9.43E-02	6.28E-02	1.34E-01	-1.89E-02	6.12E-02	7.58E-01
alpha-hydroxycaproate	-2.13E-02	6.54E-02	7.45E-01	-9.02E-02	6.31E-02	1.54E-01	-6.25E-02	6.14E-02	3.10E-01
3,4-dihydroxybutyrate	-5.91E-02	5.97E-02	3.23E-01	-5.44E-02	5.78E-02	3.47E-01	-7.34E-02	5.60E-02	1.91E-01
indoleacetylglutamine	-5.96E-02	5.71E-02	2.98E-01	-1.30E-01	5.48E-02	1.87E-02	-7.61E-03	5.38E-02	8.88E-01
hexanoylglutamine	-1.03E-01	6.23E-02	9.91E-02	-1.25E-01	6.01E-02	3.83E-02	-1.10E-01	5.85E-02	6.14E-02
N6-acetyllysine	6.90E-02	6.14E-02	2.62E-01	1.11E-01	5.92E-02	6.24E-02	1.89E-02	5.78E-02	7.44E-01
dihomo-linolenate (20:3n3 or n6)	-1.10E-01	6.36E-02	8.48E-02	-1.48E-01	6.12E-02	1.66E-02	-1.11E-01	5.97E-02	6.35E-02
mannitol/sorbitol	6.67E-02	6.35E-02	2.94E-01	1.01E-01	6.13E-02	1.01E-01	9.90E-02	5.95E-02	9.73E-02
tryptophan betaine	2.31E-01	5.85E-02	1.00E-04	2.23E-01	5.66E-02	1.06E-04	1.94E-01	5.53E-02	5.12E-04
4-vinylphenol sulfate	1.37E-01	6.49E-02	3.61E-02	1.98E-01	6.22E-02	1.61E-03	6.06E-02	6.14E-02	3.25E-01
4-ethylphenylsulfate	9.72E-02	6.58E-02	1.41E-01	1.59E-01	6.32E-02	1.25E-02	-6.34E-04	6.21E-02	9.92E-01
thymol sulfate	9.05E-02	6.49E-02	1.64E-01	-3.99E-02	6.30E-02	5.27E-01	4.81E-02	6.11E-02	4.32E-01
3-methyladipate	1.09E-01	5.83E-02	6.19E-02	1.24E-01	5.63E-02	2.80E-02	1.12E-01	5.47E-02	4.26E-02
pyrraline	2.08E-01	6.32E-02	1.10E-03	2.42E-01	6.06E-02	8.33E-05	1.72E-01	5.97E-02	4.26E-03
2-linoleoyl-GPE (18:2)*	-3.28E-02	6.27E-02	6.01E-01	2.19E-02	6.07E-02	7.18E-01	-5.45E-03	5.89E-02	9.26E-01
1-oleoyl-GPI (18:1)	1.23E-01	6.57E-02	6.24E-02	1.13E-02	6.40E-02	8.60E-01	1.06E-01	6.18E-02	8.67E-02
1-linoleoyl-GPI (18:2)*	-4.55E-02	6.36E-02	4.74E-01	-1.52E-01	6.09E-02	1.33E-02	-7.45E-03	5.98E-02	9.01E-01
1-palmitoleoyl-GPE (16:1)*	-1.03E-01	5.99E-02	8.58E-02	-1.73E-01	5.73E-02	2.73E-03	-1.07E-01	5.62E-02	5.88E-02
1-palmitoleoyl-GPI (16:1)*	-2.32E-02	6.28E-02	7.12E-01	-2.05E-01	5.96E-02	6.78E-04	-9.13E-02	5.88E-02	1.22E-01
desmethylnaproxen	3.49E-02	6.61E-02	5.98E-01	8.73E-02	6.38E-02	1.72E-01	1.08E-02	6.21E-02	8.62E-01
desmethylnaproxen sulfate	4.86E-02	6.54E-02	4.58E-01	1.09E-01	6.30E-02	8.48E-02	4.27E-02	6.15E-02	4.89E-01
2-hydroxyacetaminophen sulfate*	1.24E-01	6.60E-02	6.06E-02	1.60E-01	6.35E-02	1.21E-02	8.79E-02	6.22E-02	1.59E-01
2-methoxyacetaminophen sulfate*	9.33E-02	6.47E-02	1.50E-01	1.35E-01	6.23E-02	3.15E-02	8.83E-02	6.08E-02	1.48E-01
2-methoxyacetaminophen glucuronide*	9.09E-02	6.57E-02	1.67E-01	1.36E-01	6.33E-02	3.20E-02	7.22E-02	6.18E-02	2.44E-01
3-(cystein-S-yl)acetaminophen*	1.32E-01	6.75E-02	5.10E-02	1.42E-01	6.53E-02	3.06E-02	6.02E-02	6.38E-02	3.47E-01
o-cresol sulfate	3.15E-02	5.81E-02	5.89E-01	-7.62E-02	5.61E-02	1.75E-01	3.76E-02	5.46E-02	4.92E-01
dimethylarginine (SDMA + ADMA)	-1.74E-02	6.24E-02	7.81E-01	1.61E-02	6.04E-02	7.90E-01	-6.06E-02	5.86E-02	3.02E-01
gamma-glutamylalanine	-1.12E-01	6.43E-02	8.17E-02	-4.25E-02	6.25E-02	4.97E-01	-4.63E-02	6.07E-02	4.46E-01
N-acetylserine	2.93E-02	6.43E-02	6.49E-01	1.50E-01	6.16E-02	1.57E-02	1.61E-02	6.05E-02	7.91E-01
1-stearoyl-2-oleoyl-GPE (18:0/18:1)	1.51E-01	6.42E-02	1.92E-02	7.47E-02	6.26E-02	2.34E-01	1.72E-01	6.01E-02	4.48E-03
chiro-inositol	2.01E-01	6.68E-02	2.87E-03	2.03E-01	6.46E-02	1.87E-03	1.60E-01	6.31E-02	1.20E-02
4-allylphenol sulfate	1.68E-01	6.74E-02	1.30E-02	1.44E-01	6.54E-02	2.90E-02	9.94E-02	6.38E-02	1.20E-01
1-stearoyl-2-arachidonoyl-GPC (18:0/20:4)	-6.34E-02	6.28E-02	3.13E-01	-1.22E-01	6.04E-02	4.36E-02	-2.14E-02	5.91E-02	7.17E-01
1-palmitoyl-2-linoleoyl-GPE (16:0/18:2)	6.30E-02	6.50E-02	3.34E-01	-7.08E-03	6.30E-02	9.11E-01	4.63E-02	6.12E-02	4.50E-01
1-stearoyl-2-arachidonoyl-GPS (18:0/20:4)	-1.30E-02	6.60E-02	8.44E-01	2.67E-02	6.39E-02	6.77E-01	4.00E-03	6.21E-02	9.49E-01
sphinganine-1-phosphate	-5.19E-02	6.63E-02	4.34E-01	-2.33E-04	6.42E-02	9.97E-01	-2.24E-03	6.24E-02	9.71E-01
glycosyl-N-nervonoyl-sphingosine (d18:1/24:1)*	-3.18E-02	6.19E-02	6.09E-01	-7.75E-02	5.98E-02	1.96E-01	4.38E-02	5.82E-02	4.52E-01
glycosyl-N-stearoyl-sphingosine (d18:1/18:0)	-1.70E-01	6.13E-02	5.99E-03	-1.94E-01	5.90E-02	1.16E-03	-1.07E-01	5.81E-02	6.72E-02
cyclo(leu-pro)	-1.11E-01	6.48E-02	8.91E-02	-1.67E-01	6.23E-02	7.90E-03	-4.68E-02	6.12E-02	4.45E-01
succinylcarnitine (C4-DC)	5.02E-03	6.34E-02	9.37E-01	1.24E-01	6.09E-02	4.19E-02	-8.94E-03	5.96E-02	8.81E-01
bilirubin (E,E)*	-6.11E-02	6.08E-02	3.16E-01	-4.99E-02	5.88E-02	3.97E-01	-5.50E-03	5.72E-02	9.23E-01
bilirubin (E,Z or Z,E)*	-1.49E-02	6.14E-02	8.08E-01	-2.91E-02	5.94E-02	6.25E-01	1.26E-02	5.77E-02	8.28E-01
N-methylproline	2.45E-01	6.50E-02	1.99E-04	2.80E-01	6.22E-02	1.04E-05	1.72E-01	6.18E-02	5.72E-03
beta-cryptoxanthin	3.10E-01	5.83E-02	2.17E-07	2.43E-01	5.74E-02	3.16E-05	2.38E-01	5.57E-02	2.66E-05
5alpha-androstan-3beta,17beta-diol disulfate	-1.49E-01	5.28E-02	5.22E-03	-1.83E-01	5.06E-02	3.44E-04	-7.36E-02	5.01E-02	1.43E-01
5alpha-pregnan-3beta,20alpha-diol disulfate	-1.14E-01	6.63E-02	8.55E-02	-8.89E-02	6.43E-02	1.68E-01	-1.55E-01	6.20E-02	1.27E-02
glycocholenate sulfate*	-1.13E-01	5.94E-02	5.86E-02	-7.95E-02	5.77E-02	1.70E-01	-1.05E-01	5.59E-02	6.13E-02
taurocholenate sulfate*	-3.33E-02	6.46E-02	6.06E-01	-9.49E-02	6.23E-02	1.29E-01	-9.93E-02	6.05E-02	1.02E-01
androstenediol (3beta,17beta) disulfate (1)	-1.48E-01	5.67E-02	9.47E-03	-2.41E-01	5.36E-02	1.06E-05	-9.73E-02	5.36E-02	7.07E-02
pregnenediol disulfate (C21H34O8S2)*	-1.40E-01	5.81E-02	1.68E-02	-1.60E-01	5.60E-02	4.53E-03	-1.34E-01	5.46E-02	1.45E-02
androstenediol (3beta,17beta) disulfate (2)	-2.00E-01	5.52E-02	3.54E-04	-1.48E-01	5.39E-02	6.33E-03	-1.71E-01	5.21E-02	1.17E-03
21-hydroxypregnenolone disulfate	-1.27E-01	6.18E-02	4.08E-02	-1.09E-01	5.99E-02	6.97E-02	-1.25E-01	5.81E-02	3.28E-02
5alpha-androstan-3alpha,17alpha-diol monosulfate	-1.17E-01	5.79E-02	4.39E-02	-5.58E-03	5.64E-02	9.21E-01	-1.09E-01	5.44E-02	4.62E-02
5alpha-pregnan-3beta,20beta-diol monosulfate (1)	-1.35E-01	6.38E-02	3.48E-02	-9.08E-02	6.20E-02	1.44E-01	-1.77E-01	5.95E-02	3.16E-03
5alpha-pregnan-3beta,20alpha-diol monosulfate (2)	-8.89E-02	6.35E-02	1.62E-01	-2.88E-02	6.16E-02	6.41E-01	-1.43E-01	5.93E-02	1.62E-02
5alpha-pregnan-diol disulfate	-1.72E-01	6.21E-02	6.07E-03	-1.28E-01	6.04E-02	3.47E-02	-1.50E-01	5.85E-02	1.11E-02
5alpha-androstan-3alpha,17beta-diol disulfate	-7.81E-02	5.58E-02	1.63E-01	-5.13E-02	5.41E-02	3.44E-01	-1.02E-01	5.23E-02	5.23E-02
5alpha-androstan-3alpha,17beta-diol monosulfate (1)	-1.56E-01	6.14E-02	1.13E-02	-1.51E-01	5.94E-02	1.14E-02	-9.45E-02	5.81E-02	1.05E-01
5alpha-androstan-3beta,17alpha-diol disulfate	-1.10E-01	6.27E-02	7.97E-02	-5.15E-02	6.09E-02	3.98E-01	-6.51E-02	5.92E-02	2.72E-01
5alpha-androstan-3beta,17beta-diol monosulfate (2)	-1.36E-01	5.85E-02	2.10E-02	-1.49E-01	5.65E-02	8.87E-03	-7.29E-02	5.54E-02	1.89E-01
androstenediol (3alpha, 17alpha) monosulfate (2)	-1.49E-01	5.52E-02	7.52E-03	-1.89E-02	5.41E-02	7.27E-01	-1.42E-01	5.19E-02	6.57E-03
androstenediol (3alpha, 17alpha) monosulfate (3)	-1.87E-01	5.41E-02	6.31E-04	-1.50E-01	5.27E-02	4.69E-03	-1.61E-01	5.10E-02	1.75E-03
androstenediol (3beta,17beta) monosulfate (1)	-1.37E-01	5.69E-02	1.67E-02	-1.57E-01	5.49E-02	4.65E-03	-1.12E-01	5.37E-02	3.79E-02
androstenediol (3beta,17beta) monosulfate (2)	-1.28E-01	6.17E-02	3.85E-02	-1.30E-01	5.97E-02	2.99E-02	-4.62E-02	5.84E-02	4.29E-01
5alpha-pregnan-3beta-ol,20-one sulfate	-1.75E-02	6.40E-02	7.84E-01	-3.12E-02	6.19E-02	6.15E-01	-4.28E-02	6.01E-02	4.77E-01
4-hydroxycoumarin	1.27E-01	6.38E-02	4.79E-02	7.87E-02	6.21E-02	2.06E-01	3.27E-02	6.04E-02	5.88E-01
1-docosahexaenoyl-GPE (22:6)*	2.55E-02	6.10E-02	6.77E-01	-1.25E-01	5.86E-02	3.32E-02	9.92E-02	5.71E-02	8.35E-02
2-docosahexaenoyl-GPE (22:6)*	1.07E-02	6.64E-02	8.72E-01	-7.42E-02	6.41E-02	2.48E-01	2.14E-02	6.24E-02	7.31E-01
1-docosapentaenoyl-GPC (22:5n3)*	-2.69E-02	6.47E-02	6.77E-01	1.36E-02	6.26E-02	8.28E-01	2.60E-02	6.08E-02	6.69E-01

Supplementary Table 4: Parameter estimates for metabolome-wide association studies for diet-metabolite associations for each of: HEI-15, DASH and AMED diet, controlling for BMI

1-docosapentaenoyl-GPE (22:5n6)*	-2.16E-01	6.16E-02	5.29E-04	-2.09E-01	5.96E-02	5.28E-04	-1.87E-01	5.81E-02	1.48E-03
pregnenediol sulfate (C21H34O5S)*	-1.09E-01	5.91E-02	6.67E-02	-9.91E-02	5.72E-02	8.44E-02	-1.30E-01	5.53E-02	1.94E-02
2-hydroxyglutarate	-7.55E-02	6.66E-02	2.58E-01	-1.46E-01	6.40E-02	2.28E-02	1.07E-02	6.27E-02	8.65E-01
gamma-CEHC	-8.04E-02	6.30E-02	2.03E-01	5.68E-02	6.11E-02	3.53E-01	-5.90E-02	5.93E-02	3.21E-01
N-acetyl-beta-alanine	1.16E-01	5.90E-02	5.08E-02	7.49E-02	5.73E-02	1.92E-01	1.18E-01	5.54E-02	3.45E-02
5-methylthioribose**	5.43E-02	6.22E-02	3.83E-01	1.43E-01	5.97E-02	1.70E-02	4.65E-02	5.85E-02	4.28E-01
sphingomyelin (d18:1/18:1, d18:2/18:0)	-2.09E-01	5.62E-02	2.46E-04	-2.11E-01	5.43E-02	1.26E-04	-1.05E-01	5.38E-02	5.10E-02
palmitoyl sphingomyelin (d18:1/16:0)	-4.68E-02	6.24E-02	4.54E-01	-6.83E-02	6.03E-02	2.58E-01	-9.78E-03	5.87E-02	8.68E-01
cysteine sulfinic acid	-1.87E-01	6.02E-02	2.11E-03	-2.27E-01	5.77E-02	1.03E-04	-1.16E-01	5.72E-02	4.29E-02
3-hydroxyhippurate	1.29E-01	6.36E-02	4.39E-02	1.33E-01	6.15E-02	3.13E-02	1.41E-01	5.96E-02	1.89E-02
16a-hydroxy DHEA 3-sulfate	-1.25E-01	6.19E-02	4.49E-02	-4.91E-02	6.02E-02	4.16E-01	-1.57E-01	5.78E-02	6.91E-03
17alpha-hydroxypregnenolone 3-sulfate	-1.25E-01	5.94E-02	3.69E-02	-1.05E-01	5.76E-02	6.92E-02	-1.81E-01	5.53E-02	1.19E-03
pregnenolone sulfate	-1.20E-01	6.04E-02	4.74E-02	-1.24E-01	5.84E-02	3.52E-02	-1.64E-01	5.64E-02	3.87E-03
5-HEPE	1.50E-02	6.42E-02	8.15E-01	-4.33E-02	6.21E-02	4.87E-01	8.35E-02	6.02E-02	1.66E-01
5-HETE	-8.13E-02	6.52E-02	2.13E-01	-1.37E-01	6.27E-02	2.96E-02	-5.58E-02	6.13E-02	3.64E-01
andro steroid monosulfate C19H28O6S (1)*	-1.21E-01	6.04E-02	4.67E-02	-7.56E-02	5.87E-02	1.99E-01	-1.57E-01	5.64E-02	5.78E-03
ergothioneine	1.70E-01	6.10E-02	5.65E-03	1.05E-02	5.99E-02	8.61E-01	1.49E-01	5.75E-02	9.80E-03
1-margaroyl-GPE (17:0)*	5.90E-02	6.33E-02	3.52E-01	9.79E-02	6.11E-02	1.10E-01	-2.51E-02	5.96E-02	6.74E-01
1-pentadecanoyl-GPC (15:0)*	-7.17E-03	5.87E-02	9.03E-01	9.74E-02	5.65E-02	8.56E-02	-1.09E-01	5.48E-02	4.74E-02
S-methylmethionine	1.70E-01	6.35E-02	7.81E-03	2.14E-01	6.09E-02	5.14E-04	2.55E-01	5.85E-02	1.79E-05
indole-3-carboxylate	1.45E-01	6.51E-02	2.66E-02	1.34E-01	6.31E-02	3.48E-02	7.50E-03	6.18E-02	9.03E-01
13-HODE + 9-HODE	-5.06E-02	6.25E-02	4.18E-01	-4.72E-02	6.05E-02	4.36E-01	-9.57E-03	5.88E-02	8.71E-01
tridecenedioate (C13:1-DC)*	-2.34E-01	5.96E-02	1.10E-04	-1.10E-01	5.89E-02	6.39E-02	-2.51E-01	5.56E-02	9.20E-06
N-acetyl-3-methylhistidine*	-7.85E-02	6.59E-02	2.34E-01	-7.49E-02	6.38E-02	2.41E-01	-7.56E-02	6.19E-02	2.23E-01
4-cholesten-3-one	6.24E-02	6.46E-02	3.35E-01	-7.10E-02	6.25E-02	2.57E-01	3.36E-02	6.08E-02	5.81E-01
cinnamoylglycine	1.51E-01	6.00E-02	1.25E-02	1.56E-01	5.79E-02	7.68E-03	8.16E-02	5.68E-02	1.52E-01
stearoyl ethanolamide	-5.04E-02	6.22E-02	4.19E-01	-7.52E-02	6.01E-02	2.12E-01	-2.77E-02	5.85E-02	6.37E-01
cis-4-decenoylcarnitine (C10:1)	-1.16E-01	6.38E-02	6.95E-02	-1.15E-01	6.17E-02	6.34E-02	-3.13E-02	6.03E-02	6.04E-01
2S,3R-dihydroxybutyrate	-2.12E-02	5.79E-02	7.14E-01	-8.33E-02	5.58E-02	1.37E-01	1.63E-02	5.44E-02	7.65E-01
phenylalanylphenylalanine	1.26E-01	6.54E-02	5.52E-02	1.64E-01	6.30E-02	9.56E-03	5.59E-02	6.18E-02	3.67E-01
(12 or 13)-methylmyristate (a15:0 or i15:0)	-2.00E-01	6.02E-02	1.00E-03	-1.37E-01	5.89E-02	2.11E-02	-1.91E-01	5.66E-02	8.34E-04
(16 or 17)-methylstearate (a19:0 or i19:0)	-1.93E-01	6.26E-02	2.28E-03	-2.10E-01	6.03E-02	5.65E-04	-1.82E-01	5.89E-02	2.18E-03
2R,3R-dihydroxybutyrate	-6.48E-02	6.20E-02	2.97E-01	-3.36E-02	6.01E-02	5.77E-01	-5.41E-02	5.83E-02	3.54E-01
4-methylbenzenesulfonate	-5.37E-02	6.36E-02	3.99E-01	6.13E-03	6.16E-02	9.21E-01	-4.36E-02	5.98E-02	4.66E-01
alpha-ketoglutaramate*	1.32E-01	6.07E-02	3.09E-02	1.70E-01	5.83E-02	3.78E-03	9.43E-02	5.72E-02	1.01E-01
metformin	3.60E-02	7.24E-02	6.19E-01	4.40E-02	7.00E-02	5.30E-01	-3.30E-02	6.80E-02	6.28E-01
ibuprofen acyl glucuronide	3.99E-02	6.11E-02	5.14E-01	-8.27E-02	5.90E-02	1.62E-01	1.22E-03	5.75E-02	9.83E-01
3-hydroxyquinine	-3.12E-02	7.25E-02	6.67E-01	-3.61E-02	7.02E-02	6.08E-01	-1.74E-02	6.82E-02	7.99E-01
2,3-dihydroxyisovalerate	8.83E-02	6.02E-02	1.44E-01	-1.08E-01	5.81E-02	6.31E-02	2.09E-02	5.68E-02	7.13E-01
allopurinol riboside	-1.50E-01	7.16E-02	3.70E-02	2.04E-03	6.98E-02	9.77E-01	-1.40E-01	6.73E-02	3.77E-02
3-methylglutaconate	1.10E-02	5.96E-02	8.54E-01	1.22E-01	5.72E-02	3.30E-02	-4.59E-02	5.59E-02	4.12E-01
cysteinylglycine disulfide*	-1.69E-01	5.84E-02	4.16E-03	-1.29E-01	5.68E-02	2.42E-02	-1.55E-01	5.49E-02	5.08E-03
isoursodeoxycholate	-1.48E-01	6.52E-02	2.37E-02	-1.18E-01	6.33E-02	6.24E-02	-5.26E-02	6.18E-02	3.96E-01
oxypurinol	-1.50E-01	7.16E-02	3.70E-02	2.04E-03	6.98E-02	9.77E-01	-1.40E-01	6.73E-02	3.77E-02
formiminoglutamate	-9.42E-02	6.12E-02	1.25E-01	-1.57E-01	5.87E-02	7.92E-03	-5.96E-02	5.77E-02	3.02E-01
hydantoin-5-propionate	1.06E-01	6.30E-02	9.25E-02	2.13E-01	6.00E-02	4.57E-04	7.56E-03	5.95E-02	8.99E-01
sulfate*	1.11E-01	6.64E-02	9.49E-02	1.20E-01	6.42E-02	6.31E-02	6.96E-02	6.26E-02	2.67E-01
4-hydroxy-2-oxoglutaric acid	2.35E-02	6.36E-02	7.12E-01	7.84E-02	6.14E-02	2.03E-01	8.26E-03	5.98E-02	8.90E-01
4-hydroxyglutamate	3.45E-02	5.98E-02	5.65E-01	4.47E-02	5.79E-02	4.41E-01	-1.37E-02	5.63E-02	8.07E-01
L-urobilin	-4.51E-03	6.42E-02	9.44E-01	-2.30E-02	6.21E-02	7.12E-01	-1.13E-01	6.00E-02	6.00E-02
pantoate	1.31E-01	6.54E-02	4.56E-02	1.47E-01	6.31E-02	2.06E-02	3.39E-02	6.19E-02	5.85E-01
hydroxycotinine	-5.96E-02	4.74E-02	2.09E-01	-1.11E-01	4.55E-02	1.50E-02	-2.94E-02	4.46E-02	5.10E-01
cotinine N-oxide	-5.27E-02	4.82E-02	2.76E-01	-1.04E-01	4.64E-02	2.62E-02	-2.26E-02	4.54E-02	6.19E-01
omeprazole	2.66E-02	7.18E-02	7.11E-01	3.10E-02	6.95E-02	6.56E-01	-3.92E-03	6.75E-02	9.54E-01
atenolol	8.79E-02	5.67E-02	1.22E-01	1.13E-01	5.47E-02	4.01E-02	-7.36E-02	5.34E-02	1.69E-01
diphenhydramine	-5.71E-02	6.66E-02	3.92E-01	4.04E-02	6.45E-02	5.32E-01	-2.89E-02	6.27E-02	6.45E-01
hydrochlorothiazide	4.76E-02	6.10E-02	4.36E-01	9.72E-03	5.91E-02	8.70E-01	4.14E-02	5.74E-02	4.72E-01
pseudoephedrine	-7.03E-18	1.19E-17	5.54E-01	-2.63E-17	1.14E-17	2.16E-02	-7.49E-18	1.12E-17	5.02E-01
S-methylcysteine	1.14E-02	6.44E-02	8.60E-01	5.43E-02	6.23E-02	3.84E-01	1.03E-01	6.03E-02	8.81E-02
androsterone glucuronide	-9.23E-02	5.52E-02	9.58E-02	2.60E-02	5.37E-02	6.28E-01	-8.61E-02	5.19E-02	9.87E-02
argininate*	2.35E-01	6.23E-02	1.98E-04	2.00E-01	6.07E-02	1.11E-03	2.13E-01	5.87E-02	3.28E-04
solanidine	-1.42E-02	6.55E-02	8.29E-01	-4.49E-02	6.33E-02	4.79E-01	6.78E-02	6.14E-02	2.71E-01
2-oxoarginine*	2.00E-01	6.13E-02	1.25E-03	2.06E-01	5.92E-02	5.82E-04	1.28E-01	5.83E-02	2.87E-02
cis-4-decenoate (10:1n6)*	-1.14E-01	6.42E-02	7.57E-02	-1.66E-01	6.17E-02	7.57E-03	-4.92E-02	6.06E-02	4.17E-01
atorvastatin (lipitor)	4.66E-02	6.62E-02	4.82E-01	-1.42E-02	6.42E-02	8.25E-01	1.36E-02	6.23E-02	8.27E-01
sertraline	-2.76E-02	7.25E-02	7.03E-01	-8.47E-02	7.00E-02	2.27E-01	-2.57E-02	6.81E-02	7.07E-01
gabapentin	4.93E-03	6.68E-02	9.41E-01	-1.71E-02	6.47E-02	7.91E-01	-1.22E-03	6.28E-02	9.85E-01
venlafaxine	7.90E-02	7.21E-02	2.74E-01	-1.30E-02	7.00E-02	8.53E-01	1.07E-01	6.77E-02	1.15E-01
quetiapine	4.94E-02	7.07E-02	4.85E-01	-3.66E-02	6.84E-02	5.93E-01	-7.31E-02	6.64E-02	2.71E-01
ethyl glucuronide	4.65E-02	6.10E-02	4.47E-01	-2.26E-01	5.75E-02	1.08E-04	3.12E-02	5.74E-02	5.87E-01
1-behenoyl-GPC (22:0)	3.27E-01	5.96E-02	9.15E-08	2.45E-01	5.89E-02	4.37E-05	3.26E-01	5.57E-02	1.36E-08
1-erucoyl-GPC (22:1)*	2.15E-01	5.97E-02	3.86E-04	7.93E-02	5.89E-02	1.79E-01	2.06E-01	5.61E-02	2.96E-04
1-adrenoyl-GPC (22:4)*	-1.33E-01	6.04E-02	2.91E-02	-9.70E-02	5.87E-02	9.95E-02	-1.28E-01	5.68E-02	2.47E-02
1-lignoceroyl-GPC (24:0)	3.45E-01	5.93E-02	1.66E-08	2.64E-01	5.87E-02	1.02E-05	3.77E-01	5.46E-02	3.19E-11
1-nervonoyl-GPC (24:1n9)*	2.35E-01	5.84E-02	7.22E-05	4.67E-02	5.81E-02	4.22E-01	2.35E-01	5.47E-02	2.37E-05
1-(1-enyl-palmitoyl)-GPC (P-16:0)*	5.44E-02	6.45E-02	4.00E-01	-1.29E-02	6.25E-02	8.37E-01	4.02E-02	6.07E-02	5.08E-01
1-(1-enyl-oleoyl)-GPC (P-18:1)*	1.28E-01	6.15E-02	3.78E-02	3.40E-02	6.00E-02	5.71E-01	1.61E-01	5.75E-02	5.35E-03
1-(1-enyl-stearoyl)-GPC (P-18:0)*	-7.96E-03	6.14E-02	8.97E-01	-5.38E-03	5.95E-02	9.28E-01	-8.86E-03	5.78E-02	8.78E-01
1-methyl-5-imidazoleacetate	2.16E-02	6.41E-02	7.36E-01	4.27E-02	6.20E-02	4.91E-01	-7.01E-02	6.01E-02	2.45E-01
glycoursodeoxycholate	-8.71E-02	6.59E-02	1.87E-01	-1.11E-01	6.37E-02	8.12E-02	-8.56E-02	6.20E-02	1.69E-01
tauroursodeoxycholate	-8.90E-02	6.65E-02	1.82E-01	-1.10E-01	6.42E-02	8.65E-02	-7.45E-02	6.25E-02	2.34E-01
S-methylcysteine sulfoxide	1.49E-01	6.21E-02	1.68E-02	1.64E-01	5.99E-02	6.41E-03	1.71E-01	5.80E-02	3.52E-03

benzoylecgonine	-5.29E-02	5.37E-02	3.25E-01	-3.41E-02	5.20E-02	5.12E-01	1.48E-02	5.05E-02	7.70E-01
(14 or 15)-methylpalmitate (a17:0 or i17:0)	-2.35E-01	6.11E-02	1.53E-04	-2.04E-01	5.95E-02	6.90E-04	-2.35E-01	5.73E-02	5.29E-05
eicosanedioate (C20-DC)	-1.59E-01	6.52E-02	1.53E-02	-9.56E-02	6.36E-02	1.34E-01	-9.34E-02	6.17E-02	1.31E-01
docosadioate (C22-DC)	-9.95E-02	6.73E-02	1.41E-01	-2.30E-02	6.54E-02	7.25E-01	-9.49E-02	6.33E-02	1.35E-01
16-hydroxypalmitate	-1.72E-01	6.37E-02	7.26E-03	-1.57E-01	6.18E-02	1.18E-02	-1.41E-01	6.01E-02	2.00E-02
quinine	-3.12E-02	7.25E-02	6.67E-01	-3.61E-02	7.02E-02	6.08E-01	-1.74E-02	6.82E-02	7.99E-01
oleoyl-linoleoyl-glycerol (18:1/18:2) [1]	8.72E-02	6.24E-02	1.64E-01	7.50E-02	6.05E-02	2.16E-01	3.69E-02	5.89E-02	5.31E-01
oleoyl-linoleoyl-glycerol (18:1/18:2) [2]	1.21E-01	6.26E-02	5.36E-02	8.61E-02	6.08E-02	1.58E-01	7.61E-02	5.91E-02	1.99E-01
1-(1-enyl-palmitoyl)-GPE (P-16:0)*	-2.90E-02	6.47E-02	6.54E-01	-5.03E-02	6.26E-02	4.22E-01	7.76E-03	6.09E-02	8.99E-01
1-(1-enyl-stearoyl)-GPE (P-18:0)*	-5.27E-02	6.39E-02	4.10E-01	-5.60E-02	6.18E-02	3.66E-01	2.04E-02	6.01E-02	7.34E-01
2-stearoyl-GPI (18:0)*	5.95E-02	6.36E-02	3.51E-01	2.51E-02	6.17E-02	6.84E-01	7.74E-02	5.97E-02	1.96E-01
alpha-CEHC glucuronide*	1.45E-01	6.54E-02	2.71E-02	1.08E-01	6.36E-02	8.95E-02	8.39E-03	6.21E-02	8.93E-01
2-oxindole-3-acetate	8.97E-02	6.64E-02	1.78E-01	7.84E-02	6.44E-02	2.24E-01	7.99E-02	6.25E-02	2.02E-01
N-oleoyltaurine	1.10E-01	6.53E-02	9.42E-02	4.97E-02	6.34E-02	4.34E-01	5.91E-02	6.16E-02	3.38E-01
linoleoylcarnitine (C18:2)*	-5.16E-02	6.35E-02	4.17E-01	-5.46E-02	6.15E-02	3.75E-01	1.74E-02	5.98E-02	7.71E-01
isoleucylglycine	-1.81E-02	6.47E-02	7.80E-01	5.21E-02	6.26E-02	4.06E-01	3.53E-02	6.08E-02	5.62E-01
leucylglycine	-3.61E-02	6.55E-02	5.82E-01	5.75E-02	6.33E-02	3.65E-01	4.61E-02	6.16E-02	4.55E-01
phenylalanylisoleucine	2.56E-02	6.47E-02	6.93E-01	8.83E-02	6.24E-02	1.58E-01	3.91E-02	6.08E-02	5.21E-01
phenylalanylleucine	5.62E-02	6.85E-02	4.13E-01	5.97E-02	6.63E-02	3.69E-01	1.57E-02	6.45E-02	8.08E-01
N-palmitoyltaurine	7.39E-02	6.45E-02	2.53E-01	3.59E-02	6.26E-02	5.67E-01	3.52E-05	6.08E-02	1.00E+00
N-stearoyltaurine	-7.00E-02	6.52E-02	2.84E-01	-3.86E-02	6.32E-02	5.42E-01	-5.60E-02	6.13E-02	3.62E-01
2-O-methylascorbic acid	1.25E-01	5.90E-02	3.50E-02	1.10E-01	5.72E-02	5.60E-02	1.62E-02	5.59E-02	7.73E-01
beta-citrylglutamate	-8.88E-02	6.53E-02	1.75E-01	-7.89E-02	6.32E-02	2.13E-01	-7.91E-04	6.16E-02	9.90E-01
rosuvastatin	5.18E-03	6.82E-02	9.39E-01	4.37E-03	6.60E-02	9.47E-01	4.87E-02	6.40E-02	4.47E-01
trimethylamine N-oxide	7.25E-03	6.41E-02	9.10E-01	1.08E-02	6.20E-02	8.62E-01	3.16E-02	6.02E-02	6.00E-01
N6-methyllysine	-2.03E-02	5.83E-02	7.27E-01	-6.21E-02	5.63E-02	2.71E-01	4.68E-02	5.47E-02	3.94E-01
dihydroferulate	1.28E-01	6.44E-02	4.82E-02	1.53E-01	6.21E-02	1.44E-02	1.00E-01	6.07E-02	1.00E-01
imidazole propionate	-2.10E-02	6.40E-02	7.43E-01	-4.11E-02	6.19E-02	5.07E-01	2.57E-02	6.02E-02	6.70E-01
citalopram/escitalopram	-9.96E-02	6.86E-02	1.48E-01	-2.26E-02	6.66E-02	7.35E-01	-1.95E-02	6.47E-02	7.64E-01
duloxetine	-7.03E-18	1.19E-17	5.54E-01	-2.63E-17	1.14E-17	2.16E-02	-7.49E-18	1.12E-17	5.02E-01
milnacipran	4.19E-02	7.26E-02	5.64E-01	1.21E-01	7.00E-02	8.53E-02	-6.69E-02	6.82E-02	3.28E-01
pregnanediol-3-glucuronide	-2.99E-02	6.31E-02	6.35E-01	-5.80E-02	6.10E-02	3.43E-01	-9.87E-02	5.91E-02	9.59E-02
(15:2)-anacardic acid	4.12E-02	6.97E-02	5.55E-01	4.40E-02	6.75E-02	5.15E-01	-3.14E-02	6.56E-02	6.32E-01
alliin	1.48E-02	6.29E-02	8.14E-01	-7.36E-02	6.07E-02	2.26E-01	1.00E-01	5.88E-02	8.90E-02
seryltyrosine	1.19E-01	6.77E-02	8.03E-02	1.24E-01	6.54E-02	5.87E-02	7.77E-02	6.38E-02	2.25E-01
histidylalanine	6.33E-02	6.67E-02	3.43E-01	6.72E-02	6.45E-02	2.99E-01	5.35E-02	6.27E-02	3.94E-01
phenylalanylglycine	8.00E-02	6.68E-02	2.32E-01	4.93E-02	6.48E-02	4.48E-01	5.97E-02	6.29E-02	3.43E-01
phenylalanylmethionine	1.19E-01	6.63E-02	7.32E-02	1.41E-01	6.40E-02	2.84E-02	3.44E-02	6.27E-02	5.84E-01
phenylalanyltryptophan	-7.42E-02	6.42E-02	2.48E-01	-1.43E-01	6.17E-02	2.10E-02	-4.99E-02	6.04E-02	4.10E-01
tyrosyllysine	1.00E-01	6.77E-02	1.40E-01	1.39E-01	6.53E-02	3.39E-02	7.95E-02	6.38E-02	2.13E-01
tryptophylasparagine	1.16E-01	6.79E-02	8.78E-02	9.83E-02	6.58E-02	1.36E-01	4.71E-02	6.41E-02	4.63E-01
aspartylaspartate	4.27E-02	6.60E-02	5.18E-01	1.89E-02	6.39E-02	7.67E-01	1.11E-01	6.17E-02	7.28E-02
valylalanine	2.93E-02	6.46E-02	6.51E-01	5.86E-02	6.24E-02	3.49E-01	7.22E-02	6.06E-02	2.34E-01
valylglycine	-2.50E-02	6.63E-02	7.07E-01	6.25E-02	6.41E-02	3.30E-01	2.24E-02	6.23E-02	7.20E-01
valylphenylalanine	8.43E-02	6.49E-02	1.95E-01	1.35E-01	6.25E-02	3.16E-02	9.08E-02	6.10E-02	1.37E-01
methionylalanine	3.59E-02	6.64E-02	5.89E-01	6.42E-02	6.42E-02	3.18E-01	3.46E-02	6.24E-02	5.80E-01
N-palmitoylglycine	-6.39E-02	6.50E-02	3.26E-01	-1.10E-01	6.27E-02	8.05E-02	-9.33E-02	6.10E-02	1.27E-01
mannonate*	-5.13E-02	6.11E-02	4.02E-01	9.56E-03	5.92E-02	8.72E-01	-8.64E-02	5.73E-02	1.33E-01
norfluoxetine	8.50E-03	6.97E-02	9.03E-01	-5.63E-02	6.74E-02	4.04E-01	4.11E-02	6.55E-02	5.31E-01
2-stearoyl-GPE (18:0)*	-8.69E-02	6.58E-02	1.88E-01	-1.09E-01	6.35E-02	8.67E-02	-7.24E-02	6.19E-02	2.43E-01
(R)-3-hydroxybutyrylcarnitine	-1.28E-01	6.37E-02	4.57E-02	-1.40E-01	6.16E-02	2.34E-02	-8.21E-02	6.02E-02	1.73E-01
N-octanoylglycine	-8.06E-02	6.59E-02	2.22E-01	-3.46E-02	6.39E-02	5.89E-01	-1.05E-01	6.18E-02	9.05E-02
doxylamine	-3.98E-02	5.35E-02	4.57E-01	-6.31E-02	5.17E-02	2.23E-01	-3.15E-02	5.03E-02	5.31E-01
triamterene	3.01E-02	7.24E-02	6.78E-01	-2.11E-03	7.01E-02	9.76E-01	-4.96E-03	6.81E-02	9.42E-01
diltiazem	2.03E-02	7.21E-02	7.79E-01	7.41E-02	6.96E-02	2.88E-01	5.52E-02	6.77E-02	4.15E-01
fexofenadine	-1.71E-02	6.79E-02	8.01E-01	-7.37E-02	6.56E-02	2.62E-01	-1.18E-01	6.34E-02	6.41E-02
verapamil	6.81E-02	4.10E-02	9.79E-02	3.68E-02	3.98E-02	3.57E-01	3.80E-02	3.87E-02	3.28E-01
N-acetylcarnosine	-9.25E-03	5.06E-02	8.55E-01	8.22E-02	4.87E-02	9.27E-02	-3.41E-02	4.75E-02	4.73E-01
margaroylcarnitine (C17)*	-2.18E-01	6.09E-02	4.11E-04	-1.72E-01	5.94E-02	4.07E-03	-1.86E-01	5.75E-02	1.39E-03
N-methyltaurine	1.38E-01	6.63E-02	3.78E-02	-1.19E-02	6.47E-02	8.54E-01	2.01E-01	6.17E-02	1.24E-03
histidine betaine (hercynine)*	1.46E-01	6.38E-02	2.25E-02	-3.52E-03	6.23E-02	9.55E-01	2.10E-01	5.92E-02	4.60E-04
glycohyocholate	5.45E-02	6.43E-02	3.98E-01	4.51E-02	6.23E-02	4.69E-01	1.30E-02	6.05E-02	8.30E-01
2-hydroxydecanoate	1.53E-01	6.38E-02	1.75E-02	1.28E-01	6.20E-02	3.95E-02	5.17E-02	6.06E-02	3.94E-01
3-methyl catechol sulfate (2)	6.04E-02	6.02E-02	3.16E-01	1.85E-03	5.83E-02	9.75E-01	1.43E-02	5.66E-02	8.01E-01
4-methylcatechol sulfate	1.90E-01	6.38E-02	3.10E-03	1.87E-01	6.18E-02	2.73E-03	8.56E-02	6.07E-02	1.60E-01
3-methyl catechol sulfate (1)	-2.56E-02	6.25E-02	6.83E-01	-7.86E-02	6.03E-02	1.94E-01	-4.70E-02	5.87E-02	4.24E-01
2-hydroxyibuprofen	-1.38E-02	6.26E-02	8.26E-01	-1.52E-01	5.99E-02	1.16E-02	-4.98E-02	5.88E-02	3.98E-01
carboxyibuprofen	-1.17E-01	6.23E-02	6.08E-02	-1.51E-01	6.00E-02	1.22E-02	-1.08E-01	5.86E-02	6.56E-02
O-desmethylvenlafaxine	2.79E-02	7.31E-02	7.04E-01	-3.83E-02	7.08E-02	5.89E-01	1.27E-01	6.84E-02	6.41E-02
zolpidem	-8.38E-02	7.21E-02	2.46E-01	-1.58E-01	6.93E-02	2.32E-02	-4.04E-02	6.79E-02	5.52E-01
warfarin	5.67E-02	7.18E-02	4.31E-01	9.79E-02	6.93E-02	1.59E-01	-1.23E-01	6.72E-02	6.83E-02
3b-hydroxy-5-cholenoic acid	2.32E-02	6.20E-02	7.08E-01	6.28E-02	5.99E-02	2.95E-01	-2.89E-02	5.83E-02	6.21E-01
guaiacol sulfate	2.98E-01	6.05E-02	1.46E-06	2.43E-01	5.93E-02	5.39E-05	1.86E-01	5.82E-02	1.58E-03
2-aminooctanoate	8.50E-02	6.22E-02	1.73E-01	9.40E-02	6.02E-02	1.19E-01	6.06E-02	5.86E-02	3.02E-01
furosemide	1.18E-01	6.24E-02	5.93E-02	1.55E-01	6.01E-02	1.03E-02	1.01E-01	5.87E-02	8.81E-02
gamma-CEHC glucuronide*	-7.73E-02	6.57E-02	2.40E-01	8.37E-02	6.35E-02	1.89E-01	-6.96E-02	6.17E-02	2.61E-01
dimethyl sulfone	1.16E-02	6.67E-02	8.62E-01	8.00E-02	6.44E-02	2.15E-01	-4.64E-02	6.26E-02	4.59E-01
N-acetyl-1-methylhistidine*	-2.04E-01	6.14E-02	1.00E-03	-1.90E-01	5.95E-02	1.54E-03	-1.20E-01	5.84E-02	4.11E-02
indolin-2-one	-1.53E-02	6.80E-02	8.22E-01	7.58E-02	6.57E-02	2.49E-01	-2.86E-02	6.39E-02	6.55E-01
2,8-quinolinediol sulfate	4.91E-04	6.45E-02	9.94E-01	5.93E-02	6.23E-02	3.42E-01	-4.80E-03	6.06E-02	9.37E-01
2-aminophenol sulfate	1.33E-01	6.32E-02	3.66E-02	2.40E-01	5.99E-02	8.21E-05	9.02E-02	5.96E-02	1.31E-01
3-acetylphenol sulfate	5.92E-02	6.48E-02	3.62E-01	7.07E-02	6.27E-02	2.61E-01	9.63E-02	6.08E-02	1.14E-01

Supplementary Table 4: Parameter estimates for metabolome-wide association studies for diet-metabolite associations for each of: HEI-15, DASH and AMED diet, controlling for BMI

sphingomyelin (d18:1/14:0, d16:1/16:0)*	-5.85E-02	5.92E-02	3.24E-01	-4.78E-02	5.73E-02	4.05E-01	-1.12E-01	5.53E-02	4.38E-02
sphingomyelin (d18:2/16:0, d18:1/16:1)*	-1.06E-01	6.00E-02	7.90E-02	-1.01E-01	5.81E-02	8.24E-02	-6.58E-03	5.67E-02	9.08E-01
3-hydroxyadipate	-5.36E-02	6.32E-02	3.97E-01	4.28E-02	6.12E-02	4.85E-01	-2.24E-02	5.95E-02	7.07E-01
3-hydroxycotinine glucuronide	-4.54E-02	4.80E-02	3.46E-01	-7.73E-02	4.63E-02	9.62E-02	-1.22E-02	4.52E-02	7.87E-01
6-oxopiperidine-2-carboxylate	6.46E-02	6.46E-02	3.19E-01	1.21E-01	6.23E-02	5.32E-02	9.29E-02	6.06E-02	1.27E-01
S-allylcysteine	-1.02E-02	6.34E-02	8.72E-01	-7.91E-02	6.12E-02	1.97E-01	7.66E-02	5.94E-02	1.98E-01
allopurinol	-8.94E-02	7.21E-02	2.16E-01	1.39E-02	7.00E-02	8.43E-01	-1.16E-01	6.76E-02	8.65E-02
N-delta-acetylornithine	2.95E-01	5.53E-02	1.92E-07	1.96E-01	5.49E-02	4.11E-04	2.38E-01	5.27E-02	9.07E-06
acisoga	9.57E-02	6.42E-02	1.37E-01	6.51E-02	6.23E-02	2.97E-01	1.60E-01	5.99E-02	7.81E-03
2-aminoheptanoate	7.92E-02	6.38E-02	2.15E-01	7.37E-02	6.17E-02	2.33E-01	7.62E-02	6.00E-02	2.05E-01
1-eicosapentaenoyl-GPE (20:5)*	9.00E-02	6.28E-02	1.53E-01	-1.01E-02	6.10E-02	8.69E-01	1.43E-01	5.86E-02	1.52E-02
N-formylanthranilic acid	-2.69E-02	6.56E-02	6.83E-01	-1.95E-02	6.35E-02	7.59E-01	-2.32E-02	6.17E-02	7.07E-01
N2,N5-diacetylornithine	1.74E-01	6.12E-02	4.83E-03	-1.18E-02	6.01E-02	8.45E-01	1.79E-01	5.74E-02	2.02E-03
1H-indole-7-acetic acid	1.70E-02	6.65E-02	7.99E-01	4.59E-02	6.44E-02	4.76E-01	-1.52E-01	6.19E-02	1.48E-02
3-methoxytyramine sulfate	1.33E-01	6.12E-02	3.01E-02	5.45E-02	5.96E-02	3.62E-01	5.00E-02	5.79E-02	3.89E-01
methionine sulfone	1.97E-02	6.40E-02	7.58E-01	4.86E-02	6.19E-02	4.33E-01	-3.34E-02	6.01E-02	5.79E-01
cyclo(ala-pro)	1.48E-02	6.46E-02	8.19E-01	-5.56E-02	6.25E-02	3.75E-01	1.98E-02	6.07E-02	7.45E-01
norbenzoylecgonine*	-4.57E-02	4.76E-02	3.37E-01	-4.25E-02	4.61E-02	3.57E-01	7.66E-04	4.48E-02	9.86E-01
1-linolenoyl-GPC (18:3)*	6.01E-02	6.39E-02	3.48E-01	2.68E-02	6.20E-02	6.65E-01	7.52E-02	6.00E-02	2.12E-01
1-eicosapentaenoyl-GPC (20:5)*	1.18E-01	6.42E-02	6.80E-02	1.87E-04	6.25E-02	9.98E-01	1.52E-01	6.00E-02	1.20E-02
1-eicosenoyl-GPC (20:1)*	2.59E-01	5.76E-02	1.02E-05	1.76E-01	5.68E-02	2.10E-03	2.22E-01	5.45E-02	5.85E-05
1-nonadecanoyl-GPC (19:0)	1.29E-01	5.98E-02	3.14E-02	1.56E-01	5.76E-02	7.19E-03	1.07E-01	5.63E-02	5.87E-02
N-acetylalliin	1.06E-02	6.52E-02	8.71E-01	-6.84E-02	6.30E-02	2.79E-01	8.64E-02	6.11E-02	1.58E-01
1-dihomo-linolenoyl-GPE (20:3n3 or 6)*	7.98E-02	6.39E-02	2.13E-01	4.43E-02	6.19E-02	4.75E-01	6.85E-04	6.02E-02	9.91E-01
1-(1-enyl-oleoyl)-GPE (P-18:1)*	-8.25E-03	6.66E-02	9.02E-01	-6.36E-02	6.44E-02	3.24E-01	4.77E-02	6.26E-02	4.47E-01
fructosyllysine	-6.43E-02	6.42E-02	3.17E-01	-7.16E-02	6.21E-02	2.50E-01	-1.24E-01	6.00E-02	3.90E-02
1-eicosenoyl-GPE (20:1)*	1.58E-01	6.27E-02	1.22E-02	1.16E-01	6.10E-02	5.88E-02	1.09E-01	5.92E-02	6.68E-02
N-methylpipecolate	7.97E-02	5.61E-02	1.57E-01	2.81E-02	5.45E-02	6.07E-01	6.30E-02	5.28E-02	2.34E-01
O-sulfo-L-tyrosine	6.05E-04	6.40E-02	9.92E-01	4.76E-03	6.19E-02	9.39E-01	1.09E-02	6.02E-02	8.57E-01
ferulic acid 4-sulfate	1.73E-01	6.31E-02	6.43E-03	1.68E-01	6.11E-02	6.24E-03	1.16E-01	5.97E-02	5.35E-02
3-(3-hydroxyphenyl)propionate sulfate	1.76E-01	6.62E-02	8.35E-03	1.74E-01	6.41E-02	7.07E-03	1.22E-01	6.26E-02	5.21E-02
11-ketoetiocholanolone glucuronide	6.45E-02	6.43E-02	3.17E-01	2.39E-02	6.24E-02	7.02E-01	-9.04E-02	6.04E-02	1.36E-01
etiocholanolone glucuronide	-1.79E-02	5.98E-02	7.66E-01	-3.23E-02	5.79E-02	5.78E-01	-3.58E-02	5.62E-02	5.25E-01
17alpha-hydroxypregnanolone glucuronide	-2.21E-02	5.64E-02	6.95E-01	-6.18E-02	5.45E-02	2.57E-01	-8.50E-02	5.28E-02	1.08E-01
N-acetyltaurine	-1.14E-02	5.88E-02	8.46E-01	4.57E-02	5.69E-02	4.22E-01	2.20E-02	5.53E-02	6.91E-01
1-docosapentaenoyl-GPC (22:5n6)*	-1.29E-01	6.18E-02	3.72E-02	-1.34E-01	5.97E-02	2.57E-02	-1.86E-01	5.74E-02	1.34E-03
1-linolenoyl-GPE (18:3)*	2.93E-02	6.42E-02	6.49E-01	2.87E-02	6.22E-02	6.45E-01	3.19E-02	6.04E-02	5.98E-01
1-oleoyl-GPG (18:1)*	9.89E-02	6.49E-02	1.29E-01	9.84E-02	6.28E-02	1.18E-01	-5.35E-02	6.12E-02	3.83E-01
1-palmitoyl-GPG (16:0)*	-1.93E-02	6.36E-02	7.61E-01	-2.22E-02	6.15E-02	7.18E-01	-2.94E-02	5.98E-02	6.23E-01
N-linoleoyltaurine*	7.98E-02	6.48E-02	2.19E-01	8.53E-02	6.27E-02	1.75E-01	5.18E-02	6.10E-02	3.97E-01
N-acetyl-S-allyl-L-cysteine	-2.92E-02	6.35E-02	6.46E-01	-7.91E-02	6.13E-02	1.98E-01	3.04E-02	5.97E-02	6.10E-01
9-hydroxystearate	-1.94E-01	5.77E-02	8.97E-04	-1.52E-01	5.62E-02	7.16E-03	-1.46E-01	5.46E-02	7.98E-03
3-methylglutarylcarnitine (2)	2.20E-02	6.06E-02	7.17E-01	1.15E-01	5.83E-02	4.97E-02	-2.67E-02	5.70E-02	6.39E-01
methyl glucopyranoside (alpha + beta)	2.51E-01	6.00E-02	3.85E-05	1.99E-01	5.87E-02	7.98E-04	1.99E-01	5.70E-02	5.70E-04
2-keto-3-deoxy-gluconate	-1.94E-02	5.94E-02	7.44E-01	1.09E-01	5.71E-02	5.73E-02	-6.72E-02	5.57E-02	2.29E-01
alpha-CEHC sulfate	1.27E-01	6.64E-02	5.76E-02	8.08E-02	6.45E-02	2.11E-01	-2.04E-02	6.28E-02	7.46E-01
alpha-CMBHC glucuronide	-4.23E-03	6.47E-02	9.48E-01	2.30E-02	6.26E-02	7.14E-01	-2.91E-02	6.08E-02	6.33E-01
sphingomyelin (d18:2/14:0, d18:1/14:1)*	1.19E-02	5.19E-02	8.19E-01	-1.23E-02	5.02E-02	8.07E-01	3.14E-02	4.88E-02	5.21E-01
sphingomyelin (d18:1/24:1, d18:2/24:0)*	-7.84E-02	6.22E-02	2.08E-01	-2.12E-01	5.90E-02	3.94E-04	1.08E-02	5.86E-02	8.55E-01
octadecenedioylcarnitine (C18:1-DC)*	-1.64E-02	6.42E-02	7.99E-01	6.63E-02	6.20E-02	2.86E-01	-1.85E-02	6.04E-02	7.60E-01
octadecanedioylcarnitine (C18-DC)*	-1.16E-01	6.36E-02	6.89E-02	2.24E-02	6.19E-02	7.18E-01	-1.25E-01	5.97E-02	3.78E-02
N,O-didesmethylvenlafaxine glucuronide	3.42E-02	7.28E-02	6.39E-01	2.53E-02	7.05E-02	7.20E-01	1.03E-01	6.82E-02	1.31E-01
5alpha-androstan-3alpha,17beta-diol monosulfate (2)	-9.81E-02	4.69E-02	3.75E-02	-6.46E-02	4.56E-02	1.58E-01	-8.40E-02	4.42E-02	5.82E-02
myristoleoylcarnitine (C14:1)*	-1.09E-01	6.35E-02	8.85E-02	-1.63E-01	6.10E-02	8.06E-03	-4.81E-02	6.00E-02	4.23E-01
N-formylphenylalanine	7.93E-02	6.52E-02	2.25E-01	3.52E-02	6.32E-02	5.79E-01	4.30E-02	6.14E-02	4.84E-01
cyclo(pro-val)	-7.80E-02	6.39E-02	2.23E-01	-2.83E-02	6.20E-02	6.49E-01	7.42E-03	6.03E-02	9.02E-01
4-hydroxychlorothalonil	-1.20E-01	6.01E-02	4.79E-02	-1.54E-01	5.79E-02	8.19E-03	-8.67E-02	5.67E-02	1.28E-01
isoeugenol sulfate	9.46E-03	6.16E-02	8.78E-01	-3.67E-03	5.96E-02	9.51E-01	7.85E-02	5.77E-02	1.75E-01
tyramine O-sulfate	-5.51E-02	6.32E-02	3.84E-01	-5.11E-02	6.11E-02	4.04E-01	1.43E-02	5.95E-02	8.10E-01
3-hydroxypyridine sulfate	9.75E-02	6.05E-02	1.08E-01	-2.31E-02	5.88E-02	6.94E-01	4.37E-02	5.70E-02	4.44E-01
4-methylguaiacol sulfate	2.00E-01	6.67E-02	2.98E-03	1.73E-01	6.47E-02	7.80E-03	9.28E-02	6.34E-02	1.45E-01
maltol sulfate	7.36E-02	6.72E-02	2.75E-01	5.14E-02	6.51E-02	4.30E-01	5.25E-02	6.33E-02	4.07E-01
phenylacetylcarnitine	6.36E-02	6.46E-02	3.25E-01	1.50E-01	6.19E-02	1.60E-02	9.44E-02	6.05E-02	1.20E-01
arabonate/xylonate	1.34E-01	5.68E-02	1.87E-02	1.13E-01	5.51E-02	4.13E-02	2.05E-02	5.39E-02	7.03E-01
methyl-4-hydroxybenzoate sulfate	-3.27E-02	5.95E-02	5.84E-01	3.55E-03	5.77E-02	9.51E-01	-2.58E-02	5.60E-02	6.45E-01
1-dihomo-linolenylglycerol (20:3)	1.64E-02	6.17E-02	7.90E-01	2.37E-02	5.97E-02	6.91E-01	-4.06E-02	5.79E-02	4.84E-01
vanillic alcohol sulfate	5.23E-02	6.48E-02	4.20E-01	1.43E-01	6.22E-02	2.24E-02	5.95E-02	6.09E-02	3.29E-01
4-vinylguaiacol sulfate	1.14E-01	6.03E-02	5.99E-02	9.08E-02	5.85E-02	1.22E-01	5.89E-02	5.70E-02	3.02E-01
vanillactate	-3.17E-02	5.94E-02	5.94E-01	5.59E-02	5.75E-02	3.31E-01	-4.35E-02	5.58E-02	4.36E-01
eugenol sulfate	6.80E-02	6.47E-02	2.94E-01	3.28E-02	6.27E-02	6.01E-01	7.56E-02	6.08E-02	2.15E-01
pregnanolone/allopregnanolone sulfate	4.09E-03	6.63E-02	9.51E-01	-4.72E-02	6.41E-02	4.62E-01	-7.11E-02	6.22E-02	2.54E-01
2-methoxyresorcinol sulfate	4.26E-02	6.67E-02	5.24E-01	-5.18E-03	6.46E-02	9.36E-01	1.12E-01	6.24E-02	7.39E-02
2-acetamidophenol sulfate	1.89E-01	6.38E-02	3.30E-03	2.54E-01	6.09E-02	4.11E-05	1.17E-01	6.05E-02	5.44E-02
p-cresol glucuronide*	3.07E-02	6.55E-02	6.40E-01	1.15E-01	6.31E-02	6.87E-02	-3.17E-02	6.16E-02	6.08E-01
acesulfame	-1.27E-02	6.64E-02	8.49E-01	1.96E-02	6.42E-02	7.60E-01	4.52E-03	6.24E-02	9.42E-01
valsartan	1.74E-02	5.99E-02	7.72E-01	-1.28E-02	5.80E-02	8.26E-01	-1.49E-02	5.63E-02	7.92E-01
6-hydroxyindole sulfate	-4.52E-02	6.54E-02	4.91E-01	2.83E-02	6.34E-02	6.56E-01	-6.81E-02	6.14E-02	2.68E-01
4-methoxyphenol sulfate	1.99E-01	6.23E-02	1.59E-03	2.73E-01	5.92E-02	6.13E-06	1.83E-01	5.86E-02	2.01E-03
2,4-dichlorophenol sulfate	9.45E-04	6.54E-02	9.88E-01	-4.80E-03	6.33E-02	9.40E-01	-2.03E-02	6.15E-02	7.42E-01
propyl 4-hydroxybenzoate sulfate	-1.27E-01	5.82E-02	2.97E-02	-4.23E-02	5.68E-02	4.58E-01	-1.05E-01	5.49E-02	5.63E-02
ethylparaben sulfate	-6.40E-02	6.24E-02	3.06E-01	-1.69E-01	5.96E-02	5.04E-03	-7.59E-02	5.86E-02	1.96E-01
umbelliferone sulfate	4.73E-02	6.31E-02	4.54E-01	5.14E-02	6.10E-02	4.01E-01	7.86E-02	5.92E-02	1.85E-01

sphingomyelin (d18:1/20:0, d16:1/22:0)*	-6.54E-02	6.27E-02	2.98E-01	-7.30E-02	6.06E-02	2.29E-01	-9.19E-02	5.88E-02	1.19E-01
sphingomyelin (d18:1/20:1, d18:2/20:0)*	-8.89E-02	5.85E-02	1.30E-01	-5.06E-02	5.68E-02	3.73E-01	-1.72E-02	5.52E-02	7.55E-01
sphingomyelin (d18:1/20:2, d18:2/20:1, d16:1/22:2)*	-1.59E-01	5.66E-02	5.20E-03	-1.70E-01	5.46E-02	2.00E-03	-1.62E-01	5.31E-02	2.50E-03
behenoyl sphingomyelin (d18:1/22:0)*	-4.10E-02	6.62E-02	5.36E-01	-9.43E-02	6.39E-02	1.41E-01	-5.88E-04	6.23E-02	9.92E-01
sphingomyelin (d18:1/22:1, d18:2/22:0, d16:1/24:1)*	-3.38E-02	5.88E-02	5.66E-01	-3.73E-02	5.69E-02	5.13E-01	-9.17E-03	5.53E-02	8.69E-01
sphingomyelin (d18:1/22:2, d18:2/22:1, d16:1/24:2)*	-1.59E-01	6.00E-02	8.60E-03	-1.93E-01	5.76E-02	8.98E-04	-1.55E-01	5.63E-02	6.41E-03
lignoceroyl sphingomyelin (d18:1/24:0)	1.02E-01	6.42E-02	1.12E-01	-1.92E-02	6.24E-02	7.59E-01	1.15E-01	6.02E-02	5.70E-02
sphingomyelin (d17:1/16:0, d18:1/15:0, d16:1/17:0)*	-1.72E-01	5.91E-02	3.96E-03	-1.04E-01	5.77E-02	7.23E-02	-1.89E-01	5.53E-02	7.19E-04
dopamine 3-O-sulfate	2.26E-01	6.45E-02	5.26E-04	1.68E-01	6.30E-02	8.14E-03	1.07E-01	6.17E-02	8.45E-02
3-hydroxyhexanoate	-4.47E-02	6.04E-02	4.59E-01	-1.23E-02	5.85E-02	8.33E-01	-3.34E-02	5.68E-02	5.57E-01
N-carbamoylalanine	1.96E-01	6.23E-02	1.83E-03	2.26E-01	5.98E-02	1.89E-04	1.28E-01	5.91E-02	3.17E-02
3beta-hydroxy-5-cholestenoate	-6.19E-02	6.05E-02	3.07E-01	-8.80E-02	5.84E-02	1.33E-01	-6.48E-02	5.68E-02	2.55E-01
1,2,3-benzenetriol sulfate (2)	1.02E-02	6.70E-02	8.79E-01	-3.36E-02	6.48E-02	6.05E-01	2.59E-02	6.30E-02	6.81E-01
3-methoxycatechol sulfate (1)	6.83E-02	6.53E-02	2.97E-01	6.09E-02	6.32E-02	3.36E-01	1.66E-01	6.07E-02	6.72E-03
3-methoxycatechol sulfate (2)	1.16E-01	6.28E-02	6.50E-02	1.49E-01	6.05E-02	1.46E-02	7.41E-02	5.92E-02	2.12E-01
N-acetylkynurenine (2)	-1.21E-02	5.92E-02	8.38E-01	-2.61E-02	5.72E-02	6.49E-01	-2.08E-02	5.56E-02	7.08E-01
C-glycosyltryptophan	-5.98E-02	5.94E-02	3.15E-01	1.63E-02	5.76E-02	7.78E-01	-1.24E-01	5.55E-02	2.56E-02
tramadol	-3.67E-02	7.19E-02	6.10E-01	-8.88E-02	6.94E-02	2.02E-01	-3.63E-02	6.76E-02	5.91E-01
O-desmethyltramadol	-3.67E-02	7.19E-02	6.10E-01	-8.88E-02	6.94E-02	2.02E-01	-3.63E-02	6.76E-02	5.91E-01
O-desmethyltramadol glucuronide	-3.67E-02	7.19E-02	6.10E-01	-8.88E-02	6.94E-02	2.02E-01	-3.63E-02	6.76E-02	5.91E-01
arabitol/xylitol	8.54E-02	5.84E-02	1.45E-01	6.30E-02	5.67E-02	2.67E-01	4.17E-02	5.51E-02	4.50E-01
N-acetylglucosamine/N-acetylgalactosamine	-6.61E-02	6.25E-02	2.92E-01	-3.69E-02	6.06E-02	5.43E-01	-9.41E-02	5.87E-02	1.10E-01
citraconate/glutaconate	7.83E-02	6.14E-02	2.03E-01	-4.09E-02	5.95E-02	4.93E-01	2.14E-02	5.79E-02	7.12E-01
adipoylcarnitine (C6-DC)	-2.21E-01	6.56E-02	8.73E-04	-3.50E-02	6.48E-02	5.90E-01	-1.55E-01	6.23E-02	1.33E-02
nonanoylcarnitine (C9)	-1.84E-01	6.48E-02	4.91E-03	-1.75E-01	6.27E-02	5.62E-03	-9.65E-02	6.15E-02	1.18E-01
glycochenodeoxycholate 3-sulfate	-2.95E-02	6.11E-02	6.30E-01	-4.31E-02	5.91E-02	4.67E-01	-5.06E-02	5.74E-02	3.79E-01
glycodeoxycholate 3-sulfate	3.95E-02	6.33E-02	5.34E-01	4.23E-03	6.13E-02	9.45E-01	-6.73E-02	5.95E-02	2.59E-01
taurodeoxycholic acid 3-sulfate	-6.73E-03	6.60E-02	9.19E-01	-6.15E-02	6.37E-02	3.36E-01	-1.14E-01	6.16E-02	6.51E-02
trans-3,4-methyleneheptanoate	-9.32E-02	6.62E-02	1.60E-01	-4.85E-02	6.43E-02	4.51E-01	-5.29E-02	6.24E-02	3.98E-01
phenol glucuronide	-7.20E-02	6.55E-02	2.73E-01	-2.40E-02	6.36E-02	7.06E-01	-8.52E-03	6.18E-02	8.90E-01
linoleoyl ethanolamide	-3.30E-02	6.22E-02	5.97E-01	-1.72E-02	6.03E-02	7.75E-01	1.67E-03	5.85E-02	9.77E-01
1,2-dilinoleoyl-GPC (18:2/18:2)	8.28E-02	6.15E-02	1.79E-01	1.11E-01	5.94E-02	6.16E-02	9.22E-02	5.78E-02	1.12E-01
1-stearoyl-2-oleoyl-GPC (18:0/18:1)	4.35E-02	6.61E-02	5.12E-01	-3.14E-02	6.40E-02	6.25E-01	7.48E-02	6.21E-02	2.30E-01
1-palmitoyl-2-arachidonoyl-GPC (16:0/20:4n6)	-1.54E-01	6.41E-02	1.67E-02	-3.05E-01	6.00E-02	6.93E-07	-1.02E-01	6.06E-02	9.35E-02
1-palmitoyl-2-docosahexaenoyl-GPC (16:0/22:6)	5.67E-02	6.31E-02	3.70E-01	-1.23E-01	6.07E-02	4.38E-02	9.66E-02	5.91E-02	1.04E-01
1-stearoyl-2-docosahexaenoyl-GPC (18:0/22:6)	9.06E-02	6.19E-02	1.44E-01	-1.44E-02	6.01E-02	8.12E-01	1.39E-01	5.78E-02	1.66E-02
1-(1-enyl-stearoyl)-2-oleoyl-GPC (P-18:0/18:1)	1.73E-02	5.88E-02	7.69E-01	-5.48E-02	5.68E-02	3.36E-01	4.73E-02	5.52E-02	3.92E-01
1-(1-enyl-stearoyl)-2-arachidonoyl-GPC (P-18:0/20:4)	-1.25E-01	6.16E-02	4.32E-02	-1.38E-01	5.95E-02	2.10E-02	-8.36E-02	5.81E-02	1.52E-01
1-(1-enyl-stearoyl)-2-oleoyl-GPE (P-18:0/18:1)	3.35E-02	6.49E-02	6.06E-01	-2.39E-02	6.28E-02	7.04E-01	7.10E-02	6.09E-02	2.45E-01
sphingomyelin (d18:1/17:0, d17:1/18:0, d19:1/16:0)	-2.03E-01	6.06E-02	9.32E-04	-1.76E-01	5.89E-02	3.14E-03	-1.73E-01	5.72E-02	2.66E-03
1-palmitoyl-2-stearoyl-GPC (16:0/18:0)	-1.85E-02	6.44E-02	7.74E-01	-9.58E-02	6.21E-02	1.24E-01	6.29E-02	6.04E-02	2.99E-01
2-hydroxybutyrate/2-hydroxyisobutyrate	-2.21E-02	6.28E-02	7.26E-01	-1.38E-01	6.03E-02	2.27E-02	2.99E-02	5.91E-02	6.12E-01
oleate/vaccenate (18:1)	-1.10E-01	6.01E-02	6.73E-02	-1.23E-01	5.80E-02	3.48E-02	-9.21E-02	5.65E-02	1.05E-01
isoleucylleucine/leucylisoleucine	5.41E-02	6.45E-02	4.02E-01	1.26E-01	6.20E-02	4.40E-02	9.89E-02	6.04E-02	1.03E-01
aripiprazole	-7.69E-02	7.11E-02	2.80E-01	-2.98E-02	6.89E-02	6.66E-01	-2.28E-02	6.70E-02	7.34E-01
rivaroxaban	7.74E-02	7.25E-02	2.87E-01	8.29E-03	7.03E-02	9.06E-01	5.13E-02	6.82E-02	4.52E-01
leucylphenylalanine/isoleucylphenylalanine	1.10E-01	6.75E-02	1.04E-01	9.18E-02	6.54E-02	1.62E-01	7.01E-02	6.36E-02	2.71E-01
1-palmitoleoylglycerol (16:1)*	4.02E-02	6.37E-02	5.28E-01	-5.67E-02	6.16E-02	3.58E-01	-6.35E-02	5.98E-02	2.89E-01
palmitoyl dihydrosphingomyelin (d18:0/16:0)*	6.25E-02	6.25E-02	3.18E-01	-1.14E-01	6.02E-02	5.83E-02	1.05E-01	5.85E-02	7.45E-02
tricosanoyl sphingomyelin (d18:1/23:0)*	2.54E-02	6.10E-02	6.77E-01	-8.11E-02	5.88E-02	1.69E-01	1.68E-02	5.73E-02	7.70E-01
sphingomyelin (d18:2/23:0, d18:1/23:1, d17:1/24:1)*	-4.11E-02	5.50E-02	4.56E-01	-8.71E-02	5.31E-02	1.02E-01	-9.78E-04	5.18E-02	9.85E-01
sphingomyelin (d18:2/24:1, d18:1/24:2)*	-6.14E-02	5.76E-02	2.88E-01	-1.76E-01	5.49E-02	1.54E-03	1.81E-03	5.43E-02	9.73E-01
diclofenac	5.22E-02	7.22E-02	4.71E-01	-2.15E-04	7.00E-02	9.98E-01	-5.14E-02	6.79E-02	4.50E-01
1-stearoyl-2-linoleoyl-GPE (18:0/18:2)*	1.09E-01	6.54E-02	9.60E-02	6.47E-02	6.35E-02	3.09E-01	1.17E-01	6.14E-02	5.68E-02
1-stearoyl-2-arachidonoyl-GPE (18:0/20:4)	3.03E-02	6.64E-02	6.48E-01	-3.43E-02	6.42E-02	5.94E-01	6.19E-02	6.23E-02	3.21E-01
1-stearoyl-2-linoleoyl-GPC (18:0/18:2)*	2.33E-02	6.32E-02	7.13E-01	2.23E-02	6.12E-02	7.16E-01	8.98E-02	5.92E-02	1.31E-01
1-palmitoyl-2-palmitoleoyl-GPC (16:0/16:1)*	-8.80E-02	6.28E-02	1.62E-01	-2.06E-01	5.97E-02	6.46E-04	-9.91E-02	5.90E-02	9.38E-02
1-palmitoyl-2-eicosapentaenoyl-GPC (16:0/20:5)*	6.27E-02	6.52E-02	3.38E-01	-9.79E-02	6.30E-02	1.21E-01	1.09E-01	6.11E-02	7.61E-02
1-palmitoyl-2-arachidonoyl-GPE (16:0/20:4)*	3.38E-02	6.61E-02	6.10E-01	-9.04E-02	6.37E-02	1.57E-01	3.64E-02	6.21E-02	5.59E-01
1-palmitoyl-2-docosahexaenoyl-GPE (16:0/22:6)*	8.66E-02	6.52E-02	1.85E-01	-7.21E-02	6.32E-02	2.55E-01	8.86E-02	6.13E-02	1.50E-01
1-stearoyl-2-docosahexaenoyl-GPE (18:0/22:6)*	6.09E-02	6.46E-02	3.47E-01	-4.28E-02	6.26E-02	4.95E-01	5.17E-02	6.07E-02	3.95E-01
1-palmitoyl-2-arachidonoyl-GPI (16:0/20:4)*	-2.14E-02	6.56E-02	7.45E-01	-9.36E-02	6.32E-02	1.40E-01	-2.98E-02	6.16E-02	6.29E-01
1-stearoyl-2-linoleoyl-GPI (18:0/18:2)	1.17E-01	6.19E-02	5.89E-02	1.41E-02	6.03E-02	8.16E-01	1.50E-01	5.79E-02	1.01E-02
1-palmitoyl-2-palmitoleoyl-GPE (16:0/16:1)*	2.41E-03	6.28E-02	9.69E-01	-8.00E-02	6.06E-02	1.88E-01	-4.50E-02	5.90E-02	4.46E-01
gamma-tocopherol/beta-tocopherol	-1.38E-01	6.59E-02	3.78E-02	-1.34E-01	6.38E-02	3.66E-02	-3.15E-02	6.24E-02	6.14E-01
1-(1-enyl-stearoyl)-2-arachidonoyl-GPE (P-18:0/20:4)*	-1.52E-01	5.99E-02	1.18E-02	-1.95E-01	5.74E-02	7.64E-04	-5.48E-02	5.68E-02	3.36E-01
1-(1-enyl-palmitoyl)-2-docosahexaenoyl-GPE (P-16:0/22:6)*	5.44E-02	6.06E-02	3.70E-01	-1.02E-01	5.84E-02	8.14E-02	1.28E-01	5.65E-02	2.37E-02
1-(1-enyl-palmitoyl)-2-arachidonoyl-GPE (P-16:0/20:4)*	-3.11E-02	6.26E-02	6.19E-01	-1.11E-01	6.02E-02	6.60E-02	-6.51E-03	5.89E-02	9.12E-01
1-(1-enyl-oleoyl)-2-linoleoyl-GPE (P-18:1/18:2)*	4.31E-02	6.45E-02	5.04E-01	3.01E-02	6.24E-02	6.30E-01	1.06E-01	6.03E-02	8.10E-02
1-(1-enyl-stearoyl)-2-docosahexaenoyl-GPE (P-18:0/22:6)*	3.98E-02	5.99E-02	5.06E-01	-6.57E-02	5.79E-02	2.57E-01	1.40E-01	5.57E-02	1.24E-02
1-(1-enyl-palmitoyl)-2-oleoyl-GPE (P-16:0/18:1)*	7.68E-02	6.49E-02	2.38E-01	-1.31E-02	6.30E-02	8.35E-01	1.06E-01	6.09E-02	8.26E-02
1-(1-enyl-palmitoyl)-2-dihomo-linolenoyl-GPC (P-16:0/20:3)*	2.91E-02	6.62E-02	6.61E-01	-5.61E-02	6.40E-02	3.81E-01	-4.52E-02	6.22E-02	4.67E-01
1-(1-enyl-palmitoyl)-2-oleoyl-GPC (P-16:0/18:1)*	1.31E-02	5.78E-02	8.21E-01	-7.04E-02	5.58E-02	2.08E-01	5.36E-02	5.43E-02	3.24E-01
1-(1-enyl-palmitoyl)-2-docosahexaenoyl-GPC (P-16:0/22:6)*	8.47E-02	6.06E-02	1.63E-01	-8.07E-02	5.87E-02	1.70E-01	1.51E-01	5.65E-02	7.99E-03
1-(1-enyl-palmitoyl)-2-linoleoyl-GPC (P-16:0/18:2)*	-2.11E-02	6.12E-02	7.31E-01	-9.49E-03	5.93E-02	8.73E-01	4.75E-02	5.75E-02	4.10E-01
1-(1-enyl-palmitoyl)-2-arachidonoyl-GPC (P-16:0/20:4)*	-1.23E-01	6.02E-02	4.19E-02	-2.07E-01	5.73E-02	3.61E-04	-7.65E-02	5.68E-02	1.79E-01
1-(1-enyl-stearoyl)-2-docosahexaenoyl-GPC (P-18:0/22:6)*	5.87E-02	5.83E-02	3.15E-01	-3.84E-02	5.65E-02	4.97E-01	1.38E-01	5.43E-02	1.18E-02
1-stearyl-2-arachidonoyl-GPC (O-18:0/20:4)*	-2.30E-01	6.32E-02	3.19E-04	-1.04E-01	6.23E-02	9.64E-02	-2.40E-01	5.91E-02	6.37E-05
1-palmityl-2-oleoyl-GPC (O-16:0/18:1)*	-5.32E-02	6.26E-02	3.96E-01	-7.02E-02	6.05E-02	2.47E-01	-3.63E-02	5.88E-02	5.37E-01
1-palmityl-2-arachidonoyl-GPC (O-16:0/20:4)*	-1.78E-01	6.14E-02	4.02E-03	-2.16E-01	5.89E-02	2.93E-04	-1.26E-01	5.81E-02	3.06E-02
sphingomyelin (d18:1/21:0, d17:1/22:0, d16:1/23:0)*	-2.32E-02	5.88E-02	6.93E-01	-2.18E-02	5.69E-02	7.01E-01	-7.04E-02	5.51E-02	2.02E-01
behenoyl dihydrosphingomyelin (d18:0/22:0)*	-7.42E-03	6.57E-02	9.10E-01	-1.68E-01	6.27E-02	7.77E-03	2.72E-02	6.17E-02	6.59E-01

sphingomyelin (d18:0/18:0, d19:0/17:0)*	-2.24E-01	6.28E-02	4.23E-04	-3.81E-01	5.78E-02	2.24E-10	-1.49E-01	5.97E-02	1.32E-02
N-palmitoyl-sphinganine (d18:0/16:0)	-2.25E-03	6.46E-02	9.72E-01	-1.37E-01	6.20E-02	2.76E-02	3.85E-02	6.07E-02	5.26E-01
lactosyl-N-palmitoyl-sphingosine (d18:1/16:0)	-1.02E-01	6.21E-02	1.02E-01	-2.99E-02	6.04E-02	6.21E-01	-6.68E-02	5.85E-02	2.55E-01
cetirizine	8.17E-02	6.40E-02	2.03E-01	5.95E-02	6.20E-02	3.39E-01	1.62E-02	6.04E-02	7.88E-01
1-pentadecanoyl-2-linoleoyl-GPC (15:0/18:2)*	-8.26E-02	5.66E-02	1.46E-01	4.47E-02	5.49E-02	4.17E-01	-1.28E-01	5.29E-02	1.62E-02
1-margaroyl-2-oleoyl-GPC (17:0/18:1)*	6.94E-02	6.12E-02	2.57E-01	6.91E-02	5.92E-02	2.44E-01	5.45E-02	5.75E-02	3.44E-01
1-margaroyl-2-linoleoyl-GPC (17:0/18:2)*	8.55E-02	5.91E-02	1.50E-01	1.93E-01	5.63E-02	6.83E-04	9.11E-02	5.55E-02	1.02E-01
myristoyl dihydrosphingomyelin (d18:0/14:0)*	-4.39E-02	6.02E-02	4.66E-01	-1.02E-01	5.80E-02	7.93E-02	-6.72E-02	5.65E-02	2.35E-01
5-hydroxyindole sulfate	-1.28E-02	6.53E-02	8.45E-01	3.04E-02	6.32E-02	6.31E-01	-4.37E-02	6.13E-02	4.77E-01
7-hydroxyindole sulfate	5.67E-02	6.25E-02	3.65E-01	5.94E-02	6.05E-02	3.27E-01	6.98E-02	5.87E-02	2.35E-01
phenylacetylglutamate	-4.00E-02	6.41E-02	5.33E-01	5.44E-02	6.20E-02	3.81E-01	-2.48E-02	6.03E-02	6.81E-01
1-stearoyl-2-dihomo-linolenoyl-GPC (18:0/20:3n3 or 6)*	8.75E-02	6.21E-02	1.60E-01	3.27E-02	6.02E-02	5.88E-01	-1.07E-02	5.85E-02	8.55E-01
palmitoyl-linoleoyl-glycerol (16:0/18:2) [1]*	4.67E-02	6.19E-02	4.51E-01	2.16E-02	6.00E-02	7.19E-01	-1.45E-02	5.83E-02	8.03E-01
palmitoyl-linoleoyl-glycerol (16:0/18:2) [2]*	-1.96E-04	6.19E-02	9.97E-01	-4.10E-02	5.99E-02	4.94E-01	-5.55E-02	5.81E-02	3.40E-01
1-palmitoyl-2-oleoyl-GPI (16:0/18:1)*	3.96E-02	6.61E-02	5.50E-01	-6.63E-02	6.39E-02	3.01E-01	4.35E-02	6.22E-02	4.85E-01
1-stearoyl-2-docosahexaenoyl-GPI (18:0/22:6)*	5.26E-02	6.64E-02	4.29E-01	-7.93E-02	6.41E-02	2.18E-01	9.84E-02	6.22E-02	1.15E-01
1-(1-enyl-palmitoyl)-2-linoleoyl-GPE (P-16:0/18:2)*	-2.18E-02	6.53E-02	7.39E-01	1.67E-02	6.32E-02	7.92E-01	6.19E-03	6.14E-02	9.20E-01
1-oleoyl-2-linoleoyl-GPE (18:1/18:2)*	2.22E-01	6.23E-02	4.36E-04	2.07E-01	6.04E-02	7.00E-04	1.79E-01	5.90E-02	2.66E-03
1-pentadecanoyl-2-arachidonoyl-GPC (15:0/20:4)*	-1.37E-01	6.24E-02	2.87E-02	-4.52E-02	6.08E-02	4.58E-01	-1.81E-01	5.81E-02	2.10E-03
1-pentadecanoyl-2-docosahexaenoyl-GPC (15:0/22:6)*	5.45E-02	6.00E-02	3.65E-01	5.67E-02	5.81E-02	3.30E-01	5.08E-02	5.64E-02	3.68E-01
1-margaroyl-2-arachidonoyl-GPC (17:0/20:4)*	-1.10E-01	6.43E-02	8.88E-02	-2.86E-02	6.25E-02	6.47E-01	-1.16E-01	6.04E-02	5.51E-02
1-arachidoyl-2-arachidonoyl-GPC (20:0/20:4)*	1.29E-01	6.51E-02	4.93E-02	5.64E-02	6.34E-02	3.75E-01	1.18E-01	6.13E-02	5.55E-02
1-oleoyl-2-docosahexaenoyl-GPC (18:1/22:6)*	2.02E-01	5.77E-02	5.54E-04	1.34E-01	5.65E-02	1.89E-02	1.91E-01	5.43E-02	5.20E-04
1-linoleoyl-2-arachidonoyl-GPC (18:2/20:4n6)*	6.38E-02	6.20E-02	3.04E-01	-2.11E-02	6.01E-02	7.26E-01	8.55E-02	5.82E-02	1.43E-01
1-linoleoyl-2-docosahexaenoyl-GPC (18:2/22:6)*	1.56E-01	5.73E-02	6.84E-03	-2.24E-03	5.62E-02	9.68E-01	1.84E-01	5.34E-02	6.41E-04
1-palmityl-2-linoleoyl-GPC (O-16:0/18:2)*	-7.09E-02	6.37E-02	2.67E-01	-9.31E-02	6.16E-02	1.32E-01	-3.61E-02	6.00E-02	5.48E-01
1-(1-enyl-stearoyl)-2-linoleoyl-GPC (P-18:0/18:2)*	-2.65E-02	6.05E-02	6.61E-01	-4.63E-02	5.85E-02	4.29E-01	4.29E-02	5.68E-02	4.51E-01
1-myristoyl-2-linoleoyl-GPC (14:0/18:2)*	-2.95E-02	6.35E-02	6.42E-01	-5.80E-02	6.14E-02	3.46E-01	-5.05E-02	5.97E-02	3.98E-01
1-myristoyl-2-arachidonoyl-GPC (14:0/20:4)*	1.44E-02	6.44E-02	8.23E-01	-1.13E-01	6.19E-02	6.94E-02	-8.51E-03	6.05E-02	8.88E-01
1-myristoyl-2-docosahexaenoyl-GPC (14:0/22:6)*	1.43E-01	6.23E-02	2.20E-02	-1.37E-03	6.08E-02	9.82E-01	1.61E-01	5.83E-02	6.16E-03
1-stearoyl-2-docosapentaenoyl-GPC (18:0/22:5n3)*	3.43E-02	6.57E-02	6.02E-01	7.83E-02	6.34E-02	2.18E-01	2.67E-02	6.18E-02	6.65E-01
1-stearoyl-2-docosapentaenoyl-GPC (18:0/22:5n6)*	-1.56E-01	5.99E-02	9.76E-03	-1.36E-01	5.81E-02	2.02E-02	-2.34E-01	5.52E-02	3.17E-05
1-palmitoyl-2-adrenoyl-GPC (16:0/22:4)*	-9.54E-02	6.32E-02	1.32E-01	-1.29E-01	6.09E-02	3.53E-02	-1.31E-01	5.91E-02	2.78E-02
1-stearoyl-2-adrenoyl-GPC (18:0/22:4)*	-1.26E-01	5.81E-02	3.11E-02	-1.34E-01	5.61E-02	1.74E-02	-1.26E-01	5.45E-02	2.12E-02
1-stearyl-GPC (O-18:0)*	3.17E-03	6.23E-02	9.59E-01	2.20E-02	6.03E-02	7.15E-01	-1.79E-02	5.86E-02	7.60E-01
1-myristoyl-2-palmitoleoyl-GPC (14:0/16:1)*	-3.57E-02	6.36E-02	5.75E-01	-7.29E-02	6.14E-02	2.36E-01	-7.60E-02	5.96E-02	2.04E-01
1-stearoyl-2-meadoyl-GPC (18:0/20:3n9)*	-2.92E-02	6.47E-02	6.52E-01	-8.53E-02	6.24E-02	1.73E-01	-4.01E-02	6.08E-02	5.10E-01
1-(1-enyl-palmitoyl)-2-palmitoleoyl-GPC (P-16:0/16:1)*	-4.75E-02	6.32E-02	4.53E-01	-1.88E-01	6.02E-02	1.93E-03	-2.49E-03	5.95E-02	9.67E-01
1-(1-enyl-palmitoyl)-2-myristoyl-GPC (P-16:0/14:0)*	-1.83E-01	6.43E-02	4.83E-03	-1.42E-01	6.26E-02	2.41E-02	-1.13E-01	6.09E-02	6.38E-02
1-(1-enyl-palmitoyl)-2-palmitoyl-GPC (P-16:0/16:0)*	-1.36E-01	6.29E-02	3.17E-02	-2.02E-01	6.02E-02	9.10E-04	-5.19E-02	5.95E-02	3.84E-01
phosphatidylcholine (16:0/22:5n3, 18:1/20:4)*	2.10E-02	6.30E-02	7.39E-01	3.92E-02	6.10E-02	5.20E-01	8.12E-02	5.91E-02	1.70E-01
phosphatidylcholine (18:0/20:5, 16:0/22:5n6)*	6.75E-02	6.73E-02	3.17E-01	-5.30E-02	6.51E-02	4.17E-01	6.66E-02	6.32E-02	2.93E-01
1-palmitoyl-2-docosahexaenoyl-GPI (16:0/22:6)*	3.12E-02	6.71E-02	6.42E-01	-3.86E-02	6.49E-02	5.52E-01	6.92E-02	6.29E-02	2.72E-01
1-stearoyl-2-oleoyl-GPI (18:0/18:1)*	2.05E-01	6.44E-02	1.62E-03	1.25E-01	6.30E-02	4.85E-02	1.78E-01	6.07E-02	3.58E-03
1-stearoyl-2-dihomo-linolenoyl-GPI (18:0/20:3n3 or 6)*	1.39E-01	6.25E-02	2.65E-02	4.48E-02	6.10E-02	4.63E-01	7.14E-02	5.91E-02	2.28E-01
1-stearyl-GPE (O-18:0)*	4.02E-02	6.36E-02	5.28E-01	3.94E-02	6.16E-02	5.23E-01	7.64E-02	5.97E-02	2.02E-01
1,2-dipalmitoyl-GPE (16:0/16:0)*	6.50E-02	6.43E-02	3.12E-01	-3.16E-02	6.23E-02	6.12E-01	-2.89E-02	6.05E-02	6.33E-01
1-palmitoyl-2-stearoyl-GPE (16:0/18:0)*	3.27E-02	6.41E-02	6.11E-01	3.73E-02	6.20E-02	5.48E-01	5.81E-02	6.02E-02	3.36E-01
1-palmitoyl-2-eicosapentaenoyl-GPE (16:0/20:5)*	9.08E-02	6.33E-02	1.52E-01	-4.04E-05	6.15E-02	9.99E-01	9.50E-02	5.94E-02	1.11E-01
1-stearoyl-2-dihomo-linolenoyl-GPE (18:0/20:3n3 or 6)*	1.76E-01	6.46E-02	6.77E-03	1.18E-01	6.29E-02	6.08E-02	8.87E-02	6.13E-02	1.49E-01
1,2-dilinoleoyl-GPE (18:2/18:2)*	5.46E-02	6.40E-02	3.94E-01	7.43E-02	6.18E-02	2.31E-01	9.20E-03	6.02E-02	8.79E-01
1-oleoyl-2-arachidonoyl-GPE (18:1/20:4)*	1.24E-01	6.67E-02	6.51E-02	9.97E-02	6.47E-02	1.25E-01	1.41E-01	6.26E-02	2.51E-02
1-(1-enyl-stearoyl)-2-linoleoyl-GPE (P-18:0/18:2)*	-2.21E-02	6.41E-02	7.31E-01	-7.85E-03	6.21E-02	8.99E-01	4.61E-02	6.02E-02	4.44E-01
1-linoleoyl-GPG (18:2)*	-1.72E-02	6.48E-02	7.91E-01	-1.26E-03	6.27E-02	9.84E-01	-5.93E-02	6.08E-02	3.30E-01
thioproline	-1.57E-01	6.14E-02	1.11E-02	-1.56E-01	5.94E-02	8.85E-03	-7.97E-02	5.82E-02	1.72E-01
palmitoylcholine	1.39E-01	6.35E-02	3.01E-02	1.29E-01	6.15E-02	3.63E-02	1.40E-01	5.97E-02	1.94E-02
trans-3,4-methyleneheptanoylcarnitine	-1.09E-01	6.37E-02	8.69E-02	-2.69E-02	6.20E-02	6.65E-01	-3.77E-02	6.02E-02	5.31E-01
glycocholate glucuronide (1)	-1.38E-02	6.27E-02	8.26E-01	-2.74E-03	6.07E-02	9.64E-01	-9.67E-03	5.89E-02	8.70E-01
glycochenodeoxycholate glucuronide (1)	-6.45E-02	5.99E-02	2.83E-01	-9.18E-02	5.79E-02	1.14E-01	-8.10E-02	5.63E-02	1.51E-01
(S)-3-hydroxybutyrylcarnitine	-9.40E-02	6.13E-02	1.26E-01	-1.88E-02	5.96E-02	7.53E-01	-4.78E-02	5.78E-02	4.09E-01
glycosyl-N-palmitoyl-sphingosine (d18:1/16:0)	-1.79E-01	6.46E-02	5.94E-03	-1.80E-01	6.25E-02	4.29E-03	-9.70E-02	6.13E-02	1.15E-01
catechol glucuronide	2.36E-01	6.22E-02	1.88E-04	1.88E-01	6.07E-02	2.11E-03	1.41E-01	5.94E-02	1.83E-02
ascorbic acid 2-sulfate	2.24E-01	6.03E-02	2.46E-04	2.49E-01	5.79E-02	2.35E-05	9.86E-02	5.77E-02	8.88E-02
oleoylcholine	1.83E-01	6.28E-02	3.82E-03	1.83E-01	6.07E-02	2.85E-03	1.69E-01	5.91E-02	4.56E-03
arachidonoylcholine	9.99E-02	6.36E-02	1.17E-01	9.60E-02	6.16E-02	1.20E-01	1.22E-01	5.96E-02	4.12E-02
docosahexaenoylcholine	1.84E-01	6.09E-02	2.81E-03	1.19E-01	5.95E-02	4.56E-02	2.09E-01	5.69E-02	2.89E-04
palmitoloelycholine	8.36E-02	6.35E-02	1.89E-01	3.28E-02	6.16E-02	5.95E-01	5.28E-02	5.98E-02	3.78E-01
dihomo-linolenoyl-choline	1.99E-01	6.37E-02	2.00E-03	1.91E-01	6.17E-02	2.20E-03	1.23E-01	6.05E-02	4.27E-02
caffeic acid sulfate	1.55E-01	6.42E-02	1.67E-02	1.04E-01	6.25E-02	9.76E-02	1.15E-01	6.06E-02	5.91E-02
1-linoleoyl-2-linolenoyl-GPC (18:2/18:3)*	8.80E-02	6.12E-02	1.52E-01	8.10E-02	5.92E-02	1.72E-01	1.40E-01	5.71E-02	1.47E-02
1-palmitoleoyl-2-linolenoyl-GPC (16:1/18:3)*	-2.15E-02	6.25E-02	7.31E-01	-2.15E-02	6.05E-02	7.23E-01	1.83E-02	5.87E-02	7.55E-01
phosphatidylcholine (14:0/14:0, 16:0/12:0)	-4.61E-02	6.56E-02	4.83E-01	-5.60E-02	6.34E-02	3.78E-01	-8.20E-02	6.15E-02	1.84E-01
phosphatidylcholine (15:0/18:1, 17:0/16:1, 16:0/17:1)*	-8.79E-02	5.95E-02	1.41E-01	-6.78E-02	5.77E-02	2.41E-01	-1.25E-01	5.57E-02	2.60E-02
1-oleoyl-2-dihomo-linoleoyl-GPC (18:1/20:2)*	1.67E-01	6.37E-02	9.24E-03	1.38E-01	6.18E-02	2.61E-02	8.61E-02	6.04E-02	1.55E-01
1-oleoyl-2-docosapentaenoyl-GPC (18:1/22:5n3)*	1.14E-01	6.30E-02	7.06E-02	1.36E-01	6.08E-02	2.59E-02	7.82E-02	5.94E-02	1.89E-01
phosphatidylcholine (18:0/20:2, 20:0/18:2)*	2.88E-01	6.44E-02	1.14E-05	1.47E-01	6.39E-02	2.26E-02	2.31E-01	6.11E-02	1.90E-04
1-(1-enyl-oleoyl)-2-docosahexaenoyl-GPE (P-18:1/22:6)*	1.48E-01	5.98E-02	1.39E-02	-8.75E-03	5.85E-02	8.81E-01	2.37E-01	5.50E-02	2.23E-05
1-(1-enyl-stearoyl)-2-dihomo-linolenoyl-GPE (P-18:0/20:3)*	-4.08E-02	6.53E-02	5.33E-01	-5.99E-02	6.32E-02	3.44E-01	-8.10E-02	6.13E-02	1.87E-01
lisinopril	-4.50E-02	5.88E-02	4.45E-01	-9.48E-04	5.69E-02	9.87E-01	6.92E-02	5.52E-02	2.10E-01
lamotrigine	-6.09E-03	7.25E-02	9.33E-01	-9.60E-02	7.00E-02	1.71E-01	-9.11E-03	6.82E-02	8.94E-01
hexadecatrienoate (16:3n3)	1.75E-01	6.27E-02	5.59E-03	1.38E-01	6.10E-02	2.40E-02	1.32E-01	5.92E-02	2.69E-02
hexadecadienoate (16:2n6)	-1.56E-01	5.97E-02	9.32E-03	-1.32E-01	5.80E-02	2.39E-02	-9.43E-02	5.65E-02	9.64E-02

Supplementary Table 4: Parameter estimates for metabolome-wide association studies for diet-metabolite associations for each of: HEI-15, DASH and AMED diet, controlling for BMI

1-myristoyl-2-eicosapentaenoyl-GPC (14:0/20:5)*	1.14E-01	6.30E-02	7.05E-02	-9.42E-03	6.14E-02	8.78E-01	1.30E-01	5.91E-02	2.83E-02
palmitoleoylcarnitine (C16:1)*	-1.49E-01	6.06E-02	1.47E-02	-2.11E-01	5.79E-02	3.13E-04	-1.06E-01	5.72E-02	6.48E-02
4-acetamidobenzoate	1.40E-01	6.28E-02	2.66E-02	1.73E-01	6.04E-02	4.52E-03	1.44E-01	5.89E-02	1.52E-02
4-hydroxyphenylacetylglutamine	3.61E-02	6.30E-02	5.67E-01	7.16E-02	6.09E-02	2.41E-01	7.54E-02	5.91E-02	2.03E-01
4-aminophenol sulfate (2)	1.61E-01	7.25E-02	2.73E-02	1.47E-01	7.03E-02	3.76E-02	7.41E-02	6.86E-02	2.81E-01
2'-O-methyluridine	-1.05E-01	6.32E-02	9.64E-02	-6.53E-03	6.15E-02	9.16E-01	-7.97E-02	5.95E-02	1.81E-01
gamma-glutamyl-alpha-lysine	-3.35E-02	6.38E-02	6.00E-01	-2.40E-03	6.18E-02	9.69E-01	-7.06E-02	5.99E-02	2.40E-01
palmitoyl-oleoyl-glycerol (16:0/18:1) [1]*	1.72E-02	6.24E-02	7.83E-01	-5.23E-02	6.04E-02	3.87E-01	-5.42E-02	5.86E-02	3.56E-01
palmitoyl-oleoyl-glycerol (16:0/18:1) [2]*	5.23E-03	6.25E-02	9.33E-01	-5.39E-02	6.05E-02	3.73E-01	-5.01E-02	5.87E-02	3.94E-01
oleoyl-oleoyl-glycerol (18:1/18:1) [1]*	1.32E-01	6.40E-02	4.00E-02	1.02E-01	6.21E-02	1.03E-01	7.69E-02	6.04E-02	2.04E-01
oleoyl-oleoyl-glycerol (18:1/18:1) [2]*	1.55E-01	6.40E-02	1.64E-02	9.26E-02	6.23E-02	1.39E-01	8.35E-02	6.06E-02	1.70E-01
linoleoyl-arachidonoyl-glycerol (18:2/20:4) [1]*	1.02E-02	6.08E-02	8.67E-01	-1.40E-02	5.89E-02	8.12E-01	2.24E-02	5.72E-02	6.95E-01
linoleoyl-arachidonoyl-glycerol (18:2/20:4) [2]*	-4.94E-02	6.41E-02	4.41E-01	-9.00E-02	6.19E-02	1.47E-01	-1.46E-02	6.03E-02	8.08E-01
palmitoyl-arachidonoyl-glycerol (16:0/20:4) [1]*	-6.49E-02	6.08E-02	2.87E-01	-1.20E-01	5.86E-02	4.07E-02	-1.38E-01	5.67E-02	1.56E-02
palmitoyl-arachidonoyl-glycerol (16:0/20:4) [2]*	-7.07E-02	5.99E-02	2.39E-01	-1.43E-01	5.75E-02	1.34E-02	-6.24E-02	5.63E-02	2.69E-01
linoleoyl-linolenoyl-glycerol (18:2/18:3) [1]*	7.93E-02	6.49E-02	2.23E-01	9.96E-02	6.27E-02	1.13E-01	4.21E-02	6.12E-02	4.91E-01
linoleoyl-linolenoyl-glycerol (18:2/18:3) [2]*	9.76E-02	6.27E-02	1.21E-01	8.64E-02	6.08E-02	1.56E-01	1.14E-01	5.89E-02	5.44E-02
linoleoyl-docosahexaenoyl-glycerol (18:2/22:6) [2]*	1.36E-01	6.56E-02	3.98E-02	-5.04E-03	6.40E-02	9.37E-01	8.78E-02	6.20E-02	1.58E-01
palmitoleoyl-linoleoyl-glycerol (16:1/18:2) [1]*	-6.24E-02	6.39E-02	3.30E-01	-9.49E-02	6.17E-02	1.25E-01	-6.10E-02	6.01E-02	3.11E-01
diacylglycerol (14:0/18:1, 16:0/16:1) [1]*	-2.04E-02	6.37E-02	7.49E-01	-5.70E-02	6.16E-02	3.55E-01	-7.01E-02	5.98E-02	2.42E-01
diacylglycerol (14:0/18:1, 16:0/16:1) [2]*	5.86E-02	6.24E-02	3.49E-01	-8.55E-03	6.05E-02	8.88E-01	-8.10E-03	5.87E-02	8.90E-01
oleoyl-arachidonoyl-glycerol (18:1/20:4) [1]*	5.12E-02	6.39E-02	4.24E-01	-2.11E-02	6.19E-02	7.33E-01	1.93E-02	6.02E-02	7.49E-01
oleoyl-arachidonoyl-glycerol (18:1/20:4) [2]*	2.70E-02	6.35E-02	6.71E-01	-2.43E-02	6.15E-02	6.93E-01	-3.52E-03	5.97E-02	9.53E-01
palmitoyl-linolenoyl-glycerol (16:0/18:3) [2]*	5.37E-02	6.51E-02	4.10E-01	2.49E-02	6.31E-02	6.93E-01	-2.32E-02	6.13E-02	7.05E-01
diacylglycerol (16:1/18:2 [2], 16:0/18:3 [1])*	6.15E-02	6.52E-02	3.46E-01	2.60E-02	6.31E-02	6.81E-01	2.85E-02	6.13E-02	6.42E-01
linoleoyl-linoleoyl-glycerol (18:2/18:2) [1]*	2.77E-02	6.41E-02	6.66E-01	7.25E-02	6.19E-02	2.43E-01	6.36E-03	6.03E-02	9.16E-01
linoleoyl-linoleoyl-glycerol (18:2/18:2) [2]*	1.34E-01	6.54E-02	4.18E-02	1.04E-01	6.35E-02	1.03E-01	8.57E-02	6.18E-02	1.66E-01
stearoyl-arachidonoyl-glycerol (18:0/20:4) [1]*	1.02E-01	6.59E-02	1.22E-01	2.77E-03	6.40E-02	9.66E-01	1.08E-01	6.19E-02	8.26E-02
stearoyl-arachidonoyl-glycerol (18:0/20:4) [2]*	-3.08E-02	6.18E-02	6.18E-01	-1.11E-01	5.94E-02	6.32E-02	5.02E-02	5.80E-02	3.88E-01
perfluorooctanesulfonate (PFOS)	5.34E-02	5.82E-02	3.60E-01	1.06E-02	5.64E-02	8.52E-01	2.00E-02	5.48E-02	7.15E-01
1-palmityl-GPE (O-16:0)*	5.08E-02	6.31E-02	4.21E-01	-3.98E-02	6.11E-02	5.16E-01	4.71E-02	5.93E-02	4.28E-01
1-palmityl-2-stearoyl-GPC (O-16:0/18:0)*	-1.11E-01	6.54E-02	8.97E-02	-3.60E-02	6.36E-02	5.72E-01	-1.10E-01	6.15E-02	7.54E-02
1-palmityl-2-palmitoyl-GPC (O-16:0/16:0)*	-2.59E-01	6.34E-02	5.79E-05	-2.16E-01	6.19E-02	5.48E-04	-2.21E-01	5.99E-02	2.68E-04
1-stearyl-2-docosapentaenoyl-GPC (O-18:0/22:5n3)*	-1.27E-01	6.06E-02	3.65E-02	-4.75E-02	5.91E-02	4.22E-01	-9.54E-02	5.72E-02	9.64E-02
1-stearyl-2-linoleoyl-GPC (O-18:0/18:2)*	-1.08E-01	6.19E-02	8.25E-02	-4.65E-02	6.01E-02	4.40E-01	-4.23E-02	5.84E-02	4.69E-01
1-stearoyl-2-docosapentaenoyl-GPE (18:0/22:5n3)*	5.64E-02	6.52E-02	3.88E-01	6.10E-02	6.31E-02	3.35E-01	8.63E-02	6.12E-02	1.60E-01
1-stearoyl-2-docosapentaenoyl-GPE (18:0/22:5n6)*	-1.07E-01	6.41E-02	9.68E-02	-1.40E-01	6.18E-02	2.41E-02	-1.21E-01	6.01E-02	4.57E-02
1-stearoyl-2-adrenoyl-GPE (18:0/22:4)*	-6.02E-02	6.17E-02	3.30E-01	-9.19E-02	5.96E-02	1.24E-01	-6.50E-02	5.80E-02	2.64E-01
1-(1-enyl-stearoyl)-2-docosapentaenoyl-GPE (P-18:0/22:5n3)*	-1.51E-01	6.03E-02	1.28E-02	-1.68E-01	5.81E-02	4.07E-03	-6.33E-02	5.72E-02	2.70E-01
N-palmitoyl-sphingadienine (d18:2/16:0)*	4.62E-02	6.38E-02	4.69E-01	1.72E-02	6.18E-02	7.81E-01	8.34E-02	5.98E-02	1.64E-01
lactosyl-N-nervonoyl-sphingosine (d18:1/24:1)*	4.04E-02	5.95E-02	4.98E-01	2.15E-02	5.76E-02	7.09E-01	7.33E-02	5.58E-02	1.91E-01
glycosyl-N-behenoyl-sphingosine (d18:1/22:0)*	-2.02E-03	6.41E-02	9.75E-01	3.14E-02	6.20E-02	6.13E-01	3.56E-02	6.02E-02	5.55E-01
lactosyl-N-behenoyl-sphingosine (d18:1/22:0)*	-8.36E-03	6.12E-02	8.92E-01	1.04E-01	5.89E-02	8.00E-02	-3.45E-02	5.75E-02	5.49E-01
N-behenoyl-sphingadienine (d18:2/22:0)*	4.55E-02	6.35E-02	4.74E-01	1.30E-01	6.10E-02	3.34E-02	3.29E-02	5.97E-02	5.82E-01
glycosyl-N-behenoyl-sphingadienine (d18:2/22:0)*	-4.33E-03	6.28E-02	9.45E-01	-1.91E-02	6.08E-02	7.54E-01	7.95E-02	5.89E-02	1.78E-01
N-stearoyl-sphingadienine (d18:2/18:0)*	-1.30E-01	5.94E-02	2.90E-02	-1.29E-01	5.75E-02	2.61E-02	-9.33E-02	5.61E-02	9.73E-02
N-palmitoylserine	6.47E-02	6.46E-02	3.18E-01	-8.87E-03	6.27E-02	8.88E-01	-1.40E-02	6.09E-02	8.19E-01
N-oleoylserine	3.81E-02	6.73E-02	5.72E-01	3.97E-02	6.51E-02	5.43E-01	8.54E-02	6.31E-02	1.77E-01
sphingadienine	-9.39E-02	6.50E-02	1.50E-01	-6.81E-02	6.30E-02	2.81E-01	-6.68E-02	6.12E-02	2.76E-01
palmitoleoyl-arachidonoyl-glycerol (16:1/20:4) [2]*	5.21E-02	6.40E-02	4.17E-01	2.74E-02	6.20E-02	6.58E-01	3.77E-02	6.02E-02	5.31E-01
myristoyl-linoleoyl-glycerol (14:0/18:2) [2]*	1.07E-01	6.19E-02	8.49E-02	1.00E-01	5.99E-02	9.54E-02	3.20E-02	5.84E-02	5.84E-01
phosphatidylethanolamine (P-18:1/20:4, P-16:0/22:5n3)*	-2.39E-02	6.37E-02	7.07E-01	-1.23E-01	6.12E-02	4.49E-02	5.26E-02	5.98E-02	3.80E-01
1-stearoyl-2-(hydroxylinoleoyl)-GPC (18:0/18:2(OH))*	-1.20E-01	6.43E-02	6.19E-02	-1.34E-01	6.21E-02	3.12E-02	-4.62E-02	6.07E-02	4.47E-01
1-palmitoyl-2-(hydroxylinoleoyl)-GPC (16:0/18:2(OH))*	-1.62E-01	6.23E-02	9.84E-03	-1.75E-01	6.01E-02	3.87E-03	-4.87E-02	5.92E-02	4.12E-01
hexadecasphingosine (d16:1)*	-3.39E-02	6.38E-02	5.95E-01	-4.70E-02	6.17E-02	4.47E-01	-8.60E-02	5.97E-02	1.51E-01
ceramide (d16:1/24:1, d18:1/22:1)*	6.02E-03	6.24E-02	9.23E-01	-4.86E-02	6.03E-02	4.21E-01	-9.98E-02	5.84E-02	8.85E-02
N-palmitoyl-heptadecasphingosine (d17:1/16:0)*	-9.29E-02	6.28E-02	1.40E-01	-9.15E-02	6.08E-02	1.33E-01	-1.62E-01	5.85E-02	6.01E-03
ceramide (d18:1/14:0, d16:1/16:0)*	8.63E-02	6.41E-02	1.79E-01	6.56E-02	6.21E-02	2.92E-01	7.24E-04	6.05E-02	9.90E-01
ceramide (d18:1/17:0, d17:1/18:0)*	-1.43E-01	6.18E-02	2.11E-02	-1.29E-01	5.99E-02	3.17E-02	-1.66E-01	5.78E-02	4.33E-03
ceramide (d18:2/24:1, d18:1/24:2)*	-2.77E-02	6.36E-02	6.63E-01	-5.86E-02	6.15E-02	3.41E-01	-3.62E-02	5.98E-02	5.45E-01
glycosyl ceramide (d18:2/24:1, d18:1/24:2)*	-6.56E-02	6.12E-02	2.85E-01	-1.12E-01	5.90E-02	5.96E-02	3.13E-02	5.76E-02	5.87E-01
glycosyl-N-tricosanoyl-sphingadienine (d18:2/23:0)*	-4.18E-02	6.13E-02	4.95E-01	-7.21E-02	5.92E-02	2.24E-01	4.92E-02	5.76E-02	3.94E-01
glycosyl ceramide (d18:1/23:1, d17:1/24:1)*	-1.49E-01	6.25E-02	1.77E-02	-1.41E-01	6.05E-02	2.02E-02	-1.83E-01	5.83E-02	1.89E-03
glycosyl-N-(2-hydroxynervonoyl)-sphingosine (d18:1/24:1(2OH))*	3.68E-02	5.94E-02	5.35E-01	-9.74E-02	5.72E-02	8.96E-02	-9.32E-03	5.59E-02	8.68E-01
ceramide (d18:1/20:0, d16:1/22:0, d20:1/18:0)*	2.69E-02	6.33E-02	6.71E-01	6.51E-02	6.12E-02	2.88E-01	-7.31E-02	5.94E-02	2.19E-01
stearoylcholine*	1.49E-01	6.40E-02	2.03E-02	1.86E-01	6.16E-02	2.70E-03	1.63E-01	6.00E-02	7.17E-03
linoleoylcholine*	1.59E-01	6.24E-02	1.13E-02	1.88E-01	6.01E-02	1.94E-03	1.74E-01	5.84E-02	3.20E-03
1-adrenoyl-GPE (22:4)*	-1.37E-01	6.20E-02	2.83E-02	-8.83E-02	6.03E-02	1.44E-01	-1.43E-01	5.82E-02	1.43E-02
1-docosapentaenoyl-GPE (22:5n3)*	-1.22E-01	6.34E-02	5.62E-02	-7.62E-02	6.16E-02	2.17E-01	-4.98E-02	6.00E-02	4.07E-01
trazadone	1.09E-02	6.26E-02	8.62E-01	-4.49E-02	6.05E-02	4.58E-01	-6.72E-02	5.87E-02	2.53E-01
nisinate (24:6n3)	1.19E-01	6.69E-02	7.52E-02	1.31E-03	6.51E-02	9.84E-01	1.38E-01	6.27E-02	2.81E-02
sphingomyelin (d18:0/20:0, d16:0/22:0)*	-6.93E-02	6.47E-02	2.85E-01	-2.06E-01	6.15E-02	9.08E-04	-4.31E-02	6.09E-02	4.80E-01
sphingomyelin (d18:1/19:0, d19:1/18:0)*	-1.32E-01	5.83E-02	2.40E-02	-1.47E-01	5.63E-02	9.55E-03	-1.33E-01	5.48E-02	1.61E-02
sphingomyelin (d18:2/18:1)*	-1.97E-01	5.74E-02	6.84E-04	-1.46E-01	5.60E-02	9.87E-03	-1.18E-01	5.46E-02	3.22E-02
sphingomyelin (d18:2/24:2)*	-1.48E-01	5.87E-02	1.23E-02	-1.57E-01	5.67E-02	5.92E-03	-8.07E-02	5.56E-02	1.48E-01
sphingomyelin (d18:2/21:0, d16:2/23:0)*	-8.24E-02	5.26E-02	1.18E-01	-7.37E-02	5.09E-02	1.49E-01	-6.33E-02	4.95E-02	2.02E-01
sphingomyelin (d18:2/23:1)*	-1.62E-01	5.60E-02	4.12E-03	-2.07E-01	5.36E-02	1.42E-04	-1.55E-01	5.27E-02	3.55E-03
sphingomyelin (d18:1/25:0, d19:0/24:1, d20:1/23:0, d19:1/24:0)*	-9.27E-02	6.28E-02	1.41E-01	-1.08E-01	6.07E-02	7.73E-02	-1.02E-01	5.89E-02	8.58E-02
sphingomyelin (d17:2/16:0, d18:2/15:0)*	-5.37E-02	5.41E-02	3.21E-01	-5.96E-02	5.23E-02	2.55E-01	-3.79E-02	5.09E-02	4.57E-01
heneicosapentaenoate (21:5n3)	1.20E-01	6.38E-02	6.03E-02	-4.74E-02	6.21E-02	4.46E-01	1.63E-01	5.96E-02	6.77E-03
linolenoylcarnitine (C18:3)*	-7.19E-02	6.41E-02	2.63E-01	-3.99E-02	6.22E-02	5.21E-01	4.69E-03	6.04E-02	9.38E-01
behenoylcarnitine (C22)*	1.01E-01	6.73E-02	1.35E-01	6.93E-02	6.53E-02	2.89E-01	9.18E-02	6.33E-02	1.48E-01

arachidoylcarnitine (C20)*	5.03E-02	6.27E-02	4.23E-01	9.13E-02	6.05E-02	1.33E-01	4.54E-02	5.90E-02	4.42E-01
lignoceroylcarnitine (C24)*	1.07E-01	6.67E-02	1.11E-01	9.68E-02	6.46E-02	1.35E-01	1.06E-01	6.27E-02	9.09E-02
cerotoylcarnitine (C26)*	1.72E-01	6.41E-02	7.88E-03	1.50E-01	6.22E-02	1.65E-02	1.33E-01	6.05E-02	2.86E-02
ximenoylcarnitine (C26:1)*	1.48E-01	6.36E-02	2.05E-02	2.56E-02	6.21E-02	6.80E-01	1.36E-01	5.98E-02	2.35E-02
arachidonoylcarnitine (C20:4)	4.80E-02	6.08E-02	4.31E-01	3.86E-02	5.89E-02	5.13E-01	6.42E-02	5.71E-02	2.62E-01
eicosenoylcarnitine (C20:1)*	1.65E-02	5.94E-02	7.81E-01	7.44E-03	5.75E-02	8.97E-01	1.45E-02	5.59E-02	7.95E-01
dihomo-linoleoylcarnitine (C20:2)*	-1.86E-02	6.19E-02	7.64E-01	6.99E-03	5.99E-02	9.07E-01	3.11E-02	5.82E-02	5.93E-01
dihomo-linolenoylcarnitine (C20:3n3 or 6)*	6.95E-02	6.13E-02	2.58E-01	5.72E-02	5.94E-02	3.36E-01	3.58E-02	5.78E-02	5.36E-01
erucoylcarnitine (C22:1)*	-1.13E-03	5.82E-02	9.84E-01	-1.03E-03	5.63E-02	9.85E-01	-1.62E-02	5.47E-02	7.68E-01
nervonoylcarnitine (C24:1)*	1.80E-02	6.37E-02	7.77E-01	-7.07E-02	6.15E-02	2.52E-01	4.18E-02	5.98E-02	4.85E-01
adrenoylcarnitine (C22:4)*	1.27E-03	6.01E-02	9.83E-01	5.99E-02	5.80E-02	3.03E-01	1.72E-02	5.65E-02	7.61E-01
glycosyl ceramide (d18:1/20:0, d16:1/22:0)*	-3.66E-02	6.06E-02	5.47E-01	-5.63E-02	5.86E-02	3.38E-01	8.47E-03	5.70E-02	8.82E-01
1-lignoceroyl-2-arachidonoyl-GPC (24:0/20:4)*	2.34E-01	6.32E-02	2.60E-04	1.63E-01	6.18E-02	8.76E-03	2.19E-01	5.94E-02	2.70E-04
1-nervonoyl-2-arachidonoyl-GPC (24:1/20:4)*	7.63E-02	6.41E-02	2.35E-01	-8.98E-02	6.19E-02	1.48E-01	1.08E-01	6.00E-02	7.19E-02
N-oxalyl glycine (NOG)	8.94E-02	6.23E-02	1.52E-01	1.12E-01	6.01E-02	6.46E-02	9.60E-02	5.85E-02	1.02E-01
N,N,N-trimethyl-5-aminovalerate	9.16E-03	6.25E-02	8.84E-01	5.77E-02	6.04E-02	3.40E-01	-3.66E-02	5.87E-02	5.34E-01
ethyl alpha-glucopyranoside	-2.67E-02	6.17E-02	6.66E-01	-3.07E-01	5.68E-02	1.34E-07	-2.30E-02	5.80E-02	6.92E-01
carotene diol (1)	2.37E-01	5.83E-02	6.10E-05	1.28E-01	5.76E-02	2.73E-02	2.33E-01	5.47E-02	2.88E-05
carotene diol (2)	2.31E-01	5.72E-02	6.69E-05	1.49E-01	5.62E-02	8.43E-03	2.04E-01	5.39E-02	1.91E-04
carotene diol (3)	1.25E-01	6.44E-02	5.25E-02	7.82E-02	6.26E-02	2.13E-01	1.27E-01	6.05E-02	3.66E-02
cortolone glucuronide (1)	-1.23E-01	5.91E-02	3.86E-02	-8.30E-02	5.74E-02	1.49E-01	-6.93E-02	5.58E-02	2.15E-01
fluconazole	2.25E-02	5.41E-02	6.78E-01	-1.58E-03	5.24E-02	9.76E-01	-3.71E-03	5.09E-02	9.42E-01
Fibrinopeptide B	8.34E-02	6.73E-02	2.17E-01	6.31E-02	6.52E-02	3.34E-01	6.00E-02	6.34E-02	3.45E-01
1-oleyl-2-linoleoyl-GPC (O-18:1/18:2)*	1.24E-02	6.37E-02	8.45E-01	3.48E-02	6.16E-02	5.72E-01	5.72E-02	5.98E-02	3.40E-01
1-palmityl-2-dihomo-linolenoyl-GPC (O-16:0/20:3)*	-3.83E-02	6.63E-02	5.64E-01	-1.26E-01	6.38E-02	5.00E-02	-6.39E-02	6.23E-02	3.06E-01
phosphatidylcholine (O-18:1/20:4, O-16:0/22:5n3)*	-3.09E-02	6.30E-02	6.24E-01	-8.66E-02	6.08E-02	1.55E-01	2.71E-02	5.92E-02	6.47E-01
1-palmitoyl-2-pentadecanoyl-GPC (16:0/15:0)*	-8.56E-02	6.04E-02	1.58E-01	-9.34E-03	5.87E-02	8.74E-01	-1.31E-01	5.65E-02	2.14E-02
(N(1) + N(8))-acetylspermidine	2.04E-02	6.17E-02	7.42E-01	3.95E-02	5.97E-02	5.09E-01	4.92E-02	5.80E-02	3.97E-01
THC carboxylic acid	3.27E-02	6.02E-02	5.88E-01	-5.97E-02	5.82E-02	3.06E-01	3.75E-02	5.65E-02	5.08E-01
THC carboxylic acid glucuronide	3.45E-02	5.94E-02	5.63E-01	-5.11E-02	5.75E-02	3.75E-01	3.37E-02	5.59E-02	5.47E-01
5-dodecenoylcarnitine (C12:1)	-2.26E-01	6.18E-02	3.01E-04	-2.51E-01	5.93E-02	3.17E-05	-1.38E-01	5.89E-02	1.98E-02
2-butenoylglycine	-3.37E-02	5.99E-02	5.75E-01	3.26E-02	5.80E-02	5.74E-01	4.73E-02	5.63E-02	4.02E-01
hydroxy-CMPF*	1.48E-01	6.18E-02	1.74E-02	7.30E-02	6.02E-02	2.27E-01	2.04E-01	5.74E-02	4.36E-04
3-hydroxyoleoylcarnitine	-4.22E-02	6.04E-02	4.85E-01	-5.20E-02	5.85E-02	3.75E-01	-4.75E-02	5.68E-02	4.04E-01
3-hydroxyphenylacetoylglutamine	1.73E-01	6.15E-02	5.31E-03	1.63E-01	5.95E-02	6.43E-03	1.41E-01	5.80E-02	1.57E-02
trans-2-hexenoylglycine	-1.35E-01	5.77E-02	1.97E-02	-5.67E-02	5.63E-02	3.15E-01	-5.79E-02	5.47E-02	2.91E-01
2-hydroxyarachidate*	-9.99E-02	6.32E-02	1.15E-01	-1.35E-01	6.09E-02	2.69E-02	-1.16E-01	5.93E-02	5.24E-02
N-stearoylserine*	-8.43E-02	6.48E-02	1.94E-01	-8.98E-02	6.27E-02	1.54E-01	5.19E-03	6.11E-02	9.32E-01
lyxonate	1.23E-01	6.09E-02	4.39E-02	1.15E-02	5.94E-02	8.47E-01	4.52E-02	5.77E-02	4.34E-01
dodecenedioate (C12:1-DC)*	5.86E-02	6.25E-02	3.49E-01	4.32E-02	6.05E-02	4.76E-01	2.28E-02	5.88E-02	6.98E-01
hexadecenedioate (C16:1-DC)*	-1.33E-01	6.30E-02	3.50E-02	-1.16E-01	6.11E-02	5.82E-02	-1.26E-01	5.92E-02	3.41E-02
octadecenedioate (C18:1-DC)	7.55E-03	6.46E-02	9.07E-01	8.14E-02	6.23E-02	1.93E-01	1.25E-02	6.07E-02	8.37E-01
heptenedioate (C7:1-DC)*	-2.65E-01	6.03E-02	1.57E-05	-8.10E-02	6.01E-02	1.79E-01	-2.31E-01	5.69E-02	6.30E-05
octadecadienedioate (C18:2-DC)*	3.21E-02	6.72E-02	6.33E-01	2.17E-02	6.51E-02	7.39E-01	3.63E-02	6.32E-02	5.66E-01
glucuronide of C12H22O4 (1)*	1.59E-01	6.52E-02	1.54E-02	2.29E-01	6.23E-02	2.82E-04	5.88E-02	6.18E-02	3.42E-01
glucuronide of C10H18O2 (1)*	2.67E-02	6.40E-02	6.76E-01	2.16E-02	6.19E-02	7.27E-01	6.31E-02	6.00E-02	2.94E-01
3-carboxy-4-methyl-5-pentyl-2-furanpropionate (3-CMPFP)**	-6.04E-02	6.01E-02	3.16E-01	-1.04E-02	5.82E-02	8.58E-01	3.15E-03	5.66E-02	9.56E-01
methylnaphthyl sulfate (1)*	2.11E-02	4.94E-02	6.70E-01	-3.07E-02	4.78E-02	5.21E-01	-7.83E-02	4.62E-02	9.14E-02
methylnaphthyl sulfate (2)*	-4.17E-02	5.17E-02	4.21E-01	-8.96E-02	4.98E-02	7.30E-02	3.99E-03	4.87E-02	9.35E-01
glucuronide of C10H18O2 (7)*	2.08E-02	6.44E-02	7.47E-01	-1.04E-03	6.23E-02	9.87E-01	3.17E-02	6.05E-02	6.00E-01
glucuronide of C10H18O2 (8)*	5.66E-02	6.57E-02	3.90E-01	-1.54E-02	6.37E-02	8.09E-01	4.75E-02	6.18E-02	4.43E-01
N-acetyl-2-aminooctanoate*	1.49E-02	6.31E-02	8.13E-01	5.96E-02	6.10E-02	3.29E-01	3.14E-02	5.93E-02	5.96E-01
hydroxyasparagine**	-2.38E-02	5.42E-02	6.61E-01	4.28E-02	5.25E-02	4.16E-01	-7.08E-02	5.08E-02	1.65E-01
perfluorooctanoate (PFOA)	-1.90E-02	5.74E-02	7.41E-01	-7.01E-02	5.54E-02	2.07E-01	-3.86E-03	5.40E-02	9.43E-01
pyroglutamylphenylalanine	3.94E-02	6.43E-02	5.40E-01	-1.27E-02	6.23E-02	8.38E-01	3.70E-02	6.05E-02	5.42E-01
3-hydroxybutyroylglycine**	-1.15E-01	5.99E-02	5.53E-02	-4.95E-02	5.83E-02	3.96E-01	-7.59E-03	5.67E-02	8.94E-01
carboxyibuprofen glucuronide*	6.14E-02	6.22E-02	3.25E-01	-4.30E-02	6.03E-02	4.76E-01	-7.59E-03	5.86E-02	8.97E-01
glyco-beta-muricholate**	1.12E-01	6.57E-02	8.93E-02	1.23E-01	6.35E-02	5.37E-02	1.52E-02	6.21E-02	8.07E-01
N-methylhydroxyproline**	1.75E-01	6.61E-02	8.77E-03	1.44E-01	6.42E-02	2.59E-02	1.02E-01	6.26E-02	1.05E-01
N,N,N-trimethyl-alanylproline betaine (TMAP)	-9.40E-03	5.97E-02	8.75E-01	1.34E-01	5.73E-02	2.01E-02	-6.43E-02	5.60E-02	2.52E-01
3-formylindole	1.53E-01	5.84E-02	9.27E-03	1.60E-01	5.64E-02	4.86E-03	1.18E-01	5.52E-02	3.37E-02
1-(1-enyl-oleoyl)-2-docosahexaenoyl-GPC (P-18:1/22:6)*	1.74E-01	5.56E-02	1.94E-03	2.95E-02	5.47E-02	5.90E-01	2.34E-01	5.13E-02	7.84E-06
pyroglutamylalanine*	-1.97E-02	6.52E-02	7.63E-01	4.60E-02	6.31E-02	4.66E-01	-7.83E-02	6.12E-02	2.01E-01
pyroglutamylproline*	-1.53E-01	6.61E-02	2.16E-02	-1.18E-01	6.42E-02	6.64E-02	-1.02E-01	6.24E-02	1.03E-01
pyroglutamylleucine*	5.03E-03	6.40E-02	9.37E-01	5.51E-02	6.18E-02	3.74E-01	-9.03E-02	5.99E-02	1.33E-01
pyroglutamylisoleucine*	2.23E-02	6.59E-02	7.36E-01	2.11E-02	6.38E-02	7.41E-01	-4.64E-02	6.19E-02	4.54E-01
gamma-glutamylcitrulline*	-1.19E-01	6.37E-02	6.25E-02	-7.83E-02	6.19E-02	2.07E-01	-7.81E-02	6.01E-02	1.95E-01
resveratrol disulfate (1)*	2.31E-02	6.55E-02	7.25E-01	-9.68E-02	6.31E-02	1.27E-01	2.08E-02	6.16E-02	7.36E-01
resveratrol disulfate (2)*	5.04E-03	6.58E-02	9.39E-01	-1.68E-01	6.29E-02	8.12E-03	1.90E-03	6.18E-02	9.76E-01
glycine conjugate of C10H12O2*	-1.04E-01	6.50E-02	1.11E-01	-1.36E-01	6.26E-02	3.09E-02	-1.25E-01	6.09E-02	4.07E-02
glycine conjugate of C10H14O2 (1)*	-2.63E-01	6.36E-02	4.59E-05	-2.47E-01	6.16E-02	8.06E-05	-2.17E-01	6.02E-02	3.73E-04
glutamine conjugate of C7H12O2*	-1.87E-01	6.13E-02	2.50E-03	-8.39E-02	6.01E-02	1.64E-01	-1.36E-01	5.80E-02	1.99E-02
glutamine conjugate of C6H10O2 (1)*	-1.01E-01	6.24E-02	1.07E-01	-2.72E-02	6.07E-02	6.54E-01	-5.40E-02	5.89E-02	3.60E-01
glutamine conjugate of C6H10O2 (2)*	-1.26E-01	6.05E-02	3.87E-02	-7.80E-02	5.88E-02	1.86E-01	-7.37E-02	5.71E-02	1.98E-01
dihydroferulic acid sulfate	1.10E-01	6.64E-02	9.99E-02	1.55E-01	6.39E-02	1.61E-02	4.46E-02	6.27E-02	4.77E-01
sphingomyelin (d17:1/14:0, d16:1/15:0)*	-2.10E-02	5.61E-02	7.09E-01	-7.18E-03	5.43E-02	8.95E-01	-4.26E-02	5.27E-02	4.19E-01
tetradecadienoate (14:2)*	-1.42E-01	6.34E-02	2.58E-02	-1.63E-01	6.11E-02	8.21E-03	-8.33E-02	5.99E-02	1.66E-01
8-methoxykynurenate	-4.79E-02	6.44E-02	4.57E-01	-6.96E-02	6.23E-02	2.64E-01	-6.88E-02	6.05E-02	2.56E-01
3-amino-2-piperidone	4.69E-02	5.94E-02	4.30E-01	1.24E-01	5.71E-02	3.05E-02	5.99E-02	5.58E-02	2.84E-01
N,N-dimethylalanine	1.26E-02	6.21E-02	8.40E-01	-3.02E-03	6.01E-02	9.60E-01	4.98E-02	5.83E-02	3.94E-01
3-indoleglyoxylic acid	1.58E-01	6.17E-02	1.08E-02	1.32E-01	5.99E-02	2.85E-02	8.36E-02	5.85E-02	1.54E-01
ethyl beta-glucopyranoside	1.08E-01	6.13E-02	8.00E-02	-3.52E-02	5.97E-02	5.55E-01	8.27E-02	5.78E-02	1.54E-01

2-hydroxysebacate	1.52E-01	6.39E-02	1.78E-02	1.76E-01	6.16E-02	4.62E-03	9.80E-02	6.04E-02	1.06E-01
enterolactone sulfate	1.19E-01	6.22E-02	5.77E-02	4.51E-02	6.06E-02	4.57E-01	7.92E-02	5.87E-02	1.78E-01
ascorbic acid 3-sulfate*	1.85E-01	6.39E-02	4.18E-03	2.30E-01	6.12E-02	2.12E-04	8.33E-02	6.08E-02	1.71E-01
3-hydroxyhippurate sulfate	1.14E-01	6.47E-02	7.79E-02	1.31E-01	6.24E-02	3.75E-02	1.27E-01	6.07E-02	3.66E-02
6-bromotryptophan	2.24E-02	6.44E-02	7.28E-01	8.43E-03	6.23E-02	8.93E-01	-5.14E-02	6.05E-02	3.96E-01
delta-CEHC	-2.31E-01	6.41E-02	3.76E-04	-8.04E-02	6.33E-02	2.05E-01	-1.45E-01	6.11E-02	1.84E-02
N6,N6-dimethyllysine	-5.14E-02	5.87E-02	3.83E-01	-3.01E-02	5.69E-02	5.97E-01	2.45E-03	5.53E-02	9.65E-01
1-carboxyethylphenylalanine	6.29E-03	5.32E-02	9.06E-01	9.57E-02	5.12E-02	6.26E-02	-7.67E-03	5.00E-02	8.78E-01
1-carboxyethyltyrosine	1.38E-02	5.37E-02	7.97E-01	7.90E-02	5.18E-02	1.28E-01	1.12E-02	5.05E-02	8.24E-01
1-carboxyethylvaline	3.31E-02	5.54E-02	5.51E-01	1.26E-01	5.31E-02	1.82E-02	3.32E-02	5.20E-02	5.24E-01
1-carboxyethylleucine	1.80E-04	5.50E-02	9.97E-01	9.66E-03	5.32E-02	8.56E-01	-1.13E-02	5.17E-02	8.28E-01
1-carboxyethylisoleucine	1.22E-03	5.52E-02	9.82E-01	8.79E-02	5.32E-02	9.96E-02	3.67E-03	5.19E-02	9.44E-01
dodecadienoate (12:2)*	-5.17E-02	6.28E-02	4.11E-01	-7.21E-02	6.07E-02	2.36E-01	-3.43E-02	5.91E-02	5.62E-01
indoleacetoylcarnitine*	-7.84E-02	6.14E-02	2.03E-01	-1.86E-01	5.85E-02	1.66E-03	4.11E-02	5.78E-02	4.78E-01
gamma-CEHC sulfate	-3.37E-02	6.20E-02	5.87E-01	6.67E-02	5.99E-02	2.66E-01	-6.89E-02	5.81E-02	2.37E-01
delta-CEHC glucuronide	-1.56E-01	6.41E-02	1.54E-02	-3.67E-03	6.27E-02	9.53E-01	-9.73E-02	6.06E-02	1.10E-01
N-acetyl-isoputreanine	6.51E-02	6.35E-02	3.06E-01	2.46E-02	6.16E-02	6.90E-01	8.10E-02	5.96E-02	1.75E-01
glucuronide of piperine metabolite C17H21NO3 (3)*	7.85E-02	6.57E-02	2.33E-01	-8.65E-02	6.36E-02	1.75E-01	9.09E-02	6.17E-02	1.42E-01
glucuronide of piperine metabolite C17H21NO3 (4)*	5.96E-02	6.40E-02	3.53E-01	-9.73E-02	6.18E-02	1.16E-01	9.68E-02	6.00E-02	1.08E-01
glucuronide of piperine metabolite C17H21NO3 (5)*	6.69E-02	6.48E-02	3.03E-01	-1.14E-01	6.25E-02	6.89E-02	9.34E-02	6.08E-02	1.26E-01
sulfate of piperine metabolite C16H19NO3 (2)*	5.99E-02	6.40E-02	3.50E-01	-1.28E-01	6.16E-02	3.87E-02	8.85E-02	6.01E-02	1.42E-01
sulfate of piperine metabolite C16H19NO3 (3)*	5.82E-02	6.33E-02	3.59E-01	-1.15E-01	6.10E-02	5.95E-02	8.53E-02	5.94E-02	1.52E-01
sulfate of piperine metabolite C18H21NO3 (1)*	1.02E-02	6.20E-02	8.69E-01	-1.21E-01	5.96E-02	4.36E-02	4.53E-02	5.82E-02	4.37E-01
5-hydroxyindole glucuronide	3.14E-02	6.72E-02	6.40E-01	3.90E-02	6.50E-02	5.49E-01	3.07E-02	6.32E-02	6.28E-01
N-acetylhomocitrulline	-3.19E-03	6.20E-02	9.59E-01	-3.28E-02	5.99E-02	5.84E-01	-5.24E-02	5.82E-02	3.69E-01
2-naphthol sulfate	-5.06E-02	5.78E-02	3.82E-01	-8.30E-02	5.58E-02	1.38E-01	-6.18E-02	5.43E-02	2.56E-01
(2,4 or 2,5)-dimethylphenol sulfate	-7.19E-02	5.58E-02	1.99E-01	-1.62E-01	5.33E-02	2.56E-03	-4.49E-03	5.27E-02	9.32E-01
4-ethylcatechol sulfate	9.09E-02	6.17E-02	1.42E-01	1.15E-01	5.96E-02	5.37E-02	3.66E-02	5.82E-02	5.31E-01
11beta-hydroxyandrosterone glucuronide	-2.94E-02	6.46E-02	6.50E-01	4.58E-02	6.25E-02	4.64E-01	-7.60E-02	6.06E-02	2.11E-01
11beta-hydroxyetiocholanolone glucuronide*	4.24E-02	6.33E-02	5.03E-01	1.81E-02	6.13E-02	7.68E-01	-6.07E-02	5.94E-02	3.08E-01
N2-acetyl,N6-methyllysine	-9.33E-02	6.60E-02	1.59E-01	-1.13E-01	6.38E-02	7.63E-02	-8.79E-03	6.23E-02	8.88E-01
cholic acid glucuronide	9.25E-02	6.61E-02	1.62E-01	7.43E-02	6.40E-02	2.47E-01	1.10E-01	6.20E-02	7.73E-02
deoxycholic acid glucuronide	2.28E-02	6.53E-02	7.27E-01	-4.28E-02	6.32E-02	4.99E-01	9.58E-02	6.12E-02	1.18E-01
4-allylcatechol sulfate	1.26E-01	6.68E-02	6.04E-02	1.19E-01	6.47E-02	6.62E-02	1.16E-01	6.29E-02	6.65E-02
2-hydroxyfluorene sulfate	-2.90E-02	5.27E-02	5.83E-01	-9.07E-02	5.07E-02	7.51E-02	-2.41E-03	4.96E-02	9.61E-01
vanillate glucuronide	6.56E-02	6.70E-02	3.28E-01	1.12E-01	6.46E-02	8.35E-02	2.87E-03	6.31E-02	9.64E-01
4-methylhexanoylglutamine	-2.13E-01	6.23E-02	7.41E-04	-1.92E-01	6.05E-02	1.66E-03	-2.28E-01	5.82E-02	1.17E-04
Benazeprilat	1.45E-01	7.04E-02	4.00E-02	6.76E-02	6.86E-02	3.25E-01	-5.37E-02	6.66E-02	4.21E-01
1-nonadecenoyl-GPC (19:1)*	9.15E-02	5.99E-02	1.28E-01	6.72E-02	5.81E-02	2.48E-01	3.05E-02	5.66E-02	5.91E-01
desmethylcitalopram*	-1.45E-01	7.02E-02	4.02E-02	-2.56E-02	6.84E-02	7.09E-01	-2.16E-02	6.65E-02	7.46E-01
citalopram propionate*	-8.02E-02	6.82E-02	2.41E-01	-1.79E-02	6.61E-02	7.86E-01	-1.50E-03	6.43E-02	9.81E-01
4-hydroxy duloxetine glucuronide*	-7.03E-18	1.19E-17	5.54E-01	-2.63E-17	1.14E-17	2.16E-02	-7.49E-18	1.12E-17	5.02E-01
5-hydroxy-6-methoxy duloxetine sulfate*	-2.33E-02	7.30E-02	7.49E-01	-1.21E-02	7.07E-02	8.64E-01	-1.80E-02	6.86E-02	7.94E-01
N-desalkylquetiapine*	4.94E-02	7.07E-02	4.85E-01	-3.66E-02	6.84E-02	5.93E-01	-7.31E-02	6.64E-02	2.71E-01
ranitidine N-oxide*	-5.41E-02	7.12E-02	4.49E-01	-5.13E-03	6.90E-02	9.41E-01	1.52E-02	6.70E-02	8.21E-01
7-hydroxywarfarin	6.07E-02	7.14E-02	3.96E-01	8.26E-02	6.90E-02	2.33E-01	-1.13E-02	6.72E-02	8.66E-01
glycoursodeoxycholic acid sulfate (1)	-1.56E-01	6.00E-02	9.80E-03	-1.60E-01	5.80E-02	6.21E-03	-1.33E-01	5.65E-02	1.95E-02
dihydrocaffeate sulfate (2)	2.22E-01	6.46E-02	6.71E-04	1.92E-01	6.28E-02	2.40E-03	1.86E-01	6.10E-02	2.56E-03
lithocholate sulfate (1)	6.76E-02	6.50E-02	2.99E-01	1.06E-01	6.27E-02	9.30E-02	-1.15E-01	6.08E-02	5.96E-02
3-hydroxyhexanoylcarnitine (1)	-4.72E-02	6.52E-02	4.69E-01	3.51E-02	6.31E-02	5.79E-01	-1.46E-02	6.13E-02	8.13E-01
3-(methylthio)acetaminophen sulfate*	8.86E-02	6.47E-02	1.72E-01	1.32E-01	6.23E-02	3.52E-02	8.24E-02	6.08E-02	1.76E-01
2-ketocaprylate	1.51E-01	6.23E-02	1.59E-02	1.46E-01	6.03E-02	1.62E-02	7.14E-02	5.91E-02	2.28E-01
2,6-dihydroxybenzoic acid	2.39E-01	6.09E-02	1.11E-04	3.14E-01	5.75E-02	1.05E-07	1.74E-01	5.79E-02	2.85E-03
tetrahydrocortisol sulfate (1)	-1.09E-01	6.12E-02	7.52E-02	-3.69E-02	5.95E-02	5.36E-01	-1.40E-01	5.73E-02	1.51E-02
3-ethylcatechol sulfate (1)	-6.23E-02	6.17E-02	3.13E-01	-1.64E-01	5.90E-02	5.73E-03	-2.20E-02	5.81E-02	7.05E-01
3-ethylcatechol sulfate (2)	-1.19E-02	5.68E-02	8.34E-01	-7.20E-03	5.49E-02	8.96E-01	-1.89E-03	5.34E-02	9.72E-01
4-acetylcatechol sulfate (1)	1.64E-01	6.13E-02	7.97E-03	1.93E-01	5.89E-02	1.18E-03	1.20E-01	5.79E-02	3.95E-02
1-(14 or 15-methyl)palmitoyl-GPC (a17:0 or i17:0)*	-7.54E-02	6.18E-02	2.24E-01	-2.31E-02	5.99E-02	7.00E-01	-1.31E-01	5.77E-02	2.39E-02
montelukast	-5.58E-02	7.19E-02	4.38E-01	4.27E-02	6.96E-02	5.40E-01	-9.59E-02	6.75E-02	1.56E-01
2,3-dihydroxy-5-methylthio-4-pentenoate (DMTPA)*	-4.27E-02	5.45E-02	4.34E-01	7.74E-02	5.26E-02	1.42E-01	-9.17E-02	5.10E-02	7.32E-02
Fibrinopeptide A (3-16)**	9.69E-02	6.73E-02	1.51E-01	8.98E-02	6.52E-02	1.70E-01	4.86E-02	6.35E-02	4.44E-01
meloxicam	-9.67E-02	7.21E-02	1.81E-01	2.11E-02	7.00E-02	7.63E-01	-3.95E-02	6.79E-02	5.62E-01
Fibrinopeptide B (1-13)**	8.96E-02	6.76E-02	1.86E-01	9.94E-02	6.53E-02	1.29E-01	2.30E-02	6.37E-02	7.19E-01
losartan	-1.01E-01	6.40E-02	1.17E-01	-4.32E-02	6.21E-02	4.88E-01	-5.02E-02	6.03E-02	4.06E-01
cyclobenzaprine	-3.02E-02	5.73E-02	5.98E-01	-5.89E-02	5.54E-02	2.88E-01	-1.31E-03	5.39E-02	9.81E-01
emtricitabine	-1.12E-02	7.22E-02	8.76E-01	-7.98E-02	6.97E-02	2.53E-01	2.93E-02	6.79E-02	6.66E-01
darunavir	-2.34E-02	7.24E-02	7.46E-01	-8.12E-02	6.99E-02	2.47E-01	3.74E-03	6.81E-02	9.56E-01
5-hydroxy-2-methylpyridine sulfate	-3.21E-03	6.28E-02	9.59E-01	-2.16E-02	6.08E-02	7.23E-01	-9.23E-02	5.88E-02	1.18E-01
3-hydroxy-2-methylpyridine sulfate	9.60E-02	6.17E-02	1.21E-01	3.62E-02	6.00E-02	5.47E-01	1.04E-01	5.79E-02	7.48E-02
hydroxypalmitoyl sphingomyelin (d18:1/16:0(OH))**	1.98E-02	6.40E-02	7.57E-01	3.77E-02	6.19E-02	5.43E-01	1.73E-02	6.01E-02	7.74E-01
tamoxifen	-7.03E-18	1.19E-17	5.54E-01	-2.63E-17	1.14E-17	2.16E-02	-7.49E-18	1.12E-17	5.02E-01
taurochenodeoxycholic acid 3-sulfate	-6.45E-02	6.34E-02	3.10E-01	-6.98E-02	6.13E-02	2.56E-01	-6.86E-02	5.96E-02	2.50E-01
tetradecadienedioate (C14:2-DC)*	-5.22E-02	6.25E-02	4.05E-01	-7.46E-02	6.04E-02	2.18E-01	-8.22E-02	5.86E-02	1.62E-01
pregnenetriol sulfate*	-1.31E-01	5.85E-02	2.56E-02	-1.10E-01	5.68E-02	5.26E-02	-1.70E-01	5.46E-02	2.04E-03
pregnenetriol disulfate*	-1.79E-01	5.90E-02	2.66E-03	-1.67E-01	5.72E-02	3.85E-03	-1.76E-01	5.54E-02	1.68E-03
eicosenedioate (C20:1-DC)*	-2.16E-01	6.38E-02	8.04E-04	-1.60E-01	6.23E-02	1.06E-02	-1.64E-01	6.04E-02	6.90E-03
hydroxy-N6,N6,N6-trimethyllysine*	-1.64E-01	6.56E-02	1.32E-02	-3.89E-02	6.42E-02	5.45E-01	-1.19E-01	6.20E-02	5.56E-02
undecenoylcarnitine (C11:1)	-1.30E-01	5.86E-02	2.68E-02	-6.59E-02	5.71E-02	2.49E-01	-1.35E-01	5.50E-02	1.48E-02
3-decenoylcarnitine	-3.52E-02	6.30E-02	5.76E-01	1.82E-02	6.10E-02	7.65E-01	-5.60E-02	5.91E-02	3.44E-01
3-hydroxydecanoylcarnitine	-5.01E-02	6.03E-02	4.07E-01	2.71E-02	5.84E-02	6.43E-01	-3.83E-02	5.67E-02	5.00E-01
palmitoyl-sphingosine-phosphoethanolamine (d18:1/16:0)	-9.17E-02	6.32E-02	1.48E-01	-8.01E-02	6.12E-02	1.92E-01	-6.12E-03	5.97E-02	9.18E-01
picolinoylglycine	8.76E-02	5.83E-02	1.34E-01	1.49E-01	5.59E-02	8.06E-03	3.84E-02	5.50E-02	4.86E-01

4-vinylcatechol sulfate	1.16E-01	5.88E-02	5.00E-02	5.36E-02	5.72E-02	3.50E-01	7.20E-02	5.55E-02	1.96E-01
succinoyltaurine	3.01E-02	6.54E-02	6.46E-01	1.14E-01	6.30E-02	7.23E-02	-1.27E-02	6.15E-02	8.36E-01
phenylalanylhydroxyproline*	-1.33E-01	6.51E-02	4.14E-02	-5.48E-02	6.34E-02	3.88E-01	9.90E-04	6.16E-02	9.87E-01
ginkgolic acid C15:1	-1.77E-02	6.92E-02	7.98E-01	1.07E-02	6.70E-02	8.73E-01	-6.21E-02	6.49E-02	3.40E-01
(15:3)-anacardic acid	4.61E-02	6.55E-02	4.83E-01	3.80E-02	6.34E-02	5.50E-01	1.38E-03	6.17E-02	9.82E-01
ginkgolic acid C17:1	5.31E-02	6.23E-02	3.95E-01	8.14E-02	6.02E-02	1.77E-01	1.05E-03	5.87E-02	9.86E-01
3,5-dichloro-2,6-dihydroxybenzoic acid	-2.54E-01	6.16E-02	5.11E-05	-1.43E-01	6.09E-02	1.93E-02	-2.22E-01	5.82E-02	1.72E-04
metabolonic lactone sulfate	-7.77E-02	5.56E-02	1.64E-01	-1.02E-01	5.37E-02	5.89E-02	-9.44E-02	5.22E-02	7.14E-02
vanillic acid glycine	1.27E-01	6.13E-02	3.89E-02	1.96E-01	5.87E-02	9.62E-04	1.27E-01	5.76E-02	2.86E-02
2-hydroxy-4-(methylthio)butanoic acid	-1.19E-01	5.53E-02	3.25E-02	-8.83E-02	5.37E-02	1.01E-01	3.95E-03	5.24E-02	9.40E-01
branched chain 14:0 dicarboxylic acid**	3.52E-01	5.72E-02	2.77E-09	2.80E-01	5.66E-02	1.24E-06	2.20E-01	5.58E-02	1.00E-04
(2-butoxyethoxy)acetic acid	1.24E-02	6.72E-02	8.54E-01	6.15E-02	6.50E-02	3.44E-01	-7.12E-02	6.30E-02	2.60E-01
pentose acid*	2.78E-01	5.47E-02	7.08E-07	1.30E-01	5.48E-02	1.88E-02	1.86E-01	5.26E-02	4.65E-04
N-succinyl-phenylalanine	5.12E-02	6.61E-02	4.39E-01	3.57E-03	6.40E-02	9.56E-01	-9.35E-03	6.22E-02	8.81E-01
1-methyl-5-imidazolelactate	-1.79E-02	6.39E-02	7.80E-01	-6.25E-02	6.18E-02	3.12E-01	-1.61E-02	6.01E-02	7.88E-01
(2 or 3)-decenoate (10:1n7 or n8)	-8.94E-02	6.32E-02	1.58E-01	-9.70E-02	6.11E-02	1.13E-01	-6.34E-02	5.95E-02	2.87E-01
chenodeoxycholic acid sulfate (1)	-6.53E-02	6.38E-02	3.07E-01	-4.82E-02	6.18E-02	4.36E-01	-3.59E-02	6.00E-02	5.51E-01
branched-chain, straight-chain, or cyclopropyl 10:1 fatty acid (1)*	-1.69E-01	6.24E-02	7.25E-03	-2.11E-01	5.99E-02	5.09E-04	-1.32E-01	5.89E-02	2.55E-02
branched-chain, straight-chain, or cyclopropyl 10:1 fatty acid (2)*	-1.89E-01	6.55E-02	4.15E-03	-1.91E-01	6.33E-02	2.75E-03	-1.52E-01	6.18E-02	1.47E-02
carnitine of C10H14O2 (5)*	-9.03E-02	6.49E-02	1.65E-01	8.85E-03	6.31E-02	8.88E-01	-5.04E-02	6.12E-02	4.11E-01
3-bromo-5-chloro-2,6-dihydroxybenzoic acid*	-2.21E-01	6.33E-02	5.59E-04	-1.94E-01	6.15E-02	1.73E-03	-2.75E-01	5.85E-02	4.15E-06
branched-chain, straight-chain, or cyclopropyl 12:1 fatty acid*	-1.56E-01	6.23E-02	1.28E-02	-1.53E-01	6.02E-02	1.19E-02	-1.20E-01	5.88E-02	4.19E-02
decadienedioic acid (C10:2-DC)**	-9.81E-02	6.32E-02	1.21E-01	-1.16E-01	6.10E-02	5.80E-02	-1.21E-01	5.92E-02	4.18E-02
deoxycholic acid 12-sulfate*	-4.56E-02	6.40E-02	4.77E-01	-3.68E-02	6.19E-02	5.53E-01	-7.03E-02	6.00E-02	2.43E-01
GlcNAc sulfate conjugate of C21H34O2 steroid**	-3.00E-02	6.45E-02	6.42E-01	-6.74E-02	6.23E-02	2.80E-01	-7.61E-02	6.05E-02	2.09E-01
cis-3,4-methyleneheptanoate	-1.76E-01	6.42E-02	6.46E-03	-4.99E-02	6.29E-02	4.28E-01	-9.76E-02	6.09E-02	1.10E-01
cis-3,4-methyleneheptanoylcarnitine	-2.05E-01	6.33E-02	1.32E-03	-6.98E-02	6.23E-02	2.64E-01	-1.05E-01	6.03E-02	8.18E-02
N-acetyl-2-aminoadipate	-1.09E-01	5.82E-02	6.25E-02	-7.69E-02	5.65E-02	1.75E-01	-5.31E-02	5.50E-02	3.35E-01
De(carboxymethoxy) cetirizine acetic acid	8.42E-02	6.39E-02	1.89E-01	5.73E-02	6.19E-02	3.55E-01	1.41E-02	6.02E-02	8.16E-01
4-chlorobenzoic acid	-4.41E-02	6.35E-02	4.88E-01	-3.85E-02	6.15E-02	5.32E-01	1.03E-02	5.98E-02	8.64E-01
torasemide	-5.38E-02	7.24E-02	4.58E-01	-9.02E-02	7.00E-02	1.99E-01	-1.27E-01	6.78E-02	6.21E-02
2-methoxyhydroquinone sulfate (1)	-4.90E-03	6.37E-02	9.39E-01	4.58E-02	6.16E-02	4.58E-01	5.26E-02	5.98E-02	3.80E-01
diazepam	4.51E-02	7.23E-02	5.33E-01	8.38E-02	6.98E-02	2.31E-01	9.43E-02	6.78E-02	1.65E-01
temazepam	1.26E-01	7.21E-02	8.24E-02	7.17E-02	7.00E-02	3.06E-01	1.66E-01	6.74E-02	1.42E-02
2,4-di-tert-butylphenol	-1.00E-01	6.63E-02	1.33E-01	-1.43E-01	6.39E-02	2.59E-02	-6.34E-02	6.25E-02	3.11E-01
3-hydroxyoctanoylcarnitine (1)	-1.63E-02	6.05E-02	7.87E-01	2.43E-02	5.85E-02	6.78E-01	-3.92E-02	5.68E-02	4.91E-01
3-hydroxyoctanoylcarnitine (2)	-5.22E-03	6.03E-02	9.31E-01	3.10E-03	5.83E-02	9.58E-01	-9.87E-03	5.67E-02	8.62E-01
cis-3,4-methyleneheptanoylglycine	-2.17E-01	6.37E-02	7.48E-04	-1.29E-01	6.24E-02	3.90E-02	-1.59E-01	6.04E-02	9.12E-03
bilirubin degradation product, C16H18N2O5 (1)**	-7.02E-02	6.31E-02	2.66E-01	-1.11E-01	6.08E-02	6.95E-02	-2.42E-02	5.94E-02	6.85E-01
bilirubin degradation product, C16H18N2O5 (2)**	-6.01E-02	6.19E-02	3.32E-01	-9.35E-02	5.97E-02	1.18E-01	1.52E-03	5.83E-02	9.79E-01
bilirubin degradation product, C17H18N2O4 (1)**	-8.36E-02	6.32E-02	1.87E-01	-1.05E-01	6.11E-02	8.53E-02	-1.36E-02	5.96E-02	8.20E-01
bilirubin degradation product, C17H18N2O4 (2)**	-7.43E-02	6.23E-02	2.34E-01	-1.03E-01	6.01E-02	8.64E-02	-1.06E-02	5.87E-02	8.56E-01
bilirubin degradation product, C17H18N2O4 (3)**	-8.87E-02	6.26E-02	1.58E-01	-1.14E-01	6.04E-02	5.96E-02	-1.51E-02	5.91E-02	7.98E-01
bilirubin degradation product, C17H20N2O5 (1)**	-9.58E-02	6.40E-02	1.36E-01	-1.17E-01	6.18E-02	5.99E-02	-2.93E-02	6.04E-02	6.28E-01
bilirubin degradation product, C17H20N2O5 (2)**	-1.01E-01	6.39E-02	1.15E-01	-1.21E-01	6.17E-02	5.05E-02	-1.88E-02	6.04E-02	7.56E-01
tetrahydrocortisol glucuronide	-2.47E-02	6.07E-02	6.84E-01	-1.10E-01	5.84E-02	6.06E-02	-5.49E-02	5.69E-02	3.36E-01
bilirubin degradation product, C16H18N2O5 (3)**	-7.44E-02	6.15E-02	2.28E-01	-8.01E-02	5.95E-02	1.79E-01	8.35E-03	5.79E-02	8.86E-01
bilirubin degradation product, C16H18N2O5 (4)**	-8.99E-02	6.10E-02	1.42E-01	-9.56E-02	5.90E-02	1.07E-01	-3.01E-03	5.76E-02	9.58E-01
N,N-dimethyl-pro-pro	-1.02E-01	6.13E-02	9.63E-02	1.06E-02	5.96E-02	8.59E-01	-1.08E-01	5.76E-02	6.05E-02
oxindolylalanine	1.85E-01	6.46E-02	4.51E-03	1.93E-01	6.24E-02	2.14E-03	1.21E-01	6.12E-02	4.82E-02
tetrahydrocortisone glucuronide (5)	-2.27E-02	5.86E-02	6.99E-01	-3.83E-02	5.67E-02	5.00E-01	-8.54E-02	5.49E-02	1.21E-01
perfluorohexanesulfonic acid	5.71E-02	5.58E-02	3.07E-01	7.46E-03	5.41E-02	8.91E-01	3.98E-03	5.26E-02	9.40E-01
trans-4-hydroxyproline	-1.47E-01	6.10E-02	1.68E-02	-1.28E-01	5.92E-02	3.13E-02	-1.09E-01	5.76E-02	5.89E-02
allantoin	1.19E-01	6.29E-02	5.98E-02	1.80E-01	6.03E-02	3.04E-03	9.56E-02	5.92E-02	1.08E-01
xanthine	-1.54E-01	5.99E-02	1.07E-02	-1.20E-02	5.86E-02	8.38E-01	-1.20E-01	5.65E-02	3.49E-02
5-oxoproline	2.05E-02	6.27E-02	7.44E-01	5.06E-02	6.06E-02	4.04E-01	4.63E-03	5.89E-02	9.37E-01
sarcosine	-8.56E-02	6.60E-02	1.96E-01	-8.19E-02	6.39E-02	2.01E-01	-1.09E-01	6.19E-02	8.01E-02
pantothenate	1.24E-01	5.95E-02	3.79E-02	1.50E-01	5.73E-02	9.38E-03	4.57E-02	5.63E-02	4.17E-01
pipecolate	1.14E-01	6.42E-02	7.64E-02	5.20E-02	6.24E-02	4.05E-01	1.29E-01	6.02E-02	3.26E-02
phosphoethanolamine	2.71E-02	6.76E-02	6.89E-01	7.82E-02	6.53E-02	2.32E-01	5.55E-02	6.35E-02	3.83E-01
glycerate	2.80E-01	5.76E-02	1.95E-06	2.70E-01	5.58E-02	2.18E-06	1.96E-01	5.52E-02	4.60E-04
3-ureidopropionate	1.93E-01	5.83E-02	1.08E-03	1.24E-01	5.71E-02	3.03E-02	1.47E-01	5.52E-02	8.10E-03
5-KETE	-1.45E-02	5.90E-02	8.06E-01	-2.33E-02	5.71E-02	6.83E-01	5.48E-03	5.55E-02	9.21E-01
N-acetylleucine	-5.83E-02	5.69E-02	3.06E-01	-3.56E-02	5.51E-02	5.19E-01	4.65E-03	5.36E-02	9.31E-01
N-acetylmethionine	-1.25E-01	6.02E-02	3.80E-02	5.73E-02	5.86E-02	3.29E-01	-1.15E-01	5.66E-02	4.26E-02
N-acetylvaline	4.81E-02	6.14E-02	4.34E-01	8.46E-02	5.93E-02	1.55E-01	2.44E-02	5.78E-02	6.73E-01
erucate (22:1n9)	-1.49E-02	6.38E-02	8.15E-01	-8.36E-02	6.15E-02	1.75E-01	-1.65E-02	5.99E-02	7.83E-01
bilirubin (Z,Z)	-8.00E-03	6.14E-02	8.96E-01	-5.19E-02	5.93E-02	3.82E-01	4.05E-02	5.77E-02	4.83E-01
thyroxine	-9.53E-03	6.36E-02	8.81E-01	7.22E-02	6.14E-02	2.40E-01	-4.30E-02	5.97E-02	4.73E-01
gamma-glutamyltyrosine	2.52E-02	5.76E-02	6.62E-01	5.07E-02	5.57E-02	3.64E-01	6.48E-03	5.42E-02	9.05E-01
alpha-tocopherol	1.48E-01	6.09E-02	1.59E-02	-3.66E-04	5.95E-02	9.95E-01	7.52E-02	5.77E-02	1.93E-01
3-hydroxyisobutyrate	-1.97E-02	6.24E-02	7.52E-01	3.24E-03	6.04E-02	9.57E-01	-5.83E-02	5.86E-02	3.20E-01
N-acetylalanine	-3.40E-02	6.24E-02	5.86E-01	1.40E-01	5.98E-02	1.96E-02	-5.10E-02	5.86E-02	3.85E-01
vanillylmandelate (VMA)	7.88E-02	5.89E-02	1.82E-01	1.24E-01	5.67E-02	2.98E-02	1.67E-02	5.55E-02	7.64E-01
4-acetamidobutanoate	1.88E-02	5.99E-02	7.54E-01	1.14E-01	5.76E-02	4.91E-02	-5.90E-03	5.64E-02	9.17E-01
3-aminoisobutyrate	-6.65E-02	6.26E-02	2.89E-01	-8.18E-02	6.05E-02	1.78E-01	-5.05E-02	5.89E-02	3.92E-01
3-hydroxy-3-methylglutarate	6.87E-02	5.74E-02	2.32E-01	1.30E-01	5.52E-02	1.89E-02	6.93E-02	5.40E-02	2.00E-01
citrate	-8.13E-02	6.06E-02	1.81E-01	2.73E-02	5.88E-02	6.43E-01	-6.69E-02	5.70E-02	2.42E-01
2-aminobutyrate	-7.39E-02	6.37E-02	2.47E-01	-2.16E-01	6.04E-02	4.16E-04	-5.96E-03	6.00E-02	9.21E-01
urate	-9.03E-03	5.20E-02	8.62E-01	3.22E-02	5.03E-02	5.23E-01	1.01E-02	4.89E-02	8.37E-01
ursodeoxycholate	-1.62E-01	6.39E-02	1.21E-02	-1.54E-01	6.19E-02	1.34E-02	-2.31E-02	6.08E-02	7.04E-01
oleoyl ethanolamide	-5.10E-02	6.23E-02	4.13E-01	-8.62E-02	6.01E-02	1.53E-01	-5.48E-03	5.86E-02	9.26E-01

Supplementary Table 4: Parameter estimates for metabolome-wide association studies for diet-metabolite associations for each of: HEI-15, DASH and AMED diet, controlling for BMI

gamma-glutamylglutamine	-4.91E-02	6.15E-02	4.26E-01	2.41E-03	5.96E-02	9.68E-01	-3.64E-02	5.79E-02	5.30E-01
4-hydroxyphenylpyruvate	9.43E-03	6.35E-02	8.82E-01	4.19E-02	6.14E-02	4.95E-01	-1.89E-02	5.97E-02	7.52E-01
butyrate/isobutyrate (4:0)	3.41E-02	6.37E-02	5.92E-01	2.11E-02	6.17E-02	7.32E-01	7.56E-02	5.97E-02	2.07E-01
N-acetylneuraminate	-1.21E-02	6.47E-02	8.52E-01	7.50E-02	6.24E-02	2.30E-01	-9.45E-03	6.08E-02	8.77E-01
homovanillate (HVA)	7.37E-02	6.24E-02	2.39E-01	4.49E-02	6.05E-02	4.59E-01	3.35E-02	5.88E-02	5.69E-01
creatine	-1.10E-01	5.93E-02	6.54E-02	-1.98E-01	5.65E-02	5.29E-04	-2.71E-02	5.61E-02	6.29E-01
cys-gly, oxidized	-1.52E-01	6.03E-02	1.21E-02	-6.81E-02	5.89E-02	2.49E-01	-1.42E-01	5.67E-02	1.27E-02
2,3-diphosphoglycerate	-6.65E-02	6.51E-02	3.08E-01	-5.39E-03	6.31E-02	9.32E-01	-3.11E-02	6.13E-02	6.12E-01
estrone 3-sulfate	-2.17E-02	6.46E-02	7.37E-01	-1.51E-01	6.19E-02	1.51E-02	-8.20E-02	6.06E-02	1.77E-01
dihomo-linoleate (20:2n6)	-1.49E-01	6.23E-02	1.77E-02	-1.69E-01	6.01E-02	5.36E-03	-1.04E-01	5.89E-02	7.98E-02
gamma-glutamylhistidine	8.97E-02	6.50E-02	1.69E-01	6.69E-02	6.30E-02	2.89E-01	8.62E-02	6.11E-02	1.60E-01
2-hydroxystearate	-1.14E-01	6.54E-02	8.16E-02	-1.32E-01	6.31E-02	3.74E-02	-1.20E-01	6.14E-02	5.23E-02
N1-methyladenosine	-2.60E-02	6.09E-02	6.70E-01	5.86E-02	5.89E-02	3.20E-01	-7.39E-02	5.71E-02	1.97E-01
glycerol	-1.37E-01	5.34E-02	1.09E-02	-1.39E-01	5.16E-02	7.65E-03	-1.02E-01	5.04E-02	4.38E-02
choline	-1.05E-02	6.38E-02	8.70E-01	6.10E-02	6.17E-02	3.23E-01	1.28E-02	6.00E-02	8.31E-01
anthranilate	6.06E-02	6.48E-02	3.50E-01	-6.61E-02	6.27E-02	2.92E-01	7.12E-03	6.10E-02	9.07E-01
gamma-glutamylleucine	-6.36E-02	5.78E-02	2.72E-01	2.82E-03	5.61E-02	9.60E-01	-5.07E-02	5.44E-02	3.52E-01
3-phosphoglycerate	-1.80E-02	6.53E-02	7.82E-01	4.75E-03	6.32E-02	9.40E-01	-3.72E-02	6.13E-02	5.44E-01
3-methoxytyrosine	-4.15E-02	6.14E-02	4.99E-01	5.65E-02	5.94E-02	3.42E-01	-6.62E-02	5.76E-02	2.52E-01
cholate	1.49E-02	6.50E-02	8.19E-01	1.54E-02	6.29E-02	8.06E-01	1.11E-01	6.08E-02	6.98E-02
fluoxetine	9.50E-03	6.99E-02	8.92E-01	-5.53E-02	6.76E-02	4.14E-01	4.81E-02	6.57E-02	4.64E-01
4-acetamidophenol	6.30E-02	6.67E-02	3.45E-01	1.27E-01	6.42E-02	4.88E-02	7.31E-02	6.26E-02	2.44E-01
naproxen	5.02E-02	6.54E-02	4.44E-01	1.14E-01	6.30E-02	7.19E-02	4.98E-02	6.15E-02	4.19E-01
topiramate	-2.57E-02	5.46E-02	6.38E-01	-2.07E-02	5.28E-02	6.95E-01	4.23E-02	5.13E-02	4.10E-01
beta-hydroxyisovalerate	-8.49E-02	5.82E-02	1.46E-01	-8.29E-02	5.64E-02	1.43E-01	-1.22E-02	5.50E-02	8.24E-01
ibuprofen	4.41E-02	6.44E-02	4.95E-01	-7.92E-02	6.22E-02	2.04E-01	-6.40E-03	6.06E-02	9.16E-01
arachidonoyl ethanolamide	-8.16E-02	6.10E-02	1.82E-01	-8.50E-02	5.90E-02	1.51E-01	-7.57E-03	5.76E-02	8.95E-01
palmitoyl ethanolamide	-1.16E-01	6.23E-02	6.31E-02	-1.30E-01	6.02E-02	3.11E-02	-1.02E-01	5.86E-02	8.37E-02
N-linoleoylglycine	-1.07E-02	6.58E-02	8.70E-01	6.15E-02	6.35E-02	3.34E-01	4.00E-02	6.18E-02	5.18E-01
N-palmitoyl-sphingosine (d18:1/16:0)	-3.19E-02	6.38E-02	6.17E-01	-8.78E-02	6.15E-02	1.54E-01	-5.32E-02	5.99E-02	3.75E-01
1-palmitoyl-2-oleoyl-GPE (16:0/18:1)	8.39E-02	6.48E-02	1.96E-01	-1.97E-02	6.29E-02	7.54E-01	4.44E-02	6.10E-02	4.68E-01
1-palmitoyl-2-linoleoyl-GPI (16:0/18:2)	-2.73E-02	6.46E-02	6.73E-01	-7.59E-02	6.24E-02	2.25E-01	6.02E-03	6.07E-02	9.21E-01
1-palmitoyl-2-linoleoyl-GPC (16:0/18:2)	-1.06E-01	6.37E-02	9.67E-02	-1.82E-01	6.10E-02	3.12E-03	-3.87E-02	6.01E-02	5.20E-01
stearoyl sphingomyelin (d18:1/18:0)	-2.09E-01	6.07E-02	6.83E-04	-2.02E-01	5.88E-02	6.79E-04	-1.85E-01	5.72E-02	1.38E-03
1-palmitoyl-2-oleoyl-GPC (16:0/18:1)	-6.00E-02	6.42E-02	3.51E-01	-2.09E-01	6.10E-02	6.98E-04	-3.98E-02	6.05E-02	5.11E-01
N-stearoyl-sphingosine (d18:1/18:0)*	-1.28E-01	6.20E-02	4.06E-02	-1.51E-01	5.98E-02	1.22E-02	-1.52E-01	5.80E-02	9.39E-03
5,6-dihydrothymine	-6.67E-02	6.21E-02	2.84E-01	-5.82E-02	6.01E-02	3.34E-01	-1.31E-01	5.80E-02	2.44E-02
glycochenodeoxycholate	1.83E-02	6.49E-02	7.78E-01	-5.47E-03	6.28E-02	9.31E-01	-5.60E-02	6.09E-02	3.59E-01
taurochenodeoxycholate	-1.59E-02	6.55E-02	8.08E-01	-3.24E-02	6.34E-02	6.09E-01	-6.91E-02	6.14E-02	2.62E-01
taurocholate	6.96E-03	6.52E-02	9.15E-01	1.89E-02	6.31E-02	7.65E-01	-2.44E-02	6.12E-02	6.91E-01
taurodeoxycholate	9.53E-03	6.59E-02	8.85E-01	1.32E-02	6.38E-02	8.36E-01	-9.64E-02	6.17E-02	1.19E-01
hypoxanthine	-5.87E-02	6.65E-02	3.78E-01	1.47E-02	6.44E-02	8.20E-01	-4.28E-03	6.26E-02	9.46E-01
9,10-DiHOME	5.46E-02	6.44E-02	3.97E-01	1.77E-01	6.15E-02	4.36E-03	6.02E-02	6.05E-02	3.21E-01
linoleate (18:2n6)	-1.18E-01	6.08E-02	5.42E-02	-1.14E-01	5.89E-02	5.37E-02	-7.95E-02	5.74E-02	1.67E-01
laurate (12:0)	-1.19E-01	6.09E-02	5.22E-02	-2.60E-02	5.94E-02	6.61E-01	-7.54E-02	5.75E-02	1.91E-01
quinolinate	-5.70E-02	6.09E-02	3.50E-01	4.70E-02	5.90E-02	4.26E-01	-1.51E-01	5.67E-02	7.94E-03
2-hydroxyhippurate (salicylurate)	1.23E-01	6.55E-02	6.16E-02	1.98E-01	6.26E-02	1.73E-03	4.89E-02	6.19E-02	4.30E-01
N6,N6,N6-trimethyllysine	-8.36E-02	6.14E-02	1.74E-01	-9.33E-04	5.96E-02	9.88E-01	-5.40E-02	5.78E-02	3.51E-01
N-acetylputrescine	-6.77E-03	6.28E-02	9.14E-01	-4.37E-02	6.08E-02	4.73E-01	-3.24E-02	5.90E-02	5.84E-01
N-formylmethionine	-6.99E-02	6.14E-02	2.56E-01	7.04E-02	5.94E-02	2.37E-01	-1.04E-01	5.75E-02	7.19E-02
S-adenosylhomocysteine (SAH)	4.21E-02	6.14E-02	4.94E-01	1.23E-01	5.90E-02	3.73E-02	-4.12E-02	5.77E-02	4.76E-01
metoprolol	7.03E-02	6.82E-02	3.04E-01	1.41E-01	6.56E-02	3.21E-02	9.49E-02	6.40E-02	1.39E-01
azelate (C9-DC)	1.48E-02	6.48E-02	8.20E-01	9.45E-02	6.25E-02	1.32E-01	-6.41E-02	6.08E-02	2.93E-01
3-(N-acetyl-L-cystein-S-yl) acetaminophen	9.35E-02	6.42E-02	1.47E-01	1.41E-01	6.18E-02	2.29E-02	5.02E-02	6.05E-02	4.08E-01
4-acetaminophen sulfate	1.11E-01	6.58E-02	9.19E-02	1.66E-01	6.32E-02	9.12E-03	8.43E-02	6.19E-02	1.74E-01
eicosapentaenoate (EPA; 20:5n3)	-8.15E-03	6.30E-02	8.97E-01	-9.43E-02	6.07E-02	1.21E-01	6.23E-02	5.91E-02	2.93E-01
methylsuccinate	2.07E-01	6.11E-02	7.93E-04	1.73E-01	5.95E-02	3.86E-03	1.65E-01	5.78E-02	4.68E-03
ethylmalonate	-1.45E-02	6.03E-02	8.09E-01	-1.32E-02	5.83E-02	8.21E-01	-2.13E-02	5.66E-02	7.07E-01
adenosine 3',5'-cyclic monophosphate (cAMP)	6.23E-03	6.41E-02	9.23E-01	7.86E-02	6.19E-02	2.05E-01	-3.86E-02	6.02E-02	5.22E-01
adenosine 5'-monophosphate (AMP)	-7.05E-03	6.40E-02	9.12E-01	6.92E-02	6.18E-02	2.64E-01	1.57E-02	6.02E-02	7.94E-01
5-methylthioadenosine (MTA)	-3.29E-02	6.19E-02	5.95E-01	2.75E-02	5.99E-02	6.46E-01	-2.77E-02	5.82E-02	6.34E-01
N6-methyladenosine	-7.30E-02	6.25E-02	2.44E-01	1.66E-03	6.06E-02	9.78E-01	-9.36E-02	5.86E-02	1.11E-01
arachidonate (20:4n6)	-1.41E-01	6.55E-02	3.27E-02	-1.91E-01	6.29E-02	2.67E-03	-1.09E-01	6.18E-02	7.93E-02
arginine	6.64E-02	6.79E-02	3.29E-01	7.23E-02	6.57E-02	2.72E-01	6.53E-02	6.38E-02	3.07E-01
aspartate	6.40E-02	6.09E-02	2.94E-01	3.66E-02	5.90E-02	5.36E-01	4.07E-02	5.73E-02	4.79E-01
2-hydroxyphenylacetate	4.91E-02	6.35E-02	4.40E-01	1.10E-01	6.11E-02	7.32E-02	5.96E-02	5.96E-02	3.18E-01
3-(4-hydroxyphenyl)lactate	-1.77E-02	5.30E-02	7.38E-01	2.41E-03	5.13E-02	9.63E-01	3.44E-02	4.98E-02	4.90E-01
phenylpyruvate	-2.50E-04	6.28E-02	9.97E-01	9.30E-02	6.06E-02	1.26E-01	-2.90E-03	5.91E-02	9.61E-01
beta-alanine	2.11E-02	6.32E-02	7.39E-01	9.18E-02	6.09E-02	1.33E-01	-1.54E-02	5.94E-02	7.95E-01
biliverdin	-8.98E-02	6.32E-02	1.56E-01	-8.42E-02	6.11E-02	1.70E-01	-4.56E-02	5.95E-02	4.45E-01
succinate	5.81E-02	6.30E-02	3.58E-01	4.24E-02	6.11E-02	4.88E-01	7.61E-02	5.92E-02	2.00E-01
3-hydroxybutyrate (BHBA)	-4.23E-02	6.06E-02	4.86E-01	-7.85E-02	5.86E-02	1.81E-01	8.02E-03	5.71E-02	8.88E-01
cholesterol	2.77E-02	6.37E-02	6.64E-01	-5.86E-02	6.16E-02	3.42E-01	3.98E-02	5.98E-02	5.07E-01
corticosterone	-1.31E-03	6.05E-02	9.83E-01	-8.82E-02	5.83E-02	1.32E-01	-5.15E-02	5.68E-02	3.65E-01
cortisone	-3.02E-02	6.35E-02	6.35E-01	-1.47E-01	6.09E-02	1.65E-02	-4.22E-02	5.97E-02	4.80E-01
creatinine	-4.79E-02	5.36E-02	3.72E-01	4.04E-02	5.19E-02	4.37E-01	-6.36E-02	5.03E-02	2.07E-01
cysteinylglycine	-1.34E-01	6.00E-02	2.62E-02	-8.99E-02	5.84E-02	1.25E-01	-8.80E-02	5.67E-02	1.22E-01
cystine	-2.49E-01	6.01E-02	4.51E-05	-2.35E-01	5.83E-02	7.10E-05	-1.79E-01	5.72E-02	1.90E-03
sphingosine	-1.03E-01	6.45E-02	1.10E-01	-7.76E-02	6.25E-02	2.15E-01	-6.84E-02	6.08E-02	2.62E-01
deoxycholate	-7.14E-02	6.31E-02	2.59E-01	-9.14E-02	6.09E-02	1.35E-01	-1.90E-02	5.94E-02	7.50E-01
cystathionine	9.48E-02	6.63E-02	1.54E-01	1.54E-01	6.38E-02	1.62E-02	8.36E-02	6.24E-02	1.81E-01
sphinganine	-7.18E-02	6.60E-02	2.77E-01	-5.36E-02	6.39E-02	4.02E-01	-8.49E-02	6.19E-02	1.72E-01

Supplementary Table 4: Parameter estimates for metabolome-wide association studies for diet-metabolite associations for each of: HEI-15, DASH and AMED diet, controlling for BMI

flavin adenine dinucleotide (FAD)	7.31E-02	6.68E-02	2.75E-01	2.63E-02	6.48E-02	6.85E-01	6.32E-02	6.28E-02	3.15E-01
fumarate	-4.24E-02	6.07E-02	4.86E-01	-1.35E-02	5.88E-02	8.19E-01	5.96E-03	5.72E-02	9.17E-01
gamma-glutamylglutamate	-5.33E-02	5.95E-02	3.72E-01	-1.38E-02	5.77E-02	8.11E-01	-4.57E-02	5.60E-02	4.14E-01
gluconate	-2.39E-02	6.16E-02	6.98E-01	7.80E-02	5.95E-02	1.91E-01	3.81E-03	5.79E-02	9.48E-01
glutarate (C5-DC)	-1.56E-01	6.00E-02	9.73E-03	-6.30E-02	5.86E-02	2.84E-01	7.10E-02	5.69E-02	2.13E-01
glycine	-1.15E-01	6.20E-02	6.52E-02	9.45E-03	6.04E-02	8.76E-01	-1.00E-01	5.84E-02	8.68E-02
glycocholate	2.42E-02	6.47E-02	7.08E-01	4.31E-02	6.26E-02	4.92E-01	-1.74E-02	6.08E-02	7.75E-01
guanidinoacetate	1.31E-02	6.20E-02	8.34E-01	3.18E-02	6.00E-02	5.96E-01	2.26E-02	5.83E-02	6.99E-01
S-1-pyrroline-5-carboxylate	-9.17E-03	6.12E-02	8.81E-01	3.72E-02	5.92E-02	5.30E-01	-5.63E-04	5.75E-02	9.92E-01
histidine	8.84E-02	6.24E-02	1.58E-01	-2.49E-06	6.06E-02	1.00E+00	5.96E-02	5.87E-02	3.11E-01
cortisol	-1.30E-02	6.39E-02	8.39E-01	-1.08E-01	6.16E-02	8.17E-02	-6.57E-02	6.00E-02	2.74E-01
hypotaurine	-1.48E-03	5.74E-02	9.79E-01	6.10E-02	5.54E-02	2.72E-01	3.38E-02	5.39E-02	5.31E-01
inosine	-5.15E-03	6.37E-02	9.36E-01	3.89E-02	6.16E-02	5.29E-01	3.16E-02	5.99E-02	5.98E-01
myo-inositol	2.32E-01	5.90E-02	1.04E-04	1.42E-01	5.80E-02	1.49E-02	1.85E-01	5.59E-02	1.02E-03
isoleucine	-7.44E-02	5.42E-02	1.71E-01	-1.03E-02	5.27E-02	8.45E-01	-4.29E-02	5.11E-02	4.02E-01
2-aminoadipate	-8.54E-03	6.19E-02	8.90E-01	-1.65E-02	5.99E-02	7.83E-01	1.33E-02	5.82E-02	8.19E-01
citrulline	-5.93E-02	6.45E-02	3.58E-01	8.40E-03	6.25E-02	8.93E-01	4.10E-02	6.07E-02	5.00E-01
leucine	-2.54E-02	5.49E-02	6.44E-01	-1.43E-02	5.32E-02	7.89E-01	-4.31E-02	5.16E-02	4.05E-01
lithocholate	-3.83E-03	6.46E-02	9.53E-01	5.18E-02	6.25E-02	4.08E-01	-2.03E-02	6.08E-02	7.38E-01
lysine	-6.22E-02	6.40E-02	3.32E-01	-3.74E-02	6.20E-02	5.47E-01	-1.11E-01	5.99E-02	6.43E-02
malate	-6.24E-02	6.28E-02	3.22E-01	1.14E-02	6.09E-02	8.52E-01	-1.83E-02	5.92E-02	7.57E-01
methionine	-1.21E-01	5.98E-02	4.30E-02	-2.91E-02	5.82E-02	6.18E-01	-1.04E-01	5.62E-02	6.48E-02
methylmalonate (MMA)	-6.53E-02	6.23E-02	2.95E-01	3.93E-02	6.04E-02	5.15E-01	1.27E-03	5.87E-02	9.83E-01
palmitate (16:0)	-1.90E-01	6.15E-02	2.19E-03	-1.93E-01	5.94E-02	1.28E-03	-1.63E-01	5.80E-02	5.29E-03
nicotinamide	2.50E-02	6.51E-02	7.02E-01	1.38E-01	6.25E-02	2.76E-02	2.63E-02	6.12E-02	6.68E-01
stearate (18:0)	-1.76E-01	6.34E-02	6.00E-03	-1.34E-01	6.17E-02	3.12E-02	-1.35E-01	5.99E-02	2.53E-02
ornithine	-3.07E-02	6.47E-02	6.35E-01	3.13E-02	6.26E-02	6.17E-01	3.28E-02	6.08E-02	5.91E-01
orotate	4.67E-03	6.36E-02	9.42E-01	-3.73E-03	6.15E-02	9.52E-01	-1.83E-02	5.97E-02	7.60E-01
palmitoleate (16:1n7)	-1.70E-01	5.60E-02	2.60E-03	-2.03E-01	5.37E-02	1.90E-04	-1.27E-01	5.29E-02	1.74E-02
phenylalanine	-1.57E-02	6.25E-02	8.02E-01	6.74E-03	6.05E-02	9.11E-01	-4.81E-02	5.87E-02	4.14E-01
phosphate	-2.83E-02	6.43E-02	6.60E-01	1.68E-02	6.22E-02	7.88E-01	-6.98E-03	6.05E-02	9.08E-01
phytanate	-5.75E-02	5.92E-02	3.32E-01	-8.99E-02	5.71E-02	1.17E-01	-4.83E-02	5.57E-02	3.86E-01
proline	-1.71E-01	6.15E-02	5.68E-03	-5.27E-02	6.03E-02	3.82E-01	-1.14E-01	5.82E-02	5.09E-02
lactate	-1.94E-02	5.76E-02	7.36E-01	1.02E-02	5.58E-02	8.55E-01	1.16E-02	5.42E-02	8.30E-01
pyridoxal	1.60E-01	6.47E-02	1.40E-02	1.19E-01	6.29E-02	6.05E-02	9.27E-02	6.12E-02	1.31E-01
retinol (Vitamin A)	7.94E-02	6.20E-02	2.01E-01	3.58E-02	6.01E-02	5.52E-01	1.09E-02	5.84E-02	8.53E-01
spermidine	-1.95E-02	6.48E-02	7.64E-01	8.77E-02	6.25E-02	1.62E-01	-3.19E-02	6.09E-02	6.00E-01
salicylate	1.22E-01	6.68E-02	6.79E-02	2.01E-01	6.39E-02	1.85E-03	5.18E-02	6.31E-02	4.12E-01
serine	-1.46E-01	6.13E-02	1.79E-02	-1.45E-01	5.92E-02	1.49E-02	-6.84E-02	5.80E-02	2.39E-01
serotonin	7.18E-02	6.73E-02	2.87E-01	6.74E-02	6.52E-02	3.02E-01	5.71E-02	6.33E-02	3.68E-01
taurine	5.83E-02	6.73E-02	3.87E-01	3.33E-02	6.52E-02	6.10E-01	7.93E-02	6.32E-02	2.10E-01
myristate (14:0)	-2.10E-01	5.76E-02	3.13E-04	-1.65E-01	5.62E-02	3.69E-03	-1.71E-01	5.45E-02	1.85E-03
urea	5.58E-02	6.37E-02	3.82E-01	1.18E-01	6.14E-02	5.62E-02	6.30E-02	5.99E-02	2.94E-01
uridine	1.04E-01	6.40E-02	1.04E-01	1.13E-01	6.19E-02	6.92E-02	9.59E-02	6.02E-02	1.12E-01
2'-deoxyuridine	5.86E-03	6.60E-02	9.29E-01	1.20E-02	6.39E-02	8.51E-01	4.84E-03	6.21E-02	9.38E-01
trans-urocanate	-8.69E-02	6.35E-02	1.72E-01	-2.60E-02	6.16E-02	6.73E-01	-4.26E-02	5.98E-02	4.77E-01
1-methylnicotinamide	-6.87E-02	6.49E-02	2.91E-01	-4.65E-02	6.29E-02	4.60E-01	-1.17E-01	6.07E-02	5.50E-02
glutamate	-2.49E-02	5.65E-02	6.59E-01	-6.66E-03	5.47E-02	9.03E-01	-2.41E-02	5.31E-02	6.50E-01
glutamine	-3.35E-02	6.10E-02	5.84E-01	-2.20E-02	5.91E-02	7.09E-01	-4.32E-02	5.73E-02	4.52E-01
threonine	-1.76E-01	6.37E-02	6.04E-03	-9.21E-02	6.22E-02	1.40E-01	-8.93E-02	6.04E-02	1.41E-01
tryptophan	8.20E-02	5.95E-02	1.70E-01	6.93E-02	5.77E-02	2.30E-01	1.28E-02	5.62E-02	8.19E-01
valine	-1.88E-02	5.84E-02	7.47E-01	3.13E-02	5.65E-02	5.80E-01	1.03E-02	5.49E-02	8.51E-01
glucose	-1.25E-01	6.41E-02	5.22E-02	-1.45E-01	6.19E-02	1.97E-02	-1.06E-01	6.04E-02	7.92E-02
12,13-DiHOME	7.54E-02	6.08E-02	2.16E-01	1.48E-01	5.84E-02	1.16E-02	7.17E-02	5.72E-02	2.11E-01
alpha-ketobutyrate	-6.93E-02	6.32E-02	2.74E-01	-1.35E-01	6.08E-02	2.76E-02	-1.05E-02	5.95E-02	8.60E-01
betaine	1.38E-01	6.25E-02	2.78E-02	1.36E-01	6.05E-02	2.55E-02	1.56E-01	5.85E-02	8.02E-03
cysteine	-9.14E-02	6.16E-02	1.39E-01	-3.74E-02	5.98E-02	5.32E-01	-1.23E-01	5.77E-02	3.38E-02
mannose	-1.35E-01	5.90E-02	2.31E-02	-1.51E-01	5.69E-02	8.37E-03	-1.36E-01	5.54E-02	1.48E-02
dimethylglycine	7.46E-02	6.29E-02	2.37E-01	1.22E-01	6.06E-02	4.56E-02	6.66E-02	5.92E-02	2.62E-01
alanine	-1.53E-01	6.44E-02	1.82E-02	-1.20E-01	6.25E-02	5.63E-02	-1.32E-01	6.06E-02	2.99E-02
tyrosine	6.60E-02	5.93E-02	2.67E-01	9.30E-03	5.76E-02	8.72E-01	2.78E-02	5.59E-02	6.19E-01
pseudouridine	-1.86E-03	5.92E-02	9.75E-01	7.72E-02	5.71E-02	1.77E-01	-6.06E-02	5.55E-02	2.76E-01
pyruvate	-8.73E-02	6.12E-02	1.55E-01	-5.10E-02	5.93E-02	3.90E-01	-7.05E-02	5.75E-02	2.21E-01
uracil	8.53E-02	6.13E-02	1.65E-01	1.27E-01	5.90E-02	3.22E-02	1.71E-01	5.69E-02	2.87E-03
xylose	1.64E-01	6.66E-02	1.45E-02	1.19E-01	6.47E-02	6.76E-02	1.27E-01	6.28E-02	4.39E-02
cytidine	-3.25E-02	6.45E-02	6.15E-01	5.65E-02	6.24E-02	3.66E-01	3.02E-02	6.07E-02	6.19E-01
arabinose	7.71E-02	6.25E-02	2.19E-01	5.50E-02	6.06E-02	3.65E-01	1.05E-01	5.86E-02	7.47E-02
cotinine	-7.76E-02	4.68E-02	9.82E-02	-1.06E-01	4.50E-02	1.89E-02	-2.03E-02	4.42E-02	6.45E-01
caffeine	-1.19E-01	6.41E-02	6.56E-02	-1.23E-01	6.20E-02	4.81E-02	-7.69E-02	6.05E-02	2.05E-01
fructose	-1.23E-02	6.50E-02	8.50E-01	-4.62E-02	6.29E-02	4.63E-01	8.77E-03	6.11E-02	8.86E-01
adenine	-6.15E-02	6.40E-02	3.37E-01	-5.60E-02	6.19E-02	3.66E-01	-1.03E-01	5.99E-02	8.80E-02
cytosine	8.43E-02	6.48E-02	1.94E-01	3.52E-02	6.29E-02	5.76E-01	6.99E-02	6.10E-02	2.53E-01
caprate (10:0)	-1.31E-01	6.20E-02	3.49E-02	-1.15E-01	6.01E-02	5.68E-02	-5.65E-02	5.87E-02	3.36E-01
margarate (17:0)	-2.36E-01	6.27E-02	2.05E-04	-1.88E-01	6.12E-02	2.32E-03	-1.97E-01	5.92E-02	9.78E-04
nonadecanoate (19:0)	-1.29E-01	6.27E-02	4.12E-02	-9.22E-02	6.09E-02	1.31E-01	-1.05E-01	5.91E-02	7.63E-02
arachidate (20:0)	-2.44E-02	6.23E-02	6.96E-01	1.44E-02	6.04E-02	8.12E-01	-2.18E-02	5.86E-02	7.11E-01
maltose	1.71E-02	6.69E-02	7.99E-01	9.87E-02	6.45E-02	1.27E-01	4.04E-02	6.29E-02	5.21E-01
asparagine	1.87E-02	6.17E-02	7.63E-01	5.49E-02	5.96E-02	3.58E-01	3.75E-02	5.80E-02	5.18E-01
N-stearoyl-sphinganine (d18:0/18:0)*	-1.72E-01	6.13E-02	5.39E-03	-2.72E-01	5.79E-02	4.15E-06	-1.61E-01	5.76E-02	5.55E-03
dihydroorotate	3.37E-02	6.42E-02	6.00E-01	-2.58E-02	6.21E-02	6.78E-01	-4.79E-02	6.03E-02	4.28E-01
alpha-ketoglutarate	-9.79E-02	6.08E-02	1.09E-01	-3.24E-02	5.91E-02	5.84E-01	-5.56E-02	5.74E-02	3.33E-01
caprylate (8:0)	-6.49E-02	6.49E-02	3.18E-01	-1.09E-01	6.26E-02	8.12E-02	8.50E-03	6.11E-02	8.89E-01

sucrose	4.07E-02	5.95E-02	4.94E-01	1.70E-01	5.67E-02	3.01E-03	-5.05E-03	5.60E-02	9.28E-01
kynurenate	3.01E-02	5.91E-02	6.10E-01	4.04E-02	5.71E-02	4.80E-01	-5.20E-02	5.55E-02	3.50E-01
pentadecanoate (15:0)	-2.17E-01	5.81E-02	2.35E-04	-1.54E-01	5.69E-02	7.36E-03	-1.95E-01	5.47E-02	4.26E-04
X-07765	-1.26E-01	6.40E-02	4.95E-02	-7.14E-02	6.22E-02	2.52E-01	-1.24E-01	6.01E-02	4.04E-02
X-10458	1.04E-01	6.32E-02	9.99E-02	9.77E-02	6.12E-02	1.12E-01	5.53E-02	5.97E-02	3.55E-01
X-11299	-6.00E-02	6.78E-02	3.77E-01	-1.41E-01	6.51E-02	3.13E-02	-5.29E-02	6.37E-02	4.08E-01
X-11308	-1.92E-01	6.17E-02	2.01E-03	-1.82E-01	5.97E-02	2.55E-03	-1.23E-01	5.85E-02	3.66E-02
X-11315	3.91E-01	5.47E-02	7.83E-12	2.80E-01	5.51E-02	6.99E-07	2.94E-01	5.31E-02	7.23E-08
X-11372	-1.53E-01	6.38E-02	1.73E-02	-6.71E-02	6.23E-02	2.82E-01	-7.87E-02	6.04E-02	1.94E-01
X-11381	-1.88E-01	6.11E-02	2.28E-03	-2.39E-01	5.84E-02	5.44E-05	-1.97E-01	5.72E-02	6.81E-04
X-11444	9.41E-03	6.15E-02	8.78E-01	5.38E-02	5.94E-02	3.66E-01	-4.41E-02	5.77E-02	4.45E-01
X-11470	-3.88E-02	5.94E-02	5.14E-01	-4.51E-02	5.75E-02	4.33E-01	-8.48E-02	5.57E-02	1.29E-01
X-11478	-1.97E-01	6.49E-02	2.63E-03	-1.05E-01	6.36E-02	1.01E-01	-1.66E-01	6.13E-02	7.30E-03
X-11483	-1.01E-01	6.74E-02	1.37E-01	-1.69E-01	6.47E-02	9.40E-03	-7.23E-02	6.35E-02	2.56E-01
X-11632	-3.43E-02	6.36E-02	5.90E-01	-4.18E-02	6.16E-02	4.98E-01	-1.66E-02	5.98E-02	7.82E-01
X-11787	2.16E-01	6.00E-02	3.69E-04	1.77E-01	5.85E-02	2.66E-03	1.23E-01	5.72E-02	3.28E-02
X-11795	-4.59E-02	6.42E-02	4.75E-01	-1.65E-01	6.14E-02	7.58E-03	1.31E-02	6.04E-02	8.28E-01
X-11843	-1.68E-04	6.44E-02	9.98E-01	-1.02E-02	6.23E-02	8.71E-01	-1.14E-01	6.02E-02	5.93E-02
X-11847	2.37E-01	5.99E-02	9.70E-05	6.13E-02	5.95E-02	3.04E-01	2.01E-01	5.66E-02	4.44E-04
X-11849	2.19E-01	5.87E-02	2.35E-04	5.83E-02	5.81E-02	3.16E-01	2.13E-01	5.51E-02	1.34E-04
X-11850	5.63E-02	6.42E-02	3.81E-01	4.16E-02	6.22E-02	5.03E-01	-7.24E-02	6.03E-02	2.31E-01
X-11852	8.16E-02	6.65E-02	2.21E-01	5.24E-02	6.45E-02	4.17E-01	4.31E-02	6.27E-02	4.92E-01
X-11858	1.70E-01	6.34E-02	7.79E-03	4.44E-02	6.21E-02	4.75E-01	1.74E-01	5.95E-02	3.67E-03
X-11880	-1.95E-01	6.31E-02	2.17E-03	-1.01E-01	6.18E-02	1.03E-01	-1.28E-01	5.98E-02	3.37E-02
X-11979	8.73E-02	6.20E-02	1.60E-01	1.50E-01	5.96E-02	1.24E-02	8.14E-03	5.85E-02	8.90E-01
X-12007	1.11E-01	6.25E-02	7.71E-02	1.41E-01	6.02E-02	1.97E-02	5.57E-02	5.90E-02	3.46E-01
X-12013	1.07E-02	6.53E-02	8.70E-01	2.86E-02	6.32E-02	6.51E-01	-9.70E-02	6.11E-02	1.14E-01
X-12026	-2.74E-02	6.20E-02	6.59E-01	1.34E-01	5.95E-02	2.51E-02	-9.67E-02	5.80E-02	9.68E-02
X-12027	5.88E-02	6.62E-02	3.75E-01	1.34E-01	6.37E-02	3.64E-02	-4.18E-02	6.23E-02	5.03E-01
X-12100	5.16E-03	6.34E-02	9.35E-01	8.25E-02	6.12E-02	1.79E-01	-3.97E-02	5.96E-02	5.06E-01
X-12101	-7.87E-02	6.13E-02	2.00E-01	-6.46E-02	5.93E-02	2.77E-01	1.86E-02	5.78E-02	7.48E-01
X-12104	-7.45E-02	6.58E-02	2.59E-01	1.19E-01	6.35E-02	6.13E-02	-8.93E-02	6.18E-02	1.50E-01
X-12111	2.05E-01	6.66E-02	2.28E-03	2.48E-01	6.38E-02	1.24E-04	1.05E-01	6.33E-02	9.94E-02
X-12193	5.45E-02	6.55E-02	4.06E-01	1.16E-01	6.31E-02	6.62E-02	7.45E-02	6.15E-02	2.27E-01
X-12216	-1.22E-02	6.42E-02	8.50E-01	4.88E-02	6.21E-02	4.33E-01	-5.98E-02	6.03E-02	3.22E-01
X-12221	1.24E-01	6.52E-02	5.84E-02	5.84E-02	6.34E-02	3.58E-01	2.92E-02	6.17E-02	6.36E-01
X-12261	1.58E-02	6.42E-02	8.05E-01	3.11E-02	6.21E-02	6.16E-01	-6.95E-02	6.02E-02	2.49E-01
X-12262	3.16E-02	6.43E-02	6.23E-01	3.85E-02	6.22E-02	5.36E-01	-3.87E-02	6.04E-02	5.22E-01
X-12306	2.40E-01	5.95E-02	7.18E-05	1.64E-01	5.84E-02	5.29E-03	2.45E-01	5.56E-02	1.49E-05
X-12407	2.70E-02	6.47E-02	6.77E-01	6.95E-02	6.25E-02	2.67E-01	1.83E-02	6.08E-02	7.63E-01
X-12410	2.22E-01	6.24E-02	4.53E-04	1.79E-01	6.08E-02	3.48E-03	8.65E-02	5.98E-02	1.49E-01
X-12411	7.93E-02	5.85E-02	1.76E-01	9.00E-02	5.65E-02	1.13E-01	6.66E-03	5.52E-02	9.04E-01
X-12456	-7.97E-02	5.69E-02	1.63E-01	-9.83E-02	5.50E-02	7.49E-02	-1.29E-01	5.32E-02	1.61E-02
X-12544	6.46E-02	6.67E-02	3.34E-01	1.23E-01	6.43E-02	5.67E-02	-4.64E-02	6.28E-02	4.60E-01
X-12680	1.44E-01	6.31E-02	2.29E-02	3.30E-01	5.84E-02	3.82E-08	1.01E-01	5.96E-02	9.26E-02
X-12701	1.27E-01	6.33E-02	4.53E-02	1.32E-01	6.12E-02	3.13E-02	9.50E-03	5.99E-02	8.74E-01
X-12707	-5.28E-02	6.38E-02	4.08E-01	7.62E-02	6.17E-02	2.18E-01	-5.02E-02	6.00E-02	4.03E-01
X-12714	1.14E-01	6.42E-02	7.75E-02	9.64E-02	6.22E-02	1.22E-01	4.95E-02	6.06E-02	4.14E-01
X-12726	3.03E-01	6.06E-02	1.01E-06	2.52E-01	5.94E-02	2.99E-05	2.23E-01	5.80E-02	1.45E-04
X-12729	3.54E-02	5.63E-02	5.30E-01	7.48E-02	5.43E-02	1.69E-01	7.27E-02	5.28E-02	1.70E-01
X-12730	5.74E-02	6.38E-02	3.68E-01	-1.76E-02	6.18E-02	7.76E-01	-3.15E-02	6.00E-02	6.00E-01
X-12731	1.29E-01	6.23E-02	3.91E-02	1.52E-01	6.01E-02	1.19E-02	2.32E-02	5.90E-02	6.95E-01
X-12738	4.88E-02	6.49E-02	4.52E-01	4.60E-02	6.28E-02	4.65E-01	4.11E-02	6.10E-02	5.01E-01
X-12740	7.91E-02	6.54E-02	2.27E-01	6.41E-02	6.34E-02	3.13E-01	1.09E-01	6.13E-02	7.67E-02
X-12798	-8.60E-02	6.00E-02	1.53E-01	-1.10E-01	5.79E-02	5.75E-02	-7.85E-02	5.64E-02	1.66E-01
X-12812	1.48E-01	6.31E-02	1.97E-02	1.39E-01	6.12E-02	2.39E-02	1.12E-01	5.96E-02	6.05E-02
X-12815	3.03E-02	6.35E-02	6.33E-01	1.28E-02	6.14E-02	8.35E-01	5.11E-02	5.96E-02	3.92E-01
X-12816	-9.63E-03	6.38E-02	8.80E-01	-9.47E-02	6.15E-02	1.25E-01	-1.54E-02	5.99E-02	7.98E-01
X-12822	-9.43E-02	6.34E-02	1.38E-01	-6.89E-02	6.15E-02	2.63E-01	-1.96E-02	5.99E-02	7.43E-01
X-12830	2.63E-02	6.61E-02	6.91E-01	-8.81E-02	6.37E-02	1.68E-01	3.32E-02	6.21E-02	5.94E-01
X-12839	-8.31E-02	6.05E-02	1.71E-01	-9.05E-02	5.85E-02	1.23E-01	-6.92E-02	5.69E-02	2.25E-01
X-12844	9.92E-02	6.14E-02	1.07E-01	2.69E-02	5.96E-02	6.52E-01	1.42E-02	5.80E-02	8.06E-01
X-12847	-1.47E-02	6.63E-02	8.24E-01	-3.38E-02	6.42E-02	5.99E-01	2.05E-02	6.23E-02	7.43E-01
X-12849	3.55E-02	6.18E-02	5.66E-01	-1.90E-02	5.98E-02	7.51E-01	-7.05E-03	5.81E-02	9.04E-01
X-12851	-2.73E-03	6.39E-02	9.66E-01	-9.38E-03	6.19E-02	8.80E-01	-3.80E-03	6.01E-02	9.50E-01
X-12906	1.07E-01	6.27E-02	8.90E-02	1.55E-01	6.03E-02	1.05E-02	7.37E-02	5.91E-02	2.13E-01
X-13431	-9.14E-02	6.13E-02	1.37E-01	-3.99E-02	5.96E-02	5.03E-01	-6.56E-02	5.78E-02	2.57E-01
X-13507	1.42E-01	6.25E-02	2.38E-02	1.06E-01	6.07E-02	8.05E-02	1.39E-01	5.87E-02	1.89E-02
X-13553	-2.38E-02	5.93E-02	6.88E-01	8.26E-02	5.72E-02	1.50E-01	-4.34E-02	5.57E-02	4.37E-01
X-13658	2.89E-02	6.42E-02	6.53E-01	8.39E-02	6.19E-02	1.76E-01	4.72E-02	6.03E-02	4.34E-01
X-13695	1.08E-01	6.68E-02	1.06E-01	4.05E-02	6.49E-02	5.33E-01	3.94E-02	6.30E-02	5.32E-01
X-13723	1.23E-01	6.39E-02	5.55E-02	1.19E-01	6.19E-02	5.58E-02	1.08E-01	6.02E-02	7.26E-02
X-13726	2.04E-01	6.61E-02	2.28E-03	1.73E-01	6.43E-02	7.47E-03	9.43E-02	6.30E-02	1.35E-01
X-13728	-5.53E-02	6.27E-02	3.78E-01	5.93E-02	6.07E-02	3.29E-01	-2.41E-02	5.90E-02	6.83E-01
X-13729	-6.01E-03	6.50E-02	9.26E-01	1.05E-01	6.26E-02	9.48E-02	-4.95E-02	6.11E-02	4.18E-01
X-13844	1.28E-01	5.94E-02	3.16E-02	1.73E-01	5.70E-02	2.64E-03	5.03E-02	5.62E-02	3.71E-01
X-13846	9.44E-02	6.66E-02	1.57E-01	3.93E-02	6.46E-02	5.44E-01	3.54E-02	6.28E-02	5.74E-01
X-13866	3.41E-02	6.45E-02	5.97E-01	-3.74E-02	6.24E-02	5.49E-01	9.38E-02	6.04E-02	1.22E-01
X-14056	-2.46E-01	5.67E-02	2.01E-05	-2.00E-01	5.54E-02	3.74E-04	-1.64E-01	5.42E-02	2.67E-03
X-14939	-1.12E-01	6.28E-02	7.52E-02	-9.37E-02	6.09E-02	1.25E-01	-5.01E-02	5.93E-02	3.99E-01
X-15486	-2.28E-01	6.26E-02	3.28E-04	-1.40E-01	6.15E-02	2.34E-02	-1.84E-01	5.93E-02	2.07E-03
X-15503	2.17E-02	5.87E-02	7.12E-01	1.30E-01	5.63E-02	2.20E-02	-4.64E-02	5.51E-02	4.01E-01

X-15728	1.14E-01	6.43E-02	7.78E-02	3.60E-02	6.25E-02	5.66E-01	3.01E-02	6.07E-02	6.21E-01
X-16087	-1.15E-01	6.07E-02	6.02E-02	-1.12E-01	5.88E-02	5.68E-02	-5.38E-02	5.74E-02	3.49E-01
X-16124	5.59E-02	6.51E-02	3.91E-01	-3.31E-02	6.31E-02	6.00E-01	4.26E-02	6.13E-02	4.87E-01
X-16397	-1.14E-01	6.41E-02	7.59E-02	-8.59E-02	6.21E-02	1.68E-01	-3.52E-02	6.05E-02	5.62E-01
X-16576	2.10E-02	6.32E-02	7.40E-01	7.09E-03	6.12E-02	9.08E-01	-2.46E-03	5.94E-02	9.67E-01
X-16580	-1.47E-01	6.21E-02	1.88E-02	-8.79E-02	6.04E-02	1.47E-01	-8.48E-02	5.87E-02	1.50E-01
X-16649	7.50E-02	6.62E-02	2.58E-01	1.15E-01	6.39E-02	7.40E-02	2.82E-02	6.24E-02	6.52E-01
X-16935	-2.17E-01	5.76E-02	1.98E-04	-1.38E-01	5.65E-02	1.54E-02	-1.03E-01	5.52E-02	6.42E-02
X-16964	-1.22E-01	6.27E-02	5.34E-02	-2.24E-01	5.96E-02	2.02E-04	-1.09E-01	5.90E-02	6.52E-02
X-17010	-2.28E-02	6.15E-02	7.11E-01	-8.75E-02	5.93E-02	1.42E-01	-9.51E-03	5.79E-02	8.70E-01
X-17146	-6.77E-04	6.57E-02	9.92E-01	-8.09E-02	6.34E-02	2.03E-01	-5.67E-03	6.17E-02	9.27E-01
X-17162	-1.60E-02	6.28E-02	7.99E-01	-3.84E-02	6.08E-02	5.28E-01	-4.26E-02	5.90E-02	4.71E-01
X-17301	-2.09E-02	4.75E-02	6.60E-01	-6.50E-02	4.59E-02	1.58E-01	-1.86E-02	4.47E-02	6.77E-01
X-17306	-8.75E-03	5.94E-02	8.83E-01	-1.03E-01	5.71E-02	7.34E-02	5.87E-03	5.58E-02	9.16E-01
X-17325	1.13E-01	6.39E-02	7.72E-02	1.66E-01	6.14E-02	7.39E-03	7.20E-02	6.03E-02	2.33E-01
X-17335	-6.31E-02	6.11E-02	3.03E-01	-9.61E-02	5.90E-02	1.04E-01	-5.98E-02	5.74E-02	2.99E-01
X-17346	-1.29E-01	6.42E-02	4.49E-02	-1.41E-02	6.25E-02	8.22E-01	-1.38E-01	6.02E-02	2.31E-02
X-17348	9.44E-02	6.43E-02	1.44E-01	-5.24E-04	6.25E-02	9.93E-01	1.00E-01	6.04E-02	9.80E-02
X-17351	2.62E-01	5.83E-02	9.89E-06	2.54E-01	5.64E-02	9.53E-06	1.85E-01	5.56E-02	9.91E-04
X-17353	-1.29E-02	6.19E-02	8.36E-01	3.14E-02	5.99E-02	6.00E-01	1.28E-03	5.82E-02	9.83E-01
X-17354	2.34E-01	6.17E-02	1.76E-04	1.67E-01	6.04E-02	5.93E-03	1.48E-01	5.88E-02	1.22E-02
X-17357	2.29E-02	6.13E-02	7.09E-01	-1.79E-03	5.93E-02	9.76E-01	-2.23E-02	5.76E-02	6.99E-01
X-17365	2.96E-02	6.47E-02	6.47E-01	-2.58E-02	6.26E-02	6.80E-01	1.36E-01	6.03E-02	2.53E-02
X-17367	7.66E-02	6.46E-02	2.37E-01	1.52E-01	6.20E-02	1.50E-02	4.33E-02	6.08E-02	4.77E-01
X-17438	2.09E-01	6.25E-02	9.14E-04	1.47E-01	6.11E-02	1.69E-02	1.11E-01	5.95E-02	6.45E-02
X-17612	5.46E-02	6.52E-02	4.04E-01	3.53E-02	6.32E-02	5.77E-01	-3.37E-02	6.14E-02	5.83E-01
X-17653	-1.13E-01	6.44E-02	7.96E-02	-7.94E-02	6.25E-02	2.04E-01	-1.08E-01	6.05E-02	7.41E-02
X-17654	1.54E-02	6.58E-02	8.15E-01	9.27E-02	6.35E-02	1.45E-01	1.69E-02	6.19E-02	7.85E-01
X-17655	3.53E-02	6.55E-02	5.90E-01	-3.61E-02	6.34E-02	5.70E-01	2.68E-02	6.16E-02	6.63E-01
X-17676	5.89E-02	6.30E-02	3.51E-01	1.72E-01	6.01E-02	4.51E-03	2.93E-03	5.93E-02	9.61E-01
X-17682	-1.05E-01	6.12E-02	8.88E-02	-2.20E-02	5.96E-02	7.13E-01	-9.18E-02	5.76E-02	1.12E-01
X-17685	9.06E-02	6.39E-02	1.57E-01	5.70E-02	6.20E-02	3.58E-01	1.61E-01	5.95E-02	7.12E-03
X-17686	1.22E-01	6.66E-02	6.72E-02	7.98E-02	6.47E-02	2.18E-01	6.31E-02	6.29E-02	3.17E-01
X-17690	7.98E-02	6.61E-02	2.29E-01	-2.15E-02	6.41E-02	7.37E-01	7.89E-02	6.21E-02	2.05E-01
X-17692	-5.94E-02	6.61E-02	3.70E-01	-7.76E-02	6.39E-02	2.26E-01	2.53E-02	6.22E-02	6.85E-01
X-17735	1.14E-02	6.42E-02	8.59E-01	9.25E-02	6.19E-02	1.36E-01	-4.84E-03	6.03E-02	9.36E-01
X-17761	-1.06E-01	6.29E-02	9.40E-02	-2.30E-01	5.97E-02	1.40E-04	-7.97E-02	5.93E-02	1.80E-01
X-17765	7.62E-02	6.10E-02	2.13E-01	3.75E-02	5.92E-02	5.27E-01	-9.84E-03	5.75E-02	8.64E-01
X-18240	5.84E-03	6.37E-02	9.27E-01	-4.23E-02	6.16E-02	4.93E-01	-5.76E-02	5.98E-02	3.36E-01
X-18345	-1.89E-02	6.58E-02	7.75E-01	1.03E-01	6.34E-02	1.05E-01	4.35E-02	6.18E-02	4.82E-01
X-18779	-2.54E-02	6.46E-02	6.94E-01	-5.90E-02	6.24E-02	3.45E-01	-1.27E-01	6.02E-02	3.52E-02
X-18838	-4.80E-02	5.54E-02	3.86E-01	-8.18E-03	5.36E-02	8.79E-01	1.07E-02	5.21E-02	8.38E-01
X-18886	-1.85E-01	6.21E-02	3.19E-03	-2.17E-01	5.96E-02	3.25E-04	-1.54E-01	5.86E-02	9.04E-03
X-18887	-1.32E-01	6.27E-02	3.69E-02	-2.23E-02	6.12E-02	7.15E-01	-4.23E-02	5.94E-02	4.77E-01
X-18888	4.08E-02	6.32E-02	5.19E-01	5.92E-02	6.11E-02	3.33E-01	2.64E-02	5.94E-02	6.57E-01
X-18899	-8.76E-03	6.43E-02	8.92E-01	-2.05E-02	6.22E-02	7.42E-01	2.30E-02	6.04E-02	7.04E-01
X-18901	1.43E-01	5.65E-02	1.19E-02	8.09E-02	5.51E-02	1.43E-01	1.20E-01	5.32E-02	2.46E-02
X-18913	-1.30E-02	6.46E-02	8.40E-01	6.17E-02	6.24E-02	3.24E-01	1.47E-03	6.07E-02	9.81E-01
X-18921	-1.87E-01	6.46E-02	4.05E-03	-1.70E-01	6.26E-02	7.16E-03	-1.54E-01	6.09E-02	1.21E-02
X-18922	-6.62E-02	6.55E-02	3.13E-01	-1.49E-01	6.29E-02	1.86E-02	3.51E-02	6.17E-02	5.70E-01
X-18935	1.03E-01	6.41E-02	1.11E-01	6.49E-02	6.22E-02	2.98E-01	6.70E-02	6.04E-02	2.69E-01
X-19141	5.08E-02	6.20E-02	4.13E-01	9.95E-02	5.98E-02	9.75E-02	3.31E-02	5.84E-02	5.71E-01
X-19183	5.31E-02	6.88E-02	4.41E-01	-2.56E-02	6.67E-02	7.01E-01	1.14E-01	6.44E-02	7.81E-02
X-19299	6.98E-02	6.50E-02	2.84E-01	5.54E-02	6.30E-02	3.80E-01	6.41E-03	6.12E-02	9.17E-01
X-19438	-1.55E-02	6.59E-02	8.14E-01	-3.26E-02	6.38E-02	6.09E-01	-3.50E-02	6.20E-02	5.73E-01
X-21258	6.69E-02	6.54E-02	3.08E-01	5.11E-02	6.34E-02	4.21E-01	6.56E-02	6.15E-02	2.87E-01
X-21286	-3.24E-02	6.40E-02	6.13E-01	2.32E-02	6.20E-02	7.09E-01	-2.67E-02	6.02E-02	6.58E-01
X-21310	-1.15E-01	6.41E-02	7.30E-02	-9.28E-02	6.22E-02	1.37E-01	-1.33E-01	6.01E-02	2.73E-02
X-21312	6.91E-03	6.58E-02	9.16E-01	-1.00E-02	6.37E-02	8.75E-01	3.41E-02	6.18E-02	5.82E-01
X-21315	-4.02E-02	5.40E-02	4.57E-01	-1.93E-02	5.23E-02	7.13E-01	-4.35E-02	5.07E-02	3.92E-01
X-21319	-1.66E-01	6.48E-02	1.08E-02	-1.26E-01	6.30E-02	4.63E-02	-1.39E-01	6.11E-02	2.41E-02
X-21339	-2.31E-01	6.19E-02	2.33E-04	-1.66E-01	6.06E-02	6.48E-03	-1.46E-01	5.90E-02	1.40E-02
X-21351	-5.31E-03	6.58E-02	9.36E-01	-1.39E-02	6.37E-02	8.27E-01	-5.40E-03	6.19E-02	9.31E-01
X-21353	-8.54E-02	6.40E-02	1.83E-01	-3.89E-02	6.21E-02	5.31E-01	-5.77E-02	6.03E-02	3.39E-01
X-21364	-1.60E-01	5.85E-02	6.50E-03	-6.40E-02	5.72E-02	2.64E-01	-1.53E-01	5.49E-02	5.78E-03
X-21383	-1.09E-01	6.23E-02	8.02E-02	-1.64E-01	5.98E-02	6.66E-03	-7.78E-02	5.87E-02	1.86E-01
X-21441	-1.57E-01	6.09E-02	1.05E-02	-1.12E-01	5.92E-02	5.95E-02	-1.48E-01	5.72E-02	1.01E-02
X-21442	3.15E-02	6.18E-02	6.11E-01	-7.84E-02	5.97E-02	1.90E-01	-1.81E-02	5.81E-02	7.56E-01
X-21467	-8.68E-02	6.34E-02	1.72E-01	3.58E-02	6.15E-02	5.61E-01	-3.63E-02	5.97E-02	5.43E-01
X-21470	-9.06E-02	6.14E-02	1.41E-01	-1.52E-01	5.89E-02	1.03E-02	-1.11E-01	5.75E-02	5.40E-02
X-21471	-6.59E-02	6.01E-02	2.74E-01	4.01E-02	5.83E-02	4.92E-01	-5.84E-02	5.65E-02	3.02E-01
X-21607	1.64E-03	6.51E-02	9.80E-01	-1.97E-02	6.30E-02	7.54E-01	-5.52E-04	6.12E-02	9.93E-01
X-21628	1.59E-01	6.88E-02	2.12E-02	1.31E-01	6.68E-02	5.12E-02	8.77E-02	6.51E-02	1.79E-01
X-21661	1.46E-01	6.42E-02	2.41E-02	1.49E-02	6.27E-02	8.13E-01	1.54E-01	6.03E-02	1.12E-02
X-21733	2.15E-01	6.07E-02	4.57E-04	2.38E-01	5.83E-02	5.69E-05	7.36E-02	5.81E-02	2.07E-01
X-21736	-2.54E-01	6.10E-02	4.33E-05	-3.02E-01	5.81E-02	3.95E-07	-1.58E-01	5.84E-02	7.29E-03
X-21740	-1.69E-01	6.37E-02	8.62E-03	-1.70E-01	6.16E-02	6.07E-03	-8.47E-02	6.04E-02	1.62E-01
X-21742	2.74E-02	5.94E-02	6.45E-01	1.54E-02	5.75E-02	7.89E-01	-4.52E-02	5.58E-02	4.19E-01
X-21752	2.81E-01	5.97E-02	4.02E-06	3.92E-01	5.52E-02	9.99E-12	8.97E-02	5.81E-02	1.24E-01
X-21788	-1.19E-01	6.22E-02	5.71E-02	-4.54E-02	6.05E-02	4.54E-01	-8.80E-02	5.86E-02	1.34E-01
X-21796	-6.99E-02	6.00E-02	2.45E-01	-4.85E-02	5.81E-02	4.05E-01	-3.27E-02	5.65E-02	5.63E-01
X-21803	5.49E-03	6.52E-02	9.33E-01	1.54E-03	6.31E-02	9.81E-01	5.45E-03	6.13E-02	9.29E-01

Supplementary Table 4: Parameter estimates for metabolome-wide association studies for diet-metabolite associations for each of: HEI-15, DASH and AMED diet, controlling for BMI

X-21807	1.67E-01	6.45E-02	1.03E-02	1.64E-01	6.24E-02	8.99E-03	9.82E-02	6.11E-02	1.09E-01
X-21815	-4.24E-02	6.15E-02	4.91E-01	-1.11E-01	5.92E-02	6.12E-02	-7.86E-02	5.77E-02	1.74E-01
X-21816	-6.58E-02	6.31E-02	2.98E-01	-1.54E-02	6.11E-02	8.01E-01	-6.38E-02	5.93E-02	2.83E-01
X-21821	2.38E-01	5.84E-02	5.77E-05	2.28E-01	5.65E-02	7.33E-05	1.85E-01	5.54E-02	9.72E-04
X-21830	6.84E-02	6.35E-02	2.82E-01	6.01E-02	6.15E-02	3.29E-01	-2.43E-02	5.98E-02	6.85E-01
X-21831	9.96E-02	6.20E-02	1.09E-01	1.12E-01	5.99E-02	6.16E-02	2.62E-02	5.85E-02	6.54E-01
X-21834	1.20E-01	6.50E-02	6.62E-02	5.84E-02	6.32E-02	3.56E-01	1.08E-01	6.11E-02	7.85E-02
X-21838	-8.16E-02	6.39E-02	2.02E-01	2.15E-02	6.20E-02	7.29E-01	-4.63E-02	6.02E-02	4.42E-01
X-21839	5.30E-02	6.71E-02	4.30E-01	-5.49E-02	6.49E-02	3.98E-01	3.21E-02	6.31E-02	6.11E-01
X-21840	8.85E-02	6.42E-02	1.69E-01	6.22E-02	6.23E-02	3.19E-01	7.60E-02	6.04E-02	2.09E-01
X-21842	2.63E-01	6.05E-02	1.89E-05	2.60E-01	5.85E-02	1.28E-05	1.87E-01	5.77E-02	1.35E-03
X-21845	-2.65E-02	6.63E-02	6.90E-01	-1.68E-01	6.34E-02	8.53E-03	8.12E-03	6.23E-02	8.96E-01
X-21851	-7.91E-02	6.48E-02	2.23E-01	-4.44E-02	6.28E-02	4.80E-01	-9.89E-02	6.08E-02	1.05E-01
X-22162	1.47E-01	6.06E-02	1.58E-02	1.14E-01	5.89E-02	5.46E-02	1.55E-01	5.68E-02	6.74E-03
X-22509	2.03E-01	6.22E-02	1.27E-03	9.19E-02	6.11E-02	1.34E-01	1.43E-01	5.90E-02	1.57E-02
X-22771	-1.02E-01	6.35E-02	1.10E-01	-2.26E-01	6.03E-02	2.10E-04	-9.47E-02	5.97E-02	1.14E-01
X-22776	-1.65E-02	6.48E-02	7.99E-01	-7.54E-02	6.26E-02	2.29E-01	-1.44E-02	6.09E-02	8.14E-01
X-22834	-3.57E-02	6.44E-02	5.80E-01	-6.23E-03	6.24E-02	9.20E-01	-1.13E-01	6.02E-02	6.25E-02
X-23276	1.35E-03	6.58E-02	9.84E-01	-4.04E-02	6.36E-02	5.25E-01	-2.51E-02	6.18E-02	6.85E-01
X-23481	-5.90E-03	6.46E-02	9.27E-01	2.12E-02	6.25E-02	7.34E-01	-5.70E-02	6.06E-02	3.48E-01
X-23587	-2.08E-02	6.25E-02	7.39E-01	-1.71E-01	5.96E-02	4.41E-03	6.11E-02	5.87E-02	2.98E-01
X-23593	2.43E-02	6.42E-02	7.05E-01	8.91E-02	6.19E-02	1.51E-01	3.73E-02	6.03E-02	5.37E-01
X-23636	-4.19E-02	6.49E-02	5.19E-01	-1.00E-01	6.26E-02	1.10E-01	-1.12E-01	6.07E-02	6.70E-02
X-23639	1.75E-02	6.01E-02	7.71E-01	-1.92E-02	5.81E-02	7.42E-01	6.61E-02	5.64E-02	2.42E-01
X-23641	3.77E-02	6.33E-02	5.52E-01	3.70E-03	6.13E-02	9.52E-01	-4.43E-02	5.95E-02	4.57E-01
X-23644	2.00E-01	6.37E-02	1.88E-03	2.49E-01	6.09E-02	5.63E-05	1.42E-01	6.04E-02	1.93E-02
X-23654	1.05E-01	6.25E-02	9.41E-02	1.13E-01	6.04E-02	6.30E-02	6.23E-02	5.89E-02	2.91E-01
X-23659	1.81E-01	5.98E-02	2.68E-03	2.23E-01	5.73E-02	1.23E-04	1.29E-01	5.66E-02	2.37E-02
X-23678	-1.03E-01	6.25E-02	1.01E-01	-7.50E-02	6.06E-02	2.17E-01	-6.95E-02	5.89E-02	2.39E-01
X-23680	-1.37E-01	6.44E-02	3.38E-02	-6.95E-02	6.27E-02	2.68E-01	-8.11E-02	6.08E-02	1.83E-01
X-23739	-7.35E-04	6.32E-02	9.91E-01	-2.82E-02	6.11E-02	6.45E-01	4.58E-03	5.94E-02	9.39E-01
X-23767	-1.78E-01	6.17E-02	4.20E-03	-8.77E-02	6.04E-02	1.48E-01	-2.74E-02	5.88E-02	6.41E-01
X-23780	1.10E-01	6.54E-02	9.31E-02	5.56E-02	6.35E-02	3.82E-01	1.17E-01	6.14E-02	5.84E-02
X-23782	3.59E-02	5.87E-02	5.41E-01	-5.82E-03	5.68E-02	9.19E-01	1.39E-01	5.46E-02	1.12E-02
X-23787	-1.98E-01	6.13E-02	1.38E-03	-1.70E-01	5.96E-02	4.67E-03	-1.47E-01	5.80E-02	1.16E-02
X-23890	1.12E-01	6.38E-02	8.14E-02	1.39E-01	6.16E-02	2.42E-02	3.01E-02	6.03E-02	6.18E-01
X-23974	4.28E-03	6.47E-02	9.47E-01	3.50E-02	6.26E-02	5.76E-01	6.57E-02	6.07E-02	2.80E-01
X-23997	5.43E-02	6.49E-02	4.03E-01	5.72E-02	6.28E-02	3.63E-01	6.09E-03	6.11E-02	9.21E-01
X-24295	-5.72E-02	6.22E-02	3.59E-01	-3.01E-02	6.03E-02	6.19E-01	-3.58E-02	5.86E-02	5.41E-01
X-24306	1.58E-02	6.20E-02	7.99E-01	-5.53E-02	5.99E-02	3.57E-01	-4.62E-02	5.82E-02	4.28E-01
X-24307	-2.36E-02	6.33E-02	7.10E-01	-3.64E-02	6.12E-02	5.53E-01	-8.83E-02	5.92E-02	1.37E-01
X-24309	-1.15E-01	6.05E-02	5.79E-02	-6.04E-02	5.88E-02	3.06E-01	-1.29E-01	5.67E-02	2.34E-02
X-24328	-7.19E-02	5.25E-02	1.72E-01	-5.82E-02	5.08E-02	2.53E-01	-1.13E-01	4.90E-02	2.17E-02
X-24334	-3.45E-02	5.57E-02	5.37E-01	2.29E-02	5.39E-02	6.72E-01	-9.88E-02	5.21E-02	5.88E-02
X-24337	-9.58E-02	5.69E-02	9.34E-02	-1.67E-02	5.54E-02	7.63E-01	-1.37E-01	5.32E-02	1.04E-02
X-24338	5.58E-02	5.89E-02	3.44E-01	5.82E-02	5.70E-02	3.08E-01	5.64E-03	5.54E-02	9.19E-01
X-24344	-3.90E-02	5.86E-02	5.06E-01	-7.56E-02	5.66E-02	1.83E-01	-2.75E-02	5.51E-02	6.18E-01
X-24352	1.80E-01	6.66E-02	7.24E-03	4.72E-02	6.52E-02	4.70E-01	7.24E-02	6.33E-02	2.54E-01
X-24414	-1.68E-03	5.62E-02	9.76E-01	-1.77E-02	5.44E-02	7.44E-01	-3.77E-02	5.28E-02	4.76E-01
X-24418	8.97E-02	6.41E-02	1.63E-01	-2.51E-02	6.22E-02	6.87E-01	3.83E-02	6.04E-02	5.26E-01
X-24456	-3.28E-02	6.41E-02	6.09E-01	-5.61E-03	6.21E-02	9.28E-01	5.06E-02	6.02E-02	4.01E-01
X-24475	2.13E-01	6.08E-02	5.29E-04	1.88E-01	5.91E-02	1.62E-03	1.48E-01	5.77E-02	1.11E-02
X-24494	7.67E-02	6.47E-02	2.37E-01	-8.87E-03	6.28E-02	8.88E-01	5.52E-02	6.09E-02	3.65E-01
X-24541	1.43E-01	6.38E-02	2.53E-02	7.05E-02	6.22E-02	2.58E-01	1.54E-01	5.98E-02	1.08E-02
X-24543	-1.44E-01	6.16E-02	2.04E-02	-1.08E-01	5.99E-02	7.30E-02	-1.26E-01	5.80E-02	3.11E-02
X-24544	-1.79E-01	5.80E-02	2.24E-03	-1.14E-01	5.67E-02	4.50E-02	-2.05E-01	5.41E-02	1.93E-04
X-24545	1.20E-01	6.60E-02	7.05E-02	3.57E-02	6.42E-02	5.78E-01	4.25E-02	6.23E-02	4.96E-01
X-24546	-1.20E-01	6.05E-02	4.85E-02	-9.97E-02	5.87E-02	9.06E-02	-1.72E-01	5.64E-02	2.48E-03
X-24556	-7.58E-03	6.23E-02	9.03E-01	7.73E-02	6.01E-02	2.00E-01	8.01E-02	5.84E-02	1.71E-01
X-24565	-8.18E-02	6.73E-02	2.25E-01	6.19E-02	6.52E-02	3.44E-01	-9.11E-02	6.32E-02	1.51E-01
X-24571	-3.80E-02	6.63E-02	5.67E-01	-6.59E-02	6.41E-02	3.05E-01	-1.44E-02	6.23E-02	8.17E-01
X-24576	6.23E-02	6.84E-02	3.63E-01	6.66E-02	6.62E-02	3.15E-01	1.16E-02	6.44E-02	8.57E-01
X-24588	-1.42E-01	5.68E-02	1.28E-02	-1.39E-01	5.50E-02	1.23E-02	-8.77E-02	5.38E-02	1.04E-01
X-24657	7.06E-02	6.75E-02	2.96E-01	9.81E-02	6.52E-02	1.33E-01	1.35E-02	6.35E-02	8.32E-01
X-24736	2.61E-01	6.40E-02	5.71E-05	1.72E-01	6.29E-02	6.51E-03	2.10E-01	6.06E-02	6.13E-04
X-24747	-9.05E-03	6.17E-02	8.84E-01	8.02E-02	5.95E-02	1.79E-01	1.28E-02	5.80E-02	8.25E-01
X-24748	7.57E-02	6.65E-02	2.56E-01	5.72E-02	6.44E-02	3.76E-01	8.39E-02	6.25E-02	1.81E-01
X-24757	1.75E-01	6.52E-02	7.73E-03	2.11E-01	6.26E-02	8.42E-04	7.47E-02	6.19E-02	2.29E-01
X-24761	5.18E-02	6.29E-02	4.11E-01	5.48E-02	6.09E-02	3.69E-01	7.52E-02	5.90E-02	2.04E-01
X-24762	-5.30E-02	5.95E-02	3.74E-01	-4.25E-02	5.76E-02	4.62E-01	-6.08E-02	5.59E-02	2.78E-01
X-24947	-1.69E-01	5.87E-02	4.40E-03	-1.49E-01	5.70E-02	9.27E-03	-1.26E-01	5.55E-02	2.41E-02
X-24949	-1.33E-01	6.44E-02	3.94E-02	-1.35E-01	6.23E-02	3.06E-02	-3.93E-02	6.10E-02	5.20E-01
X-24951	-1.16E-01	6.25E-02	6.41E-02	-1.60E-01	6.01E-02	8.06E-03	-1.13E-01	5.87E-02	5.62E-02
X-24953	-6.36E-02	6.03E-02	2.92E-01	-8.61E-02	5.83E-02	1.40E-01	-5.82E-02	5.67E-02	3.05E-01
X-24970	-7.97E-03	6.27E-02	8.99E-01	-3.72E-02	6.07E-02	5.40E-01	-4.32E-02	5.89E-02	4.65E-01
X-24980	1.34E-02	6.35E-02	8.33E-01	9.05E-02	6.13E-02	1.41E-01	-7.52E-02	5.96E-02	2.08E-01
X-25009	-6.22E-02	6.45E-02	3.35E-01	4.08E-02	6.24E-02	5.14E-01	1.54E-02	6.07E-02	7.99E-01
X-25172	5.27E-03	6.54E-02	9.36E-01	1.51E-03	6.33E-02	9.81E-01	-2.96E-02	6.14E-02	6.31E-01
X-25217	3.55E-02	6.62E-02	5.92E-01	1.27E-02	6.41E-02	8.43E-01	-7.29E-04	6.23E-02	9.91E-01
X-25247	4.98E-02	6.46E-02	4.41E-01	1.34E-01	6.21E-02	3.15E-02	6.31E-02	6.07E-02	3.00E-01
X-25271	1.59E-01	6.52E-02	1.51E-02	1.36E-01	6.33E-02	3.21E-02	5.45E-02	6.19E-02	3.79E-01
X-25279	6.53E-02	5.71E-02	2.54E-01	-4.48E-02	5.53E-02	4.19E-01	1.54E-02	5.38E-02	7.75E-01

X-25343	-2.87E-02	6.44E-02	6.56E-01	-5.72E-02	6.22E-02	3.59E-01	-4.10E-02	6.05E-02	4.99E-01
X-25371	-1.94E-02	5.62E-02	7.30E-01	9.06E-02	5.42E-02	9.55E-02	-1.12E-01	5.25E-02	3.43E-02
X-25417	2.51E-02	6.73E-02	7.10E-01	-1.48E-01	6.46E-02	2.25E-02	7.93E-02	6.31E-02	2.10E-01
X-25419	6.92E-02	6.23E-02	2.67E-01	-1.52E-01	5.97E-02	1.13E-02	1.75E-01	5.77E-02	2.66E-03
X-25420	-1.68E-02	6.21E-02	7.87E-01	-2.01E-02	6.01E-02	7.39E-01	-4.80E-02	5.83E-02	4.11E-01
X-25422	-1.35E-01	5.87E-02	2.20E-02	-1.56E-01	5.66E-02	6.36E-03	-7.63E-02	5.55E-02	1.71E-01
X-25433	-6.08E-02	6.45E-02	3.46E-01	-5.97E-02	6.24E-02	3.40E-01	-1.44E-01	6.01E-02	1.75E-02
X-25450	-2.67E-02	6.08E-02	6.62E-01	6.03E-02	5.88E-02	3.06E-01	-2.03E-02	5.72E-02	7.24E-01
X-25454	8.35E-02	6.30E-02	1.86E-01	9.42E-02	6.09E-02	1.23E-01	-4.07E-02	5.94E-02	4.94E-01
X-25457	-4.69E-02	5.64E-02	4.06E-01	3.10E-02	5.46E-02	5.70E-01	2.66E-02	5.30E-02	6.17E-01
X-25468	9.60E-03	6.42E-02	8.81E-01	1.17E-01	6.18E-02	5.99E-02	-1.75E-02	6.04E-02	7.72E-01
X-25519	-1.35E-01	6.40E-02	3.53E-02	-6.92E-03	6.24E-02	9.12E-01	-4.65E-02	6.06E-02	4.44E-01
X-25520	4.94E-02	6.30E-02	4.34E-01	4.42E-02	6.09E-02	4.69E-01	4.53E-02	5.92E-02	4.45E-01
X-25524	1.69E-01	6.36E-02	8.42E-03	1.31E-01	6.18E-02	3.51E-02	1.09E-01	6.02E-02	7.21E-02
X-25656	9.47E-02	6.49E-02	1.46E-01	1.69E-02	6.31E-02	7.89E-01	5.88E-02	6.12E-02	3.37E-01
X-25790	4.42E-02	6.07E-02	4.67E-01	6.35E-02	5.87E-02	2.80E-01	3.86E-02	5.71E-02	4.99E-01
X-25805	-3.71E-02	6.43E-02	5.64E-01	-3.55E-02	6.22E-02	5.69E-01	-1.59E-03	6.04E-02	9.79E-01
X-25810	-7.12E-03	6.47E-02	9.12E-01	-4.24E-02	6.25E-02	4.98E-01	1.05E-01	6.05E-02	8.39E-02
X-25937	-1.34E-01	5.89E-02	2.38E-02	-7.38E-02	5.74E-02	2.00E-01	-9.16E-02	5.56E-02	1.01E-01
X-25957	1.16E-01	6.23E-02	6.42E-02	1.21E-01	6.02E-02	4.48E-02	1.32E-01	5.84E-02	2.48E-02
X-25991	9.82E-02	6.19E-02	1.14E-01	1.35E-01	5.97E-02	2.48E-02	7.83E-02	5.83E-02	1.81E-01
X-26054	-5.64E-05	6.35E-02	9.99E-01	5.39E-02	6.14E-02	3.80E-01	-4.86E-03	5.97E-02	9.35E-01
X-26062	4.21E-02	6.27E-02	5.02E-01	-3.25E-01	5.75E-02	3.98E-08	5.60E-02	5.89E-02	3.42E-01
X-26106	-8.18E-02	6.01E-02	1.75E-01	-1.38E-02	5.83E-02	8.13E-01	-8.06E-02	5.65E-02	1.54E-01
X-26107	-6.22E-02	6.05E-02	3.04E-01	-2.92E-02	5.86E-02	6.19E-01	-6.91E-02	5.68E-02	2.25E-01
X-26108	-9.15E-02	6.11E-02	1.36E-01	-7.18E-02	5.92E-02	2.27E-01	-8.92E-02	5.75E-02	1.22E-01
X-26109	-7.73E-02	5.61E-02	1.69E-01	-4.58E-02	5.44E-02	4.01E-01	-1.15E-01	5.25E-02	2.91E-02
X-26111	-1.60E-01	6.51E-02	1.45E-02	-2.49E-01	6.20E-02	7.76E-05	-1.13E-01	6.15E-02	6.77E-02
X-26119	-3.19E-02	6.46E-02	6.22E-01	-4.40E-03	6.25E-02	9.44E-01	-6.63E-02	6.06E-02	2.75E-01

Supplementary Table 5: Standardized parameter estimates for associations between diet-related metabolites and six measures of glucose and insulin homeostasis.

Supplementary Table 5: Standardized parameter estimates for associations between diet-related metabolites and six measures of glucose and insulin homeostasis.

		Fasting Glucose			Fasting Insulin			C Peptide			Insulin Sensitivity			Insulin Secretion			Disposition Index		
Metabolite	Diet	B	SE	P	B	SE	P	B	SE	P	B	SE	P	B	SE	P	B	SE	P
1-eicosenoyl-GPC (20:1)*	HEI-15, AMEI	**-0.32**	**0.06**	**2.77E-08**	**-0.45**	**0.05**	**4.05E-18**	**-0.51**	**0.05**	**2.54E-22**	**0.49**	**0.05**	**1.22E-22**	**-0.26**	**0.05**	**4.48E-06**	**0.32**	**0.06**	**3.13E-08**
catechol sulfate	HEI-15, DASH	**-0.28**	**0.06**	**5.98E-06**	-0.14	0.06	0.02	-0.12	0.06	0.04	0.19	0.06	8.44E-04	0.07	0.06	0.23	**0.32**	**0.06**	**1.54E-07**
1-arachidoyl-GPC (20:0)	HEI-15, DASH	**-0.27**	**0.05**	**1.93E-06**	**-0.35**	**0.05**	**1.87E-11**	**-0.42**	**0.05**	**1.39E-15**	**0.39**	**0.05**	**1.45E-14**	-0.19	0.05	7.02E-04	**0.27**	**0.06**	**1.35E-06**
X-14056	HEI-15	**0.3**	**0.06**	**6.18E-07**	**0.37**	**0.05**	**2.85E-11**	**0.5**	**0.05**	**3.59E-19**	**-0.39**	**0.05**	**7.87E-13**	0.17	0.06	0.003	**-0.28**	**0.06**	**3.97E-06**
methyl glucopyranoside (a	HEI-15	**-0.21**	**0.06**	**2.04E-04**	**-0.2**	**0.05**	**2.66E-04**	-0.16	0.06	0.01	**0.22**	**0.05**	**6.02E-05**	-0.01	0.06	0.89	**0.26**	**0.06**	**8.39E-06**
10-heptadecenoate (17:1r	HEI-15, DASH	0.15	0.06	0.02	0.04	0.06	0.51	0.1	0.06	0.11	-0.12	0.06	0.04	-0.11	0.06	0.06	**-0.28**	**0.06**	**1.01E-05**
1-erucoyl-GPC (22:1)*	HEI-15, AMEI	**-0.27**	**0.06**	**1.35E-06**	**-0.41**	**0.05**	**2.57E-15**	**-0.46**	**0.05**	**2.84E-18**	**0.43**	**0.05**	**1.15E-16**	**-0.24**	**0.05**	**1.83E-05**	**0.25**	**0.06**	**1.09E-05**
cystine	HEI-15	**0.24**	**0.06**	**3.70E-05**	0.17	0.05	0.002	**0.33**	**0.05**	**1.27E-09**	**-0.2**	**0.05**	**1.44E-04**	-0.02	0.06	0.78	**-0.25**	**0.06**	**1.32E-05**
N-stearoyl-sphinganine (d	DASH	**0.24**	**0.06**	**2.35E-05**	**0.26**	**0.05**	**1.30E-06**	**0.34**	**0.05**	**6.2E-10**	**-0.3**	**0.05**	**7.52E-09**	0.11	0.06	0.05	**-0.25**	**0.06**	**1.59E-05**
guaiacol sulfate	HEI-15	**-0.24**	**0.06**	**5.78E-05**	-0.04	0.06	0.49	0.03	0.06	0.58	0.08	0.06	0.15	0.14	0.06	0.02	**0.25**	**0.06**	**1.84E-05**
adrenate (22:4n6)	HEI-15	0.11	0.06	0.05	0.07	0.05	0.18	0.09	0.06	0.1	-0.15	0.05	0.005	-0.05	0.06	0.39	**-0.23**	**0.06**	**4.45E-05**
(14 or 15)-methylpalmitat	AMED	0.15	0.06	0.01	0.06	0.06	0.32	0.11	0.06	0.05	-0.1	0.06	0.07	-0.08	0.06	0.15	-0.21	0.06	3.62E-04
pentose acid*	HEI-15	-0.15	0.07	0.03	-0.12	0.06	0.05	-0.02	0.06	0.72	0.16	0.06	0.01	0.02	0.06	0.76	0.23	0.06	4.00E-04
1-lignoceroyl-GPC (24:0)	HEI-15, DASH	-0.18	0.06	0.001	**-0.29**	**0.05**	**2.72E-08**	**-0.34**	**0.05**	**1.25E-10**	**0.33**	**0.05**	**7.06E-11**	-0.19	0.05	5.33E-04	0.2	0.06	4.18E-04
tartronate (hydroxymalon	HEI-15, DASH	-0.2	0.06	0.002	**-0.22**	**0.06**	**2.79E-04**	**-0.27**	**0.06**	**1.31E-05**	**0.24**	**0.06**	**6.74E-05**	-0.05	0.06	0.45	0.23	0.06	4.41E-04
sphingomyelin (d18:0/18:(	DASH	0.17	0.06	0.003	**0.2**	**0.05**	**2.32E-04**	**0.23**	**0.05**	**3.96E-05**	**-0.24**	**0.05**	**8.69E-06**	0.09	0.06	0.11	-0.2	0.06	6.28E-04
X-12726	HEI-15, DASH	-0.14	0.06	0.02	-0.16	0.05	0.003	-0.17	0.06	0.004	**0.22**	**0.05**	**6.56E-05**	-0.06	0.06	0.31	0.2	0.06	6.29E-04
X-21339	HEI-15	0.16	0.06	0.01	0.18	0.05	8.13E-04	0.18	0.06	0.001	**-0.2**	**0.05**	**1.84E-04**	0.05	0.06	0.32	-0.19	0.06	0.001
1-behenoyl-GPC (22:0)	HEI-15, DASH	-0.18	0.06	0.001	**-0.27**	**0.05**	**2.82E-07**	**-0.34**	**0.05**	**3.8E-10**	**0.31**	**0.05**	**3.05E-09**	-0.18	0.05	0.001	0.19	0.06	0.001
beta-sitosterol	AMED	-0.15	0.06	0.01	**-0.22**	**0.05**	**5.57E-05**	**-0.25**	**0.05**	**4.76E-06**	**0.24**	**0.05**	**1.02E-05**	-0.09	0.06	0.1	0.19	0.06	0.001
4-acetylphenol sulfate	DASH	-0.09	0.06	0.12	-0.05	0.06	0.37	-0.06	0.06	0.33	0.1	0.06	0.08	0.05	0.06	0.38	0.19	0.06	0.002
branched chain 14:0 dicarl	HEI-15, DASH	-0.15	0.06	0.01	**-0.2**	**0.06**	**2.72E-04**	-0.19	0.06	0.001	**0.23**	**0.05**	**4.58E-05**	-0.08	0.06	0.19	0.18	0.06	0.002
tartarate	HEI-15	-0.03	0.06	0.59	-0.14	0.06	0.03	-0.03	0.06	0.64	0.15	0.06	0.01	0.01	0.06	0.87	0.19	0.06	0.003
oxalate (ethanedioate)	HEI-15, DASH	-0.13	0.06	0.03	-0.17	0.06	0.002	-0.2	0.06	6.67E-04	0.18	0.06	0.001	-0.03	0.06	0.62	0.18	0.06	0.003
glycerate	HEI-15, DASH	-0.19	0.06	0.003	-0.2	0.06	5.41E-04	**-0.26**	**0.06**	**1.55E-05**	**0.22**	**0.06**	**1.42E-04**	-0.07	0.06	0.29	0.19	0.06	0.003
glycine conjugate of C10H	HEI-15	0.15	0.05	0.01	0.19	0.05	3.18E-04	**0.25**	**0.05**	**3.04E-06**	**-0.2**	**0.05**	**1.26E-04**	0.07	0.05	0.2	-0.16	0.06	0.003
X-12306	HEI-15, AMEI	-0.14	0.06	0.02	-0.09	0.06	0.13	-0.14	0.06	0.01	0.16	0.06	0.005	-0.01	0.06	0.9	0.18	0.06	0.004
1-nervonoyl-GPC (24:1n9)'	HEI-15, AMEI	-0.18	0.06	0.002	**-0.31**	**0.05**	**2.03E-08**	**-0.39**	**0.05**	**3.76E-12**	**0.32**	**0.05**	**2.35E-09**	-0.2	0.06	4.86E-04	0.17	0.06	0.004
tridecenedioate (C13:1-DC	AMED	0.12	0.06	0.06	-0.04	0.06	0.54	0.02	0.06	0.74	0.01	0.06	0.84	-0.15	0.06	0.01	-0.15	0.06	0.01
X-21736	HEI-15, DASH	0.06	0.06	0.3	0.04	0.06	0.45	0.12	0.06	0.04	-0.05	0.06	0.39	-0.09	0.06	0.12	-0.15	0.06	0.01
X-21842	HEI-15, DASH	-0.1	0.06	0.11	-0.07	0.06	0.23	-0.02	0.06	0.76	0.08	0.06	0.15	0.05	0.06	0.36	0.16	0.06	0.01
2,6-dihydroxybenzoic acid	DASH	-0.17	0.06	0.01	-0.06	0.06	0.3	-0.04	0.06	0.45	0.08	0.06	0.14	0.05	0.06	0.42	0.17	0.06	0.01
indolepropionate	HEI-15, DASH	-0.15	0.06	0.01	-0.15	0.06	0.01	-0.2	0.06	8.51E-04	0.17	0.06	0.002	-0.04	0.06	0.45	0.16	0.06	0.01
S-methylmethionine	AMED	-0.12	0.06	0.03	-0.16	0.05	0.004	-0.18	0.06	0.002	0.16	0.05	0.003	-0.04	0.06	0.54	0.14	0.06	0.01
androstenediol (3beta,17b	DASH	0.17	0.07	0.01	0.11	0.06	0.09	0.13	0.06	0.05	-0.1	0.06	0.1	-0.03	0.06	0.68	-0.16	0.07	0.01
1-palmitoyl-2-arachidonoy	DASH	0.18	0.06	0.002	0.07	0.05	0.18	0.15	0.06	0.01	-0.13	0.05	0.01	0	0.06	1	-0.16	0.06	0.01
X-21752	HEI-15, DASH	-0.09	0.06	0.14	-0.14	0.06	0.01	-0.13	0.06	0.03	0.15	0.06	0.01	-0.04	0.06	0.45	0.14	0.06	0.02
1-stearoyl-2-docosapenta(	AMED	0.12	0.06	0.05	0.13	0.06	0.02	0.18	0.06	0.002	-0.15	0.06	0.01	0.04	0.06	0.48	-0.14	0.06	0.02
3,5-dichloro-2,6-dihydroxy	HEI-15, AMEI	0.09	0.06	0.12	0.17	0.05	0.001	0.18	0.05	0.001	-0.17	0.05	0.001	0.07	0.05	0.18	-0.12	0.06	0.03
N-delta-acetylornithine	HEI-15, AMEI	-0.13	0.06	0.03	-0.04	0.06	0.55	-0.02	0.06	0.79	0.08	0.06	0.17	0.03	0.06	0.61	0.13	0.06	0.04
X-11315	HEI-15, DASH	-0.05	0.06	0.44	-0.1	0.06	0.1	-0.09	0.06	0.12	0.09	0.06	0.11	0.01	0.06	0.88	0.13	0.06	0.04
X-26062	DASH	-0.08	0.06	0.2	-0.16	0.06	0.01	-0.15	0.06	0.01	0.17	0.06	0.002	-0.08	0.06	0.16	0.12	0.06	0.05
heptenedioate (C7:1-DC)*	HEI-15, AMEI	0.08	0.06	0.16	0.07	0.06	0.24	0.14	0.06	0.02	-0.07	0.06	0.21	-0.02	0.06	0.73	-0.11	0.06	0.06
ethyl alpha-glucopyranosi(	DASH	-0.04	0.06	0.52	**-0.2**	**0.06**	**2.73E-04**	-0.2	0.06	6.65E-04	0.2	0.05	4.39E-04	-0.12	0.06	0.04	0.11	0.06	0.07
3-bromo-5-chloro-2,6-dihy	AMED	0.07	0.06	0.24	0.11	0.05	0.04	0.1	0.05	0.07	-0.1	0.05	0.05	0.04	0.05	0.51	-0.09	0.06	0.13
X-17351	HEI-15, DASH	-0.14	0.06	0.02	-0.04	0.06	0.53	-0.02	0.06	0.72	0.06	0.06	0.29	0.02	0.06	0.78	0.09	0.06	0.14
4-methoxyphenol sulfate	DASH	0.02	0.06	0.74	-0.05	0.06	0.38	-0.06	0.06	0.32	0.08	0.06	0.17	-0.02	0.06	0.72	0.09	0.06	0.15
carotene diol (2)	HEI-15, AMEI	0.06	0.06	0.37	-0.2	0.06	6.79E-04	-0.06	0.06	0.32	0.15	0.06	0.01	-0.09	0.06	0.14	0.09	0.06	0.17
1-(1-enyl-oleoyl)-2-docosa	AMED	-0.09	0.06	0.17	-0.19	0.06	0.002	-0.17	0.06	0.01	0.18	0.06	0.002	-0.12	0.06	0.06	0.08	0.07	0.21
beta-cryptoxanthin	HEI-15, DASH	-0.01	0.06	0.85	-0.18	0.06	0.001	-0.05	0.06	0.41	0.15	0.06	0.01	-0.1	0.06	0.1	0.07	0.06	0.23
pyridoxate	DASH	-0.1	0.06	0.1	-0.02	0.06	0.75	0.03	0.06	0.66	0.06	0.06	0.26	-0.02	0.06	0.78	0.06	0.06	0.36
N-methylproline	DASH	0	0.06	0.95	0.02	0.05	0.76	0.06	0.05	0.25	-0.03	0.05	0.59	0.05	0.05	0.37	0.02	0.06	0.78
carotene diol (1)	HEI-15, AMEI	0.1	0.06	0.1	-0.14	0.06	0.02	0.02	0.06	0.75	0.09	0.06	0.13	-0.09	0.06	0.14	0.01	0.06	0.84
1-(1-enyl-oleoyl)-2-docosa	AMED	0.08	0.06	0.22	-0.08	0.06	0.18	0.01	0.06	0.92	0.05	0.06	0.36	-0.06	0.06	0.31	-0.01	0.06	0.88
X-12680	DASH	0	0.06	0.97	0.06	0.06	0.29	0.11	0.06	0.05	-0.02	0.06	0.73	0.01	0.06	0.87	0	0.06	1

Abbreviations : AMED: Meditteranean-style diet; DASH: Dietary Approaches to Stop Hypertension; HEI-15: Healthy Eating Index-2015

Note : All models controlled for age, sex, race, physical activity, education level, energy intake (kilocalories per day; kcal/day) and smoking status

Note : Significant associations (P<3.0*10-4 after a Bonferonni correction) in bold

Supplementary Table 6: Pearson Correlations Between Diet Score, amd Diet-Specific Metabolite Summary Scores

Supplementary Table 6: Pearson Correlations Between Diet Score, amd Diet-Specific Metabolite Summary S

	Dietary Intake			Metabolite Summary Scores		
	HEI	DASH	AMED	HEI	DASH	AMED
Dietary Intake						
HEI	-	$<2.0*10^{-16}$	$<2.0*10^{-16}$	$<2.0*10^{-16}$	$<2.0*10^{-16}$	$<2.0*10^{-16}$
DASH	0.62	-	$<2.0*10^{-16}$	$<2.0*10^{-16}$	$<2.0*10^{-16}$	$<2.0*10^{-16}$
AMED	0.61	0.51	-	$<2.0*10^{-16}$	$<2.0*10^{-16}$	$<2.0*10^{-16}$
Metabolite Summary Scores						
HEI	0.63	0.5	0.45	-	$<2.0*10^{-16}$	$<2.0*10^{-16}$
DASH	0.59	0.57	0.43	0.93	-	$<2.0*10^{-16}$
AMED	0.58	0.38	0.48	0.9	0.8	-

Note : Pearson correlations below the diagonal, P-values above the diagonal.

Abbreviations : AMED: Mediterranean style diet; DASH: Dietary Approaches to Stop Hypertension

HEI: Healthy Eating Index;

Supplementary Table 7: Standardized parameter estimates for associations between six glucose and insulin homeostasis traits with scores representing adherence to each of three healthy diet patterns: the Healthy Eating Index (HEI), the Dietary Approaches to Stop Hypertension (DASH), and a Mediterranean-Style Diet (AMED), and with Metabolite Summary Scores (MSSs) for the same dietary patterns, when additionall controlling for BMI

Supplementary Table 7: Standardized parameter estimates for associations between six glucose and insulin homeostasis traits with scores representing adherence to each of three healthy diet patterns: the Healthy Eating Index (HEI), the Dietary Approaches to Stop Hypertension (DASH), and a Mediterranean-Style Diet (AMED), and with Metabolite Summary Scores (MSSs) for the same dietary patterns, when additionall controlling for BMI

	HEI			DASH			AMED		
	β	SE	P	β	SE	P	β	SE	P
Fasting glucose									
Diet score	**-0.15**	**0.06**	**0.01**	-0.17	0.06	0.005	-0.12	0.06	0.03
MSS	**-0.25**	**0.06**	**1.00E-04**	**-0.24**	**-0.06**	**1.00E-04**	**-0.17**	**0.06**	**0.01**
Fasting insulin									
Diet score	-0.08	0.05	0.11	-0.03	0.05	0.52	-0.02	0.05	0.97
MSS	**-0.16**	**0.05**	**3.00E-03**	-0.1	0.05	0.05	**-0.15**	**0.05**	**0.005**
C-peptide									
Diet score	-0.08	0.05	0.09	-0.05	0.05	0.28	-0.04	0.05	0.38
MSS	**-0.17**	**0.05**	**0.001**	**-0.13**	**0.05**	**0.01**	**-0.16**	**0.05**	**0.003**
Insulin secretion									
Diet score	-0.003	0.06	0.96	0.02	0.06	0.78	0.06	0.05	0.29
MSS	0.03	0.06	0.66	0.06	0.06	0.34	-0.02	0.06	0.74
Insulin sensitivity									
Diet score	0.11	0.05	0.03	0.09	0.05	0.08	0.03	0.05	0.51
MSS	**0.24**	**0.05**	**8.60E-06**	**0.18**	**0.05**	**4.50E-04**	**0.2**	**0.05**	**1.10E-04**
Disposition Index									
Diet score	0.15	0.06	0.02	0.12	0.06	0.04	0.1	0.06	0.08
MSS	0.33	0.06	4.10E-07	0.29	0.06	2.40E-06	0.23	0.06	3.30E-04

Abbreviations : AMED: Mediterranean Style diet, DASH: Dietary Approaches to Stop Hypertension; HEI: Healthy Eating Index (2015 version); MSS: Metabolite summary score

Note: All models controlled for age, sex, race, physical activity, education level, energy intake (kilocalories per day; kcal/day), smoking status and BMI

Note: Significant associations (P<.01 after a Bonferroni correction for the effective number of independent tests) in bold.

**Note:* Partial r-squared representing the variance in outcome explained by the predictor, when controlling for age, sex, race, physical activity, education level, energy intake (kilocalories per day; kcal/day) and smoking status

Supplementary Figure 1

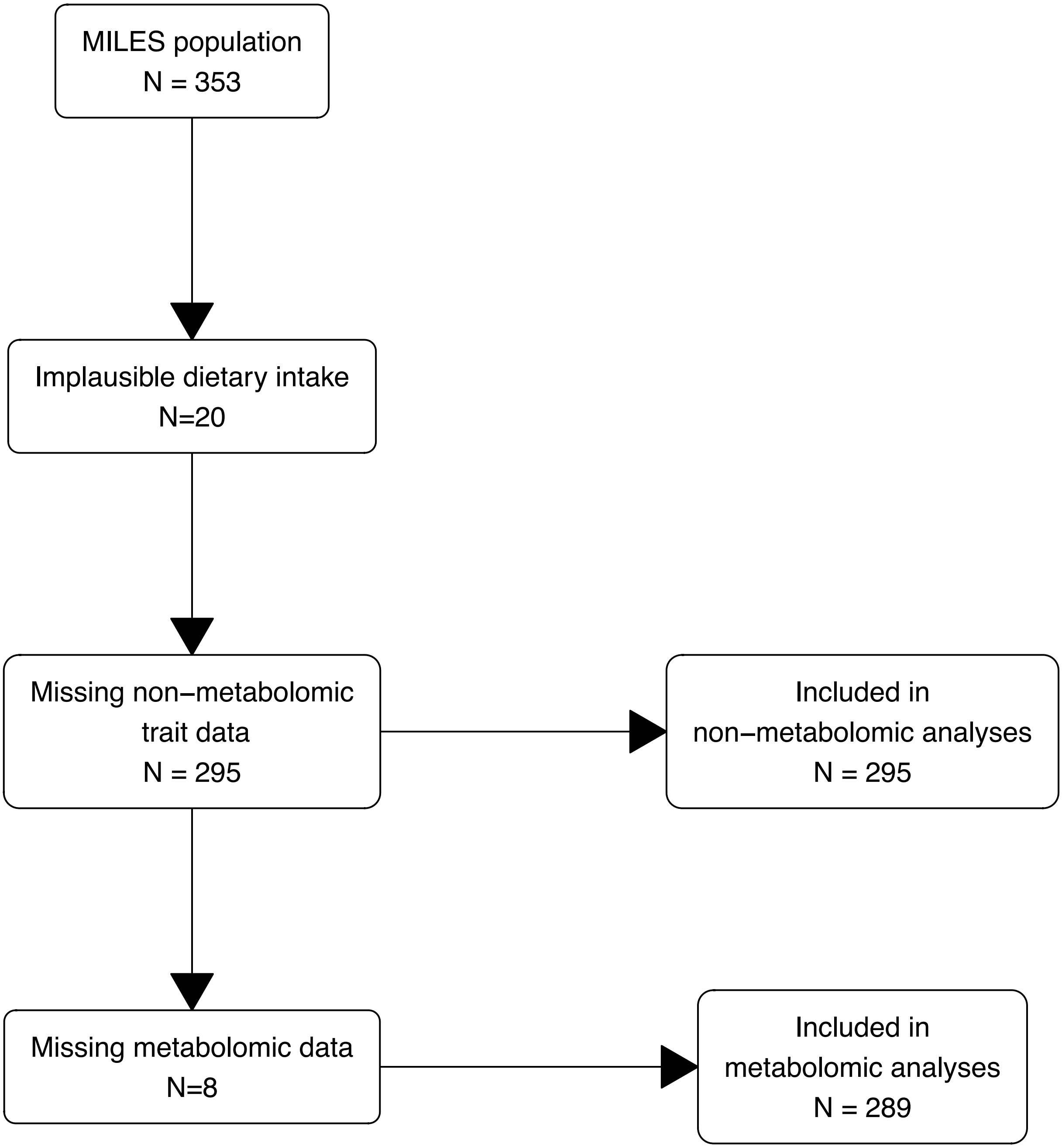

Supplementary Figure 2

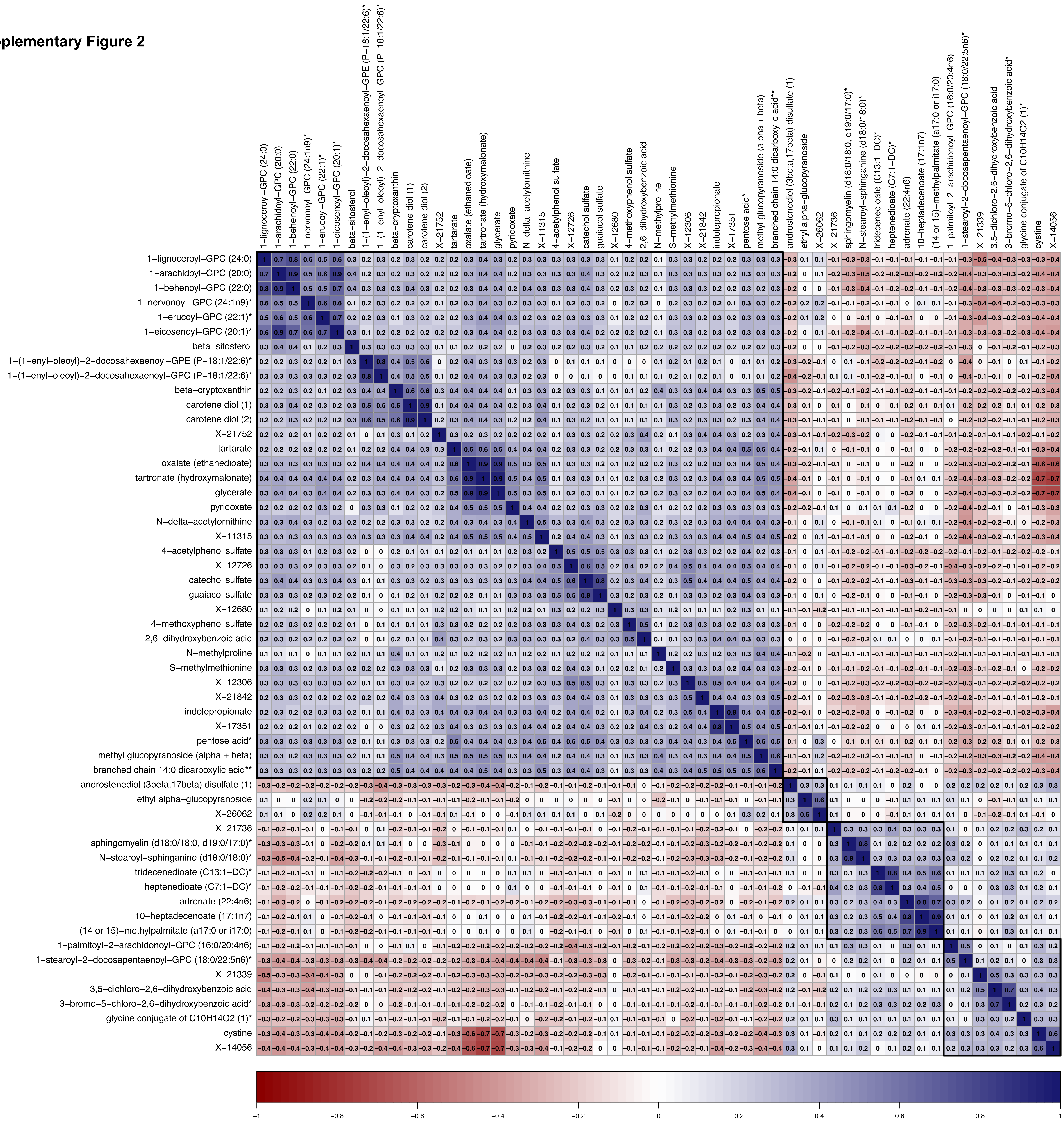

Supplement: Multimedia component 1 [file mmc1.pdf]
